# Effect and prognostic value of physical activity for lower-extremity complications of type 2 diabetes across the adult lifespan: a national cohort study using Swedish registers

**DOI:** 10.1136/bmjopen-2025-114759

**Published:** 2026-11-11

**Authors:** Thomas Vogt, Beatrice Kennedy, Emerald G Heiland, Liisa Byberg, Karl Michaëlsson, Hannah L Brooke

**Affiliations:** 1 Surgical Sciences, Uppsala University, Uppsala, Sweden; 2 Medical Sciences, Uppsala University, Uppsala, Sweden

**Keywords:** REGISTRIES, Lower Extremity, Diabetes Mellitus, Type 2, Exercise, Prognosis, Diabetic foot

## Abstract

**Abstract:**

**Objectives:**

We aimed to determine the effect of physical activity on the risk of later developing lower-extremity complications and its prognostic value for these complications across the adult lifespan of individuals with type 2 diabetes.

**Design:**

A register-based cohort study.

**Setting:**

Sweden, 2007–2019.

**Participants:**

All adults with newly diagnosed type 2 diabetes with ≥1 record in the National Diabetes Register (n=372 317).

**Outcome measures:**

We employed a landmarking approach with a 5-year follow-up and Fine-Gray subdistribution hazards models to investigate the relationships between physical activity and peripheral neuropathy/peripheral vascular disease, foot ulceration and lower-extremity amputations throughout the adult lifespan, adjusting for standard clinical covariates. We compared the performance of multivariable models with and without physical activity using cross-validation.

**Results:**

During follow-up, 85 203 (24.69%) experienced peripheral neuropathy or peripheral vascular disease, 2958 (0.81%) received hospital-based care for foot ulcers and 1669 (0.45%) underwent amputation. Low physical activity was independently associated with a higher 5-year incidence of lower-extremity complications across a wide range of ages. Between ages 47 and 78, never walking for 30 min (or equivalent) during a week was associated with a 1.17 (CI 1.07 to 1.27) to 1.52 (1.32 to 1.75) times greater subdistribution hazard of peripheral neuropathy/peripheral vascular disease—compared with daily walking. Age-specific estimates ranged from 1.36 (0.82 to 2.27) to 3.37 (2.30 to 4.93) for foot ulcers (ages 54–87) and from 1.65 (0.95 to 2.86) to 3.93 (2.22 to 6.98) for amputation (ages 69–85). When comparing the performance of multivariable models with and without physical activity using cross-validation, the addition of physical activity to other clinical variables did not improve model performance.

**Conclusions:**

Low physical activity increased the incidence of lower extremity complications across a wide age range in individuals with type 2 diabetes, with higher estimates for more severe complications. However, it did not enhance the prediction of these complications when standard clinical risk factors were taken into account.

**STRENGTHS AND LIMITATIONS OF THIS STUDY**

- Nationwide data on physical activity among a large number of individuals with type 2 diabetes, linked to other Swedish registers, with nearly no loss to follow-up (most of the censoring was administrative).
- Use of a landmarking approach to assess whether self-reported physical activity was associated with lower-extremity complications at all adult ages.
- To assess the prognostic value of physical activity, calculations of measures of predictive performance used bootstrap cross-validation to prevent overfitting.
- Peripheral neuropathy/peripheral vascular disease and foot ulcers might be subject to misclassification, but this is not a concern for amputations.
- Residual confounding may subsist, and some variables used are subject to self-reporting bias (physical activity and smoking status).

## Introduction

 Individuals with type 2 diabetes are at risk of lower-extremity complications, including peripheral neuropathy and peripheral vascular disease, which can lead to foot ulcers and amputation. Those complications can affect quality of life^1 2^ and are associated with high mortality and reduced life expectancy, especially for their most severe manifestations.^2–5^ As the prevalence of type 2 diabetes is expected to rise in Sweden and globally in the coming decades,^6 7^ more individuals are likely to experience diabetic-related complications.

Physical activity is recommended for the management of type 2 diabetes^8^ since it is known to reduce the risk of mortality and cardiovascular complications.^9^ However, very few studies have examined whether physical activity can prevent the occurrence of lower extremity complications.^10^ Nonetheless, one previous study in 18 092 individuals from the UK Biobank found that, compared with individuals performing no leisure-time physical activity, even low levels of physical activity were associated with a 30% reduced hazard of neuropathy identified from hospital inpatient records.^11^ A recent Swedish study also found low physical activity to be associated with two times the hazard of lower limb amputation.^12^ However, it is unclear if this would be the case for any diagnosed neuropathy (inpatient or outpatient diagnosed) and for other severe lower extremity complications such as foot ulcers. Moreover, it is unclear how physical activity impacts the risk of complications at different ages.

In addition to physical activity’s potential to reduce the risk of later lower extremity complications, it is also a candidate factor for improving the prediction of these complications. Lower-extremity complications affect the feet first, so low physical activity levels could be an early indicator of their emergence—similar to how pre-frailty and frailty predict future microvascular complications.^13^ Further, because physical activity can be reported repeatedly over time, it could represent a time-updated and independent indicator of prognosis. Identifying factors that improve the prediction of lower-extremity complications is useful for clinical practice,^14^ to identify high-risk individuals and facilitate subsequent prevention of serious morbidity.

In this study, we aimed to determine if low physical activity confers a higher future risk of mild and severe lower-extremity complications of type 2 diabetes, including those identified in primary care and outpatient specialist care. Moreover, we aimed to determine the independent prognostic value of self-reported physical activity for lower-extremity complications of type 2 diabetes.

## Methods

### Study population

We constructed a cohort based on all adults (≥18 years old) newly diagnosed with type 2 diabetes in Sweden in 2007–2019, with at least one record in the National Diabetes Register (NDR). The NDR contains information collected in primary and outpatient specialist care on individuals with any type of diabetes and has attained a high coverage.^15^ Coverage increased steadily from 50% in 2007 to 90% in 2011–2012,^16–21^ with similar coverage for different diabetes types at least since the early 2010s.^19–22^ We identified those with diagnosed type 2 diabetes based on clinical assessment, as it was recorded by clinicians in the variable for ‘diabetes type’ in the NDR. We used the personal identification number allocated to all inhabitants of Sweden to link data from the NDR to several nationwide registers: the Patient Register, which captures hospital-based inpatient and outpatient care^23^; the Prescribed Drug Register^24^; the Cause of Death Register^25^; the Total Population Register^26^ and the Longitudinal Integrated Database for Health Insurance and Labour Market Studies (LISA).^27^

We combined data from the NDR, the Patient Register and the Prescribed Drug Register to determine the date of diagnosis of type 2 diabetes (see the Variables section in the online supplemental file 1 for details). The flowchart (online supplemental figure S1) shows the study populations used for each outcome. We only included individuals who had immigrated to Sweden if they were diagnosed with type 2 diabetes at least 365 days after immigrating. For each outcome, we excluded individuals who already had the outcome when diagnosed with type 2 diabetes or during a prespecified washout period (online supplemental figure S2a), resulting in the following populations at risk: peripheral neuropathy/peripheral vascular disease (washout period 120 days): n=345 133; foot ulceration (washout period 90 days): n=365 398; amputation (no washout period): n=371 779; major amputation (no washout period): n=371 997). We determined the length of the washout period by graphically examining the overall incidence of all four outcomes following the diagnosis of type 2 diabetes (online supplemental figure S2b–d).

### Self-reported physical activity and other prognostic factors

Information on self-reported physical activity was retrieved from the NDR, where it was recorded at each appointment in primary care and outpatient specialist care as the frequency of 30 min walking or equivalent during a week: (1) never, (2) <1 times/week, (3) regularly 1–2 times/week, (4) regularly 3–5 times/week and (5) daily.

From the NDR, we retrieved or derived the following known risk factors to be considered in statistical analyses: sex, age at diagnosis of type 2 diabetes, BMI, HbA1c, smoking status, systolic blood pressure, ratio of total to high-density lipoprotein cholesterol, past cardiovascular event(s), time since last healthcare visit and existing peripheral neuropathy/peripheral vascular disease (described further in online supplemental file 1: Variables section). These variables were chosen based on an assessment of the literature, primarily in the Swedish context and on clinicians’ expertise, to be a fair representation of prognostic factors commonly used in clinical practice to predict the risk of lower-extremity complications in individual patients.

We planned subgroup analyses by sex (male/female) and educational attainment. The latter was obtained from LISA and categorised as (1) high education: secondary education or more and (2) low education: completion of pre-secondary education at most (described further in online supplemental file 1: Variables section).

### Outcomes

We examined four outcomes: peripheral neuropathy/peripheral vascular disease, foot ulceration, amputation and major amputation (as specified in online supplemental file 1: Variables section, Outcomes). We only included non-traumatic amputations. We included major amputation as an outcome in addition to (all) lower-extremity amputations to assess whether the results were sensitive to the severity of the amputations. Major amputations were amputations performed above the ankle and could include, for example, transtibial amputations and transpelvic amputations. Peripheral neuropathy or peripheral vascular disease was a composite outcome, as it is reported in the NDR. The other outcomes were extracted from the Patient Register, that is, hospital-based inpatient and outpatient specialist care. We considered only incident events occurring after diagnosis of type 2 diabetes (and after a washout period if relevant).

### Statistical analysis

#### Landmarking

We used a landmarking approach to take advantage of repeated measurements available for individuals in the NDR and LISA and to analyse different ages separately.^28^ We divided the data into landmark datasets, where each landmark dataset was characterised by a baseline age (19–100 years old) and a pre-specified follow-up window of 5 years, resulting in 82 landmark datasets per outcome. At each landmark age, we included individuals already diagnosed with type 2 diabetes who had not experienced the event of interest or a competing event and had not been censored (figure 1). A given individual could therefore be included in several landmark datasets if those conditions were met for several ages. Individuals could be included at most once in any given landmark dataset, and each landmark dataset was analysed separately (there was no clustering by individual in any analysis). We used the most recent value of the variables described above at each landmark age.

**Figure 1:**
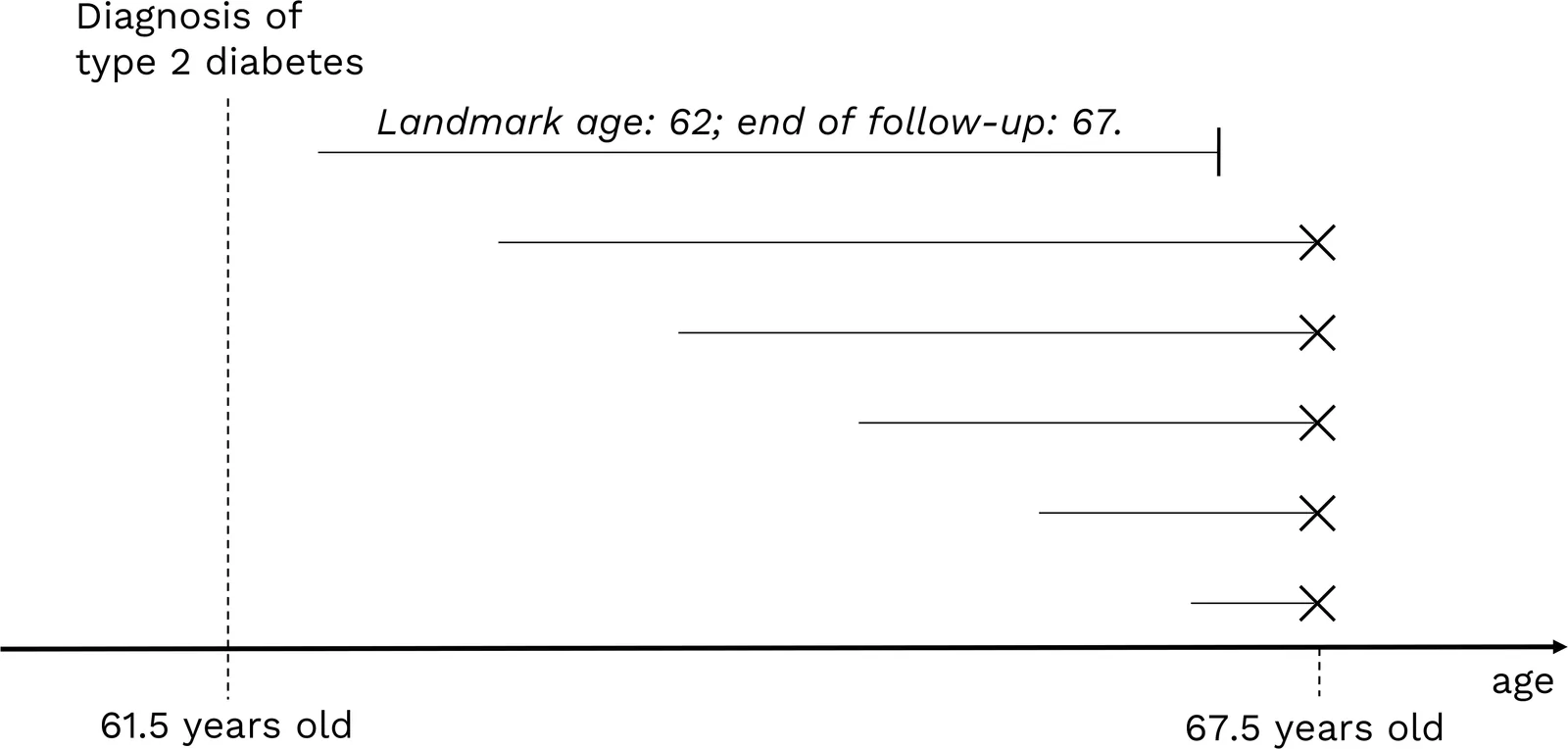
Depiction of landmarking for a hypothetical individual diagnosed with type 2 diabetes when 61.5 years old and who experienced the event of interest when 67.5 years old. Since landmark datasets in this study were characterised by a landmark age between 19 and 100 years old and a pre-specified follow-up window of 5 years, the individual depicted in the figure would be included in six landmark datasets: at age 62, 63 and so on, until age 67. From age 62, the individual would be followed until age 67, from age 63, until 68 and so on and would be censored if no event occurred. At landmark age 62, the individual above would be censored at age 67. At landmark age 67, the individual would only be followed for 6 months before experiencing the event of interest.

#### Descriptive statistics

We calculated the total follow-up time and the proportion of the study population that was censored, experienced the event of interest and died event-free, (1) in the entire study population before creating landmark datasets and (2) for each landmark dataset. Death was considered a competing event, while emigration out of Sweden and the 31 December 2019 were considered censoring events. We also computed descriptive statistics for the variables we planned to include in statistical models, comparing response and complete case samples.

#### Multiple imputation

The proportion of missing values was between 0 and 20% in 88% of variables across all outcome-landmark age combinations (online supplemental table 1.3). We applied multiple imputation by chained equations^29^ to handle missing data as described in online supplemental file 1: Multiple imputation. Multiple imputations were only performed on landmark datasets where 10 or more events per variable were observed.^30^

We used the abovementioned variables (self-reported physical activity, other prognostic factors and outcomes) when imputing data. We also added the following variables: educational attainment, calendar year, whether the individual was born in Sweden or not, disposable individual income from the previous year, the number of days spent unemployed during the last year and civil status of the prior year (see online supplemental file 1: Multiple Imputation section). Those variables were obtained from LISA, except for the calendar year, which was derived from the NDR. To help demonstrate the robustness of the study results to the choice of missing data methods, we additionally conducted complete case analyses.

#### Statistical modelling: survival analysis and prediction

First, we fitted three survival (Fine-Gray subdistribution hazards) models for each outcome-landmark age combination. Model 1 only included self-reported physical activity. Model 2 included all prognostic factors described above except physical activity. It therefore included sex, age at diagnosis of type 2 diabetes, BMI, HbA1c, smoking status, systolic blood pressure, ratio of total to high-density lipoprotein cholesterol, past cardiovascular event(s) and time since last healthcare visit. If the outcome was foot ulceration or amputation, we also included peripheral neuropathy/peripheral vascular disease. Model 3 incorporated physical activity and the variables from model 2.

We calculated subdistribution HR (sHR) and their 95% CIs using Fine-Gray subdistribution hazards models,^31^ which account for the competing risk of death and are better suited for prognosis-related research questions.^32^

Second, we repeated the model fitting steps described above separately for males and females, as well as for individuals with low and high education levels. We calculated the difference in log-sHR and their associated 95% CI^33^ between males and females and between those with low and high education levels.

Third, for the prediction of complications, we calculated time-dependent Brier scores^34^ and time-dependent area under the receiver operating characteristic curves (AUC)^35^ for model 2 and model 3, at 5 years of follow-up. The Brier score quantifies the difference between the event status of the individuals in the study and their model-estimated risk of the event. The AUC assesses a model’s ability to distinguish between individuals who will experience an event and those who will not by a certain time point.^36^ We also calculated the change in Brier score and AUC from model 2 to model 3. To limit the risk of overfitting, we applied bootstrap cross-validation (500 repetitions) and repeatedly fitted models on training data while assessing model performance on validation data. In each of the 500 repetitions, the training data was obtained by sampling with replacement from the landmark dataset. The validation data consisted of all individuals not included in the training data. To limit the computational burden, we calculated the Brier scores and AUCs only on a randomly chosen imputed dataset for each outcome-landmark age combination (see online supplemental file 1: Bootstrapping).

Data processing and analyses were conducted with R V.4.1.1 and 4.2.1 (R Foundation for Statistical Computing).^37^ The online supplemental file 1 (see Software section) provides more details about the software used. We adhered to the Strengthening the Reporting of Observational Studies in Epidemiology (STROBE) reporting guidelines.

### Patient and public involvement statement

Patients or the public were not involved in the design, or conduct, or reporting or dissemination plans of our research.

## Results

In this population of individuals newly diagnosed with type 2 diabetes, the cumulative incidence of peripheral neuropathy/peripheral vascular disease was 85 203 (24.69%), hospital care for foot ulcers was 2958 (0.81%) and amputation was 1669 (0.45%), over a maximum follow-up time of 13 years prior to landmarking (table 1). As expected, the incidence of complications varied considerably by landmark age. Unsurprisingly, there were few to no events among the youngest age groups (Supplement: online supplemental table 1.2). Self-reported physical activity varied by age. For example, in the study population for amputation, 13.1% of individuals aged 60 reported never walking for 30 min or equivalent during a week, while this number was 17.1% at age 80 and 48.7% at age 100 (online supplemental table 1.3).

**Table 1:** Summary of follow-up time by outcome and proportions of individuals who were censored, who experienced the event or who died event-free (in the entire study population before landmarking was performed)

Outcome	N	Total follow-up time (years)	Median (range))follow-up time (years)	Censored	Experienced the event	Died event-free
Peripheral neuropathy/peripheral vascular disease	345 133	1 813 372	4.6 (0.33–13)	225 628 (65.37%)	85 203 (24.69%)	34 302 (9.94%)
Foot ulceration	365 398	2 206 275	5.71 (0.25–13)	311 819 (85.34%)	2958 (0.81%)	50 621 (13.85%)
Amputation	371 779	2 209 786	5.61 (0–13)	318 638 (85.71%)	1669 (0.45%)	51 472 (13.84%)
Major amputation	371 997	2 212 749	5.62 (0–13)	319 183 (85.80%)	1055 (0.28%)	51 759 (13.91%)

Overall, we found that lower levels of self-reported physical activity were associated with a higher subdistribution hazard of lower-extremity complications compared with daily physical activity across the adult lifespan, with larger point estimates for the more severe outcomes. Never (vs daily) walking 30 mins/week was associated with 1.17 (95% CI 1.07 to 1.27) to 1.52 (95% CI 1.32 to 1.75) times greater subdistribution hazard of peripheral neuropathy/peripheral vascular disease between ages 47 and 78 years, in models adjusted for clinically relevant variables, that is, model 3 (figure 2A). Age-specific estimates for foot ulcers ranged from 1.36 (95% CI 0.82 to 2.27) to 3.37 (95% CI 2.30 to 4.93) between ages 54 and 87 years. Age-specific estimates for amputation ranged from 1.65 (95% CI 0.95 to 2.86) to 3.93 (95% CI 2.22 to 6.98) between ages 69 and 85 years (figure 2B,C). To give a specific example, at landmark age 70, reporting never walking for 30 min (or equivalent) during a week was associated with a 1.4 (95% CI 1.3 to 1.5) times greater subdistribution hazard of peripheral neuropathy/peripheral vascular disease after adjustment for clinically relevant variables—compared with daily walking. The corresponding subdistribution HRs for foot ulcers, amputation and major amputation were 2.0 (95% CI 1.4 to 2.8), 2.3 (95% CI 1.4 to 3.8) and 2.5 (95% CI 1.4 to 4.4) (figure 2 and online supplemental figure S4).

**Figure 2:**
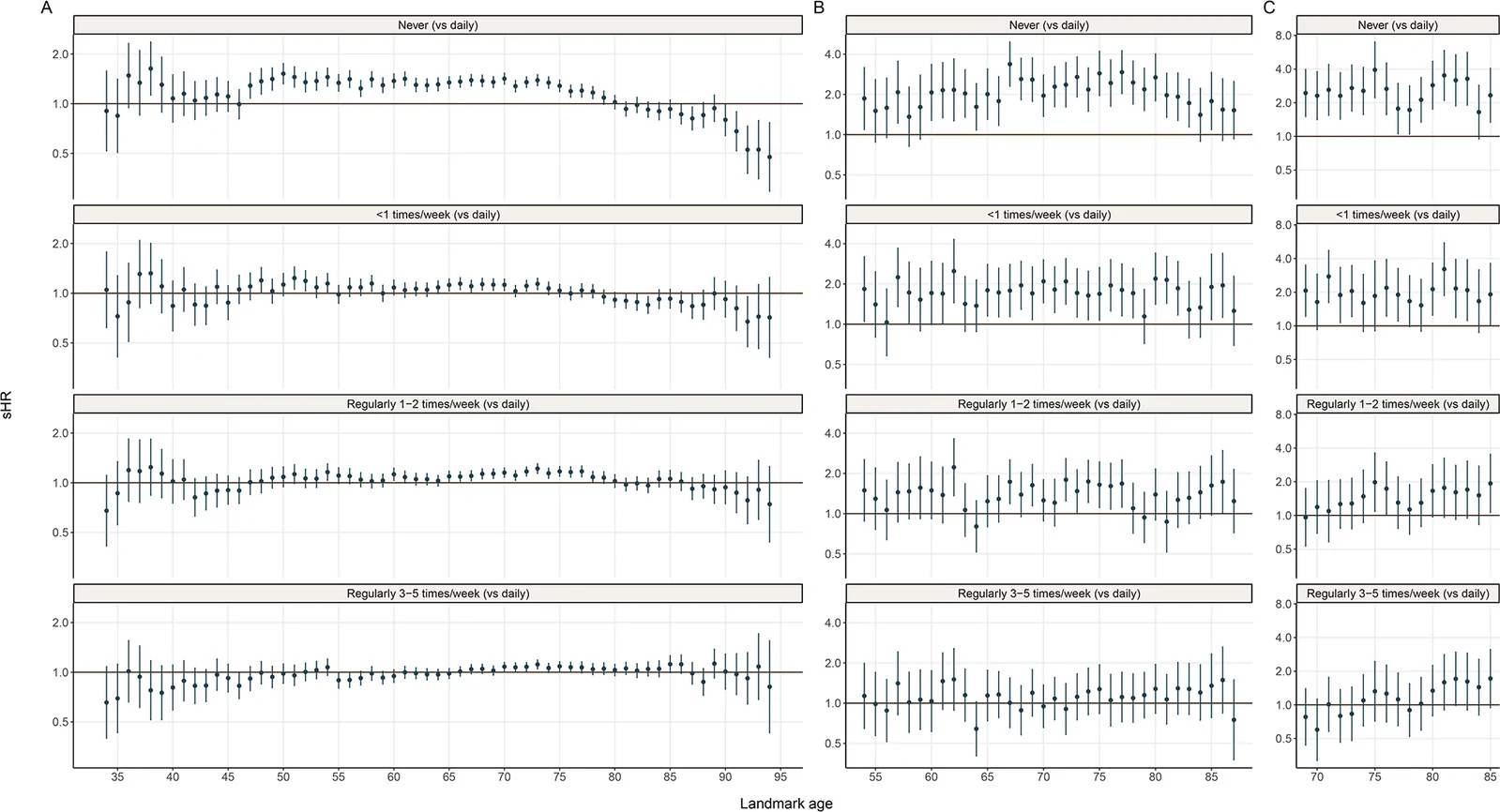
Association between level of self-reported physical activity and the subdistribution hazard of peripheral neuropathy/peripheral vascular disease (panel A), foot ulceration (**B**) and amputation (**C**), at different landmark ages, with associated 95% CI. Each physical activity category is compared with the highest category—daily walking of 30 min or equivalent, adjusted for the clinical prognostic factors (model 3). Due to the operationalisation of peripheral neuropathy/peripheral vascular disease, it is equivalent to the first occurrence of any of the outcomes (online supplemental file 1: Variables section). The y-axis is on a base-2 logarithmic scale. Results from multiply imputed data. sHR, subdistribution HR.

The four outcomes showed similar patterns in models including only physical activity (model 1: online supplemental figure S3). A large number of events allowed us to consider the risk of peripheral neuropathy/peripheral vascular disease at younger and older ages. Still, there was no consistent association with physical activity when the landmark age was below 47 or above 78 years old (figure 2). We also found no difference between the second-highest and the highest levels of self-reported physical activity for all outcomes (figure 2).

The association between physical activity and lower extremity complications was similar in males and females (online supplemental figure S5) and also similar in individuals with low and high education level (online supplemental figure S6).

Finally, we did not find evidence of improvements in model performance that resulted from considering physical activity in addition to the other prognostic factors, and we did not see differences by landmark age (figure 3, online supplemental figures S7–S10). For example, at landmark age 70, the change in Brier Score comparing model 3 to model 2 was −1.54e-4 (95% CI −4.13e-4 to 1.71e-4) for peripheral neuropathy/peripheral vascular disease. More details about predictive performance for model 2 and model 3 separately can be found in the Supplement (online supplemental figures S7–S10).

**Figure 3:**
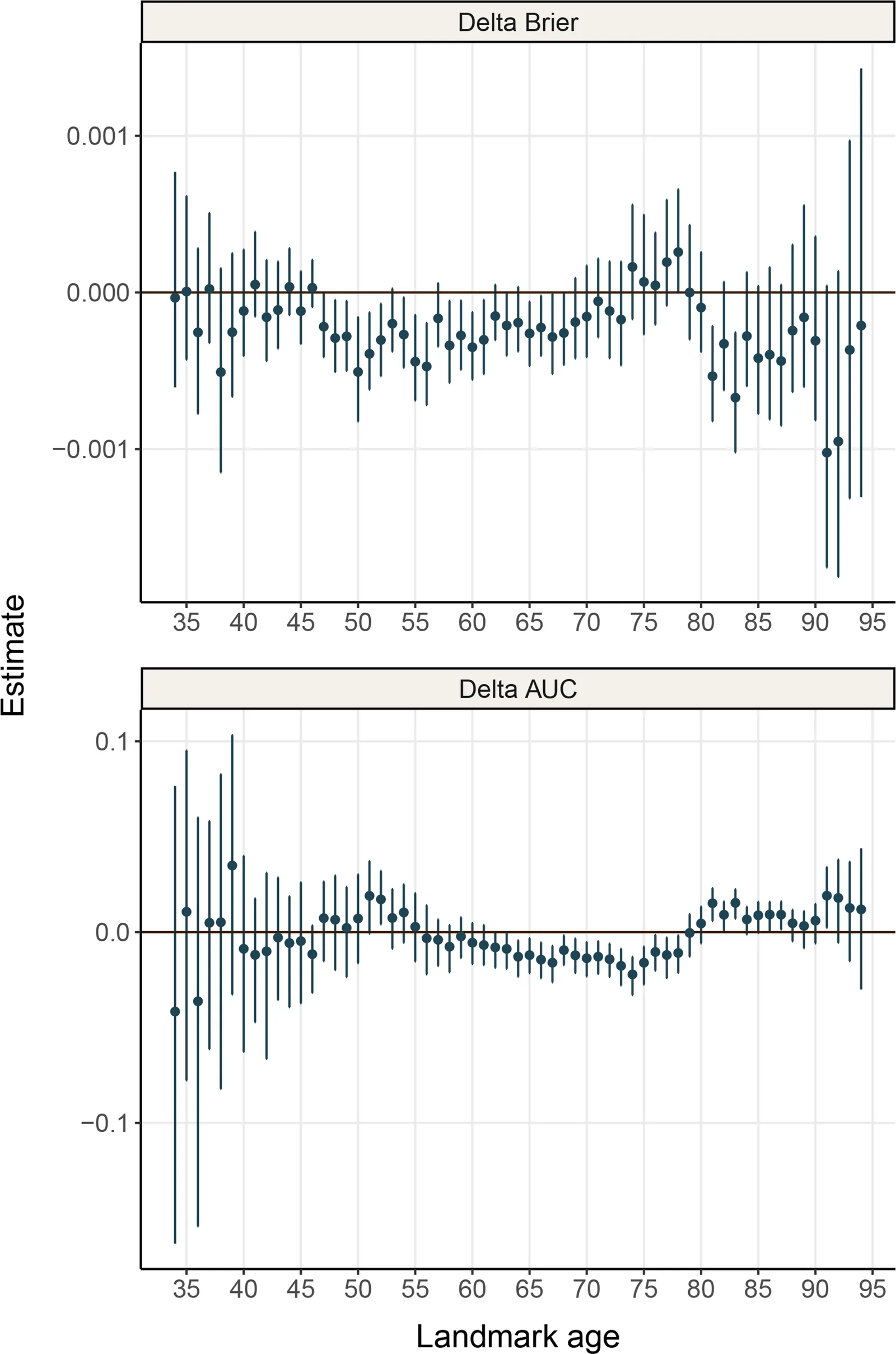
Comparison of time-dependent area under the receiver operating characteristic curve and time-dependent Brier score, between models without (model 2) and with physical activity (model 3) for different landmark ages, at 5 years of follow-up, with 95% CI. The results are for peripheral neuropathy/peripheral vascular disease (equivalent to the first occurrence of any of the outcomes, see online supplemental file 1: Variables section). If delta AUC or delta Brier is 0, it indicates that there is no change in discrimination or in performance when adding physical activity into a model that already includes the other prognostic factors. AUC, area under the receiver operating characteristic curves.

The results for complete case analyses were similar and lent themselves to the same conclusions as results using multiply imputed data.

## Discussion

Using a landmarking approach in data from Swedish national registers, with repeated assessment of physical activity and established prognostic factors, we found that lower self-reported physical activity was associated with a higher incidence of lower extremity complications, including complications treated in primary care and outpatient specialist care, across a wide range of ages in individuals with type 2 diabetes, even after controlling for clinically relevant variables and further stratifying by educational attainment. A higher relative hazard rate was observed for the more severe outcomes, ulcers and lower limb amputations. However, we did not find evidence that self-reported physical activity would improve the identification of individuals at high risk of lower-extremity complications.

Physical activity could reduce the risk of lower-extremity complications by improving glycaemic control and insulin resistance, blood pressure, nerve conduction velocity and nerve fibre density.^38 39^ However, very few studies have previously examined this potential association.^10^ Two previous studies in the UK Biobank found that low walking pace and not participating in leisure-time physical activity were associated with an increased risk of neuropathy identified from hospital inpatient records.^11 40^ Our results show that physical activity is also associated with neuropathy/peripheral vascular disease identified in primary care or outpatient specialist care and across a wide range of ages. Similarly to a previous study in Sweden, we also found that physical activity is associated with the incidence of lower extremity amputations.^12^ Importantly, while other studies used Cox proportional hazards models to investigate the association of physical activity with lower-extremity complications of type 2 diabetes,^11 12 40^ we used Fine-Gray subdistribution hazards models. That results are consistent across studies reinforces the strength of the inference on the role that physical activity might play in delaying or preventing lower-extremity complications of type 2 diabetes.

Of note, the associations of physical activity with neuropathy/peripheral vascular disease were not found among the oldest or youngest individuals with type 2 diabetes in Sweden, which could suggest that future interventions may be best targeted at those aged 45–80 years. However, we did not investigate associations over follow-up longer than 5 years, so it might be the case that young individuals with type 2 diabetes would benefit from today’s increased physical activity much later in life when it comes to lower-extremity complications. The small number of events at younger ages also did not allow the precise estimation of measures of association. In addition, in individuals older than 80, physical activity might still delay mortality^9^ even if it might not affect the risk of lower-extremity complications.

We hypothesised that self-reported physical activity could be of prognostic value for lower-extremity complications of type 2 diabetes because those complications affect the lower-extremities first, low self-reports of physical activity level could be early indicators of their emergence. However, our findings regarding model overall performance and discrimination suggest that self-reported physical activity would not improve the identification of individuals at high risk of lower-extremity complications of type 2 diabetes when indicators commonly used in clinical practice are already considered. Thus, while assessing physical activity level is of clinical importance as it can be a basis to provide recommendations to individuals with type 2 diabetes that could positively impact their health,^38 39^ in a Swedish context where other detailed clinical information is available, self-reported physical activity does not seem to have substantial clinical relevance for the specific task of identifying individuals at high risk of lower-extremity complications.

This distinction between the clinical importance of providing recommendations to improve individuals’ health and of identifying individuals at high risk of adverse events illustrates the differences between causal and prognostic factors. Although they can be conflated into ‘risk factors’, they cover different research goals that require different approaches.^41^ When evaluating a candidate prognostic factor, as in our study, determining that it is associated with the outcome(s) of interest is insufficient, especially in cases with large sample sizes where even small associations may be detected with precision. Instead, it is key to assess improvements in prediction to understand if the variable of interest could help identify high-risk individuals in clinical practice. In our study, self-reported physical activity was inversely associated with the risk of lower-extremity complications among individuals with type 2 diabetes. However, we detected no apparent improvement in overall prediction error (Brier score) or discrimination (AUC). We believe that distinguishing between prognostic and causal factors should be relevant to clinical management and diabetes research teams.

While we showed no clear improvement in prediction error or discrimination, self-reported physical activity might still be useful as a prognostic factor in settings with limited availability of prognostic information. However, this would need to be assessed in the relevant contexts.

### Strengths and limitations

The strengths of this study include access to nationwide data on physical activity among a large number of individuals with type 2 diabetes. Loss to follow-up was limited to a small number of individuals emigrating out of Sweden, and most of the censoring was therefore administrative censoring. Further, we relied on prospectively collected data, which ensured that the measurements of physical activity and of the outcomes were temporally ordered. In addition, we used multiply imputed data. This prevented a decrease in sample size and limited the potential for selection bias. Using a landmarking approach enabled us to assess whether self-reported physical activity was associated with lower-extremity complications at all ages. Finally, we supplemented survival analyses with calculations of measures of predictive performance using bootstrap cross-validation, comparing models that included our prognostic factor of interest to models that did not. This was desirable as looking solely at associations usually provides very little information about the capacity of a variable to help identify individuals at risk of adverse health outcomes.^42^

Nonetheless, some limitations apply. We could not include individuals with diagnosed type 2 diabetes who were not part of the NDR. Still, the NDR’s coverage is estimated to be high, with 87% of all individuals diagnosed with diabetes in Sweden in 2019,^15^ which limits the impact of this potential limitation. When determining the effect of physical activity on the risk of later developing lower-extremity complications, we could not adjust for variables like alcohol consumption, nutritional factors or genetic factors, since these data are not recorded in Swedish national registers, and some residual confounding is therefore possible. Physical activity was self-reported and can therefore suffer from self-reporting bias. As a socially desirable practice, it may be prone to overreporting. In addition, our measure of self-reported physical activity does not consider its intensity, duration or type. Whether self-reported or not, different physical activity measures, for example obtained through accelerometry-based technology, might provide additional prognostic value. Furthermore, foot ulcers and peripheral neuropathy/peripheral vascular disease are likely subject to misclassification. Cases of foot ulceration captured in inpatient and outpatient hospital-based specialist care are only a subset of all incident foot ulcers among individuals with type 2 diabetes. Similar issues apply to peripheral neuropathy/peripheral vascular disease, as a clinical assessment of the feet determines its presence/absence, and therefore depends on individuals attending a visit with foot examination. Still, most patients have a foot examination each year—between 79.8% and 80.5% in 2019.^43^ This limits the number of cases of peripheral neuropathy/peripheral vascular disease that could be missed and, therefore, the misclassification of actual cases as non-cases. Nevertheless, none of these issues are of concern for amputations as these procedures are always conducted in hospitals and recorded in the Patient Register. Noting that the results lend themselves to a similar interpretation across outcomes led us to believe that any misclassification of peripheral neuropathy/peripheral vascular disease or foot ulcers would not affect the overall interpretation of the study results.

To conclude, we found that low levels of self-reported physical activity were independently associated with a higher incidence of lower-extremity complications across a wide range of ages in individuals with type 2 diabetes. Importantly, we observed no discernible sex or educational differences in this association. However, integrating self-reported physical activity did not improve the predictive performance of statistical models when clinical indicators were already considered. It does not seem that self-reported physical activity would improve the identification of individuals at high risk of lower-extremity complications.

## Supplementary material

10.1136/bmjopen-2025-114759 online supplemental file 1

10.1136/bmjopen-2025-114759 online supplemental file 2

10.1136/bmjopen-2025-114759 online supplemental file 3

## Data Availability

Data may be obtained from a third party and are not publicly available.
