# Supplementary material for: Effect and prognostic value of physical activity for lower-extremity complications of type 2 diabetes across the adult lifespan: a national cohort study using Swedish registers
Source: BMJ Open. 2026 Nov 11;16(11):e114759. doi: 10.1136/bmjopen-2025-114759 (PMC13756448; doi:10.1136/bmjopen-2025-114759)
Supplement: Supplementary file 1 [file bmjopen-16-11-s001.pdf]

Table of Contents

Description of attached tables (provided as separate files) 1
Variables 2
Type 2 diabetes diagnosis date 2
Prognostic factors other than self-reported physical activity 2
Outcomes 3
Educational attainment 4
Multiple Imputation 5
Additional variables for multiple imputation 5
Bootstrapping 7
Software 8
R and R packages 8
GNU Parallel 8
Charts, diagrams and figures (Figures S1-S14) 9
References 21

Description of attached tables (provided as separate files)

Table 1.2. Summary of follow-up time and proportions of complete cases by outcome and landmark age, and number and proportions of individuals who were censored, who experienced the event, or who died event-free.

Table 1.3. Descriptive statistics for the covariates included in statistical models, comparing response sample and complete cases.

Variables

Type 2 diabetes diagnosis date

The National Diabetes Register (NDR) contains a variable indicating the diagnosis year.

The diagnosis date was determined using several data sources and operationalized as the first record among:

- The first visit recorded in the NDR,
- The first prescription of a diabetes-related drug in the Prescribed Drugs Register (Anatomical Therapeutic Chemical codes starting with "A10"),
- The first record for a diabetes-related condition in the Patient Register (see table just below).

If it was during or before the year of diagnosis of type 2 diabetes recorded in the NDR.

If the above did not suffice to assign a date of diagnosis during the recorded diagnosis year or before, a random date was assigned within the diagnosis year obtained from the NDR. 65,670 individuals had a random diagnosis date assigned, out of 348,347 (19%) – see flowchart (Figure S1).

Version of the International Classification of Diseases (ICD)	Codes used to determine type 2 diabetes diagnosis date
ICD-10	Codes starting with:<br>"E10", "E11", "E13", "E14", "O240", "O241", "O243", "O244", or "O249".
ICD-9	Codes starting with:<br>"250A", "250B", "250C", "250D", "250E", "250F", "250G", "250H", "250X", "648A".
ICD-8	Codes starting with:<br>"250,00", "250,01", "250,02", "250,03", "250,04", "250,05", "250,06", "250,07", "250,08", "250,09".
ICD-7	Codes starting with:<br>"260,09", "260,20", "260,21", "260,29", "260,30", "260,40", "260,49".

Prognostic factors other than self-reported physical activity

Quantitative variables were not categorised.

Covariate	Operationalisation	Data source
Sex	1: male<br>2: female	NDR*

Age at diagnosis of type 2 diabetes	Continuous (in years)	NDR/Patient register°
BMI	Continuous (kg/m^2)	NDR*
HbA1c	Continuous (mmol/mol)	NDR*
Smoking status	1: smoker<br>0: not a smoker	NDR*
Systolic blood pressure	Continuous (mmHg)	NDR*
Ratio of total to high-density lipoprotein cholesterol	Continuous (total cholesterol / high-density lipoprotein cholesterol - mmol/L)	NDR°
Past cardiovascular event(s)	1: ischemic heart disease or stroke<br>0: no ischemic heart disease or stroke	NDR°
Time since last healthcare visit	Continuous (in years)	NDR°
Neuropathy or peripheral vascular disease	1: yes<br>0: no	NDR°

* Used as made available in the NDR.
° Derived from several variables.

Additional information on variables from the NDR can be found on the register's website (1).

Outcomes

Peripheral neuropathy/peripheral vascular disease

This variable was obtained from the variable "fotrisk" from the NDR. The NDR variable is divided into four possible categories:

1. Healthy foot – *diabetes without complications*.
2. Neuropathy and/or angiopathy.
3. Previous diabetic ulcer(s), *foot deformities, grave callus, amputation*.
4. Ongoing serious foot disease - *ulcer, critical ischemia, infection, Charcot foot*.

Our variable included the second category above. We also assumed that if someone had a record of one of the last two categories, peripheral neuropathy/peripheral vascular disease was likely present. In addition, if someone had received hospital care for a foot ulcer or received a lower-extremity amputation not due to external causes we assumed that peripheral neuropathy/peripheral vascular disease must have been present.

Foot ulcers and amputation: ICD codes and surgical procedures codes

Event	Description	Data source	ICD-10 codes	Surgical codes
Foot ulcer	First-ever (hospitalization for a) foot ulcer	Patient register	E11.6D	-
Amputation	First-ever amputation	Patient register	-	NHQ09, NHQ11, NHQ12, NHQ13,

				NHQ14, NHQ16, NHQ17, NHQ99, NEQ19, NFQ09, NFQ19, NGQ09, NGQ19, NGQ99, NFQ99, NEQ99.
Major amputation	First-ever major amputation	Patient register	-	NEQ19, NFQ09, NFQ19, NGQ09, NGQ19, NGQ99, NFQ99, NEQ99

For foot ulcers, we used both the main and secondary diagnosis codes, as the outcome was rare. This consideration was not relevant for the other outcomes, since they were not based on diagnosis codes.

Educational attainment

We performed subgroup analysis by educational attainment, which was categorized as follows:

Low education:

**English translation**	**Original**
Preschool education	0 Förskoleutbildning
Pre-secondary education shorter than 9 years	1 Förgymnasial utbildning kortare än 9 år
Pre-secondary education 9 (10) years	2 Förgymnasial utbildning 9 (10) år

High education

**English translation**	**Original**
Secondary education	3 Gymnasial utbildning
Post-secondary education shorter than 2 years	4 Eftergymnasial utbildning kortare än två år
Post-secondary education 2 years or longer	5 Eftergymnasial utbildning två år eller längre
Postgraduate education	6 Forskarutbildning

Multiple Imputation

We imputed covariates and self-reported physical activity, but not the outcomes. In addition to the covariates mentioned above, the log of the survival time and the indicator of survival status were also included as predictors.

The number of imputations for a given landmark dataset was either:

- 20,
- the average of the proportion of missing values per variable if this was greater than 20% (2).

Based on this rule, there were always 20 imputations performed as the average of the proportion of missing values per variable was never greater than 20%.

We used predictive mean matching to impute continuous and discrete numeric variables (e.g., BMI and number of days unemployed), logistic regression to impute binary variables (e.g., smoking status), and proportional odds models to impute ordered categorical variables (e.g., physical activity). To assess the quality of the imputations, we examined density plots (continuous variables), proportion plots (categorical variables), and trace plots. These can be made available upon request.

We set the number of iterations to 20 (3).

Multiple imputations were only performed on landmark datasets where 10 or more events per variable were observed (4). Each level of a categorical variable was recoded as a dummy variable, except for the reference category, and each dummy variable counted as one variable. For example, self-reported physical activity was recoded as four dummy variables and therefore required 40 events. Landmark datasets required 130 events when peripheral neuropathy/peripheral vascular disease was the outcome, because 13 variables were included as covariates in statistical models. However, because peripheral neuropathy/peripheral vascular disease was used as a covariate in statistical models for the other outcomes, 10 additional events were required.

Additional variables for multiple imputation[1]

We did not use country of birth, but "born in Sweden". Similarly, we only used a binary variable for civil status. This is because having more categories for those variables was leading to convergence issues in multiple imputation, as some of the additional categories had very small n. Age was not used as everyone in a landmark dataset has the same age.

[1] In addition to those described under "Prognostic factors other than self-reported physical activity".

Covariate	Operationalisation	Data source
Educational attainment	See under "Educational attainment" above	Longitudinal Integrated Database for Health Insurance and Labour Market Studies (LISA)
Calendar year	Integer	NDR
Born in Sweden	- "yes"<br>- "no"	LISA
Disposable individual income (previous year)	Continuous (in 100 Swedish kronor)	LISA
Number of days unemployed (previous year)	Integer (in days)	LISA
Civil status (previous year)	- "in cohabitation"<br>- "not in cohabitation"	LISA
Foot ulcer (only for amputation)	- "yes"<br>- "no"	Patient register

Bootstrapping

We did not find that adding physical activity to clinical indicators routinely assessed in clinical practice would improve the predictive performance of statistical models. Computing model performance metrics on all imputed datasets, by communicating the greater uncertainty that comes with imputation, would not change the substantive conclusions of the article, as this would just mean greater confidence intervals for results that are already not suggesting that physical activity has added prognostic value. Because reanalysing all the imputed datasets to calculate performance measures would require substantial computing time, we did not deem these additional analyses to be justifiable given the above reasoning that the conclusions will remain the same. Based on previous analyses conducted on a randomly chosen imputed dataset for each outcome-landmark age combination, and based on the fact that multiple imputation generated 20 imputed datasets for each outcome-landmark age combination, we estimate that these additional analyses would require roughly: (i) 2 months for peripheral neuropathy/peripheral vascular disease, running the analyses in parallel on 16 cores, (ii) 3 weeks for foot ulcers, running the analyses in parallel on 12 cores, (iii) and 1 month for amputations, running the analyses in parallel on 5 cores.

Software

R and R packages

R version 4.1.1 and:

arrow: 6.0.1
haven: 2.4.3
tidyverse: 1.3.1

R version 4.2.1 and:

arrow: 12.0.0
cmprsk: 2.2.11
dplyr: 1.1.2
ggplot2: 3.4.2
lubridate: 1.9.2
magrittr: 2.0.3
mice: 3.15.0
prodlim: 2023.3.31
riskRegression: 2023.3.22
stringr: 1.5.0
tidyverse: 2.0.0

GNU Parallel

Version 20230422 ('Grand Jury') (5).

Charts, diagrams and figures (Figures S1-S14)

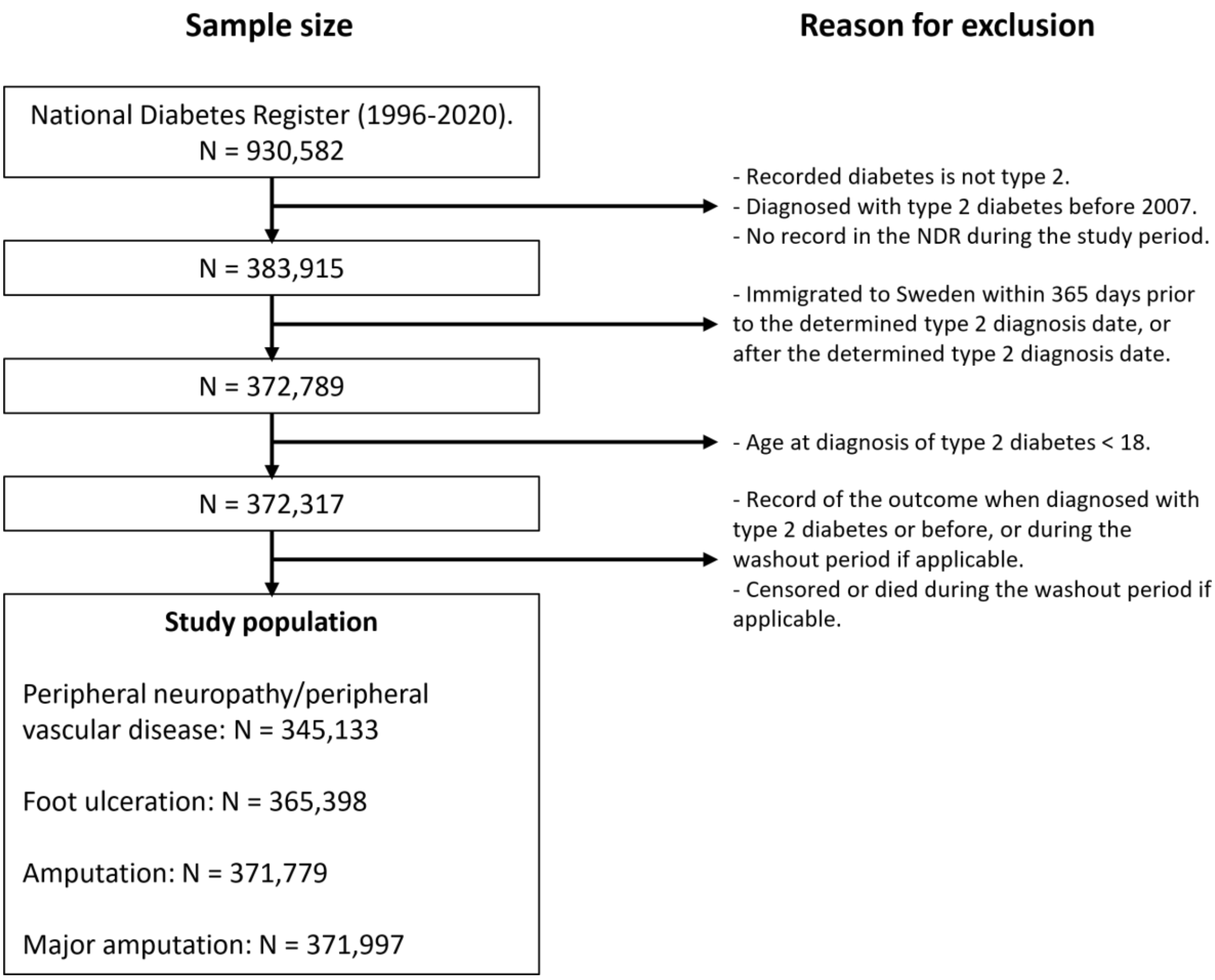

Figure S1: Flowchart describing how the study population was arrived at (before landmarking). To exclude individuals with other diabetes types, we used records of clinical diagnosis from the NDR. To determine if someone had been diagnosed with type 2 diabetes before 2007, we used the same method as described under 'Type 2 diabetes diagnosis date' in the Variables section.

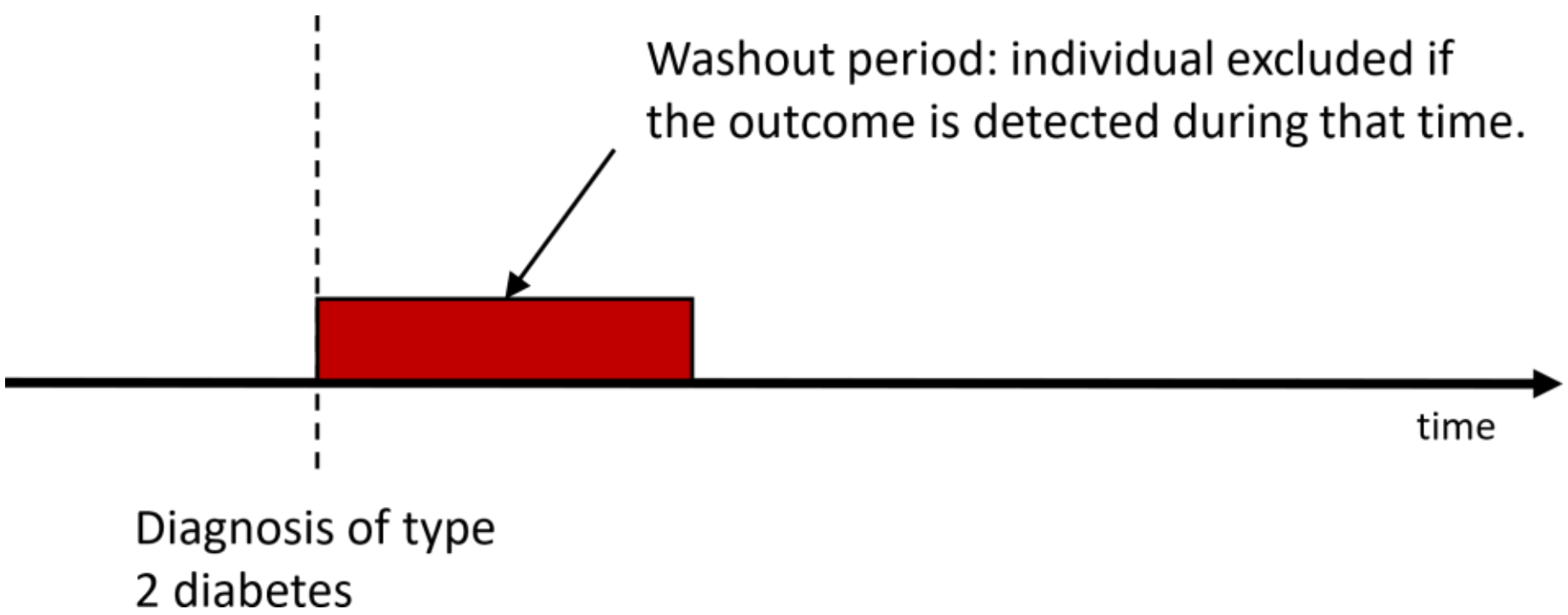

Figure S2a: Depiction of the washout period for a hypothetical individual. The period is 120 days long if the outcome is peripheral neuropathy/peripheral vascular disease, and 90 days long if the outcome is foot ulceration.

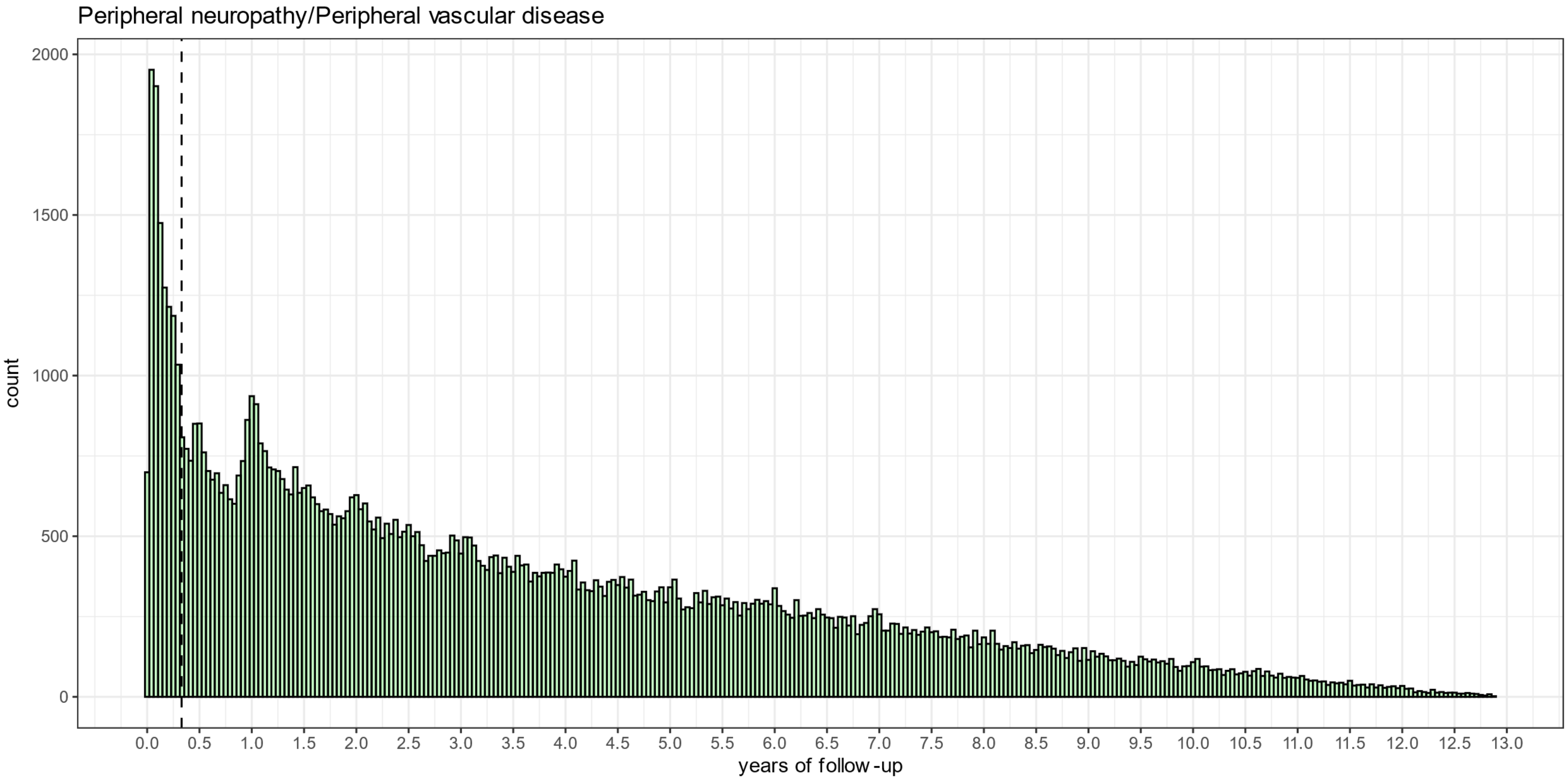

Figure S2b. Incident cases of peripheral neuropathy/peripheral vascular disease over the study period (before applying the landmarking approach). Each bar in the histogram corresponds to half a month (1 year ÷ 24). The washout period includes the time between 0 and the dashed vertical line (120 days). Individuals with a record of peripheral neuropathy/peripheral vascular disease during that time period are considered to be prevalent cases and excluded from the landmarking procedure and from subsequent analyses. n=364,568, number of events=96,282 (the numbers are larger than in Table 1.1 because they do not exclude the washout period).

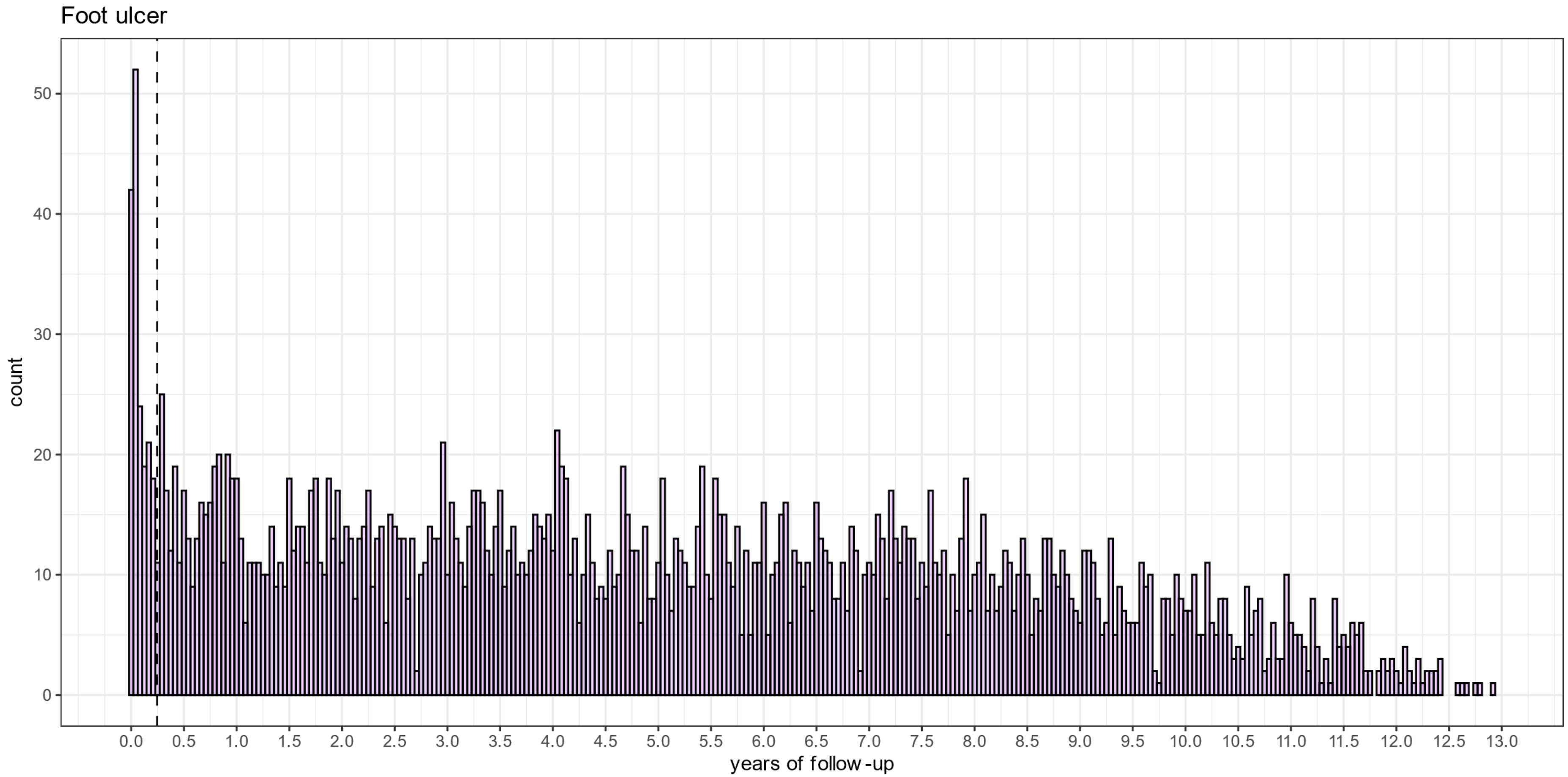

Figure S2c. Incident cases of (first-ever) foot ulcers over the study period (before applying the landmarking approach). Each bar in the histogram corresponds to half a month (1 year ÷ 24). The washout period includes the time between 0 and the dashed vertical line (90 days). Individuals with a record of foot ulcers during that time period are considered to be prevalent cases and excluded from the landmarking procedure and from subsequent analyses. n=372,157, number of events=3,140 (the numbers are larger than in Table 1.1 because they do not exclude the washout period).

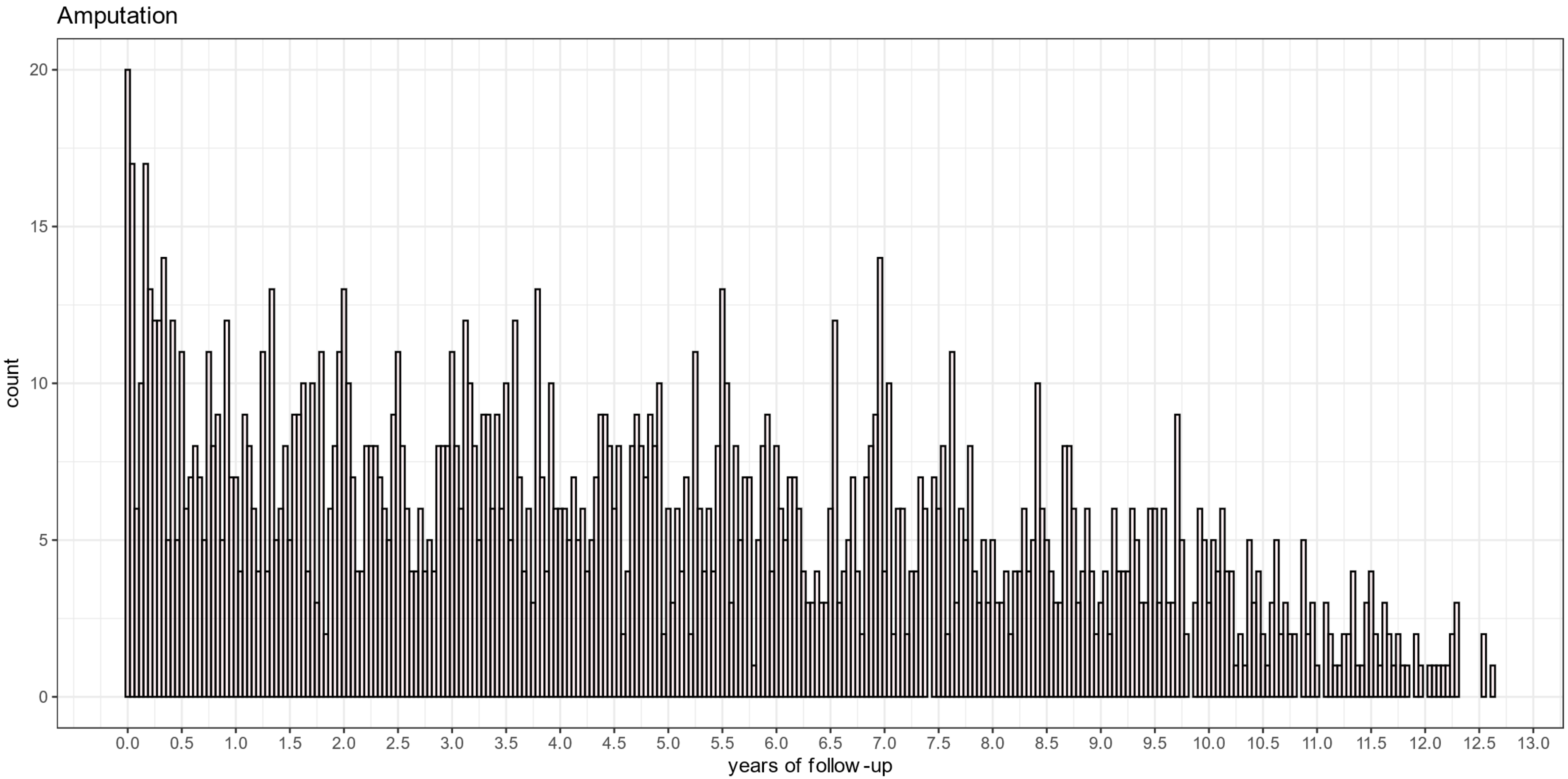

Figure S2d. Incident cases of (first-ever) lower-extremity amputations over the study period (before applying the landmarking approach). Each bar in the histogram corresponds to half a month (1 year ÷ 24). There is no washout period, because the date of the surgical procedure is recorded, and we can therefore know that all the amputations are incident amputations. Individuals with an amputation on the date of their diagnosis of type 2 diabetes do not appear in this graph as they were therefore not free of the outcome at baseline (they were not at risk of a first-ever amputation). n= 371,779, number of events= 1,669 (the numbers are the same than in Table 1.1 because there is no washout period for amputations).

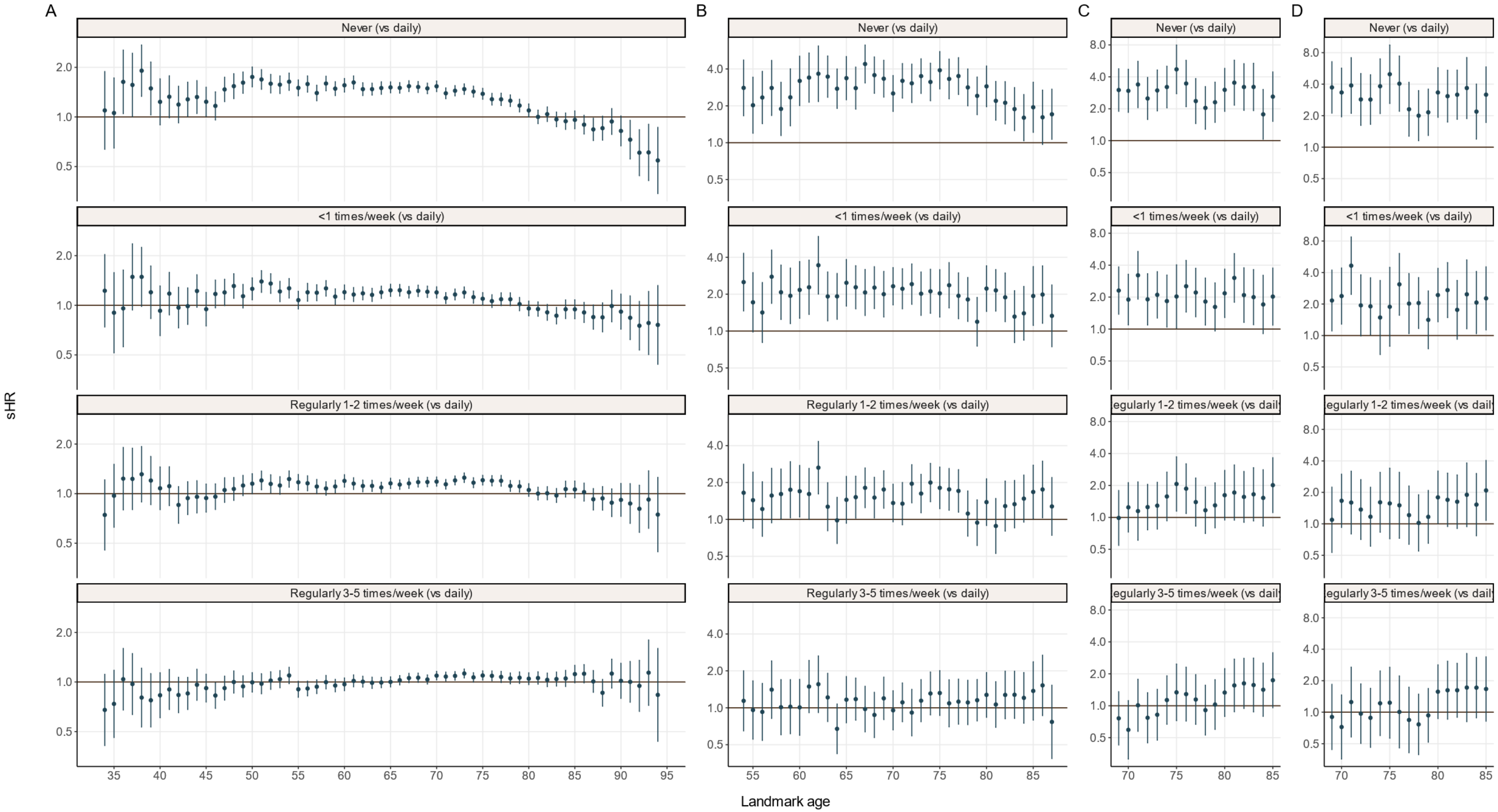

Figure S3. Association between level of self-reported physical activity and the subdistribution hazard of peripheral neuropathy/peripheral vascular disease (panel A), foot ulceration (B), amputation (C), and major amputation (D), at different landmark ages, with associated 95% confidence interval. Each physical activity category is compared with the highest category - daily walking 30 minutes or equivalent. The y-axis is on a base-2 logarithmic scale. Results from multiply imputed data, and for model 1. sHR: subdistribution hazard ratio.

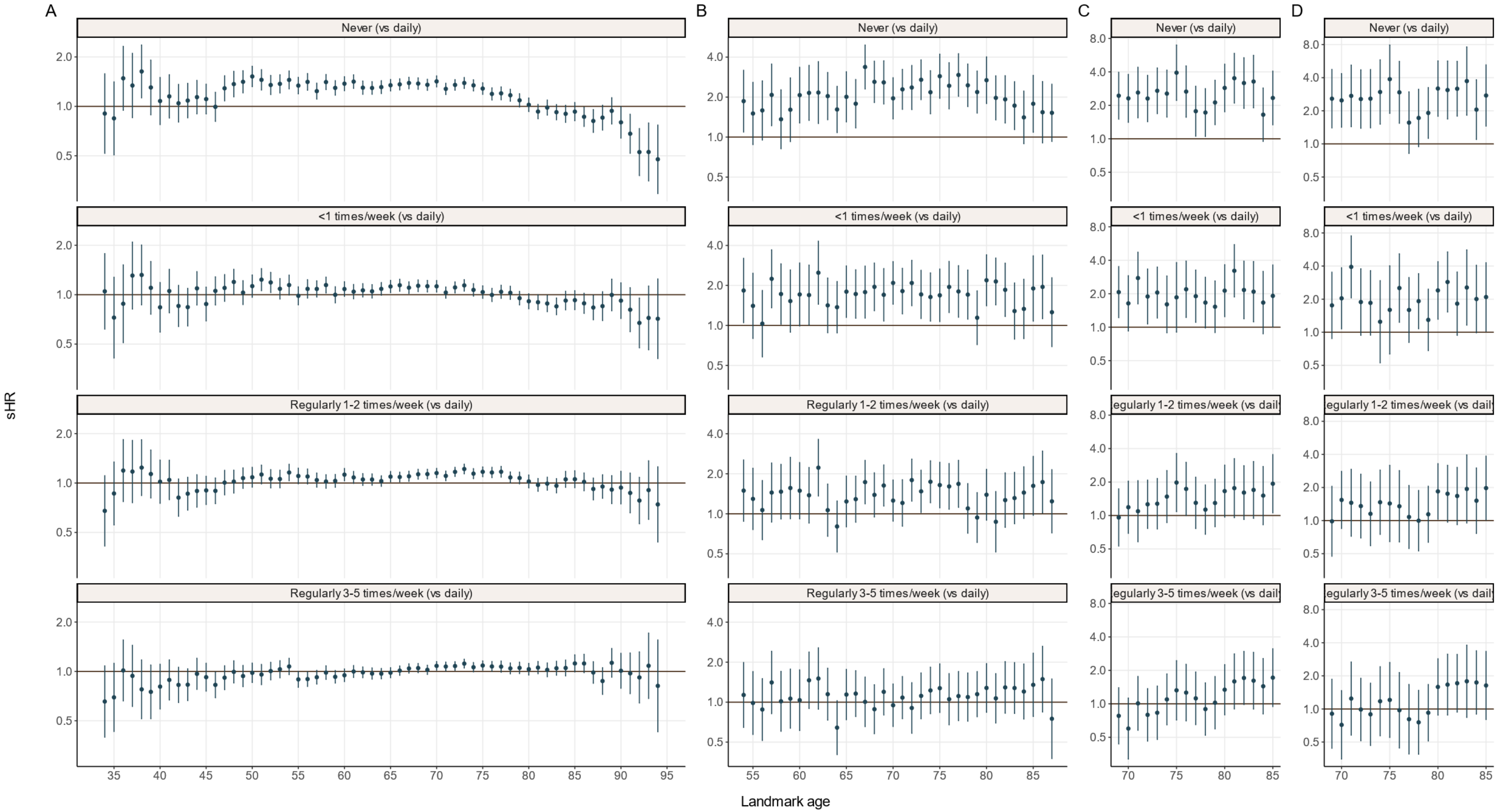

Figure S4. Association between level of self-reported physical activity and the subdistribution hazard of peripheral neuropathy/peripheral vascular disease (panel A), foot ulceration (B), amputation (C), and major amputation (D), at different landmark ages, with associated 95% confidence interval. Each physical activity category is compared with the highest category - daily walking 30 minutes or equivalent. The y-axis is on a base-2 logarithmic scale. Results from multiply imputed data, and for model 3 (the first three panels are identical to Figure 2 with the main text). sHR: subdistribution hazard ratio.

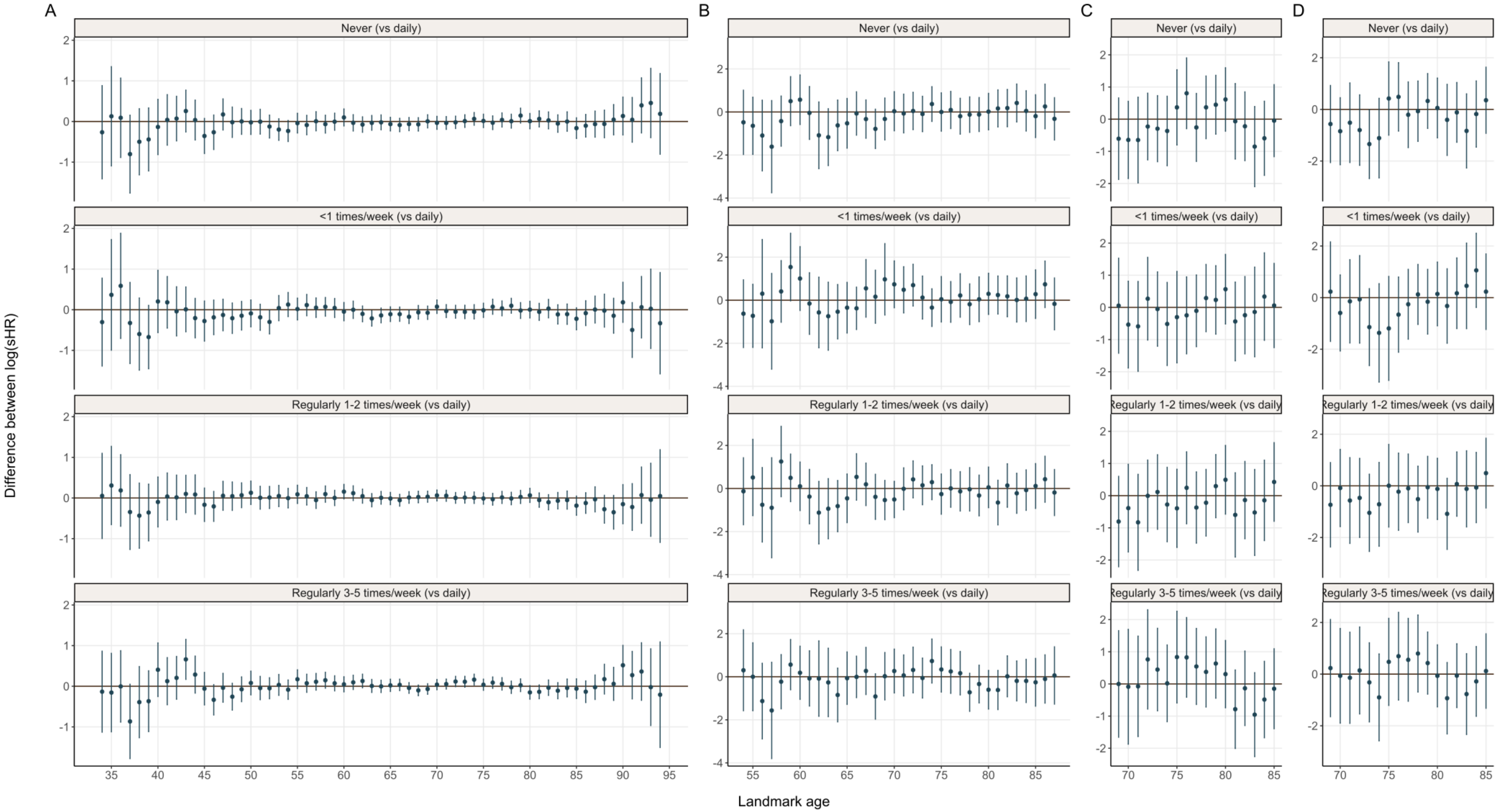

Figure S5. Difference in estimates for peripheral neuropathy/peripheral vascular disease (panel A), foot ulceration (B), amputation (C), and major amputation (D) between males and females at different landmark ages, with associated 95% confidence interval. Each point is a difference in estimate, so 0 indicates no difference between groups. Results from multiply imputed data, and for model 3. sHR: subdistribution hazard ratio.

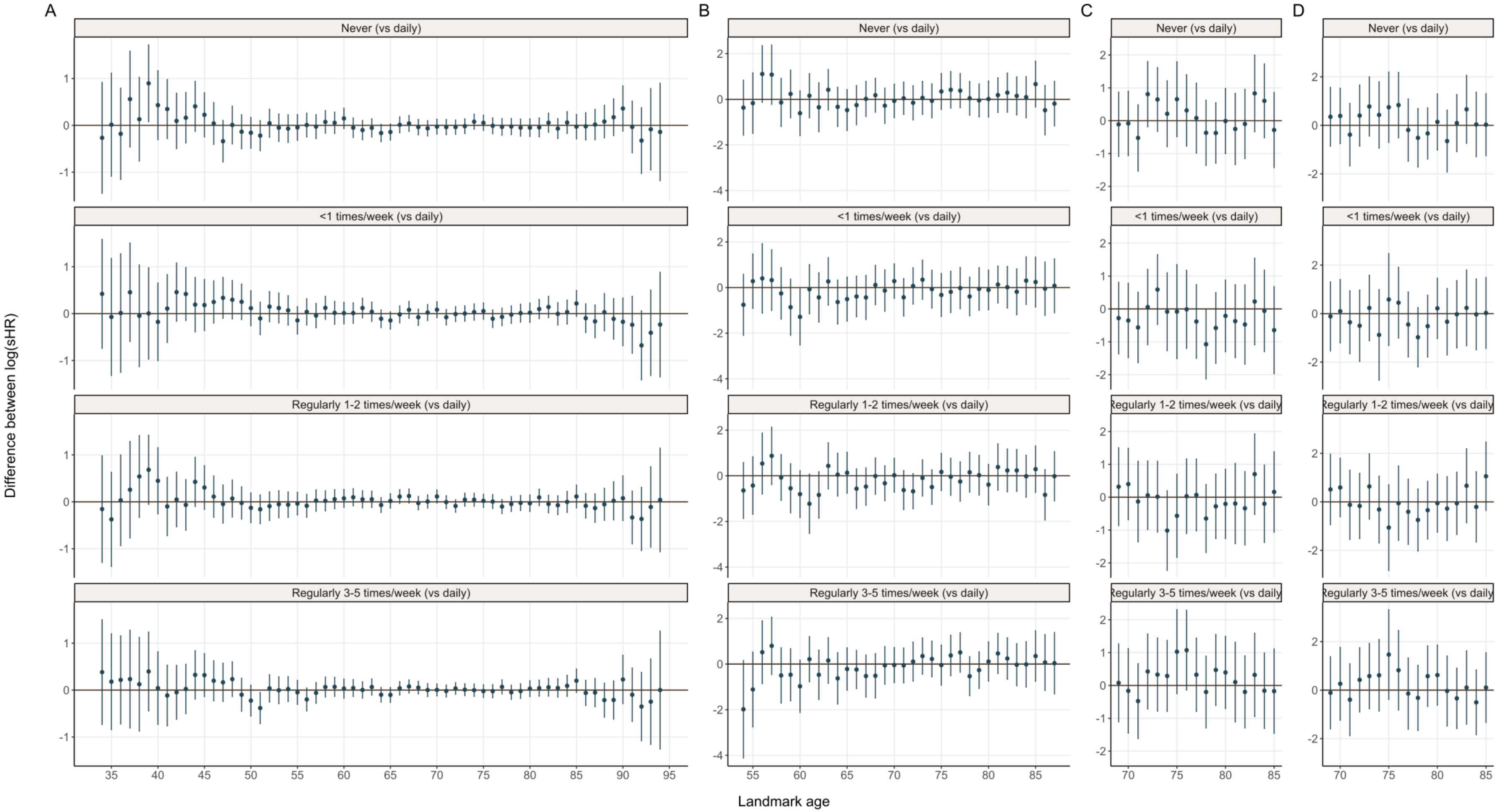

Figure S6. Difference in estimates for peripheral neuropathy/peripheral vascular disease (panel A), foot ulceration (B), amputation (C), and major amputation (D) between low and high education at different landmark ages, with associated 95% confidence interval. Each point is a difference in estimate, so 0 indicates no difference between groups. Results from multiply imputed data, and for model 3. sHR: subdistribution hazard ratio.

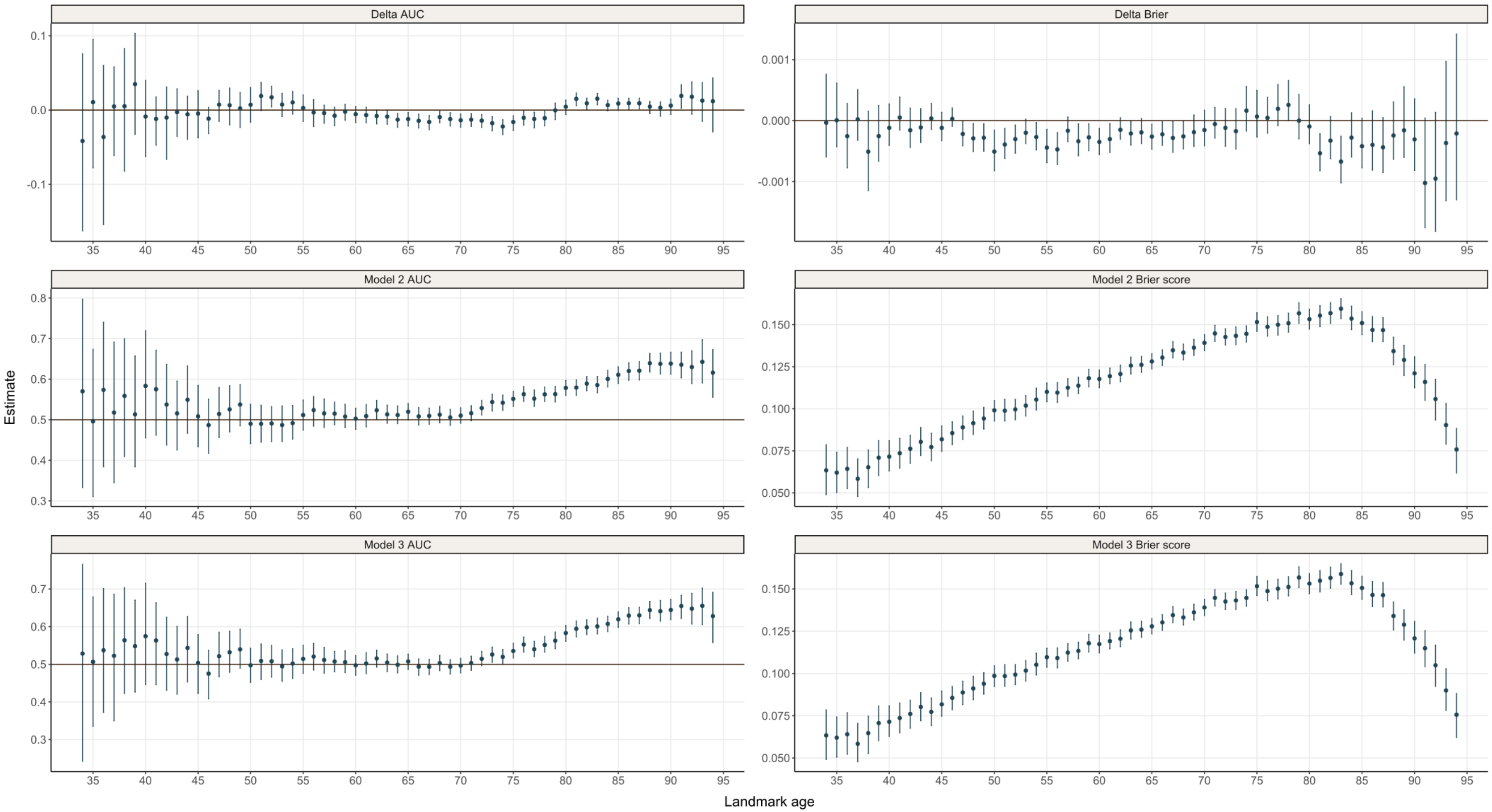

Figure S7. Comparison of time-dependent area under the receiver operating characteristic curve, and time-dependent Brier score, in models without (model 2) and with physical activity (model 3) for different landmark ages, at five years of follow-up, with 95% confidence interval. If delta AUC or delta Brier is 0, it indicates that there is no change in discrimination or in performance when adding physical activity into a model that already includes the other prognostic factors. Outcome: peripheral neuropathy/peripheral vascular disease.

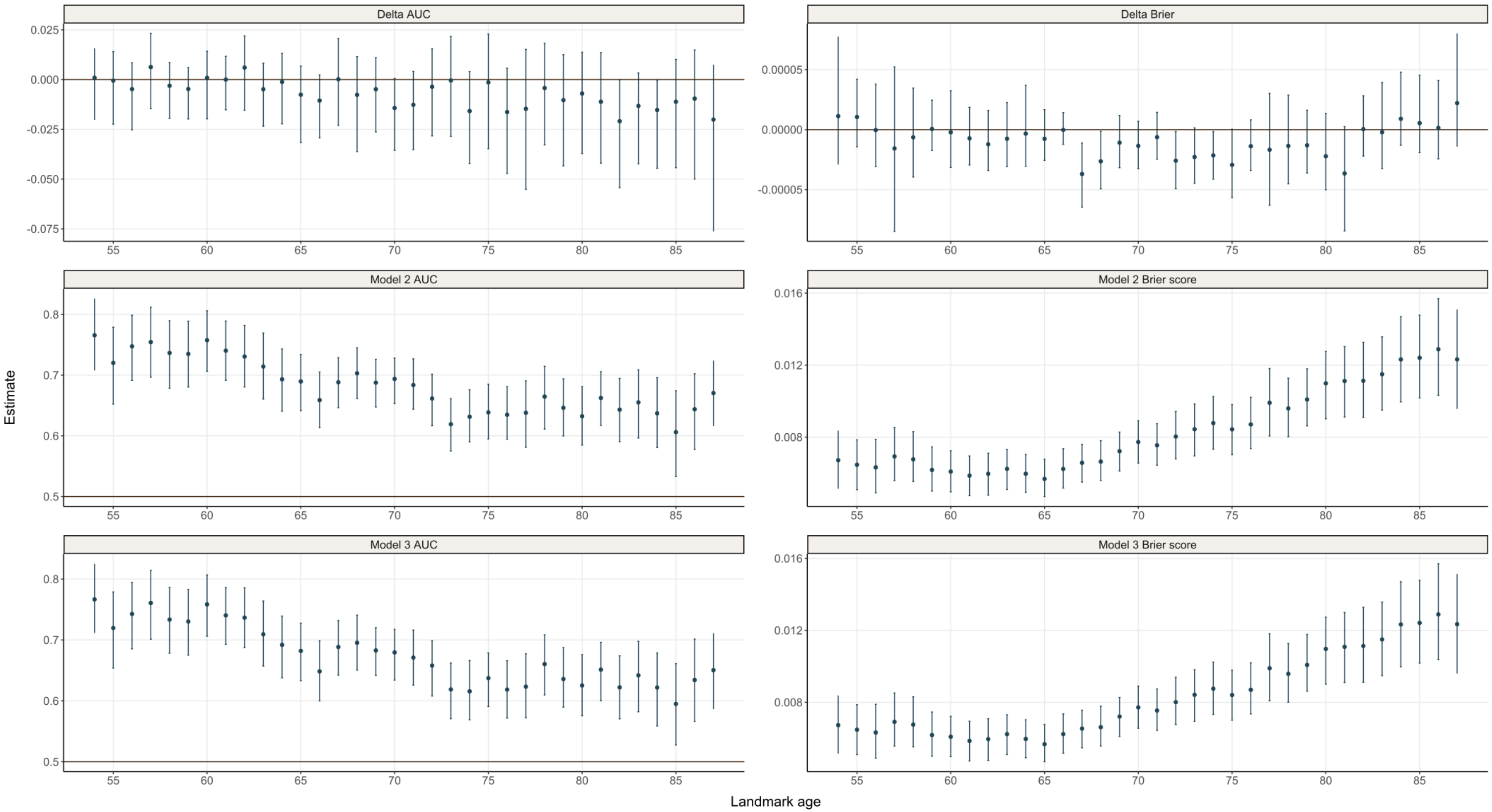

Figure S8. Comparison of time-dependent area under the receiver operating characteristic curve, and time-dependent Brier score, in models without (model 2) and with physical activity (model 3) for different landmark ages, at five years of follow-up, with 95% confidence interval. If delta AUC or delta Brier is 0, it indicates that there is no change in discrimination or in performance when adding physical activity into a model that already includes the other prognostic factors. Outcome: foot ulceration.

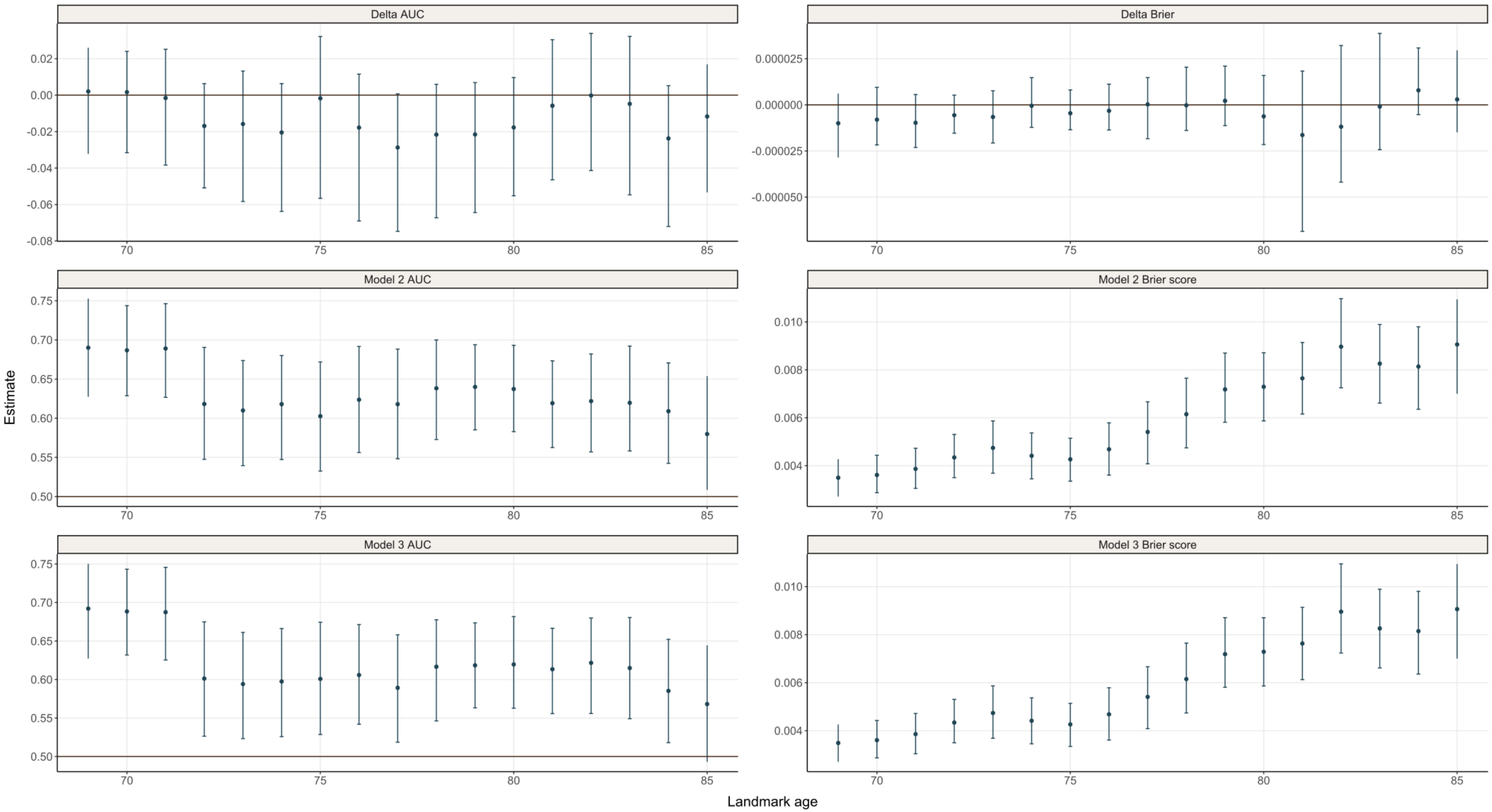

Figure S9. Comparison of time-dependent area under the receiver operating characteristic curve, and time-dependent Brier score, in models without (model 2) and with physical activity (model 3) for different landmark ages, at five years of follow-up, with 95% confidence interval. If delta AUC or delta Brier is 0, it indicates that there is no change in discrimination or in performance when adding physical activity into a model that already includes the other prognostic factors. Outcome: amputation.

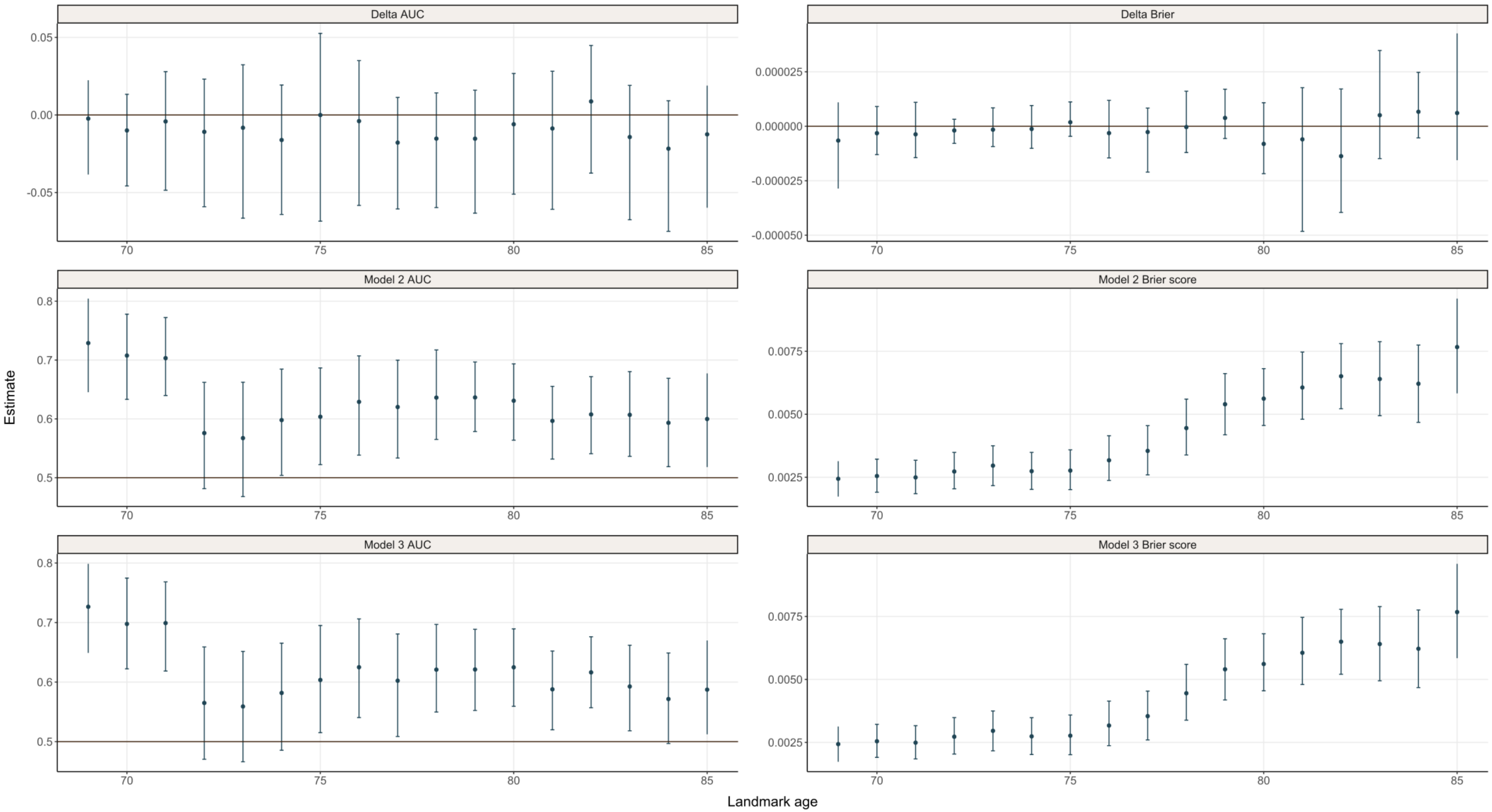

Figure S10. Comparison of time-dependent area under the receiver operating characteristic curve, and time-dependent Brier score, in models without (model 2) and with physical activity (model 3) for different landmark ages, at five years of follow-up, with 95% confidence interval. If delta AUC or delta Brier is 0, it indicates that there is no change in discrimination or in performance when adding physical activity into a model that already includes the other prognostic factors. Outcome: major amputation.

References

1. National Diabetes Register. Variable list [Available from: https://ndr.registercentrum.se/forskning/variabellista-1/p/ByJX32euh.

2. van Buuren S. 2.8 How many imputations? In: van Buuren S, editor. Flexible Imputation of Missing Data, Second Edition. 2nd ed: Chapman and Hall/CRC; 2018.

3. van Buuren S. 4.5.6 Number of iterations. In: van Buuren S, editor. Flexible Imputation of Missing Data, Second Edition. 2nd ed: Chapman and Hall/CRC; 2018.

4. Austin PC, Allignol A, Fine JP. The number of primary events per variable affects estimation of the subdistribution hazard competing risks model. Journal of Clinical Epidemiology. 2017;83:75-84.

5. Tange O. GNU Parallel 20230422 ('Grand Jury') released. Zenodo; 2023.
