# Supplementary material for: Effect and prognostic value of physical activity for lower-extremity complications of type 2 diabetes across the adult lifespan: a national cohort study using Swedish registers
Source: BMJ Open. 2026 Nov 11;16(11):e114759. doi: 10.1136/bmjopen-2025-114759 (PMC13756448; doi:10.1136/bmjopen-2025-114759)
Supplement: Supplementary file 2 [file bmjopen-16-11-s002.pdf]

Table 1.2. Summary of follow-up time and proportions of complete cases by outcome and landmark age, and number and proportions of individuals who were censored, who experienced the event, or who died event-free.

Outcome	Landmark age	n	Total follow up time	Censored (n)	Censored (%)	Experienced the event (n)	Experienced the event (%)	Died event-free (n)	Died event-free (%)	Complete cases (%)
Amputation	19	62	210	62	100	0	0	0	0	27.4
Amputation	20	142	516	142	100	0	0	0	0	35.9
Amputation	21	239	909	239	100	0	0	0	0	45.6
Amputation	22	363	1349	359	98.9	0	0	4	1.1	51
Amputation	23	518	1896	512	98.8	0	0	6	1.2	53.1
Amputation	24	717	2493	709	98.9	0	0	8	1.1	53.7
Amputation	25	892	2979	884	99.1	0	0	8	0.9	54.4
Amputation	26	1077	3598	1072	99.5	0	0	5	0.5	56
Amputation	27	1282	4173	1273	99.3	0	0	9	0.7	56.2
Amputation	28	1502	4817	1490	99.2	0	0	12	0.8	56.3
Amputation	29	1719	5452	1703	99.1	0	0	16	0.9	57.5
Amputation	30	1954	6108	1940	99.3	0	0	14	0.7	57.9
Amputation	31	2224	7044	2208	99.3	0	0	16	0.7	58.1
Amputation	32	2570	8273	2548	99.1	1	0	21	0.8	59
Amputation	33	2977	9629	2953	99.2	2	0.1	22	0.7	58.6
Amputation	34	3407	11063	3382	99.3	3	0.1	22	0.6	59.5
Amputation	35	3863	12642	3827	99.1	2	0.1	34	0.9	60.2
Amputation	36	4360	14464	4322	99.1	1	0	37	0.8	61.6
Amputation	37	5003	16629	4957	99.1	2	0	44	0.9	62
Amputation	38	5740	19247	5681	99	5	0.1	54	0.9	62.8

Amputation	39	6696	22417	6622	98.9	6	0.1	68	1	63.6
Amputation	40	7603	25837	7522	98.9	6	0.1	75	1	64.1
Amputation	41	8838	29949	8737	98.9	10	0.1	91	1	64.5
Amputation	42	10060	34152	9954	98.9	9	0.1	97	1	65.1
Amputation	43	11348	38481	11222	98.9	10	0.1	116	1	66
Amputation	44	12927	44105	12764	98.7	9	0.1	154	1.2	65.8
Amputation	45	14710	50347	14500	98.6	14	0.1	196	1.3	66
Amputation	46	16585	57209	16305	98.3	21	0.1	259	1.6	66.6
Amputation	47	18576	63755	18258	98.3	23	0.1	295	1.6	67.4
Amputation	48	20465	70070	20089	98.2	25	0.1	351	1.7	68
Amputation	49	22752	77364	22343	98.2	34	0.1	375	1.6	68.5
Amputation	50	25322	85227	24865	98.2	37	0.1	420	1.7	68.6
Amputation	51	27877	92004	27352	98.1	38	0.1	487	1.7	69.6
Amputation	52	30067	98062	29464	98	40	0.1	563	1.9	70
Amputation	53	32067	103660	31390	97.9	33	0.1	644	2	70.1
Amputation	54	34011	109381	33203	97.6	42	0.1	766	2.3	70.5
Amputation	55	35973	115898	35095	97.6	40	0.1	838	2.3	70.4
Amputation	56	37750	122740	36794	97.5	47	0.1	909	2.4	70.7
Amputation	57	39614	129668	38534	97.3	55	0.1	1025	2.6	71.1
Amputation	58	41630	136171	40342	96.9	66	0.2	1222	2.9	71.6
Amputation	59	44059	143830	42628	96.8	63	0.1	1368	3.1	71.8
Amputation	60	46664	152293	45023	96.5	71	0.2	1570	3.4	71.5
Amputation	61	49893	162978	48015	96.2	78	0.2	1800	3.6	71.7
Amputation	62	52325	170928	50228	96	79	0.2	2018	3.9	71.9

Amputation	63	54775	180010	52401	95.7	88	0.2	2286	4.2	71.6
Amputation	64	57025	188353	54363	95.3	94	0.2	2568	4.5	71.9
Amputation	65	59472	196557	56481	95	109	0.2	2882	4.8	72
Amputation	66	62040	204038	58719	94.6	109	0.2	3212	5.2	72.3
Amputation	67	63999	208798	60295	94.2	125	0.2	3579	5.6	72.4
Amputation	68	65337	211402	61311	93.8	129	0.2	3897	6	73
Amputation	69	66242	209388	61824	93.3	146	0.2	4272	6.4	73.6
Amputation	70	66413	205516	61762	93	156	0.2	4495	6.8	73.6
Amputation	71	65748	198549	60841	92.5	161	0.2	4746	7.2	73.9
Amputation	72	63847	188307	58695	91.9	169	0.3	4983	7.8	74.1
Amputation	73	61627	177892	56313	91.4	172	0.3	5142	8.3	73.9
Amputation	74	58451	165831	53014	90.7	160	0.3	5277	9	73.7
Amputation	75	54748	154956	49204	89.9	146	0.3	5398	9.9	73.5
Amputation	76	50918	145049	45246	88.9	150	0.3	5522	10.8	73
Amputation	77	47266	135566	41409	87.6	149	0.3	5708	12.1	72.5
Amputation	78	44012	126545	37854	86	165	0.4	5993	13.6	71.9
Amputation	79	41267	117941	34956	84.7	185	0.4	6126	14.8	70.8
Amputation	80	38499	108659	31949	83	190	0.5	6360	16.5	70.1
Amputation	81	35572	99245	28790	80.9	188	0.5	6594	18.5	68.6
Amputation	82	32526	89615	25634	78.8	194	0.6	6698	20.6	67.7
Amputation	83	29591	80244	22731	76.8	166	0.6	6694	22.6	66.6
Amputation	84	26648	71239	19794	74.3	152	0.6	6702	25.2	65.2
Amputation	85	23859	62323	17074	71.6	152	0.6	6633	27.8	63.7
Amputation	86	21081	53746	14544	69	135	0.6	6402	30.4	62.2

Amputation	87	18261	44991	11986	65.6	119	0.7	6156	33.7	60.2
Amputation	88	15718	36919	9842	62.6	121	0.8	5755	36.6	58.2
Amputation	89	13003	29492	7790	59.9	104	0.8	5109	39.3	56.2
Amputation	90	10465	23142	5940	56.8	87	0.8	4438	42.4	54.1
Amputation	91	8303	17477	4476	53.9	72	0.9	3755	45.2	52.3
Amputation	92	6352	12840	3257	51.3	58	0.9	3037	47.8	49.2
Amputation	93	4729	9106	2331	49.3	31	0.7	2367	50.1	46.9
Amputation	94	3446	6323	1587	46.1	25	0.7	1834	53.2	44
Amputation	95	2444	4202	1073	43.9	18	0.7	1353	55.4	40.7
Amputation	96	1614	2652	680	42.1	10	0.6	924	57.2	37.9
Amputation	97	1047	1624	407	38.9	5	0.5	635	60.6	35.9
Amputation	98	649	907	252	38.8	3	0.5	394	60.7	35
Amputation	99	375	504	140	37.3	4	1.1	231	61.6	32
Amputation	100	207	256	62	30	3	1.4	142	68.6	26.6
Major Amputation	19	62	210	62	100	0	0	0	0	27.4
Major Amputation	20	144	526	144	100	0	0	0	0	35.4
Major Amputation	21	241	919	241	100	0	0	0	0	45.6
Major Amputation	22	365	1359	361	98.9	0	0	4	1.1	51
Major Amputation	23	520	1906	514	98.8	0	0	6	1.2	53.1
Major Amputation	24	720	2507	712	98.9	0	0	8	1.1	53.6
Major Amputation	25	895	2992	887	99.1	0	0	8	0.9	54.3
Major Amputation	26	1080	3610	1075	99.5	0	0	5	0.5	55.9
Major Amputation	27	1286	4189	1277	99.3	0	0	9	0.7	56.1
Major Amputation	28	1506	4832	1494	99.2	0	0	12	0.8	56.2

Major Amputation	29	1722	5467	1706	99.1	0	0	16	0.9	57.4
Major Amputation	30	1957	6123	1943	99.3	0	0	14	0.7	57.8
Major Amputation	31	2227	7059	2211	99.3	0	0	16	0.7	58
Major Amputation	32	2574	8296	2553	99.2	0	0	21	0.8	58.9
Major Amputation	33	2981	9653	2959	99.3	0	0	22	0.7	58.5
Major Amputation	34	3413	11103	3391	99.4	0	0	22	0.6	59.4
Major Amputation	35	3871	12682	3837	99.1	0	0	34	0.9	60.2
Major Amputation	36	4370	14511	4333	99.2	0	0	37	0.8	61.6
Major Amputation	37	5014	16680	4970	99.1	0	0	44	0.9	61.9
Major Amputation	38	5753	19303	5697	99	2	0	54	0.9	62.7
Major Amputation	39	6708	22472	6637	98.9	3	0	68	1	63.6
Major Amputation	40	7616	25893	7537	99	4	0.1	75	1	64
Major Amputation	41	8855	30022	8758	98.9	6	0.1	91	1	64.4
Major Amputation	42	10076	34221	9974	99	5	0	97	1	65
Major Amputation	43	11363	38543	11240	98.9	6	0.1	117	1	65.9
Major Amputation	44	12944	44172	12784	98.8	5	0	155	1.2	65.7
Major Amputation	45	14728	50413	14524	98.6	7	0	197	1.3	66
Major Amputation	46	16609	57316	16339	98.4	10	0.1	260	1.6	66.5
Major Amputation	47	18601	63875	18294	98.3	12	0.1	295	1.6	67.3
Major Amputation	48	20493	70210	20132	98.2	10	0	351	1.7	67.9
Major Amputation	49	22783	77521	22390	98.3	16	0.1	377	1.7	68.4
Major Amputation	50	25368	85421	24921	98.2	24	0.1	423	1.7	68.6
Major Amputation	51	27931	92230	27418	98.2	21	0.1	492	1.8	69.5
Major Amputation	52	30119	98287	29529	98	22	0.1	568	1.9	69.9

Major Amputation	53	32129	103926	31462	97.9	18	0.1	649	2	70.1
Major Amputation	54	34075	109649	33285	97.7	20	0.1	770	2.3	70.5
Major Amputation	55	36043	116193	35187	97.6	15	0	841	2.3	70.4
Major Amputation	56	37827	123061	36891	97.5	21	0.1	915	2.4	70.7
Major Amputation	57	39704	130025	38649	97.3	25	0.1	1030	2.6	71
Major Amputation	58	41721	136556	40465	97	29	0.1	1227	2.9	71.6
Major Amputation	59	44156	144226	42750	96.8	27	0.1	1379	3.1	71.7
Major Amputation	60	46766	152697	45147	96.5	30	0.1	1589	3.4	71.4
Major Amputation	61	49992	163372	48135	96.3	36	0.1	1821	3.6	71.6
Major Amputation	62	52426	171345	50346	96	41	0.1	2039	3.9	71.8
Major Amputation	63	54877	180433	52516	95.7	53	0.1	2308	4.2	71.6
Major Amputation	64	57127	188775	54483	95.4	57	0.1	2587	4.5	71.8
Major Amputation	65	59584	197020	56617	95	65	0.1	2902	4.9	71.9
Major Amputation	66	62161	204532	58856	94.7	68	0.1	3237	5.2	72.2
Major Amputation	67	64123	209297	60433	94.2	83	0.1	3607	5.6	72.4
Major Amputation	68	65459	211896	61454	93.9	82	0.1	3923	6	72.9
Major Amputation	69	66373	209869	61971	93.4	99	0.1	4303	6.5	73.5
Major Amputation	70	66548	205960	61911	93	114	0.2	4523	6.8	73.5
Major Amputation	71	65869	198957	60977	92.6	108	0.2	4784	7.3	73.9
Major Amputation	72	63958	188692	58832	92	108	0.2	5018	7.8	74.1
Major Amputation	73	61744	178297	56455	91.4	111	0.2	5178	8.4	73.8
Major Amputation	74	58560	166223	53143	90.7	99	0.2	5318	9.1	73.7
Major Amputation	75	54856	155337	49322	89.9	92	0.2	5442	9.9	73.5
Major Amputation	76	51017	145424	45361	88.9	100	0.2	5556	10.9	72.9

Major Amputation	77	47367	135930	41526	87.7	100	0.2	5741	12.1	72.5
Major Amputation	78	44112	126896	37962	86.1	119	0.3	6031	13.7	71.8
Major Amputation	79	41355	118287	35058	84.8	135	0.3	6162	14.9	70.8
Major Amputation	80	38592	108980	32038	83	147	0.4	6407	16.6	70
Major Amputation	81	35657	99527	28870	81	148	0.4	6639	18.6	68.6
Major Amputation	82	32609	89890	25712	78.8	151	0.5	6746	20.7	67.6
Major Amputation	83	29668	80493	22800	76.9	127	0.4	6741	22.7	66.6
Major Amputation	84	26719	71461	19850	74.3	118	0.4	6751	25.3	65.1
Major Amputation	85	23922	62536	17123	71.6	126	0.5	6673	27.9	63.6
Major Amputation	86	21143	53927	14587	69	109	0.5	6447	30.5	62.1
Major Amputation	87	18321	45140	12026	65.6	102	0.6	6193	33.8	60.2
Major Amputation	88	15764	37038	9875	62.6	96	0.6	5793	36.7	58.1
Major Amputation	89	13035	29580	7810	59.9	85	0.7	5140	39.4	56.1
Major Amputation	90	10492	23215	5958	56.8	65	0.6	4469	42.6	54
Major Amputation	91	8327	17535	4491	53.9	57	0.7	3779	45.4	52.2
Major Amputation	92	6373	12887	3269	51.3	47	0.7	3057	48	49.2
Major Amputation	93	4745	9138	2339	49.3	25	0.5	2381	50.2	46.8
Major Amputation	94	3455	6344	1592	46.1	18	0.5	1845	53.4	44
Major Amputation	95	2450	4214	1076	43.9	16	0.7	1358	55.4	40.7
Major Amputation	96	1617	2660	682	42.2	10	0.6	925	57.2	37.8
Major Amputation	97	1049	1629	409	39	5	0.5	635	60.5	35.8
Major Amputation	98	651	910	254	39	3	0.5	394	60.5	34.9
Major Amputation	99	376	505	142	37.8	3	0.8	231	61.4	31.9
Major Amputation	100	208	257	64	30.8	2	1	142	68.3	26.4

Neuropathy	19	62	210	61	98.4	1	1.6	0	0	27.4
Neuropathy	20	140	504	136	97.1	4	2.9	0	0	35
Neuropathy	21	235	885	228	97	7	3	0	0	45.1
Neuropathy	22	356	1303	343	96.3	9	2.5	4	1.1	50.8
Neuropathy	23	504	1828	484	96	14	2.8	6	1.2	52.6
Neuropathy	24	700	2401	669	95.6	23	3.3	8	1.1	53.1
Neuropathy	25	872	2841	830	95.2	35	4	7	0.8	53.6
Neuropathy	26	1047	3416	1004	95.9	39	3.7	4	0.4	55.3
Neuropathy	27	1237	3928	1178	95.2	51	4.1	8	0.6	55.5
Neuropathy	28	1450	4543	1383	95.4	56	3.9	11	0.8	55.5
Neuropathy	29	1647	5132	1577	95.7	56	3.4	14	0.9	56.2
Neuropathy	30	1876	5741	1790	95.4	73	3.9	13	0.7	56.7
Neuropathy	31	2136	6603	2025	94.8	96	4.5	15	0.7	57.2
Neuropathy	32	2459	7793	2344	95.3	96	3.9	19	0.8	58.2
Neuropathy	33	2853	9040	2708	94.9	127	4.5	18	0.6	58
Neuropathy	34	3242	10276	3063	94.5	161	5	18	0.6	58.8
Neuropathy	35	3674	11706	3465	94.3	182	5	27	0.7	59.4
Neuropathy	36	4139	13376	3897	94.2	214	5.2	28	0.7	60.6
Neuropathy	37	4727	15351	4469	94.5	224	4.7	34	0.7	61.1
Neuropathy	38	5418	17715	5086	93.9	290	5.4	42	0.8	61.9
Neuropathy	39	6301	20550	5878	93.3	367	5.8	56	0.9	62.5
Neuropathy	40	7151	23638	6659	93.1	431	6	61	0.9	62.9
Neuropathy	41	8289	27328	7690	92.8	522	6.3	77	0.9	63.2
Neuropathy	42	9437	31016	8725	92.5	630	6.7	82	0.9	64

Neuropathy	43	10588	34598	9736	92	755	7.1	97	0.9	64.6
Neuropathy	44	12006	39600	11060	92.1	826	6.9	120	1	64.4
Neuropathy	45	13603	45032	12458	91.6	998	7.3	147	1.1	64.6
Neuropathy	46	15293	50883	13874	90.7	1217	8	202	1.3	65.1
Neuropathy	47	17088	56288	15432	90.3	1424	8.3	232	1.4	65.9
Neuropathy	48	18715	61433	16853	90.1	1594	8.5	268	1.4	66.6
Neuropathy	49	20719	67276	18597	89.8	1830	8.8	292	1.4	67
Neuropathy	50	22975	73726	20531	89.4	2116	9.2	328	1.4	67.1
Neuropathy	51	25087	79125	22466	89.6	2255	9	366	1.5	67.9
Neuropathy	52	26898	83834	23953	89.1	2526	9.4	419	1.6	68.2
Neuropathy	53	28472	88142	25268	88.7	2717	9.5	487	1.7	68.2
Neuropathy	54	30045	92333	26454	88	3018	10	573	1.9	68.6
Neuropathy	55	31627	97261	27700	87.6	3303	10.4	624	2	68.4
Neuropathy	56	33120	102441	28831	87.1	3600	10.9	689	2.1	68.8
Neuropathy	57	34559	107443	29897	86.5	3893	11.3	769	2.2	69.1
Neuropathy	58	36235	112087	31061	85.7	4252	11.7	922	2.5	69.5
Neuropathy	59	38103	117456	32484	85.3	4603	12.1	1016	2.7	69.6
Neuropathy	60	40109	123422	34024	84.8	4922	12.3	1163	2.9	69.3
Neuropathy	61	42779	131475	36003	84.2	5457	12.8	1319	3.1	69.4
Neuropathy	62	44517	136728	37247	83.7	5811	13.1	1459	3.3	69.4
Neuropathy	63	46316	142824	38363	82.8	6334	13.7	1619	3.5	69.1
Neuropathy	64	47982	147868	39341	82	6861	14.3	1780	3.7	69.1
Neuropathy	65	49573	152362	40200	81.1	7402	14.9	1971	4	69.1
Neuropathy	66	51128	156103	41105	80.4	7880	15.4	2143	4.2	69.2

Neuropathy	67	52151	157741	41650	79.9	8136	15.6	2365	4.5	69.3
Neuropathy	68	52652	157571	41678	79.2	8431	16	2543	4.8	69.7
Neuropathy	69	52693	153466	41350	78.5	8588	16.3	2755	5.2	70.2
Neuropathy	70	52099	148464	40569	77.9	8698	16.7	2832	5.4	70
Neuropathy	71	50943	141056	39209	77	8740	17.2	2994	5.9	70.1
Neuropathy	72	48682	131645	37057	76.1	8528	17.5	3097	6.4	70.1
Neuropathy	73	46325	122778	34958	75.5	8200	17.7	3167	6.8	69.6
Neuropathy	74	43160	112814	32187	74.6	7742	17.9	3231	7.5	69.2
Neuropathy	75	39964	104011	29277	73.3	7400	18.5	3287	8.2	68.7
Neuropathy	76	36820	96134	26498	72	6995	19	3327	9	67.8
Neuropathy	77	33886	88686	23914	70.6	6536	19.3	3436	10.1	67
Neuropathy	78	31211	81699	21527	69	6087	19.5	3597	11.5	66.3
Neuropathy	79	28926	75284	19554	67.6	5709	19.7	3663	12.7	64.9
Neuropathy	80	26645	68413	17635	66.2	5241	19.7	3769	14.1	63.7
Neuropathy	81	24330	61607	15573	64	4839	19.9	3918	16.1	61.7
Neuropathy	82	21946	54817	13578	61.9	4367	19.9	4001	18.2	60.5
Neuropathy	83	19642	48417	11837	60.3	3819	19.4	3986	20.3	59
Neuropathy	84	17465	42522	10173	58.2	3273	18.7	4019	23	56.8
Neuropathy	85	15434	36744	8623	55.9	2792	18.1	4019	26	54.9
Neuropathy	86	13486	31654	7310	54.2	2318	17.2	3858	28.6	52.7
Neuropathy	87	11580	26571	6012	51.9	1872	16.2	3696	31.9	50.2
Neuropathy	88	9942	21896	4981	50.1	1467	14.8	3494	35.1	47.6
Neuropathy	89	8194	17701	3955	48.3	1134	13.8	3105	37.9	44.8
Neuropathy	90	6671	14107	3101	46.5	824	12.4	2746	41.2	42.5

Neuropathy	91	5332	10764	2362	44.3	604	11.3	2366	44.4	40.5
Neuropathy	92	4144	7992	1767	42.6	425	10.3	1952	47.1	37
Neuropathy	93	3073	5760	1277	41.6	261	8.5	1535	50	34.2
Neuropathy	94	2249	4090	902	40.1	158	7	1189	52.9	32
Neuropathy	95	1636	2799	631	38.6	85	5.2	920	56.2	28.9
Neuropathy	96	1115	1832	417	37.4	48	4.3	650	58.3	25.6
Neuropathy	97	734	1148	248	33.8	27	3.7	459	62.5	23.7
Neuropathy	98	469	659	156	33.3	10	2.1	303	64.6	24.3
Neuropathy	99	279	375	86	30.8	8	2.9	185	66.3	20.4
Neuropathy	100	152	197	38	25	4	2.6	110	72.4	15.8
Ulcer	19	62	210	62	100	0	0	0	0	27.4
Ulcer	20	144	526	144	100	0	0	0	0	35.4
Ulcer	21	241	919	241	100	0	0	0	0	45.6
Ulcer	22	365	1359	361	98.9	0	0	4	1.1	51
Ulcer	23	518	1906	512	98.8	0	0	6	1.2	53.1
Ulcer	24	718	2507	709	98.7	1	0.1	8	1.1	53.8
Ulcer	25	895	2991	886	99	1	0.1	8	0.9	54.3
Ulcer	26	1079	3608	1073	99.4	1	0.1	5	0.5	56
Ulcer	27	1284	4179	1273	99.1	2	0.2	9	0.7	56.2
Ulcer	28	1504	4821	1491	99.1	1	0.1	12	0.8	56.3
Ulcer	29	1720	5459	1703	99	1	0.1	16	0.9	57.4
Ulcer	30	1954	6109	1938	99.2	2	0.1	14	0.7	57.9
Ulcer	31	2224	7044	2205	99.1	3	0.1	16	0.7	58
Ulcer	32	2569	8273	2543	99	5	0.2	21	0.8	59

Ulcer	33	2972	9623	2943	99	7	0.2	22	0.7	58.6
Ulcer	34	3402	11063	3372	99.1	8	0.2	22	0.6	59.5
Ulcer	35	3861	12629	3818	98.9	9	0.2	34	0.9	60.2
Ulcer	36	4361	14461	4314	98.9	10	0.2	37	0.8	61.5
Ulcer	37	4998	16618	4946	99	7	0.1	45	0.9	61.8
Ulcer	38	5732	19221	5664	98.8	13	0.2	55	1	62.7
Ulcer	39	6682	22384	6595	98.7	19	0.3	68	1	63.5
Ulcer	40	7592	25804	7494	98.7	23	0.3	75	1	63.9
Ulcer	41	8829	29932	8713	98.7	26	0.3	90	1	64.4
Ulcer	42	10042	34118	9913	98.7	31	0.3	98	1	65
Ulcer	43	11330	38419	11173	98.6	40	0.4	117	1	65.9
Ulcer	44	12912	44044	12716	98.5	43	0.3	153	1.2	65.6
Ulcer	45	14687	50260	14446	98.4	48	0.3	193	1.3	65.9
Ulcer	46	16567	57119	16242	98	68	0.4	257	1.6	66.5
Ulcer	47	18553	63642	18189	98	74	0.4	290	1.6	67.3
Ulcer	48	20438	69945	20010	97.9	82	0.4	346	1.7	67.9
Ulcer	49	22715	77213	22244	97.9	100	0.4	371	1.6	68.4
Ulcer	50	25279	85082	24760	97.9	102	0.4	417	1.6	68.6
Ulcer	51	27836	91826	27235	97.8	121	0.4	480	1.7	69.5
Ulcer	52	30018	97874	29332	97.7	131	0.4	555	1.8	69.9
Ulcer	53	32009	103510	31249	97.6	124	0.4	636	2	70
Ulcer	54	33945	109205	33048	97.4	145	0.4	752	2.2	70.4
Ulcer	55	35917	115735	34942	97.3	154	0.4	821	2.3	70.4
Ulcer	56	37706	122570	36646	97.2	160	0.4	900	2.4	70.7

Ulcer	57	39562	129505	38371	97	183	0.5	1008	2.5	71
Ulcer	58	41575	135990	40182	96.6	191	0.5	1202	2.9	71.5
Ulcer	59	43992	143637	42457	96.5	188	0.4	1347	3.1	71.7
Ulcer	60	46606	152112	44862	96.3	191	0.4	1553	3.3	71.4
Ulcer	61	49845	162807	47854	96	204	0.4	1787	3.6	71.6
Ulcer	62	52268	170762	50054	95.8	212	0.4	2002	3.8	71.8
Ulcer	63	54719	179840	52212	95.4	237	0.4	2270	4.1	71.5
Ulcer	64	56965	188226	54175	95.1	236	0.4	2554	4.5	71.8
Ulcer	65	59427	196453	56317	94.8	235	0.4	2875	4.8	71.9
Ulcer	66	62001	203964	58531	94.4	261	0.4	3209	5.2	72.2
Ulcer	67	63965	208717	60104	94	281	0.4	3580	5.6	72.3
Ulcer	68	65313	211341	61142	93.6	276	0.4	3895	6	72.9
Ulcer	69	66216	209304	61647	93.1	302	0.5	4267	6.4	73.5
Ulcer	70	66398	205399	61581	92.7	331	0.5	4486	6.8	73.5
Ulcer	71	65709	198442	60654	92.3	319	0.5	4736	7.2	73.8
Ulcer	72	63793	188219	58516	91.7	312	0.5	4965	7.8	74.1
Ulcer	73	61599	177856	56162	91.2	316	0.5	5121	8.3	73.8
Ulcer	74	58433	165801	52870	90.5	310	0.5	5253	9	73.6
Ulcer	75	54744	154958	49075	89.6	284	0.5	5385	9.8	73.4
Ulcer	76	50913	145066	45127	88.6	276	0.5	5510	10.8	72.8
Ulcer	77	47255	135599	41287	87.4	276	0.6	5692	12	72.4
Ulcer	78	43996	126595	37736	85.8	264	0.6	5996	13.6	71.8
Ulcer	79	41269	118009	34854	84.5	262	0.6	6153	14.9	70.7
Ulcer	80	38516	108733	31871	82.7	272	0.7	6373	16.5	69.9

Ulcer	81	35601	99308	28728	80.7	264	0.7	6609	18.6	68.5
Ulcer	82	32541	89676	25584	78.6	236	0.7	6721	20.7	67.5
Ulcer	83	29607	80300	22677	76.6	234	0.8	6696	22.6	66.5
Ulcer	84	26650	71301	19734	74	215	0.8	6701	25.1	65
Ulcer	85	23875	62399	17021	71.3	198	0.8	6656	27.9	63.4
Ulcer	86	21082	53768	14476	68.7	180	0.9	6426	30.5	61.9
Ulcer	87	18268	45024	11946	65.4	152	0.8	6170	33.8	60
Ulcer	88	15732	36955	9811	62.4	139	0.9	5782	36.8	58
Ulcer	89	13010	29517	7762	59.7	103	0.8	5145	39.5	55.9
Ulcer	90	10465	23165	5916	56.5	87	0.8	4462	42.6	53.9
Ulcer	91	8299	17494	4460	53.7	67	0.8	3772	45.5	52.1
Ulcer	92	6361	12862	3251	51.1	49	0.8	3061	48.1	49
Ulcer	93	4732	9125	2322	49.1	31	0.7	2379	50.3	46.6
Ulcer	94	3441	6338	1582	46	23	0.7	1836	53.4	43.8
Ulcer	95	2445	4215	1073	43.9	13	0.5	1359	55.6	40.5
Ulcer	96	1618	2657	677	41.8	9	0.6	932	57.6	37.6
Ulcer	97	1051	1623	407	38.7	7	0.7	637	60.6	35.7
Ulcer	98	651	907	251	38.6	3	0.5	397	61	34.7
Ulcer	99	377	502	142	37.7	3	0.8	232	61.5	31.6
Ulcer	100	207	256	64	30.9	2	1	141	68.1	25.6
