# Supplementary material for: Effect and prognostic value of physical activity for lower-extremity complications of type 2 diabetes across the adult lifespan: a national cohort study using Swedish registers
Source: BMJ Open. 2026 Nov 11;16(11):e114759. doi: 10.1136/bmjopen-2025-114759 (PMC13756448; doi:10.1136/bmjopen-2025-114759)
Supplement: Supplementary file 3 [file bmjopen-16-11-s003.pdf]

Table 1.3. Descriptive statistics for the covariates included in statistical models, comparing response sample and complete cases, for each outcome and by landmark age.

Outcome	Landmark age	Variable	Factor level (if applicable)	Response sample	Missing (%)	Complete cases	Response sample vs complete cases
Amputation	19	Age at diagnosis of type 2 diabetes, mean(SD)		18.5 (0.3)	0	18.5 (0.3)	
Amputation	19	BMI, mean(SD)		34.2 (6.9)	17.7	35.1 (7.2)	
Amputation	19	HbA1c, mean(SD)		55.1 (19.7)	19.4	57.1 (19)	
Amputation	19	HDL-cholesterol, mean(SD)		0.9 (0.2)	46.8	0.9 (0.2)	
Amputation	19	Ischemic heart disease, n(%)	No	44 (100%)	29	17 (100%)	
Amputation	19	Neuropathy, n(%)	No	62 (100%)	0	17 (100%)	
Amputation	19	Self-reported physical activity, n(%)	Never	10 (25%)	35.5	5 (29.4%)	
Amputation	19	Self-reported physical activity, n(%)	<1 times/week	8 (20%)	35.5	2 (11.8%)	
Amputation	19	Self-reported physical activity, n(%)	regularly - 1-2 times/week	8 (20%)	35.5	4 (23.5%)	
Amputation	19	Self-reported physical activity, n(%)	regularly - 3-5 times/week	8 (20%)	35.5	3 (17.6%)	
Amputation	19	Self-reported physical activity, n(%)	Daily	6 (15%)	35.5	3 (17.6%)	
Amputation	19	Sex, n(%)	Males	22 (35.5%)	0	6 (35.3%)	n=62 vs n=17
Amputation	19	Sex, n(%)	Females	40 (64.5%)	0	11 (64.7%)	
Amputation	19	Smoking status, n(%)	No	28 (68.3%)	33.9	10 (58.8%)	
Amputation	19	Smoking status, n(%)	Yes	13 (31.7%)	33.9	7 (41.2%)	
Amputation	19	Stroke, n(%)	No	43 (100%)	30.6	17 (100%)	
Amputation	19	Systolic blood pressure, mean(SD)		124.6 (10.3)	22.6	124.1 (8.5)	
Amputation	19	Time since last healthcare visit, mean(SD)		0.3 (0.2)	0	0.1 (0.1)	
Amputation	19	Total cholesterol, mean(SD)		4.8 (1.1)	45.2	5 (1.1)	

Amputation	20	Age at diagnosis of type 2 diabetes, mean(SD)		19.1 (0.6)	0	19 (0.6)	
Amputation	20	BMI, mean(SD)		35.6 (8)	21.8	36.8 (8.2)	
Amputation	20	HbA1c, mean(SD)		55.3 (21.1)	14.8	58.1 (20.5)	
Amputation	20	HDL-cholesterol, mean(SD)		1 (0.3)	41.5	1 (0.3)	
Amputation	20	Ischemic heart disease, n(%)	No	117 (100%)	17.6	51 (100%)	
Amputation	20	Neuropathy, n(%)	No	140 (98.6%)	0	49 (96.1%)	
Amputation	20	Neuropathy, n(%)	Yes	2 (1.4%)	0	2 (3.9%)	
Amputation	20	Self-reported physical activity, n(%)	Never	16 (16.8%)	33.1	12 (23.5%)	
Amputation	20	Self-reported physical activity, n(%)	<1 times/week	13 (13.7%)	33.1	6 (11.8%)	
Amputation	20	Self-reported physical activity, n(%)	regularly - 1-2 times/week	21 (22.1%)	33.1	12 (23.5%)	
Amputation	20	Self-reported physical activity, n(%)	regularly - 3-5 times/week	27 (28.4%)	33.1	14 (27.5%)	
Amputation	20	Self-reported physical activity, n(%)	Daily	18 (18.9%)	33.1	7 (13.7%)	
Amputation	20	Sex, n(%)	Males	67 (47.2%)	0	25 (49%)	n=142 vs n=51
Amputation	20	Sex, n(%)	Females	75 (52.8%)	0	26 (51%)	
Amputation	20	Smoking status, n(%)	No	72 (72.7%)	30.3	35 (68.6%)	
Amputation	20	Smoking status, n(%)	Yes	27 (27.3%)	30.3	16 (31.4%)	
Amputation	20	Stroke, n(%)	No	116 (100%)	18.3	51 (100%)	
Amputation	20	Systolic blood pressure, mean(SD)		123.9 (11.6)	21.1	125.2 (12.9)	
Amputation	20	Time since last healthcare visit, mean(SD)		0.4 (0.4)	0	0.3 (0.3)	
Amputation	20	Total cholesterol, mean(SD)		4.7 (1)	36.6	4.8 (1.1)	
Amputation	21	Age at diagnosis of type 2 diabetes, mean(SD)		19.6 (0.8)	0	19.5 (0.8)	
Amputation	21	BMI, mean(SD)		35.7 (7.7)	20.1	35.3 (7.5)	
Amputation	21	HbA1c, mean(SD)		54.9 (20.1)	12.1	54.2 (20.6)	

Amputation	21	HDL-cholesterol, mean(SD)		1 (0.3)	31.8	1 (0.3)	
Amputation	21	Ischemic heart disease, n(%)	No	193 (99%)	18.4	109 (100%)	
Amputation	21	Ischemic heart disease, n(%)	Yes	2 (1%)	18.4	0 (0%)	
Amputation	21	Neuropathy, n(%)	No	236 (98.7%)	0	106 (97.2%)	
Amputation	21	Neuropathy, n(%)	Yes	3 (1.3%)	0	3 (2.8%)	
Amputation	21	Self-reported physical activity, n(%)	Never	31 (18.2%)	28.9	20 (18.3%)	
Amputation	21	Self-reported physical activity, n(%)	<1 times/week	33 (19.4%)	28.9	21 (19.3%)	
Amputation	21	Self-reported physical activity, n(%)	regularly - 1-2 times/week	33 (19.4%)	28.9	22 (20.2%)	
Amputation	21	Self-reported physical activity, n(%)	regularly - 3-5 times/week	33 (19.4%)	28.9	22 (20.2%)	
Amputation	21	Self-reported physical activity, n(%)	Daily	40 (23.5%)	28.9	24 (22%)	
Amputation	21	Sex, n(%)	Males	118 (49.4%)	0	57 (52.3%)	n=239 vs n=109
Amputation	21	Sex, n(%)	Females	121 (50.6%)	0	52 (47.7%)	
Amputation	21	Smoking status, n(%)	No	132 (75%)	26.4	79 (72.5%)	
Amputation	21	Smoking status, n(%)	Yes	44 (25%)	26.4	30 (27.5%)	
Amputation	21	Stroke, n(%)	No	193 (100%)	19.2	109 (100%)	
Amputation	21	Systolic blood pressure, mean(SD)		124 (13)	15.9	123.4 (13)	
Amputation	21	Time since last healthcare visit, mean(SD)		0.6 (0.6)	0	0.5 (0.5)	
Amputation	21	Total cholesterol, mean(SD)		4.8 (1)	28	4.8 (1)	
Amputation	22	Age at diagnosis of type 2 diabetes, mean(SD)		20.3 (1.1)	0	20.1 (1)	
Amputation	22	BMI, mean(SD)		35.6 (7.3)	18.7	35.5 (6.9)	
Amputation	22	HbA1c, mean(SD)		55.8 (21.8)	10.5	55.6 (21.3)	
Amputation	22	HDL-cholesterol, mean(SD)		1 (0.3)	27.8	1 (0.4)	
Amputation	22	Ischemic heart disease, n(%)	No	297 (99%)	17.4	184 (99.5%)	

Amputation	22	Ischemic heart disease, n(%)	Yes	3 (1%)	17.4	1 (0.5%)	
Amputation	22	Neuropathy, n(%)	No	356 (98.1%)	0	181 (97.8%)	
Amputation	22	Neuropathy, n(%)	Yes	7 (1.9%)	0	4 (2.2%)	
Amputation	22	Self-reported physical activity, n(%)	Never	49 (18.8%)	28.4	42 (22.7%)	
Amputation	22	Self-reported physical activity, n(%)	<1 times/week	46 (17.7%)	28.4	30 (16.2%)	
Amputation	22	Self-reported physical activity, n(%)	regularly - 1-2 times/week	44 (16.9%)	28.4	28 (15.1%)	
Amputation	22	Self-reported physical activity, n(%)	regularly - 3-5 times/week	53 (20.4%)	28.4	37 (20%)	
Amputation	22	Self-reported physical activity, n(%)	Daily	68 (26.2%)	28.4	48 (25.9%)	
Amputation	22	Sex, n(%)	Males	168 (46.3%)	0	86 (46.5%)	n=363 vs n=185
Amputation	22	Sex, n(%)	Females	195 (53.7%)	0	99 (53.5%)	
Amputation	22	Smoking status, n(%)	No	202 (75.1%)	25.9	142 (76.8%)	
Amputation	22	Smoking status, n(%)	Yes	67 (24.9%)	25.9	43 (23.2%)	
Amputation	22	Stroke, n(%)	No	295 (99.7%)	18.5	185 (100%)	
Amputation	22	Stroke, n(%)	Yes	1 (0.3%)	18.5	0 (0%)	
Amputation	22	Systolic blood pressure, mean(SD)		124.3 (13.7)	15.2	125.1 (13)	
Amputation	22	Time since last healthcare visit, mean(SD)		0.7 (0.8)	0	0.5 (0.6)	
Amputation	22	Total cholesterol, mean(SD)		4.9 (1.1)	24.8	4.9 (1.1)	
Amputation	23	Age at diagnosis of type 2 diabetes, mean(SD)		21 (1.3)	0	20.7 (1.3)	
Amputation	23	BMI, mean(SD)		35.8 (7.6)	18.7	35.9 (7)	
Amputation	23	HbA1c, mean(SD)		55 (19.3)	8.9	55.4 (19.9)	
Amputation	23	HDL-cholesterol, mean(SD)		1 (0.3)	27.8	1 (0.4)	
Amputation	23	Ischemic heart disease, n(%)	No	417 (99.3%)	18.9	275 (100%)	
Amputation	23	Ischemic heart disease, n(%)	Yes	3 (0.7%)	18.9	0 (0%)	

Amputation	23	Neuropathy, n(%)	No	507 (97.9%)	0	267 (97.1%)	
Amputation	23	Neuropathy, n(%)	Yes	11 (2.1%)	0	8 (2.9%)	
Amputation	23	Self-reported physical activity, n(%)	Never	71 (18.6%)	26.3	54 (19.6%)	
Amputation	23	Self-reported physical activity, n(%)	<1 times/week	63 (16.5%)	26.3	47 (17.1%)	
Amputation	23	Self-reported physical activity, n(%)	regularly - 1-2 times/week	69 (18.1%)	26.3	50 (18.2%)	
Amputation	23	Self-reported physical activity, n(%)	regularly - 3-5 times/week	87 (22.8%)	26.3	64 (23.3%)	
Amputation	23	Self-reported physical activity, n(%)	Daily	92 (24.1%)	26.3	60 (21.8%)	
Amputation	23	Sex, n(%)	Males	247 (47.7%)	0	136 (49.5%)	n=518 vs n=275
Amputation	23	Sex, n(%)	Females	271 (52.3%)	0	139 (50.5%)	
Amputation	23	Smoking status, n(%)	No	288 (72.5%)	23.4	205 (74.5%)	
Amputation	23	Smoking status, n(%)	Yes	109 (27.5%)	23.4	70 (25.5%)	
Amputation	23	Stroke, n(%)	No	415 (99.8%)	19.7	275 (100%)	
Amputation	23	Stroke, n(%)	Yes	1 (0.2%)	19.7	0 (0%)	
Amputation	23	Systolic blood pressure, mean(SD)		124.6 (13.6)	14.9	124.7 (13.6)	
Amputation	23	Time since last healthcare visit, mean(SD)		0.8 (1)	0	0.6 (0.8)	
Amputation	23	Total cholesterol, mean(SD)		4.8 (1.1)	23.9	4.8 (1.1)	
Amputation	24	Age at diagnosis of type 2 diabetes, mean(SD)		21.7 (1.6)	0	21.4 (1.6)	
Amputation	24	BMI, mean(SD)		35.7 (7.5)	16.7	36 (7.1)	
Amputation	24	HbA1c, mean(SD)		55.2 (20.9)	8.6	54.1 (19.4)	
Amputation	24	HDL-cholesterol, mean(SD)		1 (0.4)	27.9	1 (0.4)	
Amputation	24	Ischemic heart disease, n(%)	No	600 (99.2%)	15.6	385 (100%)	
Amputation	24	Ischemic heart disease, n(%)	Yes	5 (0.8%)	15.6	0 (0%)	
Amputation	24	Neuropathy, n(%)	No	703 (98%)	0	372 (96.6%)	

Amputation	24	Neuropathy, n(%)	Yes	14 (2%)	0	13 (3.4%)	
Amputation	24	Self-reported physical activity, n(%)	Never	77 (14.3%)	25	52 (13.5%)	
Amputation	24	Self-reported physical activity, n(%)	<1 times/week	90 (16.7%)	25	65 (16.9%)	
Amputation	24	Self-reported physical activity, n(%)	regularly - 1-2 times/week	112 (20.8%)	25	83 (21.6%)	
Amputation	24	Self-reported physical activity, n(%)	regularly - 3-5 times/week	130 (24.2%)	25	96 (24.9%)	
Amputation	24	Self-reported physical activity, n(%)	Daily	129 (24%)	25	89 (23.1%)	
Amputation	24	Sex, n(%)	Males	335 (46.7%)	0	181 (47%)	n=717 vs n=385
Amputation	24	Sex, n(%)	Females	382 (53.3%)	0	204 (53%)	
Amputation	24	Smoking status, n(%)	No	420 (72.7%)	19.4	285 (74%)	
Amputation	24	Smoking status, n(%)	Yes	158 (27.3%)	19.4	100 (26%)	
Amputation	24	Stroke, n(%)	No	597 (99.5%)	16.3	383 (99.5%)	
Amputation	24	Stroke, n(%)	Yes	3 (0.5%)	16.3	2 (0.5%)	
Amputation	24	Systolic blood pressure, mean(SD)		124.3 (13.7)	12.7	124.5 (13.6)	
Amputation	24	Time since last healthcare visit, mean(SD)		0.9 (1.1)	0	0.7 (0.9)	
Amputation	24	Total cholesterol, mean(SD)		4.9 (1.3)	25.1	4.8 (1.2)	
Amputation	25	Age at diagnosis of type 2 diabetes, mean(SD)		22.4 (1.8)	0	22.1 (1.7)	
Amputation	25	BMI, mean(SD)		35.7 (7.8)	15.5	35.4 (7.5)	
Amputation	25	HbA1c, mean(SD)		53.6 (20.1)	7.5	53 (19.4)	
Amputation	25	HDL-cholesterol, mean(SD)		1 (0.4)	26.3	1 (0.4)	
Amputation	25	Ischemic heart disease, n(%)	No	756 (99.3%)	14.7	485 (100%)	
Amputation	25	Ischemic heart disease, n(%)	Yes	5 (0.7%)	14.7	0 (0%)	
Amputation	25	Neuropathy, n(%)	No	872 (97.8%)	0	467 (96.3%)	
Amputation	25	Neuropathy, n(%)	Yes	20 (2.2%)	0	18 (3.7%)	

Amputation	25	Self-reported physical activity, n(%)	Never	107 (15.6%)	23.2	72 (14.8%)	
Amputation	25	Self-reported physical activity, n(%)	<1 times/week	108 (15.8%)	23.2	81 (16.7%)	
Amputation	25	Self-reported physical activity, n(%)	regularly - 1-2 times/week	136 (19.9%)	23.2	93 (19.2%)	
Amputation	25	Self-reported physical activity, n(%)	regularly - 3-5 times/week	164 (23.9%)	23.2	124 (25.6%)	
Amputation	25	Self-reported physical activity, n(%)	Daily	170 (24.8%)	23.2	115 (23.7%)	
Amputation	25	Sex, n(%)	Males	419 (47%)	0	235 (48.5%)	n=892 vs n=485
Amputation	25	Sex, n(%)	Females	473 (53%)	0	250 (51.5%)	
Amputation	25	Smoking status, n(%)	No	543 (74.7%)	18.5	369 (76.1%)	
Amputation	25	Smoking status, n(%)	Yes	184 (25.3%)	18.5	116 (23.9%)	
Amputation	25	Stroke, n(%)	No	749 (99.6%)	15.7	484 (99.8%)	
Amputation	25	Stroke, n(%)	Yes	3 (0.4%)	15.7	1 (0.2%)	
Amputation	25	Systolic blood pressure, mean(SD)		123.8 (13.4)	10.5	123.5 (13)	
Amputation	25	Time since last healthcare visit, mean(SD)		0.9 (1.3)	0	0.7 (1)	
Amputation	25	Total cholesterol, mean(SD)		4.8 (1.2)	23.4	4.8 (1.1)	
Amputation	26	Age at diagnosis of type 2 diabetes, mean(SD)		23.1 (2)	0	22.8 (1.9)	
Amputation	26	BMI, mean(SD)		35.4 (7.7)	14.1	35.2 (7.2)	
Amputation	26	HbA1c, mean(SD)		53.5 (19.4)	7.7	52.7 (19.6)	
Amputation	26	HDL-cholesterol, mean(SD)		1 (0.3)	25.8	1 (0.3)	
Amputation	26	Ischemic heart disease, n(%)	No	917 (99.5%)	14.4	602 (99.8%)	
Amputation	26	Ischemic heart disease, n(%)	Yes	5 (0.5%)	14.4	1 (0.2%)	
Amputation	26	Neuropathy, n(%)	No	1049 (97.4%)	0	579 (96%)	
Amputation	26	Neuropathy, n(%)	Yes	28 (2.6%)	0	24 (4%)	
Amputation	26	Self-reported physical activity, n(%)	Never	120 (14.3%)	22.1	84 (13.9%)	

Amputation	26	Self-reported physical activity, n(%)	<1 times/week	113 (13.5%)	22.1	81 (13.4%)	
Amputation	26	Self-reported physical activity, n(%)	regularly - 1-2 times/week	164 (19.5%)	22.1	120 (19.9%)	
Amputation	26	Self-reported physical activity, n(%)	regularly - 3-5 times/week	231 (27.5%)	22.1	171 (28.4%)	
Amputation	26	Self-reported physical activity, n(%)	Daily	211 (25.1%)	22.1	147 (24.4%)	
Amputation	26	Sex, n(%)	Males	544 (50.5%)	0	303 (50.2%)	n=1077 vs n=603
Amputation	26	Sex, n(%)	Females	533 (49.5%)	0	300 (49.8%)	
Amputation	26	Smoking status, n(%)	No	682 (76.3%)	17	460 (76.3%)	
Amputation	26	Smoking status, n(%)	Yes	212 (23.7%)	17	143 (23.7%)	
Amputation	26	Stroke, n(%)	No	906 (99.6%)	15.5	601 (99.7%)	
Amputation	26	Stroke, n(%)	Yes	4 (0.4%)	15.5	2 (0.3%)	
Amputation	26	Systolic blood pressure, mean(SD)		124.4 (13.4)	10.1	124.4 (13.3)	
Amputation	26	Time since last healthcare visit, mean(SD)		1 (1.4)	0	0.7 (1)	
Amputation	26	Total cholesterol, mean(SD)		4.9 (1.2)	22.7	4.8 (1.2)	
Amputation	27	Age at diagnosis of type 2 diabetes, mean(SD)		24 (2.1)	0	23.6 (2.1)	
Amputation	27	BMI, mean(SD)		35 (7.5)	15.3	35.2 (7.3)	
Amputation	27	HbA1c, mean(SD)		53 (18.9)	7.3	53.5 (19.2)	
Amputation	27	HDL-cholesterol, mean(SD)		1 (0.3)	26.1	1 (0.4)	
Amputation	27	Ischemic heart disease, n(%)	No	1097 (99.3%)	13.8	718 (99.6%)	
Amputation	27	Ischemic heart disease, n(%)	Yes	8 (0.7%)	13.8	3 (0.4%)	
Amputation	27	Neuropathy, n(%)	No	1239 (96.6%)	0	686 (95.1%)	
Amputation	27	Neuropathy, n(%)	Yes	43 (3.4%)	0	35 (4.9%)	
Amputation	27	Self-reported physical activity, n(%)	Never	132 (13.4%)	23.2	96 (13.3%)	
Amputation	27	Self-reported physical activity, n(%)	<1 times/week	137 (13.9%)	23.2	98 (13.6%)	

Amputation	27	Self-reported physical activity, n(%)	regularly - 1-2 times/week	188 (19.1%)	23.2	137 (19%)	
Amputation	27	Self-reported physical activity, n(%)	regularly - 3-5 times/week	284 (28.9%)	23.2	224 (31.1%)	
Amputation	27	Self-reported physical activity, n(%)	Daily	243 (24.7%)	23.2	166 (23%)	
Amputation	27	Sex, n(%)	Males	648 (50.6%)	0.1	379 (52.6%)	n=1282 vs n=721
Amputation	27	Sex, n(%)	Females	633 (49.4%)	0.1	342 (47.4%)	
Amputation	27	Smoking status, n(%)	No	797 (75.5%)	17.7	546 (75.7%)	
Amputation	27	Smoking status, n(%)	Yes	258 (24.5%)	17.7	175 (24.3%)	
Amputation	27	Stroke, n(%)	No	1090 (99.3%)	14.4	718 (99.6%)	
Amputation	27	Stroke, n(%)	Yes	8 (0.7%)	14.4	3 (0.4%)	
Amputation	27	Systolic blood pressure, mean(SD)		124.6 (13.8)	10	124.8 (14)	
Amputation	27	Time since last healthcare visit, mean(SD)		1 (1.5)	0	0.7 (1.1)	
Amputation	27	Total cholesterol, mean(SD)		4.8 (1.2)	21.6	4.8 (1.2)	
Amputation	28	Age at diagnosis of type 2 diabetes, mean(SD)		24.8 (2.3)	0	24.4 (2.2)	
Amputation	28	BMI, mean(SD)		35.1 (7.5)	15	35.1 (7.2)	
Amputation	28	HbA1c, mean(SD)		52.9 (18.6)	7.5	53.7 (18.2)	
Amputation	28	HDL-cholesterol, mean(SD)		1 (0.3)	26	1 (0.3)	
Amputation	28	Ischemic heart disease, n(%)	No	1276 (98.8%)	14	836 (98.8%)	
Amputation	28	Ischemic heart disease, n(%)	Yes	15 (1.2%)	14	10 (1.2%)	
Amputation	28	Neuropathy, n(%)	No	1452 (96.7%)	0	805 (95.2%)	
Amputation	28	Neuropathy, n(%)	Yes	50 (3.3%)	0	41 (4.8%)	
Amputation	28	Self-reported physical activity, n(%)	Never	151 (13%)	22.8	110 (13%)	
Amputation	28	Self-reported physical activity, n(%)	<1 times/week	126 (10.9%)	22.8	88 (10.4%)	
Amputation	28	Self-reported physical activity, n(%)	regularly - 1-2 times/week	254 (21.9%)	22.8	181 (21.4%)	

Amputation	28	Self-reported physical activity, n(%)	regularly - 3-5 times/week	320 (27.6%)	22.8	243 (28.7%)	
Amputation	28	Self-reported physical activity, n(%)	Daily	308 (26.6%)	22.8	224 (26.5%)	
Amputation	28	Sex, n(%)	Males	747 (49.7%)	0	438 (51.8%)	n=1502 vs n=846
Amputation	28	Sex, n(%)	Females	755 (50.3%)	0	408 (48.2%)	
Amputation	28	Smoking status, n(%)	No	941 (76%)	17.6	651 (77%)	
Amputation	28	Smoking status, n(%)	Yes	297 (24%)	17.6	195 (23%)	
Amputation	28	Stroke, n(%)	No	1277 (99.1%)	14.2	839 (99.2%)	
Amputation	28	Stroke, n(%)	Yes	11 (0.9%)	14.2	7 (0.8%)	
Amputation	28	Systolic blood pressure, mean(SD)		124.8 (13.4)	10.1	125.4 (13.5)	
Amputation	28	Time since last healthcare visit, mean(SD)		1 (1.5)	0	0.8 (1.1)	
Amputation	28	Total cholesterol, mean(SD)		4.8 (1.1)	21.5	4.8 (1.1)	
Amputation	29	Age at diagnosis of type 2 diabetes, mean(SD)		25.7 (2.4)	0	25.2 (2.4)	
Amputation	29	BMI, mean(SD)		34.8 (7.5)	14.4	34.7 (7.1)	
Amputation	29	HbA1c, mean(SD)		53.7 (18.9)	7	54 (18.4)	
Amputation	29	HDL-cholesterol, mean(SD)		1 (0.3)	25.7	1 (0.3)	
Amputation	29	Ischemic heart disease, n(%)	No	1474 (99.1%)	13.5	980 (99.2%)	
Amputation	29	Ischemic heart disease, n(%)	Yes	13 (0.9%)	13.5	8 (0.8%)	
Amputation	29	Neuropathy, n(%)	No	1649 (95.9%)	0	927 (93.8%)	
Amputation	29	Neuropathy, n(%)	Yes	70 (4.1%)	0	61 (6.2%)	
Amputation	29	Self-reported physical activity, n(%)	Never	174 (13.1%)	22.5	127 (12.9%)	
Amputation	29	Self-reported physical activity, n(%)	<1 times/week	164 (12.3%)	22.5	122 (12.3%)	
Amputation	29	Self-reported physical activity, n(%)	regularly - 1-2 times/week	280 (21%)	22.5	200 (20.2%)	
Amputation	29	Self-reported physical activity, n(%)	regularly - 3-5 times/week	353 (26.5%)	22.5	280 (28.3%)	

Amputation	29	Self-reported physical activity, n(%)	Daily	362 (27.2%)	22.5	259 (26.2%)	
Amputation	29	Sex, n(%)	Males	877 (51%)	0	512 (51.8%)	n=1719 vs n=988
Amputation	29	Sex, n(%)	Females	842 (49%)	0	476 (48.2%)	
Amputation	29	Smoking status, n(%)	No	1108 (77.4%)	16.8	757 (76.6%)	
Amputation	29	Smoking status, n(%)	Yes	323 (22.6%)	16.8	231 (23.4%)	
Amputation	29	Stroke, n(%)	No	1469 (99.3%)	13.9	982 (99.4%)	
Amputation	29	Stroke, n(%)	Yes	11 (0.7%)	13.9	6 (0.6%)	
Amputation	29	Systolic blood pressure, mean(SD)		124.5 (13.6)	9.4	124.7 (13.5)	
Amputation	29	Time since last healthcare visit, mean(SD)		1.1 (1.5)	0	0.8 (1.1)	
Amputation	29	Total cholesterol, mean(SD)		4.8 (1.1)	21.2	4.8 (1.1)	
Amputation	30	Age at diagnosis of type 2 diabetes, mean(SD)		26.6 (2.5)	0	26.2 (2.6)	
Amputation	30	BMI, mean(SD)		34.7 (7.6)	14	34.6 (7.4)	
Amputation	30	HbA1c, mean(SD)		53.6 (19.2)	6.8	54 (18.7)	
Amputation	30	HDL-cholesterol, mean(SD)		1 (0.3)	25.5	1 (0.3)	
Amputation	30	Ischemic heart disease, n(%)	No	1680 (99.2%)	13.4	1124 (99.3%)	
Amputation	30	Ischemic heart disease, n(%)	Yes	13 (0.8%)	13.4	8 (0.7%)	
Amputation	30	Neuropathy, n(%)	No	1878 (96.1%)	0	1063 (93.9%)	
Amputation	30	Neuropathy, n(%)	Yes	76 (3.9%)	0	69 (6.1%)	
Amputation	30	Self-reported physical activity, n(%)	Never	190 (12.3%)	21	148 (13.1%)	
Amputation	30	Self-reported physical activity, n(%)	<1 times/week	207 (13.4%)	21	141 (12.5%)	
Amputation	30	Self-reported physical activity, n(%)	regularly - 1-2 times/week	339 (22%)	21	247 (21.8%)	
Amputation	30	Self-reported physical activity, n(%)	regularly - 3-5 times/week	400 (25.9%)	21	292 (25.8%)	
Amputation	30	Self-reported physical activity, n(%)	Daily	408 (26.4%)	21	304 (26.9%)	

Amputation	30	Sex, n(%)	Males	1025 (52.5%)	0	618 (54.6%)	n=1954 vs n=1132
Amputation	30	Sex, n(%)	Females	929 (47.5%)	0	514 (45.4%)	
Amputation	30	Smoking status, n(%)	No	1276 (77.4%)	15.6	890 (78.6%)	
Amputation	30	Smoking status, n(%)	Yes	373 (22.6%)	15.6	242 (21.4%)	
Amputation	30	Stroke, n(%)	No	1676 (99.3%)	13.7	1125 (99.4%)	
Amputation	30	Stroke, n(%)	Yes	11 (0.7%)	13.7	7 (0.6%)	
Amputation	30	Systolic blood pressure, mean(SD)		124.6 (13.7)	8.6	125.2 (13.7)	
Amputation	30	Time since last healthcare visit, mean(SD)		1 (1.6)	0	0.7 (1.1)	
Amputation	30	Total cholesterol, mean(SD)		4.8 (1.2)	21.6	4.8 (1.1)	
Amputation	31	Age at diagnosis of type 2 diabetes, mean(SD)		27.6 (2.6)	0	27 (2.7)	
Amputation	31	BMI, mean(SD)		34.3 (7.4)	13.4	34.2 (7)	
Amputation	31	HbA1c, mean(SD)		53.3 (18.6)	6.3	53.6 (18)	
Amputation	31	HDL-cholesterol, mean(SD)		1 (0.3)	24.9	1 (0.3)	
Amputation	31	Ischemic heart disease, n(%)	No	1934 (99.3%)	12.5	1283 (99.3%)	
Amputation	31	Ischemic heart disease, n(%)	Yes	13 (0.7%)	12.5	9 (0.7%)	
Amputation	31	Neuropathy, n(%)	No	2138 (96.1%)	0	1222 (94.6%)	
Amputation	31	Neuropathy, n(%)	Yes	86 (3.9%)	0	70 (5.4%)	
Amputation	31	Self-reported physical activity, n(%)	Never	218 (12.4%)	21.2	164 (12.7%)	
Amputation	31	Self-reported physical activity, n(%)	<1 times/week	233 (13.3%)	21.2	163 (12.6%)	
Amputation	31	Self-reported physical activity, n(%)	regularly - 1-2 times/week	373 (21.3%)	21.2	274 (21.2%)	
Amputation	31	Self-reported physical activity, n(%)	regularly - 3-5 times/week	459 (26.2%)	21.2	344 (26.6%)	
Amputation	31	Self-reported physical activity, n(%)	Daily	470 (26.8%)	21.2	347 (26.9%)	
Amputation	31	Sex, n(%)	Males	1201 (54%)	0	730 (56.5%)	n=2224 vs n=1292

Amputation	31	Sex, n(%)	Females	1023 (46%)	0	562 (43.5%)	
Amputation	31	Smoking status, n(%)	No	1463 (77%)	14.6	1002 (77.6%)	
Amputation	31	Smoking status, n(%)	Yes	436 (23%)	14.6	290 (22.4%)	
Amputation	31	Stroke, n(%)	No	1928 (99.3%)	12.7	1280 (99.1%)	
Amputation	31	Stroke, n(%)	Yes	14 (0.7%)	12.7	12 (0.9%)	
Amputation	31	Systolic blood pressure, mean(SD)		124.8 (13.8)	8.7	125.1 (13.7)	
Amputation	31	Time since last healthcare visit, mean(SD)		1 (1.5)	0	0.7 (1)	
Amputation	31	Total cholesterol, mean(SD)		4.8 (1.2)	20.4	4.8 (1.2)	
Amputation	32	Age at diagnosis of type 2 diabetes, mean(SD)		28.6 (2.6)	0	28.1 (2.7)	
Amputation	32	BMI, mean(SD)		34.1 (7.5)	13.1	33.9 (7.4)	
Amputation	32	HbA1c, mean(SD)		53 (18.7)	5.9	53.5 (18.2)	
Amputation	32	HDL-cholesterol, mean(SD)		1 (0.3)	23.9	1 (0.3)	
Amputation	32	Ischemic heart disease, n(%)	No	2241 (99.3%)	12.2	1507 (99.4%)	
Amputation	32	Ischemic heart disease, n(%)	Yes	15 (0.7%)	12.2	9 (0.6%)	
Amputation	32	Neuropathy, n(%)	No	2463 (95.8%)	0	1433 (94.5%)	
Amputation	32	Neuropathy, n(%)	Yes	107 (4.2%)	0	83 (5.5%)	
Amputation	32	Self-reported physical activity, n(%)	Never	277 (13.5%)	20.1	206 (13.6%)	
Amputation	32	Self-reported physical activity, n(%)	<1 times/week	264 (12.9%)	20.1	187 (12.3%)	
Amputation	32	Self-reported physical activity, n(%)	regularly - 1-2 times/week	432 (21%)	20.1	307 (20.3%)	
Amputation	32	Self-reported physical activity, n(%)	regularly - 3-5 times/week	541 (26.4%)	20.1	427 (28.2%)	
Amputation	32	Self-reported physical activity, n(%)	Daily	539 (26.3%)	20.1	389 (25.7%)	
Amputation	32	Sex, n(%)	Males	1401 (54.5%)	0	869 (57.3%)	n=2570 vs n=1516
Amputation	32	Sex, n(%)	Females	1169 (45.5%)	0	647 (42.7%)	

Amputation	32	Smoking status, n(%)	No	1714 (77.5%)	13.9	1191 (78.6%)	
Amputation	32	Smoking status, n(%)	Yes	499 (22.5%)	13.9	325 (21.4%)	
Amputation	32	Stroke, n(%)	No	2240 (99.4%)	12.3	1505 (99.3%)	
Amputation	32	Stroke, n(%)	Yes	13 (0.6%)	12.3	11 (0.7%)	
Amputation	32	Systolic blood pressure, mean(SD)		124.9 (14.2)	7.6	125.3 (14)	
Amputation	32	Time since last healthcare visit, mean(SD)		1 (1.4)	0	0.7 (1.1)	
Amputation	32	Total cholesterol, mean(SD)		4.8 (1.2)	19.4	4.8 (1.2)	
Amputation	33	Age at diagnosis of type 2 diabetes, mean(SD)		29.6 (2.6)	0	29.1 (2.7)	
Amputation	33	BMI, mean(SD)		34 (7.5)	13	34 (7.5)	
Amputation	33	HbA1c, mean(SD)		52.9 (17.9)	5.2	53 (17.1)	
Amputation	33	HDL-cholesterol, mean(SD)		1 (0.3)	23	1.1 (0.3)	
Amputation	33	Ischemic heart disease, n(%)	No	2585 (99.2%)	12.5	1734 (99.4%)	
Amputation	33	Ischemic heart disease, n(%)	Yes	21 (0.8%)	12.5	10 (0.6%)	
Amputation	33	Neuropathy, n(%)	No	2858 (96%)	0	1655 (94.9%)	
Amputation	33	Neuropathy, n(%)	Yes	119 (4%)	0	89 (5.1%)	
Amputation	33	Self-reported physical activity, n(%)	Never	327 (13.7%)	20	251 (14.4%)	
Amputation	33	Self-reported physical activity, n(%)	<1 times/week	316 (13.3%)	20	224 (12.8%)	
Amputation	33	Self-reported physical activity, n(%)	regularly - 1-2 times/week	502 (21.1%)	20	352 (20.2%)	
Amputation	33	Self-reported physical activity, n(%)	regularly - 3-5 times/week	589 (24.7%)	20	434 (24.9%)	
Amputation	33	Self-reported physical activity, n(%)	Daily	648 (27.2%)	20	483 (27.7%)	
Amputation	33	Sex, n(%)	Males	1656 (55.6%)	0	989 (56.7%)	n=2977 vs n=1744
Amputation	33	Sex, n(%)	Females	1321 (44.4%)	0	755 (43.3%)	
Amputation	33	Smoking status, n(%)	No	1971 (76.4%)	13.3	1348 (77.3%)	

Amputation	33	Smoking status, n(%)	Yes	610 (23.6%)	13.3	396 (22.7%)	
Amputation	33	Stroke, n(%)	No	2587 (99.5%)	12.7	1734 (99.4%)	
Amputation	33	Stroke, n(%)	Yes	13 (0.5%)	12.7	10 (0.6%)	
Amputation	33	Systolic blood pressure, mean(SD)		125 (14.2)	7.3	125.1 (13.9)	
Amputation	33	Time since last healthcare visit, mean(SD)		0.9 (1.4)	0	0.7 (1)	
Amputation	33	Total cholesterol, mean(SD)		4.9 (1.2)	18.5	4.8 (1.2)	
Amputation	34	Age at diagnosis of type 2 diabetes, mean(SD)		30.6 (2.6)	0	30.1 (2.6)	
Amputation	34	BMI, mean(SD)		33.9 (7.5)	11.8	33.8 (7.4)	
Amputation	34	HbA1c, mean(SD)		52.6 (17.4)	4.4	52.6 (16.7)	
Amputation	34	HDL-cholesterol, mean(SD)		1.1 (0.3)	22.4	1.1 (0.3)	
Amputation	34	Ischemic heart disease, n(%)	No	2971 (98.9%)	11.9	2007 (99.1%)	
Amputation	34	Ischemic heart disease, n(%)	Yes	32 (1.1%)	11.9	19 (0.9%)	
Amputation	34	Neuropathy, n(%)	No	3248 (95.3%)	0	1909 (94.2%)	
Amputation	34	Neuropathy, n(%)	Yes	159 (4.7%)	0	117 (5.8%)	
Amputation	34	Self-reported physical activity, n(%)	Never	371 (13.5%)	19.5	268 (13.2%)	
Amputation	34	Self-reported physical activity, n(%)	<1 times/week	345 (12.6%)	19.5	254 (12.5%)	
Amputation	34	Self-reported physical activity, n(%)	regularly - 1-2 times/week	595 (21.7%)	19.5	433 (21.4%)	
Amputation	34	Self-reported physical activity, n(%)	regularly - 3-5 times/week	695 (25.3%)	19.5	517 (25.5%)	
Amputation	34	Self-reported physical activity, n(%)	Daily	736 (26.8%)	19.5	554 (27.3%)	
Amputation	34	Sex, n(%)	Males	1902 (55.8%)	0	1171 (57.8%)	n=3407 vs n=2026
Amputation	34	Sex, n(%)	Females	1504 (44.2%)	0	855 (42.2%)	
Amputation	34	Smoking status, n(%)	No	2307 (78.2%)	13.4	1604 (79.2%)	
Amputation	34	Smoking status, n(%)	Yes	645 (21.8%)	13.4	422 (20.8%)	

Amputation	34	Stroke, n(%)	No	2982 (99.5%)	12	2015 (99.5%)	
Amputation	34	Stroke, n(%)	Yes	15 (0.5%)	12	11 (0.5%)	
Amputation	34	Systolic blood pressure, mean(SD)		125.5 (14.4)	7.4	125.4 (14.1)	
Amputation	34	Time since last healthcare visit, mean(SD)		0.9 (1.4)	0	0.7 (1)	
Amputation	34	Total cholesterol, mean(SD)		4.9 (1.1)	17.7	4.8 (1.1)	
Amputation	35	Age at diagnosis of type 2 diabetes, mean(SD)		31.6 (2.6)	0	31.1 (2.6)	
Amputation	35	BMI, mean(SD)		33.9 (7.4)	11.9	33.8 (7.4)	
Amputation	35	HbA1c, mean(SD)		52.6 (17.5)	4.4	52.6 (16.8)	
Amputation	35	HDL-cholesterol, mean(SD)		1.1 (0.3)	21.1	1.1 (0.3)	
Amputation	35	Ischemic heart disease, n(%)	No	3358 (99%)	12.2	2302 (98.9%)	
Amputation	35	Ischemic heart disease, n(%)	Yes	33 (1%)	12.2	25 (1.1%)	
Amputation	35	Neuropathy, n(%)	No	3676 (95.2%)	0	2182 (93.8%)	
Amputation	35	Neuropathy, n(%)	Yes	187 (4.8%)	0	145 (6.2%)	
Amputation	35	Self-reported physical activity, n(%)	Never	443 (14.2%)	19.4	324 (13.9%)	
Amputation	35	Self-reported physical activity, n(%)	<1 times/week	386 (12.4%)	19.4	279 (12%)	
Amputation	35	Self-reported physical activity, n(%)	regularly - 1-2 times/week	670 (21.5%)	19.4	510 (21.9%)	
Amputation	35	Self-reported physical activity, n(%)	regularly - 3-5 times/week	780 (25%)	19.4	582 (25%)	
Amputation	35	Self-reported physical activity, n(%)	Daily	835 (26.8%)	19.4	632 (27.2%)	
Amputation	35	Sex, n(%)	Males	2171 (56.2%)	0.1	1356 (58.3%)	n=3863 vs n=2327
Amputation	35	Sex, n(%)	Females	1690 (43.8%)	0.1	971 (41.7%)	
Amputation	35	Smoking status, n(%)	No	2658 (79.3%)	13.3	1867 (80.2%)	
Amputation	35	Smoking status, n(%)	Yes	693 (20.7%)	13.3	460 (19.8%)	
Amputation	35	Stroke, n(%)	No	3375 (99.6%)	12.3	2315 (99.5%)	

Amputation	35	Stroke, n(%)	Yes	14 (0.4%)	12.3	12 (0.5%)	
Amputation	35	Systolic blood pressure, mean(SD)		125.6 (14.4)	7.1	125.3 (14.2)	
Amputation	35	Time since last healthcare visit, mean(SD)		0.9 (1.4)	0	0.7 (1)	
Amputation	35	Total cholesterol, mean(SD)		4.9 (1.1)	16.9	4.8 (1.1)	
Amputation	36	Age at diagnosis of type 2 diabetes, mean(SD)		32.6 (2.5)	0	32.2 (2.6)	
Amputation	36	BMI, mean(SD)		33.7 (7.2)	11.2	33.6 (7.2)	
Amputation	36	HbA1c, mean(SD)		52.6 (17.6)	4	52.9 (17)	
Amputation	36	HDL-cholesterol, mean(SD)		1.1 (0.3)	20.8	1.1 (0.3)	
Amputation	36	Ischemic heart disease, n(%)	No	3770 (98.8%)	12.5	2657 (98.9%)	
Amputation	36	Ischemic heart disease, n(%)	Yes	45 (1.2%)	12.5	30 (1.1%)	
Amputation	36	Neuropathy, n(%)	No	4143 (95%)	0	2512 (93.5%)	
Amputation	36	Neuropathy, n(%)	Yes	217 (5%)	0	175 (6.5%)	
Amputation	36	Self-reported physical activity, n(%)	Never	535 (15%)	18.3	394 (14.7%)	
Amputation	36	Self-reported physical activity, n(%)	<1 times/week	450 (12.6%)	18.3	327 (12.2%)	
Amputation	36	Self-reported physical activity, n(%)	regularly - 1-2 times/week	744 (20.9%)	18.3	581 (21.6%)	
Amputation	36	Self-reported physical activity, n(%)	regularly - 3-5 times/week	877 (24.6%)	18.3	654 (24.3%)	
Amputation	36	Self-reported physical activity, n(%)	Daily	957 (26.9%)	18.3	731 (27.2%)	
Amputation	36	Sex, n(%)	Males	2474 (56.8%)	0	1607 (59.8%)	n=4360 vs n=2687
Amputation	36	Sex, n(%)	Females	1884 (43.2%)	0	1080 (40.2%)	
Amputation	36	Smoking status, n(%)	No	3034 (79%)	11.9	2159 (80.3%)	
Amputation	36	Smoking status, n(%)	Yes	807 (21%)	11.9	528 (19.7%)	
Amputation	36	Stroke, n(%)	No	3790 (99.5%)	12.6	2669 (99.3%)	
Amputation	36	Stroke, n(%)	Yes	19 (0.5%)	12.6	18 (0.7%)	

Amputation	36	Systolic blood pressure, mean(SD)		125.9 (14.4)	7.2	125.9 (14.1)	
Amputation	36	Time since last healthcare visit, mean(SD)		0.9 (1.4)	0	0.7 (1)	
Amputation	36	Total cholesterol, mean(SD)		4.9 (1.2)	16.4	4.9 (1.2)	
Amputation	37	Age at diagnosis of type 2 diabetes, mean(SD)		33.5 (2.6)	0	33.1 (2.6)	
Amputation	37	BMI, mean(SD)		33.6 (7.2)	10.4	33.5 (7.1)	
Amputation	37	HbA1c, mean(SD)		52.7 (17.5)	3.8	52.7 (16.9)	
Amputation	37	HDL-cholesterol, mean(SD)		1.1 (0.3)	20.3	1.1 (0.3)	
Amputation	37	Ischemic heart disease, n(%)	No	4354 (98.6%)	11.7	3061 (98.7%)	
Amputation	37	Ischemic heart disease, n(%)	Yes	64 (1.4%)	11.7	39 (1.3%)	
Amputation	37	Neuropathy, n(%)	No	4733 (94.6%)	0	2887 (93.1%)	
Amputation	37	Neuropathy, n(%)	Yes	270 (5.4%)	0	213 (6.9%)	
Amputation	37	Self-reported physical activity, n(%)	Never	602 (14.7%)	18.1	443 (14.3%)	
Amputation	37	Self-reported physical activity, n(%)	<1 times/week	556 (13.6%)	18.1	415 (13.4%)	
Amputation	37	Self-reported physical activity, n(%)	regularly - 1-2 times/week	840 (20.5%)	18.1	658 (21.2%)	
Amputation	37	Self-reported physical activity, n(%)	regularly - 3-5 times/week	994 (24.3%)	18.1	740 (23.9%)	
Amputation	37	Self-reported physical activity, n(%)	Daily	1106 (27%)	18.1	844 (27.2%)	
Amputation	37	Sex, n(%)	Males	2853 (57%)	0	1862 (60.1%)	n=5003 vs n=3100
Amputation	37	Sex, n(%)	Females	2148 (43%)	0	1238 (39.9%)	
Amputation	37	Smoking status, n(%)	No	3525 (79.5%)	11.4	2505 (80.8%)	
Amputation	37	Smoking status, n(%)	Yes	909 (20.5%)	11.4	595 (19.2%)	
Amputation	37	Stroke, n(%)	No	4372 (99.2%)	11.9	3075 (99.2%)	
Amputation	37	Stroke, n(%)	Yes	35 (0.8%)	11.9	25 (0.8%)	
Amputation	37	Systolic blood pressure, mean(SD)		126.1 (14.2)	5.8	125.9 (13.8)	

Amputation	37	Time since last healthcare visit, mean(SD)		0.9 (1.4)	0	0.7 (1)	
Amputation	37	Total cholesterol, mean(SD)		4.9 (1.2)	16	4.8 (1.2)	
Amputation	38	Age at diagnosis of type 2 diabetes, mean(SD)		34.5 (2.6)	0	34.1 (2.6)	
Amputation	38	BMI, mean(SD)		33.5 (7.1)	11.1	33.4 (7)	
Amputation	38	HbA1c, mean(SD)		52.4 (17.3)	3.9	52.7 (17)	
Amputation	38	HDL-cholesterol, mean(SD)		1.1 (0.3)	19.4	1.1 (0.3)	
Amputation	38	Ischemic heart disease, n(%)	No	5001 (98.3%)	11.4	3545 (98.3%)	
Amputation	38	Ischemic heart disease, n(%)	Yes	85 (1.7%)	11.4	61 (1.7%)	
Amputation	38	Neuropathy, n(%)	No	5428 (94.6%)	0	3355 (93%)	
Amputation	38	Neuropathy, n(%)	Yes	312 (5.4%)	0	251 (7%)	
Amputation	38	Self-reported physical activity, n(%)	Never	687 (14.7%)	18.3	523 (14.5%)	
Amputation	38	Self-reported physical activity, n(%)	<1 times/week	612 (13.1%)	18.3	472 (13.1%)	
Amputation	38	Self-reported physical activity, n(%)	regularly - 1-2 times/week	979 (20.9%)	18.3	779 (21.6%)	
Amputation	38	Self-reported physical activity, n(%)	regularly - 3-5 times/week	1121 (23.9%)	18.3	835 (23.2%)	
Amputation	38	Self-reported physical activity, n(%)	Daily	1289 (27.5%)	18.3	997 (27.6%)	
Amputation	38	Sex, n(%)	Males	3302 (57.5%)	0	2166 (60.1%)	n=5740 vs n=3606
Amputation	38	Sex, n(%)	Females	2436 (42.5%)	0	1440 (39.9%)	
Amputation	38	Smoking status, n(%)	No	4036 (79.1%)	11.1	2899 (80.4%)	
Amputation	38	Smoking status, n(%)	Yes	1064 (20.9%)	11.1	707 (19.6%)	
Amputation	38	Stroke, n(%)	No	5031 (99.1%)	11.6	3578 (99.2%)	
Amputation	38	Stroke, n(%)	Yes	45 (0.9%)	11.6	28 (0.8%)	
Amputation	38	Systolic blood pressure, mean(SD)		126.4 (14.6)	6	126.2 (14.1)	
Amputation	38	Time since last healthcare visit, mean(SD)		0.9 (1.4)	0	0.7 (1)	

Amputation	38	Total cholesterol, mean(SD)		4.9 (1.1)	15	4.8 (1.1)	
Amputation	39	Age at diagnosis of type 2 diabetes, mean(SD)		35.5 (2.6)	0	35 (2.7)	
Amputation	39	BMI, mean(SD)		33.4 (7)	10.4	33.2 (6.8)	
Amputation	39	HbA1c, mean(SD)		52.7 (17.5)	3.6	52.8 (17.1)	
Amputation	39	HDL-cholesterol, mean(SD)		1.1 (0.3)	19.4	1.1 (0.3)	
Amputation	39	Ischemic heart disease, n(%)	No	5839 (98.4%)	11.4	4187 (98.3%)	
Amputation	39	Ischemic heart disease, n(%)	Yes	94 (1.6%)	11.4	73 (1.7%)	
Amputation	39	Neuropathy, n(%)	No	6310 (94.2%)	0	3940 (92.5%)	
Amputation	39	Neuropathy, n(%)	Yes	386 (5.8%)	0	320 (7.5%)	
Amputation	39	Self-reported physical activity, n(%)	Never	760 (13.7%)	17.1	591 (13.9%)	
Amputation	39	Self-reported physical activity, n(%)	<1 times/week	720 (13%)	17.1	542 (12.7%)	
Amputation	39	Self-reported physical activity, n(%)	regularly - 1-2 times/week	1166 (21%)	17.1	888 (20.8%)	
Amputation	39	Self-reported physical activity, n(%)	regularly - 3-5 times/week	1403 (25.3%)	17.1	1084 (25.4%)	
Amputation	39	Self-reported physical activity, n(%)	Daily	1503 (27.1%)	17.1	1155 (27.1%)	
Amputation	39	Sex, n(%)	Males	3882 (58%)	0	2562 (60.1%)	n=6696 vs n=4260
Amputation	39	Sex, n(%)	Females	2814 (42%)	0	1698 (39.9%)	
Amputation	39	Smoking status, n(%)	No	4726 (79.1%)	10.8	3430 (80.5%)	
Amputation	39	Smoking status, n(%)	Yes	1247 (20.9%)	10.8	830 (19.5%)	
Amputation	39	Stroke, n(%)	No	5866 (99.1%)	11.6	4218 (99%)	
Amputation	39	Stroke, n(%)	Yes	54 (0.9%)	11.6	42 (1%)	
Amputation	39	Systolic blood pressure, mean(SD)		126.8 (14.6)	5.5	126.7 (14.3)	
Amputation	39	Time since last healthcare visit, mean(SD)		0.8 (1.3)	0	0.7 (1)	
Amputation	39	Total cholesterol, mean(SD)		4.9 (1.2)	15.1	4.8 (1.2)	

Amputation	40	Age at diagnosis of type 2 diabetes, mean(SD)		36.5 (2.6)	0	36 (2.7)	
Amputation	40	BMI, mean(SD)		33.4 (6.8)	9.9	33.3 (6.8)	
Amputation	40	HbA1c, mean(SD)		52.7 (17.1)	3.6	52.8 (16.9)	
Amputation	40	HDL-cholesterol, mean(SD)		1.1 (0.3)	18.7	1.1 (0.3)	
Amputation	40	Ischemic heart disease, n(%)	No	6660 (98.4%)	11	4790 (98.3%)	
Amputation	40	Ischemic heart disease, n(%)	Yes	110 (1.6%)	11	83 (1.7%)	
Amputation	40	Neuropathy, n(%)	No	7163 (94.2%)	0	4503 (92.4%)	
Amputation	40	Neuropathy, n(%)	Yes	440 (5.8%)	0	370 (7.6%)	
Amputation	40	Self-reported physical activity, n(%)	Never	959 (15%)	16.2	709 (14.5%)	
Amputation	40	Self-reported physical activity, n(%)	<1 times/week	893 (14%)	16.2	678 (13.9%)	
Amputation	40	Self-reported physical activity, n(%)	regularly - 1-2 times/week	1337 (21%)	16.2	1045 (21.4%)	
Amputation	40	Self-reported physical activity, n(%)	regularly - 3-5 times/week	1524 (23.9%)	16.2	1195 (24.5%)	
Amputation	40	Self-reported physical activity, n(%)	Daily	1662 (26.1%)	16.2	1246 (25.6%)	
Amputation	40	Sex, n(%)	Males	4459 (58.7%)	0	2941 (60.4%)	n=7603 vs n=4873
Amputation	40	Sex, n(%)	Females	3143 (41.3%)	0	1932 (39.6%)	
Amputation	40	Smoking status, n(%)	No	5341 (78.5%)	10.5	3892 (79.9%)	
Amputation	40	Smoking status, n(%)	Yes	1467 (21.5%)	10.5	981 (20.1%)	
Amputation	40	Stroke, n(%)	No	6689 (99.1%)	11.2	4821 (98.9%)	
Amputation	40	Stroke, n(%)	Yes	63 (0.9%)	11.2	52 (1.1%)	
Amputation	40	Systolic blood pressure, mean(SD)		127.2 (14.6)	5.5	127.1 (14.3)	
Amputation	40	Time since last healthcare visit, mean(SD)		0.8 (1.3)	0	0.7 (1)	
Amputation	40	Total cholesterol, mean(SD)		4.9 (1.1)	14.5	4.8 (1.1)	
Amputation	41	Age at diagnosis of type 2 diabetes, mean(SD)		37.5 (2.7)	0	37 (2.7)	

Amputation	41	BMI, mean(SD)		33.3 (6.9)	9.9	33.2 (6.8)	
Amputation	41	HbA1c, mean(SD)		52.7 (16.9)	3.2	52.8 (16.3)	
Amputation	41	HDL-cholesterol, mean(SD)		1.1 (0.3)	17.8	1.1 (0.3)	
Amputation	41	Ischemic heart disease, n(%)	No	7725 (98%)	10.8	5585 (98%)	
Amputation	41	Ischemic heart disease, n(%)	Yes	161 (2%)	10.8	114 (2%)	
Amputation	41	Neuropathy, n(%)	No	8301 (93.9%)	0	5241 (92%)	
Amputation	41	Neuropathy, n(%)	Yes	537 (6.1%)	0	458 (8%)	
Amputation	41	Self-reported physical activity, n(%)	Never	1137 (15.3%)	15.8	849 (14.9%)	
Amputation	41	Self-reported physical activity, n(%)	<1 times/week	998 (13.4%)	15.8	747 (13.1%)	
Amputation	41	Self-reported physical activity, n(%)	regularly - 1-2 times/week	1589 (21.4%)	15.8	1238 (21.7%)	
Amputation	41	Self-reported physical activity, n(%)	regularly - 3-5 times/week	1793 (24.1%)	15.8	1413 (24.8%)	
Amputation	41	Self-reported physical activity, n(%)	Daily	1923 (25.8%)	15.8	1452 (25.5%)	
Amputation	41	Sex, n(%)	Males	5231 (59.2%)	0	3469 (60.9%)	n=8838 vs n=5699
Amputation	41	Sex, n(%)	Females	3606 (40.8%)	0	2230 (39.1%)	
Amputation	41	Smoking status, n(%)	No	6234 (78.4%)	10	4550 (79.8%)	
Amputation	41	Smoking status, n(%)	Yes	1721 (21.6%)	10	1149 (20.2%)	
Amputation	41	Stroke, n(%)	No	7783 (99%)	11	5636 (98.9%)	
Amputation	41	Stroke, n(%)	Yes	79 (1%)	11	63 (1.1%)	
Amputation	41	Systolic blood pressure, mean(SD)		127.7 (14.5)	4.9	127.6 (14.3)	
Amputation	41	Time since last healthcare visit, mean(SD)		0.8 (1.3)	0	0.7 (1)	
Amputation	41	Total cholesterol, mean(SD)		4.9 (1.1)	13.6	4.9 (1.1)	
Amputation	42	Age at diagnosis of type 2 diabetes, mean(SD)		38.4 (2.7)	0	37.9 (2.7)	
Amputation	42	BMI, mean(SD)		33.2 (6.7)	9.5	33.1 (6.6)	

Amputation	42	HbA1c, mean(SD)		52.5 (16.6)	3	52.8 (16.2)	
Amputation	42	HDL-cholesterol, mean(SD)		1.1 (0.3)	17.5	1.1 (0.3)	
Amputation	42	Ischemic heart disease, n(%)	No	8747 (97.8%)	11.1	6403 (97.8%)	
Amputation	42	Ischemic heart disease, n(%)	Yes	200 (2.2%)	11.1	145 (2.2%)	
Amputation	42	Neuropathy, n(%)	No	9455 (94%)	0	6037 (92.2%)	
Amputation	42	Neuropathy, n(%)	Yes	605 (6%)	0	511 (7.8%)	
Amputation	42	Self-reported physical activity, n(%)	Never	1289 (15.2%)	15.7	958 (14.6%)	
Amputation	42	Self-reported physical activity, n(%)	<1 times/week	1122 (13.2%)	15.7	857 (13.1%)	
Amputation	42	Self-reported physical activity, n(%)	regularly - 1-2 times/week	1762 (20.8%)	15.7	1396 (21.3%)	
Amputation	42	Self-reported physical activity, n(%)	regularly - 3-5 times/week	2059 (24.3%)	15.7	1597 (24.4%)	
Amputation	42	Self-reported physical activity, n(%)	Daily	2244 (26.5%)	15.7	1740 (26.6%)	
Amputation	42	Sex, n(%)	Males	6001 (59.7%)	0	3994 (61%)	n=10060 vs n=6548
Amputation	42	Sex, n(%)	Females	4058 (40.3%)	0	2554 (39%)	
Amputation	42	Smoking status, n(%)	No	7102 (78.4%)	10	5206 (79.5%)	
Amputation	42	Smoking status, n(%)	Yes	1956 (21.6%)	10	1342 (20.5%)	
Amputation	42	Stroke, n(%)	No	8822 (98.7%)	11.2	6466 (98.7%)	
Amputation	42	Stroke, n(%)	Yes	112 (1.3%)	11.2	82 (1.3%)	
Amputation	42	Systolic blood pressure, mean(SD)		128.1 (14.7)	5.1	127.9 (14.4)	
Amputation	42	Time since last healthcare visit, mean(SD)		0.8 (1.3)	0	0.7 (1)	
Amputation	42	Total cholesterol, mean(SD)		4.9 (1.2)	13.1	4.8 (1.2)	
Amputation	43	Age at diagnosis of type 2 diabetes, mean(SD)		39.4 (2.7)	0	38.9 (2.7)	
Amputation	43	BMI, mean(SD)		33.1 (6.6)	9.1	33 (6.6)	
Amputation	43	HbA1c, mean(SD)		52.6 (16.3)	3.1	52.8 (16)	

Amputation	43	HDL-cholesterol, mean(SD)		1.1 (0.3)	17.4	1.1 (0.3)	
Amputation	43	Ischemic heart disease, n(%)	No	9879 (97.4%)	10.7	7297 (97.5%)	
Amputation	43	Ischemic heart disease, n(%)	Yes	259 (2.6%)	10.7	188 (2.5%)	
Amputation	43	Neuropathy, n(%)	No	10605 (93.5%)	0	6848 (91.5%)	
Amputation	43	Neuropathy, n(%)	Yes	743 (6.5%)	0	637 (8.5%)	
Amputation	43	Self-reported physical activity, n(%)	Never	1429 (14.9%)	15.7	1099 (14.7%)	
Amputation	43	Self-reported physical activity, n(%)	<1 times/week	1296 (13.5%)	15.7	994 (13.3%)	
Amputation	43	Self-reported physical activity, n(%)	regularly - 1-2 times/week	1980 (20.7%)	15.7	1563 (20.9%)	
Amputation	43	Self-reported physical activity, n(%)	regularly - 3-5 times/week	2284 (23.9%)	15.7	1790 (23.9%)	
Amputation	43	Self-reported physical activity, n(%)	Daily	2582 (27%)	15.7	2039 (27.2%)	
Amputation	43	Sex, n(%)	Males	6869 (60.5%)	0	4641 (62%)	n=11348 vs n=7485
Amputation	43	Sex, n(%)	Females	4476 (39.5%)	0	2844 (38%)	
Amputation	43	Smoking status, n(%)	No	8061 (78.4%)	9.4	5935 (79.3%)	
Amputation	43	Smoking status, n(%)	Yes	2225 (21.6%)	9.4	1550 (20.7%)	
Amputation	43	Stroke, n(%)	No	9977 (98.7%)	10.9	7383 (98.6%)	
Amputation	43	Stroke, n(%)	Yes	133 (1.3%)	10.9	102 (1.4%)	
Amputation	43	Systolic blood pressure, mean(SD)		128.7 (14.8)	4.7	128.5 (14.5)	
Amputation	43	Time since last healthcare visit, mean(SD)		0.8 (1.3)	0	0.7 (1)	
Amputation	43	Total cholesterol, mean(SD)		4.9 (1.2)	13.1	4.8 (1.2)	
Amputation	44	Age at diagnosis of type 2 diabetes, mean(SD)		40.4 (2.7)	0	39.9 (2.7)	
Amputation	44	BMI, mean(SD)		33 (6.6)	9	32.9 (6.5)	
Amputation	44	HbA1c, mean(SD)		52.7 (16.4)	3	52.8 (16)	
Amputation	44	HDL-cholesterol, mean(SD)		1.1 (0.3)	17.4	1.1 (0.3)	

Amputation	44	Ischemic heart disease, n(%)	No	11158 (96.8%)	10.9	8236 (96.9%)	
Amputation	44	Ischemic heart disease, n(%)	Yes	363 (3.2%)	10.9	265 (3.1%)	
Amputation	44	Neuropathy, n(%)	No	12018 (93%)	0	7731 (90.9%)	
Amputation	44	Neuropathy, n(%)	Yes	909 (7%)	0	770 (9.1%)	
Amputation	44	Self-reported physical activity, n(%)	Never	1602 (14.7%)	15.6	1220 (14.4%)	
Amputation	44	Self-reported physical activity, n(%)	<1 times/week	1504 (13.8%)	15.6	1149 (13.5%)	
Amputation	44	Self-reported physical activity, n(%)	regularly - 1-2 times/week	2241 (20.5%)	15.6	1752 (20.6%)	
Amputation	44	Self-reported physical activity, n(%)	regularly - 3-5 times/week	2686 (24.6%)	15.6	2107 (24.8%)	
Amputation	44	Self-reported physical activity, n(%)	Daily	2877 (26.4%)	15.6	2273 (26.7%)	
Amputation	44	Sex, n(%)	Males	7873 (60.9%)	0	5302 (62.4%)	n=12927 vs n=8501
Amputation	44	Sex, n(%)	Females	5052 (39.1%)	0	3199 (37.6%)	
Amputation	44	Smoking status, n(%)	No	9179 (78.4%)	9.4	6739 (79.3%)	
Amputation	44	Smoking status, n(%)	Yes	2527 (21.6%)	9.4	1762 (20.7%)	
Amputation	44	Stroke, n(%)	No	11332 (98.6%)	11.1	8382 (98.6%)	
Amputation	44	Stroke, n(%)	Yes	160 (1.4%)	11.1	119 (1.4%)	
Amputation	44	Systolic blood pressure, mean(SD)		128.8 (14.6)	4.7	128.5 (14.4)	
Amputation	44	Time since last healthcare visit, mean(SD)		0.8 (1.3)	0	0.7 (1)	
Amputation	44	Total cholesterol, mean(SD)		4.9 (1.2)	13.2	4.8 (1.2)	
Amputation	45	Age at diagnosis of type 2 diabetes, mean(SD)		41.4 (2.7)	0	40.9 (2.7)	
Amputation	45	BMI, mean(SD)		33 (6.5)	9.3	32.9 (6.5)	
Amputation	45	HbA1c, mean(SD)		52.5 (16)	2.9	52.5 (15.6)	
Amputation	45	HDL-cholesterol, mean(SD)		1.1 (0.3)	16.6	1.1 (0.3)	
Amputation	45	Ischemic heart disease, n(%)	No	12657 (96.3%)	10.6	9364 (96.4%)	

Amputation	45	Ischemic heart disease, n(%)	Yes	491 (3.7%)	10.6	350 (3.6%)	
Amputation	45	Neuropathy, n(%)	No	13618 (92.6%)	0	8791 (90.5%)	
Amputation	45	Neuropathy, n(%)	Yes	1092 (7.4%)	0	923 (9.5%)	
Amputation	45	Self-reported physical activity, n(%)	Never	1820 (14.6%)	15.4	1400 (14.4%)	
Amputation	45	Self-reported physical activity, n(%)	<1 times/week	1672 (13.4%)	15.4	1268 (13.1%)	
Amputation	45	Self-reported physical activity, n(%)	regularly - 1-2 times/week	2610 (21%)	15.4	2046 (21.1%)	
Amputation	45	Self-reported physical activity, n(%)	regularly - 3-5 times/week	3069 (24.7%)	15.4	2427 (25%)	
Amputation	45	Self-reported physical activity, n(%)	Daily	3269 (26.3%)	15.4	2573 (26.5%)	
Amputation	45	Sex, n(%)	Males	9112 (62%)	0	6074 (62.5%)	n=14710 vs n=9714
Amputation	45	Sex, n(%)	Females	5596 (38%)	0	3640 (37.5%)	
Amputation	45	Smoking status, n(%)	No	10448 (78.4%)	9.4	7712 (79.4%)	
Amputation	45	Smoking status, n(%)	Yes	2883 (21.6%)	9.4	2002 (20.6%)	
Amputation	45	Stroke, n(%)	No	12908 (98.5%)	10.9	9574 (98.6%)	
Amputation	45	Stroke, n(%)	Yes	195 (1.5%)	10.9	140 (1.4%)	
Amputation	45	Systolic blood pressure, mean(SD)		129.4 (14.8)	4.4	129.1 (14.5)	
Amputation	45	Time since last healthcare visit, mean(SD)		0.8 (1.3)	0	0.7 (1)	
Amputation	45	Total cholesterol, mean(SD)		4.9 (1.2)	12.6	4.8 (1.1)	
Amputation	46	Age at diagnosis of type 2 diabetes, mean(SD)		42.4 (2.7)	0	41.9 (2.7)	
Amputation	46	BMI, mean(SD)		32.9 (6.4)	9.1	32.8 (6.3)	
Amputation	46	HbA1c, mean(SD)		52.6 (16)	2.8	52.7 (15.6)	
Amputation	46	HDL-cholesterol, mean(SD)		1.1 (0.3)	16.2	1.1 (0.3)	
Amputation	46	Ischemic heart disease, n(%)	No	14235 (95.7%)	10.3	10579 (95.8%)	
Amputation	46	Ischemic heart disease, n(%)	Yes	634 (4.3%)	10.3	465 (4.2%)	

Amputation	46	Neuropathy, n(%)	No	15304 (92.3%)	0	9954 (90.1%)	
Amputation	46	Neuropathy, n(%)	Yes	1281 (7.7%)	0	1090 (9.9%)	
Amputation	46	Self-reported physical activity, n(%)	Never	2075 (14.7%)	15	1609 (14.6%)	
Amputation	46	Self-reported physical activity, n(%)	<1 times/week	1943 (13.8%)	15	1489 (13.5%)	
Amputation	46	Self-reported physical activity, n(%)	regularly - 1-2 times/week	2991 (21.2%)	15	2360 (21.4%)	
Amputation	46	Self-reported physical activity, n(%)	regularly - 3-5 times/week	3457 (24.5%)	15	2740 (24.8%)	
Amputation	46	Self-reported physical activity, n(%)	Daily	3625 (25.7%)	15	2846 (25.8%)	
Amputation	46	Sex, n(%)	Males	10374 (62.6%)	0	6980 (63.2%)	n=16585 vs n=11044
Amputation	46	Sex, n(%)	Females	6210 (37.4%)	0	4064 (36.8%)	
Amputation	46	Smoking status, n(%)	No	11800 (78.2%)	9.1	8762 (79.3%)	
Amputation	46	Smoking status, n(%)	Yes	3282 (21.8%)	9.1	2282 (20.7%)	
Amputation	46	Stroke, n(%)	No	14547 (98.2%)	10.7	10846 (98.2%)	
Amputation	46	Stroke, n(%)	Yes	265 (1.8%)	10.7	198 (1.8%)	
Amputation	46	Systolic blood pressure, mean(SD)		129.7 (14.8)	4.2	129.4 (14.5)	
Amputation	46	Time since last healthcare visit, mean(SD)		0.8 (1.2)	0	0.6 (1)	
Amputation	46	Total cholesterol, mean(SD)		4.9 (1.2)	11.8	4.8 (1.1)	
Amputation	47	Age at diagnosis of type 2 diabetes, mean(SD)		43.3 (2.7)	0	42.8 (2.7)	
Amputation	47	BMI, mean(SD)		32.8 (6.3)	8.9	32.7 (6.3)	
Amputation	47	HbA1c, mean(SD)		52.5 (15.7)	2.5	52.6 (15.3)	
Amputation	47	HDL-cholesterol, mean(SD)		1.1 (0.3)	15.7	1.1 (0.3)	
Amputation	47	Ischemic heart disease, n(%)	No	15924 (95.2%)	10	11954 (95.5%)	
Amputation	47	Ischemic heart disease, n(%)	Yes	798 (4.8%)	10	560 (4.5%)	
Amputation	47	Neuropathy, n(%)	No	17104 (92.1%)	0	11262 (90%)	

Amputation	47	Neuropathy, n(%)	Yes	1472 (7.9%)	0	1252 (10%)	
Amputation	47	Self-reported physical activity, n(%)	Never	2298 (14.5%)	14.7	1807 (14.4%)	
Amputation	47	Self-reported physical activity, n(%)	<1 times/week	2189 (13.8%)	14.7	1679 (13.4%)	
Amputation	47	Self-reported physical activity, n(%)	regularly - 1-2 times/week	3338 (21.1%)	14.7	2637 (21.1%)	
Amputation	47	Self-reported physical activity, n(%)	regularly - 3-5 times/week	3873 (24.5%)	14.7	3094 (24.7%)	
Amputation	47	Self-reported physical activity, n(%)	Daily	4139 (26.1%)	14.7	3297 (26.3%)	
Amputation	47	Sex, n(%)	Males	11696 (63%)	0	7987 (63.8%)	n=18576 vs n=12514
Amputation	47	Sex, n(%)	Females	6878 (37%)	0	4527 (36.2%)	
Amputation	47	Smoking status, n(%)	No	13237 (78.1%)	8.8	9901 (79.1%)	
Amputation	47	Smoking status, n(%)	Yes	3707 (21.9%)	8.8	2613 (20.9%)	
Amputation	47	Stroke, n(%)	No	16340 (98%)	10.3	12275 (98.1%)	
Amputation	47	Stroke, n(%)	Yes	329 (2%)	10.3	239 (1.9%)	
Amputation	47	Systolic blood pressure, mean(SD)		129.9 (14.7)	3.9	129.5 (14.5)	
Amputation	47	Time since last healthcare visit, mean(SD)		0.8 (1.2)	0	0.7 (1)	
Amputation	47	Total cholesterol, mean(SD)		4.9 (1.1)	11.3	4.8 (1.1)	
Amputation	48	Age at diagnosis of type 2 diabetes, mean(SD)		44.2 (2.7)	0	43.7 (2.7)	
Amputation	48	BMI, mean(SD)		32.7 (6.3)	8.6	32.6 (6.2)	
Amputation	48	HbA1c, mean(SD)		52.5 (15.6)	2.5	52.6 (15.2)	
Amputation	48	HDL-cholesterol, mean(SD)		1.1 (0.3)	15.5	1.1 (0.3)	
Amputation	48	Ischemic heart disease, n(%)	No	17517 (95%)	9.9	13251 (95.3%)	
Amputation	48	Ischemic heart disease, n(%)	Yes	926 (5%)	9.9	659 (4.7%)	
Amputation	48	Neuropathy, n(%)	No	18734 (91.5%)	0	12464 (89.6%)	
Amputation	48	Neuropathy, n(%)	Yes	1731 (8.5%)	0	1446 (10.4%)	

Amputation	48	Self-reported physical activity, n(%)	Never	2548 (14.5%)	14.2	2033 (14.6%)	
Amputation	48	Self-reported physical activity, n(%)	<1 times/week	2402 (13.7%)	14.2	1819 (13.1%)	
Amputation	48	Self-reported physical activity, n(%)	regularly - 1-2 times/week	3716 (21.2%)	14.2	2944 (21.2%)	
Amputation	48	Self-reported physical activity, n(%)	regularly - 3-5 times/week	4307 (24.5%)	14.2	3458 (24.9%)	
Amputation	48	Self-reported physical activity, n(%)	Daily	4580 (26.1%)	14.2	3656 (26.3%)	
Amputation	48	Sex, n(%)	Males	12846 (62.8%)	0	8869 (63.8%)	n=20465 vs n=13910
Amputation	48	Sex, n(%)	Females	7618 (37.2%)	0	5041 (36.2%)	
Amputation	48	Smoking status, n(%)	No	14715 (78.4%)	8.3	11051 (79.4%)	
Amputation	48	Smoking status, n(%)	Yes	4046 (21.6%)	8.3	2859 (20.6%)	
Amputation	48	Stroke, n(%)	No	18016 (98%)	10.1	13636 (98%)	
Amputation	48	Stroke, n(%)	Yes	376 (2%)	10.1	274 (2%)	
Amputation	48	Systolic blood pressure, mean(SD)		130.4 (14.7)	3.8	130.2 (14.3)	
Amputation	48	Time since last healthcare visit, mean(SD)		0.8 (1.2)	0	0.6 (1)	
Amputation	48	Total cholesterol, mean(SD)		4.9 (1.1)	11.3	4.8 (1.1)	
Amputation	49	Age at diagnosis of type 2 diabetes, mean(SD)		45.2 (2.7)	0	44.7 (2.7)	
Amputation	49	BMI, mean(SD)		32.5 (6.1)	8.7	32.4 (6)	
Amputation	49	HbA1c, mean(SD)		52.5 (15.5)	2.4	52.5 (15)	
Amputation	49	HDL-cholesterol, mean(SD)		1.1 (0.3)	15.5	1.1 (0.3)	
Amputation	49	Ischemic heart disease, n(%)	No	19448 (94.5%)	9.5	14760 (94.8%)	
Amputation	49	Ischemic heart disease, n(%)	Yes	1140 (5.5%)	9.5	817 (5.2%)	
Amputation	49	Neuropathy, n(%)	No	20748 (91.2%)	0	13897 (89.2%)	
Amputation	49	Neuropathy, n(%)	Yes	2004 (8.8%)	0	1680 (10.8%)	
Amputation	49	Self-reported physical activity, n(%)	Never	2880 (14.7%)	13.6	2307 (14.8%)	

Amputation	49	Self-reported physical activity, n(%)	<1 times/week	2647 (13.5%)	13.6	2014 (12.9%)	
Amputation	49	Self-reported physical activity, n(%)	regularly - 1-2 times/week	4122 (21%)	13.6	3266 (21%)	
Amputation	49	Self-reported physical activity, n(%)	regularly - 3-5 times/week	4694 (23.9%)	13.6	3752 (24.1%)	
Amputation	49	Self-reported physical activity, n(%)	Daily	5312 (27%)	13.6	4238 (27.2%)	
Amputation	49	Sex, n(%)	Males	14301 (62.9%)	0	9957 (63.9%)	n=22752 vs n=15577
Amputation	49	Sex, n(%)	Females	8447 (37.1%)	0	5620 (36.1%)	
Amputation	49	Smoking status, n(%)	No	16268 (78%)	8.3	12320 (79.1%)	
Amputation	49	Smoking status, n(%)	Yes	4597 (22%)	8.3	3257 (20.9%)	
Amputation	49	Stroke, n(%)	No	20074 (97.9%)	9.9	15275 (98.1%)	
Amputation	49	Stroke, n(%)	Yes	432 (2.1%)	9.9	302 (1.9%)	
Amputation	49	Systolic blood pressure, mean(SD)		130.9 (14.8)	3.7	130.5 (14.5)	
Amputation	49	Time since last healthcare visit, mean(SD)		0.8 (1.2)	0	0.6 (1)	
Amputation	49	Total cholesterol, mean(SD)		4.9 (1.2)	11.2	4.8 (1.1)	
Amputation	50	Age at diagnosis of type 2 diabetes, mean(SD)		46.2 (2.8)	0	45.7 (2.8)	
Amputation	50	BMI, mean(SD)		32.4 (6.1)	8.7	32.3 (6)	
Amputation	50	HbA1c, mean(SD)		52.4 (15.2)	2.3	52.5 (14.8)	
Amputation	50	HDL-cholesterol, mean(SD)		1.1 (0.3)	15.1	1.1 (0.3)	
Amputation	50	Ischemic heart disease, n(%)	No	21517 (93.9%)	9.5	16376 (94.2%)	
Amputation	50	Ischemic heart disease, n(%)	Yes	1387 (6.1%)	9.5	1006 (5.8%)	
Amputation	50	Neuropathy, n(%)	No	23000 (90.8%)	0	15428 (88.8%)	
Amputation	50	Neuropathy, n(%)	Yes	2322 (9.2%)	0	1954 (11.2%)	
Amputation	50	Self-reported physical activity, n(%)	Never	3206 (14.7%)	13.7	2574 (14.8%)	
Amputation	50	Self-reported physical activity, n(%)	<1 times/week	2973 (13.6%)	13.7	2291 (13.2%)	

Amputation	50	Self-reported physical activity, n(%)	regularly - 1-2 times/week	4486 (20.5%)	13.7	3574 (20.6%)	
Amputation	50	Self-reported physical activity, n(%)	regularly - 3-5 times/week	5283 (24.2%)	13.7	4224 (24.3%)	
Amputation	50	Self-reported physical activity, n(%)	Daily	5915 (27.1%)	13.7	4719 (27.1%)	
Amputation	50	Sex, n(%)	Males	15954 (63%)	0	11132 (64%)	n=25322 vs n=17382
Amputation	50	Sex, n(%)	Females	9365 (37%)	0	6250 (36%)	
Amputation	50	Smoking status, n(%)	No	18116 (78%)	8.2	13717 (78.9%)	
Amputation	50	Smoking status, n(%)	Yes	5123 (22%)	8.2	3665 (21.1%)	
Amputation	50	Stroke, n(%)	No	22326 (97.8%)	9.8	17017 (97.9%)	
Amputation	50	Stroke, n(%)	Yes	511 (2.2%)	9.8	365 (2.1%)	
Amputation	50	Systolic blood pressure, mean(SD)		131.3 (14.9)	3.7	131 (14.6)	
Amputation	50	Time since last healthcare visit, mean(SD)		0.8 (1.2)	0	0.6 (1)	
Amputation	50	Total cholesterol, mean(SD)		4.9 (1.2)	11	4.8 (1.1)	
Amputation	51	Age at diagnosis of type 2 diabetes, mean(SD)		47.2 (2.8)	0	46.6 (2.8)	
Amputation	51	BMI, mean(SD)		32.3 (6)	8.3	32.2 (5.9)	
Amputation	51	HbA1c, mean(SD)		52.4 (14.9)	2.1	52.4 (14.5)	
Amputation	51	HDL-cholesterol, mean(SD)		1.1 (0.3)	14.3	1.1 (0.3)	
Amputation	51	Ischemic heart disease, n(%)	No	23645 (93.4%)	9.2	18161 (93.7%)	
Amputation	51	Ischemic heart disease, n(%)	Yes	1679 (6.6%)	9.2	1228 (6.3%)	
Amputation	51	Neuropathy, n(%)	No	25113 (90.1%)	0	17051 (87.9%)	
Amputation	51	Neuropathy, n(%)	Yes	2764 (9.9%)	0	2338 (12.1%)	
Amputation	51	Self-reported physical activity, n(%)	Never	3509 (14.5%)	13.2	2817 (14.5%)	
Amputation	51	Self-reported physical activity, n(%)	<1 times/week	3300 (13.6%)	13.2	2588 (13.3%)	
Amputation	51	Self-reported physical activity, n(%)	regularly - 1-2 times/week	4943 (20.4%)	13.2	3960 (20.4%)	

Amputation	51	Self-reported physical activity, n(%)	regularly - 3-5 times/week	5964 (24.7%)	13.2	4842 (25%)	
Amputation	51	Self-reported physical activity, n(%)	Daily	6470 (26.8%)	13.2	5182 (26.7%)	
Amputation	51	Sex, n(%)	Males	17542 (62.9%)	0	12377 (63.8%)	n=27877 vs n=19389
Amputation	51	Sex, n(%)	Females	10327 (37.1%)	0	7012 (36.2%)	
Amputation	51	Smoking status, n(%)	No	19973 (77.8%)	7.9	15267 (78.7%)	
Amputation	51	Smoking status, n(%)	Yes	5695 (22.2%)	7.9	4122 (21.3%)	
Amputation	51	Stroke, n(%)	No	24647 (97.6%)	9.4	18948 (97.7%)	
Amputation	51	Stroke, n(%)	Yes	605 (2.4%)	9.4	441 (2.3%)	
Amputation	51	Systolic blood pressure, mean(SD)		131.7 (15)	3.4	131.4 (14.7)	
Amputation	51	Time since last healthcare visit, mean(SD)		0.7 (1.2)	0	0.6 (0.9)	
Amputation	51	Total cholesterol, mean(SD)		4.9 (1.2)	10.3	4.8 (1.1)	
Amputation	52	Age at diagnosis of type 2 diabetes, mean(SD)		48.1 (2.8)	0	47.6 (2.8)	
Amputation	52	BMI, mean(SD)		32.2 (5.9)	8.2	32.1 (5.8)	
Amputation	52	HbA1c, mean(SD)		52.5 (14.9)	2.1	52.5 (14.4)	
Amputation	52	HDL-cholesterol, mean(SD)		1.1 (0.3)	13.9	1.2 (0.3)	
Amputation	52	Ischemic heart disease, n(%)	No	25450 (93.1%)	9	19638 (93.3%)	
Amputation	52	Ischemic heart disease, n(%)	Yes	1899 (6.9%)	9	1399 (6.7%)	
Amputation	52	Neuropathy, n(%)	No	26930 (89.6%)	0	18355 (87.3%)	
Amputation	52	Neuropathy, n(%)	Yes	3137 (10.4%)	0	2682 (12.7%)	
Amputation	52	Self-reported physical activity, n(%)	Never	3794 (14.5%)	12.9	3037 (14.4%)	
Amputation	52	Self-reported physical activity, n(%)	<1 times/week	3529 (13.5%)	12.9	2760 (13.1%)	
Amputation	52	Self-reported physical activity, n(%)	regularly - 1-2 times/week	5345 (20.4%)	12.9	4244 (20.2%)	
Amputation	52	Self-reported physical activity, n(%)	regularly - 3-5 times/week	6326 (24.2%)	12.9	5157 (24.5%)	

Amputation	52	Self-reported physical activity, n(%)	Daily	7196 (27.5%)	12.9	5839 (27.8%)	
Amputation	52	Sex, n(%)	Males	18875 (62.8%)	0	13338 (63.4%)	n=30067 vs n=21037
Amputation	52	Sex, n(%)	Females	11186 (37.2%)	0	7699 (36.6%)	
Amputation	52	Smoking status, n(%)	No	21507 (77.5%)	7.8	16504 (78.5%)	
Amputation	52	Smoking status, n(%)	Yes	6228 (22.5%)	7.8	4533 (21.5%)	
Amputation	52	Stroke, n(%)	No	26533 (97.4%)	9.4	20522 (97.6%)	
Amputation	52	Stroke, n(%)	Yes	708 (2.6%)	9.4	515 (2.4%)	
Amputation	52	Systolic blood pressure, mean(SD)		132 (15)	3.2	131.8 (14.7)	
Amputation	52	Time since last healthcare visit, mean(SD)		0.7 (1.2)	0	0.6 (0.9)	
Amputation	52	Total cholesterol, mean(SD)		4.9 (1.2)	10	4.8 (1.1)	
Amputation	53	Age at diagnosis of type 2 diabetes, mean(SD)		49 (2.8)	0	48.5 (2.8)	
Amputation	53	BMI, mean(SD)		32 (5.9)	8.3	31.9 (5.8)	
Amputation	53	HbA1c, mean(SD)		52.3 (14.7)	2.3	52.4 (14.4)	
Amputation	53	HDL-cholesterol, mean(SD)		1.2 (0.3)	14.2	1.2 (0.3)	
Amputation	53	Ischemic heart disease, n(%)	No	26973 (92.5%)	9.1	20860 (92.8%)	
Amputation	53	Ischemic heart disease, n(%)	Yes	2190 (7.5%)	9.1	1624 (7.2%)	
Amputation	53	Neuropathy, n(%)	No	28511 (88.9%)	0	19437 (86.4%)	
Amputation	53	Neuropathy, n(%)	Yes	3556 (11.1%)	0	3047 (13.6%)	
Amputation	53	Self-reported physical activity, n(%)	Never	3988 (14.3%)	12.9	3184 (14.2%)	
Amputation	53	Self-reported physical activity, n(%)	<1 times/week	3714 (13.3%)	12.9	2949 (13.1%)	
Amputation	53	Self-reported physical activity, n(%)	regularly - 1-2 times/week	5731 (20.5%)	12.9	4594 (20.4%)	
Amputation	53	Self-reported physical activity, n(%)	regularly - 3-5 times/week	6751 (24.2%)	12.9	5517 (24.5%)	
Amputation	53	Self-reported physical activity, n(%)	Daily	7754 (27.8%)	12.9	6240 (27.8%)	

Amputation	53	Sex, n(%)	Males	20048 (62.5%)	0	14213 (63.2%)	n=32067 vs n=22484
Amputation	53	Sex, n(%)	Females	12014 (37.5%)	0	8271 (36.8%)	
Amputation	53	Smoking status, n(%)	No	22860 (77.4%)	7.9	17612 (78.3%)	
Amputation	53	Smoking status, n(%)	Yes	6663 (22.6%)	7.9	4872 (21.7%)	
Amputation	53	Stroke, n(%)	No	28236 (97.2%)	9.4	21872 (97.3%)	
Amputation	53	Stroke, n(%)	Yes	828 (2.8%)	9.4	612 (2.7%)	
Amputation	53	Systolic blood pressure, mean(SD)		132.2 (14.9)	3.3	131.9 (14.6)	
Amputation	53	Time since last healthcare visit, mean(SD)		0.7 (1.2)	0	0.6 (0.9)	
Amputation	53	Total cholesterol, mean(SD)		4.9 (1.2)	10.3	4.8 (1.2)	
Amputation	54	Age at diagnosis of type 2 diabetes, mean(SD)		50 (2.8)	0	49.5 (2.8)	
Amputation	54	BMI, mean(SD)		31.9 (5.8)	8.1	31.8 (5.7)	
Amputation	54	HbA1c, mean(SD)		52.4 (14.6)	2.3	52.4 (14.2)	
Amputation	54	HDL-cholesterol, mean(SD)		1.2 (0.3)	14.1	1.2 (0.3)	
Amputation	54	Ischemic heart disease, n(%)	No	28491 (91.8%)	8.8	22076 (92.1%)	
Amputation	54	Ischemic heart disease, n(%)	Yes	2535 (8.2%)	8.8	1905 (7.9%)	
Amputation	54	Neuropathy, n(%)	No	30078 (88.4%)	0	20639 (86.1%)	
Amputation	54	Neuropathy, n(%)	Yes	3933 (11.6%)	0	3342 (13.9%)	
Amputation	54	Self-reported physical activity, n(%)	Never	4115 (13.9%)	12.8	3282 (13.7%)	
Amputation	54	Self-reported physical activity, n(%)	<1 times/week	3968 (13.4%)	12.8	3133 (13.1%)	
Amputation	54	Self-reported physical activity, n(%)	regularly - 1-2 times/week	6030 (20.3%)	12.8	4866 (20.3%)	
Amputation	54	Self-reported physical activity, n(%)	regularly - 3-5 times/week	7213 (24.3%)	12.8	5905 (24.6%)	
Amputation	54	Self-reported physical activity, n(%)	Daily	8337 (28.1%)	12.8	6795 (28.3%)	
Amputation	54	Sex, n(%)	Males	21188 (62.3%)	0	15078 (62.9%)	n=34011 vs n=23981

Amputation	54	Sex, n(%)	Females	12817 (37.7%)	0	8903 (37.1%)	
Amputation	54	Smoking status, n(%)	No	24287 (77.3%)	7.6	18744 (78.2%)	
Amputation	54	Smoking status, n(%)	Yes	7124 (22.7%)	7.6	5237 (21.8%)	
Amputation	54	Stroke, n(%)	No	29978 (97%)	9.1	23280 (97.1%)	
Amputation	54	Stroke, n(%)	Yes	932 (3%)	9.1	701 (2.9%)	
Amputation	54	Systolic blood pressure, mean(SD)		132.4 (14.9)	3.2	132.1 (14.7)	
Amputation	54	Time since last healthcare visit, mean(SD)		0.7 (1.2)	0	0.6 (0.9)	
Amputation	54	Total cholesterol, mean(SD)		4.9 (1.2)	10.1	4.8 (1.2)	
Amputation	55	Age at diagnosis of type 2 diabetes, mean(SD)		51 (2.8)	0	50.5 (2.8)	
Amputation	55	BMI, mean(SD)		31.7 (5.7)	8.2	31.6 (5.6)	
Amputation	55	HbA1c, mean(SD)		52.1 (14.4)	2.3	52.2 (14)	
Amputation	55	HDL-cholesterol, mean(SD)		1.2 (0.3)	14	1.2 (0.3)	
Amputation	55	Ischemic heart disease, n(%)	No	29875 (91.1%)	8.9	23171 (91.4%)	
Amputation	55	Ischemic heart disease, n(%)	Yes	2913 (8.9%)	8.9	2168 (8.6%)	
Amputation	55	Neuropathy, n(%)	No	31669 (88%)	0	21648 (85.4%)	
Amputation	55	Neuropathy, n(%)	Yes	4304 (12%)	0	3691 (14.6%)	
Amputation	55	Self-reported physical activity, n(%)	Never	4300 (13.7%)	12.7	3440 (13.6%)	
Amputation	55	Self-reported physical activity, n(%)	<1 times/week	4196 (13.4%)	12.7	3321 (13.1%)	
Amputation	55	Self-reported physical activity, n(%)	regularly - 1-2 times/week	6477 (20.6%)	12.7	5179 (20.4%)	
Amputation	55	Self-reported physical activity, n(%)	regularly - 3-5 times/week	7600 (24.2%)	12.7	6170 (24.3%)	
Amputation	55	Self-reported physical activity, n(%)	Daily	8836 (28.1%)	12.7	7229 (28.5%)	
Amputation	55	Sex, n(%)	Males	22376 (62.2%)	0	15849 (62.5%)	n=35973 vs n=25339
Amputation	55	Sex, n(%)	Females	13593 (37.8%)	0	9490 (37.5%)	

Amputation	55	Smoking status, n(%)	No	25721 (77.3%)	7.5	19784 (78.1%)	
Amputation	55	Smoking status, n(%)	Yes	7571 (22.7%)	7.5	5555 (21.9%)	
Amputation	55	Stroke, n(%)	No	31590 (96.7%)	9.2	24521 (96.8%)	
Amputation	55	Stroke, n(%)	Yes	1067 (3.3%)	9.2	818 (3.2%)	
Amputation	55	Systolic blood pressure, mean(SD)		132.8 (14.9)	3.3	132.4 (14.7)	
Amputation	55	Time since last healthcare visit, mean(SD)		0.7 (1.2)	0	0.6 (0.9)	
Amputation	55	Total cholesterol, mean(SD)		4.9 (1.2)	10	4.8 (1.2)	
Amputation	56	Age at diagnosis of type 2 diabetes, mean(SD)		52 (2.8)	0	51.5 (2.8)	
Amputation	56	BMI, mean(SD)		31.5 (5.7)	8.2	31.5 (5.6)	
Amputation	56	HbA1c, mean(SD)		51.8 (13.9)	2.1	51.9 (13.4)	
Amputation	56	HDL-cholesterol, mean(SD)		1.2 (0.4)	13.9	1.2 (0.3)	
Amputation	56	Ischemic heart disease, n(%)	No	31032 (90.3%)	8.9	24179 (90.5%)	
Amputation	56	Ischemic heart disease, n(%)	Yes	3350 (9.7%)	8.9	2524 (9.5%)	
Amputation	56	Neuropathy, n(%)	No	33154 (87.8%)	0	22786 (85.3%)	
Amputation	56	Neuropathy, n(%)	Yes	4596 (12.2%)	0	3917 (14.7%)	
Amputation	56	Self-reported physical activity, n(%)	Never	4548 (13.8%)	12.5	3653 (13.7%)	
Amputation	56	Self-reported physical activity, n(%)	<1 times/week	4338 (13.1%)	12.5	3402 (12.7%)	
Amputation	56	Self-reported physical activity, n(%)	regularly - 1-2 times/week	6889 (20.9%)	12.5	5563 (20.8%)	
Amputation	56	Self-reported physical activity, n(%)	regularly - 3-5 times/week	8035 (24.3%)	12.5	6578 (24.6%)	
Amputation	56	Self-reported physical activity, n(%)	Daily	9227 (27.9%)	12.5	7507 (28.1%)	
Amputation	56	Sex, n(%)	Males	23449 (62.1%)	0	16691 (62.5%)	n=37750 vs n=26703
Amputation	56	Sex, n(%)	Females	14292 (37.9%)	0	10012 (37.5%)	
Amputation	56	Smoking status, n(%)	No	26967 (77.2%)	7.5	20822 (78%)	

Amputation	56	Smoking status, n(%)	Yes	7955 (22.8%)	7.5	5881 (22%)	
Amputation	56	Stroke, n(%)	No	33015 (96.5%)	9.3	25778 (96.5%)	
Amputation	56	Stroke, n(%)	Yes	1211 (3.5%)	9.3	925 (3.5%)	
Amputation	56	Systolic blood pressure, mean(SD)		132.9 (14.8)	3.1	132.6 (14.6)	
Amputation	56	Time since last healthcare visit, mean(SD)		0.7 (1.1)	0	0.6 (0.9)	
Amputation	56	Total cholesterol, mean(SD)		4.9 (1.2)	9.9	4.8 (1.2)	
Amputation	57	Age at diagnosis of type 2 diabetes, mean(SD)		53 (2.8)	0	52.4 (2.8)	
Amputation	57	BMI, mean(SD)		31.4 (5.6)	8.1	31.4 (5.5)	
Amputation	57	HbA1c, mean(SD)		51.7 (13.7)	2.1	51.7 (13.3)	
Amputation	57	HDL-cholesterol, mean(SD)		1.2 (0.4)	13.6	1.2 (0.4)	
Amputation	57	Ischemic heart disease, n(%)	No	32305 (89.4%)	8.8	25277 (89.7%)	
Amputation	57	Ischemic heart disease, n(%)	Yes	3821 (10.6%)	8.8	2887 (10.3%)	
Amputation	57	Neuropathy, n(%)	No	34602 (87.3%)	0	23894 (84.8%)	
Amputation	57	Neuropathy, n(%)	Yes	5012 (12.7%)	0	4270 (15.2%)	
Amputation	57	Self-reported physical activity, n(%)	Never	4660 (13.4%)	12.4	3756 (13.3%)	
Amputation	57	Self-reported physical activity, n(%)	<1 times/week	4550 (13.1%)	12.4	3627 (12.9%)	
Amputation	57	Self-reported physical activity, n(%)	regularly - 1-2 times/week	7203 (20.8%)	12.4	5894 (20.9%)	
Amputation	57	Self-reported physical activity, n(%)	regularly - 3-5 times/week	8377 (24.1%)	12.4	6826 (24.2%)	
Amputation	57	Self-reported physical activity, n(%)	Daily	9920 (28.6%)	12.4	8061 (28.6%)	
Amputation	57	Sex, n(%)	Males	24619 (62.2%)	0	17544 (62.3%)	n=39614 vs n=28164
Amputation	57	Sex, n(%)	Females	14987 (37.8%)	0	10620 (37.7%)	
Amputation	57	Smoking status, n(%)	No	28624 (77.9%)	7.2	22126 (78.6%)	
Amputation	57	Smoking status, n(%)	Yes	8126 (22.1%)	7.2	6038 (21.4%)	

Amputation	57	Stroke, n(%)	No	34620 (96.3%)	9.3	27151 (96.4%)	
Amputation	57	Stroke, n(%)	Yes	1324 (3.7%)	9.3	1013 (3.6%)	
Amputation	57	Systolic blood pressure, mean(SD)		133.3 (15.1)	3.1	132.9 (14.8)	
Amputation	57	Time since last healthcare visit, mean(SD)		0.7 (1.1)	0	0.6 (0.9)	
Amputation	57	Total cholesterol, mean(SD)		4.9 (1.2)	9.7	4.8 (1.2)	
Amputation	58	Age at diagnosis of type 2 diabetes, mean(SD)		53.9 (2.8)	0	53.4 (2.8)	
Amputation	58	BMI, mean(SD)		31.3 (5.5)	7.9	31.2 (5.5)	
Amputation	58	HbA1c, mean(SD)		51.5 (13.6)	2.1	51.5 (13)	
Amputation	58	HDL-cholesterol, mean(SD)		1.2 (0.4)	13.4	1.2 (0.4)	
Amputation	58	Ischemic heart disease, n(%)	No	33862 (88.9%)	8.5	26613 (89.3%)	
Amputation	58	Ischemic heart disease, n(%)	Yes	4245 (11.1%)	8.5	3205 (10.7%)	
Amputation	58	Neuropathy, n(%)	No	36282 (87.2%)	0	25199 (84.5%)	
Amputation	58	Neuropathy, n(%)	Yes	5348 (12.8%)	0	4619 (15.5%)	
Amputation	58	Self-reported physical activity, n(%)	Never	4823 (13.2%)	12.3	3905 (13.1%)	
Amputation	58	Self-reported physical activity, n(%)	<1 times/week	4658 (12.8%)	12.3	3736 (12.5%)	
Amputation	58	Self-reported physical activity, n(%)	regularly - 1-2 times/week	7667 (21%)	12.3	6254 (21%)	
Amputation	58	Self-reported physical activity, n(%)	regularly - 3-5 times/week	8789 (24.1%)	12.3	7231 (24.3%)	
Amputation	58	Self-reported physical activity, n(%)	Daily	10574 (29%)	12.3	8692 (29.2%)	
Amputation	58	Sex, n(%)	Males	25788 (61.9%)	0	18582 (62.3%)	n=41630 vs n=29818
Amputation	58	Sex, n(%)	Females	15840 (38.1%)	0	11236 (37.7%)	
Amputation	58	Smoking status, n(%)	No	30388 (78.5%)	7	23615 (79.2%)	
Amputation	58	Smoking status, n(%)	Yes	8327 (21.5%)	7	6203 (20.8%)	
Amputation	58	Stroke, n(%)	No	36430 (96.1%)	8.9	28680 (96.2%)	

Amputation	58	Stroke, n(%)	Yes	1497 (3.9%)	8.9	1138 (3.8%)	
Amputation	58	Systolic blood pressure, mean(SD)		133.5 (15)	3	133.2 (14.7)	
Amputation	58	Time since last healthcare visit, mean(SD)		0.7 (1.1)	0	0.6 (0.9)	
Amputation	58	Total cholesterol, mean(SD)		4.9 (1.2)	9.5	4.8 (1.2)	
Amputation	59	Age at diagnosis of type 2 diabetes, mean(SD)		54.9 (2.8)	0	54.4 (2.8)	
Amputation	59	BMI, mean(SD)		31.1 (5.4)	7.9	31.1 (5.4)	
Amputation	59	HbA1c, mean(SD)		51.3 (13.1)	2	51.4 (12.6)	
Amputation	59	HDL-cholesterol, mean(SD)		1.2 (0.4)	13.2	1.2 (0.4)	
Amputation	59	Ischemic heart disease, n(%)	No	35531 (88.1%)	8.4	27985 (88.5%)	
Amputation	59	Ischemic heart disease, n(%)	Yes	4819 (11.9%)	8.4	3635 (11.5%)	
Amputation	59	Neuropathy, n(%)	No	38157 (86.6%)	0	26546 (84%)	
Amputation	59	Neuropathy, n(%)	Yes	5902 (13.4%)	0	5074 (16%)	
Amputation	59	Self-reported physical activity, n(%)	Never	5053 (13%)	12	4102 (13%)	
Amputation	59	Self-reported physical activity, n(%)	<1 times/week	4977 (12.8%)	12	3993 (12.6%)	
Amputation	59	Self-reported physical activity, n(%)	regularly - 1-2 times/week	8116 (20.9%)	12	6583 (20.8%)	
Amputation	59	Self-reported physical activity, n(%)	regularly - 3-5 times/week	9313 (24%)	12	7623 (24.1%)	
Amputation	59	Self-reported physical activity, n(%)	Daily	11295 (29.1%)	12	9319 (29.5%)	
Amputation	59	Sex, n(%)	Males	27299 (62%)	0	19690 (62.3%)	n=44059 vs n=31620
Amputation	59	Sex, n(%)	Females	16756 (38%)	0	11930 (37.7%)	
Amputation	59	Smoking status, n(%)	No	32338 (78.9%)	7	25215 (79.7%)	
Amputation	59	Smoking status, n(%)	Yes	8658 (21.1%)	7	6405 (20.3%)	
Amputation	59	Stroke, n(%)	No	38486 (95.8%)	8.9	30357 (96%)	
Amputation	59	Stroke, n(%)	Yes	1672 (4.2%)	8.9	1263 (4%)	

Amputation	59	Systolic blood pressure, mean(SD)		133.9 (15.1)	2.9	133.6 (14.9)	
Amputation	59	Time since last healthcare visit, mean(SD)		0.7 (1.1)	0	0.6 (0.9)	
Amputation	59	Total cholesterol, mean(SD)		4.9 (1.2)	9.2	4.8 (1.2)	
Amputation	60	Age at diagnosis of type 2 diabetes, mean(SD)		55.9 (2.9)	0	55.4 (2.9)	
Amputation	60	BMI, mean(SD)		31 (5.4)	7.8	31 (5.4)	
Amputation	60	HbA1c, mean(SD)		51.1 (13)	2.1	51.2 (12.5)	
Amputation	60	HDL-cholesterol, mean(SD)		1.2 (0.4)	13.2	1.2 (0.4)	
Amputation	60	Ischemic heart disease, n(%)	No	37318 (87.4%)	8.5	29312 (87.9%)	
Amputation	60	Ischemic heart disease, n(%)	Yes	5362 (12.6%)	8.5	4053 (12.1%)	
Amputation	60	Neuropathy, n(%)	No	40150 (86%)	0	27799 (83.3%)	
Amputation	60	Neuropathy, n(%)	Yes	6514 (14%)	0	5566 (16.7%)	
Amputation	60	Self-reported physical activity, n(%)	Never	5367 (13.1%)	12.3	4326 (13%)	
Amputation	60	Self-reported physical activity, n(%)	<1 times/week	5162 (12.6%)	12.3	4115 (12.3%)	
Amputation	60	Self-reported physical activity, n(%)	regularly - 1-2 times/week	8538 (20.9%)	12.3	6961 (20.9%)	
Amputation	60	Self-reported physical activity, n(%)	regularly - 3-5 times/week	9991 (24.4%)	12.3	8145 (24.4%)	
Amputation	60	Self-reported physical activity, n(%)	Daily	11871 (29%)	12.3	9818 (29.4%)	
Amputation	60	Sex, n(%)	Males	28753 (61.6%)	0	20686 (62%)	n=46664 vs n=33365
Amputation	60	Sex, n(%)	Females	17908 (38.4%)	0	12679 (38%)	
Amputation	60	Smoking status, n(%)	No	34421 (79.3%)	7	26727 (80.1%)	
Amputation	60	Smoking status, n(%)	Yes	8998 (20.7%)	7	6638 (19.9%)	
Amputation	60	Stroke, n(%)	No	40600 (95.6%)	9	31960 (95.8%)	
Amputation	60	Stroke, n(%)	Yes	1885 (4.4%)	9	1405 (4.2%)	
Amputation	60	Systolic blood pressure, mean(SD)		134.3 (15.1)	2.9	133.9 (14.7)	

Amputation	60	Time since last healthcare visit, mean(SD)		0.7 (1.1)	0	0.6 (0.9)	
Amputation	60	Total cholesterol, mean(SD)		4.8 (1.2)	9.2	4.8 (1.1)	
Amputation	61	Age at diagnosis of type 2 diabetes, mean(SD)		56.9 (2.9)	0	56.4 (2.9)	
Amputation	61	BMI, mean(SD)		30.9 (5.4)	7.8	30.8 (5.3)	
Amputation	61	HbA1c, mean(SD)		50.8 (12.7)	2.1	51 (12.3)	
Amputation	61	HDL-cholesterol, mean(SD)		1.2 (0.4)	13	1.2 (0.4)	
Amputation	61	Ischemic heart disease, n(%)	No	39572 (86.8%)	8.6	31225 (87.3%)	
Amputation	61	Ischemic heart disease, n(%)	Yes	6026 (13.2%)	8.6	4541 (12.7%)	
Amputation	61	Neuropathy, n(%)	No	42833 (85.8%)	0	29707 (83.1%)	
Amputation	61	Neuropathy, n(%)	Yes	7060 (14.2%)	0	6059 (16.9%)	
Amputation	61	Self-reported physical activity, n(%)	Never	5673 (12.9%)	12.1	4524 (12.6%)	
Amputation	61	Self-reported physical activity, n(%)	<1 times/week	5548 (12.7%)	12.1	4393 (12.3%)	
Amputation	61	Self-reported physical activity, n(%)	regularly - 1-2 times/week	9139 (20.8%)	12.1	7486 (20.9%)	
Amputation	61	Self-reported physical activity, n(%)	regularly - 3-5 times/week	10756 (24.5%)	12.1	8780 (24.5%)	
Amputation	61	Self-reported physical activity, n(%)	Daily	12732 (29%)	12.1	10583 (29.6%)	
Amputation	61	Sex, n(%)	Males	30637 (61.4%)	0	22136 (61.9%)	n=49893 vs n=35766
Amputation	61	Sex, n(%)	Females	19249 (38.6%)	0	13630 (38.1%)	
Amputation	61	Smoking status, n(%)	No	37063 (79.9%)	7	28832 (80.6%)	
Amputation	61	Smoking status, n(%)	Yes	9348 (20.1%)	7	6934 (19.4%)	
Amputation	61	Stroke, n(%)	No	43300 (95.4%)	9	34223 (95.7%)	
Amputation	61	Stroke, n(%)	Yes	2098 (4.6%)	9	1543 (4.3%)	
Amputation	61	Systolic blood pressure, mean(SD)		134.4 (15)	2.9	134.1 (14.8)	
Amputation	61	Time since last healthcare visit, mean(SD)		0.7 (1.1)	0	0.6 (0.9)	

Amputation	61	Total cholesterol, mean(SD)		4.8 (1.2)	9.1	4.8 (1.1)	
Amputation	62	Age at diagnosis of type 2 diabetes, mean(SD)		57.9 (2.9)	0	57.4 (2.9)	
Amputation	62	BMI, mean(SD)		30.7 (5.3)	8	30.7 (5.3)	
Amputation	62	HbA1c, mean(SD)		50.5 (12.4)	2	50.7 (12)	
Amputation	62	HDL-cholesterol, mean(SD)		1.2 (0.4)	12.9	1.2 (0.4)	
Amputation	62	Ischemic heart disease, n(%)	No	41106 (86%)	8.6	32512 (86.5%)	
Amputation	62	Ischemic heart disease, n(%)	Yes	6705 (14%)	8.6	5091 (13.5%)	
Amputation	62	Neuropathy, n(%)	No	44559 (85.2%)	0	30898 (82.2%)	
Amputation	62	Neuropathy, n(%)	Yes	7766 (14.8%)	0	6705 (17.8%)	
Amputation	62	Self-reported physical activity, n(%)	Never	5970 (13%)	12	4819 (12.8%)	
Amputation	62	Self-reported physical activity, n(%)	<1 times/week	5921 (12.9%)	12	4695 (12.5%)	
Amputation	62	Self-reported physical activity, n(%)	regularly - 1-2 times/week	9349 (20.3%)	12	7619 (20.3%)	
Amputation	62	Self-reported physical activity, n(%)	regularly - 3-5 times/week	11273 (24.5%)	12	9265 (24.6%)	
Amputation	62	Self-reported physical activity, n(%)	Daily	13533 (29.4%)	12	11205 (29.8%)	
Amputation	62	Sex, n(%)	Males	31963 (61.1%)	0	23154 (61.6%)	n=52325 vs n=37603
Amputation	62	Sex, n(%)	Females	20359 (38.9%)	0	14449 (38.4%)	
Amputation	62	Smoking status, n(%)	No	39017 (80.2%)	7	30501 (81.1%)	
Amputation	62	Smoking status, n(%)	Yes	9645 (19.8%)	7	7102 (18.9%)	
Amputation	62	Stroke, n(%)	No	45263 (95.1%)	9.1	35865 (95.4%)	
Amputation	62	Stroke, n(%)	Yes	2326 (4.9%)	9.1	1738 (4.6%)	
Amputation	62	Systolic blood pressure, mean(SD)		134.8 (15.1)	2.8	134.4 (14.9)	
Amputation	62	Time since last healthcare visit, mean(SD)		0.7 (1.1)	0	0.6 (0.8)	
Amputation	62	Total cholesterol, mean(SD)		4.8 (1.2)	9	4.8 (1.1)	

Amputation	63	Age at diagnosis of type 2 diabetes, mean(SD)		58.9 (2.9)	0	58.3 (2.9)	
Amputation	63	BMI, mean(SD)		30.6 (5.3)	8.1	30.6 (5.2)	
Amputation	63	HbA1c, mean(SD)		50.3 (12.2)	2	50.5 (11.8)	
Amputation	63	HDL-cholesterol, mean(SD)		1.2 (0.4)	13.2	1.2 (0.4)	
Amputation	63	Ischemic heart disease, n(%)	No	42606 (85.2%)	8.7	33601 (85.6%)	
Amputation	63	Ischemic heart disease, n(%)	Yes	7388 (14.8%)	8.7	5638 (14.4%)	
Amputation	63	Neuropathy, n(%)	No	46354 (84.6%)	0	31996 (81.5%)	
Amputation	63	Neuropathy, n(%)	Yes	8421 (15.4%)	0	7243 (18.5%)	
Amputation	63	Self-reported physical activity, n(%)	Never	6189 (12.8%)	12	4968 (12.7%)	
Amputation	63	Self-reported physical activity, n(%)	<1 times/week	6062 (12.6%)	12	4826 (12.3%)	
Amputation	63	Self-reported physical activity, n(%)	regularly - 1-2 times/week	9873 (20.5%)	12	7983 (20.3%)	
Amputation	63	Self-reported physical activity, n(%)	regularly - 3-5 times/week	11690 (24.3%)	12	9568 (24.4%)	
Amputation	63	Self-reported physical activity, n(%)	Daily	14366 (29.8%)	12	11894 (30.3%)	
Amputation	63	Sex, n(%)	Males	33314 (60.8%)	0	24074 (61.4%)	n=54775 vs n=39239
Amputation	63	Sex, n(%)	Females	21453 (39.2%)	0	15165 (38.6%)	
Amputation	63	Smoking status, n(%)	No	41190 (80.8%)	7	32050 (81.7%)	
Amputation	63	Smoking status, n(%)	Yes	9773 (19.2%)	7	7189 (18.3%)	
Amputation	63	Stroke, n(%)	No	47166 (94.8%)	9.2	37315 (95.1%)	
Amputation	63	Stroke, n(%)	Yes	2581 (5.2%)	9.2	1924 (4.9%)	
Amputation	63	Systolic blood pressure, mean(SD)		135.1 (15.2)	2.9	134.7 (14.9)	
Amputation	63	Time since last healthcare visit, mean(SD)		0.7 (1.1)	0	0.6 (0.9)	
Amputation	63	Total cholesterol, mean(SD)		4.8 (1.1)	9	4.8 (1.1)	
Amputation	64	Age at diagnosis of type 2 diabetes, mean(SD)		59.8 (2.9)	0	59.3 (2.9)	

Amputation	64	BMI, mean(SD)		30.5 (5.3)	8	30.5 (5.3)	
Amputation	64	HbA1c, mean(SD)		50.1 (12)	2	50.3 (11.6)	
Amputation	64	HDL-cholesterol, mean(SD)		1.3 (0.4)	13	1.3 (0.4)	
Amputation	64	Ischemic heart disease, n(%)	No	44036 (84.5%)	8.6	34793 (84.9%)	
Amputation	64	Ischemic heart disease, n(%)	Yes	8097 (15.5%)	8.6	6182 (15.1%)	
Amputation	64	Neuropathy, n(%)	No	48026 (84.2%)	0	33177 (81%)	
Amputation	64	Neuropathy, n(%)	Yes	8999 (15.8%)	0	7798 (19%)	
Amputation	64	Self-reported physical activity, n(%)	Never	6225 (12.4%)	12.1	5009 (12.2%)	
Amputation	64	Self-reported physical activity, n(%)	<1 times/week	6042 (12.1%)	12.1	4797 (11.7%)	
Amputation	64	Self-reported physical activity, n(%)	regularly - 1-2 times/week	10110 (20.2%)	12.1	8234 (20.1%)	
Amputation	64	Self-reported physical activity, n(%)	regularly - 3-5 times/week	12304 (24.5%)	12.1	10144 (24.8%)	
Amputation	64	Self-reported physical activity, n(%)	Daily	15447 (30.8%)	12.1	12791 (31.2%)	
Amputation	64	Sex, n(%)	Males	34638 (60.7%)	0	25009 (61%)	n=57025 vs n=40975
Amputation	64	Sex, n(%)	Females	22384 (39.3%)	0	15966 (39%)	
Amputation	64	Smoking status, n(%)	No	43161 (81.4%)	7	33711 (82.3%)	
Amputation	64	Smoking status, n(%)	Yes	9859 (18.6%)	7	7264 (17.7%)	
Amputation	64	Stroke, n(%)	No	48993 (94.4%)	9	38806 (94.7%)	
Amputation	64	Stroke, n(%)	Yes	2885 (5.6%)	9	2169 (5.3%)	
Amputation	64	Systolic blood pressure, mean(SD)		135.2 (15.2)	2.8	134.8 (14.9)	
Amputation	64	Time since last healthcare visit, mean(SD)		0.7 (1.1)	0	0.6 (0.8)	
Amputation	64	Total cholesterol, mean(SD)		4.8 (1.1)	8.9	4.8 (1.1)	
Amputation	65	Age at diagnosis of type 2 diabetes, mean(SD)		60.8 (2.9)	0	60.2 (2.9)	
Amputation	65	BMI, mean(SD)		30.4 (5.3)	8.1	30.4 (5.3)	

Amputation	65	HbA1c, mean(SD)		49.7 (11.7)	2	49.9 (11.3)	
Amputation	65	HDL-cholesterol, mean(SD)		1.3 (0.4)	12.9	1.3 (0.4)	
Amputation	65	Ischemic heart disease, n(%)	No	45527 (83.7%)	8.6	36039 (84.2%)	
Amputation	65	Ischemic heart disease, n(%)	Yes	8839 (16.3%)	8.6	6759 (15.8%)	
Amputation	65	Neuropathy, n(%)	No	49621 (83.4%)	0	34257 (80%)	
Amputation	65	Neuropathy, n(%)	Yes	9851 (16.6%)	0	8541 (20%)	
Amputation	65	Self-reported physical activity, n(%)	Never	6427 (12.3%)	11.9	5178 (12.1%)	
Amputation	65	Self-reported physical activity, n(%)	<1 times/week	6136 (11.7%)	11.9	4881 (11.4%)	
Amputation	65	Self-reported physical activity, n(%)	regularly - 1-2 times/week	10471 (20%)	11.9	8500 (19.9%)	
Amputation	65	Self-reported physical activity, n(%)	regularly - 3-5 times/week	12632 (24.1%)	11.9	10429 (24.4%)	
Amputation	65	Self-reported physical activity, n(%)	Daily	16713 (31.9%)	11.9	13810 (32.3%)	
Amputation	65	Sex, n(%)	Males	35961 (60.5%)	0	25996 (60.7%)	n=59472 vs n=42798
Amputation	65	Sex, n(%)	Females	23503 (39.5%)	0	16802 (39.3%)	
Amputation	65	Smoking status, n(%)	No	45434 (82.1%)	6.9	35405 (82.7%)	
Amputation	65	Smoking status, n(%)	Yes	9926 (17.9%)	6.9	7393 (17.3%)	
Amputation	65	Stroke, n(%)	No	50882 (94.1%)	9.1	40396 (94.4%)	
Amputation	65	Stroke, n(%)	Yes	3192 (5.9%)	9.1	2402 (5.6%)	
Amputation	65	Systolic blood pressure, mean(SD)		135.3 (15.1)	2.7	134.9 (14.8)	
Amputation	65	Time since last healthcare visit, mean(SD)		0.7 (1.1)	0	0.6 (0.9)	
Amputation	65	Total cholesterol, mean(SD)		4.8 (1.1)	8.7	4.7 (1.1)	
Amputation	66	Age at diagnosis of type 2 diabetes, mean(SD)		61.8 (2.9)	0	61.2 (2.9)	
Amputation	66	BMI, mean(SD)		30.3 (5.3)	8.1	30.3 (5.2)	
Amputation	66	HbA1c, mean(SD)		49.6 (11.7)	1.9	49.7 (11.3)	

Amputation	66	HDL-cholesterol, mean(SD)		1.3 (0.4)	12.7	1.3 (0.4)	
Amputation	66	Ischemic heart disease, n(%)	No	47211 (83.1%)	8.5	37462 (83.5%)	
Amputation	66	Ischemic heart disease, n(%)	Yes	9583 (16.9%)	8.5	7386 (16.5%)	
Amputation	66	Neuropathy, n(%)	No	51185 (82.5%)	0	35413 (79%)	
Amputation	66	Neuropathy, n(%)	Yes	10855 (17.5%)	0	9435 (21%)	
Amputation	66	Self-reported physical activity, n(%)	Never	6498 (11.9%)	11.7	5203 (11.6%)	
Amputation	66	Self-reported physical activity, n(%)	<1 times/week	6143 (11.2%)	11.7	4899 (10.9%)	
Amputation	66	Self-reported physical activity, n(%)	regularly - 1-2 times/week	10560 (19.3%)	11.7	8587 (19.1%)	
Amputation	66	Self-reported physical activity, n(%)	regularly - 3-5 times/week	13251 (24.2%)	11.7	10978 (24.5%)	
Amputation	66	Self-reported physical activity, n(%)	Daily	18323 (33.5%)	11.7	15181 (33.8%)	
Amputation	66	Sex, n(%)	Males	37308 (60.1%)	0	27137 (60.5%)	n=62040 vs n=44848
Amputation	66	Sex, n(%)	Females	24721 (39.9%)	0	17711 (39.5%)	
Amputation	66	Smoking status, n(%)	No	47884 (82.8%)	6.8	37386 (83.4%)	
Amputation	66	Smoking status, n(%)	Yes	9952 (17.2%)	6.8	7462 (16.6%)	
Amputation	66	Stroke, n(%)	No	53011 (93.8%)	8.9	42209 (94.1%)	
Amputation	66	Stroke, n(%)	Yes	3501 (6.2%)	8.9	2639 (5.9%)	
Amputation	66	Systolic blood pressure, mean(SD)		135.6 (15.3)	2.6	135.2 (15)	
Amputation	66	Time since last healthcare visit, mean(SD)		0.7 (1.1)	0	0.6 (0.9)	
Amputation	66	Total cholesterol, mean(SD)		4.8 (1.1)	8.6	4.7 (1.1)	
Amputation	67	Age at diagnosis of type 2 diabetes, mean(SD)		62.7 (2.9)	0	62.1 (2.9)	
Amputation	67	BMI, mean(SD)		30.2 (5.2)	8.1	30.2 (5.2)	
Amputation	67	HbA1c, mean(SD)		49.3 (11.3)	1.9	49.5 (11)	
Amputation	67	HDL-cholesterol, mean(SD)		1.3 (0.4)	12.5	1.3 (0.4)	

Amputation	67	Ischemic heart disease, n(%)	No	48315 (82.4%)	8.3	38432 (82.9%)	
Amputation	67	Ischemic heart disease, n(%)	Yes	10345 (17.6%)	8.3	7926 (17.1%)	
Amputation	67	Neuropathy, n(%)	No	52227 (81.6%)	0	36167 (78%)	
Amputation	67	Neuropathy, n(%)	Yes	11772 (18.4%)	0	10191 (22%)	
Amputation	67	Self-reported physical activity, n(%)	Never	6671 (11.8%)	11.6	5322 (11.5%)	
Amputation	67	Self-reported physical activity, n(%)	<1 times/week	6223 (11%)	11.6	4940 (10.7%)	
Amputation	67	Self-reported physical activity, n(%)	regularly - 1-2 times/week	10499 (18.6%)	11.6	8505 (18.3%)	
Amputation	67	Self-reported physical activity, n(%)	regularly - 3-5 times/week	13721 (24.3%)	11.6	11404 (24.6%)	
Amputation	67	Self-reported physical activity, n(%)	Daily	19451 (34.4%)	11.6	16187 (34.9%)	
Amputation	67	Sex, n(%)	Males	38172 (59.7%)	0	27873 (60.1%)	n=63999 vs n=46358
Amputation	67	Sex, n(%)	Females	25815 (40.3%)	0	18485 (39.9%)	
Amputation	67	Smoking status, n(%)	No	50080 (83.8%)	6.6	39140 (84.4%)	
Amputation	67	Smoking status, n(%)	Yes	9685 (16.2%)	6.6	7218 (15.6%)	
Amputation	67	Stroke, n(%)	No	54516 (93.4%)	8.8	43477 (93.8%)	
Amputation	67	Stroke, n(%)	Yes	3836 (6.6%)	8.8	2881 (6.2%)	
Amputation	67	Systolic blood pressure, mean(SD)		135.7 (15.2)	2.6	135.4 (14.9)	
Amputation	67	Time since last healthcare visit, mean(SD)		0.7 (1.1)	0	0.6 (0.9)	
Amputation	67	Total cholesterol, mean(SD)		4.8 (1.1)	8.4	4.7 (1.1)	
Amputation	68	Age at diagnosis of type 2 diabetes, mean(SD)		63.6 (2.9)	0	63.1 (2.9)	
Amputation	68	BMI, mean(SD)		30.1 (5.3)	7.9	30.1 (5.2)	
Amputation	68	HbA1c, mean(SD)		49.1 (11.1)	1.8	49.2 (10.7)	
Amputation	68	HDL-cholesterol, mean(SD)		1.3 (0.4)	12.3	1.3 (0.4)	
Amputation	68	Ischemic heart disease, n(%)	No	48944 (81.6%)	8.2	39163 (82.1%)	

Amputation	68	Ischemic heart disease, n(%)	Yes	11030 (18.4%)	8.2	8517 (17.9%)	
Amputation	68	Neuropathy, n(%)	No	52715 (80.7%)	0	36726 (77%)	
Amputation	68	Neuropathy, n(%)	Yes	12622 (19.3%)	0	10954 (23%)	
Amputation	68	Self-reported physical activity, n(%)	Never	6886 (11.9%)	11.3	5537 (11.6%)	
Amputation	68	Self-reported physical activity, n(%)	<1 times/week	6382 (11%)	11.3	5114 (10.7%)	
Amputation	68	Self-reported physical activity, n(%)	regularly - 1-2 times/week	10457 (18.1%)	11.3	8451 (17.7%)	
Amputation	68	Self-reported physical activity, n(%)	regularly - 3-5 times/week	13968 (24.1%)	11.3	11561 (24.2%)	
Amputation	68	Self-reported physical activity, n(%)	Daily	20240 (34.9%)	11.3	17017 (35.7%)	
Amputation	68	Sex, n(%)	Males	38720 (59.3%)	0	28455 (59.7%)	n=65337 vs n=47680
Amputation	68	Sex, n(%)	Females	26610 (40.7%)	0	19225 (40.3%)	
Amputation	68	Smoking status, n(%)	No	51577 (84.5%)	6.6	40539 (85%)	
Amputation	68	Smoking status, n(%)	Yes	9474 (15.5%)	6.6	7141 (15%)	
Amputation	68	Stroke, n(%)	No	55526 (93.1%)	8.7	44534 (93.4%)	
Amputation	68	Stroke, n(%)	Yes	4117 (6.9%)	8.7	3146 (6.6%)	
Amputation	68	Systolic blood pressure, mean(SD)		135.7 (15.2)	2.5	135.3 (14.9)	
Amputation	68	Time since last healthcare visit, mean(SD)		0.7 (1.1)	0	0.6 (0.9)	
Amputation	68	Total cholesterol, mean(SD)		4.7 (1.1)	8.3	4.7 (1.1)	
Amputation	69	Age at diagnosis of type 2 diabetes, mean(SD)		64.5 (2.9)	0	64 (2.9)	
Amputation	69	BMI, mean(SD)		30 (5.2)	7.8	30 (5.2)	
Amputation	69	HbA1c, mean(SD)		49 (10.9)	1.9	49.1 (10.6)	
Amputation	69	HDL-cholesterol, mean(SD)		1.3 (0.4)	12.2	1.3 (0.4)	
Amputation	69	Ischemic heart disease, n(%)	No	49361 (80.8%)	7.8	39636 (81.3%)	
Amputation	69	Ischemic heart disease, n(%)	Yes	11722 (19.2%)	7.8	9090 (18.7%)	

Amputation	69	Neuropathy, n(%)	No	52762 (79.7%)	0	37032 (76%)	
Amputation	69	Neuropathy, n(%)	Yes	13480 (20.3%)	0	11694 (24%)	
Amputation	69	Self-reported physical activity, n(%)	Never	6993 (11.9%)	11.2	5662 (11.6%)	
Amputation	69	Self-reported physical activity, n(%)	<1 times/week	6479 (11%)	11.2	5174 (10.6%)	
Amputation	69	Self-reported physical activity, n(%)	regularly - 1-2 times/week	10717 (18.2%)	11.2	8804 (18.1%)	
Amputation	69	Self-reported physical activity, n(%)	regularly - 3-5 times/week	14021 (23.8%)	11.2	11698 (24%)	
Amputation	69	Self-reported physical activity, n(%)	Daily	20640 (35.1%)	11.2	17388 (35.7%)	
Amputation	69	Sex, n(%)	Males	38952 (58.8%)	0	28839 (59.2%)	n=66242 vs n=48726
Amputation	69	Sex, n(%)	Females	27283 (41.2%)	0	19887 (40.8%)	
Amputation	69	Smoking status, n(%)	No	52872 (85.3%)	6.4	41858 (85.9%)	
Amputation	69	Smoking status, n(%)	Yes	9098 (14.7%)	6.4	6868 (14.1%)	
Amputation	69	Stroke, n(%)	No	56425 (92.9%)	8.3	45409 (93.2%)	
Amputation	69	Stroke, n(%)	Yes	4338 (7.1%)	8.3	3317 (6.8%)	
Amputation	69	Systolic blood pressure, mean(SD)		135.8 (15.2)	2.4	135.5 (14.9)	
Amputation	69	Time since last healthcare visit, mean(SD)		0.7 (1.1)	0	0.6 (0.9)	
Amputation	69	Total cholesterol, mean(SD)		4.7 (1.1)	8.2	4.7 (1.1)	
Amputation	70	Age at diagnosis of type 2 diabetes, mean(SD)		65.5 (3)	0	64.9 (2.9)	
Amputation	70	BMI, mean(SD)		29.9 (5.2)	7.7	29.9 (5.1)	
Amputation	70	HbA1c, mean(SD)		48.9 (10.9)	1.9	48.9 (10.5)	
Amputation	70	HDL-cholesterol, mean(SD)		1.3 (0.4)	12.3	1.3 (0.4)	
Amputation	70	Ischemic heart disease, n(%)	No	49083 (80%)	7.7	39367 (80.6%)	
Amputation	70	Ischemic heart disease, n(%)	Yes	12238 (20%)	7.7	9486 (19.4%)	
Amputation	70	Neuropathy, n(%)	No	52170 (78.6%)	0	36479 (74.7%)	

Amputation	70	Neuropathy, n(%)	Yes	14243 (21.4%)	0	12374 (25.3%)	
Amputation	70	Self-reported physical activity, n(%)	Never	7071 (12%)	11.1	5787 (11.8%)	
Amputation	70	Self-reported physical activity, n(%)	<1 times/week	6358 (10.8%)	11.1	5082 (10.4%)	
Amputation	70	Self-reported physical activity, n(%)	regularly - 1-2 times/week	10707 (18.1%)	11.1	8768 (17.9%)	
Amputation	70	Self-reported physical activity, n(%)	regularly - 3-5 times/week	14145 (24%)	11.1	11809 (24.2%)	
Amputation	70	Self-reported physical activity, n(%)	Daily	20770 (35.2%)	11.1	17407 (35.6%)	
Amputation	70	Sex, n(%)	Males	38804 (58.4%)	0	28771 (58.9%)	n=66413 vs n=48853
Amputation	70	Sex, n(%)	Females	27602 (41.6%)	0	20082 (41.1%)	
Amputation	70	Smoking status, n(%)	No	53461 (86.1%)	6.5	42330 (86.6%)	
Amputation	70	Smoking status, n(%)	Yes	8628 (13.9%)	6.5	6523 (13.4%)	
Amputation	70	Stroke, n(%)	No	56452 (92.6%)	8.2	45355 (92.8%)	
Amputation	70	Stroke, n(%)	Yes	4529 (7.4%)	8.2	3498 (7.2%)	
Amputation	70	Systolic blood pressure, mean(SD)		135.9 (15.3)	2.4	135.5 (15)	
Amputation	70	Time since last healthcare visit, mean(SD)		0.7 (1.1)	0	0.6 (0.9)	
Amputation	70	Total cholesterol, mean(SD)		4.7 (1.1)	8.2	4.7 (1.1)	
Amputation	71	Age at diagnosis of type 2 diabetes, mean(SD)		66.4 (3)	0	65.8 (3)	
Amputation	71	BMI, mean(SD)		29.8 (5.1)	7.6	29.8 (5.1)	
Amputation	71	HbA1c, mean(SD)		48.8 (10.7)	1.7	48.9 (10.2)	
Amputation	71	HDL-cholesterol, mean(SD)		1.3 (0.4)	12.2	1.3 (0.4)	
Amputation	71	Ischemic heart disease, n(%)	No	48171 (79.2%)	7.4	38681 (79.6%)	
Amputation	71	Ischemic heart disease, n(%)	Yes	12687 (20.8%)	7.4	9922 (20.4%)	
Amputation	71	Neuropathy, n(%)	No	51013 (77.6%)	0	35753 (73.6%)	
Amputation	71	Neuropathy, n(%)	Yes	14735 (22.4%)	0	12850 (26.4%)	

Amputation	71	Self-reported physical activity, n(%)	Never	7075 (12.1%)	10.9	5801 (11.9%)	
Amputation	71	Self-reported physical activity, n(%)	<1 times/week	6578 (11.2%)	10.9	5263 (10.8%)	
Amputation	71	Self-reported physical activity, n(%)	regularly - 1-2 times/week	10664 (18.2%)	10.9	8760 (18%)	
Amputation	71	Self-reported physical activity, n(%)	regularly - 3-5 times/week	13752 (23.5%)	10.9	11507 (23.7%)	
Amputation	71	Self-reported physical activity, n(%)	Daily	20540 (35%)	10.9	17272 (35.5%)	
Amputation	71	Sex, n(%)	Males	38135 (58%)	0	28325 (58.3%)	n=65748 vs n=48603
Amputation	71	Sex, n(%)	Females	27605 (42%)	0	20278 (41.7%)	
Amputation	71	Smoking status, n(%)	No	53408 (86.9%)	6.5	42478 (87.4%)	
Amputation	71	Smoking status, n(%)	Yes	8070 (13.1%)	6.5	6125 (12.6%)	
Amputation	71	Stroke, n(%)	No	55785 (92.2%)	8	44982 (92.5%)	
Amputation	71	Stroke, n(%)	Yes	4721 (7.8%)	8	3621 (7.5%)	
Amputation	71	Systolic blood pressure, mean(SD)		136 (15.3)	2.3	135.7 (15)	
Amputation	71	Time since last healthcare visit, mean(SD)		0.7 (1.2)	0	0.6 (0.9)	
Amputation	71	Total cholesterol, mean(SD)		4.7 (1.1)	8.3	4.6 (1.1)	
Amputation	72	Age at diagnosis of type 2 diabetes, mean(SD)		67.3 (3)	0	66.8 (3)	
Amputation	72	BMI, mean(SD)		29.7 (5.1)	7.6	29.6 (5.1)	
Amputation	72	HbA1c, mean(SD)		48.6 (10.4)	1.8	48.7 (10)	
Amputation	72	HDL-cholesterol, mean(SD)		1.3 (0.4)	12.2	1.3 (0.4)	
Amputation	72	Ischemic heart disease, n(%)	No	46375 (78.4%)	7.3	37357 (78.9%)	
Amputation	72	Ischemic heart disease, n(%)	Yes	12805 (21.6%)	7.3	9983 (21.1%)	
Amputation	72	Neuropathy, n(%)	No	48749 (76.4%)	0	34142 (72.1%)	
Amputation	72	Neuropathy, n(%)	Yes	15098 (23.6%)	0	13198 (27.9%)	
Amputation	72	Self-reported physical activity, n(%)	Never	7056 (12.4%)	10.6	5782 (12.2%)	

Amputation	72	Self-reported physical activity, n(%)	<1 times/week	6270 (11%)	10.6	5031 (10.6%)	
Amputation	72	Self-reported physical activity, n(%)	regularly - 1-2 times/week	10485 (18.4%)	10.6	8617 (18.2%)	
Amputation	72	Self-reported physical activity, n(%)	regularly - 3-5 times/week	13329 (23.4%)	10.6	11128 (23.5%)	
Amputation	72	Self-reported physical activity, n(%)	Daily	19921 (34.9%)	10.6	16782 (35.4%)	
Amputation	72	Sex, n(%)	Males	36668 (57.4%)	0	27427 (57.9%)	n=63847 vs n=47340
Amputation	72	Sex, n(%)	Females	27171 (42.6%)	0	19913 (42.1%)	
Amputation	72	Smoking status, n(%)	No	52373 (87.5%)	6.3	41599 (87.9%)	
Amputation	72	Smoking status, n(%)	Yes	7480 (12.5%)	6.3	5741 (12.1%)	
Amputation	72	Stroke, n(%)	No	53884 (91.6%)	7.8	43537 (92%)	
Amputation	72	Stroke, n(%)	Yes	4960 (8.4%)	7.8	3803 (8%)	
Amputation	72	Systolic blood pressure, mean(SD)		136 (15.5)	2.3	135.7 (15.2)	
Amputation	72	Time since last healthcare visit, mean(SD)		0.7 (1.2)	0	0.6 (0.9)	
Amputation	72	Total cholesterol, mean(SD)		4.7 (1.1)	8.2	4.6 (1.1)	
Amputation	73	Age at diagnosis of type 2 diabetes, mean(SD)		68.3 (3)	0	67.7 (3)	
Amputation	73	BMI, mean(SD)		29.5 (5.1)	7.8	29.5 (5.1)	
Amputation	73	HbA1c, mean(SD)		48.6 (10.4)	1.8	48.7 (10)	
Amputation	73	HDL-cholesterol, mean(SD)		1.3 (0.4)	12.3	1.3 (0.4)	
Amputation	73	Ischemic heart disease, n(%)	No	44208 (77.5%)	7.4	35514 (78%)	
Amputation	73	Ischemic heart disease, n(%)	Yes	12843 (22.5%)	7.4	10023 (22%)	
Amputation	73	Neuropathy, n(%)	No	46396 (75.3%)	0	32245 (70.8%)	
Amputation	73	Neuropathy, n(%)	Yes	15231 (24.7%)	0	13292 (29.2%)	
Amputation	73	Self-reported physical activity, n(%)	Never	7059 (12.8%)	10.8	5753 (12.6%)	
Amputation	73	Self-reported physical activity, n(%)	<1 times/week	6162 (11.2%)	10.8	4929 (10.8%)	

Amputation	73	Self-reported physical activity, n(%)	regularly - 1-2 times/week	10122 (18.4%)	10.8	8350 (18.3%)	
Amputation	73	Self-reported physical activity, n(%)	regularly - 3-5 times/week	12751 (23.2%)	10.8	10659 (23.4%)	
Amputation	73	Self-reported physical activity, n(%)	Daily	18897 (34.4%)	10.8	15846 (34.8%)	
Amputation	73	Sex, n(%)	Males	34907 (56.7%)	0	26058 (57.2%)	n=61627 vs n=45537
Amputation	73	Sex, n(%)	Females	26711 (43.3%)	0	19479 (42.8%)	
Amputation	73	Smoking status, n(%)	No	50971 (88.4%)	6.5	40388 (88.7%)	
Amputation	73	Smoking status, n(%)	Yes	6661 (11.6%)	6.5	5149 (11.3%)	
Amputation	73	Stroke, n(%)	No	51551 (90.9%)	8	41510 (91.2%)	
Amputation	73	Stroke, n(%)	Yes	5161 (9.1%)	8	4027 (8.8%)	
Amputation	73	Systolic blood pressure, mean(SD)		136 (15.5)	2.3	135.7 (15.2)	
Amputation	73	Time since last healthcare visit, mean(SD)		0.7 (1.2)	0	0.6 (0.9)	
Amputation	73	Total cholesterol, mean(SD)		4.7 (1.1)	8.4	4.6 (1.1)	
Amputation	74	Age at diagnosis of type 2 diabetes, mean(SD)		69.2 (3.1)	0	68.7 (3)	
Amputation	74	BMI, mean(SD)		29.4 (5.1)	7.8	29.4 (5.1)	
Amputation	74	HbA1c, mean(SD)		48.6 (10.2)	1.7	48.6 (9.8)	
Amputation	74	HDL-cholesterol, mean(SD)		1.3 (0.4)	12.4	1.3 (0.4)	
Amputation	74	Ischemic heart disease, n(%)	No	41413 (76.6%)	7.5	33279 (77.2%)	
Amputation	74	Ischemic heart disease, n(%)	Yes	12648 (23.4%)	7.5	9815 (22.8%)	
Amputation	74	Neuropathy, n(%)	No	43226 (74%)	0	29902 (69.4%)	
Amputation	74	Neuropathy, n(%)	Yes	15225 (26%)	0	13192 (30.6%)	
Amputation	74	Self-reported physical activity, n(%)	Never	7010 (13.4%)	10.8	5696 (13.2%)	
Amputation	74	Self-reported physical activity, n(%)	<1 times/week	6033 (11.6%)	10.8	4800 (11.1%)	
Amputation	74	Self-reported physical activity, n(%)	regularly - 1-2 times/week	9677 (18.6%)	10.8	7941 (18.4%)	

Amputation	74	Self-reported physical activity, n(%)	regularly - 3-5 times/week	11710 (22.5%)	10.8	9802 (22.7%)	
Amputation	74	Self-reported physical activity, n(%)	Daily	17710 (34%)	10.8	14855 (34.5%)	
Amputation	74	Sex, n(%)	Males	32755 (56.1%)	0	24310 (56.4%)	n=58451 vs n=43094
Amputation	74	Sex, n(%)	Females	25683 (43.9%)	0	18784 (43.6%)	
Amputation	74	Smoking status, n(%)	No	48693 (89.2%)	6.6	38537 (89.4%)	
Amputation	74	Smoking status, n(%)	Yes	5922 (10.8%)	6.6	4557 (10.6%)	
Amputation	74	Stroke, n(%)	No	48579 (90.4%)	8.1	39085 (90.7%)	
Amputation	74	Stroke, n(%)	Yes	5165 (9.6%)	8.1	4009 (9.3%)	
Amputation	74	Systolic blood pressure, mean(SD)		136 (15.7)	2.3	135.7 (15.4)	
Amputation	74	Time since last healthcare visit, mean(SD)		0.7 (1.2)	0	0.6 (0.9)	
Amputation	74	Total cholesterol, mean(SD)		4.6 (1.1)	8.5	4.6 (1.1)	
Amputation	75	Age at diagnosis of type 2 diabetes, mean(SD)		70.2 (3.1)	0	69.6 (3)	
Amputation	75	BMI, mean(SD)		29.3 (5)	8	29.3 (5)	
Amputation	75	HbA1c, mean(SD)		48.5 (10.1)	1.8	48.6 (9.8)	
Amputation	75	HDL-cholesterol, mean(SD)		1.3 (0.4)	12.5	1.3 (0.4)	
Amputation	75	Ischemic heart disease, n(%)	No	38266 (75.5%)	7.5	30601 (76%)	
Amputation	75	Ischemic heart disease, n(%)	Yes	12395 (24.5%)	7.5	9639 (24%)	
Amputation	75	Neuropathy, n(%)	No	40034 (73.1%)	0	27475 (68.3%)	
Amputation	75	Neuropathy, n(%)	Yes	14714 (26.9%)	0	12765 (31.7%)	
Amputation	75	Self-reported physical activity, n(%)	Never	6816 (14%)	10.9	5549 (13.8%)	
Amputation	75	Self-reported physical activity, n(%)	<1 times/week	5633 (11.6%)	10.9	4472 (11.1%)	
Amputation	75	Self-reported physical activity, n(%)	regularly - 1-2 times/week	9121 (18.7%)	10.9	7459 (18.5%)	
Amputation	75	Self-reported physical activity, n(%)	regularly - 3-5 times/week	10863 (22.3%)	10.9	9124 (22.7%)	

Amputation	75	Self-reported physical activity, n(%)	Daily	16321 (33.5%)	10.9	13636 (33.9%)	
Amputation	75	Sex, n(%)	Males	30180 (55.1%)	0	22357 (55.6%)	n=54748 vs n=40240
Amputation	75	Sex, n(%)	Females	24564 (44.9%)	0	17883 (44.4%)	
Amputation	75	Smoking status, n(%)	No	45937 (90%)	6.8	36354 (90.3%)	
Amputation	75	Smoking status, n(%)	Yes	5113 (10%)	6.8	3886 (9.7%)	
Amputation	75	Stroke, n(%)	No	45247 (90%)	8.1	36308 (90.2%)	
Amputation	75	Stroke, n(%)	Yes	5045 (10%)	8.1	3932 (9.8%)	
Amputation	75	Systolic blood pressure, mean(SD)		136.3 (15.7)	2.3	135.9 (15.5)	
Amputation	75	Time since last healthcare visit, mean(SD)		0.7 (1.2)	0	0.6 (0.9)	
Amputation	75	Total cholesterol, mean(SD)		4.6 (1.1)	8.6	4.6 (1.1)	
Amputation	76	Age at diagnosis of type 2 diabetes, mean(SD)		71.3 (3.1)	0	70.6 (3)	
Amputation	76	BMI, mean(SD)		29.2 (5)	8.1	29.2 (5)	
Amputation	76	HbA1c, mean(SD)		48.6 (10.1)	1.9	48.6 (9.8)	
Amputation	76	HDL-cholesterol, mean(SD)		1.3 (0.4)	13.1	1.3 (0.4)	
Amputation	76	Ischemic heart disease, n(%)	No	35048 (74.5%)	7.6	27864 (75%)	
Amputation	76	Ischemic heart disease, n(%)	Yes	11998 (25.5%)	7.6	9284 (25%)	
Amputation	76	Neuropathy, n(%)	No	36890 (72.4%)	0	24973 (67.2%)	
Amputation	76	Neuropathy, n(%)	Yes	14028 (27.6%)	0	12175 (32.8%)	
Amputation	76	Self-reported physical activity, n(%)	Never	6569 (14.5%)	11.2	5331 (14.4%)	
Amputation	76	Self-reported physical activity, n(%)	<1 times/week	5257 (11.6%)	11.2	4164 (11.2%)	
Amputation	76	Self-reported physical activity, n(%)	regularly - 1-2 times/week	8407 (18.6%)	11.2	6829 (18.4%)	
Amputation	76	Self-reported physical activity, n(%)	regularly - 3-5 times/week	9943 (22%)	11.2	8278 (22.3%)	
Amputation	76	Self-reported physical activity, n(%)	Daily	15019 (33.2%)	11.2	12546 (33.8%)	

Amputation	76	Sex, n(%)	Males	27736 (54.5%)	0	20427 (55%)	n=50918 vs n=37148
Amputation	76	Sex, n(%)	Females	23179 (45.5%)	0	16721 (45%)	
Amputation	76	Smoking status, n(%)	No	42995 (90.7%)	6.9	33830 (91.1%)	
Amputation	76	Smoking status, n(%)	Yes	4393 (9.3%)	6.9	3318 (8.9%)	
Amputation	76	Stroke, n(%)	No	41728 (89.4%)	8.3	33312 (89.7%)	
Amputation	76	Stroke, n(%)	Yes	4973 (10.6%)	8.3	3836 (10.3%)	
Amputation	76	Systolic blood pressure, mean(SD)		136.1 (15.8)	2.4	135.8 (15.5)	
Amputation	76	Time since last healthcare visit, mean(SD)		0.7 (1.2)	0	0.6 (0.9)	
Amputation	76	Total cholesterol, mean(SD)		4.6 (1.1)	9.1	4.6 (1.1)	
Amputation	77	Age at diagnosis of type 2 diabetes, mean(SD)		72.3 (3.1)	0	71.6 (3)	
Amputation	77	BMI, mean(SD)		29 (4.9)	8.3	29 (4.9)	
Amputation	77	HbA1c, mean(SD)		48.5 (10)	1.9	48.6 (9.6)	
Amputation	77	HDL-cholesterol, mean(SD)		1.3 (0.4)	13.6	1.3 (0.4)	
Amputation	77	Ischemic heart disease, n(%)	No	32011 (73.4%)	7.7	25309 (73.9%)	
Amputation	77	Ischemic heart disease, n(%)	Yes	11594 (26.6%)	7.7	8954 (26.1%)	
Amputation	77	Neuropathy, n(%)	No	33939 (71.8%)	0	22721 (66.3%)	
Amputation	77	Neuropathy, n(%)	Yes	13327 (28.2%)	0	11542 (33.7%)	
Amputation	77	Self-reported physical activity, n(%)	Never	6215 (14.9%)	11.6	5009 (14.6%)	
Amputation	77	Self-reported physical activity, n(%)	<1 times/week	5026 (12%)	11.6	3974 (11.6%)	
Amputation	77	Self-reported physical activity, n(%)	regularly - 1-2 times/week	7734 (18.5%)	11.6	6315 (18.4%)	
Amputation	77	Self-reported physical activity, n(%)	regularly - 3-5 times/week	9086 (21.8%)	11.6	7548 (22%)	
Amputation	77	Self-reported physical activity, n(%)	Daily	13710 (32.8%)	11.6	11417 (33.3%)	
Amputation	77	Sex, n(%)	Males	25364 (53.7%)	0	18686 (54.5%)	n=47266 vs n=34263

Amputation	77	Sex, n(%)	Females	21896 (46.3%)	0	15577 (45.5%)	
Amputation	77	Smoking status, n(%)	No	40196 (91.5%)	7.1	31472 (91.9%)	
Amputation	77	Smoking status, n(%)	Yes	3737 (8.5%)	7.1	2791 (8.1%)	
Amputation	77	Stroke, n(%)	No	38519 (88.9%)	8.3	30595 (89.3%)	
Amputation	77	Stroke, n(%)	Yes	4810 (11.1%)	8.3	3668 (10.7%)	
Amputation	77	Systolic blood pressure, mean(SD)		136.2 (15.9)	2.4	135.9 (15.6)	
Amputation	77	Time since last healthcare visit, mean(SD)		0.7 (1.2)	0	0.6 (0.9)	
Amputation	77	Total cholesterol, mean(SD)		4.6 (1.1)	9.5	4.6 (1.1)	
Amputation	78	Age at diagnosis of type 2 diabetes, mean(SD)		73.3 (3)	0	72.6 (3)	
Amputation	78	BMI, mean(SD)		28.9 (4.9)	8.6	28.9 (4.9)	
Amputation	78	HbA1c, mean(SD)		48.6 (10)	2	48.7 (9.7)	
Amputation	78	HDL-cholesterol, mean(SD)		1.3 (0.4)	14	1.3 (0.4)	
Amputation	78	Ischemic heart disease, n(%)	No	29340 (72.4%)	7.9	23013 (72.8%)	
Amputation	78	Ischemic heart disease, n(%)	Yes	11199 (27.6%)	7.9	8618 (27.2%)	
Amputation	78	Neuropathy, n(%)	No	31256 (71%)	0	20707 (65.5%)	
Amputation	78	Neuropathy, n(%)	Yes	12756 (29%)	0	10924 (34.5%)	
Amputation	78	Self-reported physical activity, n(%)	Never	6016 (15.5%)	11.9	4790 (15.1%)	
Amputation	78	Self-reported physical activity, n(%)	<1 times/week	4802 (12.4%)	11.9	3767 (11.9%)	
Amputation	78	Self-reported physical activity, n(%)	regularly - 1-2 times/week	7116 (18.4%)	11.9	5815 (18.4%)	
Amputation	78	Self-reported physical activity, n(%)	regularly - 3-5 times/week	8338 (21.5%)	11.9	6884 (21.8%)	
Amputation	78	Self-reported physical activity, n(%)	Daily	12491 (32.2%)	11.9	10375 (32.8%)	
Amputation	78	Sex, n(%)	Males	23121 (52.5%)	0	16874 (53.3%)	n=44012 vs n=31631
Amputation	78	Sex, n(%)	Females	20886 (47.5%)	0	14757 (46.7%)	

Amputation	78	Smoking status, n(%)	No	37576 (92%)	7.2	29208 (92.3%)	
Amputation	78	Smoking status, n(%)	Yes	3262 (8%)	7.2	2423 (7.7%)	
Amputation	78	Stroke, n(%)	No	35584 (88.5%)	8.6	28109 (88.9%)	
Amputation	78	Stroke, n(%)	Yes	4636 (11.5%)	8.6	3522 (11.1%)	
Amputation	78	Systolic blood pressure, mean(SD)		136.4 (16)	2.5	136 (15.7)	
Amputation	78	Time since last healthcare visit, mean(SD)		0.7 (1.2)	0	0.6 (0.9)	
Amputation	78	Total cholesterol, mean(SD)		4.6 (1.1)	9.8	4.6 (1.1)	
Amputation	79	Age at diagnosis of type 2 diabetes, mean(SD)		74.2 (3.1)	0	73.6 (3)	
Amputation	79	BMI, mean(SD)		28.8 (4.9)	9	28.7 (4.8)	
Amputation	79	HbA1c, mean(SD)		48.8 (10.1)	2	48.9 (9.8)	
Amputation	79	HDL-cholesterol, mean(SD)		1.3 (0.4)	14.8	1.3 (0.4)	
Amputation	79	Ischemic heart disease, n(%)	No	27113 (71.6%)	8.2	21049 (72%)	
Amputation	79	Ischemic heart disease, n(%)	Yes	10756 (28.4%)	8.2	8185 (28%)	
Amputation	79	Neuropathy, n(%)	No	28979 (70.2%)	0	18787 (64.3%)	
Amputation	79	Neuropathy, n(%)	Yes	12288 (29.8%)	0	10447 (35.7%)	
Amputation	79	Self-reported physical activity, n(%)	Never	5837 (16.1%)	12.3	4540 (15.5%)	
Amputation	79	Self-reported physical activity, n(%)	<1 times/week	4608 (12.7%)	12.3	3545 (12.1%)	
Amputation	79	Self-reported physical activity, n(%)	regularly - 1-2 times/week	6789 (18.8%)	12.3	5474 (18.7%)	
Amputation	79	Self-reported physical activity, n(%)	regularly - 3-5 times/week	7586 (21%)	12.3	6273 (21.5%)	
Amputation	79	Self-reported physical activity, n(%)	Daily	11373 (31.4%)	12.3	9402 (32.2%)	
Amputation	79	Sex, n(%)	Males	21181 (51.3%)	0	15280 (52.3%)	n=41267 vs n=29234
Amputation	79	Sex, n(%)	Females	20081 (48.7%)	0	13954 (47.7%)	
Amputation	79	Smoking status, n(%)	No	35426 (92.7%)	7.4	27182 (93%)	

Amputation	79	Smoking status, n(%)	Yes	2784 (7.3%)	7.4	2052 (7%)	
Amputation	79	Stroke, n(%)	No	32974 (87.8%)	9	25814 (88.3%)	
Amputation	79	Stroke, n(%)	Yes	4586 (12.2%)	9	3420 (11.7%)	
Amputation	79	Systolic blood pressure, mean(SD)		136.4 (16.1)	2.5	136.1 (15.9)	
Amputation	79	Time since last healthcare visit, mean(SD)		0.8 (1.2)	0	0.6 (1)	
Amputation	79	Total cholesterol, mean(SD)		4.6 (1.1)	10.4	4.6 (1.1)	
Amputation	80	Age at diagnosis of type 2 diabetes, mean(SD)		75.2 (3.1)	0	74.5 (3)	
Amputation	80	BMI, mean(SD)		28.6 (4.8)	9.5	28.6 (4.8)	
Amputation	80	HbA1c, mean(SD)		48.9 (10.3)	2.1	49 (10)	
Amputation	80	HDL-cholesterol, mean(SD)		1.3 (0.4)	15.4	1.3 (0.4)	
Amputation	80	Ischemic heart disease, n(%)	No	24888 (70.7%)	8.5	19205 (71.2%)	
Amputation	80	Ischemic heart disease, n(%)	Yes	10334 (29.3%)	8.5	7775 (28.8%)	
Amputation	80	Neuropathy, n(%)	No	26681 (69.3%)	0	16994 (63%)	
Amputation	80	Neuropathy, n(%)	Yes	11818 (30.7%)	0	9986 (37%)	
Amputation	80	Self-reported physical activity, n(%)	Never	5730 (17.1%)	12.8	4425 (16.4%)	
Amputation	80	Self-reported physical activity, n(%)	<1 times/week	4452 (13.3%)	12.8	3442 (12.8%)	
Amputation	80	Self-reported physical activity, n(%)	regularly - 1-2 times/week	6399 (19.1%)	12.8	5120 (19%)	
Amputation	80	Self-reported physical activity, n(%)	regularly - 3-5 times/week	6873 (20.5%)	12.8	5645 (20.9%)	
Amputation	80	Self-reported physical activity, n(%)	Daily	10115 (30.1%)	12.8	8348 (30.9%)	
Amputation	80	Sex, n(%)	Males	19335 (50.2%)	0	13768 (51%)	n=38499 vs n=26980
Amputation	80	Sex, n(%)	Females	19161 (49.8%)	0	13212 (49%)	
Amputation	80	Smoking status, n(%)	No	33104 (93.3%)	7.8	25239 (93.5%)	
Amputation	80	Smoking status, n(%)	Yes	2376 (6.7%)	7.8	1741 (6.5%)	

Amputation	80	Stroke, n(%)	No	30520 (87.3%)	9.2	23714 (87.9%)	
Amputation	80	Stroke, n(%)	Yes	4429 (12.7%)	9.2	3266 (12.1%)	
Amputation	80	Systolic blood pressure, mean(SD)		136.5 (16.2)	2.7	136.2 (15.9)	
Amputation	80	Time since last healthcare visit, mean(SD)		0.8 (1.3)	0	0.6 (1)	
Amputation	80	Total cholesterol, mean(SD)		4.6 (1.1)	10.8	4.6 (1.1)	
Amputation	81	Age at diagnosis of type 2 diabetes, mean(SD)		76.2 (3)	0	75.5 (3)	
Amputation	81	BMI, mean(SD)		28.5 (4.8)	10.1	28.5 (4.8)	
Amputation	81	HbA1c, mean(SD)		49.1 (10.4)	2.2	49.2 (10.1)	
Amputation	81	HDL-cholesterol, mean(SD)		1.3 (0.4)	16.8	1.3 (0.4)	
Amputation	81	Ischemic heart disease, n(%)	No	22643 (69.7%)	8.7	17184 (70.4%)	
Amputation	81	Ischemic heart disease, n(%)	Yes	9823 (30.3%)	8.7	7232 (29.6%)	
Amputation	81	Neuropathy, n(%)	No	24361 (68.5%)	0	15015 (61.5%)	
Amputation	81	Neuropathy, n(%)	Yes	11211 (31.5%)	0	9401 (38.5%)	
Amputation	81	Self-reported physical activity, n(%)	Never	5442 (17.7%)	13.6	4134 (16.9%)	
Amputation	81	Self-reported physical activity, n(%)	<1 times/week	4242 (13.8%)	13.6	3225 (13.2%)	
Amputation	81	Self-reported physical activity, n(%)	regularly - 1-2 times/week	5931 (19.3%)	13.6	4711 (19.3%)	
Amputation	81	Self-reported physical activity, n(%)	regularly - 3-5 times/week	6048 (19.7%)	13.6	4937 (20.2%)	
Amputation	81	Self-reported physical activity, n(%)	Daily	9082 (29.5%)	13.6	7409 (30.3%)	
Amputation	81	Sex, n(%)	Males	17492 (49.2%)	0	12261 (50.2%)	n=35572 vs n=24416
Amputation	81	Sex, n(%)	Females	18075 (50.8%)	0	12155 (49.8%)	
Amputation	81	Smoking status, n(%)	No	30595 (93.8%)	8.3	22973 (94.1%)	
Amputation	81	Smoking status, n(%)	Yes	2016 (6.2%)	8.3	1443 (5.9%)	
Amputation	81	Stroke, n(%)	No	27880 (86.7%)	9.6	21276 (87.1%)	

Amputation	81	Stroke, n(%)	Yes	4282 (13.3%)	9.6	3140 (12.9%)	
Amputation	81	Systolic blood pressure, mean(SD)		136.6 (16.4)	2.7	136.3 (16.1)	
Amputation	81	Time since last healthcare visit, mean(SD)		0.8 (1.3)	0	0.6 (1)	
Amputation	81	Total cholesterol, mean(SD)		4.7 (1.1)	11.7	4.6 (1.1)	
Amputation	82	Age at diagnosis of type 2 diabetes, mean(SD)		77.1 (3)	0	76.4 (3)	
Amputation	82	BMI, mean(SD)		28.4 (4.8)	10.4	28.4 (4.7)	
Amputation	82	HbA1c, mean(SD)		49.3 (10.5)	2.1	49.5 (10.2)	
Amputation	82	HDL-cholesterol, mean(SD)		1.3 (0.4)	17.8	1.3 (0.4)	
Amputation	82	Ischemic heart disease, n(%)	No	20420 (68.9%)	8.9	15274 (69.4%)	
Amputation	82	Ischemic heart disease, n(%)	Yes	9220 (31.1%)	8.9	6733 (30.6%)	
Amputation	82	Neuropathy, n(%)	No	21978 (67.6%)	0	13292 (60.4%)	
Amputation	82	Neuropathy, n(%)	Yes	10548 (32.4%)	0	8715 (39.6%)	
Amputation	82	Self-reported physical activity, n(%)	Never	5282 (18.9%)	14	3950 (17.9%)	
Amputation	82	Self-reported physical activity, n(%)	<1 times/week	3898 (13.9%)	14	2954 (13.4%)	
Amputation	82	Self-reported physical activity, n(%)	regularly - 1-2 times/week	5397 (19.3%)	14	4242 (19.3%)	
Amputation	82	Self-reported physical activity, n(%)	regularly - 3-5 times/week	5473 (19.6%)	14	4472 (20.3%)	
Amputation	82	Self-reported physical activity, n(%)	Daily	7923 (28.3%)	14	6389 (29%)	
Amputation	82	Sex, n(%)	Males	15688 (48.2%)	0	10821 (49.2%)	n=32526 vs n=22007
Amputation	82	Sex, n(%)	Females	16833 (51.8%)	0	11186 (50.8%)	
Amputation	82	Smoking status, n(%)	No	28062 (94.4%)	8.6	20866 (94.8%)	
Amputation	82	Smoking status, n(%)	Yes	1657 (5.6%)	8.6	1141 (5.2%)	
Amputation	82	Stroke, n(%)	No	25259 (86.1%)	9.8	19032 (86.5%)	
Amputation	82	Stroke, n(%)	Yes	4080 (13.9%)	9.8	2975 (13.5%)	

Amputation	82	Systolic blood pressure, mean(SD)		136.8 (16.6)	2.9	136.4 (16.3)	
Amputation	82	Time since last healthcare visit, mean(SD)		0.8 (1.3)	0	0.7 (1)	
Amputation	82	Total cholesterol, mean(SD)		4.7 (1.1)	12.5	4.6 (1.1)	
Amputation	83	Age at diagnosis of type 2 diabetes, mean(SD)		78.1 (3)	0	77.3 (3)	
Amputation	83	BMI, mean(SD)		28.2 (4.7)	10.8	28.2 (4.7)	
Amputation	83	HbA1c, mean(SD)		49.4 (10.5)	2.1	49.6 (10.3)	
Amputation	83	HDL-cholesterol, mean(SD)		1.3 (0.4)	18.9	1.3 (0.4)	
Amputation	83	Ischemic heart disease, n(%)	No	18281 (67.9%)	9	13508 (68.5%)	
Amputation	83	Ischemic heart disease, n(%)	Yes	8642 (32.1%)	9	6205 (31.5%)	
Amputation	83	Neuropathy, n(%)	No	19682 (66.5%)	0	11599 (58.8%)	
Amputation	83	Neuropathy, n(%)	Yes	9909 (33.5%)	0	8114 (41.2%)	
Amputation	83	Self-reported physical activity, n(%)	Never	5037 (19.9%)	14.6	3749 (19%)	
Amputation	83	Self-reported physical activity, n(%)	<1 times/week	3504 (13.9%)	14.6	2629 (13.3%)	
Amputation	83	Self-reported physical activity, n(%)	regularly - 1-2 times/week	5025 (19.9%)	14.6	3942 (20%)	
Amputation	83	Self-reported physical activity, n(%)	regularly - 3-5 times/week	4751 (18.8%)	14.6	3805 (19.3%)	
Amputation	83	Self-reported physical activity, n(%)	Daily	6947 (27.5%)	14.6	5588 (28.3%)	
Amputation	83	Sex, n(%)	Males	13949 (47.1%)	0	9446 (47.9%)	n=29591 vs n=19713
Amputation	83	Sex, n(%)	Females	15638 (52.9%)	0	10267 (52.1%)	
Amputation	83	Smoking status, n(%)	No	25522 (94.7%)	8.9	18733 (95%)	
Amputation	83	Smoking status, n(%)	Yes	1429 (5.3%)	8.9	980 (5%)	
Amputation	83	Stroke, n(%)	No	22789 (85.5%)	9.9	16909 (85.8%)	
Amputation	83	Stroke, n(%)	Yes	3870 (14.5%)	9.9	2804 (14.2%)	
Amputation	83	Systolic blood pressure, mean(SD)		136.8 (16.8)	2.8	136.3 (16.5)	

Amputation	83	Time since last healthcare visit, mean(SD)		0.9 (1.3)	0	0.7 (1.1)	
Amputation	83	Total cholesterol, mean(SD)		4.7 (1.1)	13.3	4.6 (1.1)	
Amputation	84	Age at diagnosis of type 2 diabetes, mean(SD)		79.1 (3.1)	0	78.2 (3)	
Amputation	84	BMI, mean(SD)		28 (4.6)	11.7	28.1 (4.6)	
Amputation	84	HbA1c, mean(SD)		49.6 (10.7)	2.6	49.7 (10.4)	
Amputation	84	HDL-cholesterol, mean(SD)		1.3 (0.4)	20.1	1.3 (0.4)	
Amputation	84	Ischemic heart disease, n(%)	No	16143 (66.7%)	9.2	11655 (67.1%)	
Amputation	84	Ischemic heart disease, n(%)	Yes	8044 (33.3%)	9.2	5707 (32.9%)	
Amputation	84	Neuropathy, n(%)	No	17496 (65.7%)	0	9921 (57.1%)	
Amputation	84	Neuropathy, n(%)	Yes	9152 (34.3%)	0	7441 (42.9%)	
Amputation	84	Self-reported physical activity, n(%)	Never	4837 (21.5%)	15.5	3576 (20.6%)	
Amputation	84	Self-reported physical activity, n(%)	<1 times/week	3268 (14.5%)	15.5	2420 (13.9%)	
Amputation	84	Self-reported physical activity, n(%)	regularly - 1-2 times/week	4359 (19.4%)	15.5	3358 (19.3%)	
Amputation	84	Self-reported physical activity, n(%)	regularly - 3-5 times/week	4154 (18.4%)	15.5	3306 (19%)	
Amputation	84	Self-reported physical activity, n(%)	Daily	5902 (26.2%)	15.5	4702 (27.1%)	
Amputation	84	Sex, n(%)	Males	12231 (45.9%)	0	8114 (46.7%)	n=26648 vs n=17362
Amputation	84	Sex, n(%)	Females	14411 (54.1%)	0	9248 (53.3%)	
Amputation	84	Smoking status, n(%)	No	22890 (95.1%)	9.7	16580 (95.5%)	
Amputation	84	Smoking status, n(%)	Yes	1169 (4.9%)	9.7	782 (4.5%)	
Amputation	84	Stroke, n(%)	No	20374 (85.1%)	10.2	14848 (85.5%)	
Amputation	84	Stroke, n(%)	Yes	3569 (14.9%)	10.2	2514 (14.5%)	
Amputation	84	Systolic blood pressure, mean(SD)		136.9 (17)	3.1	136.5 (16.7)	
Amputation	84	Time since last healthcare visit, mean(SD)		0.9 (1.4)	0	0.7 (1.1)	

Amputation	84	Total cholesterol, mean(SD)		4.7 (1.1)	14.5	4.6 (1.1)	
Amputation	85	Age at diagnosis of type 2 diabetes, mean(SD)		80 (3.1)	0	79.2 (3)	
Amputation	85	BMI, mean(SD)		27.9 (4.6)	12.4	27.9 (4.6)	
Amputation	85	HbA1c, mean(SD)		49.6 (10.6)	2.7	49.7 (10.2)	
Amputation	85	HDL-cholesterol, mean(SD)		1.3 (0.4)	21.3	1.3 (0.4)	
Amputation	85	Ischemic heart disease, n(%)	No	14193 (65.8%)	9.6	10043 (66.1%)	
Amputation	85	Ischemic heart disease, n(%)	Yes	7381 (34.2%)	9.6	5149 (33.9%)	
Amputation	85	Neuropathy, n(%)	No	15453 (64.8%)	0	8478 (55.8%)	
Amputation	85	Neuropathy, n(%)	Yes	8406 (35.2%)	0	6714 (44.2%)	
Amputation	85	Self-reported physical activity, n(%)	Never	4461 (22.4%)	16.4	3268 (21.5%)	
Amputation	85	Self-reported physical activity, n(%)	<1 times/week	2979 (14.9%)	16.4	2185 (14.4%)	
Amputation	85	Self-reported physical activity, n(%)	regularly - 1-2 times/week	3915 (19.6%)	16.4	2997 (19.7%)	
Amputation	85	Self-reported physical activity, n(%)	regularly - 3-5 times/week	3533 (17.7%)	16.4	2790 (18.4%)	
Amputation	85	Self-reported physical activity, n(%)	Daily	5066 (25.4%)	16.4	3952 (26%)	
Amputation	85	Sex, n(%)	Males	10664 (44.7%)	0	6943 (45.7%)	n=23859 vs n=15192
Amputation	85	Sex, n(%)	Females	13189 (55.3%)	0	8249 (54.3%)	
Amputation	85	Smoking status, n(%)	No	20459 (95.6%)	10.3	14579 (96%)	
Amputation	85	Smoking status, n(%)	Yes	939 (4.4%)	10.3	613 (4%)	
Amputation	85	Stroke, n(%)	No	18073 (84.6%)	10.5	12910 (85%)	
Amputation	85	Stroke, n(%)	Yes	3291 (15.4%)	10.5	2282 (15%)	
Amputation	85	Systolic blood pressure, mean(SD)		137.1 (17.1)	3	136.8 (16.8)	
Amputation	85	Time since last healthcare visit, mean(SD)		0.9 (1.4)	0	0.7 (1.1)	
Amputation	85	Total cholesterol, mean(SD)		4.7 (1.2)	15.7	4.7 (1.1)	

Amputation	86	Age at diagnosis of type 2 diabetes, mean(SD)		81 (3.1)	0	80.1 (3)	
Amputation	86	BMI, mean(SD)		27.7 (4.6)	13.2	27.7 (4.6)	
Amputation	86	HbA1c, mean(SD)		49.8 (10.9)	2.8	49.9 (10.6)	
Amputation	86	HDL-cholesterol, mean(SD)		1.3 (0.4)	22.9	1.3 (0.4)	
Amputation	86	Ischemic heart disease, n(%)	No	12409 (65.3%)	9.8	8578 (65.5%)	
Amputation	86	Ischemic heart disease, n(%)	Yes	6608 (34.7%)	9.8	4524 (34.5%)	
Amputation	86	Neuropathy, n(%)	No	13511 (64.1%)	0	7113 (54.3%)	
Amputation	86	Neuropathy, n(%)	Yes	7570 (35.9%)	0	5989 (45.7%)	
Amputation	86	Self-reported physical activity, n(%)	Never	4050 (23.2%)	17.2	2900 (22.1%)	
Amputation	86	Self-reported physical activity, n(%)	<1 times/week	2628 (15.1%)	17.2	1912 (14.6%)	
Amputation	86	Self-reported physical activity, n(%)	regularly - 1-2 times/week	3414 (19.6%)	17.2	2570 (19.6%)	
Amputation	86	Self-reported physical activity, n(%)	regularly - 3-5 times/week	3042 (17.4%)	17.2	2369 (18.1%)	
Amputation	86	Self-reported physical activity, n(%)	Daily	4325 (24.8%)	17.2	3351 (25.6%)	
Amputation	86	Sex, n(%)	Males	9088 (43.1%)	0	5815 (44.4%)	n=21081 vs n=13102
Amputation	86	Sex, n(%)	Females	11990 (56.9%)	0	7287 (55.6%)	
Amputation	86	Smoking status, n(%)	No	18041 (95.9%)	10.8	12608 (96.2%)	
Amputation	86	Smoking status, n(%)	Yes	772 (4.1%)	10.8	494 (3.8%)	
Amputation	86	Stroke, n(%)	No	15885 (84.4%)	10.7	11070 (84.5%)	
Amputation	86	Stroke, n(%)	Yes	2944 (15.6%)	10.7	2032 (15.5%)	
Amputation	86	Systolic blood pressure, mean(SD)		137.2 (17.3)	3.3	136.9 (17.1)	
Amputation	86	Time since last healthcare visit, mean(SD)		1 (1.5)	0	0.8 (1.2)	
Amputation	86	Total cholesterol, mean(SD)		4.7 (1.1)	17	4.7 (1.1)	
Amputation	87	Age at diagnosis of type 2 diabetes, mean(SD)		82 (3.1)	0	81 (3)	

Amputation	87	BMI, mean(SD)		27.5 (4.5)	14.2	27.5 (4.4)	
Amputation	87	HbA1c, mean(SD)		49.9 (10.9)	3	50 (10.6)	
Amputation	87	HDL-cholesterol, mean(SD)		1.3 (0.4)	25	1.4 (0.4)	
Amputation	87	Ischemic heart disease, n(%)	No	10551 (64.5%)	10.4	7151 (65%)	
Amputation	87	Ischemic heart disease, n(%)	Yes	5817 (35.5%)	10.4	3846 (35%)	
Amputation	87	Neuropathy, n(%)	No	11601 (63.5%)	0	5812 (52.9%)	
Amputation	87	Neuropathy, n(%)	Yes	6660 (36.5%)	0	5185 (47.1%)	
Amputation	87	Self-reported physical activity, n(%)	Never	3631 (24.4%)	18.6	2558 (23.3%)	
Amputation	87	Self-reported physical activity, n(%)	<1 times/week	2262 (15.2%)	18.6	1604 (14.6%)	
Amputation	87	Self-reported physical activity, n(%)	regularly - 1-2 times/week	2985 (20.1%)	18.6	2199 (20%)	
Amputation	87	Self-reported physical activity, n(%)	regularly - 3-5 times/week	2563 (17.2%)	18.6	1990 (18.1%)	
Amputation	87	Self-reported physical activity, n(%)	Daily	3420 (23%)	18.6	2646 (24.1%)	
Amputation	87	Sex, n(%)	Males	7640 (41.8%)	0	4727 (43%)	n=18261 vs n=10997
Amputation	87	Sex, n(%)	Females	10617 (58.2%)	0	6270 (57%)	
Amputation	87	Smoking status, n(%)	No	15553 (96.4%)	11.6	10643 (96.8%)	
Amputation	87	Smoking status, n(%)	Yes	587 (3.6%)	11.6	354 (3.2%)	
Amputation	87	Stroke, n(%)	No	13588 (83.9%)	11.3	9290 (84.5%)	
Amputation	87	Stroke, n(%)	Yes	2604 (16.1%)	11.3	1707 (15.5%)	
Amputation	87	Systolic blood pressure, mean(SD)		137.3 (17.5)	3.6	136.9 (17.1)	
Amputation	87	Time since last healthcare visit, mean(SD)		1 (1.5)	0	0.8 (1.2)	
Amputation	87	Total cholesterol, mean(SD)		4.8 (1.2)	18.9	4.7 (1.2)	
Amputation	88	Age at diagnosis of type 2 diabetes, mean(SD)		82.9 (3.1)	0	81.9 (3)	
Amputation	88	BMI, mean(SD)		27.3 (4.5)	15.3	27.3 (4.4)	

Amputation	88	HbA1c, mean(SD)		50 (11)	3	50.2 (10.7)	
Amputation	88	HDL-cholesterol, mean(SD)		1.3 (0.4)	26.7	1.4 (0.4)	
Amputation	88	Ischemic heart disease, n(%)	No	9008 (64%)	10.5	5873 (64.2%)	
Amputation	88	Ischemic heart disease, n(%)	Yes	5065 (36%)	10.5	3272 (35.8%)	
Amputation	88	Neuropathy, n(%)	No	9960 (63.4%)	0	4733 (51.8%)	
Amputation	88	Neuropathy, n(%)	Yes	5758 (36.6%)	0	4412 (48.2%)	
Amputation	88	Self-reported physical activity, n(%)	Never	3294 (26.1%)	19.7	2243 (24.5%)	
Amputation	88	Self-reported physical activity, n(%)	<1 times/week	2035 (16.1%)	19.7	1439 (15.7%)	
Amputation	88	Self-reported physical activity, n(%)	regularly - 1-2 times/week	2421 (19.2%)	19.7	1794 (19.6%)	
Amputation	88	Self-reported physical activity, n(%)	regularly - 3-5 times/week	2009 (15.9%)	19.7	1523 (16.7%)	
Amputation	88	Self-reported physical activity, n(%)	Daily	2858 (22.7%)	19.7	2146 (23.5%)	
Amputation	88	Sex, n(%)	Males	6404 (40.8%)	0	3873 (42.4%)	n=15718 vs n=9145
Amputation	88	Sex, n(%)	Females	9310 (59.2%)	0	5272 (57.6%)	
Amputation	88	Smoking status, n(%)	No	13318 (96.7%)	12.4	8873 (97%)	
Amputation	88	Smoking status, n(%)	Yes	454 (3.3%)	12.4	272 (3%)	
Amputation	88	Stroke, n(%)	No	11616 (83.5%)	11.5	7696 (84.2%)	
Amputation	88	Stroke, n(%)	Yes	2294 (16.5%)	11.5	1449 (15.8%)	
Amputation	88	Systolic blood pressure, mean(SD)		137.4 (17.6)	3.9	136.8 (17.1)	
Amputation	88	Time since last healthcare visit, mean(SD)		1.1 (1.6)	0	0.8 (1.3)	
Amputation	88	Total cholesterol, mean(SD)		4.8 (1.2)	20.5	4.7 (1.2)	
Amputation	89	Age at diagnosis of type 2 diabetes, mean(SD)		83.8 (3.1)	0	82.7 (2.9)	
Amputation	89	BMI, mean(SD)		27.1 (4.5)	16.6	27 (4.3)	
Amputation	89	HbA1c, mean(SD)		50.2 (11.1)	3.2	50.2 (10.5)	

Amputation	89	HDL-cholesterol, mean(SD)		1.3 (0.4)	28.7	1.4 (0.4)	
Amputation	89	Ischemic heart disease, n(%)	No	7336 (63.3%)	10.9	4620 (63.2%)	
Amputation	89	Ischemic heart disease, n(%)	Yes	4256 (36.7%)	10.9	2686 (36.8%)	
Amputation	89	Neuropathy, n(%)	No	8208 (63.1%)	0	3674 (50.3%)	
Amputation	89	Neuropathy, n(%)	Yes	4795 (36.9%)	0	3632 (49.7%)	
Amputation	89	Self-reported physical activity, n(%)	Never	2789 (27.1%)	20.9	1838 (25.2%)	
Amputation	89	Self-reported physical activity, n(%)	<1 times/week	1711 (16.6%)	20.9	1198 (16.4%)	
Amputation	89	Self-reported physical activity, n(%)	regularly - 1-2 times/week	1929 (18.8%)	20.9	1379 (18.9%)	
Amputation	89	Self-reported physical activity, n(%)	regularly - 3-5 times/week	1605 (15.6%)	20.9	1208 (16.5%)	
Amputation	89	Self-reported physical activity, n(%)	Daily	2252 (21.9%)	20.9	1683 (23%)	
Amputation	89	Sex, n(%)	Males	5131 (39.5%)	0	2997 (41%)	n=13003 vs n=7306
Amputation	89	Sex, n(%)	Females	7869 (60.5%)	0	4309 (59%)	
Amputation	89	Smoking status, n(%)	No	10926 (96.9%)	13.3	7100 (97.2%)	
Amputation	89	Smoking status, n(%)	Yes	345 (3.1%)	13.3	206 (2.8%)	
Amputation	89	Stroke, n(%)	No	9654 (84.3%)	11.9	6196 (84.8%)	
Amputation	89	Stroke, n(%)	Yes	1796 (15.7%)	11.9	1110 (15.2%)	
Amputation	89	Systolic blood pressure, mean(SD)		137.4 (17.8)	4.1	137.1 (17.5)	
Amputation	89	Time since last healthcare visit, mean(SD)		1.1 (1.6)	0	0.9 (1.3)	
Amputation	89	Total cholesterol, mean(SD)		4.8 (1.2)	22.3	4.8 (1.2)	
Amputation	90	Age at diagnosis of type 2 diabetes, mean(SD)		84.9 (3.1)	0	83.7 (2.9)	
Amputation	90	BMI, mean(SD)		26.8 (4.3)	17.8	26.8 (4.3)	
Amputation	90	HbA1c, mean(SD)		50.3 (11.2)	3.5	50.4 (10.8)	
Amputation	90	HDL-cholesterol, mean(SD)		1.4 (0.4)	30.6	1.4 (0.4)	

Amputation	90	Ischemic heart disease, n(%)	No	5875 (62.9%)	10.8	3552 (62.7%)	
Amputation	90	Ischemic heart disease, n(%)	Yes	3463 (37.1%)	10.8	2110 (37.3%)	
Amputation	90	Neuropathy, n(%)	No	6690 (63.9%)	0	2834 (50.1%)	
Amputation	90	Neuropathy, n(%)	Yes	3775 (36.1%)	0	2828 (49.9%)	
Amputation	90	Self-reported physical activity, n(%)	Never	2278 (28%)	22.2	1484 (26.2%)	
Amputation	90	Self-reported physical activity, n(%)	<1 times/week	1374 (16.9%)	22.2	936 (16.5%)	
Amputation	90	Self-reported physical activity, n(%)	regularly - 1-2 times/week	1529 (18.8%)	22.2	1052 (18.6%)	
Amputation	90	Self-reported physical activity, n(%)	regularly - 3-5 times/week	1202 (14.8%)	22.2	875 (15.5%)	
Amputation	90	Self-reported physical activity, n(%)	Daily	1758 (21.6%)	22.2	1315 (23.2%)	
Amputation	90	Sex, n(%)	Males	3987 (38.1%)	0	2296 (40.6%)	n=10465 vs n=5662
Amputation	90	Sex, n(%)	Females	6474 (61.9%)	0	3366 (59.4%)	
Amputation	90	Smoking status, n(%)	No	8690 (97.3%)	14.6	5528 (97.6%)	
Amputation	90	Smoking status, n(%)	Yes	245 (2.7%)	14.6	134 (2.4%)	
Amputation	90	Stroke, n(%)	No	7788 (84.7%)	12.1	4833 (85.4%)	
Amputation	90	Stroke, n(%)	Yes	1408 (15.3%)	12.1	829 (14.6%)	
Amputation	90	Systolic blood pressure, mean(SD)		137.9 (17.8)	4.2	137.8 (17.5)	
Amputation	90	Time since last healthcare visit, mean(SD)		1.2 (1.6)	0	0.9 (1.3)	
Amputation	90	Total cholesterol, mean(SD)		4.8 (1.2)	24.5	4.8 (1.2)	
Amputation	91	Age at diagnosis of type 2 diabetes, mean(SD)		85.7 (3.1)	0	84.6 (2.9)	
Amputation	91	BMI, mean(SD)		26.6 (4.3)	18.9	26.6 (4.2)	
Amputation	91	HbA1c, mean(SD)		50.6 (11.6)	3.9	50.7 (11)	
Amputation	91	HDL-cholesterol, mean(SD)		1.4 (0.4)	32.4	1.3 (0.4)	
Amputation	91	Ischemic heart disease, n(%)	No	4653 (62.7%)	10.6	2713 (62.5%)	

Amputation	91	Ischemic heart disease, n(%)	Yes	2773 (37.3%)	10.6	1628 (37.5%)	
Amputation	91	Neuropathy, n(%)	No	5342 (64.3%)	0	2158 (49.7%)	
Amputation	91	Neuropathy, n(%)	Yes	2961 (35.7%)	0	2183 (50.3%)	
Amputation	91	Self-reported physical activity, n(%)	Never	1873 (29.6%)	23.9	1191 (27.4%)	
Amputation	91	Self-reported physical activity, n(%)	<1 times/week	1039 (16.4%)	23.9	709 (16.3%)	
Amputation	91	Self-reported physical activity, n(%)	regularly - 1-2 times/week	1187 (18.8%)	23.9	821 (18.9%)	
Amputation	91	Self-reported physical activity, n(%)	regularly - 3-5 times/week	895 (14.2%)	23.9	651 (15%)	
Amputation	91	Self-reported physical activity, n(%)	Daily	1326 (21%)	23.9	969 (22.3%)	
Amputation	91	Sex, n(%)	Males	3033 (36.5%)	0	1737 (40%)	n=8303 vs n=4341
Amputation	91	Sex, n(%)	Females	5268 (63.5%)	0	2604 (60%)	
Amputation	91	Smoking status, n(%)	No	6804 (97.5%)	15.9	4242 (97.7%)	
Amputation	91	Smoking status, n(%)	Yes	175 (2.5%)	15.9	99 (2.3%)	
Amputation	91	Stroke, n(%)	No	6161 (84.4%)	12	3688 (85%)	
Amputation	91	Stroke, n(%)	Yes	1142 (15.6%)	12	653 (15%)	
Amputation	91	Systolic blood pressure, mean(SD)		137.8 (18)	4.4	137.7 (17.6)	
Amputation	91	Time since last healthcare visit, mean(SD)		1.2 (1.7)	0	1 (1.4)	
Amputation	91	Total cholesterol, mean(SD)		4.9 (1.2)	26.2	4.8 (1.2)	
Amputation	92	Age at diagnosis of type 2 diabetes, mean(SD)		86.7 (3.1)	0	85.4 (2.9)	
Amputation	92	BMI, mean(SD)		26.5 (4.3)	20.8	26.4 (4.3)	
Amputation	92	HbA1c, mean(SD)		50.6 (11.5)	4.1	50.7 (11.3)	
Amputation	92	HDL-cholesterol, mean(SD)		1.4 (0.4)	35	1.4 (0.4)	
Amputation	92	Ischemic heart disease, n(%)	No	3507 (62.5%)	11.6	1977 (63.2%)	
Amputation	92	Ischemic heart disease, n(%)	Yes	2106 (37.5%)	11.6	1151 (36.8%)	

Amputation	92	Neuropathy, n(%)	No	4153 (65.4%)	0	1534 (49%)	
Amputation	92	Neuropathy, n(%)	Yes	2199 (34.6%)	0	1594 (51%)	
Amputation	92	Self-reported physical activity, n(%)	Never	1437 (30.7%)	26.3	886 (28.3%)	
Amputation	92	Self-reported physical activity, n(%)	<1 times/week	775 (16.5%)	26.3	510 (16.3%)	
Amputation	92	Self-reported physical activity, n(%)	regularly - 1-2 times/week	880 (18.8%)	26.3	591 (18.9%)	
Amputation	92	Self-reported physical activity, n(%)	regularly - 3-5 times/week	631 (13.5%)	26.3	448 (14.3%)	
Amputation	92	Self-reported physical activity, n(%)	Daily	960 (20.5%)	26.3	693 (22.2%)	
Amputation	92	Sex, n(%)	Males	2269 (35.7%)	0	1235 (39.5%)	n=6352 vs n=3128
Amputation	92	Sex, n(%)	Females	4081 (64.3%)	0	1893 (60.5%)	
Amputation	92	Smoking status, n(%)	No	5118 (97.8%)	17.6	3071 (98.2%)	
Amputation	92	Smoking status, n(%)	Yes	115 (2.2%)	17.6	57 (1.8%)	
Amputation	92	Stroke, n(%)	No	4653 (84.4%)	13.2	2650 (84.7%)	
Amputation	92	Stroke, n(%)	Yes	863 (15.6%)	13.2	478 (15.3%)	
Amputation	92	Systolic blood pressure, mean(SD)		137.9 (18)	5.1	137.4 (17.5)	
Amputation	92	Time since last healthcare visit, mean(SD)		1.3 (1.7)	0	1 (1.5)	
Amputation	92	Total cholesterol, mean(SD)		4.9 (1.2)	28.7	4.9 (1.2)	
Amputation	93	Age at diagnosis of type 2 diabetes, mean(SD)		87.6 (3.1)	0	86.3 (2.8)	
Amputation	93	BMI, mean(SD)		26.3 (4.3)	22.4	26.2 (4.2)	
Amputation	93	HbA1c, mean(SD)		50.6 (11.5)	5	50.8 (11)	
Amputation	93	HDL-cholesterol, mean(SD)		1.4 (0.4)	37.1	1.4 (0.4)	
Amputation	93	Ischemic heart disease, n(%)	No	2617 (63%)	12.1	1421 (64.1%)	
Amputation	93	Ischemic heart disease, n(%)	Yes	1538 (37%)	12.1	795 (35.9%)	
Amputation	93	Neuropathy, n(%)	No	3080 (65.1%)	0	1052 (47.5%)	

Amputation	93	Neuropathy, n(%)	Yes	1649 (34.9%)	0	1164 (52.5%)	
Amputation	93	Self-reported physical activity, n(%)	Never	1087 (32.1%)	28.3	632 (28.5%)	
Amputation	93	Self-reported physical activity, n(%)	<1 times/week	530 (15.6%)	28.3	364 (16.4%)	
Amputation	93	Self-reported physical activity, n(%)	regularly - 1-2 times/week	618 (18.2%)	28.3	399 (18%)	
Amputation	93	Self-reported physical activity, n(%)	regularly - 3-5 times/week	465 (13.7%)	28.3	322 (14.5%)	
Amputation	93	Self-reported physical activity, n(%)	Daily	691 (20.4%)	28.3	499 (22.5%)	
Amputation	93	Sex, n(%)	Males	1653 (35%)	0	853 (38.5%)	n=4729 vs n=2216
Amputation	93	Sex, n(%)	Females	3075 (65%)	0	1363 (61.5%)	
Amputation	93	Smoking status, n(%)	No	3776 (98%)	18.5	2181 (98.4%)	
Amputation	93	Smoking status, n(%)	Yes	76 (2%)	18.5	35 (1.6%)	
Amputation	93	Stroke, n(%)	No	3459 (84.9%)	13.9	1900 (85.7%)	
Amputation	93	Stroke, n(%)	Yes	614 (15.1%)	13.9	316 (14.3%)	
Amputation	93	Systolic blood pressure, mean(SD)		137.9 (18.5)	5.8	137.3 (18.2)	
Amputation	93	Time since last healthcare visit, mean(SD)		1.4 (1.8)	0	1.1 (1.5)	
Amputation	93	Total cholesterol, mean(SD)		4.9 (1.2)	30.7	4.9 (1.2)	
Amputation	94	Age at diagnosis of type 2 diabetes, mean(SD)		88.6 (3.1)	0	87.2 (2.8)	
Amputation	94	BMI, mean(SD)		26 (4.2)	24.5	26 (4.1)	
Amputation	94	HbA1c, mean(SD)		50.7 (11.4)	5.3	50.9 (11.4)	
Amputation	94	HDL-cholesterol, mean(SD)		1.4 (0.4)	39.8	1.4 (0.4)	
Amputation	94	Ischemic heart disease, n(%)	No	1889 (62.8%)	12.8	959 (63.2%)	
Amputation	94	Ischemic heart disease, n(%)	Yes	1117 (37.2%)	12.8	558 (36.8%)	
Amputation	94	Neuropathy, n(%)	No	2258 (65.5%)	0	719 (47.4%)	
Amputation	94	Neuropathy, n(%)	Yes	1188 (34.5%)	0	798 (52.6%)	

Amputation	94	Self-reported physical activity, n(%)	Never	797 (33.2%)	30.4	439 (28.9%)	
Amputation	94	Self-reported physical activity, n(%)	<1 times/week	396 (16.5%)	30.4	258 (17%)	
Amputation	94	Self-reported physical activity, n(%)	regularly - 1-2 times/week	430 (17.9%)	30.4	284 (18.7%)	
Amputation	94	Self-reported physical activity, n(%)	regularly - 3-5 times/week	317 (13.2%)	30.4	220 (14.5%)	
Amputation	94	Self-reported physical activity, n(%)	Daily	459 (19.1%)	30.4	316 (20.8%)	
Amputation	94	Sex, n(%)	Males	1119 (32.5%)	0	549 (36.2%)	n=3446 vs n=1517
Amputation	94	Sex, n(%)	Females	2327 (67.5%)	0	968 (63.8%)	
Amputation	94	Smoking status, n(%)	No	2698 (97.8%)	20	1488 (98.1%)	
Amputation	94	Smoking status, n(%)	Yes	60 (2.2%)	20	29 (1.9%)	
Amputation	94	Stroke, n(%)	No	2486 (84.4%)	14.5	1305 (86%)	
Amputation	94	Stroke, n(%)	Yes	459 (15.6%)	14.5	212 (14%)	
Amputation	94	Systolic blood pressure, mean(SD)		138.3 (18.1)	6	137.6 (17.7)	
Amputation	94	Time since last healthcare visit, mean(SD)		1.5 (1.9)	0	1.2 (1.6)	
Amputation	94	Total cholesterol, mean(SD)		4.9 (1.2)	33.3	4.9 (1.2)	
Amputation	95	Age at diagnosis of type 2 diabetes, mean(SD)		89.5 (3.1)	0	87.9 (2.7)	
Amputation	95	BMI, mean(SD)		25.9 (4.2)	26.1	25.8 (4.1)	
Amputation	95	HbA1c, mean(SD)		50.7 (11.4)	6.4	50.6 (11.2)	
Amputation	95	HDL-cholesterol, mean(SD)		1.4 (0.4)	43.4	1.4 (0.4)	
Amputation	95	Ischemic heart disease, n(%)	No	1353 (64%)	13.5	637 (64.1%)	
Amputation	95	Ischemic heart disease, n(%)	Yes	762 (36%)	13.5	357 (35.9%)	
Amputation	95	Neuropathy, n(%)	No	1641 (67.1%)	0	472 (47.5%)	
Amputation	95	Neuropathy, n(%)	Yes	803 (32.9%)	0	522 (52.5%)	
Amputation	95	Self-reported physical activity, n(%)	Never	578 (35.3%)	33.1	297 (29.9%)	

Amputation	95	Self-reported physical activity, n(%)	<1 times/week	236 (14.4%)	33.1	143 (14.4%)	
Amputation	95	Self-reported physical activity, n(%)	regularly - 1-2 times/week	278 (17%)	33.1	172 (17.3%)	
Amputation	95	Self-reported physical activity, n(%)	regularly - 3-5 times/week	226 (13.8%)	33.1	154 (15.5%)	
Amputation	95	Self-reported physical activity, n(%)	Daily	318 (19.4%)	33.1	228 (22.9%)	
Amputation	95	Sex, n(%)	Males	729 (29.8%)	0	333 (33.5%)	n=2444 vs n=994
Amputation	95	Sex, n(%)	Females	1715 (70.2%)	0	661 (66.5%)	
Amputation	95	Smoking status, n(%)	No	1870 (98.1%)	22	978 (98.4%)	
Amputation	95	Smoking status, n(%)	Yes	37 (1.9%)	22	16 (1.6%)	
Amputation	95	Stroke, n(%)	No	1757 (84.8%)	15.2	860 (86.5%)	
Amputation	95	Stroke, n(%)	Yes	316 (15.2%)	15.2	134 (13.5%)	
Amputation	95	Systolic blood pressure, mean(SD)		138 (18.3)	6.5	137.4 (18.2)	
Amputation	95	Time since last healthcare visit, mean(SD)		1.6 (2)	0	1.3 (1.7)	
Amputation	95	Total cholesterol, mean(SD)		5 (1.2)	36.5	4.9 (1.2)	
Amputation	96	Age at diagnosis of type 2 diabetes, mean(SD)		90.4 (3.1)	0	88.9 (2.7)	
Amputation	96	BMI, mean(SD)		25.7 (4.1)	28.8	25.4 (3.9)	
Amputation	96	HbA1c, mean(SD)		50.8 (11.4)	7	50.6 (11)	
Amputation	96	HDL-cholesterol, mean(SD)		1.4 (0.4)	46.1	1.4 (0.4)	
Amputation	96	Ischemic heart disease, n(%)	No	885 (64.2%)	14.6	390 (63.8%)	
Amputation	96	Ischemic heart disease, n(%)	Yes	494 (35.8%)	14.6	221 (36.2%)	
Amputation	96	Neuropathy, n(%)	No	1117 (69.2%)	0	285 (46.6%)	
Amputation	96	Neuropathy, n(%)	Yes	497 (30.8%)	0	326 (53.4%)	
Amputation	96	Self-reported physical activity, n(%)	Never	395 (38.4%)	36.3	208 (34%)	
Amputation	96	Self-reported physical activity, n(%)	<1 times/week	148 (14.4%)	36.3	89 (14.6%)	

Amputation	96	Self-reported physical activity, n(%)	regularly - 1-2 times/week	171 (16.6%)	36.3	104 (17%)	
Amputation	96	Self-reported physical activity, n(%)	regularly - 3-5 times/week	140 (13.6%)	36.3	93 (15.2%)	
Amputation	96	Self-reported physical activity, n(%)	Daily	174 (16.9%)	36.3	117 (19.1%)	
Amputation	96	Sex, n(%)	Males	461 (28.6%)	0	187 (30.6%)	n=1614 vs n=611
Amputation	96	Sex, n(%)	Females	1153 (71.4%)	0	424 (69.4%)	
Amputation	96	Smoking status, n(%)	No	1185 (98.1%)	25.2	599 (98%)	
Amputation	96	Smoking status, n(%)	Yes	23 (1.9%)	25.2	12 (2%)	
Amputation	96	Stroke, n(%)	No	1153 (85%)	15.9	535 (87.6%)	
Amputation	96	Stroke, n(%)	Yes	204 (15%)	15.9	76 (12.4%)	
Amputation	96	Systolic blood pressure, mean(SD)		138.7 (18.6)	7.2	138 (18.4)	
Amputation	96	Time since last healthcare visit, mean(SD)		1.7 (2.1)	0	1.3 (1.8)	
Amputation	96	Total cholesterol, mean(SD)		5 (1.2)	39.3	5 (1.2)	
Amputation	97	Age at diagnosis of type 2 diabetes, mean(SD)		91.4 (3.1)	0	89.8 (2.7)	
Amputation	97	BMI, mean(SD)		25.8 (4)	31.2	25.4 (3.8)	
Amputation	97	HbA1c, mean(SD)		50.5 (10.9)	7.2	50.8 (10.9)	
Amputation	97	HDL-cholesterol, mean(SD)		1.4 (0.4)	49.2	1.4 (0.4)	
Amputation	97	Ischemic heart disease, n(%)	No	557 (62.3%)	14.6	232 (61.7%)	
Amputation	97	Ischemic heart disease, n(%)	Yes	337 (37.7%)	14.6	144 (38.3%)	
Amputation	97	Neuropathy, n(%)	No	735 (70.2%)	0	174 (46.3%)	
Amputation	97	Neuropathy, n(%)	Yes	312 (29.8%)	0	202 (53.7%)	
Amputation	97	Self-reported physical activity, n(%)	Never	267 (40.5%)	37.1	138 (36.7%)	
Amputation	97	Self-reported physical activity, n(%)	<1 times/week	97 (14.7%)	37.1	50 (13.3%)	
Amputation	97	Self-reported physical activity, n(%)	regularly - 1-2 times/week	109 (16.5%)	37.1	68 (18.1%)	

Amputation	97	Self-reported physical activity, n(%)	regularly - 3-5 times/week	85 (12.9%)	37.1	56 (14.9%)	
Amputation	97	Self-reported physical activity, n(%)	Daily	101 (15.3%)	37.1	64 (17%)	
Amputation	97	Sex, n(%)	Males	274 (26.2%)	0	112 (29.8%)	n=1047 vs n=376
Amputation	97	Sex, n(%)	Females	773 (73.8%)	0	264 (70.2%)	
Amputation	97	Smoking status, n(%)	No	758 (97.9%)	26.1	367 (97.6%)	
Amputation	97	Smoking status, n(%)	Yes	16 (2.1%)	26.1	9 (2.4%)	
Amputation	97	Stroke, n(%)	No	746 (84.7%)	15.9	324 (86.2%)	
Amputation	97	Stroke, n(%)	Yes	135 (15.3%)	15.9	52 (13.8%)	
Amputation	97	Systolic blood pressure, mean(SD)		138.5 (18.7)	6.2	136.9 (19.2)	
Amputation	97	Time since last healthcare visit, mean(SD)		1.8 (2.1)	0	1.4 (1.7)	
Amputation	97	Total cholesterol, mean(SD)		5.1 (1.1)	41.8	5 (1.1)	
Amputation	98	Age at diagnosis of type 2 diabetes, mean(SD)		92.2 (3)	0	90.5 (2.8)	
Amputation	98	BMI, mean(SD)		25.5 (3.9)	32.8	25.3 (3.7)	
Amputation	98	HbA1c, mean(SD)		50.4 (11.5)	8.2	51.1 (11.1)	
Amputation	98	HDL-cholesterol, mean(SD)		1.4 (0.4)	49	1.4 (0.5)	
Amputation	98	Ischemic heart disease, n(%)	No	350 (62.8%)	14.2	142 (62.6%)	
Amputation	98	Ischemic heart disease, n(%)	Yes	207 (37.2%)	14.2	85 (37.4%)	
Amputation	98	Neuropathy, n(%)	No	469 (72.3%)	0	114 (50.2%)	
Amputation	98	Neuropathy, n(%)	Yes	180 (27.7%)	0	113 (49.8%)	
Amputation	98	Self-reported physical activity, n(%)	Never	166 (41.8%)	38.8	79 (34.8%)	
Amputation	98	Self-reported physical activity, n(%)	<1 times/week	60 (15.1%)	38.8	36 (15.9%)	
Amputation	98	Self-reported physical activity, n(%)	regularly - 1-2 times/week	69 (17.4%)	38.8	46 (20.3%)	
Amputation	98	Self-reported physical activity, n(%)	regularly - 3-5 times/week	48 (12.1%)	38.8	28 (12.3%)	

Amputation	98	Self-reported physical activity, n(%)	Daily	54 (13.6%)	38.8	38 (16.7%)	
Amputation	98	Sex, n(%)	Males	152 (23.4%)	0	68 (30%)	n=649 vs n=227
Amputation	98	Sex, n(%)	Females	497 (76.6%)	0	159 (70%)	
Amputation	98	Smoking status, n(%)	No	464 (98.3%)	27.3	223 (98.2%)	
Amputation	98	Smoking status, n(%)	Yes	8 (1.7%)	27.3	4 (1.8%)	
Amputation	98	Stroke, n(%)	No	461 (84.1%)	15.6	190 (83.7%)	
Amputation	98	Stroke, n(%)	Yes	87 (15.9%)	15.6	37 (16.3%)	
Amputation	98	Systolic blood pressure, mean(SD)		137.9 (18.4)	6.5	136.1 (17.9)	
Amputation	98	Time since last healthcare visit, mean(SD)		1.9 (2.2)	0	1.5 (1.9)	
Amputation	98	Total cholesterol, mean(SD)		5.1 (1.2)	41.4	5 (1.2)	
Amputation	99	Age at diagnosis of type 2 diabetes, mean(SD)		93.2 (3.1)	0	91.3 (2.8)	
Amputation	99	BMI, mean(SD)		25.6 (4.1)	35.2	25.1 (3.9)	
Amputation	99	HbA1c, mean(SD)		51.1 (12.9)	9.3	52.4 (12.9)	
Amputation	99	HDL-cholesterol, mean(SD)		1.4 (0.4)	51.7	1.3 (0.4)	
Amputation	99	Ischemic heart disease, n(%)	No	200 (62.7%)	14.9	74 (61.7%)	
Amputation	99	Ischemic heart disease, n(%)	Yes	119 (37.3%)	14.9	46 (38.3%)	
Amputation	99	Neuropathy, n(%)	No	280 (74.7%)	0	57 (47.5%)	
Amputation	99	Neuropathy, n(%)	Yes	95 (25.3%)	0	63 (52.5%)	
Amputation	99	Self-reported physical activity, n(%)	Never	104 (47.1%)	41.1	40 (33.3%)	
Amputation	99	Self-reported physical activity, n(%)	<1 times/week	30 (13.6%)	41.1	22 (18.3%)	
Amputation	99	Self-reported physical activity, n(%)	regularly - 1-2 times/week	34 (15.4%)	41.1	20 (16.7%)	
Amputation	99	Self-reported physical activity, n(%)	regularly - 3-5 times/week	19 (8.6%)	41.1	13 (10.8%)	
Amputation	99	Self-reported physical activity, n(%)	Daily	34 (15.4%)	41.1	25 (20.8%)	

Amputation	99	Sex, n(%)	Males	87 (23.2%)	0	29 (24.2%)	n=375 vs n=120
Amputation	99	Sex, n(%)	Females	288 (76.8%)	0	91 (75.8%)	
Amputation	99	Smoking status, n(%)	No	264 (98.1%)	28.3	119 (99.2%)	
Amputation	99	Smoking status, n(%)	Yes	5 (1.9%)	28.3	1 (0.8%)	
Amputation	99	Stroke, n(%)	No	271 (85.5%)	15.5	104 (86.7%)	
Amputation	99	Stroke, n(%)	Yes	46 (14.5%)	15.5	16 (13.3%)	
Amputation	99	Systolic blood pressure, mean(SD)		138.6 (18)	7.5	138 (16.2)	
Amputation	99	Time since last healthcare visit, mean(SD)		2 (2.3)	0	1.6 (2)	
Amputation	99	Total cholesterol, mean(SD)		5.2 (1.2)	45.9	5.1 (1.2)	
Amputation	100	Age at diagnosis of type 2 diabetes, mean(SD)		94.2 (3.1)	0	92 (2.5)	
Amputation	100	BMI, mean(SD)		25.5 (4.1)	40.6	25.2 (4.1)	
Amputation	100	HbA1c, mean(SD)		51.8 (13.2)	12.6	53.7 (14.4)	
Amputation	100	HDL-cholesterol, mean(SD)		1.3 (0.4)	60.4	1.3 (0.3)	
Amputation	100	Ischemic heart disease, n(%)	No	111 (63.8%)	15.9	35 (63.6%)	
Amputation	100	Ischemic heart disease, n(%)	Yes	63 (36.2%)	15.9	20 (36.4%)	
Amputation	100	Neuropathy, n(%)	No	152 (73.4%)	0	24 (43.6%)	
Amputation	100	Neuropathy, n(%)	Yes	55 (26.6%)	0	31 (56.4%)	
Amputation	100	Self-reported physical activity, n(%)	<1 times/week	19 (16.2%)	43.5	14 (25.5%)	
Amputation	100	Self-reported physical activity, n(%)	regularly - 1-2 times/week	16 (13.7%)	43.5	10 (18.2%)	
Amputation	100	Self-reported physical activity, n(%)	regularly - 3-5 times/week	11 (9.4%)	43.5	8 (14.5%)	
Amputation	100	Self-reported physical activity, n(%)	Daily	14 (12%)	43.5	7 (12.7%)	
Amputation	100	Self-reported physical activity, n(%)	Never	57 (48.7%)	43.5	16 (29.1%)	
Amputation	100	Sex, n(%)	Males	45 (21.7%)	0	11 (20%)	n=207 vs n=55

Amputation	100	Sex, n(%)	Females	162 (78.3%)	0	44 (80%)
Amputation	100	Smoking status, n(%)	No	141 (97.9%)	30.4	55 (100%)
Amputation	100	Smoking status, n(%)	Yes	3 (2.1%)	30.4	0 (0%)
Amputation	100	Stroke, n(%)	No	152 (88.9%)	17.4	52 (94.5%)
Amputation	100	Stroke, n(%)	Yes	19 (11.1%)	17.4	3 (5.5%)
Amputation	100	Systolic blood pressure, mean(SD)		137.4 (17.6)	8.7	136.6 (15.8)
Amputation	100	Time since last healthcare visit, mean(SD)		2.2 (2.4)	0	1.3 (1.9)
Amputation	100	Total cholesterol, mean(SD)		5.2 (1.1)	54.6	5 (1.1)
Major amputation	19	Age at diagnosis of type 2 diabetes, mean(SD)		18.5 (0.3)	0	18.5 (0.3)
Major amputation	19	BMI, mean(SD)		34.2 (6.9)	17.7	35.1 (7.2)
Major amputation	19	HbA1c, mean(SD)		55.1 (19.7)	19.4	57.1 (19)
Major amputation	19	HDL-cholesterol, mean(SD)		0.9 (0.2)	46.8	0.9 (0.2)
Major amputation	19	Ischemic heart disease, n(%)	No	44 (100%)	29	17 (100%)
Major amputation	19	Neuropathy, n(%)	No	62 (100%)	0	17 (100%)
Major amputation	19	Self-reported physical activity, n(%)	Never	10 (25%)	35.5	5 (29.4%)
Major amputation	19	Self-reported physical activity, n(%)	<1 times/week	8 (20%)	35.5	2 (11.8%)
Major amputation	19	Self-reported physical activity, n(%)	regularly - 1-2 times/week	8 (20%)	35.5	4 (23.5%)
Major amputation	19	Self-reported physical activity, n(%)	regularly - 3-5 times/week	8 (20%)	35.5	3 (17.6%)

Major amputation	19	Self-reported physical activity, n(%)	Daily	6 (15%)	35.5	3 (17.6%)	
Major amputation	19	Sex, n(%)	Males	22 (35.5%)	0	6 (35.3%)	n=62 vs n=17
Major amputation	19	Sex, n(%)	Females	40 (64.5%)	0	11 (64.7%)	
Major amputation	19	Smoking status, n(%)	No	28 (68.3%)	33.9	10 (58.8%)	
Major amputation	19	Smoking status, n(%)	Yes	13 (31.7%)	33.9	7 (41.2%)	
Major amputation	19	Stroke, n(%)	No	43 (100%)	30.6	17 (100%)	
Major amputation	19	Systolic blood pressure, mean(SD)		124.6 (10.3)	22.6	124.1 (8.5)	
Major amputation	19	Time since last healthcare visit, mean(SD)		0.3 (0.2)	0	0.1 (0.1)	
Major amputation	19	Total cholesterol, mean(SD)		4.8 (1.1)	45.2	5 (1.1)	
Major amputation	20	Age at diagnosis of type 2 diabetes, mean(SD)		19.2 (1.9)	0	19 (0.6)	
Major amputation	20	BMI, mean(SD)		35.6 (8)	22.2	36.8 (8.2)	
Major amputation	20	HbA1c, mean(SD)		55.6 (21.2)	15.3	58.1 (20.5)	
Major amputation	20	HDL-cholesterol, mean(SD)		1 (0.3)	42.4	1 (0.3)	
Major amputation	20	Ischemic heart disease, n(%)	No	118 (100%)	18.1	51 (100%)	
Major amputation	20	Neuropathy, n(%)	No	140 (97.2%)	0	49 (96.1%)	

Major amputation	20	Neuropathy, n(%)	Yes	4 (2.8%)	0	2 (3.9%)	
Major amputation	20	Self-reported physical activity, n(%)	Never	16 (16.7%)	33.3	12 (23.5%)	
Major amputation	20	Self-reported physical activity, n(%)	<1 times/week	13 (13.5%)	33.3	6 (11.8%)	
Major amputation	20	Self-reported physical activity, n(%)	regularly - 1-2 times/week	21 (21.9%)	33.3	12 (23.5%)	
Major amputation	20	Self-reported physical activity, n(%)	regularly - 3-5 times/week	28 (29.2%)	33.3	14 (27.5%)	
Major amputation	20	Self-reported physical activity, n(%)	Daily	18 (18.8%)	33.3	7 (13.7%)	
Major amputation	20	Sex, n(%)	Males	67 (46.9%)	0.7	25 (49%)	n=144 vs n=51
Major amputation	20	Sex, n(%)	Females	76 (53.1%)	0.7	26 (51%)	
Major amputation	20	Smoking status, n(%)	No	73 (73%)	30.6	35 (68.6%)	
Major amputation	20	Smoking status, n(%)	Yes	27 (27%)	30.6	16 (31.4%)	
Major amputation	20	Stroke, n(%)	No	117 (100%)	18.8	51 (100%)	
Major amputation	20	Systolic blood pressure, mean(SD)		123.7 (11.8)	21.5	125.2 (12.9)	
Major amputation	20	Time since last healthcare visit, mean(SD)		0.3 (1.8)	0	0.3 (0.3)	
Major amputation	20	Total cholesterol, mean(SD)		4.7 (1)	37.5	4.8 (1.1)	
Major amputation	21	Age at diagnosis of type 2 diabetes, mean(SD)		19.7 (1.6)	0	19.5 (0.8)	

Major amputation	21	BMI, mean(SD)		35.7 (7.6)	20.3	35.3 (7.4)	
Major amputation	21	HbA1c, mean(SD)		55.1 (20.2)	12.4	54.6 (20.8)	
Major amputation	21	HDL-cholesterol, mean(SD)		1 (0.3)	32	1 (0.3)	
Major amputation	21	Ischemic heart disease, n(%)	No	194 (99%)	18.7	110 (100%)	
Major amputation	21	Ischemic heart disease, n(%)	Yes	2 (1%)	18.7	0 (0%)	
Major amputation	21	Neuropathy, n(%)	No	236 (97.9%)	0	106 (96.4%)	
Major amputation	21	Neuropathy, n(%)	Yes	5 (2.1%)	0	4 (3.6%)	
Major amputation	21	Self-reported physical activity, n(%)	Never	31 (18.1%)	29	20 (18.2%)	
Major amputation	21	Self-reported physical activity, n(%)	<1 times/week	33 (19.3%)	29	21 (19.1%)	
Major amputation	21	Self-reported physical activity, n(%)	regularly - 1-2 times/week	33 (19.3%)	29	22 (20%)	
Major amputation	21	Self-reported physical activity, n(%)	regularly - 3-5 times/week	34 (19.9%)	29	23 (20.9%)	
Major amputation	21	Self-reported physical activity, n(%)	Daily	40 (23.4%)	29	24 (21.8%)	
Major amputation	21	Sex, n(%)	Males	118 (49.2%)	0.4	57 (51.8%)	n=241 vs n=110
Major amputation	21	Sex, n(%)	Females	122 (50.8%)	0.4	53 (48.2%)	
Major amputation	21	Smoking status, n(%)	No	133 (75.1%)	26.6	80 (72.7%)	

Major amputation	21	Smoking status, n(%)	Yes	44 (24.9%)	26.6	30 (27.3%)
Major amputation	21	Stroke, n(%)	No	194 (100%)	19.5	110 (100%)
Major amputation	21	Systolic blood pressure, mean(SD)		124 (13)	16.2	123.3 (12.9)
Major amputation	21	Time since last healthcare visit, mean(SD)		0.5 (1.4)	0	0.5 (0.5)
Major amputation	21	Total cholesterol, mean(SD)		4.8 (1)	28.2	4.8 (1)
Major amputation	22	Age at diagnosis of type 2 diabetes, mean(SD)		20.3 (1.5)	0	20.1 (1)
Major amputation	22	BMI, mean(SD)		35.6 (7.3)	18.9	35.5 (6.9)
Major amputation	22	HbA1c, mean(SD)		55.9 (21.9)	10.7	55.7 (21.4)
Major amputation	22	HDL-cholesterol, mean(SD)		1 (0.3)	27.9	1 (0.4)
Major amputation	22	Ischemic heart disease, n(%)	No	298 (99%)	17.5	185 (99.5%)
Major amputation	22	Ischemic heart disease, n(%)	Yes	3 (1%)	17.5	1 (0.5%)
Major amputation	22	Neuropathy, n(%)	No	356 (97.5%)	0	181 (97.3%)
Major amputation	22	Neuropathy, n(%)	Yes	9 (2.5%)	0	5 (2.7%)
Major amputation	22	Self-reported physical activity, n(%)	Never	49 (18.8%)	28.5	42 (22.6%)
Major amputation	22	Self-reported physical activity, n(%)	<1 times/week	46 (17.6%)	28.5	30 (16.1%)

Major amputation	22	Self-reported physical activity, n(%)	regularly - 1-2 times/week	45 (17.2%)	28.5	29 (15.6%)	
Major amputation	22	Self-reported physical activity, n(%)	regularly - 3-5 times/week	53 (20.3%)	28.5	37 (19.9%)	
Major amputation	22	Self-reported physical activity, n(%)	Daily	68 (26.1%)	28.5	48 (25.8%)	
Major amputation	22	Sex, n(%)	Males	168 (46.2%)	0.3	86 (46.2%)	n=365 vs n=186
Major amputation	22	Sex, n(%)	Females	196 (53.8%)	0.3	100 (53.8%)	
Major amputation	22	Smoking status, n(%)	No	203 (75.2%)	26	143 (76.9%)	
Major amputation	22	Smoking status, n(%)	Yes	67 (24.8%)	26	43 (23.1%)	
Major amputation	22	Stroke, n(%)	No	296 (99.7%)	18.6	186 (100%)	
Major amputation	22	Stroke, n(%)	Yes	1 (0.3%)	18.6	0 (0%)	
Major amputation	22	Systolic blood pressure, mean(SD)		124.3 (13.7)	15.3	125.1 (13)	
Major amputation	22	Time since last healthcare visit, mean(SD)		0.6 (1.3)	0	0.5 (0.6)	
Major amputation	22	Total cholesterol, mean(SD)		4.9 (1.1)	24.9	4.9 (1.1)	
Major amputation	23	Age at diagnosis of type 2 diabetes, mean(SD)		21 (1.6)	0	20.7 (1.3)	
Major amputation	23	BMI, mean(SD)		35.8 (7.6)	18.8	35.9 (7)	
Major amputation	23	HbA1c, mean(SD)		55.1 (19.3)	9	55.4 (19.9)	

Major amputation	23	HDL-cholesterol, mean(SD)		1 (0.3)	27.9	1 (0.4)	
Major amputation	23	Ischemic heart disease, n(%)	No	418 (99.3%)	19	276 (100%)	
Major amputation	23	Ischemic heart disease, n(%)	Yes	3 (0.7%)	19	0 (0%)	
Major amputation	23	Neuropathy, n(%)	No	507 (97.5%)	0	267 (96.7%)	
Major amputation	23	Neuropathy, n(%)	Yes	13 (2.5%)	0	9 (3.3%)	
Major amputation	23	Self-reported physical activity, n(%)	Never	71 (18.5%)	26.3	54 (19.6%)	
Major amputation	23	Self-reported physical activity, n(%)	<1 times/week	63 (16.4%)	26.3	47 (17%)	
Major amputation	23	Self-reported physical activity, n(%)	regularly - 1-2 times/week	70 (18.3%)	26.3	51 (18.5%)	
Major amputation	23	Self-reported physical activity, n(%)	regularly - 3-5 times/week	87 (22.7%)	26.3	64 (23.2%)	
Major amputation	23	Self-reported physical activity, n(%)	Daily	92 (24%)	26.3	60 (21.7%)	
Major amputation	23	Sex, n(%)	Males	247 (47.6%)	0.2	136 (49.3%)	n=520 vs n=276
Major amputation	23	Sex, n(%)	Females	272 (52.4%)	0.2	140 (50.7%)	
Major amputation	23	Smoking status, n(%)	No	289 (72.6%)	23.5	206 (74.6%)	
Major amputation	23	Smoking status, n(%)	Yes	109 (27.4%)	23.5	70 (25.4%)	
Major amputation	23	Stroke, n(%)	No	416 (99.8%)	19.8	276 (100%)	

Major amputation	23	Stroke, n(%)	Yes	1 (0.2%)	19.8	0 (0%)
Major amputation	23	Systolic blood pressure, mean(SD)		124.6 (13.5)	15	124.7 (13.5)
Major amputation	23	Time since last healthcare visit, mean(SD)		0.7 (1.3)	0	0.6 (0.8)
Major amputation	23	Total cholesterol, mean(SD)		4.8 (1.1)	24	4.8 (1.1)
Major amputation	24	Age at diagnosis of type 2 diabetes, mean(SD)		21.8 (1.9)	0	21.4 (1.6)
Major amputation	24	BMI, mean(SD)		35.7 (7.5)	16.9	36 (7)
Major amputation	24	HbA1c, mean(SD)		55.2 (20.9)	8.9	54.2 (19.4)
Major amputation	24	HDL-cholesterol, mean(SD)		1 (0.4)	28.1	1 (0.4)
Major amputation	24	Ischemic heart disease, n(%)	No	601 (99.2%)	15.8	386 (100%)
Major amputation	24	Ischemic heart disease, n(%)	Yes	5 (0.8%)	15.8	0 (0%)
Major amputation	24	Neuropathy, n(%)	No	703 (97.6%)	0	372 (96.4%)
Major amputation	24	Neuropathy, n(%)	Yes	17 (2.4%)	0	14 (3.6%)
Major amputation	24	Self-reported physical activity, n(%)	Never	77 (14.3%)	25.1	52 (13.5%)
Major amputation	24	Self-reported physical activity, n(%)	<1 times/week	90 (16.7%)	25.1	65 (16.8%)
Major amputation	24	Self-reported physical activity, n(%)	regularly - 1-2 times/week	113 (21%)	25.1	84 (21.8%)

Major amputation	24	Self-reported physical activity, n(%)	regularly - 3-5 times/week	130 (24.1%)	25.1	96 (24.9%)	
Major amputation	24	Self-reported physical activity, n(%)	Daily	129 (23.9%)	25.1	89 (23.1%)	
Major amputation	24	Sex, n(%)	Males	335 (46.7%)	0.3	181 (46.9%)	n=720 vs n=386
Major amputation	24	Sex, n(%)	Females	383 (53.3%)	0.3	205 (53.1%)	
Major amputation	24	Smoking status, n(%)	No	421 (72.7%)	19.6	286 (74.1%)	
Major amputation	24	Smoking status, n(%)	Yes	158 (27.3%)	19.6	100 (25.9%)	
Major amputation	24	Stroke, n(%)	No	598 (99.5%)	16.5	384 (99.5%)	
Major amputation	24	Stroke, n(%)	Yes	3 (0.5%)	16.5	2 (0.5%)	
Major amputation	24	Systolic blood pressure, mean(SD)		124.3 (13.6)	12.9	124.5 (13.5)	
Major amputation	24	Time since last healthcare visit, mean(SD)		0.8 (1.6)	0	0.7 (0.9)	
Major amputation	24	Total cholesterol, mean(SD)		4.9 (1.3)	25.3	4.8 (1.2)	
Major amputation	25	Age at diagnosis of type 2 diabetes, mean(SD)		22.4 (2)	0	22.1 (1.7)	
Major amputation	25	BMI, mean(SD)		35.7 (7.8)	15.6	35.4 (7.5)	
Major amputation	25	HbA1c, mean(SD)		53.6 (20.1)	7.7	53 (19.4)	
Major amputation	25	HDL-cholesterol, mean(SD)		1 (0.4)	26.5	1 (0.4)	

Major amputation	25	Ischemic heart disease, n(%)	No	757 (99.3%)	14.9	486 (100%)	
Major amputation	25	Ischemic heart disease, n(%)	Yes	5 (0.7%)	14.9	0 (0%)	
Major amputation	25	Neuropathy, n(%)	No	872 (97.4%)	0	467 (96.1%)	
Major amputation	25	Neuropathy, n(%)	Yes	23 (2.6%)	0	19 (3.9%)	
Major amputation	25	Self-reported physical activity, n(%)	Never	107 (15.6%)	23.4	72 (14.8%)	
Major amputation	25	Self-reported physical activity, n(%)	<1 times/week	108 (15.7%)	23.4	81 (16.7%)	
Major amputation	25	Self-reported physical activity, n(%)	regularly - 1-2 times/week	136 (19.8%)	23.4	93 (19.1%)	
Major amputation	25	Self-reported physical activity, n(%)	regularly - 3-5 times/week	165 (24.1%)	23.4	125 (25.7%)	
Major amputation	25	Self-reported physical activity, n(%)	Daily	170 (24.8%)	23.4	115 (23.7%)	
Major amputation	25	Sex, n(%)	Males	419 (46.9%)	0.2	235 (48.4%)	n=895 vs n=486
Major amputation	25	Sex, n(%)	Females	474 (53.1%)	0.2	251 (51.6%)	
Major amputation	25	Smoking status, n(%)	No	544 (74.7%)	18.7	370 (76.1%)	
Major amputation	25	Smoking status, n(%)	Yes	184 (25.3%)	18.7	116 (23.9%)	
Major amputation	25	Stroke, n(%)	No	750 (99.6%)	15.9	485 (99.8%)	
Major amputation	25	Stroke, n(%)	Yes	3 (0.4%)	15.9	1 (0.2%)	

Major amputation	25	Systolic blood pressure, mean(SD)		123.8 (13.4)	10.7	123.5 (12.9)
Major amputation	25	Time since last healthcare visit, mean(SD)		0.9 (1.6)	0	0.7 (1)
Major amputation	25	Total cholesterol, mean(SD)		4.8 (1.2)	23.6	4.8 (1.1)
Major amputation	26	Age at diagnosis of type 2 diabetes, mean(SD)		23.2 (2.1)	0	22.8 (1.9)
Major amputation	26	BMI, mean(SD)		35.4 (7.7)	14.3	35.2 (7.2)
Major amputation	26	HbA1c, mean(SD)		53.5 (19.4)	7.9	52.8 (19.6)
Major amputation	26	HDL-cholesterol, mean(SD)		1 (0.3)	25.9	1 (0.3)
Major amputation	26	Ischemic heart disease, n(%)	No	918 (99.5%)	14.5	603 (99.8%)
Major amputation	26	Ischemic heart disease, n(%)	Yes	5 (0.5%)	14.5	1 (0.2%)
Major amputation	26	Neuropathy, n(%)	No	1049 (97.1%)	0	579 (95.9%)
Major amputation	26	Neuropathy, n(%)	Yes	31 (2.9%)	0	25 (4.1%)
Major amputation	26	Self-reported physical activity, n(%)	Never	120 (14.3%)	22.2	84 (13.9%)
Major amputation	26	Self-reported physical activity, n(%)	<1 times/week	113 (13.5%)	22.2	81 (13.4%)
Major amputation	26	Self-reported physical activity, n(%)	regularly - 1-2 times/week	164 (19.5%)	22.2	120 (19.9%)
Major amputation	26	Self-reported physical activity, n(%)	regularly - 3-5 times/week	232 (27.6%)	22.2	172 (28.5%)

Major amputation	26	Self-reported physical activity, n(%)	Daily	211 (25.1%)	22.2	147 (24.3%)	
Major amputation	26	Sex, n(%)	Males	544 (50.5%)	0.2	303 (50.2%)	n=1080 vs n=604
Major amputation	26	Sex, n(%)	Females	534 (49.5%)	0.2	301 (49.8%)	
Major amputation	26	Smoking status, n(%)	No	683 (76.3%)	17.1	461 (76.3%)	
Major amputation	26	Smoking status, n(%)	Yes	212 (23.7%)	17.1	143 (23.7%)	
Major amputation	26	Stroke, n(%)	No	907 (99.6%)	15.6	602 (99.7%)	
Major amputation	26	Stroke, n(%)	Yes	4 (0.4%)	15.6	2 (0.3%)	
Major amputation	26	Systolic blood pressure, mean(SD)		124.4 (13.4)	10.3	124.4 (13.3)	
Major amputation	26	Time since last healthcare visit, mean(SD)		0.9 (1.6)	0	0.7 (1)	
Major amputation	26	Total cholesterol, mean(SD)		4.9 (1.2)	22.9	4.8 (1.2)	
Major amputation	27	Age at diagnosis of type 2 diabetes, mean(SD)		24 (2.3)	0	23.6 (2.1)	
Major amputation	27	BMI, mean(SD)		35 (7.5)	15.5	35.2 (7.3)	
Major amputation	27	HbA1c, mean(SD)		53 (18.9)	7.5	53.6 (19.2)	
Major amputation	27	HDL-cholesterol, mean(SD)		1 (0.3)	26.2	1 (0.4)	
Major amputation	27	Ischemic heart disease, n(%)	No	1098 (99.3%)	14	719 (99.6%)	

Major amputation	27	Ischemic heart disease, n(%)	Yes	8 (0.7%)	14	3 (0.4%)	
Major amputation	27	Neuropathy, n(%)	No	1239 (96.3%)	0	686 (95%)	
Major amputation	27	Neuropathy, n(%)	Yes	47 (3.7%)	0	36 (5%)	
Major amputation	27	Self-reported physical activity, n(%)	Never	132 (13.4%)	23.4	96 (13.3%)	
Major amputation	27	Self-reported physical activity, n(%)	<1 times/week	137 (13.9%)	23.4	98 (13.6%)	
Major amputation	27	Self-reported physical activity, n(%)	regularly - 1-2 times/week	188 (19.1%)	23.4	137 (19%)	
Major amputation	27	Self-reported physical activity, n(%)	regularly - 3-5 times/week	285 (28.9%)	23.4	225 (31.2%)	
Major amputation	27	Self-reported physical activity, n(%)	Daily	243 (24.7%)	23.4	166 (23%)	
Major amputation	27	Sex, n(%)	Males	648 (50.5%)	0.3	379 (52.5%)	n=1286 vs n=722
Major amputation	27	Sex, n(%)	Females	634 (49.5%)	0.3	343 (47.5%)	
Major amputation	27	Smoking status, n(%)	No	798 (75.6%)	17.9	547 (75.8%)	
Major amputation	27	Smoking status, n(%)	Yes	258 (24.4%)	17.9	175 (24.2%)	
Major amputation	27	Stroke, n(%)	No	1091 (99.3%)	14.5	719 (99.6%)	
Major amputation	27	Stroke, n(%)	Yes	8 (0.7%)	14.5	3 (0.4%)	
Major amputation	27	Systolic blood pressure, mean(SD)		124.6 (13.8)	10.2	124.7 (14)	

Major amputation	27	Time since last healthcare visit, mean(SD)		1 (1.7)	0	0.7 (1.1)
Major amputation	27	Total cholesterol, mean(SD)		4.8 (1.2)	21.8	4.8 (1.2)
Major amputation	28	Age at diagnosis of type 2 diabetes, mean(SD)		24.8 (2.5)	0	24.4 (2.2)
Major amputation	28	BMI, mean(SD)		35.1 (7.4)	15.2	35.1 (7.2)
Major amputation	28	HbA1c, mean(SD)		52.9 (18.6)	7.6	53.7 (18.2)
Major amputation	28	HDL-cholesterol, mean(SD)		1 (0.3)	26.1	1 (0.3)
Major amputation	28	Ischemic heart disease, n(%)	No	1277 (98.8%)	14.2	837 (98.8%)
Major amputation	28	Ischemic heart disease, n(%)	Yes	15 (1.2%)	14.2	10 (1.2%)
Major amputation	28	Neuropathy, n(%)	No	1452 (96.4%)	0	805 (95%)
Major amputation	28	Neuropathy, n(%)	Yes	54 (3.6%)	0	42 (5%)
Major amputation	28	Self-reported physical activity, n(%)	Never	151 (13%)	23	110 (13%)
Major amputation	28	Self-reported physical activity, n(%)	<1 times/week	126 (10.9%)	23	88 (10.4%)
Major amputation	28	Self-reported physical activity, n(%)	regularly - 1-2 times/week	254 (21.9%)	23	181 (21.4%)
Major amputation	28	Self-reported physical activity, n(%)	regularly - 3-5 times/week	321 (27.7%)	23	244 (28.8%)
Major amputation	28	Self-reported physical activity, n(%)	Daily	308 (26.6%)	23	224 (26.4%)

Major amputation	28	Sex, n(%)	Males	747 (49.7%)	0.2	438 (51.7%)	n=1506 vs n=847
Major amputation	28	Sex, n(%)	Females	756 (50.3%)	0.2	409 (48.3%)	
Major amputation	28	Smoking status, n(%)	No	942 (76%)	17.7	652 (77%)	
Major amputation	28	Smoking status, n(%)	Yes	297 (24%)	17.7	195 (23%)	
Major amputation	28	Stroke, n(%)	No	1278 (99.1%)	14.4	840 (99.2%)	
Major amputation	28	Stroke, n(%)	Yes	11 (0.9%)	14.4	7 (0.8%)	
Major amputation	28	Systolic blood pressure, mean(SD)		124.8 (13.4)	10.2	125.4 (13.5)	
Major amputation	28	Time since last healthcare visit, mean(SD)		1 (1.7)	0	0.8 (1.1)	
Major amputation	28	Total cholesterol, mean(SD)		4.8 (1.1)	21.6	4.8 (1.1)	
Major amputation	29	Age at diagnosis of type 2 diabetes, mean(SD)		25.7 (2.5)	0	25.2 (2.4)	
Major amputation	29	BMI, mean(SD)		34.8 (7.5)	14.6	34.7 (7.1)	
Major amputation	29	HbA1c, mean(SD)		53.7 (18.9)	7.2	54 (18.4)	
Major amputation	29	HDL-cholesterol, mean(SD)		1 (0.3)	25.8	1 (0.3)	
Major amputation	29	Ischemic heart disease, n(%)	No	1474 (99.1%)	13.6	980 (99.2%)	
Major amputation	29	Ischemic heart disease, n(%)	Yes	13 (0.9%)	13.6	8 (0.8%)	

Major amputation	29	Neuropathy, n(%)	No	1649 (95.8%)	0	927 (93.8%)	
Major amputation	29	Neuropathy, n(%)	Yes	73 (4.2%)	0	61 (6.2%)	
Major amputation	29	Self-reported physical activity, n(%)	Never	174 (13.1%)	22.6	127 (12.9%)	
Major amputation	29	Self-reported physical activity, n(%)	<1 times/week	164 (12.3%)	22.6	122 (12.3%)	
Major amputation	29	Self-reported physical activity, n(%)	regularly - 1-2 times/week	280 (21%)	22.6	200 (20.2%)	
Major amputation	29	Self-reported physical activity, n(%)	regularly - 3-5 times/week	353 (26.5%)	22.6	280 (28.3%)	
Major amputation	29	Self-reported physical activity, n(%)	Daily	362 (27.2%)	22.6	259 (26.2%)	
Major amputation	29	Sex, n(%)	Males	877 (51%)	0.2	512 (51.8%)	n=1722 vs n=988
Major amputation	29	Sex, n(%)	Females	842 (49%)	0.2	476 (48.2%)	
Major amputation	29	Smoking status, n(%)	No	1108 (77.4%)	16.9	757 (76.6%)	
Major amputation	29	Smoking status, n(%)	Yes	323 (22.6%)	16.9	231 (23.4%)	
Major amputation	29	Stroke, n(%)	No	1469 (99.3%)	14.1	982 (99.4%)	
Major amputation	29	Stroke, n(%)	Yes	11 (0.7%)	14.1	6 (0.6%)	
Major amputation	29	Systolic blood pressure, mean(SD)		124.5 (13.6)	9.6	124.7 (13.5)	
Major amputation	29	Time since last healthcare visit, mean(SD)		1 (1.7)	0	0.8 (1.1)	

Major amputation	29	Total cholesterol, mean(SD)		4.8 (1.1)	21.3	4.8 (1.1)	
Major amputation	30	Age at diagnosis of type 2 diabetes, mean(SD)		26.7 (2.6)	0	26.2 (2.6)	
Major amputation	30	BMI, mean(SD)		34.7 (7.6)	14.2	34.6 (7.4)	
Major amputation	30	HbA1c, mean(SD)		53.6 (19.2)	6.9	54 (18.7)	
Major amputation	30	HDL-cholesterol, mean(SD)		1 (0.3)	25.7	1 (0.3)	
Major amputation	30	Ischemic heart disease, n(%)	No	1680 (99.2%)	13.5	1124 (99.3%)	
Major amputation	30	Ischemic heart disease, n(%)	Yes	13 (0.8%)	13.5	8 (0.7%)	
Major amputation	30	Neuropathy, n(%)	No	1878 (96%)	0	1063 (93.9%)	
Major amputation	30	Neuropathy, n(%)	Yes	79 (4%)	0	69 (6.1%)	
Major amputation	30	Self-reported physical activity, n(%)	Never	190 (12.3%)	21.1	148 (13.1%)	
Major amputation	30	Self-reported physical activity, n(%)	<1 times/week	207 (13.4%)	21.1	141 (12.5%)	
Major amputation	30	Self-reported physical activity, n(%)	regularly - 1-2 times/week	339 (22%)	21.1	247 (21.8%)	
Major amputation	30	Self-reported physical activity, n(%)	regularly - 3-5 times/week	400 (25.9%)	21.1	292 (25.8%)	
Major amputation	30	Self-reported physical activity, n(%)	Daily	408 (26.4%)	21.1	304 (26.9%)	
Major amputation	30	Sex, n(%)	Males	1025 (52.5%)	0.2	618 (54.6%)	n=1957 vs n=1132

Major amputation	30	Sex, n(%)	Females	929 (47.5%)	0.2	514 (45.4%)
Major amputation	30	Smoking status, n(%)	No	1276 (77.4%)	15.7	890 (78.6%)
Major amputation	30	Smoking status, n(%)	Yes	373 (22.6%)	15.7	242 (21.4%)
Major amputation	30	Stroke, n(%)	No	1676 (99.3%)	13.8	1125 (99.4%)
Major amputation	30	Stroke, n(%)	Yes	11 (0.7%)	13.8	7 (0.6%)
Major amputation	30	Systolic blood pressure, mean(SD)		124.6 (13.7)	8.7	125.2 (13.7)
Major amputation	30	Time since last healthcare visit, mean(SD)		1 (1.7)	0	0.7 (1.1)
Major amputation	30	Total cholesterol, mean(SD)		4.8 (1.2)	21.8	4.8 (1.1)
Major amputation	31	Age at diagnosis of type 2 diabetes, mean(SD)		27.6 (2.7)	0	27 (2.7)
Major amputation	31	BMI, mean(SD)		34.3 (7.4)	13.5	34.2 (7)
Major amputation	31	HbA1c, mean(SD)		53.3 (18.6)	6.5	53.6 (18)
Major amputation	31	HDL-cholesterol, mean(SD)		1 (0.3)	25	1 (0.3)
Major amputation	31	Ischemic heart disease, n(%)	No	1934 (99.3%)	12.6	1283 (99.3%)
Major amputation	31	Ischemic heart disease, n(%)	Yes	13 (0.7%)	12.6	9 (0.7%)
Major amputation	31	Neuropathy, n(%)	No	2138 (96%)	0	1222 (94.6%)

Major amputation	31	Neuropathy, n(%)	Yes	89 (4%)	0	70 (5.4%)	
Major amputation	31	Self-reported physical activity, n(%)	Never	218 (12.4%)	21.3	164 (12.7%)	
Major amputation	31	Self-reported physical activity, n(%)	<1 times/week	233 (13.3%)	21.3	163 (12.6%)	
Major amputation	31	Self-reported physical activity, n(%)	regularly - 1-2 times/week	373 (21.3%)	21.3	274 (21.2%)	
Major amputation	31	Self-reported physical activity, n(%)	regularly - 3-5 times/week	459 (26.2%)	21.3	344 (26.6%)	
Major amputation	31	Self-reported physical activity, n(%)	Daily	470 (26.8%)	21.3	347 (26.9%)	
Major amputation	31	Sex, n(%)	Males	1201 (54%)	0.1	730 (56.5%)	n=2227 vs n=1292
Major amputation	31	Sex, n(%)	Females	1023 (46%)	0.1	562 (43.5%)	
Major amputation	31	Smoking status, n(%)	No	1463 (77%)	14.7	1002 (77.6%)	
Major amputation	31	Smoking status, n(%)	Yes	436 (23%)	14.7	290 (22.4%)	
Major amputation	31	Stroke, n(%)	No	1928 (99.3%)	12.8	1280 (99.1%)	
Major amputation	31	Stroke, n(%)	Yes	14 (0.7%)	12.8	12 (0.9%)	
Major amputation	31	Systolic blood pressure, mean(SD)		124.8 (13.8)	8.8	125.1 (13.7)	
Major amputation	31	Time since last healthcare visit, mean(SD)		1 (1.6)	0	0.7 (1)	
Major amputation	31	Total cholesterol, mean(SD)		4.8 (1.2)	20.5	4.8 (1.2)	

Major amputation	32	Age at diagnosis of type 2 diabetes, mean(SD)		28.6 (2.7)	0	28.1 (2.7)	
Major amputation	32	BMI, mean(SD)		34.1 (7.5)	13.2	33.9 (7.4)	
Major amputation	32	HbA1c, mean(SD)		53 (18.7)	6	53.5 (18.2)	
Major amputation	32	HDL-cholesterol, mean(SD)		1 (0.3)	24	1 (0.3)	
Major amputation	32	Ischemic heart disease, n(%)	No	2241 (99.3%)	12.4	1507 (99.4%)	
Major amputation	32	Ischemic heart disease, n(%)	Yes	15 (0.7%)	12.4	9 (0.6%)	
Major amputation	32	Neuropathy, n(%)	No	2463 (95.7%)	0	1433 (94.5%)	
Major amputation	32	Neuropathy, n(%)	Yes	111 (4.3%)	0	83 (5.5%)	
Major amputation	32	Self-reported physical activity, n(%)	Never	277 (13.5%)	20.2	206 (13.6%)	
Major amputation	32	Self-reported physical activity, n(%)	<1 times/week	264 (12.9%)	20.2	187 (12.3%)	
Major amputation	32	Self-reported physical activity, n(%)	regularly - 1-2 times/week	432 (21%)	20.2	307 (20.3%)	
Major amputation	32	Self-reported physical activity, n(%)	regularly - 3-5 times/week	541 (26.4%)	20.2	427 (28.2%)	
Major amputation	32	Self-reported physical activity, n(%)	Daily	539 (26.3%)	20.2	389 (25.7%)	
Major amputation	32	Sex, n(%)	Males	1401 (54.5%)	0.2	869 (57.3%)	n=2574 vs n=1516
Major amputation	32	Sex, n(%)	Females	1169 (45.5%)	0.2	647 (42.7%)	

Major amputation	32	Smoking status, n(%)	No	1714 (77.5%)	14	1191 (78.6%)
Major amputation	32	Smoking status, n(%)	Yes	499 (22.5%)	14	325 (21.4%)
Major amputation	32	Stroke, n(%)	No	2240 (99.4%)	12.5	1505 (99.3%)
Major amputation	32	Stroke, n(%)	Yes	13 (0.6%)	12.5	11 (0.7%)
Major amputation	32	Systolic blood pressure, mean(SD)		124.9 (14.2)	7.7	125.3 (14)
Major amputation	32	Time since last healthcare visit, mean(SD)		0.9 (1.5)	0	0.7 (1.1)
Major amputation	32	Total cholesterol, mean(SD)		4.8 (1.2)	19.5	4.8 (1.2)
Major amputation	33	Age at diagnosis of type 2 diabetes, mean(SD)		29.7 (2.6)	0	29.1 (2.7)
Major amputation	33	BMI, mean(SD)		34 (7.5)	13.1	34 (7.5)
Major amputation	33	HbA1c, mean(SD)		52.9 (17.9)	5.3	53 (17.1)
Major amputation	33	HDL-cholesterol, mean(SD)		1 (0.3)	23.1	1.1 (0.3)
Major amputation	33	Ischemic heart disease, n(%)	No	2585 (99.2%)	12.6	1734 (99.4%)
Major amputation	33	Ischemic heart disease, n(%)	Yes	21 (0.8%)	12.6	10 (0.6%)
Major amputation	33	Neuropathy, n(%)	No	2858 (95.9%)	0	1655 (94.9%)
Major amputation	33	Neuropathy, n(%)	Yes	123 (4.1%)	0	89 (5.1%)

Major amputation	33	Self-reported physical activity, n(%)	Never	327 (13.7%)	20.1	251 (14.4%)	
Major amputation	33	Self-reported physical activity, n(%)	<1 times/week	316 (13.3%)	20.1	224 (12.8%)	
Major amputation	33	Self-reported physical activity, n(%)	regularly - 1-2 times/week	502 (21.1%)	20.1	352 (20.2%)	
Major amputation	33	Self-reported physical activity, n(%)	regularly - 3-5 times/week	589 (24.7%)	20.1	434 (24.9%)	
Major amputation	33	Self-reported physical activity, n(%)	Daily	648 (27.2%)	20.1	483 (27.7%)	
Major amputation	33	Sex, n(%)	Males	1656 (55.6%)	0.1	989 (56.7%)	n=2981 vs n=1744
Major amputation	33	Sex, n(%)	Females	1321 (44.4%)	0.1	755 (43.3%)	
Major amputation	33	Smoking status, n(%)	No	1971 (76.4%)	13.4	1348 (77.3%)	
Major amputation	33	Smoking status, n(%)	Yes	610 (23.6%)	13.4	396 (22.7%)	
Major amputation	33	Stroke, n(%)	No	2587 (99.5%)	12.8	1734 (99.4%)	
Major amputation	33	Stroke, n(%)	Yes	13 (0.5%)	12.8	10 (0.6%)	
Major amputation	33	Systolic blood pressure, mean(SD)		125 (14.2)	7.4	125.1 (13.9)	
Major amputation	33	Time since last healthcare visit, mean(SD)		0.9 (1.4)	0	0.7 (1)	
Major amputation	33	Total cholesterol, mean(SD)		4.9 (1.2)	18.6	4.8 (1.2)	
Major amputation	34	Age at diagnosis of type 2 diabetes, mean(SD)		30.6 (2.6)	0	30.1 (2.6)	

Major amputation	34	BMI, mean(SD)		33.9 (7.5)	11.9	33.8 (7.4)	
Major amputation	34	HbA1c, mean(SD)		52.6 (17.4)	4.6	52.6 (16.7)	
Major amputation	34	HDL-cholesterol, mean(SD)		1.1 (0.3)	22.5	1.1 (0.3)	
Major amputation	34	Ischemic heart disease, n(%)	No	2972 (98.9%)	12	2008 (99.1%)	
Major amputation	34	Ischemic heart disease, n(%)	Yes	32 (1.1%)	12	19 (0.9%)	
Major amputation	34	Neuropathy, n(%)	No	3248 (95.2%)	0	1909 (94.2%)	
Major amputation	34	Neuropathy, n(%)	Yes	165 (4.8%)	0	118 (5.8%)	
Major amputation	34	Self-reported physical activity, n(%)	Never	371 (13.5%)	19.6	268 (13.2%)	
Major amputation	34	Self-reported physical activity, n(%)	<1 times/week	345 (12.6%)	19.6	254 (12.5%)	
Major amputation	34	Self-reported physical activity, n(%)	regularly - 1-2 times/week	595 (21.7%)	19.6	433 (21.4%)	
Major amputation	34	Self-reported physical activity, n(%)	regularly - 3-5 times/week	696 (25.4%)	19.6	518 (25.6%)	
Major amputation	34	Self-reported physical activity, n(%)	Daily	736 (26.8%)	19.6	554 (27.3%)	
Major amputation	34	Sex, n(%)	Males	1903 (55.9%)	0.2	1172 (57.8%)	n=3413 vs n=2027
Major amputation	34	Sex, n(%)	Females	1504 (44.1%)	0.2	855 (42.2%)	
Major amputation	34	Smoking status, n(%)	No	2308 (78.2%)	13.5	1605 (79.2%)	

Major amputation	34	Smoking status, n(%)	Yes	645 (21.8%)	13.5	422 (20.8%)
Major amputation	34	Stroke, n(%)	No	2983 (99.5%)	12.2	2016 (99.5%)
Major amputation	34	Stroke, n(%)	Yes	15 (0.5%)	12.2	11 (0.5%)
Major amputation	34	Systolic blood pressure, mean(SD)		125.5 (14.4)	7.5	125.4 (14.1)
Major amputation	34	Time since last healthcare visit, mean(SD)		0.9 (1.4)	0	0.7 (1)
Major amputation	34	Total cholesterol, mean(SD)		4.9 (1.1)	17.8	4.8 (1.1)
Major amputation	35	Age at diagnosis of type 2 diabetes, mean(SD)		31.6 (2.6)	0	31.1 (2.6)
Major amputation	35	BMI, mean(SD)		33.9 (7.4)	12	33.8 (7.4)
Major amputation	35	HbA1c, mean(SD)		52.6 (17.5)	4.5	52.6 (16.8)
Major amputation	35	HDL-cholesterol, mean(SD)		1.1 (0.3)	21.2	1.1 (0.3)
Major amputation	35	Ischemic heart disease, n(%)	No	3361 (99%)	12.3	2305 (98.9%)
Major amputation	35	Ischemic heart disease, n(%)	Yes	33 (1%)	12.3	25 (1.1%)
Major amputation	35	Neuropathy, n(%)	No	3676 (95%)	0	2182 (93.6%)
Major amputation	35	Neuropathy, n(%)	Yes	195 (5%)	0	148 (6.4%)
Major amputation	35	Self-reported physical activity, n(%)	Never	443 (14.2%)	19.5	324 (13.9%)

Major amputation	35	Self-reported physical activity, n(%)	<1 times/week	387 (12.4%)	19.5	280 (12%)	
Major amputation	35	Self-reported physical activity, n(%)	regularly - 1-2 times/week	671 (21.5%)	19.5	511 (21.9%)	
Major amputation	35	Self-reported physical activity, n(%)	regularly - 3-5 times/week	780 (25%)	19.5	582 (25%)	
Major amputation	35	Self-reported physical activity, n(%)	Daily	836 (26.8%)	19.5	633 (27.2%)	
Major amputation	35	Sex, n(%)	Males	2174 (56.2%)	0.2	1359 (58.3%)	n=3871 vs n=2330
Major amputation	35	Sex, n(%)	Females	1691 (43.8%)	0.2	971 (41.7%)	
Major amputation	35	Smoking status, n(%)	No	2660 (79.3%)	13.3	1869 (80.2%)	
Major amputation	35	Smoking status, n(%)	Yes	695 (20.7%)	13.3	461 (19.8%)	
Major amputation	35	Stroke, n(%)	No	3378 (99.6%)	12.4	2318 (99.5%)	
Major amputation	35	Stroke, n(%)	Yes	14 (0.4%)	12.4	12 (0.5%)	
Major amputation	35	Systolic blood pressure, mean(SD)		125.6 (14.5)	7.2	125.3 (14.2)	
Major amputation	35	Time since last healthcare visit, mean(SD)		0.9 (1.4)	0	0.7 (1)	
Major amputation	35	Total cholesterol, mean(SD)		4.9 (1.1)	16.9	4.8 (1.1)	
Major amputation	36	Age at diagnosis of type 2 diabetes, mean(SD)		32.6 (2.6)	0	32.2 (2.6)	
Major amputation	36	BMI, mean(SD)		33.7 (7.2)	11.3	33.6 (7.2)	

Major amputation	36	HbA1c, mean(SD)		52.6 (17.6)	4.1	52.9 (17)	
Major amputation	36	HDL-cholesterol, mean(SD)		1.1 (0.3)	20.9	1.1 (0.3)	
Major amputation	36	Ischemic heart disease, n(%)	No	3773 (98.8%)	12.6	2660 (98.9%)	
Major amputation	36	Ischemic heart disease, n(%)	Yes	45 (1.2%)	12.6	30 (1.1%)	
Major amputation	36	Neuropathy, n(%)	No	4143 (94.8%)	0	2512 (93.4%)	
Major amputation	36	Neuropathy, n(%)	Yes	227 (5.2%)	0	178 (6.6%)	
Major amputation	36	Self-reported physical activity, n(%)	Never	535 (15%)	18.4	394 (14.6%)	
Major amputation	36	Self-reported physical activity, n(%)	<1 times/week	450 (12.6%)	18.4	327 (12.2%)	
Major amputation	36	Self-reported physical activity, n(%)	regularly - 1-2 times/week	744 (20.9%)	18.4	581 (21.6%)	
Major amputation	36	Self-reported physical activity, n(%)	regularly - 3-5 times/week	879 (24.6%)	18.4	656 (24.4%)	
Major amputation	36	Self-reported physical activity, n(%)	Daily	958 (26.9%)	18.4	732 (27.2%)	
Major amputation	36	Sex, n(%)	Males	2477 (56.8%)	0.2	1610 (59.9%)	n=4370 vs n=2690
Major amputation	36	Sex, n(%)	Females	1885 (43.2%)	0.2	1080 (40.1%)	
Major amputation	36	Smoking status, n(%)	No	3037 (79%)	12	2162 (80.4%)	
Major amputation	36	Smoking status, n(%)	Yes	808 (21%)	12	528 (19.6%)	

Major amputation	36	Stroke, n(%)	No	3793 (99.5%)	12.8	2672 (99.3%)
Major amputation	36	Stroke, n(%)	Yes	19 (0.5%)	12.8	18 (0.7%)
Major amputation	36	Systolic blood pressure, mean(SD)		125.9 (14.4)	7.3	125.9 (14.1)
Major amputation	36	Time since last healthcare visit, mean(SD)		0.9 (1.4)	0	0.7 (1)
Major amputation	36	Total cholesterol, mean(SD)		4.9 (1.2)	16.5	4.9 (1.2)
Major amputation	37	Age at diagnosis of type 2 diabetes, mean(SD)		33.6 (2.6)	0	33.1 (2.6)
Major amputation	37	BMI, mean(SD)		33.6 (7.2)	10.5	33.5 (7.1)
Major amputation	37	HbA1c, mean(SD)		52.7 (17.5)	3.9	52.7 (16.9)
Major amputation	37	HDL-cholesterol, mean(SD)		1.1 (0.3)	20.4	1.1 (0.3)
Major amputation	37	Ischemic heart disease, n(%)	No	4357 (98.6%)	11.8	3064 (98.7%)
Major amputation	37	Ischemic heart disease, n(%)	Yes	64 (1.4%)	11.8	39 (1.3%)
Major amputation	37	Neuropathy, n(%)	No	4733 (94.4%)	0	2887 (93%)
Major amputation	37	Neuropathy, n(%)	Yes	281 (5.6%)	0	216 (7%)
Major amputation	37	Self-reported physical activity, n(%)	Never	602 (14.7%)	18.2	443 (14.3%)
Major amputation	37	Self-reported physical activity, n(%)	<1 times/week	556 (13.6%)	18.2	415 (13.4%)

Major amputation	37	Self-reported physical activity, n(%)	regularly - 1-2 times/week	840 (20.5%)	18.2	658 (21.2%)	
Major amputation	37	Self-reported physical activity, n(%)	regularly - 3-5 times/week	996 (24.3%)	18.2	742 (23.9%)	
Major amputation	37	Self-reported physical activity, n(%)	Daily	1107 (27%)	18.2	845 (27.2%)	
Major amputation	37	Sex, n(%)	Males	2856 (57.1%)	0.2	1865 (60.1%)	n=5014 vs n=3103
Major amputation	37	Sex, n(%)	Females	2149 (42.9%)	0.2	1238 (39.9%)	
Major amputation	37	Smoking status, n(%)	No	3528 (79.5%)	11.5	2508 (80.8%)	
Major amputation	37	Smoking status, n(%)	Yes	910 (20.5%)	11.5	595 (19.2%)	
Major amputation	37	Stroke, n(%)	No	4375 (99.2%)	12	3078 (99.2%)	
Major amputation	37	Stroke, n(%)	Yes	35 (0.8%)	12	25 (0.8%)	
Major amputation	37	Systolic blood pressure, mean(SD)		126.1 (14.2)	5.9	125.9 (13.8)	
Major amputation	37	Time since last healthcare visit, mean(SD)		0.9 (1.4)	0	0.7 (1)	
Major amputation	37	Total cholesterol, mean(SD)		4.9 (1.2)	16.1	4.8 (1.2)	
Major amputation	38	Age at diagnosis of type 2 diabetes, mean(SD)		34.5 (2.6)	0	34.1 (2.6)	
Major amputation	38	BMI, mean(SD)		33.5 (7.1)	11.2	33.4 (7)	
Major amputation	38	HbA1c, mean(SD)		52.4 (17.3)	4.1	52.7 (17)	

Major amputation	38	HDL-cholesterol, mean(SD)		1.1 (0.3)	19.5	1.1 (0.3)	
Major amputation	38	Ischemic heart disease, n(%)	No	5005 (98.3%)	11.5	3549 (98.3%)	
Major amputation	38	Ischemic heart disease, n(%)	Yes	85 (1.7%)	11.5	61 (1.7%)	
Major amputation	38	Neuropathy, n(%)	No	5428 (94.4%)	0	3355 (92.9%)	
Major amputation	38	Neuropathy, n(%)	Yes	325 (5.6%)	0	255 (7.1%)	
Major amputation	38	Self-reported physical activity, n(%)	Never	687 (14.6%)	18.4	523 (14.5%)	
Major amputation	38	Self-reported physical activity, n(%)	<1 times/week	612 (13%)	18.4	472 (13.1%)	
Major amputation	38	Self-reported physical activity, n(%)	regularly - 1-2 times/week	979 (20.9%)	18.4	779 (21.6%)	
Major amputation	38	Self-reported physical activity, n(%)	regularly - 3-5 times/week	1123 (23.9%)	18.4	837 (23.2%)	
Major amputation	38	Self-reported physical activity, n(%)	Daily	1291 (27.5%)	18.4	999 (27.7%)	
Major amputation	38	Sex, n(%)	Males	3306 (57.6%)	0.2	2170 (60.1%)	n=5753 vs n=3610
Major amputation	38	Sex, n(%)	Females	2437 (42.4%)	0.2	1440 (39.9%)	
Major amputation	38	Smoking status, n(%)	No	4040 (79.1%)	11.3	2903 (80.4%)	
Major amputation	38	Smoking status, n(%)	Yes	1065 (20.9%)	11.3	707 (19.6%)	
Major amputation	38	Stroke, n(%)	No	5035 (99.1%)	11.7	3582 (99.2%)	

Major amputation	38	Stroke, n(%)	Yes	45 (0.9%)	11.7	28 (0.8%)
Major amputation	38	Systolic blood pressure, mean(SD)		126.4 (14.6)	6.1	126.2 (14.1)
Major amputation	38	Time since last healthcare visit, mean(SD)		0.9 (1.4)	0	0.7 (1)
Major amputation	38	Total cholesterol, mean(SD)		4.9 (1.1)	15.1	4.8 (1.1)
Major amputation	39	Age at diagnosis of type 2 diabetes, mean(SD)		35.5 (2.7)	0	35 (2.7)
Major amputation	39	BMI, mean(SD)		33.4 (7)	10.5	33.2 (6.8)
Major amputation	39	HbA1c, mean(SD)		52.7 (17.5)	3.8	52.8 (17.1)
Major amputation	39	HDL-cholesterol, mean(SD)		1.1 (0.3)	19.5	1.1 (0.3)
Major amputation	39	Ischemic heart disease, n(%)	No	5842 (98.4%)	11.5	4190 (98.3%)
Major amputation	39	Ischemic heart disease, n(%)	Yes	94 (1.6%)	11.5	73 (1.7%)
Major amputation	39	Neuropathy, n(%)	No	6310 (94.1%)	0	3940 (92.4%)
Major amputation	39	Neuropathy, n(%)	Yes	398 (5.9%)	0	323 (7.6%)
Major amputation	39	Self-reported physical activity, n(%)	Never	760 (13.7%)	17.2	591 (13.9%)
Major amputation	39	Self-reported physical activity, n(%)	<1 times/week	721 (13%)	17.2	543 (12.7%)
Major amputation	39	Self-reported physical activity, n(%)	regularly - 1-2 times/week	1166 (21%)	17.2	888 (20.8%)

Major amputation	39	Self-reported physical activity, n(%)	regularly - 3-5 times/week	1405 (25.3%)	17.2	1086 (25.5%)	
Major amputation	39	Self-reported physical activity, n(%)	Daily	1503 (27.1%)	17.2	1155 (27.1%)	
Major amputation	39	Sex, n(%)	Males	3885 (58%)	0.1	2565 (60.2%)	n=6708 vs n=4263
Major amputation	39	Sex, n(%)	Females	2814 (42%)	0.1	1698 (39.8%)	
Major amputation	39	Smoking status, n(%)	No	4729 (79.1%)	10.9	3433 (80.5%)	
Major amputation	39	Smoking status, n(%)	Yes	1247 (20.9%)	10.9	830 (19.5%)	
Major amputation	39	Stroke, n(%)	No	5869 (99.1%)	11.7	4221 (99%)	
Major amputation	39	Stroke, n(%)	Yes	54 (0.9%)	11.7	42 (1%)	
Major amputation	39	Systolic blood pressure, mean(SD)		126.8 (14.6)	5.6	126.7 (14.3)	
Major amputation	39	Time since last healthcare visit, mean(SD)		0.8 (1.3)	0	0.7 (1)	
Major amputation	39	Total cholesterol, mean(SD)		4.9 (1.2)	15.2	4.8 (1.2)	
Major amputation	40	Age at diagnosis of type 2 diabetes, mean(SD)		36.5 (2.6)	0	36 (2.7)	
Major amputation	40	BMI, mean(SD)		33.4 (6.8)	10	33.3 (6.8)	
Major amputation	40	HbA1c, mean(SD)		52.7 (17.1)	3.7	52.8 (16.9)	
Major amputation	40	HDL-cholesterol, mean(SD)		1.1 (0.3)	18.8	1.1 (0.3)	

Major amputation	40	Ischemic heart disease, n(%)	No	6664 (98.4%)	11.1	4793 (98.3%)	
Major amputation	40	Ischemic heart disease, n(%)	Yes	110 (1.6%)	11.1	83 (1.7%)	
Major amputation	40	Neuropathy, n(%)	No	7163 (94.1%)	0	4503 (92.4%)	
Major amputation	40	Neuropathy, n(%)	Yes	453 (5.9%)	0	373 (7.6%)	
Major amputation	40	Self-reported physical activity, n(%)	Never	960 (15%)	16.2	709 (14.5%)	
Major amputation	40	Self-reported physical activity, n(%)	<1 times/week	894 (14%)	16.2	679 (13.9%)	
Major amputation	40	Self-reported physical activity, n(%)	regularly - 1-2 times/week	1337 (21%)	16.2	1045 (21.4%)	
Major amputation	40	Self-reported physical activity, n(%)	regularly - 3-5 times/week	1526 (23.9%)	16.2	1196 (24.5%)	
Major amputation	40	Self-reported physical activity, n(%)	Daily	1664 (26.1%)	16.2	1247 (25.6%)	
Major amputation	40	Sex, n(%)	Males	4465 (58.7%)	0.1	2944 (60.4%)	n=7616 vs n=4876
Major amputation	40	Sex, n(%)	Females	3143 (41.3%)	0.1	1932 (39.6%)	
Major amputation	40	Smoking status, n(%)	No	5346 (78.5%)	10.5	3895 (79.9%)	
Major amputation	40	Smoking status, n(%)	Yes	1468 (21.5%)	10.5	981 (20.1%)	
Major amputation	40	Stroke, n(%)	No	6693 (99.1%)	11.3	4824 (98.9%)	
Major amputation	40	Stroke, n(%)	Yes	63 (0.9%)	11.3	52 (1.1%)	

Major amputation	40	Systolic blood pressure, mean(SD)		127.2 (14.6)	5.6	127.1 (14.3)
Major amputation	40	Time since last healthcare visit, mean(SD)		0.8 (1.3)	0	0.7 (1)
Major amputation	40	Total cholesterol, mean(SD)		4.9 (1.1)	14.6	4.8 (1.1)
Major amputation	41	Age at diagnosis of type 2 diabetes, mean(SD)		37.5 (2.7)	0	37 (2.7)
Major amputation	41	BMI, mean(SD)		33.3 (6.9)	10	33.2 (6.8)
Major amputation	41	HbA1c, mean(SD)		52.7 (16.9)	3.3	52.8 (16.3)
Major amputation	41	HDL-cholesterol, mean(SD)		1.1 (0.3)	17.9	1.1 (0.3)
Major amputation	41	Ischemic heart disease, n(%)	No	7731 (98%)	10.9	5591 (98%)
Major amputation	41	Ischemic heart disease, n(%)	Yes	161 (2%)	10.9	114 (2%)
Major amputation	41	Neuropathy, n(%)	No	8301 (93.7%)	0	5241 (91.9%)
Major amputation	41	Neuropathy, n(%)	Yes	554 (6.3%)	0	464 (8.1%)
Major amputation	41	Self-reported physical activity, n(%)	Never	1137 (15.3%)	15.9	849 (14.9%)
Major amputation	41	Self-reported physical activity, n(%)	<1 times/week	999 (13.4%)	15.9	748 (13.1%)
Major amputation	41	Self-reported physical activity, n(%)	regularly - 1-2 times/week	1591 (21.4%)	15.9	1239 (21.7%)
Major amputation	41	Self-reported physical activity, n(%)	regularly - 3-5 times/week	1796 (24.1%)	15.9	1416 (24.8%)

Major amputation	41	Self-reported physical activity, n(%)	Daily	1924 (25.8%)	15.9	1453 (25.5%)	
Major amputation	41	Sex, n(%)	Males	5237 (59.2%)	0.1	3474 (60.9%)	n=8855 vs n=5705
Major amputation	41	Sex, n(%)	Females	3607 (40.8%)	0.1	2231 (39.1%)	
Major amputation	41	Smoking status, n(%)	No	6240 (78.4%)	10.1	4555 (79.8%)	
Major amputation	41	Smoking status, n(%)	Yes	1722 (21.6%)	10.1	1150 (20.2%)	
Major amputation	41	Stroke, n(%)	No	7789 (99%)	11.1	5642 (98.9%)	
Major amputation	41	Stroke, n(%)	Yes	79 (1%)	11.1	63 (1.1%)	
Major amputation	41	Systolic blood pressure, mean(SD)		127.7 (14.5)	5	127.6 (14.3)	
Major amputation	41	Time since last healthcare visit, mean(SD)		0.8 (1.3)	0	0.7 (1)	
Major amputation	41	Total cholesterol, mean(SD)		4.9 (1.1)	13.7	4.9 (1.2)	
Major amputation	42	Age at diagnosis of type 2 diabetes, mean(SD)		38.4 (2.7)	0	37.9 (2.7)	
Major amputation	42	BMI, mean(SD)		33.2 (6.7)	9.6	33.1 (6.6)	
Major amputation	42	HbA1c, mean(SD)		52.5 (16.6)	3.1	52.8 (16.2)	
Major amputation	42	HDL-cholesterol, mean(SD)		1.1 (0.3)	17.6	1.1 (0.3)	
Major amputation	42	Ischemic heart disease, n(%)	No	8752 (97.8%)	11.2	6408 (97.8%)	

Major amputation	42	Ischemic heart disease, n(%)	Yes	200 (2.2%)	11.2	145 (2.2%)	
Major amputation	42	Neuropathy, n(%)	No	9455 (93.8%)	0	6037 (92.1%)	
Major amputation	42	Neuropathy, n(%)	Yes	621 (6.2%)	0	516 (7.9%)	
Major amputation	42	Self-reported physical activity, n(%)	Never	1289 (15.2%)	15.8	958 (14.6%)	
Major amputation	42	Self-reported physical activity, n(%)	<1 times/week	1124 (13.3%)	15.8	859 (13.1%)	
Major amputation	42	Self-reported physical activity, n(%)	regularly - 1-2 times/week	1763 (20.8%)	15.8	1397 (21.3%)	
Major amputation	42	Self-reported physical activity, n(%)	regularly - 3-5 times/week	2061 (24.3%)	15.8	1599 (24.4%)	
Major amputation	42	Self-reported physical activity, n(%)	Daily	2245 (26.5%)	15.8	1740 (26.6%)	
Major amputation	42	Sex, n(%)	Males	6007 (59.7%)	0.1	3999 (61%)	n=10076 vs n=6553
Major amputation	42	Sex, n(%)	Females	4058 (40.3%)	0.1	2554 (39%)	
Major amputation	42	Smoking status, n(%)	No	7106 (78.4%)	10	5209 (79.5%)	
Major amputation	42	Smoking status, n(%)	Yes	1958 (21.6%)	10	1344 (20.5%)	
Major amputation	42	Stroke, n(%)	No	8827 (98.7%)	11.3	6471 (98.7%)	
Major amputation	42	Stroke, n(%)	Yes	112 (1.3%)	11.3	82 (1.3%)	
Major amputation	42	Systolic blood pressure, mean(SD)		128.1 (14.7)	5.2	127.9 (14.4)	

Major amputation	42	Time since last healthcare visit, mean(SD)		0.8 (1.3)	0	0.7 (1)
Major amputation	42	Total cholesterol, mean(SD)		4.9 (1.2)	13.2	4.8 (1.2)
Major amputation	43	Age at diagnosis of type 2 diabetes, mean(SD)		39.4 (2.7)	0	38.9 (2.7)
Major amputation	43	BMI, mean(SD)		33.1 (6.6)	9.2	33 (6.6)
Major amputation	43	HbA1c, mean(SD)		52.6 (16.3)	3.2	52.8 (16)
Major amputation	43	HDL-cholesterol, mean(SD)		1.1 (0.3)	17.5	1.1 (0.3)
Major amputation	43	Ischemic heart disease, n(%)	No	9883 (97.4%)	10.7	7301 (97.5%)
Major amputation	43	Ischemic heart disease, n(%)	Yes	259 (2.6%)	10.7	188 (2.5%)
Major amputation	43	Neuropathy, n(%)	No	10605 (93.3%)	0	6848 (91.4%)
Major amputation	43	Neuropathy, n(%)	Yes	758 (6.7%)	0	641 (8.6%)
Major amputation	43	Self-reported physical activity, n(%)	Never	1429 (14.9%)	15.7	1099 (14.7%)
Major amputation	43	Self-reported physical activity, n(%)	<1 times/week	1298 (13.6%)	15.7	996 (13.3%)
Major amputation	43	Self-reported physical activity, n(%)	regularly - 1-2 times/week	1980 (20.7%)	15.7	1563 (20.9%)
Major amputation	43	Self-reported physical activity, n(%)	regularly - 3-5 times/week	2286 (23.9%)	15.7	1792 (23.9%)
Major amputation	43	Self-reported physical activity, n(%)	Daily	2583 (27%)	15.7	2039 (27.2%)

Major amputation	43	Sex, n(%)	Males	6874 (60.6%)	0.1	4645 (62%)	n=11363 vs n=7489
Major amputation	43	Sex, n(%)	Females	4476 (39.4%)	0.1	2844 (38%)	
Major amputation	43	Smoking status, n(%)	No	8065 (78.4%)	9.4	5938 (79.3%)	
Major amputation	43	Smoking status, n(%)	Yes	2226 (21.6%)	9.4	1551 (20.7%)	
Major amputation	43	Stroke, n(%)	No	9981 (98.7%)	11	7387 (98.6%)	
Major amputation	43	Stroke, n(%)	Yes	133 (1.3%)	11	102 (1.4%)	
Major amputation	43	Systolic blood pressure, mean(SD)		128.7 (14.8)	4.8	128.5 (14.5)	
Major amputation	43	Time since last healthcare visit, mean(SD)		0.8 (1.3)	0	0.7 (1)	
Major amputation	43	Total cholesterol, mean(SD)		4.9 (1.2)	13.2	4.8 (1.2)	
Major amputation	44	Age at diagnosis of type 2 diabetes, mean(SD)		40.4 (2.7)	0	39.9 (2.7)	
Major amputation	44	BMI, mean(SD)		33 (6.6)	9.1	32.9 (6.5)	
Major amputation	44	HbA1c, mean(SD)		52.7 (16.4)	3	52.8 (16)	
Major amputation	44	HDL-cholesterol, mean(SD)		1.1 (0.3)	17.5	1.1 (0.3)	
Major amputation	44	Ischemic heart disease, n(%)	No	11164 (96.9%)	10.9	8241 (96.9%)	
Major amputation	44	Ischemic heart disease, n(%)	Yes	363 (3.1%)	10.9	265 (3.1%)	

Major amputation	44	Neuropathy, n(%)	No	12018 (92.8%)	0	7731 (90.9%)	
Major amputation	44	Neuropathy, n(%)	Yes	926 (7.2%)	0	775 (9.1%)	
Major amputation	44	Self-reported physical activity, n(%)	Never	1603 (14.7%)	15.7	1221 (14.4%)	
Major amputation	44	Self-reported physical activity, n(%)	<1 times/week	1506 (13.8%)	15.7	1151 (13.5%)	
Major amputation	44	Self-reported physical activity, n(%)	regularly - 1-2 times/week	2242 (20.5%)	15.7	1752 (20.6%)	
Major amputation	44	Self-reported physical activity, n(%)	regularly - 3-5 times/week	2688 (24.6%)	15.7	2109 (24.8%)	
Major amputation	44	Self-reported physical activity, n(%)	Daily	2878 (26.4%)	15.7	2273 (26.7%)	
Major amputation	44	Sex, n(%)	Males	7879 (60.9%)	0.1	5307 (62.4%)	n=12944 vs n=8506
Major amputation	44	Sex, n(%)	Females	5053 (39.1%)	0.1	3199 (37.6%)	
Major amputation	44	Smoking status, n(%)	No	9185 (78.4%)	9.5	6743 (79.3%)	
Major amputation	44	Smoking status, n(%)	Yes	2528 (21.6%)	9.5	1763 (20.7%)	
Major amputation	44	Stroke, n(%)	No	11337 (98.6%)	11.2	8387 (98.6%)	
Major amputation	44	Stroke, n(%)	Yes	160 (1.4%)	11.2	119 (1.4%)	
Major amputation	44	Systolic blood pressure, mean(SD)		128.8 (14.6)	4.8	128.5 (14.4)	
Major amputation	44	Time since last healthcare visit, mean(SD)		0.8 (1.3)	0	0.7 (1)	

Major amputation	44	Total cholesterol, mean(SD)		4.9 (1.2)	13.3	4.8 (1.2)	
Major amputation	45	Age at diagnosis of type 2 diabetes, mean(SD)		41.4 (2.7)	0	40.9 (2.7)	
Major amputation	45	BMI, mean(SD)		33 (6.5)	9.3	32.9 (6.5)	
Major amputation	45	HbA1c, mean(SD)		52.6 (16)	3	52.6 (15.6)	
Major amputation	45	HDL-cholesterol, mean(SD)		1.1 (0.3)	16.7	1.1 (0.3)	
Major amputation	45	Ischemic heart disease, n(%)	No	12665 (96.3%)	10.7	9369 (96.4%)	
Major amputation	45	Ischemic heart disease, n(%)	Yes	491 (3.7%)	10.7	350 (3.6%)	
Major amputation	45	Neuropathy, n(%)	No	13618 (92.5%)	0	8791 (90.5%)	
Major amputation	45	Neuropathy, n(%)	Yes	1110 (7.5%)	0	928 (9.5%)	
Major amputation	45	Self-reported physical activity, n(%)	Never	1823 (14.6%)	15.5	1403 (14.4%)	
Major amputation	45	Self-reported physical activity, n(%)	<1 times/week	1673 (13.4%)	15.5	1268 (13%)	
Major amputation	45	Self-reported physical activity, n(%)	regularly - 1-2 times/week	2611 (21%)	15.5	2046 (21.1%)	
Major amputation	45	Self-reported physical activity, n(%)	regularly - 3-5 times/week	3072 (24.7%)	15.5	2429 (25%)	
Major amputation	45	Self-reported physical activity, n(%)	Daily	3270 (26.3%)	15.5	2573 (26.5%)	
Major amputation	45	Sex, n(%)	Males	9120 (62%)	0.1	6079 (62.5%)	n=14728 vs n=9719

Major amputation	45	Sex, n(%)	Females	5597 (38%)	0.1	3640 (37.5%)
Major amputation	45	Smoking status, n(%)	No	10457 (78.4%)	9.4	7717 (79.4%)
Major amputation	45	Smoking status, n(%)	Yes	2883 (21.6%)	9.4	2002 (20.6%)
Major amputation	45	Stroke, n(%)	No	12915 (98.5%)	11	9579 (98.6%)
Major amputation	45	Stroke, n(%)	Yes	195 (1.5%)	11	140 (1.4%)
Major amputation	45	Systolic blood pressure, mean(SD)		129.4 (14.8)	4.5	129.1 (14.5)
Major amputation	45	Time since last healthcare visit, mean(SD)		0.8 (1.3)	0	0.7 (1)
Major amputation	45	Total cholesterol, mean(SD)		4.9 (1.2)	12.6	4.8 (1.1)
Major amputation	46	Age at diagnosis of type 2 diabetes, mean(SD)		42.4 (2.7)	0	41.9 (2.7)
Major amputation	46	BMI, mean(SD)		32.9 (6.4)	9.1	32.8 (6.3)
Major amputation	46	HbA1c, mean(SD)		52.6 (16)	2.8	52.7 (15.6)
Major amputation	46	HDL-cholesterol, mean(SD)		1.1 (0.3)	16.3	1.1 (0.3)
Major amputation	46	Ischemic heart disease, n(%)	No	14246 (95.7%)	10.4	10588 (95.8%)
Major amputation	46	Ischemic heart disease, n(%)	Yes	634 (4.3%)	10.4	465 (4.2%)
Major amputation	46	Neuropathy, n(%)	No	15304 (92.1%)	0	9954 (90.1%)

Major amputation	46	Neuropathy, n(%)	Yes	1305 (7.9%)	0	1099 (9.9%)	
Major amputation	46	Self-reported physical activity, n(%)	Never	2077 (14.7%)	15.1	1611 (14.6%)	
Major amputation	46	Self-reported physical activity, n(%)	<1 times/week	1946 (13.8%)	15.1	1491 (13.5%)	
Major amputation	46	Self-reported physical activity, n(%)	regularly - 1-2 times/week	2992 (21.2%)	15.1	2361 (21.4%)	
Major amputation	46	Self-reported physical activity, n(%)	regularly - 3-5 times/week	3459 (24.5%)	15.1	2741 (24.8%)	
Major amputation	46	Self-reported physical activity, n(%)	Daily	3629 (25.7%)	15.1	2849 (25.8%)	
Major amputation	46	Sex, n(%)	Males	10386 (62.6%)	0.1	6988 (63.2%)	n=16609 vs n=11053
Major amputation	46	Sex, n(%)	Females	6211 (37.4%)	0.1	4065 (36.8%)	
Major amputation	46	Smoking status, n(%)	No	11812 (78.3%)	9.1	8770 (79.3%)	
Major amputation	46	Smoking status, n(%)	Yes	3283 (21.7%)	9.1	2283 (20.7%)	
Major amputation	46	Stroke, n(%)	No	14558 (98.2%)	10.8	10855 (98.2%)	
Major amputation	46	Stroke, n(%)	Yes	265 (1.8%)	10.8	198 (1.8%)	
Major amputation	46	Systolic blood pressure, mean(SD)		129.7 (14.8)	4.3	129.4 (14.5)	
Major amputation	46	Time since last healthcare visit, mean(SD)		0.8 (1.3)	0	0.6 (1)	
Major amputation	46	Total cholesterol, mean(SD)		4.9 (1.2)	11.9	4.8 (1.1)	

Major amputation	47	Age at diagnosis of type 2 diabetes, mean(SD)		43.3 (2.7)	0	42.8 (2.7)	
Major amputation	47	BMI, mean(SD)		32.8 (6.3)	8.9	32.7 (6.3)	
Major amputation	47	HbA1c, mean(SD)		52.5 (15.7)	2.5	52.6 (15.3)	
Major amputation	47	HDL-cholesterol, mean(SD)		1.1 (0.3)	15.7	1.1 (0.3)	
Major amputation	47	Ischemic heart disease, n(%)	No	15937 (95.2%)	10	11962 (95.5%)	
Major amputation	47	Ischemic heart disease, n(%)	Yes	798 (4.8%)	10	560 (4.5%)	
Major amputation	47	Neuropathy, n(%)	No	17104 (92%)	0	11262 (89.9%)	
Major amputation	47	Neuropathy, n(%)	Yes	1497 (8%)	0	1260 (10.1%)	
Major amputation	47	Self-reported physical activity, n(%)	Never	2303 (14.5%)	14.8	1811 (14.5%)	
Major amputation	47	Self-reported physical activity, n(%)	<1 times/week	2191 (13.8%)	14.8	1680 (13.4%)	
Major amputation	47	Self-reported physical activity, n(%)	regularly - 1-2 times/week	3340 (21.1%)	14.8	2638 (21.1%)	
Major amputation	47	Self-reported physical activity, n(%)	regularly - 3-5 times/week	3875 (24.4%)	14.8	3095 (24.7%)	
Major amputation	47	Self-reported physical activity, n(%)	Daily	4140 (26.1%)	14.8	3298 (26.3%)	
Major amputation	47	Sex, n(%)	Males	11708 (63%)	0.1	7994 (63.8%)	n=18601 vs n=12522
Major amputation	47	Sex, n(%)	Females	6880 (37%)	0.1	4528 (36.2%)	

Major amputation	47	Smoking status, n(%)	No	13248 (78.1%)	8.8	9908 (79.1%)
Major amputation	47	Smoking status, n(%)	Yes	3709 (21.9%)	8.8	2614 (20.9%)
Major amputation	47	Stroke, n(%)	No	16353 (98%)	10.3	12283 (98.1%)
Major amputation	47	Stroke, n(%)	Yes	329 (2%)	10.3	239 (1.9%)
Major amputation	47	Systolic blood pressure, mean(SD)		129.9 (14.8)	4	129.5 (14.5)
Major amputation	47	Time since last healthcare visit, mean(SD)		0.8 (1.3)	0	0.7 (1)
Major amputation	47	Total cholesterol, mean(SD)		4.9 (1.1)	11.3	4.8 (1.1)
Major amputation	48	Age at diagnosis of type 2 diabetes, mean(SD)		44.2 (2.7)	0	43.7 (2.7)
Major amputation	48	BMI, mean(SD)		32.7 (6.3)	8.6	32.6 (6.2)
Major amputation	48	HbA1c, mean(SD)		52.5 (15.5)	2.5	52.6 (15.2)
Major amputation	48	HDL-cholesterol, mean(SD)		1.1 (0.3)	15.6	1.1 (0.3)
Major amputation	48	Ischemic heart disease, n(%)	No	17529 (95%)	9.9	13261 (95.3%)
Major amputation	48	Ischemic heart disease, n(%)	Yes	927 (5%)	9.9	660 (4.7%)
Major amputation	48	Neuropathy, n(%)	No	18734 (91.4%)	0	12464 (89.5%)
Major amputation	48	Neuropathy, n(%)	Yes	1759 (8.6%)	0	1457 (10.5%)

Major amputation	48	Self-reported physical activity, n(%)	Never	2554 (14.5%)	14.3	2037 (14.6%)	
Major amputation	48	Self-reported physical activity, n(%)	<1 times/week	2404 (13.7%)	14.3	1821 (13.1%)	
Major amputation	48	Self-reported physical activity, n(%)	regularly - 1-2 times/week	3719 (21.2%)	14.3	2947 (21.2%)	
Major amputation	48	Self-reported physical activity, n(%)	regularly - 3-5 times/week	4309 (24.5%)	14.3	3460 (24.9%)	
Major amputation	48	Self-reported physical activity, n(%)	Daily	4580 (26.1%)	14.3	3656 (26.3%)	
Major amputation	48	Sex, n(%)	Males	12858 (62.8%)	0.1	8879 (63.8%)	n=20493 vs n=13921
Major amputation	48	Sex, n(%)	Females	7620 (37.2%)	0.1	5042 (36.2%)	
Major amputation	48	Smoking status, n(%)	No	14725 (78.4%)	8.4	11059 (79.4%)	
Major amputation	48	Smoking status, n(%)	Yes	4050 (21.6%)	8.4	2862 (20.6%)	
Major amputation	48	Stroke, n(%)	No	18029 (98%)	10.2	13647 (98%)	
Major amputation	48	Stroke, n(%)	Yes	376 (2%)	10.2	274 (2%)	
Major amputation	48	Systolic blood pressure, mean(SD)		130.4 (14.7)	3.9	130.2 (14.4)	
Major amputation	48	Time since last healthcare visit, mean(SD)		0.8 (1.2)	0	0.6 (1)	
Major amputation	48	Total cholesterol, mean(SD)		4.9 (1.1)	11.4	4.8 (1.1)	
Major amputation	49	Age at diagnosis of type 2 diabetes, mean(SD)		45.2 (2.7)	0	44.7 (2.7)	

Major amputation	49	BMI, mean(SD)		32.5 (6.1)	8.7	32.4 (6)	
Major amputation	49	HbA1c, mean(SD)		52.5 (15.5)	2.5	52.5 (15)	
Major amputation	49	HDL-cholesterol, mean(SD)		1.1 (0.3)	15.5	1.1 (0.3)	
Major amputation	49	Ischemic heart disease, n(%)	No	19467 (94.5%)	9.5	14773 (94.7%)	
Major amputation	49	Ischemic heart disease, n(%)	Yes	1142 (5.5%)	9.5	819 (5.3%)	
Major amputation	49	Neuropathy, n(%)	No	20748 (91.1%)	0	13897 (89.1%)	
Major amputation	49	Neuropathy, n(%)	Yes	2035 (8.9%)	0	1695 (10.9%)	
Major amputation	49	Self-reported physical activity, n(%)	Never	2884 (14.7%)	13.6	2310 (14.8%)	
Major amputation	49	Self-reported physical activity, n(%)	<1 times/week	2650 (13.5%)	13.6	2016 (12.9%)	
Major amputation	49	Self-reported physical activity, n(%)	regularly - 1-2 times/week	4126 (21%)	13.6	3270 (21%)	
Major amputation	49	Self-reported physical activity, n(%)	regularly - 3-5 times/week	4701 (23.9%)	13.6	3757 (24.1%)	
Major amputation	49	Self-reported physical activity, n(%)	Daily	5313 (27%)	13.6	4239 (27.2%)	
Major amputation	49	Sex, n(%)	Males	14319 (62.9%)	0.1	9971 (63.9%)	n=22783 vs n=15592
Major amputation	49	Sex, n(%)	Females	8450 (37.1%)	0.1	5621 (36.1%)	
Major amputation	49	Smoking status, n(%)	No	16284 (78%)	8.3	12332 (79.1%)	

Major amputation	49	Smoking status, n(%)	Yes	4601 (22%)	8.3	3260 (20.9%)	
Major amputation	49	Stroke, n(%)	No	20095 (97.9%)	9.9	15290 (98.1%)	
Major amputation	49	Stroke, n(%)	Yes	432 (2.1%)	9.9	302 (1.9%)	
Major amputation	49	Systolic blood pressure, mean(SD)		130.9 (14.8)	3.8	130.5 (14.5)	
Major amputation	49	Time since last healthcare visit, mean(SD)		0.8 (1.2)	0	0.6 (1)	
Major amputation	49	Total cholesterol, mean(SD)		4.9 (1.2)	11.2	4.8 (1.1)	
Major amputation	50	Age at diagnosis of type 2 diabetes, mean(SD)		46.2 (2.8)	0	45.7 (2.8)	
Major amputation	50	BMI, mean(SD)		32.4 (6.1)	8.8	32.3 (6)	
Major amputation	50	HbA1c, mean(SD)		52.4 (15.2)	2.3	52.5 (14.8)	
Major amputation	50	HDL-cholesterol, mean(SD)		1.1 (0.3)	15.2	1.1 (0.3)	
Major amputation	50	Ischemic heart disease, n(%)	No	21546 (93.9%)	9.6	16399 (94.2%)	
Major amputation	50	Ischemic heart disease, n(%)	Yes	1388 (6.1%)	9.6	1007 (5.8%)	
Major amputation	50	Neuropathy, n(%)	No	23000 (90.7%)	0	15428 (88.6%)	
Major amputation	50	Neuropathy, n(%)	Yes	2368 (9.3%)	0	1978 (11.4%)	
Major amputation	50	Self-reported physical activity, n(%)	Never	3216 (14.7%)	13.7	2581 (14.8%)	

Major amputation	50	Self-reported physical activity, n(%)	<1 times/week	2976 (13.6%)	13.7	2294 (13.2%)	
Major amputation	50	Self-reported physical activity, n(%)	regularly - 1-2 times/week	4490 (20.5%)	13.7	3576 (20.5%)	
Major amputation	50	Self-reported physical activity, n(%)	regularly - 3-5 times/week	5289 (24.2%)	13.7	4230 (24.3%)	
Major amputation	50	Self-reported physical activity, n(%)	Daily	5922 (27%)	13.7	4725 (27.1%)	
Major amputation	50	Sex, n(%)	Males	15979 (63%)	0.1	11150 (64.1%)	n=25368 vs n=17406
Major amputation	50	Sex, n(%)	Females	9373 (37%)	0.1	6256 (35.9%)	
Major amputation	50	Smoking status, n(%)	No	18139 (78%)	8.3	13736 (78.9%)	
Major amputation	50	Smoking status, n(%)	Yes	5131 (22%)	8.3	3670 (21.1%)	
Major amputation	50	Stroke, n(%)	No	22355 (97.8%)	9.9	17040 (97.9%)	
Major amputation	50	Stroke, n(%)	Yes	513 (2.2%)	9.9	366 (2.1%)	
Major amputation	50	Systolic blood pressure, mean(SD)		131.4 (14.9)	3.7	131 (14.6)	
Major amputation	50	Time since last healthcare visit, mean(SD)		0.7 (1.2)	0	0.6 (1)	
Major amputation	50	Total cholesterol, mean(SD)		4.9 (1.2)	11	4.8 (1.1)	
Major amputation	51	Age at diagnosis of type 2 diabetes, mean(SD)		47.2 (2.8)	0	46.6 (2.8)	
Major amputation	51	BMI, mean(SD)		32.3 (6)	8.4	32.2 (5.9)	

Major amputation	51	HbA1c, mean(SD)		52.4 (14.9)	2.2	52.4 (14.5)	
Major amputation	51	HDL-cholesterol, mean(SD)		1.1 (0.3)	14.3	1.1 (0.3)	
Major amputation	51	Ischemic heart disease, n(%)	No	23678 (93.4%)	9.2	18188 (93.7%)	
Major amputation	51	Ischemic heart disease, n(%)	Yes	1681 (6.6%)	9.2	1229 (6.3%)	
Major amputation	51	Neuropathy, n(%)	No	25113 (89.9%)	0	17051 (87.8%)	
Major amputation	51	Neuropathy, n(%)	Yes	2818 (10.1%)	0	2366 (12.2%)	
Major amputation	51	Self-reported physical activity, n(%)	Never	3520 (14.5%)	13.3	2827 (14.6%)	
Major amputation	51	Self-reported physical activity, n(%)	<1 times/week	3304 (13.6%)	13.3	2592 (13.3%)	
Major amputation	51	Self-reported physical activity, n(%)	regularly - 1-2 times/week	4948 (20.4%)	13.3	3963 (20.4%)	
Major amputation	51	Self-reported physical activity, n(%)	regularly - 3-5 times/week	5971 (24.7%)	13.3	4848 (25%)	
Major amputation	51	Self-reported physical activity, n(%)	Daily	6475 (26.7%)	13.3	5187 (26.7%)	
Major amputation	51	Sex, n(%)	Males	17570 (63%)	0.1	12398 (63.9%)	n=27931 vs n=19417
Major amputation	51	Sex, n(%)	Females	10336 (37%)	0.1	7019 (36.1%)	
Major amputation	51	Smoking status, n(%)	No	19996 (77.8%)	8	15285 (78.7%)	
Major amputation	51	Smoking status, n(%)	Yes	5707 (22.2%)	8	4132 (21.3%)	

Major amputation	51	Stroke, n(%)	No	24680 (97.6%)	9.5	18975 (97.7%)
Major amputation	51	Stroke, n(%)	Yes	607 (2.4%)	9.5	442 (2.3%)
Major amputation	51	Systolic blood pressure, mean(SD)		131.7 (15)	3.4	131.4 (14.7)
Major amputation	51	Time since last healthcare visit, mean(SD)		0.7 (1.2)	0	0.6 (0.9)
Major amputation	51	Total cholesterol, mean(SD)		4.9 (1.2)	10.4	4.8 (1.1)
Major amputation	52	Age at diagnosis of type 2 diabetes, mean(SD)		48.1 (2.8)	0	47.6 (2.8)
Major amputation	52	BMI, mean(SD)		32.2 (5.9)	8.3	32.1 (5.8)
Major amputation	52	HbA1c, mean(SD)		52.5 (14.9)	2.2	52.5 (14.4)
Major amputation	52	HDL-cholesterol, mean(SD)		1.1 (0.3)	14	1.2 (0.3)
Major amputation	52	Ischemic heart disease, n(%)	No	25479 (93.1%)	9.1	19662 (93.4%)
Major amputation	52	Ischemic heart disease, n(%)	Yes	1901 (6.9%)	9.1	1400 (6.6%)
Major amputation	52	Neuropathy, n(%)	No	26930 (89.4%)	0	18355 (87.1%)
Major amputation	52	Neuropathy, n(%)	Yes	3189 (10.6%)	0	2707 (12.9%)
Major amputation	52	Self-reported physical activity, n(%)	Never	3801 (14.5%)	12.9	3043 (14.4%)
Major amputation	52	Self-reported physical activity, n(%)	<1 times/week	3535 (13.5%)	12.9	2765 (13.1%)

Major amputation	52	Self-reported physical activity, n(%)	regularly - 1-2 times/week	5349 (20.4%)	12.9	4245 (20.2%)	
Major amputation	52	Self-reported physical activity, n(%)	regularly - 3-5 times/week	6333 (24.2%)	12.9	5164 (24.5%)	
Major amputation	52	Self-reported physical activity, n(%)	Daily	7203 (27.5%)	12.9	5845 (27.8%)	
Major amputation	52	Sex, n(%)	Males	18899 (62.8%)	0.1	13356 (63.4%)	n=30119 vs n=21062
Major amputation	52	Sex, n(%)	Females	11196 (37.2%)	0.1	7706 (36.6%)	
Major amputation	52	Smoking status, n(%)	No	21529 (77.5%)	7.8	16520 (78.4%)	
Major amputation	52	Smoking status, n(%)	Yes	6239 (22.5%)	7.8	4542 (21.6%)	
Major amputation	52	Stroke, n(%)	No	26563 (97.4%)	9.5	20546 (97.6%)	
Major amputation	52	Stroke, n(%)	Yes	709 (2.6%)	9.5	516 (2.4%)	
Major amputation	52	Systolic blood pressure, mean(SD)		132 (15)	3.3	131.8 (14.7)	
Major amputation	52	Time since last healthcare visit, mean(SD)		0.7 (1.2)	0	0.6 (0.9)	
Major amputation	52	Total cholesterol, mean(SD)		4.9 (1.2)	10.1	4.8 (1.1)	
Major amputation	53	Age at diagnosis of type 2 diabetes, mean(SD)		49.1 (2.8)	0	48.5 (2.8)	
Major amputation	53	BMI, mean(SD)		32 (5.9)	8.4	31.9 (5.8)	
Major amputation	53	HbA1c, mean(SD)		52.3 (14.7)	2.3	52.4 (14.4)	

Major amputation	53	HDL-cholesterol, mean(SD)		1.2 (0.3)	14.3	1.2 (0.3)	
Major amputation	53	Ischemic heart disease, n(%)	No	27006 (92.5%)	9.1	20887 (92.8%)	
Major amputation	53	Ischemic heart disease, n(%)	Yes	2191 (7.5%)	9.1	1625 (7.2%)	
Major amputation	53	Neuropathy, n(%)	No	28511 (88.7%)	0	19437 (86.3%)	
Major amputation	53	Neuropathy, n(%)	Yes	3618 (11.3%)	0	3075 (13.7%)	
Major amputation	53	Self-reported physical activity, n(%)	Never	3995 (14.3%)	12.9	3190 (14.2%)	
Major amputation	53	Self-reported physical activity, n(%)	<1 times/week	3722 (13.3%)	12.9	2956 (13.1%)	
Major amputation	53	Self-reported physical activity, n(%)	regularly - 1-2 times/week	5735 (20.5%)	12.9	4595 (20.4%)	
Major amputation	53	Self-reported physical activity, n(%)	regularly - 3-5 times/week	6757 (24.2%)	12.9	5523 (24.5%)	
Major amputation	53	Self-reported physical activity, n(%)	Daily	7762 (27.8%)	12.9	6248 (27.8%)	
Major amputation	53	Sex, n(%)	Males	20078 (62.5%)	0.1	14235 (63.2%)	n=32129 vs n=22512
Major amputation	53	Sex, n(%)	Females	12023 (37.5%)	0.1	8277 (36.8%)	
Major amputation	53	Smoking status, n(%)	No	22884 (77.4%)	8	17630 (78.3%)	
Major amputation	53	Smoking status, n(%)	Yes	6674 (22.6%)	8	4882 (21.7%)	
Major amputation	53	Stroke, n(%)	No	28270 (97.2%)	9.4	21899 (97.3%)	

Major amputation	53	Stroke, n(%)	Yes	829 (2.8%)	9.4	613 (2.7%)
Major amputation	53	Systolic blood pressure, mean(SD)		132.2 (14.9)	3.4	131.9 (14.6)
Major amputation	53	Time since last healthcare visit, mean(SD)		0.7 (1.2)	0	0.6 (0.9)
Major amputation	53	Total cholesterol, mean(SD)		4.9 (1.2)	10.4	4.8 (1.2)
Major amputation	54	Age at diagnosis of type 2 diabetes, mean(SD)		50 (2.8)	0	49.5 (2.8)
Major amputation	54	BMI, mean(SD)		31.9 (5.8)	8.2	31.8 (5.7)
Major amputation	54	HbA1c, mean(SD)		52.4 (14.6)	2.4	52.4 (14.2)
Major amputation	54	HDL-cholesterol, mean(SD)		1.2 (0.3)	14.1	1.2 (0.3)
Major amputation	54	Ischemic heart disease, n(%)	No	28526 (91.8%)	8.8	22104 (92.1%)
Major amputation	54	Ischemic heart disease, n(%)	Yes	2538 (8.2%)	8.8	1908 (7.9%)
Major amputation	54	Neuropathy, n(%)	No	30078 (88.3%)	0	20639 (86%)
Major amputation	54	Neuropathy, n(%)	Yes	3997 (11.7%)	0	3373 (14%)
Major amputation	54	Self-reported physical activity, n(%)	Never	4123 (13.9%)	12.8	3290 (13.7%)
Major amputation	54	Self-reported physical activity, n(%)	<1 times/week	3975 (13.4%)	12.8	3139 (13.1%)
Major amputation	54	Self-reported physical activity, n(%)	regularly - 1-2 times/week	6036 (20.3%)	12.8	4871 (20.3%)

Major amputation	54	Self-reported physical activity, n(%)	regularly - 3-5 times/week	7222 (24.3%)	12.8	5911 (24.6%)	
Major amputation	54	Self-reported physical activity, n(%)	Daily	8343 (28.1%)	12.8	6801 (28.3%)	
Major amputation	54	Sex, n(%)	Males	21222 (62.3%)	0.1	15102 (62.9%)	n=34075 vs n=24012
Major amputation	54	Sex, n(%)	Females	12826 (37.7%)	0.1	8910 (37.1%)	
Major amputation	54	Smoking status, n(%)	No	24314 (77.3%)	7.7	18766 (78.2%)	
Major amputation	54	Smoking status, n(%)	Yes	7135 (22.7%)	7.7	5246 (21.8%)	
Major amputation	54	Stroke, n(%)	No	30015 (97%)	9.2	23309 (97.1%)	
Major amputation	54	Stroke, n(%)	Yes	934 (3%)	9.2	703 (2.9%)	
Major amputation	54	Systolic blood pressure, mean(SD)		132.4 (14.9)	3.3	132.1 (14.7)	
Major amputation	54	Time since last healthcare visit, mean(SD)		0.7 (1.2)	0	0.6 (0.9)	
Major amputation	54	Total cholesterol, mean(SD)		4.9 (1.2)	10.1	4.8 (1.2)	
Major amputation	55	Age at diagnosis of type 2 diabetes, mean(SD)		51 (2.8)	0	50.5 (2.8)	
Major amputation	55	BMI, mean(SD)		31.7 (5.7)	8.3	31.6 (5.6)	
Major amputation	55	HbA1c, mean(SD)		52.1 (14.4)	2.4	52.2 (14)	
Major amputation	55	HDL-cholesterol, mean(SD)		1.2 (0.3)	14.1	1.2 (0.3)	

Major amputation	55	Ischemic heart disease, n(%)	No	29913 (91.1%)	8.9	23201 (91.4%)	
Major amputation	55	Ischemic heart disease, n(%)	Yes	2915 (8.9%)	8.9	2170 (8.6%)	
Major amputation	55	Neuropathy, n(%)	No	31669 (87.9%)	0	21648 (85.3%)	
Major amputation	55	Neuropathy, n(%)	Yes	4374 (12.1%)	0	3723 (14.7%)	
Major amputation	55	Self-reported physical activity, n(%)	Never	4308 (13.7%)	12.8	3447 (13.6%)	
Major amputation	55	Self-reported physical activity, n(%)	<1 times/week	4202 (13.4%)	12.8	3327 (13.1%)	
Major amputation	55	Self-reported physical activity, n(%)	regularly - 1-2 times/week	6485 (20.6%)	12.8	5185 (20.4%)	
Major amputation	55	Self-reported physical activity, n(%)	regularly - 3-5 times/week	7609 (24.2%)	12.8	6178 (24.4%)	
Major amputation	55	Self-reported physical activity, n(%)	Daily	8841 (28.1%)	12.8	7234 (28.5%)	
Major amputation	55	Sex, n(%)	Males	22414 (62.2%)	0.1	15875 (62.6%)	n=36043 vs n=25371
Major amputation	55	Sex, n(%)	Females	13600 (37.8%)	0.1	9496 (37.4%)	
Major amputation	55	Smoking status, n(%)	No	25751 (77.3%)	7.5	19808 (78.1%)	
Major amputation	55	Smoking status, n(%)	Yes	7582 (22.7%)	7.5	5563 (21.9%)	
Major amputation	55	Stroke, n(%)	No	31628 (96.7%)	9.3	24551 (96.8%)	
Major amputation	55	Stroke, n(%)	Yes	1069 (3.3%)	9.3	820 (3.2%)	

Major amputation	55	Systolic blood pressure, mean(SD)		132.8 (14.9)	3.4	132.4 (14.7)
Major amputation	55	Time since last healthcare visit, mean(SD)		0.7 (1.2)	0	0.6 (0.9)
Major amputation	55	Total cholesterol, mean(SD)		4.9 (1.2)	10.1	4.8 (1.2)
Major amputation	56	Age at diagnosis of type 2 diabetes, mean(SD)		52 (2.8)	0	51.5 (2.8)
Major amputation	56	BMI, mean(SD)		31.5 (5.7)	8.2	31.5 (5.6)
Major amputation	56	HbA1c, mean(SD)		51.8 (13.9)	2.2	51.9 (13.4)
Major amputation	56	HDL-cholesterol, mean(SD)		1.2 (0.4)	14	1.2 (0.3)
Major amputation	56	Ischemic heart disease, n(%)	No	31073 (90.3%)	9	24213 (90.5%)
Major amputation	56	Ischemic heart disease, n(%)	Yes	3354 (9.7%)	9	2527 (9.5%)
Major amputation	56	Neuropathy, n(%)	No	33154 (87.6%)	0	22786 (85.2%)
Major amputation	56	Neuropathy, n(%)	Yes	4673 (12.4%)	0	3954 (14.8%)
Major amputation	56	Self-reported physical activity, n(%)	Never	4555 (13.8%)	12.6	3660 (13.7%)
Major amputation	56	Self-reported physical activity, n(%)	<1 times/week	4343 (13.1%)	12.6	3407 (12.7%)
Major amputation	56	Self-reported physical activity, n(%)	regularly - 1-2 times/week	6904 (20.9%)	12.6	5574 (20.8%)
Major amputation	56	Self-reported physical activity, n(%)	regularly - 3-5 times/week	8042 (24.3%)	12.6	6584 (24.6%)

Major amputation	56	Self-reported physical activity, n(%)	Daily	9235 (27.9%)	12.6	7515 (28.1%)	
Major amputation	56	Sex, n(%)	Males	23492 (62.2%)	0.1	16725 (62.5%)	n=37827 vs n=26740
Major amputation	56	Sex, n(%)	Females	14297 (37.8%)	0.1	10015 (37.5%)	
Major amputation	56	Smoking status, n(%)	No	26998 (77.2%)	7.6	20847 (78%)	
Major amputation	56	Smoking status, n(%)	Yes	7970 (22.8%)	7.6	5893 (22%)	
Major amputation	56	Stroke, n(%)	No	33058 (96.5%)	9.4	25813 (96.5%)	
Major amputation	56	Stroke, n(%)	Yes	1213 (3.5%)	9.4	927 (3.5%)	
Major amputation	56	Systolic blood pressure, mean(SD)		132.9 (14.9)	3.2	132.6 (14.6)	
Major amputation	56	Time since last healthcare visit, mean(SD)		0.7 (1.2)	0	0.6 (0.9)	
Major amputation	56	Total cholesterol, mean(SD)		4.9 (1.2)	9.9	4.8 (1.2)	
Major amputation	57	Age at diagnosis of type 2 diabetes, mean(SD)		53 (2.8)	0	52.4 (2.8)	
Major amputation	57	BMI, mean(SD)		31.4 (5.6)	8.2	31.4 (5.5)	
Major amputation	57	HbA1c, mean(SD)		51.7 (13.8)	2.2	51.8 (13.3)	
Major amputation	57	HDL-cholesterol, mean(SD)		1.2 (0.4)	13.7	1.2 (0.4)	
Major amputation	57	Ischemic heart disease, n(%)	No	32352 (89.4%)	8.9	25312 (89.7%)	

Major amputation	57	Ischemic heart disease, n(%)	Yes	3828 (10.6%)	8.9	2893 (10.3%)	
Major amputation	57	Neuropathy, n(%)	No	34602 (87.1%)	0	23894 (84.7%)	
Major amputation	57	Neuropathy, n(%)	Yes	5102 (12.9%)	0	4311 (15.3%)	
Major amputation	57	Self-reported physical activity, n(%)	Never	4666 (13.4%)	12.5	3761 (13.3%)	
Major amputation	57	Self-reported physical activity, n(%)	<1 times/week	4556 (13.1%)	12.5	3633 (12.9%)	
Major amputation	57	Self-reported physical activity, n(%)	regularly - 1-2 times/week	7220 (20.8%)	12.5	5905 (20.9%)	
Major amputation	57	Self-reported physical activity, n(%)	regularly - 3-5 times/week	8383 (24.1%)	12.5	6832 (24.2%)	
Major amputation	57	Self-reported physical activity, n(%)	Daily	9934 (28.6%)	12.5	8074 (28.6%)	
Major amputation	57	Sex, n(%)	Males	24670 (62.2%)	0.1	17581 (62.3%)	n=39704 vs n=28205
Major amputation	57	Sex, n(%)	Females	14994 (37.8%)	0.1	10624 (37.7%)	
Major amputation	57	Smoking status, n(%)	No	28656 (77.9%)	7.3	22153 (78.5%)	
Major amputation	57	Smoking status, n(%)	Yes	8147 (22.1%)	7.3	6052 (21.5%)	
Major amputation	57	Stroke, n(%)	No	34670 (96.3%)	9.3	27189 (96.4%)	
Major amputation	57	Stroke, n(%)	Yes	1328 (3.7%)	9.3	1016 (3.6%)	
Major amputation	57	Systolic blood pressure, mean(SD)		133.3 (15.1)	3.2	132.9 (14.8)	

Major amputation	57	Time since last healthcare visit, mean(SD)		0.7 (1.1)	0	0.6 (0.9)
Major amputation	57	Total cholesterol, mean(SD)		4.9 (1.2)	9.7	4.8 (1.2)
Major amputation	58	Age at diagnosis of type 2 diabetes, mean(SD)		53.9 (2.8)	0	53.4 (2.8)
Major amputation	58	BMI, mean(SD)		31.3 (5.5)	7.9	31.2 (5.5)
Major amputation	58	HbA1c, mean(SD)		51.5 (13.6)	2.1	51.6 (13)
Major amputation	58	HDL-cholesterol, mean(SD)		1.2 (0.4)	13.5	1.2 (0.4)
Major amputation	58	Ischemic heart disease, n(%)	No	33906 (88.9%)	8.5	26644 (89.2%)
Major amputation	58	Ischemic heart disease, n(%)	Yes	4251 (11.1%)	8.5	3211 (10.8%)
Major amputation	58	Neuropathy, n(%)	No	36282 (87%)	0	25199 (84.4%)
Major amputation	58	Neuropathy, n(%)	Yes	5439 (13%)	0	4656 (15.6%)
Major amputation	58	Self-reported physical activity, n(%)	Never	4835 (13.2%)	12.4	3917 (13.1%)
Major amputation	58	Self-reported physical activity, n(%)	<1 times/week	4668 (12.8%)	12.4	3746 (12.5%)
Major amputation	58	Self-reported physical activity, n(%)	regularly - 1-2 times/week	7676 (21%)	12.4	6260 (21%)
Major amputation	58	Self-reported physical activity, n(%)	regularly - 3-5 times/week	8794 (24.1%)	12.4	7235 (24.2%)
Major amputation	58	Self-reported physical activity, n(%)	Daily	10581 (28.9%)	12.4	8697 (29.1%)

Major amputation	58	Sex, n(%)	Males	25836 (62%)	0.1	18614 (62.3%)	n=41721 vs n=29855
Major amputation	58	Sex, n(%)	Females	15846 (38%)	0.1	11241 (37.7%)	
Major amputation	58	Smoking status, n(%)	No	30417 (78.5%)	7.1	23638 (79.2%)	
Major amputation	58	Smoking status, n(%)	Yes	8346 (21.5%)	7.1	6217 (20.8%)	
Major amputation	58	Stroke, n(%)	No	36477 (96.1%)	9	28715 (96.2%)	
Major amputation	58	Stroke, n(%)	Yes	1500 (3.9%)	9	1140 (3.8%)	
Major amputation	58	Systolic blood pressure, mean(SD)		133.5 (15)	3.1	133.2 (14.7)	
Major amputation	58	Time since last healthcare visit, mean(SD)		0.7 (1.1)	0	0.6 (0.9)	
Major amputation	58	Total cholesterol, mean(SD)		4.9 (1.2)	9.5	4.8 (1.2)	
Major amputation	59	Age at diagnosis of type 2 diabetes, mean(SD)		54.9 (2.9)	0	54.4 (2.8)	
Major amputation	59	BMI, mean(SD)		31.1 (5.4)	8	31.1 (5.4)	
Major amputation	59	HbA1c, mean(SD)		51.3 (13.1)	2.1	51.4 (12.6)	
Major amputation	59	HDL-cholesterol, mean(SD)		1.2 (0.4)	13.2	1.2 (0.4)	
Major amputation	59	Ischemic heart disease, n(%)	No	35581 (88.1%)	8.5	28018 (88.5%)	
Major amputation	59	Ischemic heart disease, n(%)	Yes	4826 (11.9%)	8.5	3642 (11.5%)	

Major amputation	59	Neuropathy, n(%)	No	38157 (86.4%)	0	26546 (83.8%)	
Major amputation	59	Neuropathy, n(%)	Yes	5999 (13.6%)	0	5114 (16.2%)	
Major amputation	59	Self-reported physical activity, n(%)	Never	5062 (13%)	12.1	4107 (13%)	
Major amputation	59	Self-reported physical activity, n(%)	<1 times/week	4987 (12.9%)	12.1	4002 (12.6%)	
Major amputation	59	Self-reported physical activity, n(%)	regularly - 1-2 times/week	8128 (20.9%)	12.1	6593 (20.8%)	
Major amputation	59	Self-reported physical activity, n(%)	regularly - 3-5 times/week	9322 (24%)	12.1	7631 (24.1%)	
Major amputation	59	Self-reported physical activity, n(%)	Daily	11304 (29.1%)	12.1	9327 (29.5%)	
Major amputation	59	Sex, n(%)	Males	27350 (62%)	0.1	19725 (62.3%)	n=44156 vs n=31660
Major amputation	59	Sex, n(%)	Females	16763 (38%)	0.1	11935 (37.7%)	
Major amputation	59	Smoking status, n(%)	No	32373 (78.9%)	7	25241 (79.7%)	
Major amputation	59	Smoking status, n(%)	Yes	8677 (21.1%)	7	6419 (20.3%)	
Major amputation	59	Stroke, n(%)	No	38540 (95.8%)	8.9	30396 (96%)	
Major amputation	59	Stroke, n(%)	Yes	1675 (4.2%)	8.9	1264 (4%)	
Major amputation	59	Systolic blood pressure, mean(SD)		133.9 (15.2)	3	133.6 (14.9)	
Major amputation	59	Time since last healthcare visit, mean(SD)		0.7 (1.1)	0	0.6 (0.9)	

Major amputation	59	Total cholesterol, mean(SD)		4.9 (1.2)	9.3	4.8 (1.2)	
Major amputation	60	Age at diagnosis of type 2 diabetes, mean(SD)		55.9 (2.9)	0	55.4 (2.9)	
Major amputation	60	BMI, mean(SD)		31 (5.4)	7.9	31 (5.4)	
Major amputation	60	HbA1c, mean(SD)		51.1 (13)	2.2	51.2 (12.5)	
Major amputation	60	HDL-cholesterol, mean(SD)		1.2 (0.4)	13.3	1.2 (0.4)	
Major amputation	60	Ischemic heart disease, n(%)	No	37362 (87.4%)	8.6	29345 (87.8%)	
Major amputation	60	Ischemic heart disease, n(%)	Yes	5372 (12.6%)	8.6	4062 (12.2%)	
Major amputation	60	Neuropathy, n(%)	No	40150 (85.9%)	0	27799 (83.2%)	
Major amputation	60	Neuropathy, n(%)	Yes	6616 (14.1%)	0	5608 (16.8%)	
Major amputation	60	Self-reported physical activity, n(%)	Never	5383 (13.1%)	12.4	4338 (13%)	
Major amputation	60	Self-reported physical activity, n(%)	<1 times/week	5170 (12.6%)	12.4	4122 (12.3%)	
Major amputation	60	Self-reported physical activity, n(%)	regularly - 1-2 times/week	8543 (20.8%)	12.4	6965 (20.8%)	
Major amputation	60	Self-reported physical activity, n(%)	regularly - 3-5 times/week	9999 (24.4%)	12.4	8153 (24.4%)	
Major amputation	60	Self-reported physical activity, n(%)	Daily	11883 (29%)	12.4	9829 (29.4%)	
Major amputation	60	Sex, n(%)	Males	28805 (61.7%)	0.1	20723 (62%)	n=46766 vs n=33407

Major amputation	60	Sex, n(%)	Females	17914 (38.3%)	0.1	12684 (38%)
Major amputation	60	Smoking status, n(%)	No	34458 (79.3%)	7	26756 (80.1%)
Major amputation	60	Smoking status, n(%)	Yes	9014 (20.7%)	7	6651 (19.9%)
Major amputation	60	Stroke, n(%)	No	40650 (95.6%)	9	32001 (95.8%)
Major amputation	60	Stroke, n(%)	Yes	1889 (4.4%)	9	1406 (4.2%)
Major amputation	60	Systolic blood pressure, mean(SD)		134.3 (15.1)	3	133.9 (14.7)
Major amputation	60	Time since last healthcare visit, mean(SD)		0.7 (1.1)	0	0.6 (0.9)
Major amputation	60	Total cholesterol, mean(SD)		4.8 (1.2)	9.3	4.8 (1.1)
Major amputation	61	Age at diagnosis of type 2 diabetes, mean(SD)		56.9 (2.9)	0	56.4 (2.9)
Major amputation	61	BMI, mean(SD)		30.9 (5.4)	7.9	30.8 (5.3)
Major amputation	61	HbA1c, mean(SD)		50.8 (12.7)	2.1	51 (12.3)
Major amputation	61	HDL-cholesterol, mean(SD)		1.2 (0.4)	13.1	1.2 (0.4)
Major amputation	61	Ischemic heart disease, n(%)	No	39619 (86.8%)	8.7	31259 (87.3%)
Major amputation	61	Ischemic heart disease, n(%)	Yes	6037 (13.2%)	8.7	4549 (12.7%)
Major amputation	61	Neuropathy, n(%)	No	42833 (85.7%)	0	29707 (83%)

Major amputation	61	Neuropathy, n(%)	Yes	7159 (14.3%)	0	6101 (17%)	
Major amputation	61	Self-reported physical activity, n(%)	Never	5687 (13%)	12.2	4534 (12.7%)	
Major amputation	61	Self-reported physical activity, n(%)	<1 times/week	5557 (12.7%)	12.2	4400 (12.3%)	
Major amputation	61	Self-reported physical activity, n(%)	regularly - 1-2 times/week	9151 (20.8%)	12.2	7496 (20.9%)	
Major amputation	61	Self-reported physical activity, n(%)	regularly - 3-5 times/week	10760 (24.5%)	12.2	8783 (24.5%)	
Major amputation	61	Self-reported physical activity, n(%)	Daily	12744 (29%)	12.2	10595 (29.6%)	
Major amputation	61	Sex, n(%)	Males	30693 (61.5%)	0.1	22175 (61.9%)	n=49992 vs n=35808
Major amputation	61	Sex, n(%)	Females	19254 (38.5%)	0.1	13633 (38.1%)	
Major amputation	61	Smoking status, n(%)	No	37106 (79.9%)	7	28865 (80.6%)	
Major amputation	61	Smoking status, n(%)	Yes	9362 (20.1%)	7	6943 (19.4%)	
Major amputation	61	Stroke, n(%)	No	43353 (95.4%)	9.1	34262 (95.7%)	
Major amputation	61	Stroke, n(%)	Yes	2103 (4.6%)	9.1	1546 (4.3%)	
Major amputation	61	Systolic blood pressure, mean(SD)		134.4 (15)	3	134.1 (14.8)	
Major amputation	61	Time since last healthcare visit, mean(SD)		0.7 (1.1)	0	0.6 (0.9)	
Major amputation	61	Total cholesterol, mean(SD)		4.8 (1.2)	9.1	4.8 (1.1)	

Major amputation	62	Age at diagnosis of type 2 diabetes, mean(SD)		57.9 (2.9)	0	57.3 (2.9)	
Major amputation	62	BMI, mean(SD)		30.7 (5.3)	8.1	30.7 (5.3)	
Major amputation	62	HbA1c, mean(SD)		50.5 (12.4)	2.1	50.7 (12.1)	
Major amputation	62	HDL-cholesterol, mean(SD)		1.2 (0.4)	13	1.2 (0.4)	
Major amputation	62	Ischemic heart disease, n(%)	No	41152 (86%)	8.7	32544 (86.5%)	
Major amputation	62	Ischemic heart disease, n(%)	Yes	6717 (14%)	8.7	5097 (13.5%)	
Major amputation	62	Neuropathy, n(%)	No	44559 (85%)	0	30898 (82.1%)	
Major amputation	62	Neuropathy, n(%)	Yes	7867 (15%)	0	6743 (17.9%)	
Major amputation	62	Self-reported physical activity, n(%)	Never	5986 (13%)	12.1	4829 (12.8%)	
Major amputation	62	Self-reported physical activity, n(%)	<1 times/week	5929 (12.9%)	12.1	4702 (12.5%)	
Major amputation	62	Self-reported physical activity, n(%)	regularly - 1-2 times/week	9359 (20.3%)	12.1	7627 (20.3%)	
Major amputation	62	Self-reported physical activity, n(%)	regularly - 3-5 times/week	11277 (24.5%)	12.1	9268 (24.6%)	
Major amputation	62	Self-reported physical activity, n(%)	Daily	13544 (29.4%)	12.1	11215 (29.8%)	
Major amputation	62	Sex, n(%)	Males	32016 (61.1%)	0.1	23189 (61.6%)	n=52426 vs n=37641
Major amputation	62	Sex, n(%)	Females	20366 (38.9%)	0.1	14452 (38.4%)	

Major amputation	62	Smoking status, n(%)	No	39059 (80.2%)	7.1	30531 (81.1%)
Major amputation	62	Smoking status, n(%)	Yes	9658 (19.8%)	7.1	7110 (18.9%)
Major amputation	62	Stroke, n(%)	No	45315 (95.1%)	9.1	35900 (95.4%)
Major amputation	62	Stroke, n(%)	Yes	2333 (4.9%)	9.1	1741 (4.6%)
Major amputation	62	Systolic blood pressure, mean(SD)		134.8 (15.1)	2.8	134.4 (14.9)
Major amputation	62	Time since last healthcare visit, mean(SD)		0.7 (1.1)	0	0.6 (0.8)
Major amputation	62	Total cholesterol, mean(SD)		4.8 (1.2)	9.1	4.8 (1.1)
Major amputation	63	Age at diagnosis of type 2 diabetes, mean(SD)		58.9 (2.9)	0	58.3 (2.9)
Major amputation	63	BMI, mean(SD)		30.6 (5.3)	8.2	30.6 (5.2)
Major amputation	63	HbA1c, mean(SD)		50.3 (12.2)	2.1	50.5 (11.8)
Major amputation	63	HDL-cholesterol, mean(SD)		1.2 (0.4)	13.2	1.2 (0.4)
Major amputation	63	Ischemic heart disease, n(%)	No	42651 (85.2%)	8.8	33639 (85.6%)
Major amputation	63	Ischemic heart disease, n(%)	Yes	7401 (14.8%)	8.8	5647 (14.4%)
Major amputation	63	Neuropathy, n(%)	No	46354 (84.5%)	0	31996 (81.4%)
Major amputation	63	Neuropathy, n(%)	Yes	8523 (15.5%)	0	7290 (18.6%)

Major amputation	63	Self-reported physical activity, n(%)	Never	6205 (12.9%)	12.1	4981 (12.7%)	
Major amputation	63	Self-reported physical activity, n(%)	<1 times/week	6072 (12.6%)	12.1	4833 (12.3%)	
Major amputation	63	Self-reported physical activity, n(%)	regularly - 1-2 times/week	9888 (20.5%)	12.1	7996 (20.4%)	
Major amputation	63	Self-reported physical activity, n(%)	regularly - 3-5 times/week	11698 (24.3%)	12.1	9574 (24.4%)	
Major amputation	63	Self-reported physical activity, n(%)	Daily	14374 (29.8%)	12.1	11902 (30.3%)	
Major amputation	63	Sex, n(%)	Males	33370 (60.9%)	0.1	24114 (61.4%)	n=54877 vs n=39286
Major amputation	63	Sex, n(%)	Females	21461 (39.1%)	0.1	15172 (38.6%)	
Major amputation	63	Smoking status, n(%)	No	41239 (80.8%)	7	32088 (81.7%)	
Major amputation	63	Smoking status, n(%)	Yes	9785 (19.2%)	7	7198 (18.3%)	
Major amputation	63	Stroke, n(%)	No	47214 (94.8%)	9.2	37354 (95.1%)	
Major amputation	63	Stroke, n(%)	Yes	2593 (5.2%)	9.2	1932 (4.9%)	
Major amputation	63	Systolic blood pressure, mean(SD)		135.1 (15.2)	2.9	134.7 (14.9)	
Major amputation	63	Time since last healthcare visit, mean(SD)		0.7 (1.1)	0	0.6 (0.9)	
Major amputation	63	Total cholesterol, mean(SD)		4.8 (1.1)	9	4.8 (1.1)	
Major amputation	64	Age at diagnosis of type 2 diabetes, mean(SD)		59.8 (2.9)	0	59.3 (2.9)	

Major amputation	64	BMI, mean(SD)		30.5 (5.3)	8.1	30.5 (5.3)	
Major amputation	64	HbA1c, mean(SD)		50.1 (12)	2.1	50.3 (11.6)	
Major amputation	64	HDL-cholesterol, mean(SD)		1.3 (0.4)	13.1	1.3 (0.4)	
Major amputation	64	Ischemic heart disease, n(%)	No	44083 (84.5%)	8.6	34830 (84.9%)	
Major amputation	64	Ischemic heart disease, n(%)	Yes	8108 (15.5%)	8.6	6191 (15.1%)	
Major amputation	64	Neuropathy, n(%)	No	48026 (84.1%)	0	33177 (80.9%)	
Major amputation	64	Neuropathy, n(%)	Yes	9101 (15.9%)	0	7844 (19.1%)	
Major amputation	64	Self-reported physical activity, n(%)	Never	6241 (12.4%)	12.2	5021 (12.2%)	
Major amputation	64	Self-reported physical activity, n(%)	<1 times/week	6051 (12.1%)	12.2	4805 (11.7%)	
Major amputation	64	Self-reported physical activity, n(%)	regularly - 1-2 times/week	10120 (20.2%)	12.2	8242 (20.1%)	
Major amputation	64	Self-reported physical activity, n(%)	regularly - 3-5 times/week	12313 (24.5%)	12.2	10150 (24.7%)	
Major amputation	64	Self-reported physical activity, n(%)	Daily	15460 (30.8%)	12.2	12803 (31.2%)	
Major amputation	64	Sex, n(%)	Males	34689 (60.8%)	0.1	25047 (61.1%)	n=57127 vs n=41021
Major amputation	64	Sex, n(%)	Females	22397 (39.2%)	0.1	15974 (38.9%)	
Major amputation	64	Smoking status, n(%)	No	43207 (81.4%)	7.1	33746 (82.3%)	

Major amputation	64	Smoking status, n(%)	Yes	9875 (18.6%)	7.1	7275 (17.7%)
Major amputation	64	Stroke, n(%)	No	49041 (94.4%)	9.1	38844 (94.7%)
Major amputation	64	Stroke, n(%)	Yes	2896 (5.6%)	9.1	2177 (5.3%)
Major amputation	64	Systolic blood pressure, mean(SD)		135.2 (15.2)	2.8	134.8 (14.9)
Major amputation	64	Time since last healthcare visit, mean(SD)		0.7 (1.1)	0	0.6 (0.8)
Major amputation	64	Total cholesterol, mean(SD)		4.8 (1.1)	9	4.8 (1.1)
Major amputation	65	Age at diagnosis of type 2 diabetes, mean(SD)		60.8 (2.9)	0	60.2 (2.9)
Major amputation	65	BMI, mean(SD)		30.4 (5.3)	8.1	30.4 (5.3)
Major amputation	65	HbA1c, mean(SD)		49.7 (11.7)	2	49.9 (11.3)
Major amputation	65	HDL-cholesterol, mean(SD)		1.3 (0.4)	13	1.3 (0.4)
Major amputation	65	Ischemic heart disease, n(%)	No	45582 (83.7%)	8.6	36081 (84.2%)
Major amputation	65	Ischemic heart disease, n(%)	Yes	8850 (16.3%)	8.6	6767 (15.8%)
Major amputation	65	Neuropathy, n(%)	No	49621 (83.3%)	0	34257 (80%)
Major amputation	65	Neuropathy, n(%)	Yes	9963 (16.7%)	0	8591 (20%)
Major amputation	65	Self-reported physical activity, n(%)	Never	6441 (12.3%)	12	5190 (12.1%)

Major amputation	65	Self-reported physical activity, n(%)	<1 times/week	6149 (11.7%)	12	4890 (11.4%)	
Major amputation	65	Self-reported physical activity, n(%)	regularly - 1-2 times/week	10479 (20%)	12	8507 (19.9%)	
Major amputation	65	Self-reported physical activity, n(%)	regularly - 3-5 times/week	12647 (24.1%)	12	10440 (24.4%)	
Major amputation	65	Self-reported physical activity, n(%)	Daily	16726 (31.9%)	12	13821 (32.3%)	
Major amputation	65	Sex, n(%)	Males	36020 (60.5%)	0.1	26035 (60.8%)	n=59584 vs n=42848
Major amputation	65	Sex, n(%)	Females	23519 (39.5%)	0.1	16813 (39.2%)	
Major amputation	65	Smoking status, n(%)	No	45485 (82.1%)	7	35443 (82.7%)	
Major amputation	65	Smoking status, n(%)	Yes	9942 (17.9%)	7	7405 (17.3%)	
Major amputation	65	Stroke, n(%)	No	50938 (94.1%)	9.1	40438 (94.4%)	
Major amputation	65	Stroke, n(%)	Yes	3202 (5.9%)	9.1	2410 (5.6%)	
Major amputation	65	Systolic blood pressure, mean(SD)		135.3 (15.1)	2.8	134.9 (14.8)	
Major amputation	65	Time since last healthcare visit, mean(SD)		0.7 (1.1)	0	0.6 (0.9)	
Major amputation	65	Total cholesterol, mean(SD)		4.8 (1.1)	8.8	4.7 (1.1)	
Major amputation	66	Age at diagnosis of type 2 diabetes, mean(SD)		61.8 (2.9)	0	61.2 (2.9)	
Major amputation	66	BMI, mean(SD)		30.3 (5.3)	8.1	30.3 (5.2)	

Major amputation	66	HbA1c, mean(SD)		49.6 (11.7)	1.9	49.7 (11.3)	
Major amputation	66	HDL-cholesterol, mean(SD)		1.3 (0.4)	12.7	1.3 (0.4)	
Major amputation	66	Ischemic heart disease, n(%)	No	47269 (83.1%)	8.5	37504 (83.5%)	
Major amputation	66	Ischemic heart disease, n(%)	Yes	9594 (16.9%)	8.5	7395 (16.5%)	
Major amputation	66	Neuropathy, n(%)	No	51185 (82.3%)	0	35413 (78.9%)	
Major amputation	66	Neuropathy, n(%)	Yes	10976 (17.7%)	0	9486 (21.1%)	
Major amputation	66	Self-reported physical activity, n(%)	Never	6517 (11.9%)	11.8	5216 (11.6%)	
Major amputation	66	Self-reported physical activity, n(%)	<1 times/week	6152 (11.2%)	11.8	4903 (10.9%)	
Major amputation	66	Self-reported physical activity, n(%)	regularly - 1-2 times/week	10568 (19.3%)	11.8	8594 (19.1%)	
Major amputation	66	Self-reported physical activity, n(%)	regularly - 3-5 times/week	13264 (24.2%)	11.8	10987 (24.5%)	
Major amputation	66	Self-reported physical activity, n(%)	Daily	18342 (33.4%)	11.8	15199 (33.9%)	
Major amputation	66	Sex, n(%)	Males	37371 (60.2%)	0.1	27179 (60.5%)	n=62161 vs n=44899
Major amputation	66	Sex, n(%)	Females	24737 (39.8%)	0.1	17720 (39.5%)	
Major amputation	66	Smoking status, n(%)	No	47940 (82.8%)	6.8	37424 (83.4%)	
Major amputation	66	Smoking status, n(%)	Yes	9968 (17.2%)	6.8	7475 (16.6%)	

Major amputation	66	Stroke, n(%)	No	53073 (93.8%)	9	42254 (94.1%)
Major amputation	66	Stroke, n(%)	Yes	3508 (6.2%)	9	2645 (5.9%)
Major amputation	66	Systolic blood pressure, mean(SD)		135.6 (15.3)	2.7	135.2 (15)
Major amputation	66	Time since last healthcare visit, mean(SD)		0.7 (1.1)	0	0.6 (0.9)
Major amputation	66	Total cholesterol, mean(SD)		4.8 (1.1)	8.7	4.7 (1.1)
Major amputation	67	Age at diagnosis of type 2 diabetes, mean(SD)		62.7 (2.9)	0	62.1 (2.9)
Major amputation	67	BMI, mean(SD)		30.2 (5.2)	8.1	30.2 (5.2)
Major amputation	67	HbA1c, mean(SD)		49.3 (11.3)	2	49.5 (11)
Major amputation	67	HDL-cholesterol, mean(SD)		1.3 (0.4)	12.5	1.3 (0.4)
Major amputation	67	Ischemic heart disease, n(%)	No	48376 (82.4%)	8.4	38474 (82.9%)
Major amputation	67	Ischemic heart disease, n(%)	Yes	10362 (17.6%)	8.4	7939 (17.1%)
Major amputation	67	Neuropathy, n(%)	No	52227 (81.4%)	0	36167 (77.9%)
Major amputation	67	Neuropathy, n(%)	Yes	11896 (18.6%)	0	10246 (22.1%)
Major amputation	67	Self-reported physical activity, n(%)	Never	6694 (11.8%)	11.7	5337 (11.5%)
Major amputation	67	Self-reported physical activity, n(%)	<1 times/week	6235 (11%)	11.7	4946 (10.7%)

Major amputation	67	Self-reported physical activity, n(%)	regularly - 1-2 times/week	10514 (18.6%)	11.7	8516 (18.3%)	
Major amputation	67	Self-reported physical activity, n(%)	regularly - 3-5 times/week	13732 (24.2%)	11.7	11415 (24.6%)	
Major amputation	67	Self-reported physical activity, n(%)	Daily	19468 (34.4%)	11.7	16199 (34.9%)	
Major amputation	67	Sex, n(%)	Males	38238 (59.7%)	0.1	27918 (60.2%)	n=64123 vs n=46413
Major amputation	67	Sex, n(%)	Females	25835 (40.3%)	0.1	18495 (39.8%)	
Major amputation	67	Smoking status, n(%)	No	50141 (83.8%)	6.7	39181 (84.4%)	
Major amputation	67	Smoking status, n(%)	Yes	9705 (16.2%)	6.7	7232 (15.6%)	
Major amputation	67	Stroke, n(%)	No	54589 (93.4%)	8.9	43527 (93.8%)	
Major amputation	67	Stroke, n(%)	Yes	3841 (6.6%)	8.9	2886 (6.2%)	
Major amputation	67	Systolic blood pressure, mean(SD)		135.7 (15.2)	2.7	135.4 (14.9)	
Major amputation	67	Time since last healthcare visit, mean(SD)		0.7 (1.1)	0	0.6 (0.9)	
Major amputation	67	Total cholesterol, mean(SD)		4.8 (1.1)	8.5	4.7 (1.1)	
Major amputation	68	Age at diagnosis of type 2 diabetes, mean(SD)		63.6 (2.9)	0	63.1 (2.9)	
Major amputation	68	BMI, mean(SD)		30.1 (5.3)	7.9	30.1 (5.2)	
Major amputation	68	HbA1c, mean(SD)		49.1 (11.1)	1.9	49.2 (10.7)	

Major amputation	68	HDL-cholesterol, mean(SD)		1.3 (0.4)	12.4	1.3 (0.4)	
Major amputation	68	Ischemic heart disease, n(%)	No	49007 (81.6%)	8.3	39208 (82.1%)	
Major amputation	68	Ischemic heart disease, n(%)	Yes	11045 (18.4%)	8.3	8530 (17.9%)	
Major amputation	68	Neuropathy, n(%)	No	52715 (80.5%)	0	36726 (76.9%)	
Major amputation	68	Neuropathy, n(%)	Yes	12744 (19.5%)	0	11012 (23.1%)	
Major amputation	68	Self-reported physical activity, n(%)	Never	6912 (11.9%)	11.4	5554 (11.6%)	
Major amputation	68	Self-reported physical activity, n(%)	<1 times/week	6393 (11%)	11.4	5122 (10.7%)	
Major amputation	68	Self-reported physical activity, n(%)	regularly - 1-2 times/week	10470 (18%)	11.4	8461 (17.7%)	
Major amputation	68	Self-reported physical activity, n(%)	regularly - 3-5 times/week	13978 (24.1%)	11.4	11571 (24.2%)	
Major amputation	68	Self-reported physical activity, n(%)	Daily	20256 (34.9%)	11.4	17030 (35.7%)	
Major amputation	68	Sex, n(%)	Males	38785 (59.3%)	0.1	28501 (59.7%)	n=65459 vs n=47738
Major amputation	68	Sex, n(%)	Females	26632 (40.7%)	0.1	19237 (40.3%)	
Major amputation	68	Smoking status, n(%)	No	51641 (84.5%)	6.6	40583 (85%)	
Major amputation	68	Smoking status, n(%)	Yes	9491 (15.5%)	6.6	7155 (15%)	
Major amputation	68	Stroke, n(%)	No	55598 (93.1%)	8.8	44588 (93.4%)	

Major amputation	68	Stroke, n(%)	Yes	4121 (6.9%)	8.8	3150 (6.6%)
Major amputation	68	Systolic blood pressure, mean(SD)		135.7 (15.2)	2.5	135.3 (14.9)
Major amputation	68	Time since last healthcare visit, mean(SD)		0.7 (1.1)	0	0.6 (0.9)
Major amputation	68	Total cholesterol, mean(SD)		4.7 (1.1)	8.4	4.7 (1.1)
Major amputation	69	Age at diagnosis of type 2 diabetes, mean(SD)		64.5 (2.9)	0	64 (2.9)
Major amputation	69	BMI, mean(SD)		30 (5.2)	7.8	30 (5.2)
Major amputation	69	HbA1c, mean(SD)		49 (10.9)	1.9	49.1 (10.6)
Major amputation	69	HDL-cholesterol, mean(SD)		1.3 (0.4)	12.2	1.3 (0.4)
Major amputation	69	Ischemic heart disease, n(%)	No	49429 (80.8%)	7.8	39690 (81.3%)
Major amputation	69	Ischemic heart disease, n(%)	Yes	11743 (19.2%)	7.8	9108 (18.7%)
Major amputation	69	Neuropathy, n(%)	No	52762 (79.5%)	0	37032 (75.9%)
Major amputation	69	Neuropathy, n(%)	Yes	13611 (20.5%)	0	11766 (24.1%)
Major amputation	69	Self-reported physical activity, n(%)	Never	7017 (11.9%)	11.2	5677 (11.6%)
Major amputation	69	Self-reported physical activity, n(%)	<1 times/week	6495 (11%)	11.2	5187 (10.6%)
Major amputation	69	Self-reported physical activity, n(%)	regularly - 1-2 times/week	10732 (18.2%)	11.2	8816 (18.1%)

Major amputation	69	Self-reported physical activity, n(%)	regularly - 3-5 times/week	14036 (23.8%)	11.2	11712 (24%)	
Major amputation	69	Self-reported physical activity, n(%)	Daily	20659 (35.1%)	11.2	17406 (35.7%)	
Major amputation	69	Sex, n(%)	Males	39024 (58.8%)	0.1	28895 (59.2%)	n=66373 vs n=48798
Major amputation	69	Sex, n(%)	Females	27311 (41.2%)	0.1	19903 (40.8%)	
Major amputation	69	Smoking status, n(%)	No	52944 (85.3%)	6.5	41913 (85.9%)	
Major amputation	69	Smoking status, n(%)	Yes	9119 (14.7%)	6.5	6885 (14.1%)	
Major amputation	69	Stroke, n(%)	No	56502 (92.9%)	8.3	45472 (93.2%)	
Major amputation	69	Stroke, n(%)	Yes	4347 (7.1%)	8.3	3326 (6.8%)	
Major amputation	69	Systolic blood pressure, mean(SD)		135.8 (15.2)	2.5	135.5 (14.9)	
Major amputation	69	Time since last healthcare visit, mean(SD)		0.7 (1.1)	0	0.6 (0.9)	
Major amputation	69	Total cholesterol, mean(SD)		4.7 (1.1)	8.2	4.7 (1.1)	
Major amputation	70	Age at diagnosis of type 2 diabetes, mean(SD)		65.5 (3)	0	64.9 (2.9)	
Major amputation	70	BMI, mean(SD)		29.9 (5.2)	7.7	29.9 (5.1)	
Major amputation	70	HbA1c, mean(SD)		48.9 (10.9)	1.9	48.9 (10.5)	
Major amputation	70	HDL-cholesterol, mean(SD)		1.3 (0.4)	12.3	1.3 (0.4)	

Major amputation	70	Ischemic heart disease, n(%)	No	49154 (80%)	7.7	39419 (80.6%)	
Major amputation	70	Ischemic heart disease, n(%)	Yes	12262 (20%)	7.7	9507 (19.4%)	
Major amputation	70	Neuropathy, n(%)	No	52170 (78.4%)	0	36479 (74.6%)	
Major amputation	70	Neuropathy, n(%)	Yes	14378 (21.6%)	0	12447 (25.4%)	
Major amputation	70	Self-reported physical activity, n(%)	Never	7095 (12%)	11.1	5803 (11.9%)	
Major amputation	70	Self-reported physical activity, n(%)	<1 times/week	6375 (10.8%)	11.1	5097 (10.4%)	
Major amputation	70	Self-reported physical activity, n(%)	regularly - 1-2 times/week	10721 (18.1%)	11.1	8779 (17.9%)	
Major amputation	70	Self-reported physical activity, n(%)	regularly - 3-5 times/week	14164 (23.9%)	11.1	11826 (24.2%)	
Major amputation	70	Self-reported physical activity, n(%)	Daily	20786 (35.1%)	11.1	17421 (35.6%)	
Major amputation	70	Sex, n(%)	Males	38879 (58.4%)	0	28821 (58.9%)	n=66548 vs n=48926
Major amputation	70	Sex, n(%)	Females	27638 (41.6%)	0	20105 (41.1%)	
Major amputation	70	Smoking status, n(%)	No	53536 (86.1%)	6.6	42381 (86.6%)	
Major amputation	70	Smoking status, n(%)	Yes	8653 (13.9%)	6.6	6545 (13.4%)	
Major amputation	70	Stroke, n(%)	No	56533 (92.6%)	8.2	45418 (92.8%)	
Major amputation	70	Stroke, n(%)	Yes	4540 (7.4%)	8.2	3508 (7.2%)	

Major amputation	70	Systolic blood pressure, mean(SD)		135.9 (15.3)	2.4	135.5 (15)
Major amputation	70	Time since last healthcare visit, mean(SD)		0.7 (1.1)	0	0.6 (0.9)
Major amputation	70	Total cholesterol, mean(SD)		4.7 (1.1)	8.3	4.7 (1.1)
Major amputation	71	Age at diagnosis of type 2 diabetes, mean(SD)		66.4 (3)	0	65.8 (3)
Major amputation	71	BMI, mean(SD)		29.8 (5.1)	7.6	29.8 (5.1)
Major amputation	71	HbA1c, mean(SD)		48.8 (10.7)	1.8	48.9 (10.2)
Major amputation	71	HDL-cholesterol, mean(SD)		1.3 (0.4)	12.3	1.3 (0.4)
Major amputation	71	Ischemic heart disease, n(%)	No	48239 (79.2%)	7.5	38730 (79.6%)
Major amputation	71	Ischemic heart disease, n(%)	Yes	12704 (20.8%)	7.5	9938 (20.4%)
Major amputation	71	Neuropathy, n(%)	No	51013 (77.4%)	0	35753 (73.5%)
Major amputation	71	Neuropathy, n(%)	Yes	14856 (22.6%)	0	12915 (26.5%)
Major amputation	71	Self-reported physical activity, n(%)	Never	7096 (12.1%)	10.9	5816 (12%)
Major amputation	71	Self-reported physical activity, n(%)	<1 times/week	6595 (11.2%)	10.9	5278 (10.8%)
Major amputation	71	Self-reported physical activity, n(%)	regularly - 1-2 times/week	10676 (18.2%)	10.9	8771 (18%)
Major amputation	71	Self-reported physical activity, n(%)	regularly - 3-5 times/week	13765 (23.5%)	10.9	11518 (23.7%)

Major amputation	71	Self-reported physical activity, n(%)	Daily	20556 (35%)	10.9	17285 (35.5%)	
Major amputation	71	Sex, n(%)	Males	38200 (58%)	0	28370 (58.3%)	n=65869 vs n=48668
Major amputation	71	Sex, n(%)	Females	27637 (42%)	0	20298 (41.7%)	
Major amputation	71	Smoking status, n(%)	No	53473 (86.9%)	6.5	42526 (87.4%)	
Major amputation	71	Smoking status, n(%)	Yes	8092 (13.1%)	6.5	6142 (12.6%)	
Major amputation	71	Stroke, n(%)	No	55860 (92.2%)	8	45039 (92.5%)	
Major amputation	71	Stroke, n(%)	Yes	4730 (7.8%)	8	3629 (7.5%)	
Major amputation	71	Systolic blood pressure, mean(SD)		136 (15.3)	2.4	135.7 (15)	
Major amputation	71	Time since last healthcare visit, mean(SD)		0.7 (1.2)	0	0.6 (0.9)	
Major amputation	71	Total cholesterol, mean(SD)		4.7 (1.1)	8.3	4.6 (1.1)	
Major amputation	72	Age at diagnosis of type 2 diabetes, mean(SD)		67.3 (3)	0	66.8 (3)	
Major amputation	72	BMI, mean(SD)		29.7 (5.1)	7.6	29.6 (5.1)	
Major amputation	72	HbA1c, mean(SD)		48.6 (10.4)	1.8	48.7 (10)	
Major amputation	72	HDL-cholesterol, mean(SD)		1.3 (0.4)	12.2	1.3 (0.4)	
Major amputation	72	Ischemic heart disease, n(%)	No	46433 (78.4%)	7.3	37395 (78.9%)	

Major amputation	72	Ischemic heart disease, n(%)	Yes	12825 (21.6%)	7.3	9999 (21.1%)	
Major amputation	72	Neuropathy, n(%)	No	48749 (76.2%)	0	34142 (72%)	
Major amputation	72	Neuropathy, n(%)	Yes	15209 (23.8%)	0	13252 (28%)	
Major amputation	72	Self-reported physical activity, n(%)	Never	7074 (12.4%)	10.7	5794 (12.2%)	
Major amputation	72	Self-reported physical activity, n(%)	<1 times/week	6279 (11%)	10.7	5039 (10.6%)	
Major amputation	72	Self-reported physical activity, n(%)	regularly - 1-2 times/week	10495 (18.4%)	10.7	8626 (18.2%)	
Major amputation	72	Self-reported physical activity, n(%)	regularly - 3-5 times/week	13341 (23.4%)	10.7	11138 (23.5%)	
Major amputation	72	Self-reported physical activity, n(%)	Daily	19940 (34.9%)	10.7	16797 (35.4%)	
Major amputation	72	Sex, n(%)	Males	36728 (57.5%)	0	27464 (57.9%)	n=63958 vs n=47394
Major amputation	72	Sex, n(%)	Females	27199 (42.5%)	0	19930 (42.1%)	
Major amputation	72	Smoking status, n(%)	No	52435 (87.5%)	6.3	41642 (87.9%)	
Major amputation	72	Smoking status, n(%)	Yes	7497 (12.5%)	6.3	5752 (12.1%)	
Major amputation	72	Stroke, n(%)	No	53947 (91.6%)	7.9	43579 (92%)	
Major amputation	72	Stroke, n(%)	Yes	4974 (8.4%)	7.9	3815 (8%)	
Major amputation	72	Systolic blood pressure, mean(SD)		136 (15.5)	2.4	135.7 (15.2)	

Major amputation	72	Time since last healthcare visit, mean(SD)		0.7 (1.2)	0	0.6 (0.9)
Major amputation	72	Total cholesterol, mean(SD)		4.7 (1.1)	8.3	4.6 (1.1)
Major amputation	73	Age at diagnosis of type 2 diabetes, mean(SD)		68.3 (3.1)	0	67.7 (3)
Major amputation	73	BMI, mean(SD)		29.5 (5.1)	7.9	29.5 (5.1)
Major amputation	73	HbA1c, mean(SD)		48.6 (10.4)	1.9	48.7 (10)
Major amputation	73	HDL-cholesterol, mean(SD)		1.3 (0.4)	12.3	1.3 (0.4)
Major amputation	73	Ischemic heart disease, n(%)	No	44268 (77.5%)	7.5	35558 (78%)
Major amputation	73	Ischemic heart disease, n(%)	Yes	12863 (22.5%)	7.5	10035 (22%)
Major amputation	73	Neuropathy, n(%)	No	46396 (75.1%)	0	32245 (70.7%)
Major amputation	73	Neuropathy, n(%)	Yes	15348 (24.9%)	0	13348 (29.3%)
Major amputation	73	Self-reported physical activity, n(%)	Never	7082 (12.9%)	10.8	5773 (12.7%)
Major amputation	73	Self-reported physical activity, n(%)	<1 times/week	6175 (11.2%)	10.8	4939 (10.8%)
Major amputation	73	Self-reported physical activity, n(%)	regularly - 1-2 times/week	10130 (18.4%)	10.8	8355 (18.3%)
Major amputation	73	Self-reported physical activity, n(%)	regularly - 3-5 times/week	12762 (23.2%)	10.8	10667 (23.4%)
Major amputation	73	Self-reported physical activity, n(%)	Daily	18913 (34.3%)	10.8	15859 (34.8%)

Major amputation	73	Sex, n(%)	Males	34972 (56.7%)	0.1	26097 (57.2%)	n=61744 vs n=45593
Major amputation	73	Sex, n(%)	Females	26736 (43.3%)	0.1	19496 (42.8%)	
Major amputation	73	Smoking status, n(%)	No	51038 (88.4%)	6.5	40433 (88.7%)	
Major amputation	73	Smoking status, n(%)	Yes	6677 (11.6%)	6.5	5160 (11.3%)	
Major amputation	73	Stroke, n(%)	No	51612 (90.9%)	8	41552 (91.1%)	
Major amputation	73	Stroke, n(%)	Yes	5177 (9.1%)	8	4041 (8.9%)	
Major amputation	73	Systolic blood pressure, mean(SD)		136 (15.5)	2.4	135.7 (15.2)	
Major amputation	73	Time since last healthcare visit, mean(SD)		0.7 (1.2)	0	0.6 (0.9)	
Major amputation	73	Total cholesterol, mean(SD)		4.7 (1.1)	8.5	4.6 (1.1)	
Major amputation	74	Age at diagnosis of type 2 diabetes, mean(SD)		69.2 (3.1)	0	68.7 (3)	
Major amputation	74	BMI, mean(SD)		29.4 (5.1)	7.9	29.4 (5.1)	
Major amputation	74	HbA1c, mean(SD)		48.6 (10.2)	1.8	48.6 (9.8)	
Major amputation	74	HDL-cholesterol, mean(SD)		1.3 (0.4)	12.5	1.3 (0.4)	
Major amputation	74	Ischemic heart disease, n(%)	No	41467 (76.6%)	7.6	33320 (77.2%)	
Major amputation	74	Ischemic heart disease, n(%)	Yes	12670 (23.4%)	7.6	9827 (22.8%)	

Major amputation	74	Neuropathy, n(%)	No	43226 (73.8%)	0	29902 (69.3%)	
Major amputation	74	Neuropathy, n(%)	Yes	15334 (26.2%)	0	13245 (30.7%)	
Major amputation	74	Self-reported physical activity, n(%)	Never	7035 (13.5%)	10.8	5719 (13.3%)	
Major amputation	74	Self-reported physical activity, n(%)	<1 times/week	6045 (11.6%)	10.8	4808 (11.1%)	
Major amputation	74	Self-reported physical activity, n(%)	regularly - 1-2 times/week	9687 (18.6%)	10.8	7947 (18.4%)	
Major amputation	74	Self-reported physical activity, n(%)	regularly - 3-5 times/week	11717 (22.4%)	10.8	9804 (22.7%)	
Major amputation	74	Self-reported physical activity, n(%)	Daily	17726 (34%)	10.8	14869 (34.5%)	
Major amputation	74	Sex, n(%)	Males	32816 (56.1%)	0.1	24348 (56.4%)	n=58560 vs n=43147
Major amputation	74	Sex, n(%)	Females	25707 (43.9%)	0.1	18799 (43.6%)	
Major amputation	74	Smoking status, n(%)	No	48754 (89.1%)	6.6	38578 (89.4%)	
Major amputation	74	Smoking status, n(%)	Yes	5938 (10.9%)	6.6	4569 (10.6%)	
Major amputation	74	Stroke, n(%)	No	48641 (90.4%)	8.1	39128 (90.7%)	
Major amputation	74	Stroke, n(%)	Yes	5178 (9.6%)	8.1	4019 (9.3%)	
Major amputation	74	Systolic blood pressure, mean(SD)		136.1 (15.7)	2.4	135.7 (15.4)	
Major amputation	74	Time since last healthcare visit, mean(SD)		0.7 (1.2)	0	0.6 (0.9)	

Major amputation	74	Total cholesterol, mean(SD)		4.6 (1.1)	8.6	4.6 (1.1)	
Major amputation	75	Age at diagnosis of type 2 diabetes, mean(SD)		70.2 (3.1)	0	69.6 (3)	
Major amputation	75	BMI, mean(SD)		29.3 (5)	8	29.3 (5)	
Major amputation	75	HbA1c, mean(SD)		48.5 (10.1)	1.8	48.6 (9.8)	
Major amputation	75	HDL-cholesterol, mean(SD)		1.3 (0.4)	12.5	1.3 (0.4)	
Major amputation	75	Ischemic heart disease, n(%)	No	38321 (75.5%)	7.5	30644 (76%)	
Major amputation	75	Ischemic heart disease, n(%)	Yes	12416 (24.5%)	7.5	9656 (24%)	
Major amputation	75	Neuropathy, n(%)	No	40034 (73%)	0	27475 (68.2%)	
Major amputation	75	Neuropathy, n(%)	Yes	14822 (27%)	0	12825 (31.8%)	
Major amputation	75	Self-reported physical activity, n(%)	Never	6846 (14%)	11	5577 (13.8%)	
Major amputation	75	Self-reported physical activity, n(%)	<1 times/week	5646 (11.6%)	11	4482 (11.1%)	
Major amputation	75	Self-reported physical activity, n(%)	regularly - 1-2 times/week	9129 (18.7%)	11	7465 (18.5%)	
Major amputation	75	Self-reported physical activity, n(%)	regularly - 3-5 times/week	10875 (22.3%)	11	9129 (22.7%)	
Major amputation	75	Self-reported physical activity, n(%)	Daily	16333 (33.4%)	11	13647 (33.9%)	
Major amputation	75	Sex, n(%)	Males	30235 (55.1%)	0.1	22395 (55.6%)	n=54856 vs n=40300

Major amputation	75	Sex, n(%)	Females	24593 (44.9%)	0.1	17905 (44.4%)
Major amputation	75	Smoking status, n(%)	No	46004 (90%)	6.8	36404 (90.3%)
Major amputation	75	Smoking status, n(%)	Yes	5126 (10%)	6.8	3896 (9.7%)
Major amputation	75	Stroke, n(%)	No	45310 (90%)	8.2	36357 (90.2%)
Major amputation	75	Stroke, n(%)	Yes	5057 (10%)	8.2	3943 (9.8%)
Major amputation	75	Systolic blood pressure, mean(SD)		136.3 (15.7)	2.3	135.9 (15.5)
Major amputation	75	Time since last healthcare visit, mean(SD)		0.7 (1.2)	0	0.6 (0.9)
Major amputation	75	Total cholesterol, mean(SD)		4.6 (1.1)	8.6	4.6 (1.1)
Major amputation	76	Age at diagnosis of type 2 diabetes, mean(SD)		71.3 (3.1)	0	70.6 (3)
Major amputation	76	BMI, mean(SD)		29.2 (5)	8.2	29.2 (5)
Major amputation	76	HbA1c, mean(SD)		48.6 (10.1)	1.9	48.6 (9.8)
Major amputation	76	HDL-cholesterol, mean(SD)		1.3 (0.4)	13.2	1.3 (0.4)
Major amputation	76	Ischemic heart disease, n(%)	No	35101 (74.5%)	7.6	27906 (75%)
Major amputation	76	Ischemic heart disease, n(%)	Yes	12016 (25.5%)	7.6	9299 (25%)
Major amputation	76	Neuropathy, n(%)	No	36890 (72.3%)	0	24973 (67.1%)

Major amputation	76	Neuropathy, n(%)	Yes	14127 (27.7%)	0	12232 (32.9%)	
Major amputation	76	Self-reported physical activity, n(%)	Never	6594 (14.6%)	11.3	5355 (14.4%)	
Major amputation	76	Self-reported physical activity, n(%)	<1 times/week	5267 (11.6%)	11.3	4172 (11.2%)	
Major amputation	76	Self-reported physical activity, n(%)	regularly - 1-2 times/week	8415 (18.6%)	11.3	6835 (18.4%)	
Major amputation	76	Self-reported physical activity, n(%)	regularly - 3-5 times/week	9953 (22%)	11.3	8284 (22.3%)	
Major amputation	76	Self-reported physical activity, n(%)	Daily	15033 (33.2%)	11.3	12559 (33.8%)	
Major amputation	76	Sex, n(%)	Males	27787 (54.5%)	0.1	20465 (55%)	n=51017 vs n=37205
Major amputation	76	Sex, n(%)	Females	23203 (45.5%)	0.1	16740 (45%)	
Major amputation	76	Smoking status, n(%)	No	43053 (90.7%)	7	33878 (91.1%)	
Major amputation	76	Smoking status, n(%)	Yes	4404 (9.3%)	7	3327 (8.9%)	
Major amputation	76	Stroke, n(%)	No	41789 (89.4%)	8.3	33361 (89.7%)	
Major amputation	76	Stroke, n(%)	Yes	4981 (10.6%)	8.3	3844 (10.3%)	
Major amputation	76	Systolic blood pressure, mean(SD)		136.1 (15.8)	2.4	135.8 (15.5)	
Major amputation	76	Time since last healthcare visit, mean(SD)		0.7 (1.2)	0	0.6 (0.9)	
Major amputation	76	Total cholesterol, mean(SD)		4.6 (1.1)	9.1	4.6 (1.1)	

Major amputation	77	Age at diagnosis of type 2 diabetes, mean(SD)		72.3 (3.1)	0	71.6 (3)	
Major amputation	77	BMI, mean(SD)		29 (4.9)	8.3	29 (4.9)	
Major amputation	77	HbA1c, mean(SD)		48.5 (10)	1.9	48.6 (9.6)	
Major amputation	77	HDL-cholesterol, mean(SD)		1.3 (0.4)	13.6	1.3 (0.4)	
Major amputation	77	Ischemic heart disease, n(%)	No	32063 (73.4%)	7.8	25349 (73.9%)	
Major amputation	77	Ischemic heart disease, n(%)	Yes	11616 (26.6%)	7.8	8973 (26.1%)	
Major amputation	77	Neuropathy, n(%)	No	33939 (71.7%)	0	22721 (66.2%)	
Major amputation	77	Neuropathy, n(%)	Yes	13428 (28.3%)	0	11601 (33.8%)	
Major amputation	77	Self-reported physical activity, n(%)	Never	6236 (14.9%)	11.7	5028 (14.6%)	
Major amputation	77	Self-reported physical activity, n(%)	<1 times/week	5036 (12%)	11.7	3983 (11.6%)	
Major amputation	77	Self-reported physical activity, n(%)	regularly - 1-2 times/week	7746 (18.5%)	11.7	6323 (18.4%)	
Major amputation	77	Self-reported physical activity, n(%)	regularly - 3-5 times/week	9098 (21.7%)	11.7	7556 (22%)	
Major amputation	77	Self-reported physical activity, n(%)	Daily	13727 (32.8%)	11.7	11432 (33.3%)	
Major amputation	77	Sex, n(%)	Males	25415 (53.7%)	0.1	18724 (54.6%)	n=47367 vs n=34322
Major amputation	77	Sex, n(%)	Females	21925 (46.3%)	0.1	15598 (45.4%)	

Major amputation	77	Smoking status, n(%)	No	40261 (91.5%)	7.1	31521 (91.8%)
Major amputation	77	Smoking status, n(%)	Yes	3748 (8.5%)	7.1	2801 (8.2%)
Major amputation	77	Stroke, n(%)	No	38580 (88.9%)	8.4	30644 (89.3%)
Major amputation	77	Stroke, n(%)	Yes	4822 (11.1%)	8.4	3678 (10.7%)
Major amputation	77	Systolic blood pressure, mean(SD)		136.2 (15.9)	2.5	135.9 (15.6)
Major amputation	77	Time since last healthcare visit, mean(SD)		0.7 (1.2)	0	0.6 (0.9)
Major amputation	77	Total cholesterol, mean(SD)		4.6 (1.1)	9.5	4.6 (1.1)
Major amputation	78	Age at diagnosis of type 2 diabetes, mean(SD)		73.3 (3)	0	72.6 (3)
Major amputation	78	BMI, mean(SD)		28.9 (4.9)	8.6	28.9 (4.9)
Major amputation	78	HbA1c, mean(SD)		48.6 (10)	2	48.7 (9.7)
Major amputation	78	HDL-cholesterol, mean(SD)		1.3 (0.4)	14.1	1.3 (0.4)
Major amputation	78	Ischemic heart disease, n(%)	No	29396 (72.4%)	7.9	23057 (72.8%)
Major amputation	78	Ischemic heart disease, n(%)	Yes	11217 (27.6%)	7.9	8635 (27.2%)
Major amputation	78	Neuropathy, n(%)	No	31256 (70.9%)	0	20707 (65.3%)
Major amputation	78	Neuropathy, n(%)	Yes	12856 (29.1%)	0	10985 (34.7%)

Major amputation	78	Self-reported physical activity, n(%)	Never	6045 (15.6%)	12	4816 (15.2%)	
Major amputation	78	Self-reported physical activity, n(%)	<1 times/week	4809 (12.4%)	12	3774 (11.9%)	
Major amputation	78	Self-reported physical activity, n(%)	regularly - 1-2 times/week	7128 (18.4%)	12	5825 (18.4%)	
Major amputation	78	Self-reported physical activity, n(%)	regularly - 3-5 times/week	8349 (21.5%)	12	6890 (21.7%)	
Major amputation	78	Self-reported physical activity, n(%)	Daily	12505 (32.2%)	12	10387 (32.8%)	
Major amputation	78	Sex, n(%)	Males	23173 (52.6%)	0.1	16915 (53.4%)	n=44112 vs n=31692
Major amputation	78	Sex, n(%)	Females	20914 (47.4%)	0.1	14777 (46.6%)	
Major amputation	78	Smoking status, n(%)	No	37642 (92%)	7.2	29262 (92.3%)	
Major amputation	78	Smoking status, n(%)	Yes	3272 (8%)	7.2	2430 (7.7%)	
Major amputation	78	Stroke, n(%)	No	35647 (88.5%)	8.7	28162 (88.9%)	
Major amputation	78	Stroke, n(%)	Yes	4647 (11.5%)	8.7	3530 (11.1%)	
Major amputation	78	Systolic blood pressure, mean(SD)		136.4 (16)	2.5	136 (15.7)	
Major amputation	78	Time since last healthcare visit, mean(SD)		0.7 (1.2)	0	0.6 (0.9)	
Major amputation	78	Total cholesterol, mean(SD)		4.6 (1.1)	9.8	4.6 (1.1)	
Major amputation	79	Age at diagnosis of type 2 diabetes, mean(SD)		74.2 (3.1)	0	73.6 (3)	

Major amputation	79	BMI, mean(SD)		28.8 (4.9)	9.1	28.7 (4.8)	
Major amputation	79	HbA1c, mean(SD)		48.8 (10.1)	2	48.9 (9.8)	
Major amputation	79	HDL-cholesterol, mean(SD)		1.3 (0.4)	14.9	1.3 (0.4)	
Major amputation	79	Ischemic heart disease, n(%)	No	27159 (71.6%)	8.3	21088 (72%)	
Major amputation	79	Ischemic heart disease, n(%)	Yes	10767 (28.4%)	8.3	8193 (28%)	
Major amputation	79	Neuropathy, n(%)	No	28979 (70.1%)	0	18787 (64.2%)	
Major amputation	79	Neuropathy, n(%)	Yes	12376 (29.9%)	0	10494 (35.8%)	
Major amputation	79	Self-reported physical activity, n(%)	Never	5859 (16.2%)	12.3	4561 (15.6%)	
Major amputation	79	Self-reported physical activity, n(%)	<1 times/week	4613 (12.7%)	12.3	3550 (12.1%)	
Major amputation	79	Self-reported physical activity, n(%)	regularly - 1-2 times/week	6797 (18.8%)	12.3	5479 (18.7%)	
Major amputation	79	Self-reported physical activity, n(%)	regularly - 3-5 times/week	7591 (20.9%)	12.3	6277 (21.4%)	
Major amputation	79	Self-reported physical activity, n(%)	Daily	11389 (31.4%)	12.3	9414 (32.2%)	
Major amputation	79	Sex, n(%)	Males	21219 (51.3%)	0.1	15310 (52.3%)	n=41355 vs n=29281
Major amputation	79	Sex, n(%)	Females	20107 (48.7%)	0.1	13971 (47.7%)	
Major amputation	79	Smoking status, n(%)	No	35477 (92.7%)	7.5	27223 (93%)	

Major amputation	79	Smoking status, n(%)	Yes	2793 (7.3%)	7.5	2058 (7%)
Major amputation	79	Stroke, n(%)	No	33023 (87.8%)	9	25855 (88.3%)
Major amputation	79	Stroke, n(%)	Yes	4593 (12.2%)	9	3426 (11.7%)
Major amputation	79	Systolic blood pressure, mean(SD)		136.4 (16.1)	2.6	136.1 (15.9)
Major amputation	79	Time since last healthcare visit, mean(SD)		0.8 (1.2)	0	0.6 (1)
Major amputation	79	Total cholesterol, mean(SD)		4.6 (1.1)	10.4	4.6 (1.1)
Major amputation	80	Age at diagnosis of type 2 diabetes, mean(SD)		75.2 (3.1)	0	74.5 (3)
Major amputation	80	BMI, mean(SD)		28.6 (4.8)	9.6	28.6 (4.8)
Major amputation	80	HbA1c, mean(SD)		48.9 (10.3)	2.2	49 (10)
Major amputation	80	HDL-cholesterol, mean(SD)		1.3 (0.4)	15.5	1.3 (0.4)
Major amputation	80	Ischemic heart disease, n(%)	No	24933 (70.7%)	8.6	19242 (71.2%)
Major amputation	80	Ischemic heart disease, n(%)	Yes	10351 (29.3%)	8.6	7786 (28.8%)
Major amputation	80	Neuropathy, n(%)	No	26681 (69.1%)	0	16994 (62.9%)
Major amputation	80	Neuropathy, n(%)	Yes	11911 (30.9%)	0	10034 (37.1%)
Major amputation	80	Self-reported physical activity, n(%)	Never	5756 (17.1%)	12.9	4447 (16.5%)

Major amputation	80	Self-reported physical activity, n(%)	<1 times/week	4462 (13.3%)	12.9	3451 (12.8%)	
Major amputation	80	Self-reported physical activity, n(%)	regularly - 1-2 times/week	6403 (19%)	12.9	5123 (19%)	
Major amputation	80	Self-reported physical activity, n(%)	regularly - 3-5 times/week	6879 (20.5%)	12.9	5648 (20.9%)	
Major amputation	80	Self-reported physical activity, n(%)	Daily	10128 (30.1%)	12.9	8359 (30.9%)	
Major amputation	80	Sex, n(%)	Males	19376 (50.2%)	0.1	13800 (51.1%)	n=38592 vs n=27028
Major amputation	80	Sex, n(%)	Females	19188 (49.8%)	0.1	13228 (48.9%)	
Major amputation	80	Smoking status, n(%)	No	33158 (93.3%)	7.9	25280 (93.5%)	
Major amputation	80	Smoking status, n(%)	Yes	2385 (6.7%)	7.9	1748 (6.5%)	
Major amputation	80	Stroke, n(%)	No	30571 (87.3%)	9.3	23754 (87.9%)	
Major amputation	80	Stroke, n(%)	Yes	4439 (12.7%)	9.3	3274 (12.1%)	
Major amputation	80	Systolic blood pressure, mean(SD)		136.5 (16.2)	2.7	136.2 (15.9)	
Major amputation	80	Time since last healthcare visit, mean(SD)		0.8 (1.3)	0	0.6 (1)	
Major amputation	80	Total cholesterol, mean(SD)		4.6 (1.1)	10.9	4.6 (1.1)	
Major amputation	81	Age at diagnosis of type 2 diabetes, mean(SD)		76.2 (3.1)	0	75.5 (3)	
Major amputation	81	BMI, mean(SD)		28.5 (4.8)	10.2	28.5 (4.8)	

Major amputation	81	HbA1c, mean(SD)		49.1 (10.4)	2.3	49.2 (10.1)	
Major amputation	81	HDL-cholesterol, mean(SD)		1.3 (0.4)	16.8	1.3 (0.4)	
Major amputation	81	Ischemic heart disease, n(%)	No	22683 (69.7%)	8.8	17218 (70.4%)	
Major amputation	81	Ischemic heart disease, n(%)	Yes	9838 (30.3%)	8.8	7242 (29.6%)	
Major amputation	81	Neuropathy, n(%)	No	24361 (68.3%)	0	15015 (61.4%)	
Major amputation	81	Neuropathy, n(%)	Yes	11296 (31.7%)	0	9445 (38.6%)	
Major amputation	81	Self-reported physical activity, n(%)	Never	5464 (17.7%)	13.6	4151 (17%)	
Major amputation	81	Self-reported physical activity, n(%)	<1 times/week	4251 (13.8%)	13.6	3234 (13.2%)	
Major amputation	81	Self-reported physical activity, n(%)	regularly - 1-2 times/week	5939 (19.3%)	13.6	4717 (19.3%)	
Major amputation	81	Self-reported physical activity, n(%)	regularly - 3-5 times/week	6055 (19.7%)	13.6	4943 (20.2%)	
Major amputation	81	Self-reported physical activity, n(%)	Daily	9090 (29.5%)	13.6	7415 (30.3%)	
Major amputation	81	Sex, n(%)	Males	17527 (49.2%)	0.1	12285 (50.2%)	n=35657 vs n=24460
Major amputation	81	Sex, n(%)	Females	18101 (50.8%)	0.1	12175 (49.8%)	
Major amputation	81	Smoking status, n(%)	No	30645 (93.8%)	8.4	23012 (94.1%)	
Major amputation	81	Smoking status, n(%)	Yes	2023 (6.2%)	8.4	1448 (5.9%)	

Major amputation	81	Stroke, n(%)	No	27923 (86.7%)	9.6	21310 (87.1%)
Major amputation	81	Stroke, n(%)	Yes	4294 (13.3%)	9.6	3150 (12.9%)
Major amputation	81	Systolic blood pressure, mean(SD)		136.6 (16.4)	2.8	136.3 (16.1)
Major amputation	81	Time since last healthcare visit, mean(SD)		0.8 (1.3)	0	0.6 (1)
Major amputation	81	Total cholesterol, mean(SD)		4.7 (1.1)	11.8	4.6 (1.1)
Major amputation	82	Age at diagnosis of type 2 diabetes, mean(SD)		77.1 (3.1)	0	76.4 (3)
Major amputation	82	BMI, mean(SD)		28.4 (4.8)	10.5	28.4 (4.7)
Major amputation	82	HbA1c, mean(SD)		49.3 (10.4)	2.2	49.5 (10.2)
Major amputation	82	HDL-cholesterol, mean(SD)		1.3 (0.4)	17.8	1.3 (0.4)
Major amputation	82	Ischemic heart disease, n(%)	No	20456 (68.9%)	8.9	15306 (69.4%)
Major amputation	82	Ischemic heart disease, n(%)	Yes	9238 (31.1%)	8.9	6745 (30.6%)
Major amputation	82	Neuropathy, n(%)	No	21978 (67.4%)	0	13292 (60.3%)
Major amputation	82	Neuropathy, n(%)	Yes	10631 (32.6%)	0	8759 (39.7%)
Major amputation	82	Self-reported physical activity, n(%)	Never	5301 (18.9%)	14	3965 (18%)
Major amputation	82	Self-reported physical activity, n(%)	<1 times/week	3911 (14%)	14	2961 (13.4%)

Major amputation	82	Self-reported physical activity, n(%)	regularly - 1-2 times/week	5407 (19.3%)	14	4251 (19.3%)	
Major amputation	82	Self-reported physical activity, n(%)	regularly - 3-5 times/week	5478 (19.5%)	14	4477 (20.3%)	
Major amputation	82	Self-reported physical activity, n(%)	Daily	7931 (28.3%)	14	6397 (29%)	
Major amputation	82	Sex, n(%)	Males	15719 (48.2%)	0.1	10843 (49.2%)	n=32609 vs n=22051
Major amputation	82	Sex, n(%)	Females	16861 (51.8%)	0.1	11208 (50.8%)	
Major amputation	82	Smoking status, n(%)	No	28112 (94.4%)	8.7	20905 (94.8%)	
Major amputation	82	Smoking status, n(%)	Yes	1664 (5.6%)	8.7	1146 (5.2%)	
Major amputation	82	Stroke, n(%)	No	25302 (86.1%)	9.9	19066 (86.5%)	
Major amputation	82	Stroke, n(%)	Yes	4092 (13.9%)	9.9	2985 (13.5%)	
Major amputation	82	Systolic blood pressure, mean(SD)		136.8 (16.6)	2.9	136.5 (16.3)	
Major amputation	82	Time since last healthcare visit, mean(SD)		0.8 (1.3)	0	0.7 (1)	
Major amputation	82	Total cholesterol, mean(SD)		4.7 (1.1)	12.5	4.6 (1.1)	
Major amputation	83	Age at diagnosis of type 2 diabetes, mean(SD)		78.1 (3)	0	77.3 (3)	
Major amputation	83	BMI, mean(SD)		28.2 (4.7)	10.9	28.2 (4.7)	
Major amputation	83	HbA1c, mean(SD)		49.4 (10.5)	2.2	49.6 (10.3)	

Major amputation	83	HDL-cholesterol, mean(SD)		1.3 (0.4)	19	1.3 (0.4)	
Major amputation	83	Ischemic heart disease, n(%)	No	18319 (67.9%)	9.1	13539 (68.5%)	
Major amputation	83	Ischemic heart disease, n(%)	Yes	8659 (32.1%)	9.1	6216 (31.5%)	
Major amputation	83	Neuropathy, n(%)	No	19682 (66.3%)	0	11599 (58.7%)	
Major amputation	83	Neuropathy, n(%)	Yes	9986 (33.7%)	0	8156 (41.3%)	
Major amputation	83	Self-reported physical activity, n(%)	Never	5054 (20%)	14.7	3763 (19%)	
Major amputation	83	Self-reported physical activity, n(%)	<1 times/week	3520 (13.9%)	14.7	2641 (13.4%)	
Major amputation	83	Self-reported physical activity, n(%)	regularly - 1-2 times/week	5033 (19.9%)	14.7	3948 (20%)	
Major amputation	83	Self-reported physical activity, n(%)	regularly - 3-5 times/week	4753 (18.8%)	14.7	3807 (19.3%)	
Major amputation	83	Self-reported physical activity, n(%)	Daily	6956 (27.5%)	14.7	5596 (28.3%)	
Major amputation	83	Sex, n(%)	Males	13982 (47.2%)	0.1	9468 (47.9%)	n=29668 vs n=19755
Major amputation	83	Sex, n(%)	Females	15664 (52.8%)	0.1	10287 (52.1%)	
Major amputation	83	Smoking status, n(%)	No	25571 (94.7%)	9	18771 (95%)	
Major amputation	83	Smoking status, n(%)	Yes	1436 (5.3%)	9	984 (5%)	
Major amputation	83	Stroke, n(%)	No	22837 (85.5%)	10	16946 (85.8%)	

Major amputation	83	Stroke, n(%)	Yes	3877 (14.5%)	10	2809 (14.2%)
Major amputation	83	Systolic blood pressure, mean(SD)		136.8 (16.8)	2.8	136.4 (16.5)
Major amputation	83	Time since last healthcare visit, mean(SD)		0.8 (1.3)	0	0.7 (1.1)
Major amputation	83	Total cholesterol, mean(SD)		4.7 (1.1)	13.4	4.6 (1.1)
Major amputation	84	Age at diagnosis of type 2 diabetes, mean(SD)		79.1 (3.1)	0	78.2 (3)
Major amputation	84	BMI, mean(SD)		28 (4.6)	11.8	28.1 (4.6)
Major amputation	84	HbA1c, mean(SD)		49.6 (10.7)	2.6	49.7 (10.4)
Major amputation	84	HDL-cholesterol, mean(SD)		1.3 (0.4)	20.1	1.3 (0.4)
Major amputation	84	Ischemic heart disease, n(%)	No	16177 (66.7%)	9.3	11681 (67.1%)
Major amputation	84	Ischemic heart disease, n(%)	Yes	8059 (33.3%)	9.3	5716 (32.9%)
Major amputation	84	Neuropathy, n(%)	No	17496 (65.5%)	0	9921 (57%)
Major amputation	84	Neuropathy, n(%)	Yes	9223 (34.5%)	0	7476 (43%)
Major amputation	84	Self-reported physical activity, n(%)	Never	4857 (21.5%)	15.5	3590 (20.6%)
Major amputation	84	Self-reported physical activity, n(%)	<1 times/week	3278 (14.5%)	15.5	2427 (14%)
Major amputation	84	Self-reported physical activity, n(%)	regularly - 1-2 times/week	4366 (19.3%)	15.5	3362 (19.3%)

Major amputation	84	Self-reported physical activity, n(%)	regularly - 3-5 times/week	4158 (18.4%)	15.5	3310 (19%)	
Major amputation	84	Self-reported physical activity, n(%)	Daily	5908 (26.2%)	15.5	4708 (27.1%)	
Major amputation	84	Sex, n(%)	Males	12266 (45.9%)	0.1	8136 (46.8%)	n=26719 vs n=17397
Major amputation	84	Sex, n(%)	Females	14430 (54.1%)	0.1	9261 (53.2%)	
Major amputation	84	Smoking status, n(%)	No	22934 (95.1%)	9.8	16612 (95.5%)	
Major amputation	84	Smoking status, n(%)	Yes	1174 (4.9%)	9.8	785 (4.5%)	
Major amputation	84	Stroke, n(%)	No	20416 (85.1%)	10.2	14880 (85.5%)	
Major amputation	84	Stroke, n(%)	Yes	3576 (14.9%)	10.2	2517 (14.5%)	
Major amputation	84	Systolic blood pressure, mean(SD)		136.9 (17)	3.2	136.5 (16.7)	
Major amputation	84	Time since last healthcare visit, mean(SD)		0.9 (1.4)	0	0.7 (1.1)	
Major amputation	84	Total cholesterol, mean(SD)		4.7 (1.1)	14.5	4.6 (1.1)	
Major amputation	85	Age at diagnosis of type 2 diabetes, mean(SD)		80 (3.1)	0	79.2 (3)	
Major amputation	85	BMI, mean(SD)		27.9 (4.6)	12.5	27.9 (4.6)	
Major amputation	85	HbA1c, mean(SD)		49.6 (10.6)	2.7	49.7 (10.3)	
Major amputation	85	HDL-cholesterol, mean(SD)		1.3 (0.4)	21.3	1.3 (0.4)	

Major amputation	85	Ischemic heart disease, n(%)	No	14216 (65.8%)	9.6	10061 (66.1%)	
Major amputation	85	Ischemic heart disease, n(%)	Yes	7398 (34.2%)	9.6	5158 (33.9%)	
Major amputation	85	Neuropathy, n(%)	No	15453 (64.6%)	0	8478 (55.7%)	
Major amputation	85	Neuropathy, n(%)	Yes	8469 (35.4%)	0	6741 (44.3%)	
Major amputation	85	Self-reported physical activity, n(%)	Never	4471 (22.4%)	16.4	3274 (21.5%)	
Major amputation	85	Self-reported physical activity, n(%)	<1 times/week	2989 (14.9%)	16.4	2192 (14.4%)	
Major amputation	85	Self-reported physical activity, n(%)	regularly - 1-2 times/week	3925 (19.6%)	16.4	3003 (19.7%)	
Major amputation	85	Self-reported physical activity, n(%)	regularly - 3-5 times/week	3538 (17.7%)	16.4	2794 (18.4%)	
Major amputation	85	Self-reported physical activity, n(%)	Daily	5071 (25.4%)	16.4	3956 (26%)	
Major amputation	85	Sex, n(%)	Males	10693 (44.7%)	0.1	6960 (45.7%)	n=23922 vs n=15219
Major amputation	85	Sex, n(%)	Females	13206 (55.3%)	0.1	8259 (54.3%)	
Major amputation	85	Smoking status, n(%)	No	20496 (95.6%)	10.4	14603 (96%)	
Major amputation	85	Smoking status, n(%)	Yes	943 (4.4%)	10.4	616 (4%)	
Major amputation	85	Stroke, n(%)	No	18105 (84.6%)	10.5	12933 (85%)	
Major amputation	85	Stroke, n(%)	Yes	3299 (15.4%)	10.5	2286 (15%)	

Major amputation	85	Systolic blood pressure, mean(SD)		137.1 (17.1)	3.1	136.8 (16.8)
Major amputation	85	Time since last healthcare visit, mean(SD)		0.9 (1.4)	0	0.7 (1.1)
Major amputation	85	Total cholesterol, mean(SD)		4.7 (1.2)	15.7	4.7 (1.1)
Major amputation	86	Age at diagnosis of type 2 diabetes, mean(SD)		81 (3.1)	0	80.1 (3)
Major amputation	86	BMI, mean(SD)		27.7 (4.6)	13.3	27.7 (4.6)
Major amputation	86	HbA1c, mean(SD)		49.8 (10.9)	2.9	49.9 (10.6)
Major amputation	86	HDL-cholesterol, mean(SD)		1.3 (0.4)	22.9	1.3 (0.4)
Major amputation	86	Ischemic heart disease, n(%)	No	12432 (65.2%)	9.9	8592 (65.4%)
Major amputation	86	Ischemic heart disease, n(%)	Yes	6627 (34.8%)	9.9	4537 (34.6%)
Major amputation	86	Neuropathy, n(%)	No	13511 (63.9%)	0	7113 (54.2%)
Major amputation	86	Neuropathy, n(%)	Yes	7632 (36.1%)	0	6016 (45.8%)
Major amputation	86	Self-reported physical activity, n(%)	Never	4067 (23.2%)	17.2	2911 (22.2%)
Major amputation	86	Self-reported physical activity, n(%)	<1 times/week	2634 (15.1%)	17.2	1917 (14.6%)
Major amputation	86	Self-reported physical activity, n(%)	regularly - 1-2 times/week	3420 (19.5%)	17.2	2575 (19.6%)
Major amputation	86	Self-reported physical activity, n(%)	regularly - 3-5 times/week	3043 (17.4%)	17.2	2370 (18.1%)

Major amputation	86	Self-reported physical activity, n(%)	Daily	4333 (24.8%)	17.2	3356 (25.6%)	
Major amputation	86	Sex, n(%)	Males	9121 (43.2%)	0.1	5835 (44.4%)	n=21143 vs n=13129
Major amputation	86	Sex, n(%)	Females	12004 (56.8%)	0.1	7294 (55.6%)	
Major amputation	86	Smoking status, n(%)	No	18079 (95.9%)	10.8	12633 (96.2%)	
Major amputation	86	Smoking status, n(%)	Yes	775 (4.1%)	10.8	496 (3.8%)	
Major amputation	86	Stroke, n(%)	No	15916 (84.3%)	10.8	11094 (84.5%)	
Major amputation	86	Stroke, n(%)	Yes	2954 (15.7%)	10.8	2035 (15.5%)	
Major amputation	86	Systolic blood pressure, mean(SD)		137.2 (17.3)	3.4	136.9 (17.1)	
Major amputation	86	Time since last healthcare visit, mean(SD)		0.9 (1.5)	0	0.8 (1.2)	
Major amputation	86	Total cholesterol, mean(SD)		4.7 (1.1)	17.1	4.7 (1.1)	
Major amputation	87	Age at diagnosis of type 2 diabetes, mean(SD)		82 (3.1)	0	81 (3)	
Major amputation	87	BMI, mean(SD)		27.5 (4.5)	14.3	27.5 (4.4)	
Major amputation	87	HbA1c, mean(SD)		49.9 (10.9)	3	50 (10.6)	
Major amputation	87	HDL-cholesterol, mean(SD)		1.3 (0.4)	25	1.4 (0.4)	
Major amputation	87	Ischemic heart disease, n(%)	No	10569 (64.4%)	10.5	7163 (65%)	

Major amputation	87	Ischemic heart disease, n(%)	Yes	5837 (35.6%)	10.5	3861 (35%)	
Major amputation	87	Neuropathy, n(%)	No	11601 (63.3%)	0	5812 (52.7%)	
Major amputation	87	Neuropathy, n(%)	Yes	6720 (36.7%)	0	5212 (47.3%)	
Major amputation	87	Self-reported physical activity, n(%)	Never	3652 (24.5%)	18.7	2573 (23.3%)	
Major amputation	87	Self-reported physical activity, n(%)	<1 times/week	2267 (15.2%)	18.7	1608 (14.6%)	
Major amputation	87	Self-reported physical activity, n(%)	regularly - 1-2 times/week	2991 (20.1%)	18.7	2204 (20%)	
Major amputation	87	Self-reported physical activity, n(%)	regularly - 3-5 times/week	2565 (17.2%)	18.7	1990 (18.1%)	
Major amputation	87	Self-reported physical activity, n(%)	Daily	3425 (23%)	18.7	2649 (24%)	
Major amputation	87	Sex, n(%)	Males	7671 (41.9%)	0.1	4747 (43.1%)	n=18321 vs n=11024
Major amputation	87	Sex, n(%)	Females	10631 (58.1%)	0.1	6277 (56.9%)	
Major amputation	87	Smoking status, n(%)	No	15587 (96.3%)	11.7	10667 (96.8%)	
Major amputation	87	Smoking status, n(%)	Yes	591 (3.7%)	11.7	357 (3.2%)	
Major amputation	87	Stroke, n(%)	No	13617 (83.9%)	11.4	9314 (84.5%)	
Major amputation	87	Stroke, n(%)	Yes	2611 (16.1%)	11.4	1710 (15.5%)	
Major amputation	87	Systolic blood pressure, mean(SD)		137.3 (17.5)	3.7	136.9 (17.2)	

Major amputation	87	Time since last healthcare visit, mean(SD)		1 (1.5)	0	0.8 (1.2)
Major amputation	87	Total cholesterol, mean(SD)		4.8 (1.2)	18.9	4.7 (1.2)
Major amputation	88	Age at diagnosis of type 2 diabetes, mean(SD)		82.9 (3.1)	0	81.9 (3)
Major amputation	88	BMI, mean(SD)		27.3 (4.5)	15.3	27.3 (4.4)
Major amputation	88	HbA1c, mean(SD)		50 (11)	3.1	50.2 (10.8)
Major amputation	88	HDL-cholesterol, mean(SD)		1.3 (0.4)	26.8	1.4 (0.4)
Major amputation	88	Ischemic heart disease, n(%)	No	9026 (64%)	10.5	5881 (64.2%)
Major amputation	88	Ischemic heart disease, n(%)	Yes	5082 (36%)	10.5	3282 (35.8%)
Major amputation	88	Neuropathy, n(%)	No	9960 (63.2%)	0	4733 (51.7%)
Major amputation	88	Neuropathy, n(%)	Yes	5804 (36.8%)	0	4430 (48.3%)
Major amputation	88	Self-reported physical activity, n(%)	Never	3309 (26.2%)	19.8	2251 (24.6%)
Major amputation	88	Self-reported physical activity, n(%)	<1 times/week	2037 (16.1%)	19.8	1441 (15.7%)
Major amputation	88	Self-reported physical activity, n(%)	regularly - 1-2 times/week	2428 (19.2%)	19.8	1801 (19.7%)
Major amputation	88	Self-reported physical activity, n(%)	regularly - 3-5 times/week	2010 (15.9%)	19.8	1523 (16.6%)
Major amputation	88	Self-reported physical activity, n(%)	Daily	2860 (22.6%)	19.8	2147 (23.4%)

Major amputation	88	Sex, n(%)	Males	6430 (40.8%)	0.1	3886 (42.4%)	n=15764 vs n=9163
Major amputation	88	Sex, n(%)	Females	9322 (59.2%)	0.1	5277 (57.6%)	
Major amputation	88	Smoking status, n(%)	No	13344 (96.7%)	12.4	8889 (97%)	
Major amputation	88	Smoking status, n(%)	Yes	458 (3.3%)	12.4	274 (3%)	
Major amputation	88	Stroke, n(%)	No	11644 (83.5%)	11.5	7710 (84.1%)	
Major amputation	88	Stroke, n(%)	Yes	2300 (16.5%)	11.5	1453 (15.9%)	
Major amputation	88	Systolic blood pressure, mean(SD)		137.4 (17.6)	3.9	136.8 (17.2)	
Major amputation	88	Time since last healthcare visit, mean(SD)		1.1 (1.6)	0	0.8 (1.3)	
Major amputation	88	Total cholesterol, mean(SD)		4.8 (1.2)	20.6	4.7 (1.2)	
Major amputation	89	Age at diagnosis of type 2 diabetes, mean(SD)		83.8 (3.1)	0	82.7 (2.9)	
Major amputation	89	BMI, mean(SD)		27.1 (4.5)	16.7	27 (4.3)	
Major amputation	89	HbA1c, mean(SD)		50.2 (11.1)	3.3	50.2 (10.5)	
Major amputation	89	HDL-cholesterol, mean(SD)		1.3 (0.4)	28.8	1.4 (0.4)	
Major amputation	89	Ischemic heart disease, n(%)	No	7351 (63.3%)	10.9	4626 (63.2%)	
Major amputation	89	Ischemic heart disease, n(%)	Yes	4263 (36.7%)	10.9	2689 (36.8%)	

Major amputation	89	Neuropathy, n(%)	No	8208 (63%)	0	3674 (50.2%)	
Major amputation	89	Neuropathy, n(%)	Yes	4827 (37%)	0	3641 (49.8%)	
Major amputation	89	Self-reported physical activity, n(%)	Never	2795 (27.1%)	20.9	1841 (25.2%)	
Major amputation	89	Self-reported physical activity, n(%)	<1 times/week	1718 (16.7%)	20.9	1201 (16.4%)	
Major amputation	89	Self-reported physical activity, n(%)	regularly - 1-2 times/week	1932 (18.7%)	20.9	1382 (18.9%)	
Major amputation	89	Self-reported physical activity, n(%)	regularly - 3-5 times/week	1606 (15.6%)	20.9	1208 (16.5%)	
Major amputation	89	Self-reported physical activity, n(%)	Daily	2254 (21.9%)	20.9	1683 (23%)	
Major amputation	89	Sex, n(%)	Males	5146 (39.5%)	0.1	3003 (41.1%)	n=13035 vs n=7315
Major amputation	89	Sex, n(%)	Females	7879 (60.5%)	0.1	4312 (58.9%)	
Major amputation	89	Smoking status, n(%)	No	10944 (96.9%)	13.4	7108 (97.2%)	
Major amputation	89	Smoking status, n(%)	Yes	347 (3.1%)	13.4	207 (2.8%)	
Major amputation	89	Stroke, n(%)	No	9672 (84.3%)	12	6203 (84.8%)	
Major amputation	89	Stroke, n(%)	Yes	1800 (15.7%)	12	1112 (15.2%)	
Major amputation	89	Systolic blood pressure, mean(SD)		137.4 (17.8)	4.2	137.1 (17.5)	
Major amputation	89	Time since last healthcare visit, mean(SD)		1.1 (1.6)	0	0.9 (1.3)	

Major amputation	89	Total cholesterol, mean(SD)		4.8 (1.2)	22.4	4.8 (1.2)	
Major amputation	90	Age at diagnosis of type 2 diabetes, mean(SD)		84.9 (3.1)	0	83.7 (2.9)	
Major amputation	90	BMI, mean(SD)		26.8 (4.3)	17.9	26.8 (4.3)	
Major amputation	90	HbA1c, mean(SD)		50.3 (11.2)	3.6	50.4 (10.8)	
Major amputation	90	HDL-cholesterol, mean(SD)		1.4 (0.4)	30.7	1.4 (0.4)	
Major amputation	90	Ischemic heart disease, n(%)	No	5889 (62.9%)	10.8	3558 (62.8%)	
Major amputation	90	Ischemic heart disease, n(%)	Yes	3471 (37.1%)	10.8	2112 (37.2%)	
Major amputation	90	Neuropathy, n(%)	No	6690 (63.8%)	0	2834 (50%)	
Major amputation	90	Neuropathy, n(%)	Yes	3802 (36.2%)	0	2836 (50%)	
Major amputation	90	Self-reported physical activity, n(%)	Never	2285 (28%)	22.3	1488 (26.2%)	
Major amputation	90	Self-reported physical activity, n(%)	<1 times/week	1377 (16.9%)	22.3	937 (16.5%)	
Major amputation	90	Self-reported physical activity, n(%)	regularly - 1-2 times/week	1532 (18.8%)	22.3	1054 (18.6%)	
Major amputation	90	Self-reported physical activity, n(%)	regularly - 3-5 times/week	1204 (14.8%)	22.3	875 (15.4%)	
Major amputation	90	Self-reported physical activity, n(%)	Daily	1759 (21.6%)	22.3	1316 (23.2%)	
Major amputation	90	Sex, n(%)	Males	3998 (38.1%)	0.1	2300 (40.6%)	n=10492 vs n=5670

Major amputation	90	Sex, n(%)	Females	6486 (61.9%)	0.1	3370 (59.4%)
Major amputation	90	Smoking status, n(%)	No	8704 (97.2%)	14.7	5535 (97.6%)
Major amputation	90	Smoking status, n(%)	Yes	247 (2.8%)	14.7	135 (2.4%)
Major amputation	90	Stroke, n(%)	No	7808 (84.7%)	12.2	4841 (85.4%)
Major amputation	90	Stroke, n(%)	Yes	1409 (15.3%)	12.2	829 (14.6%)
Major amputation	90	Systolic blood pressure, mean(SD)		137.9 (17.8)	4.3	137.8 (17.5)
Major amputation	90	Time since last healthcare visit, mean(SD)		1.2 (1.6)	0	0.9 (1.3)
Major amputation	90	Total cholesterol, mean(SD)		4.8 (1.2)	24.6	4.8 (1.2)
Major amputation	91	Age at diagnosis of type 2 diabetes, mean(SD)		85.7 (3.1)	0	84.6 (2.9)
Major amputation	91	BMI, mean(SD)		26.6 (4.3)	19	26.6 (4.2)
Major amputation	91	HbA1c, mean(SD)		50.6 (11.6)	4	50.7 (11)
Major amputation	91	HDL-cholesterol, mean(SD)		1.4 (0.4)	32.5	1.3 (0.4)
Major amputation	91	Ischemic heart disease, n(%)	No	4665 (62.7%)	10.6	2717 (62.5%)
Major amputation	91	Ischemic heart disease, n(%)	Yes	2780 (37.3%)	10.6	1630 (37.5%)
Major amputation	91	Neuropathy, n(%)	No	5342 (64.2%)	0	2158 (49.6%)

Major amputation	91	Neuropathy, n(%)	Yes	2985 (35.8%)	0	2189 (50.4%)	
Major amputation	91	Self-reported physical activity, n(%)	Never	1879 (29.7%)	23.9	1193 (27.4%)	
Major amputation	91	Self-reported physical activity, n(%)	<1 times/week	1039 (16.4%)	23.9	709 (16.3%)	
Major amputation	91	Self-reported physical activity, n(%)	regularly - 1-2 times/week	1193 (18.8%)	23.9	824 (19%)	
Major amputation	91	Self-reported physical activity, n(%)	regularly - 3-5 times/week	897 (14.2%)	23.9	651 (15%)	
Major amputation	91	Self-reported physical activity, n(%)	Daily	1327 (20.9%)	23.9	970 (22.3%)	
Major amputation	91	Sex, n(%)	Males	3040 (36.5%)	0.1	1738 (40%)	n=8327 vs n=4347
Major amputation	91	Sex, n(%)	Females	5281 (63.5%)	0.1	2609 (60%)	
Major amputation	91	Smoking status, n(%)	No	6818 (97.5%)	16	4248 (97.7%)	
Major amputation	91	Smoking status, n(%)	Yes	175 (2.5%)	16	99 (2.3%)	
Major amputation	91	Stroke, n(%)	No	6179 (84.4%)	12.1	3694 (85%)	
Major amputation	91	Stroke, n(%)	Yes	1143 (15.6%)	12.1	653 (15%)	
Major amputation	91	Systolic blood pressure, mean(SD)		137.8 (17.9)	4.4	137.6 (17.6)	
Major amputation	91	Time since last healthcare visit, mean(SD)		1.2 (1.7)	0	1 (1.4)	
Major amputation	91	Total cholesterol, mean(SD)		4.9 (1.2)	26.3	4.8 (1.2)	

Major amputation	92	Age at diagnosis of type 2 diabetes, mean(SD)		86.7 (3.1)	0	85.4 (2.9)	
Major amputation	92	BMI, mean(SD)		26.5 (4.3)	20.9	26.4 (4.3)	
Major amputation	92	HbA1c, mean(SD)		50.6 (11.5)	4.2	50.7 (11.3)	
Major amputation	92	HDL-cholesterol, mean(SD)		1.4 (0.4)	35.1	1.4 (0.4)	
Major amputation	92	Ischemic heart disease, n(%)	No	3517 (62.5%)	11.7	1981 (63.2%)	
Major amputation	92	Ischemic heart disease, n(%)	Yes	2113 (37.5%)	11.7	1155 (36.8%)	
Major amputation	92	Neuropathy, n(%)	No	4153 (65.2%)	0	1534 (48.9%)	
Major amputation	92	Neuropathy, n(%)	Yes	2220 (34.8%)	0	1602 (51.1%)	
Major amputation	92	Self-reported physical activity, n(%)	Never	1442 (30.7%)	26.3	889 (28.3%)	
Major amputation	92	Self-reported physical activity, n(%)	<1 times/week	775 (16.5%)	26.3	510 (16.3%)	
Major amputation	92	Self-reported physical activity, n(%)	regularly - 1-2 times/week	887 (18.9%)	26.3	596 (19%)	
Major amputation	92	Self-reported physical activity, n(%)	regularly - 3-5 times/week	631 (13.4%)	26.3	448 (14.3%)	
Major amputation	92	Self-reported physical activity, n(%)	Daily	960 (20.4%)	26.3	693 (22.1%)	
Major amputation	92	Sex, n(%)	Males	2275 (35.7%)	0.1	1238 (39.5%)	n=6373 vs n=3136
Major amputation	92	Sex, n(%)	Females	4092 (64.3%)	0.1	1898 (60.5%)	

Major amputation	92	Smoking status, n(%)	No	5130 (97.8%)	17.7	3079 (98.2%)
Major amputation	92	Smoking status, n(%)	Yes	115 (2.2%)	17.7	57 (1.8%)
Major amputation	92	Stroke, n(%)	No	4668 (84.4%)	13.2	2657 (84.7%)
Major amputation	92	Stroke, n(%)	Yes	865 (15.6%)	13.2	479 (15.3%)
Major amputation	92	Systolic blood pressure, mean(SD)		137.8 (18)	5.1	137.4 (17.5)
Major amputation	92	Time since last healthcare visit, mean(SD)		1.3 (1.7)	0	1 (1.5)
Major amputation	92	Total cholesterol, mean(SD)		4.9 (1.2)	28.8	4.9 (1.2)
Major amputation	93	Age at diagnosis of type 2 diabetes, mean(SD)		87.6 (3.1)	0	86.3 (2.8)
Major amputation	93	BMI, mean(SD)		26.3 (4.3)	22.5	26.2 (4.2)
Major amputation	93	HbA1c, mean(SD)		50.6 (11.5)	5	50.8 (11)
Major amputation	93	HDL-cholesterol, mean(SD)		1.4 (0.4)	37.2	1.4 (0.4)
Major amputation	93	Ischemic heart disease, n(%)	No	2627 (63%)	12.2	1425 (64.1%)
Major amputation	93	Ischemic heart disease, n(%)	Yes	1541 (37%)	12.2	797 (35.9%)
Major amputation	93	Neuropathy, n(%)	No	3080 (64.9%)	0	1052 (47.3%)
Major amputation	93	Neuropathy, n(%)	Yes	1665 (35.1%)	0	1170 (52.7%)

Major amputation	93	Self-reported physical activity, n(%)	Never	1089 (32%)	28.3	634 (28.5%)	
Major amputation	93	Self-reported physical activity, n(%)	<1 times/week	530 (15.6%)	28.3	364 (16.4%)	
Major amputation	93	Self-reported physical activity, n(%)	regularly - 1-2 times/week	622 (18.3%)	28.3	402 (18.1%)	
Major amputation	93	Self-reported physical activity, n(%)	regularly - 3-5 times/week	465 (13.7%)	28.3	322 (14.5%)	
Major amputation	93	Self-reported physical activity, n(%)	Daily	694 (20.4%)	28.3	500 (22.5%)	
Major amputation	93	Sex, n(%)	Males	1657 (34.9%)	0.1	854 (38.4%)	n=4745 vs n=2222
Major amputation	93	Sex, n(%)	Females	3085 (65.1%)	0.1	1368 (61.6%)	
Major amputation	93	Smoking status, n(%)	No	3785 (98%)	18.6	2187 (98.4%)	
Major amputation	93	Smoking status, n(%)	Yes	76 (2%)	18.6	35 (1.6%)	
Major amputation	93	Stroke, n(%)	No	3471 (84.9%)	13.9	1906 (85.8%)	
Major amputation	93	Stroke, n(%)	Yes	615 (15.1%)	13.9	316 (14.2%)	
Major amputation	93	Systolic blood pressure, mean(SD)		137.9 (18.5)	5.8	137.3 (18.1)	
Major amputation	93	Time since last healthcare visit, mean(SD)		1.4 (1.8)	0	1.1 (1.5)	
Major amputation	93	Total cholesterol, mean(SD)		4.9 (1.2)	30.8	4.9 (1.2)	
Major amputation	94	Age at diagnosis of type 2 diabetes, mean(SD)		88.6 (3.1)	0	87.2 (2.8)	

Major amputation	94	BMI, mean(SD)		26 (4.2)	24.5	26 (4.1)	
Major amputation	94	HbA1c, mean(SD)		50.7 (11.4)	5.4	50.9 (11.4)	
Major amputation	94	HDL-cholesterol, mean(SD)		1.4 (0.4)	39.9	1.4 (0.4)	
Major amputation	94	Ischemic heart disease, n(%)	No	1894 (62.9%)	12.8	961 (63.2%)	
Major amputation	94	Ischemic heart disease, n(%)	Yes	1119 (37.1%)	12.8	559 (36.8%)	
Major amputation	94	Neuropathy, n(%)	No	2258 (65.4%)	0	719 (47.3%)	
Major amputation	94	Neuropathy, n(%)	Yes	1197 (34.6%)	0	801 (52.7%)	
Major amputation	94	Self-reported physical activity, n(%)	Never	797 (33.2%)	30.4	439 (28.9%)	
Major amputation	94	Self-reported physical activity, n(%)	<1 times/week	396 (16.5%)	30.4	258 (17%)	
Major amputation	94	Self-reported physical activity, n(%)	regularly - 1-2 times/week	432 (18%)	30.4	286 (18.8%)	
Major amputation	94	Self-reported physical activity, n(%)	regularly - 3-5 times/week	317 (13.2%)	30.4	220 (14.5%)	
Major amputation	94	Self-reported physical activity, n(%)	Daily	462 (19.2%)	30.4	317 (20.9%)	
Major amputation	94	Sex, n(%)	Males	1121 (32.5%)	0	549 (36.1%)	n=3455 vs n=1520
Major amputation	94	Sex, n(%)	Females	2333 (67.5%)	0	971 (63.9%)	
Major amputation	94	Smoking status, n(%)	No	2703 (97.8%)	20	1491 (98.1%)	

Major amputation	94	Smoking status, n(%)	Yes	60 (2.2%)	20	29 (1.9%)
Major amputation	94	Stroke, n(%)	No	2492 (84.4%)	14.6	1308 (86.1%)
Major amputation	94	Stroke, n(%)	Yes	459 (15.6%)	14.6	212 (13.9%)
Major amputation	94	Systolic blood pressure, mean(SD)		138.3 (18.1)	6	137.6 (17.7)
Major amputation	94	Time since last healthcare visit, mean(SD)		1.5 (1.9)	0	1.2 (1.6)
Major amputation	94	Total cholesterol, mean(SD)		4.9 (1.2)	33.3	4.9 (1.2)
Major amputation	95	Age at diagnosis of type 2 diabetes, mean(SD)		89.5 (3.1)	0	87.9 (2.7)
Major amputation	95	BMI, mean(SD)		25.9 (4.2)	26.1	25.8 (4.1)
Major amputation	95	HbA1c, mean(SD)		50.7 (11.4)	6.4	50.6 (11.2)
Major amputation	95	HDL-cholesterol, mean(SD)		1.4 (0.4)	43.4	1.4 (0.4)
Major amputation	95	Ischemic heart disease, n(%)	No	1355 (63.9%)	13.5	639 (64.1%)
Major amputation	95	Ischemic heart disease, n(%)	Yes	765 (36.1%)	13.5	358 (35.9%)
Major amputation	95	Neuropathy, n(%)	No	1641 (67%)	0	472 (47.3%)
Major amputation	95	Neuropathy, n(%)	Yes	809 (33%)	0	525 (52.7%)
Major amputation	95	Self-reported physical activity, n(%)	Never	579 (35.3%)	33.1	297 (29.8%)

Major amputation	95	Self-reported physical activity, n(%)	<1 times/week	236 (14.4%)	33.1	143 (14.3%)	
Major amputation	95	Self-reported physical activity, n(%)	regularly - 1-2 times/week	280 (17.1%)	33.1	174 (17.5%)	
Major amputation	95	Self-reported physical activity, n(%)	regularly - 3-5 times/week	226 (13.8%)	33.1	154 (15.4%)	
Major amputation	95	Self-reported physical activity, n(%)	Daily	319 (19.5%)	33.1	229 (23%)	
Major amputation	95	Sex, n(%)	Males	731 (29.8%)	0	333 (33.4%)	n=2450 vs n=997
Major amputation	95	Sex, n(%)	Females	1718 (70.2%)	0	664 (66.6%)	
Major amputation	95	Smoking status, n(%)	No	1874 (98.1%)	22	981 (98.4%)	
Major amputation	95	Smoking status, n(%)	Yes	37 (1.9%)	22	16 (1.6%)	
Major amputation	95	Stroke, n(%)	No	1761 (84.8%)	15.2	863 (86.6%)	
Major amputation	95	Stroke, n(%)	Yes	316 (15.2%)	15.2	134 (13.4%)	
Major amputation	95	Systolic blood pressure, mean(SD)		138 (18.3)	6.5	137.4 (18.2)	
Major amputation	95	Time since last healthcare visit, mean(SD)		1.6 (2)	0	1.3 (1.7)	
Major amputation	95	Total cholesterol, mean(SD)		5 (1.2)	36.5	4.9 (1.2)	
Major amputation	96	Age at diagnosis of type 2 diabetes, mean(SD)		90.4 (3.1)	0	88.9 (2.7)	
Major amputation	96	BMI, mean(SD)		25.7 (4.1)	28.8	25.4 (3.9)	

Major amputation	96	HbA1c, mean(SD)		50.8 (11.4)	7	50.6 (11)	
Major amputation	96	HDL-cholesterol, mean(SD)		1.4 (0.4)	46.1	1.4 (0.4)	
Major amputation	96	Ischemic heart disease, n(%)	No	886 (64.1%)	14.5	390 (63.7%)	
Major amputation	96	Ischemic heart disease, n(%)	Yes	496 (35.9%)	14.5	222 (36.3%)	
Major amputation	96	Neuropathy, n(%)	No	1117 (69.1%)	0	285 (46.6%)	
Major amputation	96	Neuropathy, n(%)	Yes	500 (30.9%)	0	327 (53.4%)	
Major amputation	96	Self-reported physical activity, n(%)	Never	395 (38.3%)	36.3	208 (34%)	
Major amputation	96	Self-reported physical activity, n(%)	<1 times/week	149 (14.5%)	36.3	89 (14.5%)	
Major amputation	96	Self-reported physical activity, n(%)	regularly - 1-2 times/week	172 (16.7%)	36.3	105 (17.2%)	
Major amputation	96	Self-reported physical activity, n(%)	regularly - 3-5 times/week	140 (13.6%)	36.3	93 (15.2%)	
Major amputation	96	Self-reported physical activity, n(%)	Daily	174 (16.9%)	36.3	117 (19.1%)	
Major amputation	96	Sex, n(%)	Males	462 (28.6%)	0	187 (30.6%)	n=1617 vs n=612
Major amputation	96	Sex, n(%)	Females	1155 (71.4%)	0	425 (69.4%)	
Major amputation	96	Smoking status, n(%)	No	1188 (98.1%)	25.1	600 (98%)	
Major amputation	96	Smoking status, n(%)	Yes	23 (1.9%)	25.1	12 (2%)	

Major amputation	96	Stroke, n(%)	No	1155 (84.9%)	15.9	535 (87.4%)
Major amputation	96	Stroke, n(%)	Yes	205 (15.1%)	15.9	77 (12.6%)
Major amputation	96	Systolic blood pressure, mean(SD)		138.7 (18.6)	7.2	138.1 (18.4)
Major amputation	96	Time since last healthcare visit, mean(SD)		1.7 (2.1)	0	1.3 (1.8)
Major amputation	96	Total cholesterol, mean(SD)		5 (1.2)	39.3	5 (1.2)
Major amputation	97	Age at diagnosis of type 2 diabetes, mean(SD)		91.4 (3.1)	0	89.8 (2.7)
Major amputation	97	BMI, mean(SD)		25.8 (4)	31.2	25.4 (3.8)
Major amputation	97	HbA1c, mean(SD)		50.5 (10.9)	7.1	50.8 (10.9)
Major amputation	97	HDL-cholesterol, mean(SD)		1.4 (0.4)	49.2	1.4 (0.4)
Major amputation	97	Ischemic heart disease, n(%)	No	558 (62.3%)	14.6	232 (61.7%)
Major amputation	97	Ischemic heart disease, n(%)	Yes	338 (37.7%)	14.6	144 (38.3%)
Major amputation	97	Neuropathy, n(%)	No	735 (70.1%)	0	174 (46.3%)
Major amputation	97	Neuropathy, n(%)	Yes	314 (29.9%)	0	202 (53.7%)
Major amputation	97	Self-reported physical activity, n(%)	Never	267 (40.5%)	37.1	138 (36.7%)
Major amputation	97	Self-reported physical activity, n(%)	<1 times/week	98 (14.8%)	37.1	50 (13.3%)

Major amputation	97	Self-reported physical activity, n(%)	regularly - 1-2 times/week	109 (16.5%)	37.1	68 (18.1%)	
Major amputation	97	Self-reported physical activity, n(%)	regularly - 3-5 times/week	85 (12.9%)	37.1	56 (14.9%)	
Major amputation	97	Self-reported physical activity, n(%)	Daily	101 (15.3%)	37.1	64 (17%)	
Major amputation	97	Sex, n(%)	Males	275 (26.2%)	0	112 (29.8%)	n=1049 vs n=376
Major amputation	97	Sex, n(%)	Females	774 (73.8%)	0	264 (70.2%)	
Major amputation	97	Smoking status, n(%)	No	760 (97.9%)	26	367 (97.6%)	
Major amputation	97	Smoking status, n(%)	Yes	16 (2.1%)	26	9 (2.4%)	
Major amputation	97	Stroke, n(%)	No	748 (84.7%)	15.8	324 (86.2%)	
Major amputation	97	Stroke, n(%)	Yes	135 (15.3%)	15.8	52 (13.8%)	
Major amputation	97	Systolic blood pressure, mean(SD)		138.4 (18.8)	6.2	136.9 (19.2)	
Major amputation	97	Time since last healthcare visit, mean(SD)		1.8 (2.1)	0	1.4 (1.7)	
Major amputation	97	Total cholesterol, mean(SD)		5.1 (1.1)	41.8	5 (1.1)	
Major amputation	98	Age at diagnosis of type 2 diabetes, mean(SD)		92.2 (3)	0	90.5 (2.8)	
Major amputation	98	BMI, mean(SD)		25.5 (3.9)	32.7	25.3 (3.7)	
Major amputation	98	HbA1c, mean(SD)		50.4 (11.6)	8.1	51.1 (11.1)	

Major amputation	98	HDL-cholesterol, mean(SD)		1.4 (0.4)	49	1.4 (0.5)	
Major amputation	98	Ischemic heart disease, n(%)	No	351 (62.8%)	14.1	142 (62.6%)	
Major amputation	98	Ischemic heart disease, n(%)	Yes	208 (37.2%)	14.1	85 (37.4%)	
Major amputation	98	Neuropathy, n(%)	No	469 (72%)	0	114 (50.2%)	
Major amputation	98	Neuropathy, n(%)	Yes	182 (28%)	0	113 (49.8%)	
Major amputation	98	Self-reported physical activity, n(%)	Never	166 (41.7%)	38.9	79 (34.8%)	
Major amputation	98	Self-reported physical activity, n(%)	<1 times/week	61 (15.3%)	38.9	36 (15.9%)	
Major amputation	98	Self-reported physical activity, n(%)	regularly - 1-2 times/week	69 (17.3%)	38.9	46 (20.3%)	
Major amputation	98	Self-reported physical activity, n(%)	regularly - 3-5 times/week	48 (12.1%)	38.9	28 (12.3%)	
Major amputation	98	Self-reported physical activity, n(%)	Daily	54 (13.6%)	38.9	38 (16.7%)	
Major amputation	98	Sex, n(%)	Males	153 (23.5%)	0	68 (30%)	n=651 vs n=227
Major amputation	98	Sex, n(%)	Females	498 (76.5%)	0	159 (70%)	
Major amputation	98	Smoking status, n(%)	No	466 (98.3%)	27.2	223 (98.2%)	
Major amputation	98	Smoking status, n(%)	Yes	8 (1.7%)	27.2	4 (1.8%)	
Major amputation	98	Stroke, n(%)	No	463 (84.2%)	15.5	190 (83.7%)	

Major amputation	98	Stroke, n(%)	Yes	87 (15.8%)	15.5	37 (16.3%)
Major amputation	98	Systolic blood pressure, mean(SD)		137.9 (18.4)	6.5	136.1 (17.9)
Major amputation	98	Time since last healthcare visit, mean(SD)		1.9 (2.2)	0	1.5 (1.9)
Major amputation	98	Total cholesterol, mean(SD)		5.1 (1.2)	41.5	5 (1.2)
Major amputation	99	Age at diagnosis of type 2 diabetes, mean(SD)		93.2 (3.1)	0	91.3 (2.8)
Major amputation	99	BMI, mean(SD)		25.6 (4.1)	35.1	25.1 (3.9)
Major amputation	99	HbA1c, mean(SD)		51.1 (12.9)	9.3	52.4 (12.9)
Major amputation	99	HDL-cholesterol, mean(SD)		1.4 (0.4)	51.6	1.3 (0.4)
Major amputation	99	Ischemic heart disease, n(%)	No	201 (62.8%)	14.9	74 (61.7%)
Major amputation	99	Ischemic heart disease, n(%)	Yes	119 (37.2%)	14.9	46 (38.3%)
Major amputation	99	Neuropathy, n(%)	No	280 (74.5%)	0	57 (47.5%)
Major amputation	99	Neuropathy, n(%)	Yes	96 (25.5%)	0	63 (52.5%)
Major amputation	99	Self-reported physical activity, n(%)	Never	104 (47.1%)	41.2	40 (33.3%)
Major amputation	99	Self-reported physical activity, n(%)	<1 times/week	30 (13.6%)	41.2	22 (18.3%)
Major amputation	99	Self-reported physical activity, n(%)	regularly - 1-2 times/week	34 (15.4%)	41.2	20 (16.7%)

Major amputation	99	Self-reported physical activity, n(%)	regularly - 3-5 times/week	19 (8.6%)	41.2	13 (10.8%)	
Major amputation	99	Self-reported physical activity, n(%)	Daily	34 (15.4%)	41.2	25 (20.8%)	
Major amputation	99	Sex, n(%)	Males	87 (23.1%)	0	29 (24.2%)	n=376 vs n=120
Major amputation	99	Sex, n(%)	Females	289 (76.9%)	0	91 (75.8%)	
Major amputation	99	Smoking status, n(%)	No	265 (98.1%)	28.2	119 (99.2%)	
Major amputation	99	Smoking status, n(%)	Yes	5 (1.9%)	28.2	1 (0.8%)	
Major amputation	99	Stroke, n(%)	No	272 (85.5%)	15.4	104 (86.7%)	
Major amputation	99	Stroke, n(%)	Yes	46 (14.5%)	15.4	16 (13.3%)	
Major amputation	99	Systolic blood pressure, mean(SD)		138.6 (18)	7.4	138 (16.2)	
Major amputation	99	Time since last healthcare visit, mean(SD)		2 (2.3)	0	1.6 (2)	
Major amputation	99	Total cholesterol, mean(SD)		5.3 (1.2)	45.7	5.1 (1.2)	
Major amputation	100	Age at diagnosis of type 2 diabetes, mean(SD)		94.2 (3.1)	0	92 (2.5)	
Major amputation	100	BMI, mean(SD)		25.5 (4.1)	40.4	25.2 (4.1)	
Major amputation	100	HbA1c, mean(SD)		51.8 (13.2)	12.5	53.7 (14.4)	
Major amputation	100	HDL-cholesterol, mean(SD)		1.3 (0.4)	60.1	1.3 (0.3)	

Major amputation	100	Ischemic heart disease, n(%)	No	112 (64%)	15.9	35 (63.6%)	
Major amputation	100	Ischemic heart disease, n(%)	Yes	63 (36%)	15.9	20 (36.4%)	
Major amputation	100	Neuropathy, n(%)	No	152 (73.1%)	0	24 (43.6%)	
Major amputation	100	Neuropathy, n(%)	Yes	56 (26.9%)	0	31 (56.4%)	
Major amputation	100	Self-reported physical activity, n(%)	Never	57 (48.7%)	43.8	16 (29.1%)	
Major amputation	100	Self-reported physical activity, n(%)	<1 times/week	19 (16.2%)	43.8	14 (25.5%)	
Major amputation	100	Self-reported physical activity, n(%)	regularly - 1-2 times/week	16 (13.7%)	43.8	10 (18.2%)	
Major amputation	100	Self-reported physical activity, n(%)	regularly - 3-5 times/week	11 (9.4%)	43.8	8 (14.5%)	
Major amputation	100	Self-reported physical activity, n(%)	Daily	14 (12%)	43.8	7 (12.7%)	
Major amputation	100	Sex, n(%)	Males	45 (21.6%)	0	11 (20%)	n=208 vs n=55
Major amputation	100	Sex, n(%)	Females	163 (78.4%)	0	44 (80%)	
Major amputation	100	Smoking status, n(%)	No	142 (97.9%)	30.3	55 (100%)	
Major amputation	100	Smoking status, n(%)	Yes	3 (2.1%)	30.3	0 (0%)	
Major amputation	100	Stroke, n(%)	No	153 (89%)	17.3	52 (94.5%)	
Major amputation	100	Stroke, n(%)	Yes	19 (11%)	17.3	3 (5.5%)	

Major amputation	100	Systolic blood pressure, mean(SD)		137.4 (17.6)	8.7	136.6 (15.8)	
Major amputation	100	Time since last healthcare visit, mean(SD)		2.2 (2.4)	0	1.3 (1.9)	
Major amputation	100	Total cholesterol, mean(SD)		5.2 (1.1)	54.3	5 (1.1)	
Neuropathy	19	Age at diagnosis of type 2 diabetes, mean(SD)		18.5 (0.3)	0	18.5 (0.3)	
Neuropathy	19	BMI, mean(SD)		34.2 (6.9)	17.7	35.1 (7.2)	
Neuropathy	19	HbA1c, mean(SD)		55.1 (19.7)	19.4	57.1 (19)	
Neuropathy	19	HDL-cholesterol, mean(SD)		0.9 (0.2)	46.8	0.9 (0.2)	
Neuropathy	19	Ischemic heart disease, n(%)	No	44 (100%)	29	17 (100%)	
Neuropathy	19	Self-reported physical activity, n(%)	Never	10 (25%)	35.5	5 (29.4%)	
Neuropathy	19	Self-reported physical activity, n(%)	<1 times/week	8 (20%)	35.5	2 (11.8%)	
Neuropathy	19	Self-reported physical activity, n(%)	regularly - 1-2 times/week	8 (20%)	35.5	4 (23.5%)	
Neuropathy	19	Self-reported physical activity, n(%)	regularly - 3-5 times/week	8 (20%)	35.5	3 (17.6%)	
Neuropathy	19	Self-reported physical activity, n(%)	Daily	6 (15%)	35.5	3 (17.6%)	
Neuropathy	19	Sex, n(%)	Males	22 (35.5%)	0	6 (35.3%)	n=62 vs n=17
Neuropathy	19	Sex, n(%)	Females	40 (64.5%)	0	11 (64.7%)	
Neuropathy	19	Smoking status, n(%)	No	28 (68.3%)	33.9	10 (58.8%)	
Neuropathy	19	Smoking status, n(%)	Yes	13 (31.7%)	33.9	7 (41.2%)	
Neuropathy	19	Stroke, n(%)	No	43 (100%)	30.6	17 (100%)	
Neuropathy	19	Systolic blood pressure, mean(SD)		124.6 (10.3)	22.6	124.1 (8.5)	
Neuropathy	19	Time since last healthcare visit, mean(SD)		0.3 (0.2)	0	0.1 (0.1)	
Neuropathy	19	Total cholesterol, mean(SD)		4.8 (1.1)	45.2	5 (1.1)	

Neuropathy	20	Age at diagnosis of type 2 diabetes, mean(SD)		19.1 (0.6)	0	19 (0.6)	
Neuropathy	20	BMI, mean(SD)		35.7 (8.1)	22.1	37 (8.3)	
Neuropathy	20	HbA1c, mean(SD)		55.1 (21.2)	15	57.5 (20.7)	
Neuropathy	20	HDL-cholesterol, mean(SD)		1 (0.3)	42.1	1 (0.3)	
Neuropathy	20	Ischemic heart disease, n(%)	No	115 (100%)	17.9	49 (100%)	
Neuropathy	20	Self-reported physical activity, n(%)	Never	16 (17.2%)	33.6	12 (24.5%)	
Neuropathy	20	Self-reported physical activity, n(%)	<1 times/week	12 (12.9%)	33.6	5 (10.2%)	
Neuropathy	20	Self-reported physical activity, n(%)	regularly - 1-2 times/week	20 (21.5%)	33.6	11 (22.4%)	
Neuropathy	20	Self-reported physical activity, n(%)	regularly - 3-5 times/week	27 (29%)	33.6	14 (28.6%)	
Neuropathy	20	Self-reported physical activity, n(%)	Daily	18 (19.4%)	33.6	7 (14.3%)	
Neuropathy	20	Sex, n(%)	Males	67 (47.9%)	0	25 (51%)	n=140 vs n=49
Neuropathy	20	Sex, n(%)	Females	73 (52.1%)	0	24 (49%)	
Neuropathy	20	Smoking status, n(%)	No	70 (72.2%)	30.7	33 (67.3%)	
Neuropathy	20	Smoking status, n(%)	Yes	27 (27.8%)	30.7	16 (32.7%)	
Neuropathy	20	Stroke, n(%)	No	114 (100%)	18.6	49 (100%)	
Neuropathy	20	Systolic blood pressure, mean(SD)		124 (11.6)	21.4	125.6 (12.9)	
Neuropathy	20	Time since last healthcare visit, mean(SD)		0.4 (0.4)	0	0.3 (0.3)	
Neuropathy	20	Total cholesterol, mean(SD)		4.7 (1)	37.1	4.8 (1.1)	
Neuropathy	21	Age at diagnosis of type 2 diabetes, mean(SD)		19.6 (0.8)	0	19.5 (0.8)	
Neuropathy	21	BMI, mean(SD)		35.8 (7.7)	20.4	35.5 (7.5)	
Neuropathy	21	HbA1c, mean(SD)		54.7 (19.9)	12.3	53.7 (20.1)	
Neuropathy	21	HDL-cholesterol, mean(SD)		1 (0.3)	31.9	1 (0.3)	
Neuropathy	21	Ischemic heart disease, n(%)	No	189 (99%)	18.7	106 (100%)	

Neuropathy	21	Ischemic heart disease, n(%)	Yes	2 (1%)	18.7	0 (0%)	
Neuropathy	21	Self-reported physical activity, n(%)	Never	31 (18.7%)	29.4	20 (18.9%)	
Neuropathy	21	Self-reported physical activity, n(%)	<1 times/week	32 (19.3%)	29.4	20 (18.9%)	
Neuropathy	21	Self-reported physical activity, n(%)	regularly - 1-2 times/week	31 (18.7%)	29.4	21 (19.8%)	
Neuropathy	21	Self-reported physical activity, n(%)	regularly - 3-5 times/week	33 (19.9%)	29.4	22 (20.8%)	
Neuropathy	21	Self-reported physical activity, n(%)	Daily	39 (23.5%)	29.4	23 (21.7%)	
Neuropathy	21	Sex, n(%)	Males	118 (50.2%)	0	57 (53.8%)	n=235 vs n=106
Neuropathy	21	Sex, n(%)	Females	117 (49.8%)	0	49 (46.2%)	
Neuropathy	21	Smoking status, n(%)	No	128 (74.4%)	26.8	76 (71.7%)	
Neuropathy	21	Smoking status, n(%)	Yes	44 (25.6%)	26.8	30 (28.3%)	
Neuropathy	21	Stroke, n(%)	No	189 (100%)	19.6	106 (100%)	
Neuropathy	21	Systolic blood pressure, mean(SD)		124.2 (13)	15.7	123.7 (13)	
Neuropathy	21	Time since last healthcare visit, mean(SD)		0.6 (0.6)	0	0.5 (0.5)	
Neuropathy	21	Total cholesterol, mean(SD)		4.8 (1)	28.1	4.8 (1)	
Neuropathy	22	Age at diagnosis of type 2 diabetes, mean(SD)		20.3 (1.1)	0	20.1 (1)	
Neuropathy	22	BMI, mean(SD)		35.5 (7.3)	19.1	35.5 (6.9)	
Neuropathy	22	HbA1c, mean(SD)		55.8 (22)	10.7	55.7 (21.5)	
Neuropathy	22	HDL-cholesterol, mean(SD)		1 (0.4)	27.8	1 (0.4)	
Neuropathy	22	Ischemic heart disease, n(%)	No	290 (99%)	17.7	180 (99.4%)	
Neuropathy	22	Ischemic heart disease, n(%)	Yes	3 (1%)	17.7	1 (0.6%)	
Neuropathy	22	Self-reported physical activity, n(%)	Never	46 (18.1%)	28.7	39 (21.5%)	
Neuropathy	22	Self-reported physical activity, n(%)	<1 times/week	45 (17.7%)	28.7	30 (16.6%)	
Neuropathy	22	Self-reported physical activity, n(%)	regularly - 1-2 times/week	44 (17.3%)	28.7	28 (15.5%)	

Neuropathy	22	Self-reported physical activity, n(%)	regularly - 3-5 times/week	52 (20.5%)	28.7	37 (20.4%)	
Neuropathy	22	Self-reported physical activity, n(%)	Daily	67 (26.4%)	28.7	47 (26%)	
Neuropathy	22	Sex, n(%)	Males	164 (46.1%)	0	83 (45.9%)	n=356 vs n=181
Neuropathy	22	Sex, n(%)	Females	192 (53.9%)	0	98 (54.1%)	
Neuropathy	22	Smoking status, n(%)	No	199 (75.4%)	25.8	140 (77.3%)	
Neuropathy	22	Smoking status, n(%)	Yes	65 (24.6%)	25.8	41 (22.7%)	
Neuropathy	22	Stroke, n(%)	No	288 (99.7%)	18.8	181 (100%)	
Neuropathy	22	Stroke, n(%)	Yes	1 (0.3%)	18.8	0 (0%)	
Neuropathy	22	Systolic blood pressure, mean(SD)		124.3 (13.8)	15.4	125.2 (13.1)	
Neuropathy	22	Time since last healthcare visit, mean(SD)		0.7 (0.8)	0	0.5 (0.6)	
Neuropathy	22	Total cholesterol, mean(SD)		4.9 (1.1)	25	4.9 (1.1)	
Neuropathy	23	Age at diagnosis of type 2 diabetes, mean(SD)		21 (1.3)	0	20.7 (1.3)	
Neuropathy	23	BMI, mean(SD)		35.6 (7.6)	19.2	35.7 (7)	
Neuropathy	23	HbA1c, mean(SD)		55.1 (19.4)	9.1	55.5 (20)	
Neuropathy	23	HDL-cholesterol, mean(SD)		1 (0.4)	28.2	1 (0.4)	
Neuropathy	23	Ischemic heart disease, n(%)	No	404 (99.3%)	19.2	265 (100%)	
Neuropathy	23	Ischemic heart disease, n(%)	Yes	3 (0.7%)	19.2	0 (0%)	
Neuropathy	23	Self-reported physical activity, n(%)	Never	67 (18.2%)	27	50 (18.9%)	
Neuropathy	23	Self-reported physical activity, n(%)	<1 times/week	59 (16%)	27	44 (16.6%)	
Neuropathy	23	Self-reported physical activity, n(%)	regularly - 1-2 times/week	68 (18.5%)	27	50 (18.9%)	
Neuropathy	23	Self-reported physical activity, n(%)	regularly - 3-5 times/week	86 (23.4%)	27	63 (23.8%)	
Neuropathy	23	Self-reported physical activity, n(%)	Daily	88 (23.9%)	27	58 (21.9%)	
Neuropathy	23	Sex, n(%)	Males	238 (47.2%)	0	129 (48.7%)	n=504 vs n=265

Neuropathy	23	Sex, n(%)	Females	266 (52.8%)	0	136 (51.3%)	
Neuropathy	23	Smoking status, n(%)	No	281 (73%)	23.6	200 (75.5%)	
Neuropathy	23	Smoking status, n(%)	Yes	104 (27%)	23.6	65 (24.5%)	
Neuropathy	23	Stroke, n(%)	No	402 (99.8%)	20	265 (100%)	
Neuropathy	23	Stroke, n(%)	Yes	1 (0.2%)	20	0 (0%)	
Neuropathy	23	Systolic blood pressure, mean(SD)		124.4 (13.6)	15.1	124.6 (13.7)	
Neuropathy	23	Time since last healthcare visit, mean(SD)		0.8 (1)	0	0.6 (0.8)	
Neuropathy	23	Total cholesterol, mean(SD)		4.8 (1.1)	24.4	4.8 (1.1)	
Neuropathy	24	Age at diagnosis of type 2 diabetes, mean(SD)		21.7 (1.6)	0	21.4 (1.6)	
Neuropathy	24	BMI, mean(SD)		35.7 (7.5)	17.1	35.9 (7.1)	
Neuropathy	24	HbA1c, mean(SD)		55.1 (20.8)	8.7	54.1 (19.2)	
Neuropathy	24	HDL-cholesterol, mean(SD)		1 (0.4)	28.1	1 (0.4)	
Neuropathy	24	Ischemic heart disease, n(%)	No	583 (99.1%)	16	372 (100%)	
Neuropathy	24	Ischemic heart disease, n(%)	Yes	5 (0.9%)	16	0 (0%)	
Neuropathy	24	Self-reported physical activity, n(%)	Never	73 (14%)	25.3	48 (12.9%)	
Neuropathy	24	Self-reported physical activity, n(%)	<1 times/week	88 (16.8%)	25.3	64 (17.2%)	
Neuropathy	24	Self-reported physical activity, n(%)	regularly - 1-2 times/week	109 (20.8%)	25.3	80 (21.5%)	
Neuropathy	24	Self-reported physical activity, n(%)	regularly - 3-5 times/week	127 (24.3%)	25.3	94 (25.3%)	
Neuropathy	24	Self-reported physical activity, n(%)	Daily	126 (24.1%)	25.3	86 (23.1%)	
Neuropathy	24	Sex, n(%)	Males	324 (46.3%)	0	172 (46.2%)	n=700 vs n=372
Neuropathy	24	Sex, n(%)	Females	376 (53.7%)	0	200 (53.8%)	
Neuropathy	24	Smoking status, n(%)	No	410 (73%)	19.7	277 (74.5%)	
Neuropathy	24	Smoking status, n(%)	Yes	152 (27%)	19.7	95 (25.5%)	

Neuropathy	24	Stroke, n(%)	No	580 (99.5%)	16.7	370 (99.5%)	
Neuropathy	24	Stroke, n(%)	Yes	3 (0.5%)	16.7	2 (0.5%)	
Neuropathy	24	Systolic blood pressure, mean(SD)		124.3 (13.7)	13	124.4 (13.6)	
Neuropathy	24	Time since last healthcare visit, mean(SD)		0.9 (1.1)	0	0.7 (0.9)	
Neuropathy	24	Total cholesterol, mean(SD)		4.9 (1.3)	25.4	4.8 (1.2)	
Neuropathy	25	Age at diagnosis of type 2 diabetes, mean(SD)		22.4 (1.8)	0	22.1 (1.7)	
Neuropathy	25	BMI, mean(SD)		35.6 (7.8)	15.8	35.3 (7.4)	
Neuropathy	25	HbA1c, mean(SD)		53.7 (20.2)	7.7	53.1 (19.5)	
Neuropathy	25	HDL-cholesterol, mean(SD)		1 (0.4)	26.7	1 (0.4)	
Neuropathy	25	Ischemic heart disease, n(%)	No	736 (99.3%)	15	467 (100%)	
Neuropathy	25	Ischemic heart disease, n(%)	Yes	5 (0.7%)	15	0 (0%)	
Neuropathy	25	Self-reported physical activity, n(%)	Never	102 (15.3%)	23.7	67 (14.3%)	
Neuropathy	25	Self-reported physical activity, n(%)	<1 times/week	102 (15.3%)	23.7	76 (16.3%)	
Neuropathy	25	Self-reported physical activity, n(%)	regularly - 1-2 times/week	135 (20.3%)	23.7	92 (19.7%)	
Neuropathy	25	Self-reported physical activity, n(%)	regularly - 3-5 times/week	160 (24.1%)	23.7	121 (25.9%)	
Neuropathy	25	Self-reported physical activity, n(%)	Daily	166 (25%)	23.7	111 (23.8%)	
Neuropathy	25	Sex, n(%)	Males	407 (46.7%)	0	224 (48%)	n=872 vs n=467
Neuropathy	25	Sex, n(%)	Females	465 (53.3%)	0	243 (52%)	
Neuropathy	25	Smoking status, n(%)	No	528 (74.6%)	18.8	355 (76%)	
Neuropathy	25	Smoking status, n(%)	Yes	180 (25.4%)	18.8	112 (24%)	
Neuropathy	25	Stroke, n(%)	No	729 (99.6%)	16.1	466 (99.8%)	
Neuropathy	25	Stroke, n(%)	Yes	3 (0.4%)	16.1	1 (0.2%)	
Neuropathy	25	Systolic blood pressure, mean(SD)		123.8 (13.5)	10.8	123.4 (13.1)	

Neuropathy	25	Time since last healthcare visit, mean(SD)		1 (1.3)	0	0.7 (1)	
Neuropathy	25	Total cholesterol, mean(SD)		4.8 (1.2)	23.7	4.8 (1.1)	
Neuropathy	26	Age at diagnosis of type 2 diabetes, mean(SD)		23.1 (2)	0	22.9 (1.9)	
Neuropathy	26	BMI, mean(SD)		35.3 (7.7)	14.5	35.1 (7.2)	
Neuropathy	26	HbA1c, mean(SD)		53.4 (19.1)	7.9	52.8 (19.3)	
Neuropathy	26	HDL-cholesterol, mean(SD)		1 (0.3)	26.2	1 (0.3)	
Neuropathy	26	Ischemic heart disease, n(%)	No	888 (99.4%)	14.7	578 (99.8%)	
Neuropathy	26	Ischemic heart disease, n(%)	Yes	5 (0.6%)	14.7	1 (0.2%)	
Neuropathy	26	Self-reported physical activity, n(%)	Never	112 (13.8%)	22.6	77 (13.3%)	
Neuropathy	26	Self-reported physical activity, n(%)	<1 times/week	108 (13.3%)	22.6	76 (13.1%)	
Neuropathy	26	Self-reported physical activity, n(%)	regularly - 1-2 times/week	159 (19.6%)	22.6	116 (20%)	
Neuropathy	26	Self-reported physical activity, n(%)	regularly - 3-5 times/week	224 (27.7%)	22.6	166 (28.7%)	
Neuropathy	26	Self-reported physical activity, n(%)	Daily	207 (25.6%)	22.6	144 (24.9%)	
Neuropathy	26	Sex, n(%)	Males	527 (50.3%)	0	289 (49.9%)	n=1047 vs n=579
Neuropathy	26	Sex, n(%)	Females	520 (49.7%)	0	290 (50.1%)	
Neuropathy	26	Smoking status, n(%)	No	660 (76.2%)	17.3	441 (76.2%)	
Neuropathy	26	Smoking status, n(%)	Yes	206 (23.8%)	17.3	138 (23.8%)	
Neuropathy	26	Stroke, n(%)	No	877 (99.5%)	15.9	577 (99.7%)	
Neuropathy	26	Stroke, n(%)	Yes	4 (0.5%)	15.9	2 (0.3%)	
Neuropathy	26	Systolic blood pressure, mean(SD)		124.3 (13.3)	10.4	124.3 (13.2)	
Neuropathy	26	Time since last healthcare visit, mean(SD)		1 (1.4)	0	0.7 (1)	
Neuropathy	26	Total cholesterol, mean(SD)		4.8 (1.2)	23.2	4.8 (1.2)	
Neuropathy	27	Age at diagnosis of type 2 diabetes, mean(SD)		24 (2.1)	0	23.7 (2.1)	

Neuropathy	27	BMI, mean(SD)		35 (7.5)	15.6	35.1 (7.3)	
Neuropathy	27	HbA1c, mean(SD)		52.9 (18.8)	7.4	53.4 (19)	
Neuropathy	27	HDL-cholesterol, mean(SD)		1 (0.4)	26.4	1 (0.4)	
Neuropathy	27	Ischemic heart disease, n(%)	No	1056 (99.3%)	14.1	684 (99.7%)	
Neuropathy	27	Ischemic heart disease, n(%)	Yes	7 (0.7%)	14.1	2 (0.3%)	
Neuropathy	27	Self-reported physical activity, n(%)	Never	122 (13%)	23.9	87 (12.7%)	
Neuropathy	27	Self-reported physical activity, n(%)	<1 times/week	130 (13.8%)	23.9	92 (13.4%)	
Neuropathy	27	Self-reported physical activity, n(%)	regularly - 1-2 times/week	183 (19.4%)	23.9	132 (19.2%)	
Neuropathy	27	Self-reported physical activity, n(%)	regularly - 3-5 times/week	268 (28.5%)	23.9	213 (31%)	
Neuropathy	27	Self-reported physical activity, n(%)	Daily	238 (25.3%)	23.9	162 (23.6%)	
Neuropathy	27	Sex, n(%)	Males	624 (50.4%)	0	361 (52.6%)	n=1237 vs n=686
Neuropathy	27	Sex, n(%)	Females	613 (49.6%)	0	325 (47.4%)	
Neuropathy	27	Smoking status, n(%)	No	765 (75.4%)	18	518 (75.5%)	
Neuropathy	27	Smoking status, n(%)	Yes	249 (24.6%)	18	168 (24.5%)	
Neuropathy	27	Stroke, n(%)	No	1048 (99.2%)	14.6	683 (99.6%)	
Neuropathy	27	Stroke, n(%)	Yes	8 (0.8%)	14.6	3 (0.4%)	
Neuropathy	27	Systolic blood pressure, mean(SD)		124.5 (13.8)	10.3	124.5 (14)	
Neuropathy	27	Time since last healthcare visit, mean(SD)		1 (1.5)	0	0.7 (1.1)	
Neuropathy	27	Total cholesterol, mean(SD)		4.8 (1.2)	22.2	4.8 (1.2)	
Neuropathy	28	Age at diagnosis of type 2 diabetes, mean(SD)		24.8 (2.3)	0	24.4 (2.2)	
Neuropathy	28	BMI, mean(SD)		35.1 (7.4)	15.4	35.1 (7.3)	
Neuropathy	28	HbA1c, mean(SD)		52.7 (18.2)	7.7	53.5 (17.9)	
Neuropathy	28	HDL-cholesterol, mean(SD)		1 (0.3)	26.7	1 (0.3)	

Neuropathy	28	Ischemic heart disease, n(%)	No	1228 (99%)	14.4	797 (99%)	
Neuropathy	28	Ischemic heart disease, n(%)	Yes	13 (1%)	14.4	8 (1%)	
Neuropathy	28	Self-reported physical activity, n(%)	Never	144 (13%)	23.4	104 (12.9%)	
Neuropathy	28	Self-reported physical activity, n(%)	<1 times/week	119 (10.7%)	23.4	82 (10.2%)	
Neuropathy	28	Self-reported physical activity, n(%)	regularly - 1-2 times/week	240 (21.6%)	23.4	170 (21.1%)	
Neuropathy	28	Self-reported physical activity, n(%)	regularly - 3-5 times/week	305 (27.5%)	23.4	230 (28.6%)	
Neuropathy	28	Self-reported physical activity, n(%)	Daily	302 (27.2%)	23.4	219 (27.2%)	
Neuropathy	28	Sex, n(%)	Males	719 (49.6%)	0	419 (52%)	n=1450 vs n=805
Neuropathy	28	Sex, n(%)	Females	731 (50.4%)	0	386 (48%)	
Neuropathy	28	Smoking status, n(%)	No	903 (75.8%)	17.8	617 (76.6%)	
Neuropathy	28	Smoking status, n(%)	Yes	289 (24.2%)	17.8	188 (23.4%)	
Neuropathy	28	Stroke, n(%)	No	1227 (99.1%)	14.6	798 (99.1%)	
Neuropathy	28	Stroke, n(%)	Yes	11 (0.9%)	14.6	7 (0.9%)	
Neuropathy	28	Systolic blood pressure, mean(SD)		124.7 (13.3)	10.3	125.3 (13.5)	
Neuropathy	28	Time since last healthcare visit, mean(SD)		1 (1.5)	0	0.8 (1.1)	
Neuropathy	28	Total cholesterol, mean(SD)		4.8 (1.1)	22.1	4.8 (1.1)	
Neuropathy	29	Age at diagnosis of type 2 diabetes, mean(SD)		25.7 (2.4)	0	25.3 (2.4)	
Neuropathy	29	BMI, mean(SD)		34.8 (7.5)	14.9	34.7 (7.1)	
Neuropathy	29	HbA1c, mean(SD)		53.5 (18.7)	7.3	53.7 (18)	
Neuropathy	29	HDL-cholesterol, mean(SD)		1 (0.3)	26.5	1 (0.3)	
Neuropathy	29	Ischemic heart disease, n(%)	No	1407 (99.2%)	13.9	920 (99.4%)	
Neuropathy	29	Ischemic heart disease, n(%)	Yes	11 (0.8%)	13.9	6 (0.6%)	
Neuropathy	29	Self-reported physical activity, n(%)	Never	163 (12.9%)	23.1	117 (12.6%)	

Neuropathy	29	Self-reported physical activity, n(%)	<1 times/week	154 (12.2%)	23.1	112 (12.1%)	
Neuropathy	29	Self-reported physical activity, n(%)	regularly - 1-2 times/week	266 (21%)	23.1	188 (20.3%)	
Neuropathy	29	Self-reported physical activity, n(%)	regularly - 3-5 times/week	336 (26.5%)	23.1	263 (28.4%)	
Neuropathy	29	Self-reported physical activity, n(%)	Daily	347 (27.4%)	23.1	246 (26.6%)	
Neuropathy	29	Sex, n(%)	Males	842 (51.1%)	0	483 (52.2%)	n=1647 vs n=926
Neuropathy	29	Sex, n(%)	Females	805 (48.9%)	0	443 (47.8%)	
Neuropathy	29	Smoking status, n(%)	No	1059 (77.6%)	17.2	712 (76.9%)	
Neuropathy	29	Smoking status, n(%)	Yes	305 (22.4%)	17.2	214 (23.1%)	
Neuropathy	29	Stroke, n(%)	No	1400 (99.2%)	14.3	920 (99.4%)	
Neuropathy	29	Stroke, n(%)	Yes	11 (0.8%)	14.3	6 (0.6%)	
Neuropathy	29	Systolic blood pressure, mean(SD)		124.4 (13.6)	9.8	124.6 (13.4)	
Neuropathy	29	Time since last healthcare visit, mean(SD)		1.1 (1.6)	0	0.8 (1.1)	
Neuropathy	29	Total cholesterol, mean(SD)		4.8 (1.1)	21.9	4.8 (1.1)	
Neuropathy	30	Age at diagnosis of type 2 diabetes, mean(SD)		26.7 (2.5)	0	26.2 (2.6)	
Neuropathy	30	BMI, mean(SD)		34.6 (7.6)	14.5	34.6 (7.4)	
Neuropathy	30	HbA1c, mean(SD)		53.5 (19.1)	7	53.8 (18.6)	
Neuropathy	30	HDL-cholesterol, mean(SD)		1 (0.3)	26.3	1 (0.3)	
Neuropathy	30	Ischemic heart disease, n(%)	No	1606 (99.3%)	13.8	1056 (99.3%)	
Neuropathy	30	Ischemic heart disease, n(%)	Yes	12 (0.7%)	13.8	7 (0.7%)	
Neuropathy	30	Self-reported physical activity, n(%)	Never	173 (11.8%)	21.8	133 (12.5%)	
Neuropathy	30	Self-reported physical activity, n(%)	<1 times/week	200 (13.6%)	21.8	134 (12.6%)	
Neuropathy	30	Self-reported physical activity, n(%)	regularly - 1-2 times/week	324 (22.1%)	21.8	233 (21.9%)	
Neuropathy	30	Self-reported physical activity, n(%)	regularly - 3-5 times/week	381 (26%)	21.8	275 (25.9%)	

Neuropathy	30	Self-reported physical activity, n(%)	Daily	389 (26.5%)	21.8	288 (27.1%)	
Neuropathy	30	Sex, n(%)	Males	982 (52.3%)	0	579 (54.5%)	n=1876 vs n=1063
Neuropathy	30	Sex, n(%)	Females	894 (47.7%)	0	484 (45.5%)	
Neuropathy	30	Smoking status, n(%)	No	1217 (77.4%)	16.2	838 (78.8%)	
Neuropathy	30	Smoking status, n(%)	Yes	355 (22.6%)	16.2	225 (21.2%)	
Neuropathy	30	Stroke, n(%)	No	1601 (99.3%)	14.1	1056 (99.3%)	
Neuropathy	30	Stroke, n(%)	Yes	11 (0.7%)	14.1	7 (0.7%)	
Neuropathy	30	Systolic blood pressure, mean(SD)		124.6 (13.7)	8.9	125.1 (13.7)	
Neuropathy	30	Time since last healthcare visit, mean(SD)		1.1 (1.6)	0	0.7 (1.1)	
Neuropathy	30	Total cholesterol, mean(SD)		4.8 (1.2)	22.2	4.8 (1.1)	
Neuropathy	31	Age at diagnosis of type 2 diabetes, mean(SD)		27.6 (2.6)	0	27.1 (2.7)	
Neuropathy	31	BMI, mean(SD)		34.3 (7.4)	13.6	34.2 (7)	
Neuropathy	31	HbA1c, mean(SD)		53.2 (18.5)	6.6	53.4 (17.9)	
Neuropathy	31	HDL-cholesterol, mean(SD)		1 (0.3)	25.6	1 (0.3)	
Neuropathy	31	Ischemic heart disease, n(%)	No	1852 (99.3%)	12.7	1212 (99.3%)	
Neuropathy	31	Ischemic heart disease, n(%)	Yes	13 (0.7%)	12.7	9 (0.7%)	
Neuropathy	31	Self-reported physical activity, n(%)	Never	197 (11.8%)	21.8	148 (12.1%)	
Neuropathy	31	Self-reported physical activity, n(%)	<1 times/week	224 (13.4%)	21.8	154 (12.6%)	
Neuropathy	31	Self-reported physical activity, n(%)	regularly - 1-2 times/week	357 (21.4%)	21.8	259 (21.2%)	
Neuropathy	31	Self-reported physical activity, n(%)	regularly - 3-5 times/week	443 (26.5%)	21.8	330 (27%)	
Neuropathy	31	Self-reported physical activity, n(%)	Daily	450 (26.9%)	21.8	330 (27%)	
Neuropathy	31	Sex, n(%)	Males	1159 (54.3%)	0	694 (56.8%)	n=2136 vs n=1221
Neuropathy	31	Sex, n(%)	Females	977 (45.7%)	0	527 (43.2%)	

Neuropathy	31	Smoking status, n(%)	No	1401 (77.2%)	15	949 (77.7%)	
Neuropathy	31	Smoking status, n(%)	Yes	414 (22.8%)	15	272 (22.3%)	
Neuropathy	31	Stroke, n(%)	No	1848 (99.3%)	12.9	1210 (99.1%)	
Neuropathy	31	Stroke, n(%)	Yes	13 (0.7%)	12.9	11 (0.9%)	
Neuropathy	31	Systolic blood pressure, mean(SD)		124.7 (13.7)	9	125 (13.5)	
Neuropathy	31	Time since last healthcare visit, mean(SD)		1 (1.5)	0	0.7 (1)	
Neuropathy	31	Total cholesterol, mean(SD)		4.8 (1.2)	20.9	4.8 (1.2)	
Neuropathy	32	Age at diagnosis of type 2 diabetes, mean(SD)		28.7 (2.6)	0	28.2 (2.7)	
Neuropathy	32	BMI, mean(SD)		34.1 (7.5)	13.4	33.9 (7.3)	
Neuropathy	32	HbA1c, mean(SD)		52.8 (18.6)	6.1	53.2 (18.1)	
Neuropathy	32	HDL-cholesterol, mean(SD)		1 (0.3)	24.4	1 (0.3)	
Neuropathy	32	Ischemic heart disease, n(%)	No	2135 (99.3%)	12.6	1423 (99.4%)	
Neuropathy	32	Ischemic heart disease, n(%)	Yes	15 (0.7%)	12.6	9 (0.6%)	
Neuropathy	32	Self-reported physical activity, n(%)	Never	259 (13.3%)	20.7	196 (13.7%)	
Neuropathy	32	Self-reported physical activity, n(%)	<1 times/week	256 (13.1%)	20.7	181 (12.6%)	
Neuropathy	32	Self-reported physical activity, n(%)	regularly - 1-2 times/week	409 (21%)	20.7	287 (20%)	
Neuropathy	32	Self-reported physical activity, n(%)	regularly - 3-5 times/week	518 (26.6%)	20.7	408 (28.5%)	
Neuropathy	32	Self-reported physical activity, n(%)	Daily	508 (26.1%)	20.7	360 (25.1%)	
Neuropathy	32	Sex, n(%)	Males	1340 (54.5%)	0	823 (57.5%)	n=2459 vs n=1432
Neuropathy	32	Sex, n(%)	Females	1119 (45.5%)	0	609 (42.5%)	
Neuropathy	32	Smoking status, n(%)	No	1638 (77.7%)	14.2	1129 (78.8%)	
Neuropathy	32	Smoking status, n(%)	Yes	471 (22.3%)	14.2	303 (21.2%)	
Neuropathy	32	Stroke, n(%)	No	2135 (99.4%)	12.6	1421 (99.2%)	

Neuropathy	32	Stroke, n(%)	Yes	13 (0.6%)	12.6	11 (0.8%)	
Neuropathy	32	Systolic blood pressure, mean(SD)		125 (14.2)	7.8	125.3 (14)	
Neuropathy	32	Time since last healthcare visit, mean(SD)		1 (1.5)	0	0.7 (1.1)	
Neuropathy	32	Total cholesterol, mean(SD)		4.8 (1.2)	19.6	4.8 (1.2)	
Neuropathy	33	Age at diagnosis of type 2 diabetes, mean(SD)		29.7 (2.6)	0	29.2 (2.6)	
Neuropathy	33	BMI, mean(SD)		34 (7.5)	13.3	34 (7.4)	
Neuropathy	33	HbA1c, mean(SD)		52.7 (17.7)	5.4	52.7 (16.9)	
Neuropathy	33	HDL-cholesterol, mean(SD)		1 (0.3)	23.4	1.1 (0.3)	
Neuropathy	33	Ischemic heart disease, n(%)	No	2466 (99.2%)	12.8	1644 (99.4%)	
Neuropathy	33	Ischemic heart disease, n(%)	Yes	21 (0.8%)	12.8	10 (0.6%)	
Neuropathy	33	Self-reported physical activity, n(%)	Never	304 (13.4%)	20.5	237 (14.3%)	
Neuropathy	33	Self-reported physical activity, n(%)	<1 times/week	305 (13.4%)	20.5	215 (13%)	
Neuropathy	33	Self-reported physical activity, n(%)	regularly - 1-2 times/week	475 (20.9%)	20.5	331 (20%)	
Neuropathy	33	Self-reported physical activity, n(%)	regularly - 3-5 times/week	566 (24.9%)	20.5	413 (25%)	
Neuropathy	33	Self-reported physical activity, n(%)	Daily	619 (27.3%)	20.5	458 (27.7%)	
Neuropathy	33	Sex, n(%)	Males	1583 (55.5%)	0	936 (56.6%)	n=2853 vs n=1654
Neuropathy	33	Sex, n(%)	Females	1270 (44.5%)	0	718 (43.4%)	
Neuropathy	33	Smoking status, n(%)	No	1892 (76.8%)	13.6	1285 (77.7%)	
Neuropathy	33	Smoking status, n(%)	Yes	572 (23.2%)	13.6	369 (22.3%)	
Neuropathy	33	Stroke, n(%)	No	2469 (99.5%)	13	1644 (99.4%)	
Neuropathy	33	Stroke, n(%)	Yes	13 (0.5%)	13	10 (0.6%)	
Neuropathy	33	Systolic blood pressure, mean(SD)		125 (14.2)	7.5	125.1 (13.9)	
Neuropathy	33	Time since last healthcare visit, mean(SD)		0.9 (1.4)	0	0.7 (1)	

Neuropathy	33	Total cholesterol, mean(SD)		4.9 (1.2)	18.8	4.8 (1.2)	
Neuropathy	34	Age at diagnosis of type 2 diabetes, mean(SD)		30.7 (2.6)	0	30.2 (2.6)	
Neuropathy	34	BMI, mean(SD)		33.9 (7.4)	12.1	33.7 (7.3)	
Neuropathy	34	HbA1c, mean(SD)		52.4 (17.2)	4.6	52.4 (16.4)	
Neuropathy	34	HDL-cholesterol, mean(SD)		1.1 (0.3)	22.9	1.1 (0.3)	
Neuropathy	34	Ischemic heart disease, n(%)	No	2817 (98.9%)	12.2	1888 (99%)	
Neuropathy	34	Ischemic heart disease, n(%)	Yes	31 (1.1%)	12.2	19 (1%)	
Neuropathy	34	Self-reported physical activity, n(%)	Never	341 (13.1%)	19.9	244 (12.8%)	
Neuropathy	34	Self-reported physical activity, n(%)	<1 times/week	331 (12.7%)	19.9	242 (12.7%)	
Neuropathy	34	Self-reported physical activity, n(%)	regularly - 1-2 times/week	565 (21.8%)	19.9	407 (21.3%)	
Neuropathy	34	Self-reported physical activity, n(%)	regularly - 3-5 times/week	662 (25.5%)	19.9	490 (25.7%)	
Neuropathy	34	Self-reported physical activity, n(%)	Daily	698 (26.9%)	19.9	524 (27.5%)	
Neuropathy	34	Sex, n(%)	Males	1806 (55.7%)	0	1098 (57.6%)	n=3242 vs n=1907
Neuropathy	34	Sex, n(%)	Females	1436 (44.3%)	0	809 (42.4%)	
Neuropathy	34	Smoking status, n(%)	No	2199 (78.5%)	13.6	1520 (79.7%)	
Neuropathy	34	Smoking status, n(%)	Yes	602 (21.5%)	13.6	387 (20.3%)	
Neuropathy	34	Stroke, n(%)	No	2828 (99.5%)	12.3	1896 (99.4%)	
Neuropathy	34	Stroke, n(%)	Yes	15 (0.5%)	12.3	11 (0.6%)	
Neuropathy	34	Systolic blood pressure, mean(SD)		125.4 (14.3)	7.6	125.3 (13.9)	
Neuropathy	34	Time since last healthcare visit, mean(SD)		0.9 (1.4)	0	0.7 (1)	
Neuropathy	34	Total cholesterol, mean(SD)		4.9 (1.1)	18.2	4.8 (1.1)	
Neuropathy	35	Age at diagnosis of type 2 diabetes, mean(SD)		31.7 (2.5)	0	31.2 (2.5)	
Neuropathy	35	BMI, mean(SD)		33.9 (7.3)	12.3	33.8 (7.3)	

Neuropathy	35	HbA1c, mean(SD)		52.4 (17.4)	4.6	52.4 (16.6)	
Neuropathy	35	HDL-cholesterol, mean(SD)		1.1 (0.3)	21.8	1.1 (0.3)	
Neuropathy	35	Ischemic heart disease, n(%)	No	3181 (99%)	12.5	2156 (98.9%)	
Neuropathy	35	Ischemic heart disease, n(%)	Yes	32 (1%)	12.5	25 (1.1%)	
Neuropathy	35	Self-reported physical activity, n(%)	Never	406 (13.8%)	19.9	291 (13.3%)	
Neuropathy	35	Self-reported physical activity, n(%)	<1 times/week	359 (12.2%)	19.9	257 (11.8%)	
Neuropathy	35	Self-reported physical activity, n(%)	regularly - 1-2 times/week	636 (21.6%)	19.9	481 (22.1%)	
Neuropathy	35	Self-reported physical activity, n(%)	regularly - 3-5 times/week	748 (25.4%)	19.9	555 (25.4%)	
Neuropathy	35	Self-reported physical activity, n(%)	Daily	794 (27%)	19.9	597 (27.4%)	
Neuropathy	35	Sex, n(%)	Males	2068 (56.3%)	0	1272 (58.3%)	n=3674 vs n=2181
Neuropathy	35	Sex, n(%)	Females	1606 (43.7%)	0	909 (41.7%)	
Neuropathy	35	Smoking status, n(%)	No	2530 (79.7%)	13.6	1760 (80.7%)	
Neuropathy	35	Smoking status, n(%)	Yes	643 (20.3%)	13.6	421 (19.3%)	
Neuropathy	35	Stroke, n(%)	No	3198 (99.6%)	12.6	2169 (99.4%)	
Neuropathy	35	Stroke, n(%)	Yes	14 (0.4%)	12.6	12 (0.6%)	
Neuropathy	35	Systolic blood pressure, mean(SD)		125.5 (14.3)	7.3	125.3 (14)	
Neuropathy	35	Time since last healthcare visit, mean(SD)		0.9 (1.4)	0	0.7 (1.1)	
Neuropathy	35	Total cholesterol, mean(SD)		4.9 (1.1)	17.3	4.8 (1.1)	
Neuropathy	36	Age at diagnosis of type 2 diabetes, mean(SD)		32.6 (2.5)	0	32.2 (2.5)	
Neuropathy	36	BMI, mean(SD)		33.7 (7.1)	11.6	33.6 (7.1)	
Neuropathy	36	HbA1c, mean(SD)		52.5 (17.5)	4.2	52.8 (16.8)	
Neuropathy	36	HDL-cholesterol, mean(SD)		1.1 (0.3)	21.4	1.1 (0.3)	
Neuropathy	36	Ischemic heart disease, n(%)	No	3559 (98.8%)	13	2481 (98.8%)	

Neuropathy	36	Ischemic heart disease, n(%)	Yes	43 (1.2%)	13	29 (1.2%)	
Neuropathy	36	Self-reported physical activity, n(%)	Never	494 (14.7%)	18.8	358 (14.3%)	
Neuropathy	36	Self-reported physical activity, n(%)	<1 times/week	422 (12.6%)	18.8	302 (12%)	
Neuropathy	36	Self-reported physical activity, n(%)	regularly - 1-2 times/week	704 (21%)	18.8	549 (21.9%)	
Neuropathy	36	Self-reported physical activity, n(%)	regularly - 3-5 times/week	838 (24.9%)	18.8	619 (24.7%)	
Neuropathy	36	Self-reported physical activity, n(%)	Daily	902 (26.8%)	18.8	682 (27.2%)	
Neuropathy	36	Sex, n(%)	Males	2345 (56.7%)	0	1500 (59.8%)	n=4139 vs n=2510
Neuropathy	36	Sex, n(%)	Females	1794 (43.3%)	0	1010 (40.2%)	
Neuropathy	36	Smoking status, n(%)	No	2876 (79.2%)	12.3	2027 (80.8%)	
Neuropathy	36	Smoking status, n(%)	Yes	754 (20.8%)	12.3	483 (19.2%)	
Neuropathy	36	Stroke, n(%)	No	3579 (99.5%)	13.1	2493 (99.3%)	
Neuropathy	36	Stroke, n(%)	Yes	18 (0.5%)	13.1	17 (0.7%)	
Neuropathy	36	Systolic blood pressure, mean(SD)		125.9 (14.4)	7.5	125.8 (14.1)	
Neuropathy	36	Time since last healthcare visit, mean(SD)		0.9 (1.4)	0	0.7 (1)	
Neuropathy	36	Total cholesterol, mean(SD)		4.9 (1.2)	16.8	4.9 (1.2)	
Neuropathy	37	Age at diagnosis of type 2 diabetes, mean(SD)		33.6 (2.5)	0	33.1 (2.6)	
Neuropathy	37	BMI, mean(SD)		33.6 (7.2)	10.8	33.5 (7.1)	
Neuropathy	37	HbA1c, mean(SD)		52.5 (17.4)	3.9	52.6 (16.7)	
Neuropathy	37	HDL-cholesterol, mean(SD)		1.1 (0.3)	20.7	1.1 (0.3)	
Neuropathy	37	Ischemic heart disease, n(%)	No	4096 (98.6%)	12.1	2852 (98.8%)	
Neuropathy	37	Ischemic heart disease, n(%)	Yes	58 (1.4%)	12.1	34 (1.2%)	
Neuropathy	37	Self-reported physical activity, n(%)	Never	556 (14.5%)	18.7	400 (13.9%)	
Neuropathy	37	Self-reported physical activity, n(%)	<1 times/week	525 (13.7%)	18.7	391 (13.5%)	

Neuropathy	37	Self-reported physical activity, n(%)	regularly - 1-2 times/week	788 (20.5%)	18.7	617 (21.4%)	
Neuropathy	37	Self-reported physical activity, n(%)	regularly - 3-5 times/week	942 (24.5%)	18.7	694 (24%)	
Neuropathy	37	Self-reported physical activity, n(%)	Daily	1033 (26.9%)	18.7	784 (27.2%)	
Neuropathy	37	Sex, n(%)	Males	2685 (56.8%)	0	1723 (59.7%)	n=4727 vs n=2886
Neuropathy	37	Sex, n(%)	Females	2042 (43.2%)	0	1163 (40.3%)	
Neuropathy	37	Smoking status, n(%)	No	3321 (79.6%)	11.8	2343 (81.2%)	
Neuropathy	37	Smoking status, n(%)	Yes	849 (20.4%)	11.8	543 (18.8%)	
Neuropathy	37	Stroke, n(%)	No	4110 (99.2%)	12.4	2861 (99.1%)	
Neuropathy	37	Stroke, n(%)	Yes	33 (0.8%)	12.4	25 (0.9%)	
Neuropathy	37	Systolic blood pressure, mean(SD)		126.1 (14.2)	6.1	125.8 (13.8)	
Neuropathy	37	Time since last healthcare visit, mean(SD)		0.9 (1.4)	0	0.7 (1)	
Neuropathy	37	Total cholesterol, mean(SD)		4.9 (1.2)	16.4	4.8 (1.2)	
Neuropathy	38	Age at diagnosis of type 2 diabetes, mean(SD)		34.6 (2.6)	0	34.1 (2.6)	
Neuropathy	38	BMI, mean(SD)		33.5 (7.1)	11.4	33.3 (6.9)	
Neuropathy	38	HbA1c, mean(SD)		52.2 (17.1)	4.1	52.4 (16.7)	
Neuropathy	38	HDL-cholesterol, mean(SD)		1.1 (0.3)	20	1.1 (0.3)	
Neuropathy	38	Ischemic heart disease, n(%)	No	4699 (98.4%)	11.9	3301 (98.4%)	
Neuropathy	38	Ischemic heart disease, n(%)	Yes	76 (1.6%)	11.9	52 (1.6%)	
Neuropathy	38	Self-reported physical activity, n(%)	Never	629 (14.3%)	18.8	470 (14%)	
Neuropathy	38	Self-reported physical activity, n(%)	<1 times/week	570 (13%)	18.8	436 (13%)	
Neuropathy	38	Self-reported physical activity, n(%)	regularly - 1-2 times/week	923 (21%)	18.8	734 (21.9%)	
Neuropathy	38	Self-reported physical activity, n(%)	regularly - 3-5 times/week	1074 (24.4%)	18.8	793 (23.7%)	
Neuropathy	38	Self-reported physical activity, n(%)	Daily	1201 (27.3%)	18.8	920 (27.4%)	

Neuropathy	38	Sex, n(%)	Males	3099 (57.2%)	0	2005 (59.8%)	n=5418 vs n=3353
Neuropathy	38	Sex, n(%)	Females	2319 (42.8%)	0	1348 (40.2%)	
Neuropathy	38	Smoking status, n(%)	No	3797 (79.2%)	11.5	2702 (80.6%)	
Neuropathy	38	Smoking status, n(%)	Yes	996 (20.8%)	11.5	651 (19.4%)	
Neuropathy	38	Stroke, n(%)	No	4725 (99.2%)	12.1	3328 (99.3%)	
Neuropathy	38	Stroke, n(%)	Yes	40 (0.8%)	12.1	25 (0.7%)	
Neuropathy	38	Systolic blood pressure, mean(SD)		126.4 (14.5)	6.2	126.2 (14)	
Neuropathy	38	Time since last healthcare visit, mean(SD)		0.9 (1.4)	0	0.7 (1)	
Neuropathy	38	Total cholesterol, mean(SD)		4.9 (1.1)	15.4	4.8 (1.1)	
Neuropathy	39	Age at diagnosis of type 2 diabetes, mean(SD)		35.6 (2.6)	0	35.1 (2.7)	
Neuropathy	39	BMI, mean(SD)		33.3 (7)	10.9	33.2 (6.8)	
Neuropathy	39	HbA1c, mean(SD)		52.4 (17.3)	3.8	52.4 (16.9)	
Neuropathy	39	HDL-cholesterol, mean(SD)		1.1 (0.3)	19.9	1.1 (0.3)	
Neuropathy	39	Ischemic heart disease, n(%)	No	5467 (98.5%)	11.9	3878 (98.5%)	
Neuropathy	39	Ischemic heart disease, n(%)	Yes	82 (1.5%)	11.9	61 (1.5%)	
Neuropathy	39	Self-reported physical activity, n(%)	Never	685 (13.2%)	17.7	528 (13.4%)	
Neuropathy	39	Self-reported physical activity, n(%)	<1 times/week	671 (12.9%)	17.7	504 (12.8%)	
Neuropathy	39	Self-reported physical activity, n(%)	regularly - 1-2 times/week	1096 (21.1%)	17.7	824 (20.9%)	
Neuropathy	39	Self-reported physical activity, n(%)	regularly - 3-5 times/week	1319 (25.4%)	17.7	1005 (25.5%)	
Neuropathy	39	Self-reported physical activity, n(%)	Daily	1415 (27.3%)	17.7	1078 (27.4%)	
Neuropathy	39	Sex, n(%)	Males	3625 (57.5%)	0	2348 (59.6%)	n=6301 vs n=3939
Neuropathy	39	Sex, n(%)	Females	2676 (42.5%)	0	1591 (40.4%)	
Neuropathy	39	Smoking status, n(%)	No	4431 (79.3%)	11.3	3183 (80.8%)	

Neuropathy	39	Smoking status, n(%)	Yes	1159 (20.7%)	11.3	756 (19.2%)	
Neuropathy	39	Stroke, n(%)	No	5487 (99.1%)	12.1	3901 (99%)	
Neuropathy	39	Stroke, n(%)	Yes	50 (0.9%)	12.1	38 (1%)	
Neuropathy	39	Systolic blood pressure, mean(SD)		126.7 (14.6)	5.8	126.6 (14.1)	
Neuropathy	39	Time since last healthcare visit, mean(SD)		0.9 (1.3)	0	0.7 (1)	
Neuropathy	39	Total cholesterol, mean(SD)		4.9 (1.1)	15.6	4.8 (1.1)	
Neuropathy	40	Age at diagnosis of type 2 diabetes, mean(SD)		36.6 (2.6)	0	36.1 (2.7)	
Neuropathy	40	BMI, mean(SD)		33.3 (6.8)	10.4	33.2 (6.7)	
Neuropathy	40	HbA1c, mean(SD)		52.4 (16.8)	3.8	52.5 (16.6)	
Neuropathy	40	HDL-cholesterol, mean(SD)		1.1 (0.3)	19.3	1.1 (0.3)	
Neuropathy	40	Ischemic heart disease, n(%)	No	6235 (98.4%)	11.4	4425 (98.4%)	
Neuropathy	40	Ischemic heart disease, n(%)	Yes	99 (1.6%)	11.4	73 (1.6%)	
Neuropathy	40	Self-reported physical activity, n(%)	Never	878 (14.8%)	16.9	641 (14.3%)	
Neuropathy	40	Self-reported physical activity, n(%)	<1 times/week	833 (14%)	16.9	629 (14%)	
Neuropathy	40	Self-reported physical activity, n(%)	regularly - 1-2 times/week	1257 (21.2%)	16.9	975 (21.7%)	
Neuropathy	40	Self-reported physical activity, n(%)	regularly - 3-5 times/week	1433 (24.1%)	16.9	1118 (24.9%)	
Neuropathy	40	Self-reported physical activity, n(%)	Daily	1541 (25.9%)	16.9	1135 (25.2%)	
Neuropathy	40	Sex, n(%)	Males	4177 (58.4%)	0	2704 (60.1%)	n=7151 vs n=4498
Neuropathy	40	Sex, n(%)	Females	2974 (41.6%)	0	1794 (39.9%)	
Neuropathy	40	Smoking status, n(%)	No	5024 (78.8%)	10.9	3614 (80.3%)	
Neuropathy	40	Smoking status, n(%)	Yes	1348 (21.2%)	10.9	884 (19.7%)	
Neuropathy	40	Stroke, n(%)	No	6262 (99.1%)	11.7	4454 (99%)	
Neuropathy	40	Stroke, n(%)	Yes	55 (0.9%)	11.7	44 (1%)	

Neuropathy	40	Systolic blood pressure, mean(SD)		127.2 (14.6)	5.7	127 (14.2)	
Neuropathy	40	Time since last healthcare visit, mean(SD)		0.9 (1.3)	0	0.7 (1)	
Neuropathy	40	Total cholesterol, mean(SD)		4.9 (1.1)	15	4.9 (1.1)	
Neuropathy	41	Age at diagnosis of type 2 diabetes, mean(SD)		37.6 (2.6)	0	37.1 (2.7)	
Neuropathy	41	BMI, mean(SD)		33.3 (6.8)	10.3	33.1 (6.7)	
Neuropathy	41	HbA1c, mean(SD)		52.5 (16.7)	3.4	52.5 (16.1)	
Neuropathy	41	HDL-cholesterol, mean(SD)		1.1 (0.3)	18.4	1.1 (0.3)	
Neuropathy	41	Ischemic heart disease, n(%)	No	7208 (98%)	11.3	5135 (98.1%)	
Neuropathy	41	Ischemic heart disease, n(%)	Yes	147 (2%)	11.3	102 (1.9%)	
Neuropathy	41	Self-reported physical activity, n(%)	Never	1015 (14.7%)	16.6	747 (14.3%)	
Neuropathy	41	Self-reported physical activity, n(%)	<1 times/week	929 (13.4%)	16.6	687 (13.1%)	
Neuropathy	41	Self-reported physical activity, n(%)	regularly - 1-2 times/week	1493 (21.6%)	16.6	1149 (21.9%)	
Neuropathy	41	Self-reported physical activity, n(%)	regularly - 3-5 times/week	1679 (24.3%)	16.6	1311 (25%)	
Neuropathy	41	Self-reported physical activity, n(%)	Daily	1799 (26%)	16.6	1343 (25.6%)	
Neuropathy	41	Sex, n(%)	Males	4895 (59.1%)	0	3187 (60.9%)	n=8289 vs n=5237
Neuropathy	41	Sex, n(%)	Females	3394 (40.9%)	0	2050 (39.1%)	
Neuropathy	41	Smoking status, n(%)	No	5826 (78.5%)	10.5	4192 (80%)	
Neuropathy	41	Smoking status, n(%)	Yes	1592 (21.5%)	10.5	1045 (20%)	
Neuropathy	41	Stroke, n(%)	No	7264 (99.1%)	11.6	5186 (99%)	
Neuropathy	41	Stroke, n(%)	Yes	67 (0.9%)	11.6	51 (1%)	
Neuropathy	41	Systolic blood pressure, mean(SD)		127.7 (14.5)	5.1	127.5 (14.3)	
Neuropathy	41	Time since last healthcare visit, mean(SD)		0.8 (1.3)	0	0.7 (1)	
Neuropathy	41	Total cholesterol, mean(SD)		4.9 (1.1)	14	4.9 (1.1)	

Neuropathy	42	Age at diagnosis of type 2 diabetes, mean(SD)		38.5 (2.6)	0	38 (2.7)	
Neuropathy	42	BMI, mean(SD)		33.2 (6.7)	9.9	33 (6.5)	
Neuropathy	42	HbA1c, mean(SD)		52.3 (16.4)	3.2	52.4 (16)	
Neuropathy	42	HDL-cholesterol, mean(SD)		1.1 (0.3)	18.1	1.1 (0.3)	
Neuropathy	42	Ischemic heart disease, n(%)	No	8169 (97.9%)	11.6	5909 (97.9%)	
Neuropathy	42	Ischemic heart disease, n(%)	Yes	177 (2.1%)	11.6	126 (2.1%)	
Neuropathy	42	Self-reported physical activity, n(%)	Never	1167 (14.8%)	16.4	854 (14.2%)	
Neuropathy	42	Self-reported physical activity, n(%)	<1 times/week	1029 (13%)	16.4	777 (12.9%)	
Neuropathy	42	Self-reported physical activity, n(%)	regularly - 1-2 times/week	1637 (20.8%)	16.4	1281 (21.2%)	
Neuropathy	42	Self-reported physical activity, n(%)	regularly - 3-5 times/week	1951 (24.7%)	16.4	1510 (25%)	
Neuropathy	42	Self-reported physical activity, n(%)	Daily	2102 (26.7%)	16.4	1613 (26.7%)	
Neuropathy	42	Sex, n(%)	Males	5611 (59.5%)	0	3668 (60.8%)	n=9437 vs n=6035
Neuropathy	42	Sex, n(%)	Females	3826 (40.5%)	0	2367 (39.2%)	
Neuropathy	42	Smoking status, n(%)	No	6640 (78.5%)	10.4	4807 (79.7%)	
Neuropathy	42	Smoking status, n(%)	Yes	1816 (21.5%)	10.4	1228 (20.3%)	
Neuropathy	42	Stroke, n(%)	No	8238 (98.9%)	11.7	5968 (98.9%)	
Neuropathy	42	Stroke, n(%)	Yes	95 (1.1%)	11.7	67 (1.1%)	
Neuropathy	42	Systolic blood pressure, mean(SD)		128.1 (14.7)	5.3	127.9 (14.4)	
Neuropathy	42	Time since last healthcare visit, mean(SD)		0.8 (1.3)	0	0.7 (1)	
Neuropathy	42	Total cholesterol, mean(SD)		4.9 (1.2)	13.6	4.8 (1.2)	
Neuropathy	43	Age at diagnosis of type 2 diabetes, mean(SD)		39.4 (2.6)	0	39 (2.7)	
Neuropathy	43	BMI, mean(SD)		33.1 (6.6)	9.5	32.9 (6.5)	
Neuropathy	43	HbA1c, mean(SD)		52.3 (16.1)	3.2	52.4 (15.7)	

Neuropathy	43	HDL-cholesterol, mean(SD)		1.1 (0.3)	18	1.1 (0.3)	
Neuropathy	43	Ischemic heart disease, n(%)	No	9168 (97.5%)	11.2	6675 (97.5%)	
Neuropathy	43	Ischemic heart disease, n(%)	Yes	239 (2.5%)	11.2	169 (2.5%)	
Neuropathy	43	Self-reported physical activity, n(%)	Never	1283 (14.5%)	16.3	968 (14.1%)	
Neuropathy	43	Self-reported physical activity, n(%)	<1 times/week	1199 (13.5%)	16.3	911 (13.3%)	
Neuropathy	43	Self-reported physical activity, n(%)	regularly - 1-2 times/week	1848 (20.9%)	16.3	1442 (21.1%)	
Neuropathy	43	Self-reported physical activity, n(%)	regularly - 3-5 times/week	2152 (24.3%)	16.3	1671 (24.4%)	
Neuropathy	43	Self-reported physical activity, n(%)	Daily	2380 (26.9%)	16.3	1852 (27.1%)	
Neuropathy	43	Sex, n(%)	Males	6395 (60.4%)	0	4233 (61.8%)	n=10588 vs n=6844
Neuropathy	43	Sex, n(%)	Females	4193 (39.6%)	0	2611 (38.2%)	
Neuropathy	43	Smoking status, n(%)	No	7501 (78.6%)	9.8	5443 (79.5%)	
Neuropathy	43	Smoking status, n(%)	Yes	2045 (21.4%)	9.8	1401 (20.5%)	
Neuropathy	43	Stroke, n(%)	No	9262 (98.8%)	11.4	6758 (98.7%)	
Neuropathy	43	Stroke, n(%)	Yes	117 (1.2%)	11.4	86 (1.3%)	
Neuropathy	43	Systolic blood pressure, mean(SD)		128.7 (14.8)	5	128.4 (14.5)	
Neuropathy	43	Time since last healthcare visit, mean(SD)		0.8 (1.3)	0	0.7 (1)	
Neuropathy	43	Total cholesterol, mean(SD)		4.9 (1.2)	13.7	4.9 (1.2)	
Neuropathy	44	Age at diagnosis of type 2 diabetes, mean(SD)		40.5 (2.6)	0	39.9 (2.7)	
Neuropathy	44	BMI, mean(SD)		33 (6.5)	9.5	32.8 (6.4)	
Neuropathy	44	HbA1c, mean(SD)		52.4 (16.2)	3.1	52.4 (15.6)	
Neuropathy	44	HDL-cholesterol, mean(SD)		1.1 (0.3)	18.1	1.1 (0.3)	
Neuropathy	44	Ischemic heart disease, n(%)	No	10310 (96.9%)	11.4	7494 (97%)	
Neuropathy	44	Ischemic heart disease, n(%)	Yes	326 (3.1%)	11.4	232 (3%)	

Neuropathy	44	Self-reported physical activity, n(%)	Never	1443 (14.4%)	16.3	1083 (14%)	
Neuropathy	44	Self-reported physical activity, n(%)	<1 times/week	1381 (13.7%)	16.3	1039 (13.4%)	
Neuropathy	44	Self-reported physical activity, n(%)	regularly - 1-2 times/week	2084 (20.7%)	16.3	1608 (20.8%)	
Neuropathy	44	Self-reported physical activity, n(%)	regularly - 3-5 times/week	2493 (24.8%)	16.3	1938 (25.1%)	
Neuropathy	44	Self-reported physical activity, n(%)	Daily	2647 (26.3%)	16.3	2058 (26.6%)	
Neuropathy	44	Sex, n(%)	Males	7290 (60.7%)	0	4804 (62.2%)	n=12006 vs n=7726
Neuropathy	44	Sex, n(%)	Females	4716 (39.3%)	0	2922 (37.8%)	
Neuropathy	44	Smoking status, n(%)	No	8510 (78.7%)	10	6156 (79.7%)	
Neuropathy	44	Smoking status, n(%)	Yes	2297 (21.3%)	10	1570 (20.3%)	
Neuropathy	44	Stroke, n(%)	No	10468 (98.7%)	11.7	7626 (98.7%)	
Neuropathy	44	Stroke, n(%)	Yes	139 (1.3%)	11.7	100 (1.3%)	
Neuropathy	44	Systolic blood pressure, mean(SD)		128.7 (14.6)	5	128.4 (14.3)	
Neuropathy	44	Time since last healthcare visit, mean(SD)		0.8 (1.3)	0	0.7 (1)	
Neuropathy	44	Total cholesterol, mean(SD)		4.9 (1.2)	13.8	4.9 (1.1)	
Neuropathy	45	Age at diagnosis of type 2 diabetes, mean(SD)		41.5 (2.6)	0	41 (2.7)	
Neuropathy	45	BMI, mean(SD)		32.9 (6.5)	9.8	32.8 (6.4)	
Neuropathy	45	HbA1c, mean(SD)		52.2 (15.7)	3.1	52.1 (15.2)	
Neuropathy	45	HDL-cholesterol, mean(SD)		1.1 (0.3)	17.4	1.1 (0.3)	
Neuropathy	45	Ischemic heart disease, n(%)	No	11640 (96.4%)	11.2	8477 (96.5%)	
Neuropathy	45	Ischemic heart disease, n(%)	Yes	440 (3.6%)	11.2	306 (3.5%)	
Neuropathy	45	Self-reported physical activity, n(%)	Never	1627 (14.3%)	16.2	1225 (13.9%)	
Neuropathy	45	Self-reported physical activity, n(%)	<1 times/week	1518 (13.3%)	16.2	1132 (12.9%)	
Neuropathy	45	Self-reported physical activity, n(%)	regularly - 1-2 times/week	2406 (21.1%)	16.2	1863 (21.2%)	

Neuropathy	45	Self-reported physical activity, n(%)	regularly - 3-5 times/week	2845 (25%)	16.2	2230 (25.4%)	
Neuropathy	45	Self-reported physical activity, n(%)	Daily	3005 (26.4%)	16.2	2333 (26.6%)	
Neuropathy	45	Sex, n(%)	Males	8381 (61.6%)	0	5457 (62.1%)	n=13603 vs n=8783
Neuropathy	45	Sex, n(%)	Females	5222 (38.4%)	0	3326 (37.9%)	
Neuropathy	45	Smoking status, n(%)	No	9619 (78.6%)	10	7001 (79.7%)	
Neuropathy	45	Smoking status, n(%)	Yes	2626 (21.4%)	10	1782 (20.3%)	
Neuropathy	45	Stroke, n(%)	No	11870 (98.6%)	11.5	8669 (98.7%)	
Neuropathy	45	Stroke, n(%)	Yes	166 (1.4%)	11.5	114 (1.3%)	
Neuropathy	45	Systolic blood pressure, mean(SD)		129.3 (14.8)	4.7	129 (14.4)	
Neuropathy	45	Time since last healthcare visit, mean(SD)		0.8 (1.3)	0	0.7 (1)	
Neuropathy	45	Total cholesterol, mean(SD)		4.9 (1.2)	13.2	4.8 (1.1)	
Neuropathy	46	Age at diagnosis of type 2 diabetes, mean(SD)		42.5 (2.6)	0	42 (2.7)	
Neuropathy	46	BMI, mean(SD)		32.9 (6.4)	9.6	32.7 (6.3)	
Neuropathy	46	HbA1c, mean(SD)		52.3 (15.8)	3	52.3 (15.2)	
Neuropathy	46	HDL-cholesterol, mean(SD)		1.1 (0.3)	17	1.1 (0.3)	
Neuropathy	46	Ischemic heart disease, n(%)	No	13047 (95.8%)	11	9543 (95.9%)	
Neuropathy	46	Ischemic heart disease, n(%)	Yes	568 (4.2%)	11	407 (4.1%)	
Neuropathy	46	Self-reported physical activity, n(%)	Never	1832 (14.2%)	15.8	1387 (13.9%)	
Neuropathy	46	Self-reported physical activity, n(%)	<1 times/week	1774 (13.8%)	15.8	1343 (13.5%)	
Neuropathy	46	Self-reported physical activity, n(%)	regularly - 1-2 times/week	2754 (21.4%)	15.8	2146 (21.6%)	
Neuropathy	46	Self-reported physical activity, n(%)	regularly - 3-5 times/week	3194 (24.8%)	15.8	2508 (25.2%)	
Neuropathy	46	Self-reported physical activity, n(%)	Daily	3318 (25.8%)	15.8	2566 (25.8%)	
Neuropathy	46	Sex, n(%)	Males	9527 (62.3%)	0	6268 (63%)	n=15293 vs n=9950

Neuropathy	46	Sex, n(%)	Females	5766 (37.7%)	0	3682 (37%)	
Neuropathy	46	Smoking status, n(%)	No	10833 (78.4%)	9.7	7924 (79.6%)	
Neuropathy	46	Smoking status, n(%)	Yes	2981 (21.6%)	9.7	2026 (20.4%)	
Neuropathy	46	Stroke, n(%)	No	13323 (98.3%)	11.4	9781 (98.3%)	
Neuropathy	46	Stroke, n(%)	Yes	232 (1.7%)	11.4	169 (1.7%)	
Neuropathy	46	Systolic blood pressure, mean(SD)		129.7 (14.7)	4.5	129.4 (14.4)	
Neuropathy	46	Time since last healthcare visit, mean(SD)		0.8 (1.3)	0	0.7 (1)	
Neuropathy	46	Total cholesterol, mean(SD)		4.9 (1.2)	12.4	4.8 (1.1)	
Neuropathy	47	Age at diagnosis of type 2 diabetes, mean(SD)		43.4 (2.6)	0	42.9 (2.7)	
Neuropathy	47	BMI, mean(SD)		32.7 (6.3)	9.4	32.6 (6.2)	
Neuropathy	47	HbA1c, mean(SD)		52.1 (15.4)	2.6	52.1 (14.9)	
Neuropathy	47	HDL-cholesterol, mean(SD)		1.1 (0.3)	16.3	1.1 (0.3)	
Neuropathy	47	Ischemic heart disease, n(%)	No	14576 (95.3%)	10.5	10773 (95.7%)	
Neuropathy	47	Ischemic heart disease, n(%)	Yes	713 (4.7%)	10.5	486 (4.3%)	
Neuropathy	47	Self-reported physical activity, n(%)	Never	2007 (13.9%)	15.5	1548 (13.7%)	
Neuropathy	47	Self-reported physical activity, n(%)	<1 times/week	2009 (13.9%)	15.5	1520 (13.5%)	
Neuropathy	47	Self-reported physical activity, n(%)	regularly - 1-2 times/week	3048 (21.1%)	15.5	2372 (21.1%)	
Neuropathy	47	Self-reported physical activity, n(%)	regularly - 3-5 times/week	3602 (24.9%)	15.5	2855 (25.4%)	
Neuropathy	47	Self-reported physical activity, n(%)	Daily	3774 (26.1%)	15.5	2964 (26.3%)	
Neuropathy	47	Sex, n(%)	Males	10719 (62.7%)	0	7163 (63.6%)	n=17088 vs n=11259
Neuropathy	47	Sex, n(%)	Females	6369 (37.3%)	0	4096 (36.4%)	
Neuropathy	47	Smoking status, n(%)	No	12143 (78.4%)	9.4	8949 (79.5%)	
Neuropathy	47	Smoking status, n(%)	Yes	3346 (21.6%)	9.4	2310 (20.5%)	

Neuropathy	47	Stroke, n(%)	No	14946 (98.1%)	10.8	11056 (98.2%)	
Neuropathy	47	Stroke, n(%)	Yes	288 (1.9%)	10.8	203 (1.8%)	
Neuropathy	47	Systolic blood pressure, mean(SD)		129.9 (14.7)	4.2	129.6 (14.4)	
Neuropathy	47	Time since last healthcare visit, mean(SD)		0.8 (1.3)	0	0.7 (1)	
Neuropathy	47	Total cholesterol, mean(SD)		4.9 (1.1)	11.8	4.8 (1.1)	
Neuropathy	48	Age at diagnosis of type 2 diabetes, mean(SD)		44.4 (2.7)	0	43.9 (2.7)	
Neuropathy	48	BMI, mean(SD)		32.7 (6.2)	9.1	32.5 (6.1)	
Neuropathy	48	HbA1c, mean(SD)		52.2 (15.3)	2.6	52.2 (14.9)	
Neuropathy	48	HDL-cholesterol, mean(SD)		1.1 (0.3)	16.2	1.1 (0.3)	
Neuropathy	48	Ischemic heart disease, n(%)	No	15925 (95%)	10.5	11882 (95.4%)	
Neuropathy	48	Ischemic heart disease, n(%)	Yes	831 (5%)	10.5	576 (4.6%)	
Neuropathy	48	Self-reported physical activity, n(%)	Never	2204 (13.9%)	15	1734 (13.9%)	
Neuropathy	48	Self-reported physical activity, n(%)	<1 times/week	2187 (13.8%)	15	1638 (13.1%)	
Neuropathy	48	Self-reported physical activity, n(%)	regularly - 1-2 times/week	3376 (21.2%)	15	2643 (21.2%)	
Neuropathy	48	Self-reported physical activity, n(%)	regularly - 3-5 times/week	3986 (25.1%)	15	3176 (25.5%)	
Neuropathy	48	Self-reported physical activity, n(%)	Daily	4147 (26.1%)	15	3267 (26.2%)	
Neuropathy	48	Sex, n(%)	Males	11703 (62.5%)	0	7909 (63.5%)	n=18715 vs n=12458
Neuropathy	48	Sex, n(%)	Females	7012 (37.5%)	0	4549 (36.5%)	
Neuropathy	48	Smoking status, n(%)	No	13415 (78.7%)	8.9	9936 (79.8%)	
Neuropathy	48	Smoking status, n(%)	Yes	3627 (21.3%)	8.9	2522 (20.2%)	
Neuropathy	48	Stroke, n(%)	No	16378 (98%)	10.7	12223 (98.1%)	
Neuropathy	48	Stroke, n(%)	Yes	326 (2%)	10.7	235 (1.9%)	
Neuropathy	48	Systolic blood pressure, mean(SD)		130.5 (14.7)	4.1	130.2 (14.3)	

Neuropathy	48	Time since last healthcare visit, mean(SD)		0.8 (1.3)	0	0.7 (1)	
Neuropathy	48	Total cholesterol, mean(SD)		4.9 (1.1)	11.9	4.8 (1.1)	
Neuropathy	49	Age at diagnosis of type 2 diabetes, mean(SD)		45.3 (2.7)	0	44.8 (2.7)	
Neuropathy	49	BMI, mean(SD)		32.5 (6.1)	9.2	32.3 (6)	
Neuropathy	49	HbA1c, mean(SD)		52.2 (15.3)	2.6	52.1 (14.8)	
Neuropathy	49	HDL-cholesterol, mean(SD)		1.1 (0.3)	16.2	1.1 (0.3)	
Neuropathy	49	Ischemic heart disease, n(%)	No	17610 (94.6%)	10.1	13182 (94.9%)	
Neuropathy	49	Ischemic heart disease, n(%)	Yes	1014 (5.4%)	10.1	707 (5.1%)	
Neuropathy	49	Self-reported physical activity, n(%)	Never	2482 (14%)	14.4	1954 (14.1%)	
Neuropathy	49	Self-reported physical activity, n(%)	<1 times/week	2405 (13.6%)	14.4	1808 (13%)	
Neuropathy	49	Self-reported physical activity, n(%)	regularly - 1-2 times/week	3723 (21%)	14.4	2911 (21%)	
Neuropathy	49	Self-reported physical activity, n(%)	regularly - 3-5 times/week	4324 (24.4%)	14.4	3417 (24.6%)	
Neuropathy	49	Self-reported physical activity, n(%)	Daily	4804 (27.1%)	14.4	3799 (27.4%)	
Neuropathy	49	Sex, n(%)	Males	12965 (62.6%)	0	8848 (63.7%)	n=20719 vs n=13889
Neuropathy	49	Sex, n(%)	Females	7754 (37.4%)	0	5041 (36.3%)	
Neuropathy	49	Smoking status, n(%)	No	14802 (78.4%)	8.9	11065 (79.7%)	
Neuropathy	49	Smoking status, n(%)	Yes	4075 (21.6%)	8.9	2824 (20.3%)	
Neuropathy	49	Stroke, n(%)	No	18178 (98%)	10.5	13636 (98.2%)	
Neuropathy	49	Stroke, n(%)	Yes	368 (2%)	10.5	253 (1.8%)	
Neuropathy	49	Systolic blood pressure, mean(SD)		130.9 (14.8)	4	130.6 (14.4)	
Neuropathy	49	Time since last healthcare visit, mean(SD)		0.8 (1.3)	0	0.7 (1)	
Neuropathy	49	Total cholesterol, mean(SD)		4.9 (1.2)	11.8	4.8 (1.1)	
Neuropathy	50	Age at diagnosis of type 2 diabetes, mean(SD)		46.3 (2.7)	0	45.8 (2.7)	

Neuropathy	50	BMI, mean(SD)		32.4 (6)	9.3	32.2 (5.9)	
Neuropathy	50	HbA1c, mean(SD)		52.1 (15)	2.5	52.2 (14.6)	
Neuropathy	50	HDL-cholesterol, mean(SD)		1.1 (0.3)	15.8	1.1 (0.3)	
Neuropathy	50	Ischemic heart disease, n(%)	No	19392 (94%)	10.2	14542 (94.3%)	
Neuropathy	50	Ischemic heart disease, n(%)	Yes	1239 (6%)	10.2	875 (5.7%)	
Neuropathy	50	Self-reported physical activity, n(%)	Never	2735 (13.9%)	14.5	2157 (14%)	
Neuropathy	50	Self-reported physical activity, n(%)	<1 times/week	2700 (13.7%)	14.5	2050 (13.3%)	
Neuropathy	50	Self-reported physical activity, n(%)	regularly - 1-2 times/week	4060 (20.7%)	14.5	3200 (20.8%)	
Neuropathy	50	Self-reported physical activity, n(%)	regularly - 3-5 times/week	4843 (24.7%)	14.5	3826 (24.8%)	
Neuropathy	50	Self-reported physical activity, n(%)	Daily	5309 (27%)	14.5	4184 (27.1%)	
Neuropathy	50	Sex, n(%)	Males	14405 (62.7%)	0	9823 (63.7%)	n=22975 vs n=15417
Neuropathy	50	Sex, n(%)	Females	8570 (37.3%)	0	5594 (36.3%)	
Neuropathy	50	Smoking status, n(%)	No	16391 (78.3%)	8.9	12238 (79.4%)	
Neuropathy	50	Smoking status, n(%)	Yes	4549 (21.7%)	8.9	3179 (20.6%)	
Neuropathy	50	Stroke, n(%)	No	20125 (97.9%)	10.5	15109 (98%)	
Neuropathy	50	Stroke, n(%)	Yes	442 (2.1%)	10.5	308 (2%)	
Neuropathy	50	Systolic blood pressure, mean(SD)		131.4 (14.9)	4	131.1 (14.5)	
Neuropathy	50	Time since last healthcare visit, mean(SD)		0.8 (1.2)	0	0.6 (1)	
Neuropathy	50	Total cholesterol, mean(SD)		4.9 (1.1)	11.6	4.8 (1.1)	
Neuropathy	51	Age at diagnosis of type 2 diabetes, mean(SD)		47.3 (2.7)	0	46.8 (2.8)	
Neuropathy	51	BMI, mean(SD)		32.2 (6)	8.8	32.1 (5.9)	
Neuropathy	51	HbA1c, mean(SD)		52.1 (14.8)	2.2	52.1 (14.3)	
Neuropathy	51	HDL-cholesterol, mean(SD)		1.1 (0.3)	15	1.2 (0.3)	

Neuropathy	51	Ischemic heart disease, n(%)	No	21143 (93.5%)	9.9	15994 (93.9%)	
Neuropathy	51	Ischemic heart disease, n(%)	Yes	1467 (6.5%)	9.9	1048 (6.1%)	
Neuropathy	51	Self-reported physical activity, n(%)	Never	2972 (13.8%)	14.1	2354 (13.8%)	
Neuropathy	51	Self-reported physical activity, n(%)	<1 times/week	2957 (13.7%)	14.1	2285 (13.4%)	
Neuropathy	51	Self-reported physical activity, n(%)	regularly - 1-2 times/week	4412 (20.5%)	14.1	3497 (20.5%)	
Neuropathy	51	Self-reported physical activity, n(%)	regularly - 3-5 times/week	5433 (25.2%)	14.1	4351 (25.5%)	
Neuropathy	51	Self-reported physical activity, n(%)	Daily	5771 (26.8%)	14.1	4555 (26.7%)	
Neuropathy	51	Sex, n(%)	Males	15693 (62.6%)	0	10813 (63.4%)	n=25087 vs n=17042
Neuropathy	51	Sex, n(%)	Females	9394 (37.4%)	0	6229 (36.6%)	
Neuropathy	51	Smoking status, n(%)	No	17981 (78.3%)	8.5	13533 (79.4%)	
Neuropathy	51	Smoking status, n(%)	Yes	4970 (21.7%)	8.5	3509 (20.6%)	
Neuropathy	51	Stroke, n(%)	No	22031 (97.7%)	10.1	16673 (97.8%)	
Neuropathy	51	Stroke, n(%)	Yes	517 (2.3%)	10.1	369 (2.2%)	
Neuropathy	51	Systolic blood pressure, mean(SD)		131.7 (14.9)	3.7	131.4 (14.6)	
Neuropathy	51	Time since last healthcare visit, mean(SD)		0.8 (1.2)	0	0.6 (1)	
Neuropathy	51	Total cholesterol, mean(SD)		4.9 (1.2)	11	4.8 (1.1)	
Neuropathy	52	Age at diagnosis of type 2 diabetes, mean(SD)		48.2 (2.7)	0	47.7 (2.8)	
Neuropathy	52	BMI, mean(SD)		32.1 (5.9)	8.8	32 (5.8)	
Neuropathy	52	HbA1c, mean(SD)		52.1 (14.7)	2.3	52.1 (14.1)	
Neuropathy	52	HDL-cholesterol, mean(SD)		1.2 (0.3)	14.7	1.2 (0.3)	
Neuropathy	52	Ischemic heart disease, n(%)	No	22613 (93.2%)	9.8	17149 (93.5%)	
Neuropathy	52	Ischemic heart disease, n(%)	Yes	1653 (6.8%)	9.8	1191 (6.5%)	
Neuropathy	52	Self-reported physical activity, n(%)	Never	3180 (13.7%)	13.8	2491 (13.6%)	

Neuropathy	52	Self-reported physical activity, n(%)	<1 times/week	3148 (13.6%)	13.8	2429 (13.2%)	
Neuropathy	52	Self-reported physical activity, n(%)	regularly - 1-2 times/week	4763 (20.5%)	13.8	3729 (20.3%)	
Neuropathy	52	Self-reported physical activity, n(%)	regularly - 3-5 times/week	5720 (24.7%)	13.8	4609 (25.1%)	
Neuropathy	52	Self-reported physical activity, n(%)	Daily	6369 (27.5%)	13.8	5082 (27.7%)	
Neuropathy	52	Sex, n(%)	Males	16794 (62.4%)	0	11562 (63%)	n=26898 vs n=18340
Neuropathy	52	Sex, n(%)	Females	10104 (37.6%)	0	6778 (37%)	
Neuropathy	52	Smoking status, n(%)	No	19262 (78.1%)	8.4	14538 (79.3%)	
Neuropathy	52	Smoking status, n(%)	Yes	5389 (21.9%)	8.4	3802 (20.7%)	
Neuropathy	52	Stroke, n(%)	No	23567 (97.5%)	10.2	17913 (97.7%)	
Neuropathy	52	Stroke, n(%)	Yes	594 (2.5%)	10.2	427 (2.3%)	
Neuropathy	52	Systolic blood pressure, mean(SD)		132.1 (14.9)	3.5	131.8 (14.6)	
Neuropathy	52	Time since last healthcare visit, mean(SD)		0.8 (1.2)	0	0.6 (1)	
Neuropathy	52	Total cholesterol, mean(SD)		4.9 (1.2)	10.7	4.8 (1.1)	
Neuropathy	53	Age at diagnosis of type 2 diabetes, mean(SD)		49.2 (2.7)	0	48.7 (2.8)	
Neuropathy	53	BMI, mean(SD)		31.9 (5.8)	8.9	31.8 (5.7)	
Neuropathy	53	HbA1c, mean(SD)		52 (14.6)	2.4	52 (14.1)	
Neuropathy	53	HDL-cholesterol, mean(SD)		1.2 (0.3)	15.1	1.2 (0.3)	
Neuropathy	53	Ischemic heart disease, n(%)	No	23775 (92.6%)	9.9	18061 (93%)	
Neuropathy	53	Ischemic heart disease, n(%)	Yes	1887 (7.4%)	9.9	1365 (7%)	
Neuropathy	53	Self-reported physical activity, n(%)	Never	3307 (13.5%)	13.9	2582 (13.3%)	
Neuropathy	53	Self-reported physical activity, n(%)	<1 times/week	3265 (13.3%)	13.9	2549 (13.1%)	
Neuropathy	53	Self-reported physical activity, n(%)	regularly - 1-2 times/week	5092 (20.8%)	13.9	4024 (20.7%)	
Neuropathy	53	Self-reported physical activity, n(%)	regularly - 3-5 times/week	6062 (24.7%)	13.9	4886 (25.2%)	

Neuropathy	53	Self-reported physical activity, n(%)	Daily	6801 (27.7%)	13.9	5385 (27.7%)	
Neuropathy	53	Sex, n(%)	Males	17691 (62.1%)	0	12192 (62.8%)	n=28472 vs n=19426
Neuropathy	53	Sex, n(%)	Females	10781 (37.9%)	0	7234 (37.2%)	
Neuropathy	53	Smoking status, n(%)	No	20273 (77.9%)	8.6	15363 (79.1%)	
Neuropathy	53	Smoking status, n(%)	Yes	5738 (22.1%)	8.6	4063 (20.9%)	
Neuropathy	53	Stroke, n(%)	No	24860 (97.2%)	10.2	18905 (97.3%)	
Neuropathy	53	Stroke, n(%)	Yes	706 (2.8%)	10.2	521 (2.7%)	
Neuropathy	53	Systolic blood pressure, mean(SD)		132.3 (14.8)	3.6	132 (14.4)	
Neuropathy	53	Time since last healthcare visit, mean(SD)		0.8 (1.2)	0	0.6 (1)	
Neuropathy	53	Total cholesterol, mean(SD)		4.9 (1.2)	11	4.8 (1.2)	
Neuropathy	54	Age at diagnosis of type 2 diabetes, mean(SD)		50.2 (2.8)	0	49.6 (2.8)	
Neuropathy	54	BMI, mean(SD)		31.8 (5.8)	8.7	31.7 (5.6)	
Neuropathy	54	HbA1c, mean(SD)		52 (14.4)	2.5	52 (13.9)	
Neuropathy	54	HDL-cholesterol, mean(SD)		1.2 (0.3)	15	1.2 (0.3)	
Neuropathy	54	Ischemic heart disease, n(%)	No	25009 (92%)	9.5	19043 (92.3%)	
Neuropathy	54	Ischemic heart disease, n(%)	Yes	2170 (8%)	9.5	1582 (7.7%)	
Neuropathy	54	Self-reported physical activity, n(%)	Never	3365 (13%)	13.8	2628 (12.7%)	
Neuropathy	54	Self-reported physical activity, n(%)	<1 times/week	3484 (13.5%)	13.8	2709 (13.1%)	
Neuropathy	54	Self-reported physical activity, n(%)	regularly - 1-2 times/week	5329 (20.6%)	13.8	4252 (20.6%)	
Neuropathy	54	Self-reported physical activity, n(%)	regularly - 3-5 times/week	6407 (24.7%)	13.8	5179 (25.1%)	
Neuropathy	54	Self-reported physical activity, n(%)	Daily	7307 (28.2%)	13.8	5857 (28.4%)	
Neuropathy	54	Sex, n(%)	Males	18617 (62%)	0	12902 (62.6%)	n=30045 vs n=20625
Neuropathy	54	Sex, n(%)	Females	11428 (38%)	0	7723 (37.4%)	

Neuropathy	54	Smoking status, n(%)	No	21460 (77.9%)	8.3	16294 (79%)	
Neuropathy	54	Smoking status, n(%)	Yes	6093 (22.1%)	8.3	4331 (21%)	
Neuropathy	54	Stroke, n(%)	No	26294 (97.1%)	9.9	20048 (97.2%)	
Neuropathy	54	Stroke, n(%)	Yes	775 (2.9%)	9.9	577 (2.8%)	
Neuropathy	54	Systolic blood pressure, mean(SD)		132.4 (14.8)	3.6	132.1 (14.5)	
Neuropathy	54	Time since last healthcare visit, mean(SD)		0.8 (1.2)	0	0.6 (0.9)	
Neuropathy	54	Total cholesterol, mean(SD)		4.9 (1.2)	10.8	4.8 (1.2)	
Neuropathy	55	Age at diagnosis of type 2 diabetes, mean(SD)		51.2 (2.8)	0	50.6 (2.8)	
Neuropathy	55	BMI, mean(SD)		31.6 (5.7)	8.9	31.5 (5.6)	
Neuropathy	55	HbA1c, mean(SD)		51.8 (14.2)	2.5	51.8 (13.7)	
Neuropathy	55	HDL-cholesterol, mean(SD)		1.2 (0.3)	15	1.2 (0.3)	
Neuropathy	55	Ischemic heart disease, n(%)	No	26067 (91.3%)	9.7	19835 (91.7%)	
Neuropathy	55	Ischemic heart disease, n(%)	Yes	2485 (8.7%)	9.7	1802 (8.3%)	
Neuropathy	55	Self-reported physical activity, n(%)	Never	3514 (12.9%)	13.7	2749 (12.7%)	
Neuropathy	55	Self-reported physical activity, n(%)	<1 times/week	3654 (13.4%)	13.7	2841 (13.1%)	
Neuropathy	55	Self-reported physical activity, n(%)	regularly - 1-2 times/week	5694 (20.9%)	13.7	4481 (20.7%)	
Neuropathy	55	Self-reported physical activity, n(%)	regularly - 3-5 times/week	6717 (24.6%)	13.7	5373 (24.8%)	
Neuropathy	55	Self-reported physical activity, n(%)	Daily	7700 (28.2%)	13.7	6193 (28.6%)	
Neuropathy	55	Sex, n(%)	Males	19588 (61.9%)	0	13474 (62.3%)	n=31627 vs n=21637
Neuropathy	55	Sex, n(%)	Females	12039 (38.1%)	0	8163 (37.7%)	
Neuropathy	55	Smoking status, n(%)	No	22598 (77.8%)	8.2	17070 (78.9%)	
Neuropathy	55	Smoking status, n(%)	Yes	6447 (22.2%)	8.2	4567 (21.1%)	
Neuropathy	55	Stroke, n(%)	No	27558 (96.9%)	10.1	20979 (97%)	

Neuropathy	55	Stroke, n(%)	Yes	874 (3.1%)	10.1	658 (3%)	
Neuropathy	55	Systolic blood pressure, mean(SD)		132.8 (14.9)	3.6	132.4 (14.6)	
Neuropathy	55	Time since last healthcare visit, mean(SD)		0.8 (1.2)	0	0.6 (0.9)	
Neuropathy	55	Total cholesterol, mean(SD)		4.9 (1.2)	10.8	4.8 (1.2)	
Neuropathy	56	Age at diagnosis of type 2 diabetes, mean(SD)		52.1 (2.8)	0	51.6 (2.8)	
Neuropathy	56	BMI, mean(SD)		31.5 (5.6)	8.9	31.4 (5.5)	
Neuropathy	56	HbA1c, mean(SD)		51.5 (13.8)	2.3	51.5 (13.2)	
Neuropathy	56	HDL-cholesterol, mean(SD)		1.2 (0.4)	14.8	1.2 (0.3)	
Neuropathy	56	Ischemic heart disease, n(%)	No	27055 (90.5%)	9.8	20695 (90.9%)	
Neuropathy	56	Ischemic heart disease, n(%)	Yes	2834 (9.5%)	9.8	2080 (9.1%)	
Neuropathy	56	Self-reported physical activity, n(%)	Never	3704 (12.9%)	13.6	2909 (12.8%)	
Neuropathy	56	Self-reported physical activity, n(%)	<1 times/week	3768 (13.2%)	13.6	2892 (12.7%)	
Neuropathy	56	Self-reported physical activity, n(%)	regularly - 1-2 times/week	5989 (20.9%)	13.6	4759 (20.9%)	
Neuropathy	56	Self-reported physical activity, n(%)	regularly - 3-5 times/week	7090 (24.8%)	13.6	5740 (25.2%)	
Neuropathy	56	Self-reported physical activity, n(%)	Daily	8068 (28.2%)	13.6	6475 (28.4%)	
Neuropathy	56	Sex, n(%)	Males	20481 (61.8%)	0	14167 (62.2%)	n=33120 vs n=22775
Neuropathy	56	Sex, n(%)	Females	12639 (38.2%)	0	8608 (37.8%)	
Neuropathy	56	Smoking status, n(%)	No	23677 (77.9%)	8.2	17940 (78.8%)	
Neuropathy	56	Smoking status, n(%)	Yes	6731 (22.1%)	8.2	4835 (21.2%)	
Neuropathy	56	Stroke, n(%)	No	28752 (96.7%)	10.2	22027 (96.7%)	
Neuropathy	56	Stroke, n(%)	Yes	996 (3.3%)	10.2	748 (3.3%)	
Neuropathy	56	Systolic blood pressure, mean(SD)		132.9 (14.8)	3.4	132.5 (14.4)	
Neuropathy	56	Time since last healthcare visit, mean(SD)		0.8 (1.2)	0	0.6 (0.9)	

Neuropathy	56	Total cholesterol, mean(SD)		4.9 (1.2)	10.6	4.8 (1.2)	
Neuropathy	57	Age at diagnosis of type 2 diabetes, mean(SD)		53.1 (2.8)	0	52.6 (2.8)	
Neuropathy	57	BMI, mean(SD)		31.3 (5.6)	8.8	31.3 (5.5)	
Neuropathy	57	HbA1c, mean(SD)		51.4 (13.6)	2.3	51.4 (13)	
Neuropathy	57	HDL-cholesterol, mean(SD)		1.2 (0.4)	14.5	1.2 (0.4)	
Neuropathy	57	Ischemic heart disease, n(%)	No	27998 (89.6%)	9.6	21500 (90%)	
Neuropathy	57	Ischemic heart disease, n(%)	Yes	3246 (10.4%)	9.6	2381 (10%)	
Neuropathy	57	Self-reported physical activity, n(%)	Never	3755 (12.6%)	13.5	2969 (12.4%)	
Neuropathy	57	Self-reported physical activity, n(%)	<1 times/week	3910 (13.1%)	13.5	3057 (12.8%)	
Neuropathy	57	Self-reported physical activity, n(%)	regularly - 1-2 times/week	6279 (21%)	13.5	5069 (21.2%)	
Neuropathy	57	Self-reported physical activity, n(%)	regularly - 3-5 times/week	7362 (24.6%)	13.5	5917 (24.8%)	
Neuropathy	57	Self-reported physical activity, n(%)	Daily	8598 (28.8%)	13.5	6869 (28.8%)	
Neuropathy	57	Sex, n(%)	Males	21315 (61.7%)	0	14746 (61.7%)	n=34559 vs n=23881
Neuropathy	57	Sex, n(%)	Females	13244 (38.3%)	0	9135 (38.3%)	
Neuropathy	57	Smoking status, n(%)	No	24962 (78.5%)	7.9	18932 (79.3%)	
Neuropathy	57	Smoking status, n(%)	Yes	6853 (21.5%)	7.9	4949 (20.7%)	
Neuropathy	57	Stroke, n(%)	No	29974 (96.5%)	10.1	23054 (96.5%)	
Neuropathy	57	Stroke, n(%)	Yes	1098 (3.5%)	10.1	827 (3.5%)	
Neuropathy	57	Systolic blood pressure, mean(SD)		133.3 (15)	3.4	133 (14.7)	
Neuropathy	57	Time since last healthcare visit, mean(SD)		0.7 (1.2)	0	0.6 (0.9)	
Neuropathy	57	Total cholesterol, mean(SD)		4.9 (1.2)	10.5	4.8 (1.2)	
Neuropathy	58	Age at diagnosis of type 2 diabetes, mean(SD)		54.1 (2.8)	0	53.6 (2.8)	
Neuropathy	58	BMI, mean(SD)		31.2 (5.5)	8.6	31.1 (5.4)	

Neuropathy	58	HbA1c, mean(SD)		51.2 (13.4)	2.3	51.2 (12.8)	
Neuropathy	58	HDL-cholesterol, mean(SD)		1.2 (0.4)	14.3	1.2 (0.4)	
Neuropathy	58	Ischemic heart disease, n(%)	No	29283 (89.1%)	9.3	22564 (89.6%)	
Neuropathy	58	Ischemic heart disease, n(%)	Yes	3573 (10.9%)	9.3	2617 (10.4%)	
Neuropathy	58	Self-reported physical activity, n(%)	Never	3855 (12.3%)	13.4	3069 (12.2%)	
Neuropathy	58	Self-reported physical activity, n(%)	<1 times/week	4029 (12.8%)	13.4	3167 (12.6%)	
Neuropathy	58	Self-reported physical activity, n(%)	regularly - 1-2 times/week	6648 (21.2%)	13.4	5329 (21.2%)	
Neuropathy	58	Self-reported physical activity, n(%)	regularly - 3-5 times/week	7686 (24.5%)	13.4	6225 (24.7%)	
Neuropathy	58	Self-reported physical activity, n(%)	Daily	9150 (29.2%)	13.4	7391 (29.4%)	
Neuropathy	58	Sex, n(%)	Males	22235 (61.4%)	0	15532 (61.7%)	n=36235 vs n=25181
Neuropathy	58	Sex, n(%)	Females	14000 (38.6%)	0	9649 (38.3%)	
Neuropathy	58	Smoking status, n(%)	No	26365 (78.9%)	7.8	20079 (79.7%)	
Neuropathy	58	Smoking status, n(%)	Yes	7044 (21.1%)	7.8	5102 (20.3%)	
Neuropathy	58	Stroke, n(%)	No	31433 (96.2%)	9.8	24250 (96.3%)	
Neuropathy	58	Stroke, n(%)	Yes	1252 (3.8%)	9.8	931 (3.7%)	
Neuropathy	58	Systolic blood pressure, mean(SD)		133.6 (14.9)	3.3	133.2 (14.7)	
Neuropathy	58	Time since last healthcare visit, mean(SD)		0.7 (1.1)	0	0.6 (0.9)	
Neuropathy	58	Total cholesterol, mean(SD)		4.9 (1.2)	10.3	4.8 (1.2)	
Neuropathy	59	Age at diagnosis of type 2 diabetes, mean(SD)		55.1 (2.8)	0	54.6 (2.8)	
Neuropathy	59	BMI, mean(SD)		31 (5.4)	8.6	31 (5.4)	
Neuropathy	59	HbA1c, mean(SD)		50.9 (12.9)	2.2	51 (12.3)	
Neuropathy	59	HDL-cholesterol, mean(SD)		1.2 (0.4)	14.1	1.2 (0.4)	
Neuropathy	59	Ischemic heart disease, n(%)	No	30517 (88.3%)	9.3	23568 (88.8%)	

Neuropathy	59	Ischemic heart disease, n(%)	Yes	4043 (11.7%)	9.3	2960 (11.2%)	
Neuropathy	59	Self-reported physical activity, n(%)	Never	4002 (12.1%)	13.2	3180 (12%)	
Neuropathy	59	Self-reported physical activity, n(%)	<1 times/week	4253 (12.9%)	13.2	3353 (12.6%)	
Neuropathy	59	Self-reported physical activity, n(%)	regularly - 1-2 times/week	6959 (21%)	13.2	5552 (20.9%)	
Neuropathy	59	Self-reported physical activity, n(%)	regularly - 3-5 times/week	8161 (24.7%)	13.2	6587 (24.8%)	
Neuropathy	59	Self-reported physical activity, n(%)	Daily	9692 (29.3%)	13.2	7856 (29.6%)	
Neuropathy	59	Sex, n(%)	Males	23361 (61.3%)	0	16322 (61.5%)	n=38103 vs n=26528
Neuropathy	59	Sex, n(%)	Females	14742 (38.7%)	0	10206 (38.5%)	
Neuropathy	59	Smoking status, n(%)	No	27938 (79.5%)	7.7	21336 (80.4%)	
Neuropathy	59	Smoking status, n(%)	Yes	7218 (20.5%)	7.7	5192 (19.6%)	
Neuropathy	59	Stroke, n(%)	No	33010 (96%)	9.8	25531 (96.2%)	
Neuropathy	59	Stroke, n(%)	Yes	1363 (4%)	9.8	997 (3.8%)	
Neuropathy	59	Systolic blood pressure, mean(SD)		134 (15.1)	3.3	133.7 (14.8)	
Neuropathy	59	Time since last healthcare visit, mean(SD)		0.7 (1.1)	0	0.6 (0.9)	
Neuropathy	59	Total cholesterol, mean(SD)		4.9 (1.2)	10	4.8 (1.2)	
Neuropathy	60	Age at diagnosis of type 2 diabetes, mean(SD)		56.1 (2.8)	0	55.6 (2.8)	
Neuropathy	60	BMI, mean(SD)		30.9 (5.3)	8.6	30.9 (5.3)	
Neuropathy	60	HbA1c, mean(SD)		50.7 (12.8)	2.3	50.8 (12.2)	
Neuropathy	60	HDL-cholesterol, mean(SD)		1.2 (0.4)	14.1	1.2 (0.4)	
Neuropathy	60	Ischemic heart disease, n(%)	No	31851 (87.7%)	9.5	24499 (88.2%)	
Neuropathy	60	Ischemic heart disease, n(%)	Yes	4462 (12.3%)	9.5	3283 (11.8%)	
Neuropathy	60	Self-reported physical activity, n(%)	Never	4182 (12.1%)	13.5	3281 (11.8%)	
Neuropathy	60	Self-reported physical activity, n(%)	<1 times/week	4395 (12.7%)	13.5	3429 (12.3%)	

Neuropathy	60	Self-reported physical activity, n(%)	regularly - 1-2 times/week	7292 (21%)	13.5	5838 (21%)	
Neuropathy	60	Self-reported physical activity, n(%)	regularly - 3-5 times/week	8713 (25.1%)	13.5	7001 (25.2%)	
Neuropathy	60	Self-reported physical activity, n(%)	Daily	10113 (29.1%)	13.5	8233 (29.6%)	
Neuropathy	60	Sex, n(%)	Males	24422 (60.9%)	0	16977 (61.1%)	n=40109 vs n=27782
Neuropathy	60	Sex, n(%)	Females	15687 (39.1%)	0	10805 (38.9%)	
Neuropathy	60	Smoking status, n(%)	No	29565 (79.9%)	7.8	22479 (80.9%)	
Neuropathy	60	Smoking status, n(%)	Yes	7425 (20.1%)	7.8	5303 (19.1%)	
Neuropathy	60	Stroke, n(%)	No	34595 (95.7%)	9.9	26673 (96%)	
Neuropathy	60	Stroke, n(%)	Yes	1536 (4.3%)	9.9	1109 (4%)	
Neuropathy	60	Systolic blood pressure, mean(SD)		134.4 (15)	3.3	134 (14.7)	
Neuropathy	60	Time since last healthcare visit, mean(SD)		0.7 (1.1)	0	0.6 (0.9)	
Neuropathy	60	Total cholesterol, mean(SD)		4.9 (1.2)	10.1	4.8 (1.1)	
Neuropathy	61	Age at diagnosis of type 2 diabetes, mean(SD)		57.1 (2.8)	0	56.6 (2.8)	
Neuropathy	61	BMI, mean(SD)		30.8 (5.3)	8.7	30.7 (5.3)	
Neuropathy	61	HbA1c, mean(SD)		50.5 (12.5)	2.3	50.6 (12.1)	
Neuropathy	61	HDL-cholesterol, mean(SD)		1.2 (0.4)	14	1.2 (0.4)	
Neuropathy	61	Ischemic heart disease, n(%)	No	33672 (87%)	9.5	26017 (87.6%)	
Neuropathy	61	Ischemic heart disease, n(%)	Yes	5042 (13%)	9.5	3678 (12.4%)	
Neuropathy	61	Self-reported physical activity, n(%)	Never	4383 (11.8%)	13.4	3388 (11.4%)	
Neuropathy	61	Self-reported physical activity, n(%)	<1 times/week	4676 (12.6%)	13.4	3630 (12.2%)	
Neuropathy	61	Self-reported physical activity, n(%)	regularly - 1-2 times/week	7763 (21%)	13.4	6256 (21.1%)	
Neuropathy	61	Self-reported physical activity, n(%)	regularly - 3-5 times/week	9336 (25.2%)	13.4	7517 (25.3%)	
Neuropathy	61	Self-reported physical activity, n(%)	Daily	10885 (29.4%)	13.4	8904 (30%)	

Neuropathy	61	Sex, n(%)	Males	25931 (60.6%)	0	18119 (61%)	n=42779 vs n=29695
Neuropathy	61	Sex, n(%)	Females	16848 (39.4%)	0	11576 (39%)	
Neuropathy	61	Smoking status, n(%)	No	31740 (80.5%)	7.8	24171 (81.4%)	
Neuropathy	61	Smoking status, n(%)	Yes	7712 (19.5%)	7.8	5524 (18.6%)	
Neuropathy	61	Stroke, n(%)	No	36810 (95.6%)	10	28478 (95.9%)	
Neuropathy	61	Stroke, n(%)	Yes	1706 (4.4%)	10	1217 (4.1%)	
Neuropathy	61	Systolic blood pressure, mean(SD)		134.5 (15)	3.2	134.2 (14.7)	
Neuropathy	61	Time since last healthcare visit, mean(SD)		0.7 (1.1)	0	0.6 (0.9)	
Neuropathy	61	Total cholesterol, mean(SD)		4.9 (1.2)	9.9	4.8 (1.1)	
Neuropathy	62	Age at diagnosis of type 2 diabetes, mean(SD)		58.1 (2.8)	0	57.6 (2.8)	
Neuropathy	62	BMI, mean(SD)		30.7 (5.3)	8.9	30.6 (5.2)	
Neuropathy	62	HbA1c, mean(SD)		50.2 (12.3)	2.3	50.4 (11.8)	
Neuropathy	62	HDL-cholesterol, mean(SD)		1.2 (0.4)	14	1.2 (0.4)	
Neuropathy	62	Ischemic heart disease, n(%)	No	34677 (86.2%)	9.6	26798 (86.8%)	
Neuropathy	62	Ischemic heart disease, n(%)	Yes	5571 (13.8%)	9.6	4087 (13.2%)	
Neuropathy	62	Self-reported physical activity, n(%)	Never	4523 (11.7%)	13.4	3532 (11.4%)	
Neuropathy	62	Self-reported physical activity, n(%)	<1 times/week	4986 (12.9%)	13.4	3869 (12.5%)	
Neuropathy	62	Self-reported physical activity, n(%)	regularly - 1-2 times/week	7891 (20.5%)	13.4	6334 (20.5%)	
Neuropathy	62	Self-reported physical activity, n(%)	regularly - 3-5 times/week	9661 (25.1%)	13.4	7802 (25.3%)	
Neuropathy	62	Self-reported physical activity, n(%)	Daily	11503 (29.8%)	13.4	9348 (30.3%)	
Neuropathy	62	Sex, n(%)	Males	26801 (60.2%)	0	18712 (60.6%)	n=44517 vs n=30885
Neuropathy	62	Sex, n(%)	Females	17716 (39.8%)	0	12173 (39.4%)	
Neuropathy	62	Smoking status, n(%)	No	33140 (80.8%)	7.9	25331 (82%)	

Neuropathy	62	Smoking status, n(%)	Yes	7861 (19.2%)	7.9	5554 (18%)	
Neuropathy	62	Stroke, n(%)	No	38162 (95.3%)	10.1	29525 (95.6%)	
Neuropathy	62	Stroke, n(%)	Yes	1880 (4.7%)	10.1	1360 (4.4%)	
Neuropathy	62	Systolic blood pressure, mean(SD)		135 (15.1)	3.1	134.6 (14.8)	
Neuropathy	62	Time since last healthcare visit, mean(SD)		0.7 (1.1)	0	0.6 (0.9)	
Neuropathy	62	Total cholesterol, mean(SD)		4.9 (1.2)	9.9	4.8 (1.1)	
Neuropathy	63	Age at diagnosis of type 2 diabetes, mean(SD)		59.1 (2.8)	0	58.5 (2.8)	
Neuropathy	63	BMI, mean(SD)		30.5 (5.3)	9	30.5 (5.2)	
Neuropathy	63	HbA1c, mean(SD)		50 (12)	2.3	50.2 (11.5)	
Neuropathy	63	HDL-cholesterol, mean(SD)		1.3 (0.4)	14.3	1.3 (0.4)	
Neuropathy	63	Ischemic heart disease, n(%)	No	35698 (85.4%)	9.7	27473 (85.9%)	
Neuropathy	63	Ischemic heart disease, n(%)	Yes	6107 (14.6%)	9.7	4511 (14.1%)	
Neuropathy	63	Self-reported physical activity, n(%)	Never	4686 (11.7%)	13.5	3650 (11.4%)	
Neuropathy	63	Self-reported physical activity, n(%)	<1 times/week	5059 (12.6%)	13.5	3941 (12.3%)	
Neuropathy	63	Self-reported physical activity, n(%)	regularly - 1-2 times/week	8251 (20.6%)	13.5	6551 (20.5%)	
Neuropathy	63	Self-reported physical activity, n(%)	regularly - 3-5 times/week	9956 (24.8%)	13.5	8002 (25%)	
Neuropathy	63	Self-reported physical activity, n(%)	Daily	12128 (30.3%)	13.5	9840 (30.8%)	
Neuropathy	63	Sex, n(%)	Males	27647 (59.7%)	0	19210 (60.1%)	n=46316 vs n=31984
Neuropathy	63	Sex, n(%)	Females	18669 (40.3%)	0	12774 (39.9%)	
Neuropathy	63	Smoking status, n(%)	No	34717 (81.4%)	7.9	26361 (82.4%)	
Neuropathy	63	Smoking status, n(%)	Yes	7958 (18.6%)	7.9	5623 (17.6%)	
Neuropathy	63	Stroke, n(%)	No	39496 (95%)	10.2	30492 (95.3%)	
Neuropathy	63	Stroke, n(%)	Yes	2073 (5%)	10.2	1492 (4.7%)	

Neuropathy	63	Systolic blood pressure, mean(SD)		135.3 (15.2)	3.2	134.9 (14.8)	
Neuropathy	63	Time since last healthcare visit, mean(SD)		0.7 (1.1)	0	0.6 (0.9)	
Neuropathy	63	Total cholesterol, mean(SD)		4.8 (1.2)	9.9	4.8 (1.1)	
Neuropathy	64	Age at diagnosis of type 2 diabetes, mean(SD)		60 (2.8)	0	59.5 (2.8)	
Neuropathy	64	BMI, mean(SD)		30.4 (5.3)	9	30.4 (5.2)	
Neuropathy	64	HbA1c, mean(SD)		49.8 (11.8)	2.3	49.9 (11.3)	
Neuropathy	64	HDL-cholesterol, mean(SD)		1.3 (0.4)	14.3	1.3 (0.4)	
Neuropathy	64	Ischemic heart disease, n(%)	No	36750 (84.8%)	9.6	28284 (85.3%)	
Neuropathy	64	Ischemic heart disease, n(%)	Yes	6611 (15.2%)	9.6	4877 (14.7%)	
Neuropathy	64	Self-reported physical activity, n(%)	Never	4628 (11.2%)	13.6	3596 (10.8%)	
Neuropathy	64	Self-reported physical activity, n(%)	<1 times/week	4975 (12%)	13.6	3875 (11.7%)	
Neuropathy	64	Self-reported physical activity, n(%)	regularly - 1-2 times/week	8364 (20.2%)	13.6	6663 (20.1%)	
Neuropathy	64	Self-reported physical activity, n(%)	regularly - 3-5 times/week	10454 (25.2%)	13.6	8470 (25.5%)	
Neuropathy	64	Self-reported physical activity, n(%)	Daily	13022 (31.4%)	13.6	10557 (31.8%)	
Neuropathy	64	Sex, n(%)	Males	28609 (59.6%)	0	19798 (59.7%)	n=47982 vs n=33161
Neuropathy	64	Sex, n(%)	Females	19373 (40.4%)	0	13363 (40.3%)	
Neuropathy	64	Smoking status, n(%)	No	36167 (82%)	8	27522 (83%)	
Neuropathy	64	Smoking status, n(%)	Yes	7960 (18%)	8	5639 (17%)	
Neuropathy	64	Stroke, n(%)	No	40807 (94.6%)	10.1	31488 (95%)	
Neuropathy	64	Stroke, n(%)	Yes	2314 (5.4%)	10.1	1673 (5%)	
Neuropathy	64	Systolic blood pressure, mean(SD)		135.4 (15.2)	3.2	134.9 (14.9)	
Neuropathy	64	Time since last healthcare visit, mean(SD)		0.7 (1.1)	0	0.6 (0.9)	
Neuropathy	64	Total cholesterol, mean(SD)		4.8 (1.1)	9.9	4.8 (1.1)	

Neuropathy	65	Age at diagnosis of type 2 diabetes, mean(SD)		61 (2.8)	0	60.5 (2.8)	
Neuropathy	65	BMI, mean(SD)		30.3 (5.2)	9.1	30.3 (5.2)	
Neuropathy	65	HbA1c, mean(SD)		49.4 (11.5)	2.3	49.5 (11)	
Neuropathy	65	HDL-cholesterol, mean(SD)		1.3 (0.4)	14.2	1.3 (0.4)	
Neuropathy	65	Ischemic heart disease, n(%)	No	37609 (84%)	9.7	28964 (84.6%)	
Neuropathy	65	Ischemic heart disease, n(%)	Yes	7139 (16%)	9.7	5276 (15.4%)	
Neuropathy	65	Self-reported physical activity, n(%)	Never	4742 (11.1%)	13.5	3679 (10.7%)	
Neuropathy	65	Self-reported physical activity, n(%)	<1 times/week	5025 (11.7%)	13.5	3911 (11.4%)	
Neuropathy	65	Self-reported physical activity, n(%)	regularly - 1-2 times/week	8594 (20%)	13.5	6815 (19.9%)	
Neuropathy	65	Self-reported physical activity, n(%)	regularly - 3-5 times/week	10632 (24.8%)	13.5	8606 (25.1%)	
Neuropathy	65	Self-reported physical activity, n(%)	Daily	13884 (32.4%)	13.5	11229 (32.8%)	
Neuropathy	65	Sex, n(%)	Males	29346 (59.2%)	0	20277 (59.2%)	n=49573 vs n=34240
Neuropathy	65	Sex, n(%)	Females	20227 (40.8%)	0	13963 (40.8%)	
Neuropathy	65	Smoking status, n(%)	No	37740 (82.7%)	7.9	28593 (83.5%)	
Neuropathy	65	Smoking status, n(%)	Yes	7898 (17.3%)	7.9	5647 (16.5%)	
Neuropathy	65	Stroke, n(%)	No	41967 (94.4%)	10.3	32422 (94.7%)	
Neuropathy	65	Stroke, n(%)	Yes	2511 (5.6%)	10.3	1818 (5.3%)	
Neuropathy	65	Systolic blood pressure, mean(SD)		135.5 (15.1)	3.1	135.1 (14.8)	
Neuropathy	65	Time since last healthcare visit, mean(SD)		0.7 (1.1)	0	0.6 (0.9)	
Neuropathy	65	Total cholesterol, mean(SD)		4.8 (1.1)	9.7	4.8 (1.1)	
Neuropathy	66	Age at diagnosis of type 2 diabetes, mean(SD)		62 (2.8)	0	61.4 (2.8)	
Neuropathy	66	BMI, mean(SD)		30.2 (5.2)	9.2	30.2 (5.1)	
Neuropathy	66	HbA1c, mean(SD)		49.2 (11.5)	2.2	49.3 (11)	

Neuropathy	66	HDL-cholesterol, mean(SD)		1.3 (0.4)	14	1.3 (0.4)	
Neuropathy	66	Ischemic heart disease, n(%)	No	38541 (83.4%)	9.6	29712 (84%)	
Neuropathy	66	Ischemic heart disease, n(%)	Yes	7654 (16.6%)	9.6	5675 (16%)	
Neuropathy	66	Self-reported physical activity, n(%)	Never	4690 (10.6%)	13.4	3623 (10.2%)	
Neuropathy	66	Self-reported physical activity, n(%)	<1 times/week	4960 (11.2%)	13.4	3856 (10.9%)	
Neuropathy	66	Self-reported physical activity, n(%)	regularly - 1-2 times/week	8503 (19.2%)	13.4	6722 (19%)	
Neuropathy	66	Self-reported physical activity, n(%)	regularly - 3-5 times/week	11032 (24.9%)	13.4	8944 (25.3%)	
Neuropathy	66	Self-reported physical activity, n(%)	Daily	15117 (34.1%)	13.4	12242 (34.6%)	
Neuropathy	66	Sex, n(%)	Males	30033 (58.7%)	0	20821 (58.8%)	n=51128 vs n=35387
Neuropathy	66	Sex, n(%)	Females	21095 (41.3%)	0	14566 (41.2%)	
Neuropathy	66	Smoking status, n(%)	No	39259 (83.3%)	7.9	29752 (84.1%)	
Neuropathy	66	Smoking status, n(%)	Yes	7854 (16.7%)	7.9	5635 (15.9%)	
Neuropathy	66	Stroke, n(%)	No	43217 (94.1%)	10.2	33427 (94.5%)	
Neuropathy	66	Stroke, n(%)	Yes	2714 (5.9%)	10.2	1960 (5.5%)	
Neuropathy	66	Systolic blood pressure, mean(SD)		135.8 (15.2)	3.1	135.4 (14.9)	
Neuropathy	66	Time since last healthcare visit, mean(SD)		0.7 (1.1)	0	0.6 (0.9)	
Neuropathy	66	Total cholesterol, mean(SD)		4.8 (1.1)	9.7	4.8 (1.1)	
Neuropathy	67	Age at diagnosis of type 2 diabetes, mean(SD)		62.9 (2.8)	0	62.4 (2.8)	
Neuropathy	67	BMI, mean(SD)		30.1 (5.2)	9.2	30.1 (5.1)	
Neuropathy	67	HbA1c, mean(SD)		49 (11.2)	2.2	49.1 (10.7)	
Neuropathy	67	HDL-cholesterol, mean(SD)		1.3 (0.4)	13.8	1.3 (0.4)	
Neuropathy	67	Ischemic heart disease, n(%)	No	39034 (82.8%)	9.6	30172 (83.5%)	
Neuropathy	67	Ischemic heart disease, n(%)	Yes	8122 (17.2%)	9.6	5966 (16.5%)	

Neuropathy	67	Self-reported physical activity, n(%)	Never	4700 (10.4%)	13.3	3595 (9.9%)	
Neuropathy	67	Self-reported physical activity, n(%)	<1 times/week	4889 (10.8%)	13.3	3771 (10.4%)	
Neuropathy	67	Self-reported physical activity, n(%)	regularly - 1-2 times/week	8455 (18.7%)	13.3	6652 (18.4%)	
Neuropathy	67	Self-reported physical activity, n(%)	regularly - 3-5 times/week	11245 (24.9%)	13.3	9164 (25.4%)	
Neuropathy	67	Self-reported physical activity, n(%)	Daily	15907 (35.2%)	13.3	12956 (35.9%)	
Neuropathy	67	Sex, n(%)	Males	30354 (58.2%)	0	21114 (58.4%)	n=52151 vs n=36138
Neuropathy	67	Sex, n(%)	Females	21797 (41.8%)	0	15024 (41.6%)	
Neuropathy	67	Smoking status, n(%)	No	40593 (84.4%)	7.7	30776 (85.2%)	
Neuropathy	67	Smoking status, n(%)	Yes	7523 (15.6%)	7.7	5362 (14.8%)	
Neuropathy	67	Stroke, n(%)	No	43959 (93.8%)	10.1	34046 (94.2%)	
Neuropathy	67	Stroke, n(%)	Yes	2915 (6.2%)	10.1	2092 (5.8%)	
Neuropathy	67	Systolic blood pressure, mean(SD)		136 (15.2)	3.1	135.6 (14.9)	
Neuropathy	67	Time since last healthcare visit, mean(SD)		0.7 (1.1)	0	0.6 (0.9)	
Neuropathy	67	Total cholesterol, mean(SD)		4.8 (1.1)	9.5	4.7 (1.1)	
Neuropathy	68	Age at diagnosis of type 2 diabetes, mean(SD)		63.9 (2.8)	0	63.3 (2.9)	
Neuropathy	68	BMI, mean(SD)		30 (5.2)	9	30 (5.1)	
Neuropathy	68	HbA1c, mean(SD)		48.8 (10.9)	2.2	48.8 (10.4)	
Neuropathy	68	HDL-cholesterol, mean(SD)		1.3 (0.4)	13.7	1.3 (0.4)	
Neuropathy	68	Ischemic heart disease, n(%)	No	39103 (82.1%)	9.6	30404 (82.8%)	
Neuropathy	68	Ischemic heart disease, n(%)	Yes	8520 (17.9%)	9.6	6305 (17.2%)	
Neuropathy	68	Self-reported physical activity, n(%)	Never	4757 (10.4%)	13.1	3657 (10%)	
Neuropathy	68	Self-reported physical activity, n(%)	<1 times/week	4977 (10.9%)	13.1	3857 (10.5%)	
Neuropathy	68	Self-reported physical activity, n(%)	regularly - 1-2 times/week	8234 (18%)	13.1	6490 (17.7%)	

Neuropathy	68	Self-reported physical activity, n(%)	regularly - 3-5 times/week	11403 (24.9%)	13.1	9237 (25.2%)	
Neuropathy	68	Self-reported physical activity, n(%)	Daily	16364 (35.8%)	13.1	13468 (36.7%)	
Neuropathy	68	Sex, n(%)	Males	30411 (57.8%)	0	21260 (57.9%)	n=52652 vs n=36709
Neuropathy	68	Sex, n(%)	Females	22241 (42.2%)	0	15449 (42.1%)	
Neuropathy	68	Smoking status, n(%)	No	41340 (85.1%)	7.7	31510 (85.8%)	
Neuropathy	68	Smoking status, n(%)	Yes	7232 (14.9%)	7.7	5199 (14.2%)	
Neuropathy	68	Stroke, n(%)	No	44201 (93.4%)	10.1	34437 (93.8%)	
Neuropathy	68	Stroke, n(%)	Yes	3111 (6.6%)	10.1	2272 (6.2%)	
Neuropathy	68	Systolic blood pressure, mean(SD)		136 (15.2)	2.9	135.6 (14.8)	
Neuropathy	68	Time since last healthcare visit, mean(SD)		0.7 (1.2)	0	0.6 (0.9)	
Neuropathy	68	Total cholesterol, mean(SD)		4.8 (1.1)	9.4	4.7 (1.1)	
Neuropathy	69	Age at diagnosis of type 2 diabetes, mean(SD)		64.8 (2.9)	0	64.2 (2.9)	
Neuropathy	69	BMI, mean(SD)		29.9 (5.1)	9	29.9 (5.1)	
Neuropathy	69	HbA1c, mean(SD)		48.6 (10.7)	2.3	48.7 (10.3)	
Neuropathy	69	HDL-cholesterol, mean(SD)		1.3 (0.4)	13.6	1.3 (0.4)	
Neuropathy	69	Ischemic heart disease, n(%)	No	38972 (81.4%)	9.1	30403 (82.1%)	
Neuropathy	69	Ischemic heart disease, n(%)	Yes	8901 (18.6%)	9.1	6608 (17.9%)	
Neuropathy	69	Self-reported physical activity, n(%)	Never	4734 (10.3%)	13.1	3695 (10%)	
Neuropathy	69	Self-reported physical activity, n(%)	<1 times/week	4958 (10.8%)	13.1	3846 (10.4%)	
Neuropathy	69	Self-reported physical activity, n(%)	regularly - 1-2 times/week	8296 (18.1%)	13.1	6610 (17.9%)	
Neuropathy	69	Self-reported physical activity, n(%)	regularly - 3-5 times/week	11257 (24.6%)	13.1	9202 (24.9%)	
Neuropathy	69	Self-reported physical activity, n(%)	Daily	16570 (36.2%)	13.1	13658 (36.9%)	
Neuropathy	69	Sex, n(%)	Males	30117 (57.2%)	0	21184 (57.2%)	n=52693 vs n=37011

Neuropathy	69	Sex, n(%)	Females	22576 (42.8%)	0	15827 (42.8%)	
Neuropathy	69	Smoking status, n(%)	No	41838 (86%)	7.7	32086 (86.7%)	
Neuropathy	69	Smoking status, n(%)	Yes	6815 (14%)	7.7	4925 (13.3%)	
Neuropathy	69	Stroke, n(%)	No	44317 (93.2%)	9.7	34619 (93.5%)	
Neuropathy	69	Stroke, n(%)	Yes	3256 (6.8%)	9.7	2392 (6.5%)	
Neuropathy	69	Systolic blood pressure, mean(SD)		136.1 (15.2)	2.9	135.8 (14.8)	
Neuropathy	69	Time since last healthcare visit, mean(SD)		0.8 (1.2)	0	0.6 (0.9)	
Neuropathy	69	Total cholesterol, mean(SD)		4.8 (1.1)	9.3	4.7 (1.1)	
Neuropathy	70	Age at diagnosis of type 2 diabetes, mean(SD)		65.7 (2.9)	0	65.1 (2.9)	
Neuropathy	70	BMI, mean(SD)		29.8 (5.1)	9	29.8 (5.1)	
Neuropathy	70	HbA1c, mean(SD)		48.5 (10.8)	2.3	48.5 (10.2)	
Neuropathy	70	HDL-cholesterol, mean(SD)		1.3 (0.4)	13.8	1.3 (0.4)	
Neuropathy	70	Ischemic heart disease, n(%)	No	38192 (80.6%)	9.1	29693 (81.4%)	
Neuropathy	70	Ischemic heart disease, n(%)	Yes	9169 (19.4%)	9.1	6766 (18.6%)	
Neuropathy	70	Self-reported physical activity, n(%)	Never	4708 (10.4%)	13.1	3714 (10.2%)	
Neuropathy	70	Self-reported physical activity, n(%)	<1 times/week	4819 (10.6%)	13.1	3729 (10.2%)	
Neuropathy	70	Self-reported physical activity, n(%)	regularly - 1-2 times/week	8109 (17.9%)	13.1	6436 (17.7%)	
Neuropathy	70	Self-reported physical activity, n(%)	regularly - 3-5 times/week	11181 (24.7%)	13.1	9117 (25%)	
Neuropathy	70	Self-reported physical activity, n(%)	Daily	16444 (36.3%)	13.1	13463 (36.9%)	
Neuropathy	70	Sex, n(%)	Males	29528 (56.7%)	0	20721 (56.8%)	n=52099 vs n=36459
Neuropathy	70	Sex, n(%)	Females	22571 (43.3%)	0	15738 (43.2%)	
Neuropathy	70	Smoking status, n(%)	No	41621 (86.7%)	7.9	31867 (87.4%)	
Neuropathy	70	Smoking status, n(%)	Yes	6385 (13.3%)	7.9	4592 (12.6%)	

Neuropathy	70	Stroke, n(%)	No	43683 (92.9%)	9.7	33971 (93.2%)	
Neuropathy	70	Stroke, n(%)	Yes	3353 (7.1%)	9.7	2488 (6.8%)	
Neuropathy	70	Systolic blood pressure, mean(SD)		136.2 (15.3)	2.9	135.9 (14.9)	
Neuropathy	70	Time since last healthcare visit, mean(SD)		0.8 (1.2)	0	0.6 (1)	
Neuropathy	70	Total cholesterol, mean(SD)		4.8 (1.1)	9.4	4.7 (1.1)	
Neuropathy	71	Age at diagnosis of type 2 diabetes, mean(SD)		66.7 (2.9)	0	66.1 (2.9)	
Neuropathy	71	BMI, mean(SD)		29.7 (5.1)	9	29.6 (5)	
Neuropathy	71	HbA1c, mean(SD)		48.4 (10.6)	2.1	48.5 (10)	
Neuropathy	71	HDL-cholesterol, mean(SD)		1.3 (0.4)	13.8	1.3 (0.4)	
Neuropathy	71	Ischemic heart disease, n(%)	No	36971 (79.7%)	8.9	28685 (80.3%)	
Neuropathy	71	Ischemic heart disease, n(%)	Yes	9436 (20.3%)	8.9	7043 (19.7%)	
Neuropathy	71	Self-reported physical activity, n(%)	Never	4596 (10.4%)	12.9	3598 (10.1%)	
Neuropathy	71	Self-reported physical activity, n(%)	<1 times/week	4923 (11.1%)	12.9	3823 (10.7%)	
Neuropathy	71	Self-reported physical activity, n(%)	regularly - 1-2 times/week	7971 (18%)	12.9	6333 (17.7%)	
Neuropathy	71	Self-reported physical activity, n(%)	regularly - 3-5 times/week	10714 (24.1%)	12.9	8727 (24.4%)	
Neuropathy	71	Self-reported physical activity, n(%)	Daily	16172 (36.4%)	12.9	13247 (37.1%)	
Neuropathy	71	Sex, n(%)	Males	28621 (56.2%)	0	20059 (56.1%)	n=50943 vs n=35728
Neuropathy	71	Sex, n(%)	Females	22322 (43.8%)	0	15669 (43.9%)	
Neuropathy	71	Smoking status, n(%)	No	41052 (87.5%)	7.9	31520 (88.2%)	
Neuropathy	71	Smoking status, n(%)	Yes	5851 (12.5%)	7.9	4208 (11.8%)	
Neuropathy	71	Stroke, n(%)	No	42592 (92.4%)	9.5	33174 (92.9%)	
Neuropathy	71	Stroke, n(%)	Yes	3486 (7.6%)	9.5	2554 (7.1%)	
Neuropathy	71	Systolic blood pressure, mean(SD)		136.3 (15.2)	2.8	136 (14.9)	

Neuropathy	71	Time since last healthcare visit, mean(SD)		0.8 (1.2)	0	0.6 (1)	
Neuropathy	71	Total cholesterol, mean(SD)		4.7 (1.1)	9.6	4.7 (1.1)	
Neuropathy	72	Age at diagnosis of type 2 diabetes, mean(SD)		67.6 (3)	0	67 (3)	
Neuropathy	72	BMI, mean(SD)		29.5 (5)	9.1	29.5 (5)	
Neuropathy	72	HbA1c, mean(SD)		48.3 (10.3)	2.2	48.4 (9.8)	
Neuropathy	72	HDL-cholesterol, mean(SD)		1.3 (0.4)	13.9	1.3 (0.4)	
Neuropathy	72	Ischemic heart disease, n(%)	No	34961 (78.8%)	8.9	27155 (79.6%)	
Neuropathy	72	Ischemic heart disease, n(%)	Yes	9384 (21.2%)	8.9	6966 (20.4%)	
Neuropathy	72	Self-reported physical activity, n(%)	Never	4529 (10.7%)	12.9	3554 (10.4%)	
Neuropathy	72	Self-reported physical activity, n(%)	<1 times/week	4579 (10.8%)	12.9	3535 (10.4%)	
Neuropathy	72	Self-reported physical activity, n(%)	regularly - 1-2 times/week	7686 (18.1%)	12.9	6105 (17.9%)	
Neuropathy	72	Self-reported physical activity, n(%)	regularly - 3-5 times/week	10239 (24.1%)	12.9	8314 (24.4%)	
Neuropathy	72	Self-reported physical activity, n(%)	Daily	15384 (36.3%)	12.9	12613 (37%)	
Neuropathy	72	Sex, n(%)	Males	27052 (55.6%)	0	19043 (55.8%)	n=48682 vs n=34121
Neuropathy	72	Sex, n(%)	Females	21630 (44.4%)	0	15078 (44.2%)	
Neuropathy	72	Smoking status, n(%)	No	39600 (88.2%)	7.8	30269 (88.7%)	
Neuropathy	72	Smoking status, n(%)	Yes	5308 (11.8%)	7.8	3852 (11.3%)	
Neuropathy	72	Stroke, n(%)	No	40464 (91.9%)	9.5	31514 (92.4%)	
Neuropathy	72	Stroke, n(%)	Yes	3582 (8.1%)	9.5	2607 (7.6%)	
Neuropathy	72	Systolic blood pressure, mean(SD)		136.4 (15.4)	2.9	136.1 (15.1)	
Neuropathy	72	Time since last healthcare visit, mean(SD)		0.8 (1.3)	0	0.6 (1)	
Neuropathy	72	Total cholesterol, mean(SD)		4.7 (1.1)	9.7	4.7 (1.1)	
Neuropathy	73	Age at diagnosis of type 2 diabetes, mean(SD)		68.6 (3)	0	68 (3)	

Neuropathy	73	BMI, mean(SD)		29.4 (5)	9.5	29.4 (5)	
Neuropathy	73	HbA1c, mean(SD)		48.3 (10.3)	2.3	48.3 (9.7)	
Neuropathy	73	HDL-cholesterol, mean(SD)		1.3 (0.4)	14.1	1.3 (0.4)	
Neuropathy	73	Ischemic heart disease, n(%)	No	32898 (78.2%)	9.2	25433 (78.9%)	
Neuropathy	73	Ischemic heart disease, n(%)	Yes	9187 (21.8%)	9.2	6795 (21.1%)	
Neuropathy	73	Self-reported physical activity, n(%)	Never	4433 (11%)	13.1	3444 (10.7%)	
Neuropathy	73	Self-reported physical activity, n(%)	<1 times/week	4468 (11.1%)	13.1	3436 (10.7%)	
Neuropathy	73	Self-reported physical activity, n(%)	regularly - 1-2 times/week	7313 (18.2%)	13.1	5817 (18%)	
Neuropathy	73	Self-reported physical activity, n(%)	regularly - 3-5 times/week	9629 (23.9%)	13.1	7799 (24.2%)	
Neuropathy	73	Self-reported physical activity, n(%)	Daily	14394 (35.8%)	13.1	11732 (36.4%)	
Neuropathy	73	Sex, n(%)	Males	25311 (54.6%)	0	17700 (54.9%)	n=46325 vs n=32228
Neuropathy	73	Sex, n(%)	Females	21014 (45.4%)	0	14528 (45.1%)	
Neuropathy	73	Smoking status, n(%)	No	37966 (89.2%)	8.1	28883 (89.6%)	
Neuropathy	73	Smoking status, n(%)	Yes	4603 (10.8%)	8.1	3345 (10.4%)	
Neuropathy	73	Stroke, n(%)	No	38168 (91.3%)	9.8	29557 (91.7%)	
Neuropathy	73	Stroke, n(%)	Yes	3617 (8.7%)	9.8	2671 (8.3%)	
Neuropathy	73	Systolic blood pressure, mean(SD)		136.4 (15.5)	2.9	136.1 (15.2)	
Neuropathy	73	Time since last healthcare visit, mean(SD)		0.8 (1.3)	0	0.6 (1)	
Neuropathy	73	Total cholesterol, mean(SD)		4.7 (1.1)	10	4.7 (1.1)	
Neuropathy	74	Age at diagnosis of type 2 diabetes, mean(SD)		69.6 (3)	0	69 (3)	
Neuropathy	74	BMI, mean(SD)		29.3 (5)	9.6	29.3 (5)	
Neuropathy	74	HbA1c, mean(SD)		48.3 (10.1)	2.2	48.3 (9.5)	
Neuropathy	74	HDL-cholesterol, mean(SD)		1.3 (0.4)	14.4	1.3 (0.4)	

Neuropathy	74	Ischemic heart disease, n(%)	No	30257 (77.3%)	9.4	23344 (78.1%)	
Neuropathy	74	Ischemic heart disease, n(%)	Yes	8862 (22.7%)	9.4	6532 (21.9%)	
Neuropathy	74	Self-reported physical activity, n(%)	Never	4304 (11.5%)	13.3	3307 (11.1%)	
Neuropathy	74	Self-reported physical activity, n(%)	<1 times/week	4266 (11.4%)	13.3	3240 (10.8%)	
Neuropathy	74	Self-reported physical activity, n(%)	regularly - 1-2 times/week	6887 (18.4%)	13.3	5428 (18.2%)	
Neuropathy	74	Self-reported physical activity, n(%)	regularly - 3-5 times/week	8639 (23.1%)	13.3	7012 (23.5%)	
Neuropathy	74	Self-reported physical activity, n(%)	Daily	13343 (35.6%)	13.3	10889 (36.4%)	
Neuropathy	74	Sex, n(%)	Males	23309 (54%)	0	16133 (54%)	n=43160 vs n=29876
Neuropathy	74	Sex, n(%)	Females	19851 (46%)	0	13743 (46%)	
Neuropathy	74	Smoking status, n(%)	No	35577 (89.8%)	8.2	26963 (90.2%)	
Neuropathy	74	Smoking status, n(%)	Yes	4047 (10.2%)	8.2	2913 (9.8%)	
Neuropathy	74	Stroke, n(%)	No	35284 (90.8%)	10	27239 (91.2%)	
Neuropathy	74	Stroke, n(%)	Yes	3557 (9.2%)	10	2637 (8.8%)	
Neuropathy	74	Systolic blood pressure, mean(SD)		136.5 (15.6)	3	136.2 (15.3)	
Neuropathy	74	Time since last healthcare visit, mean(SD)		0.8 (1.3)	0	0.6 (1)	
Neuropathy	74	Total cholesterol, mean(SD)		4.7 (1.1)	10.2	4.6 (1.1)	
Neuropathy	75	Age at diagnosis of type 2 diabetes, mean(SD)		70.6 (3)	0	70 (3)	
Neuropathy	75	BMI, mean(SD)		29.2 (4.9)	9.9	29.2 (4.9)	
Neuropathy	75	HbA1c, mean(SD)		48.2 (10)	2.3	48.2 (9.4)	
Neuropathy	75	HDL-cholesterol, mean(SD)		1.3 (0.4)	14.7	1.3 (0.4)	
Neuropathy	75	Ischemic heart disease, n(%)	No	27582 (76.2%)	9.4	21093 (76.8%)	
Neuropathy	75	Ischemic heart disease, n(%)	Yes	8622 (23.8%)	9.4	6359 (23.2%)	
Neuropathy	75	Self-reported physical activity, n(%)	Never	4091 (11.8%)	13.6	3153 (11.5%)	

Neuropathy	75	Self-reported physical activity, n(%)	<1 times/week	3963 (11.5%)	13.6	2983 (10.9%)	
Neuropathy	75	Self-reported physical activity, n(%)	regularly - 1-2 times/week	6456 (18.7%)	13.6	5067 (18.5%)	
Neuropathy	75	Self-reported physical activity, n(%)	regularly - 3-5 times/week	7898 (22.9%)	13.6	6423 (23.4%)	
Neuropathy	75	Self-reported physical activity, n(%)	Daily	12119 (35.1%)	13.6	9826 (35.8%)	
Neuropathy	75	Sex, n(%)	Males	21198 (53%)	0	14583 (53.1%)	n=39964 vs n=27452
Neuropathy	75	Sex, n(%)	Females	18766 (47%)	0	12869 (46.9%)	
Neuropathy	75	Smoking status, n(%)	No	33054 (90.5%)	8.6	24978 (91%)	
Neuropathy	75	Smoking status, n(%)	Yes	3476 (9.5%)	8.6	2474 (9%)	
Neuropathy	75	Stroke, n(%)	No	32429 (90.3%)	10.2	24890 (90.7%)	
Neuropathy	75	Stroke, n(%)	Yes	3468 (9.7%)	10.2	2562 (9.3%)	
Neuropathy	75	Systolic blood pressure, mean(SD)		136.7 (15.6)	3	136.4 (15.3)	
Neuropathy	75	Time since last healthcare visit, mean(SD)		0.8 (1.3)	0	0.6 (1)	
Neuropathy	75	Total cholesterol, mean(SD)		4.7 (1.1)	10.4	4.6 (1.1)	
Neuropathy	76	Age at diagnosis of type 2 diabetes, mean(SD)		71.6 (3)	0	71 (3)	
Neuropathy	76	BMI, mean(SD)		29 (4.9)	10.2	29 (4.9)	
Neuropathy	76	HbA1c, mean(SD)		48.2 (9.9)	2.4	48.2 (9.4)	
Neuropathy	76	HDL-cholesterol, mean(SD)		1.3 (0.4)	15.5	1.3 (0.4)	
Neuropathy	76	Ischemic heart disease, n(%)	No	25014 (75.1%)	9.6	18903 (75.8%)	
Neuropathy	76	Ischemic heart disease, n(%)	Yes	8281 (24.9%)	9.6	6045 (24.2%)	
Neuropathy	76	Self-reported physical activity, n(%)	Never	3835 (12.1%)	14.2	2924 (11.7%)	
Neuropathy	76	Self-reported physical activity, n(%)	<1 times/week	3638 (11.5%)	14.2	2737 (11%)	
Neuropathy	76	Self-reported physical activity, n(%)	regularly - 1-2 times/week	5896 (18.7%)	14.2	4579 (18.4%)	
Neuropathy	76	Self-reported physical activity, n(%)	regularly - 3-5 times/week	7196 (22.8%)	14.2	5809 (23.3%)	

Neuropathy	76	Self-reported physical activity, n(%)	Daily	11018 (34.9%)	14.2	8899 (35.7%)	
Neuropathy	76	Sex, n(%)	Males	19184 (52.1%)	0	13037 (52.3%)	n=36820 vs n=24948
Neuropathy	76	Sex, n(%)	Females	17636 (47.9%)	0	11911 (47.7%)	
Neuropathy	76	Smoking status, n(%)	No	30581 (91.2%)	8.9	22883 (91.7%)	
Neuropathy	76	Smoking status, n(%)	Yes	2949 (8.8%)	8.9	2065 (8.3%)	
Neuropathy	76	Stroke, n(%)	No	29594 (89.7%)	10.4	22474 (90.1%)	
Neuropathy	76	Stroke, n(%)	Yes	3397 (10.3%)	10.4	2474 (9.9%)	
Neuropathy	76	Systolic blood pressure, mean(SD)		136.7 (15.8)	3.1	136.4 (15.4)	
Neuropathy	76	Time since last healthcare visit, mean(SD)		0.8 (1.3)	0	0.7 (1)	
Neuropathy	76	Total cholesterol, mean(SD)		4.7 (1.1)	11.1	4.6 (1.1)	
Neuropathy	77	Age at diagnosis of type 2 diabetes, mean(SD)		72.6 (3)	0	72 (3)	
Neuropathy	77	BMI, mean(SD)		28.9 (4.8)	10.4	28.9 (4.8)	
Neuropathy	77	HbA1c, mean(SD)		48.1 (9.8)	2.5	48.2 (9.3)	
Neuropathy	77	HDL-cholesterol, mean(SD)		1.3 (0.4)	16.1	1.3 (0.4)	
Neuropathy	77	Ischemic heart disease, n(%)	No	22589 (73.9%)	9.8	16902 (74.5%)	
Neuropathy	77	Ischemic heart disease, n(%)	Yes	7977 (26.1%)	9.8	5797 (25.5%)	
Neuropathy	77	Self-reported physical activity, n(%)	Never	3662 (12.7%)	14.8	2764 (12.2%)	
Neuropathy	77	Self-reported physical activity, n(%)	<1 times/week	3428 (11.9%)	14.8	2558 (11.3%)	
Neuropathy	77	Self-reported physical activity, n(%)	regularly - 1-2 times/week	5385 (18.6%)	14.8	4212 (18.6%)	
Neuropathy	77	Self-reported physical activity, n(%)	regularly - 3-5 times/week	6468 (22.4%)	14.8	5191 (22.9%)	
Neuropathy	77	Self-reported physical activity, n(%)	Daily	9938 (34.4%)	14.8	7974 (35.1%)	
Neuropathy	77	Sex, n(%)	Males	17346 (51.2%)	0	11701 (51.5%)	n=33886 vs n=22699
Neuropathy	77	Sex, n(%)	Females	16540 (48.8%)	0	10998 (48.5%)	

Neuropathy	77	Smoking status, n(%)	No	28287 (91.9%)	9.1	20962 (92.3%)	
Neuropathy	77	Smoking status, n(%)	Yes	2508 (8.1%)	9.1	1737 (7.7%)	
Neuropathy	77	Stroke, n(%)	No	27069 (89.3%)	10.5	20377 (89.8%)	
Neuropathy	77	Stroke, n(%)	Yes	3251 (10.7%)	10.5	2322 (10.2%)	
Neuropathy	77	Systolic blood pressure, mean(SD)		136.8 (15.9)	3.2	136.5 (15.5)	
Neuropathy	77	Time since last healthcare visit, mean(SD)		0.8 (1.3)	0	0.7 (1)	
Neuropathy	77	Total cholesterol, mean(SD)		4.7 (1.1)	11.6	4.6 (1.1)	
Neuropathy	78	Age at diagnosis of type 2 diabetes, mean(SD)		73.6 (3)	0	73 (3)	
Neuropathy	78	BMI, mean(SD)		28.8 (4.8)	10.7	28.7 (4.8)	
Neuropathy	78	HbA1c, mean(SD)		48.2 (9.9)	2.6	48.3 (9.5)	
Neuropathy	78	HDL-cholesterol, mean(SD)		1.3 (0.4)	16.6	1.3 (0.4)	
Neuropathy	78	Ischemic heart disease, n(%)	No	20476 (73%)	10.1	15213 (73.5%)	
Neuropathy	78	Ischemic heart disease, n(%)	Yes	7583 (27%)	10.1	5481 (26.5%)	
Neuropathy	78	Self-reported physical activity, n(%)	Never	3525 (13.3%)	15.3	2622 (12.7%)	
Neuropathy	78	Self-reported physical activity, n(%)	<1 times/week	3277 (12.4%)	15.3	2444 (11.8%)	
Neuropathy	78	Self-reported physical activity, n(%)	regularly - 1-2 times/week	4886 (18.5%)	15.3	3835 (18.5%)	
Neuropathy	78	Self-reported physical activity, n(%)	regularly - 3-5 times/week	5823 (22%)	15.3	4623 (22.3%)	
Neuropathy	78	Self-reported physical activity, n(%)	Daily	8940 (33.8%)	15.3	7170 (34.6%)	
Neuropathy	78	Sex, n(%)	Males	15610 (50%)	0	10408 (50.3%)	n=31211 vs n=20694
Neuropathy	78	Sex, n(%)	Females	15601 (50%)	0	10286 (49.7%)	
Neuropathy	78	Smoking status, n(%)	No	26143 (92.4%)	9.4	19212 (92.8%)	
Neuropathy	78	Smoking status, n(%)	Yes	2145 (7.6%)	9.4	1482 (7.2%)	
Neuropathy	78	Stroke, n(%)	No	24651 (88.8%)	11	18466 (89.2%)	

Neuropathy	78	Stroke, n(%)	Yes	3122 (11.2%)	11	2228 (10.8%)	
Neuropathy	78	Systolic blood pressure, mean(SD)		137 (16)	3.3	136.7 (15.6)	
Neuropathy	78	Time since last healthcare visit, mean(SD)		0.8 (1.3)	0	0.7 (1)	
Neuropathy	78	Total cholesterol, mean(SD)		4.7 (1.1)	12	4.6 (1.1)	
Neuropathy	79	Age at diagnosis of type 2 diabetes, mean(SD)		74.6 (3)	0	73.9 (3)	
Neuropathy	79	BMI, mean(SD)		28.7 (4.8)	11.5	28.6 (4.7)	
Neuropathy	79	HbA1c, mean(SD)		48.4 (10)	2.7	48.4 (9.5)	
Neuropathy	79	HDL-cholesterol, mean(SD)		1.3 (0.4)	17.6	1.3 (0.4)	
Neuropathy	79	Ischemic heart disease, n(%)	No	18724 (72.5%)	10.7	13715 (73.1%)	
Neuropathy	79	Ischemic heart disease, n(%)	Yes	7119 (27.5%)	10.7	5054 (26.9%)	
Neuropathy	79	Self-reported physical activity, n(%)	Never	3352 (13.8%)	15.9	2405 (12.8%)	
Neuropathy	79	Self-reported physical activity, n(%)	<1 times/week	3102 (12.8%)	15.9	2242 (11.9%)	
Neuropathy	79	Self-reported physical activity, n(%)	regularly - 1-2 times/week	4618 (19%)	15.9	3570 (19%)	
Neuropathy	79	Self-reported physical activity, n(%)	regularly - 3-5 times/week	5192 (21.3%)	15.9	4148 (22.1%)	
Neuropathy	79	Self-reported physical activity, n(%)	Daily	8060 (33.1%)	15.9	6404 (34.1%)	
Neuropathy	79	Sex, n(%)	Males	14051 (48.6%)	0	9184 (48.9%)	n=28926 vs n=18769
Neuropathy	79	Sex, n(%)	Females	14875 (51.4%)	0	9585 (51.1%)	
Neuropathy	79	Smoking status, n(%)	No	24306 (93.1%)	9.7	17549 (93.5%)	
Neuropathy	79	Smoking status, n(%)	Yes	1807 (6.9%)	9.7	1220 (6.5%)	
Neuropathy	79	Stroke, n(%)	No	22529 (88%)	11.5	16643 (88.7%)	
Neuropathy	79	Stroke, n(%)	Yes	3063 (12%)	11.5	2126 (11.3%)	
Neuropathy	79	Systolic blood pressure, mean(SD)		137.1 (16.1)	3.4	136.8 (15.8)	
Neuropathy	79	Time since last healthcare visit, mean(SD)		0.9 (1.4)	0	0.7 (1.1)	

Neuropathy	79	Total cholesterol, mean(SD)		4.7 (1.1)	12.8	4.6 (1.1)	
Neuropathy	80	Age at diagnosis of type 2 diabetes, mean(SD)		75.6 (3)	0	74.9 (3)	
Neuropathy	80	BMI, mean(SD)		28.5 (4.8)	12.3	28.5 (4.7)	
Neuropathy	80	HbA1c, mean(SD)		48.4 (10.2)	2.8	48.5 (9.6)	
Neuropathy	80	HDL-cholesterol, mean(SD)		1.3 (0.4)	18.5	1.4 (0.4)	
Neuropathy	80	Ischemic heart disease, n(%)	No	16935 (71.5%)	11.1	12278 (72.3%)	
Neuropathy	80	Ischemic heart disease, n(%)	Yes	6763 (28.5%)	11.1	4706 (27.7%)	
Neuropathy	80	Self-reported physical activity, n(%)	Never	3274 (14.8%)	16.8	2328 (13.7%)	
Neuropathy	80	Self-reported physical activity, n(%)	<1 times/week	2872 (12.9%)	16.8	2092 (12.3%)	
Neuropathy	80	Self-reported physical activity, n(%)	regularly - 1-2 times/week	4320 (19.5%)	16.8	3298 (19.4%)	
Neuropathy	80	Self-reported physical activity, n(%)	regularly - 3-5 times/week	4648 (21%)	16.8	3675 (21.6%)	
Neuropathy	80	Self-reported physical activity, n(%)	Daily	7067 (31.9%)	16.8	5591 (32.9%)	
Neuropathy	80	Sex, n(%)	Males	12642 (47.4%)	0	8064 (47.5%)	n=26645 vs n=16984
Neuropathy	80	Sex, n(%)	Females	14003 (52.6%)	0	8920 (52.5%)	
Neuropathy	80	Smoking status, n(%)	No	22348 (93.6%)	10.4	15972 (94%)	
Neuropathy	80	Smoking status, n(%)	Yes	1516 (6.4%)	10.4	1012 (6%)	
Neuropathy	80	Stroke, n(%)	No	20573 (87.7%)	11.9	15015 (88.4%)	
Neuropathy	80	Stroke, n(%)	Yes	2890 (12.3%)	11.9	1969 (11.6%)	
Neuropathy	80	Systolic blood pressure, mean(SD)		137.2 (16.3)	3.6	136.9 (15.8)	
Neuropathy	80	Time since last healthcare visit, mean(SD)		0.9 (1.4)	0	0.7 (1.1)	
Neuropathy	80	Total cholesterol, mean(SD)		4.7 (1.1)	13.5	4.7 (1.1)	
Neuropathy	81	Age at diagnosis of type 2 diabetes, mean(SD)		76.6 (3)	0	75.8 (2.9)	
Neuropathy	81	BMI, mean(SD)		28.4 (4.7)	13.1	28.4 (4.6)	

Neuropathy	81	HbA1c, mean(SD)		48.6 (10.2)	3	48.7 (9.8)	
Neuropathy	81	HDL-cholesterol, mean(SD)		1.4 (0.4)	20.2	1.4 (0.4)	
Neuropathy	81	Ischemic heart disease, n(%)	No	15199 (70.5%)	11.4	10714 (71.4%)	
Neuropathy	81	Ischemic heart disease, n(%)	Yes	6355 (29.5%)	11.4	4295 (28.6%)	
Neuropathy	81	Self-reported physical activity, n(%)	Never	2991 (15%)	17.9	2073 (13.8%)	
Neuropathy	81	Self-reported physical activity, n(%)	<1 times/week	2756 (13.8%)	17.9	1950 (13%)	
Neuropathy	81	Self-reported physical activity, n(%)	regularly - 1-2 times/week	3924 (19.6%)	17.9	2965 (19.8%)	
Neuropathy	81	Self-reported physical activity, n(%)	regularly - 3-5 times/week	4087 (20.5%)	17.9	3200 (21.3%)	
Neuropathy	81	Self-reported physical activity, n(%)	Daily	6213 (31.1%)	17.9	4821 (32.1%)	
Neuropathy	81	Sex, n(%)	Males	11257 (46.3%)	0	6997 (46.6%)	n=24330 vs n=15009
Neuropathy	81	Sex, n(%)	Females	13073 (53.7%)	0	8012 (53.4%)	
Neuropathy	81	Smoking status, n(%)	No	20306 (94.1%)	11.3	14173 (94.4%)	
Neuropathy	81	Smoking status, n(%)	Yes	1279 (5.9%)	11.3	836 (5.6%)	
Neuropathy	81	Stroke, n(%)	No	18572 (87.3%)	12.5	13186 (87.9%)	
Neuropathy	81	Stroke, n(%)	Yes	2709 (12.7%)	12.5	1823 (12.1%)	
Neuropathy	81	Systolic blood pressure, mean(SD)		137.4 (16.4)	3.8	137.2 (16)	
Neuropathy	81	Time since last healthcare visit, mean(SD)		0.9 (1.4)	0	0.7 (1.1)	
Neuropathy	81	Total cholesterol, mean(SD)		4.7 (1.1)	14.7	4.7 (1.1)	
Neuropathy	82	Age at diagnosis of type 2 diabetes, mean(SD)		77.6 (3)	0	76.8 (2.9)	
Neuropathy	82	BMI, mean(SD)		28.3 (4.7)	13.6	28.3 (4.7)	
Neuropathy	82	HbA1c, mean(SD)		48.8 (10.4)	2.9	48.9 (10)	
Neuropathy	82	HDL-cholesterol, mean(SD)		1.4 (0.4)	21.4	1.4 (0.4)	
Neuropathy	82	Ischemic heart disease, n(%)	No	13473 (69.5%)	11.7	9344 (70.3%)	

Neuropathy	82	Ischemic heart disease, n(%)	Yes	5903 (30.5%)	11.7	3939 (29.7%)	
Neuropathy	82	Self-reported physical activity, n(%)	Never	2961 (16.6%)	18.7	1994 (15%)	
Neuropathy	82	Self-reported physical activity, n(%)	<1 times/week	2501 (14%)	18.7	1792 (13.5%)	
Neuropathy	82	Self-reported physical activity, n(%)	regularly - 1-2 times/week	3441 (19.3%)	18.7	2568 (19.3%)	
Neuropathy	82	Self-reported physical activity, n(%)	regularly - 3-5 times/week	3631 (20.4%)	18.7	2841 (21.4%)	
Neuropathy	82	Self-reported physical activity, n(%)	Daily	5308 (29.8%)	18.7	4088 (30.8%)	
Neuropathy	82	Sex, n(%)	Males	9956 (45.4%)	0	6051 (45.6%)	n=21946 vs n=13283
Neuropathy	82	Sex, n(%)	Females	11990 (54.6%)	0	7232 (54.4%)	
Neuropathy	82	Smoking status, n(%)	No	18303 (94.6%)	11.8	12624 (95%)	
Neuropathy	82	Smoking status, n(%)	Yes	1050 (5.4%)	11.8	659 (5%)	
Neuropathy	82	Stroke, n(%)	No	16512 (86.5%)	13	11554 (87%)	
Neuropathy	82	Stroke, n(%)	Yes	2588 (13.5%)	13	1729 (13%)	
Neuropathy	82	Systolic blood pressure, mean(SD)		137.5 (16.7)	4	137.2 (16.3)	
Neuropathy	82	Time since last healthcare visit, mean(SD)		1 (1.5)	0	0.8 (1.2)	
Neuropathy	82	Total cholesterol, mean(SD)		4.7 (1.1)	15.7	4.7 (1.1)	
Neuropathy	83	Age at diagnosis of type 2 diabetes, mean(SD)		78.6 (3)	0	77.7 (2.9)	
Neuropathy	83	BMI, mean(SD)		28.1 (4.7)	14.2	28.1 (4.7)	
Neuropathy	83	HbA1c, mean(SD)		48.9 (10.4)	3	48.9 (9.9)	
Neuropathy	83	HDL-cholesterol, mean(SD)		1.4 (0.4)	23	1.4 (0.4)	
Neuropathy	83	Ischemic heart disease, n(%)	No	11864 (68.8%)	12.2	8081 (69.7%)	
Neuropathy	83	Ischemic heart disease, n(%)	Yes	5390 (31.2%)	12.2	3510 (30.3%)	
Neuropathy	83	Self-reported physical activity, n(%)	Never	2804 (17.8%)	19.7	1885 (16.3%)	
Neuropathy	83	Self-reported physical activity, n(%)	<1 times/week	2175 (13.8%)	19.7	1525 (13.2%)	

Neuropathy	83	Self-reported physical activity, n(%)	regularly - 1-2 times/week	3122 (19.8%)	19.7	2299 (19.8%)	
Neuropathy	83	Self-reported physical activity, n(%)	regularly - 3-5 times/week	3081 (19.5%)	19.7	2353 (20.3%)	
Neuropathy	83	Self-reported physical activity, n(%)	Daily	4594 (29.1%)	19.7	3529 (30.4%)	
Neuropathy	83	Sex, n(%)	Males	8680 (44.2%)	0	5127 (44.2%)	n=19642 vs n=11591
Neuropathy	83	Sex, n(%)	Females	10962 (55.8%)	0	6464 (55.8%)	
Neuropathy	83	Smoking status, n(%)	No	16331 (95%)	12.4	11062 (95.4%)	
Neuropathy	83	Smoking status, n(%)	Yes	868 (5%)	12.4	529 (4.6%)	
Neuropathy	83	Stroke, n(%)	No	14601 (85.8%)	13.4	10003 (86.3%)	
Neuropathy	83	Stroke, n(%)	Yes	2417 (14.2%)	13.4	1588 (13.7%)	
Neuropathy	83	Systolic blood pressure, mean(SD)		137.6 (16.8)	3.9	137.1 (16.4)	
Neuropathy	83	Time since last healthcare visit, mean(SD)		1 (1.5)	0	0.8 (1.2)	
Neuropathy	83	Total cholesterol, mean(SD)		4.8 (1.1)	16.9	4.7 (1.1)	
Neuropathy	84	Age at diagnosis of type 2 diabetes, mean(SD)		79.5 (3)	0	78.6 (3)	
Neuropathy	84	BMI, mean(SD)		27.9 (4.6)	15.7	28 (4.6)	
Neuropathy	84	HbA1c, mean(SD)		49 (10.7)	3.6	49 (10)	
Neuropathy	84	HDL-cholesterol, mean(SD)		1.4 (0.4)	24.6	1.4 (0.4)	
Neuropathy	84	Ischemic heart disease, n(%)	No	10306 (67.5%)	12.6	6755 (68.1%)	
Neuropathy	84	Ischemic heart disease, n(%)	Yes	4961 (32.5%)	12.6	3162 (31.9%)	
Neuropathy	84	Self-reported physical activity, n(%)	Never	2620 (19%)	21.2	1749 (17.6%)	
Neuropathy	84	Self-reported physical activity, n(%)	<1 times/week	2051 (14.9%)	21.2	1407 (14.2%)	
Neuropathy	84	Self-reported physical activity, n(%)	regularly - 1-2 times/week	2668 (19.4%)	21.2	1899 (19.1%)	
Neuropathy	84	Self-reported physical activity, n(%)	regularly - 3-5 times/week	2629 (19.1%)	21.2	2004 (20.2%)	
Neuropathy	84	Self-reported physical activity, n(%)	Daily	3799 (27.6%)	21.2	2858 (28.8%)	

Neuropathy	84	Sex, n(%)	Males	7465 (42.7%)	0	4251 (42.9%)	n=17465 vs n=9917
Neuropathy	84	Sex, n(%)	Females	10000 (57.3%)	0	5666 (57.1%)	
Neuropathy	84	Smoking status, n(%)	No	14372 (95.3%)	13.6	9492 (95.7%)	
Neuropathy	84	Smoking status, n(%)	Yes	715 (4.7%)	13.6	425 (4.3%)	
Neuropathy	84	Stroke, n(%)	No	12850 (85.4%)	13.8	8534 (86.1%)	
Neuropathy	84	Stroke, n(%)	Yes	2198 (14.6%)	13.8	1383 (13.9%)	
Neuropathy	84	Systolic blood pressure, mean(SD)		137.7 (17.1)	4.5	137.4 (16.7)	
Neuropathy	84	Time since last healthcare visit, mean(SD)		1 (1.5)	0	0.8 (1.2)	
Neuropathy	84	Total cholesterol, mean(SD)		4.8 (1.2)	18.5	4.7 (1.1)	
Neuropathy	85	Age at diagnosis of type 2 diabetes, mean(SD)		80.5 (3)	0	79.5 (3)	
Neuropathy	85	BMI, mean(SD)		27.7 (4.6)	16.9	27.8 (4.5)	
Neuropathy	85	HbA1c, mean(SD)		49.1 (10.5)	3.8	49.1 (9.8)	
Neuropathy	85	HDL-cholesterol, mean(SD)		1.4 (0.4)	26.1	1.4 (0.4)	
Neuropathy	85	Ischemic heart disease, n(%)	No	8940 (66.6%)	13.1	5689 (67.1%)	
Neuropathy	85	Ischemic heart disease, n(%)	Yes	4479 (33.4%)	13.1	2787 (32.9%)	
Neuropathy	85	Self-reported physical activity, n(%)	Never	2396 (20%)	22.5	1569 (18.5%)	
Neuropathy	85	Self-reported physical activity, n(%)	<1 times/week	1833 (15.3%)	22.5	1245 (14.7%)	
Neuropathy	85	Self-reported physical activity, n(%)	regularly - 1-2 times/week	2346 (19.6%)	22.5	1678 (19.8%)	
Neuropathy	85	Self-reported physical activity, n(%)	regularly - 3-5 times/week	2189 (18.3%)	22.5	1634 (19.3%)	
Neuropathy	85	Self-reported physical activity, n(%)	Daily	3197 (26.7%)	22.5	2350 (27.7%)	
Neuropathy	85	Sex, n(%)	Males	6412 (41.5%)	0	3526 (41.6%)	n=15434 vs n=8476
Neuropathy	85	Sex, n(%)	Females	9022 (58.5%)	0	4950 (58.4%)	
Neuropathy	85	Smoking status, n(%)	No	12651 (95.9%)	14.6	8172 (96.4%)	

Neuropathy	85	Smoking status, n(%)	Yes	536 (4.1%)	14.6	304 (3.6%)	
Neuropathy	85	Stroke, n(%)	No	11218 (84.8%)	14.2	7225 (85.2%)	
Neuropathy	85	Stroke, n(%)	Yes	2018 (15.2%)	14.2	1251 (14.8%)	
Neuropathy	85	Systolic blood pressure, mean(SD)		138 (17.3)	4.4	137.7 (16.8)	
Neuropathy	85	Time since last healthcare visit, mean(SD)		1.1 (1.6)	0	0.9 (1.3)	
Neuropathy	85	Total cholesterol, mean(SD)		4.8 (1.2)	19.9	4.7 (1.2)	
Neuropathy	86	Age at diagnosis of type 2 diabetes, mean(SD)		81.5 (3)	0	80.5 (3)	
Neuropathy	86	BMI, mean(SD)		27.6 (4.5)	18.2	27.6 (4.5)	
Neuropathy	86	HbA1c, mean(SD)		49.3 (10.8)	4.2	49.3 (10.2)	
Neuropathy	86	HDL-cholesterol, mean(SD)		1.4 (0.4)	28.3	1.4 (0.4)	
Neuropathy	86	Ischemic heart disease, n(%)	No	7690 (66%)	13.6	4710 (66.3%)	
Neuropathy	86	Ischemic heart disease, n(%)	Yes	3965 (34%)	13.6	2394 (33.7%)	
Neuropathy	86	Self-reported physical activity, n(%)	Never	2167 (21.1%)	23.8	1374 (19.3%)	
Neuropathy	86	Self-reported physical activity, n(%)	<1 times/week	1573 (15.3%)	23.8	1061 (14.9%)	
Neuropathy	86	Self-reported physical activity, n(%)	regularly - 1-2 times/week	2024 (19.7%)	23.8	1406 (19.8%)	
Neuropathy	86	Self-reported physical activity, n(%)	regularly - 3-5 times/week	1825 (17.8%)	23.8	1329 (18.7%)	
Neuropathy	86	Self-reported physical activity, n(%)	Daily	2682 (26.1%)	23.8	1934 (27.2%)	
Neuropathy	86	Sex, n(%)	Males	5375 (39.9%)	0	2842 (40%)	n=13486 vs n=7104
Neuropathy	86	Sex, n(%)	Females	8111 (60.1%)	0	4262 (60%)	
Neuropathy	86	Smoking status, n(%)	No	10953 (96%)	15.4	6857 (96.5%)	
Neuropathy	86	Smoking status, n(%)	Yes	453 (4%)	15.4	247 (3.5%)	
Neuropathy	86	Stroke, n(%)	No	9718 (84.6%)	14.8	6028 (84.9%)	
Neuropathy	86	Stroke, n(%)	Yes	1771 (15.4%)	14.8	1076 (15.1%)	

Neuropathy	86	Systolic blood pressure, mean(SD)		138 (17.4)	4.8	137.7 (17.1)	
Neuropathy	86	Time since last healthcare visit, mean(SD)		1.1 (1.6)	0	0.9 (1.3)	
Neuropathy	86	Total cholesterol, mean(SD)		4.8 (1.2)	21.9	4.8 (1.2)	
Neuropathy	87	Age at diagnosis of type 2 diabetes, mean(SD)		82.5 (3)	0	81.4 (3)	
Neuropathy	87	BMI, mean(SD)		27.4 (4.5)	19.7	27.5 (4.4)	
Neuropathy	87	HbA1c, mean(SD)		49.4 (10.9)	4.3	49.4 (10.4)	
Neuropathy	87	HDL-cholesterol, mean(SD)		1.4 (0.4)	31.1	1.4 (0.4)	
Neuropathy	87	Ischemic heart disease, n(%)	No	6401 (64.8%)	14.6	3793 (65.3%)	
Neuropathy	87	Ischemic heart disease, n(%)	Yes	3484 (35.2%)	14.6	2017 (34.7%)	
Neuropathy	87	Self-reported physical activity, n(%)	Never	1897 (22.1%)	25.9	1187 (20.4%)	
Neuropathy	87	Self-reported physical activity, n(%)	<1 times/week	1329 (15.5%)	25.9	860 (14.8%)	
Neuropathy	87	Self-reported physical activity, n(%)	regularly - 1-2 times/week	1772 (20.7%)	25.9	1200 (20.7%)	
Neuropathy	87	Self-reported physical activity, n(%)	regularly - 3-5 times/week	1510 (17.6%)	25.9	1088 (18.7%)	
Neuropathy	87	Self-reported physical activity, n(%)	Daily	2069 (24.1%)	25.9	1475 (25.4%)	
Neuropathy	87	Sex, n(%)	Males	4477 (38.7%)	0	2241 (38.6%)	n=11580 vs n=5810
Neuropathy	87	Sex, n(%)	Females	7103 (61.3%)	0	3569 (61.4%)	
Neuropathy	87	Smoking status, n(%)	No	9282 (96.3%)	16.8	5634 (97%)	
Neuropathy	87	Smoking status, n(%)	Yes	355 (3.7%)	16.8	176 (3%)	
Neuropathy	87	Stroke, n(%)	No	8174 (84%)	16	4909 (84.5%)	
Neuropathy	87	Stroke, n(%)	Yes	1557 (16%)	16	901 (15.5%)	
Neuropathy	87	Systolic blood pressure, mean(SD)		138.2 (17.7)	5.4	137.9 (17.2)	
Neuropathy	87	Time since last healthcare visit, mean(SD)		1.2 (1.7)	0	0.9 (1.4)	
Neuropathy	87	Total cholesterol, mean(SD)		4.8 (1.2)	24.6	4.8 (1.2)	

Neuropathy	88	Age at diagnosis of type 2 diabetes, mean(SD)		83.4 (3)	0	82.2 (3)	
Neuropathy	88	BMI, mean(SD)		27.2 (4.5)	21.5	27.2 (4.4)	
Neuropathy	88	HbA1c, mean(SD)		49.6 (11)	4.4	49.7 (10.6)	
Neuropathy	88	HDL-cholesterol, mean(SD)		1.4 (0.4)	33.3	1.4 (0.4)	
Neuropathy	88	Ischemic heart disease, n(%)	No	5459 (64.4%)	14.7	3048 (64.4%)	
Neuropathy	88	Ischemic heart disease, n(%)	Yes	3017 (35.6%)	14.7	1683 (35.6%)	
Neuropathy	88	Self-reported physical activity, n(%)	Never	1770 (24.5%)	27.3	1053 (22.3%)	
Neuropathy	88	Self-reported physical activity, n(%)	<1 times/week	1177 (16.3%)	27.3	763 (16.1%)	
Neuropathy	88	Self-reported physical activity, n(%)	regularly - 1-2 times/week	1432 (19.8%)	27.3	958 (20.2%)	
Neuropathy	88	Self-reported physical activity, n(%)	regularly - 3-5 times/week	1148 (15.9%)	27.3	804 (17%)	
Neuropathy	88	Self-reported physical activity, n(%)	Daily	1699 (23.5%)	27.3	1153 (24.4%)	
Neuropathy	88	Sex, n(%)	Males	3722 (37.4%)	0	1800 (38%)	n=9942 vs n=4731
Neuropathy	88	Sex, n(%)	Females	6220 (62.6%)	0	2931 (62%)	
Neuropathy	88	Smoking status, n(%)	No	7871 (96.6%)	18	4590 (97%)	
Neuropathy	88	Smoking status, n(%)	Yes	281 (3.4%)	18	141 (3%)	
Neuropathy	88	Stroke, n(%)	No	6966 (83.7%)	16.3	3988 (84.3%)	
Neuropathy	88	Stroke, n(%)	Yes	1359 (16.3%)	16.3	743 (15.7%)	
Neuropathy	88	Systolic blood pressure, mean(SD)		138.2 (17.8)	5.8	137.6 (17.3)	
Neuropathy	88	Time since last healthcare visit, mean(SD)		1.3 (1.8)	0	1 (1.5)	
Neuropathy	88	Total cholesterol, mean(SD)		4.8 (1.2)	26.6	4.8 (1.2)	
Neuropathy	89	Age at diagnosis of type 2 diabetes, mean(SD)		84.4 (3)	0	83.1 (3)	
Neuropathy	89	BMI, mean(SD)		27 (4.5)	23.4	27 (4.4)	
Neuropathy	89	HbA1c, mean(SD)		49.7 (11)	4.7	49.6 (10.3)	

Neuropathy	89	HDL-cholesterol, mean(SD)		1.4 (0.4)	35.5	1.4 (0.4)	
Neuropathy	89	Ischemic heart disease, n(%)	No	4433 (64%)	15.5	2344 (63.8%)	
Neuropathy	89	Ischemic heart disease, n(%)	Yes	2494 (36%)	15.5	1330 (36.2%)	
Neuropathy	89	Self-reported physical activity, n(%)	Never	1463 (25.3%)	29.4	840 (22.9%)	
Neuropathy	89	Self-reported physical activity, n(%)	<1 times/week	1000 (17.3%)	29.4	625 (17%)	
Neuropathy	89	Self-reported physical activity, n(%)	regularly - 1-2 times/week	1089 (18.8%)	29.4	689 (18.8%)	
Neuropathy	89	Self-reported physical activity, n(%)	regularly - 3-5 times/week	918 (15.9%)	29.4	628 (17.1%)	
Neuropathy	89	Self-reported physical activity, n(%)	Daily	1315 (22.7%)	29.4	892 (24.3%)	
Neuropathy	89	Sex, n(%)	Males	2965 (36.2%)	0	1350 (36.7%)	n=8194 vs n=3674
Neuropathy	89	Sex, n(%)	Females	5229 (63.8%)	0	2324 (63.3%)	
Neuropathy	89	Smoking status, n(%)	No	6384 (96.8%)	19.5	3572 (97.2%)	
Neuropathy	89	Smoking status, n(%)	Yes	214 (3.2%)	19.5	102 (2.8%)	
Neuropathy	89	Stroke, n(%)	No	5720 (84.2%)	17	3108 (84.6%)	
Neuropathy	89	Stroke, n(%)	Yes	1077 (15.8%)	17	566 (15.4%)	
Neuropathy	89	Systolic blood pressure, mean(SD)		138.4 (17.9)	6.1	138.1 (17.5)	
Neuropathy	89	Time since last healthcare visit, mean(SD)		1.3 (1.8)	0	1.1 (1.5)	
Neuropathy	89	Total cholesterol, mean(SD)		4.9 (1.2)	28.6	4.9 (1.2)	
Neuropathy	90	Age at diagnosis of type 2 diabetes, mean(SD)		85.4 (3)	0	84.1 (2.9)	
Neuropathy	90	BMI, mean(SD)		26.8 (4.4)	24.7	26.7 (4.4)	
Neuropathy	90	HbA1c, mean(SD)		49.8 (11.3)	5.1	49.7 (10.8)	
Neuropathy	90	HDL-cholesterol, mean(SD)		1.4 (0.4)	37.9	1.4 (0.4)	
Neuropathy	90	Ischemic heart disease, n(%)	No	3618 (64%)	15.2	1809 (63.9%)	
Neuropathy	90	Ischemic heart disease, n(%)	Yes	2036 (36%)	15.2	1023 (36.1%)	

Neuropathy	90	Self-reported physical activity, n(%)	Never	1184 (25.7%)	31	654 (23.1%)	
Neuropathy	90	Self-reported physical activity, n(%)	<1 times/week	818 (17.8%)	31	498 (17.6%)	
Neuropathy	90	Self-reported physical activity, n(%)	regularly - 1-2 times/week	868 (18.9%)	31	518 (18.3%)	
Neuropathy	90	Self-reported physical activity, n(%)	regularly - 3-5 times/week	695 (15.1%)	31	454 (16%)	
Neuropathy	90	Self-reported physical activity, n(%)	Daily	1038 (22.6%)	31	708 (25%)	
Neuropathy	90	Sex, n(%)	Males	2329 (34.9%)	0	1045 (36.9%)	n=6671 vs n=2832
Neuropathy	90	Sex, n(%)	Females	4342 (65.1%)	0	1787 (63.1%)	
Neuropathy	90	Smoking status, n(%)	No	5087 (97%)	21.3	2768 (97.7%)	
Neuropathy	90	Smoking status, n(%)	Yes	160 (3%)	21.3	64 (2.3%)	
Neuropathy	90	Stroke, n(%)	No	4675 (84.6%)	17.2	2424 (85.6%)	
Neuropathy	90	Stroke, n(%)	Yes	850 (15.4%)	17.2	408 (14.4%)	
Neuropathy	90	Systolic blood pressure, mean(SD)		138.6 (17.8)	6.2	138.9 (17.4)	
Neuropathy	90	Time since last healthcare visit, mean(SD)		1.4 (1.8)	0	1.1 (1.5)	
Neuropathy	90	Total cholesterol, mean(SD)		4.9 (1.2)	31.3	4.9 (1.2)	
Neuropathy	91	Age at diagnosis of type 2 diabetes, mean(SD)		86.3 (3)	0	85 (2.9)	
Neuropathy	91	BMI, mean(SD)		26.6 (4.3)	26.3	26.5 (4.2)	
Neuropathy	91	HbA1c, mean(SD)		50.2 (11.5)	5.7	50.1 (10.9)	
Neuropathy	91	HDL-cholesterol, mean(SD)		1.4 (0.4)	40.2	1.4 (0.4)	
Neuropathy	91	Ischemic heart disease, n(%)	No	2888 (63.7%)	15	1393 (64.6%)	
Neuropathy	91	Ischemic heart disease, n(%)	Yes	1645 (36.3%)	15	765 (35.4%)	
Neuropathy	91	Self-reported physical activity, n(%)	Never	949 (26.8%)	33.5	505 (23.4%)	
Neuropathy	91	Self-reported physical activity, n(%)	<1 times/week	612 (17.3%)	33.5	377 (17.5%)	
Neuropathy	91	Self-reported physical activity, n(%)	regularly - 1-2 times/week	688 (19.4%)	33.5	422 (19.6%)	

Neuropathy	91	Self-reported physical activity, n(%)	regularly - 3-5 times/week	518 (14.6%)	33.5	343 (15.9%)	
Neuropathy	91	Self-reported physical activity, n(%)	Daily	780 (22%)	33.5	511 (23.7%)	
Neuropathy	91	Sex, n(%)	Males	1755 (32.9%)	0	782 (36.2%)	n=5332 vs n=2158
Neuropathy	91	Sex, n(%)	Females	3577 (67.1%)	0	1376 (63.8%)	
Neuropathy	91	Smoking status, n(%)	No	3990 (97.3%)	23.1	2111 (97.8%)	
Neuropathy	91	Smoking status, n(%)	Yes	111 (2.7%)	23.1	47 (2.2%)	
Neuropathy	91	Stroke, n(%)	No	3714 (83.8%)	16.9	1828 (84.7%)	
Neuropathy	91	Stroke, n(%)	Yes	719 (16.2%)	16.9	330 (15.3%)	
Neuropathy	91	Systolic blood pressure, mean(SD)		138.4 (17.9)	6.4	138.4 (17.4)	
Neuropathy	91	Time since last healthcare visit, mean(SD)		1.4 (1.9)	0	1.2 (1.5)	
Neuropathy	91	Total cholesterol, mean(SD)		4.9 (1.2)	33.4	4.9 (1.2)	
Neuropathy	92	Age at diagnosis of type 2 diabetes, mean(SD)		87.3 (3)	0	85.8 (2.8)	
Neuropathy	92	BMI, mean(SD)		26.5 (4.4)	28.5	26.5 (4.2)	
Neuropathy	92	HbA1c, mean(SD)		50 (11.3)	6	50 (11)	
Neuropathy	92	HDL-cholesterol, mean(SD)		1.4 (0.4)	43.6	1.4 (0.4)	
Neuropathy	92	Ischemic heart disease, n(%)	No	2207 (63.7%)	16.4	1011 (65.9%)	
Neuropathy	92	Ischemic heart disease, n(%)	Yes	1258 (36.3%)	16.4	523 (34.1%)	
Neuropathy	92	Self-reported physical activity, n(%)	Never	748 (28.3%)	36.3	380 (24.8%)	
Neuropathy	92	Self-reported physical activity, n(%)	<1 times/week	474 (17.9%)	36.3	279 (18.2%)	
Neuropathy	92	Self-reported physical activity, n(%)	regularly - 1-2 times/week	506 (19.2%)	36.3	291 (19%)	
Neuropathy	92	Self-reported physical activity, n(%)	regularly - 3-5 times/week	363 (13.7%)	36.3	229 (14.9%)	
Neuropathy	92	Self-reported physical activity, n(%)	Daily	550 (20.8%)	36.3	355 (23.1%)	
Neuropathy	92	Sex, n(%)	Males	1319 (31.8%)	0	529 (34.5%)	n=4144 vs n=1534

Neuropathy	92	Sex, n(%)	Females	2825 (68.2%)	0	1005 (65.5%)	
Neuropathy	92	Smoking status, n(%)	No	3036 (97.7%)	25	1507 (98.2%)	
Neuropathy	92	Smoking status, n(%)	Yes	72 (2.3%)	25	27 (1.8%)	
Neuropathy	92	Stroke, n(%)	No	2845 (84.1%)	18.4	1304 (85%)	
Neuropathy	92	Stroke, n(%)	Yes	536 (15.9%)	18.4	230 (15%)	
Neuropathy	92	Systolic blood pressure, mean(SD)		138.6 (17.9)	7.4	138.5 (17)	
Neuropathy	92	Time since last healthcare visit, mean(SD)		1.5 (1.9)	0	1.2 (1.7)	
Neuropathy	92	Total cholesterol, mean(SD)		4.9 (1.2)	36.7	4.9 (1.2)	
Neuropathy	93	Age at diagnosis of type 2 diabetes, mean(SD)		88.1 (3)	0	86.6 (2.8)	
Neuropathy	93	BMI, mean(SD)		26.3 (4.4)	30.5	26.3 (4.2)	
Neuropathy	93	HbA1c, mean(SD)		50 (11.6)	7.2	50.1 (11.2)	
Neuropathy	93	HDL-cholesterol, mean(SD)		1.4 (0.4)	46.5	1.4 (0.4)	
Neuropathy	93	Ischemic heart disease, n(%)	No	1642 (64.3%)	17	711 (67.6%)	
Neuropathy	93	Ischemic heart disease, n(%)	Yes	910 (35.7%)	17	341 (32.4%)	
Neuropathy	93	Self-reported physical activity, n(%)	Never	585 (31.1%)	38.8	265 (25.2%)	
Neuropathy	93	Self-reported physical activity, n(%)	<1 times/week	299 (15.9%)	38.8	183 (17.4%)	
Neuropathy	93	Self-reported physical activity, n(%)	regularly - 1-2 times/week	356 (18.9%)	38.8	202 (19.2%)	
Neuropathy	93	Self-reported physical activity, n(%)	regularly - 3-5 times/week	246 (13.1%)	38.8	149 (14.2%)	
Neuropathy	93	Self-reported physical activity, n(%)	Daily	396 (21%)	38.8	253 (24%)	
Neuropathy	93	Sex, n(%)	Males	965 (31.4%)	0	347 (33%)	n=3073 vs n=1052
Neuropathy	93	Sex, n(%)	Females	2108 (68.6%)	0	705 (67%)	
Neuropathy	93	Smoking status, n(%)	No	2214 (98.1%)	26.5	1039 (98.8%)	
Neuropathy	93	Smoking status, n(%)	Yes	44 (1.9%)	26.5	13 (1.2%)	

Neuropathy	93	Stroke, n(%)	No	2094 (84.3%)	19.2	902 (85.7%)	
Neuropathy	93	Stroke, n(%)	Yes	390 (15.7%)	19.2	150 (14.3%)	
Neuropathy	93	Systolic blood pressure, mean(SD)		138.7 (18.4)	8.3	138.4 (17.9)	
Neuropathy	93	Time since last healthcare visit, mean(SD)		1.7 (2)	0	1.3 (1.7)	
Neuropathy	93	Total cholesterol, mean(SD)		5 (1.2)	39.7	4.9 (1.2)	
Neuropathy	94	Age at diagnosis of type 2 diabetes, mean(SD)		89 (3)	0	87.4 (2.8)	
Neuropathy	94	BMI, mean(SD)		26.1 (4.2)	33.1	26.2 (4)	
Neuropathy	94	HbA1c, mean(SD)		50.1 (11.3)	7.8	50.4 (11.6)	
Neuropathy	94	HDL-cholesterol, mean(SD)		1.4 (0.4)	49.1	1.4 (0.4)	
Neuropathy	94	Ischemic heart disease, n(%)	No	1187 (64.2%)	17.8	470 (65.4%)	
Neuropathy	94	Ischemic heart disease, n(%)	Yes	661 (35.8%)	17.8	249 (34.6%)	
Neuropathy	94	Self-reported physical activity, n(%)	Never	435 (32.9%)	41.2	190 (26.4%)	
Neuropathy	94	Self-reported physical activity, n(%)	<1 times/week	214 (16.2%)	41.2	125 (17.4%)	
Neuropathy	94	Self-reported physical activity, n(%)	regularly - 1-2 times/week	240 (18.1%)	41.2	135 (18.8%)	
Neuropathy	94	Self-reported physical activity, n(%)	regularly - 3-5 times/week	175 (13.2%)	41.2	115 (16%)	
Neuropathy	94	Self-reported physical activity, n(%)	Daily	259 (19.6%)	41.2	154 (21.4%)	
Neuropathy	94	Sex, n(%)	Males	663 (29.5%)	0	230 (32%)	n=2249 vs n=719
Neuropathy	94	Sex, n(%)	Females	1586 (70.5%)	0	489 (68%)	
Neuropathy	94	Smoking status, n(%)	No	1569 (97.8%)	28.6	709 (98.6%)	
Neuropathy	94	Smoking status, n(%)	Yes	36 (2.2%)	28.6	10 (1.4%)	
Neuropathy	94	Stroke, n(%)	No	1510 (83.9%)	20	621 (86.4%)	
Neuropathy	94	Stroke, n(%)	Yes	289 (16.1%)	20	98 (13.6%)	
Neuropathy	94	Systolic blood pressure, mean(SD)		138.8 (18.4)	8.8	138.4 (17.9)	

Neuropathy	94	Time since last healthcare visit, mean(SD)		1.8 (2.1)	0	1.4 (1.8)	
Neuropathy	94	Total cholesterol, mean(SD)		5 (1.2)	42.6	5 (1.2)	
Neuropathy	95	Age at diagnosis of type 2 diabetes, mean(SD)		89.9 (3.1)	0	88 (2.8)	
Neuropathy	95	BMI, mean(SD)		25.9 (4.2)	34.6	25.9 (4)	
Neuropathy	95	HbA1c, mean(SD)		50 (10.8)	8.9	49.9 (10.9)	
Neuropathy	95	HDL-cholesterol, mean(SD)		1.4 (0.4)	52.6	1.4 (0.4)	
Neuropathy	95	Ischemic heart disease, n(%)	No	873 (65.3%)	18.3	314 (66.5%)	
Neuropathy	95	Ischemic heart disease, n(%)	Yes	463 (34.7%)	18.3	158 (33.5%)	
Neuropathy	95	Self-reported physical activity, n(%)	Never	336 (36.8%)	44.1	136 (28.8%)	
Neuropathy	95	Self-reported physical activity, n(%)	<1 times/week	124 (13.6%)	44.1	68 (14.4%)	
Neuropathy	95	Self-reported physical activity, n(%)	regularly - 1-2 times/week	164 (17.9%)	44.1	91 (19.3%)	
Neuropathy	95	Self-reported physical activity, n(%)	regularly - 3-5 times/week	117 (12.8%)	44.1	71 (15%)	
Neuropathy	95	Self-reported physical activity, n(%)	Daily	173 (18.9%)	44.1	106 (22.5%)	
Neuropathy	95	Sex, n(%)	Males	446 (27.3%)	0	144 (30.5%)	n=1636 vs n=472
Neuropathy	95	Sex, n(%)	Females	1190 (72.7%)	0	328 (69.5%)	
Neuropathy	95	Smoking status, n(%)	No	1109 (97.9%)	30.7	465 (98.5%)	
Neuropathy	95	Smoking status, n(%)	Yes	24 (2.1%)	30.7	7 (1.5%)	
Neuropathy	95	Stroke, n(%)	No	1084 (83.3%)	20.5	404 (85.6%)	
Neuropathy	95	Stroke, n(%)	Yes	217 (16.7%)	20.5	68 (14.4%)	
Neuropathy	95	Systolic blood pressure, mean(SD)		138.3 (18.8)	9.2	137.4 (19.2)	
Neuropathy	95	Time since last healthcare visit, mean(SD)		1.9 (2.2)	0	1.6 (2)	
Neuropathy	95	Total cholesterol, mean(SD)		5 (1.2)	45.6	5 (1.2)	
Neuropathy	96	Age at diagnosis of type 2 diabetes, mean(SD)		90.9 (3)	0	89.2 (2.9)	

Neuropathy	96	BMI, mean(SD)		25.7 (4.1)	37.7	25.5 (3.8)	
Neuropathy	96	HbA1c, mean(SD)		50.3 (11.2)	9.8	50.6 (11.3)	
Neuropathy	96	HDL-cholesterol, mean(SD)		1.4 (0.4)	55.5	1.4 (0.4)	
Neuropathy	96	Ischemic heart disease, n(%)	No	578 (64.6%)	19.7	183 (64.2%)	
Neuropathy	96	Ischemic heart disease, n(%)	Yes	317 (35.4%)	19.7	102 (35.8%)	
Neuropathy	96	Self-reported physical activity, n(%)	Never	226 (39%)	48.1	92 (32.3%)	
Neuropathy	96	Self-reported physical activity, n(%)	<1 times/week	77 (13.3%)	48.1	44 (15.4%)	
Neuropathy	96	Self-reported physical activity, n(%)	regularly - 1-2 times/week	104 (18%)	48.1	56 (19.6%)	
Neuropathy	96	Self-reported physical activity, n(%)	regularly - 3-5 times/week	75 (13%)	48.1	40 (14%)	
Neuropathy	96	Self-reported physical activity, n(%)	Daily	97 (16.8%)	48.1	53 (18.6%)	
Neuropathy	96	Sex, n(%)	Males	287 (25.7%)	0	75 (26.3%)	n=1115 vs n=285
Neuropathy	96	Sex, n(%)	Females	828 (74.3%)	0	210 (73.7%)	
Neuropathy	96	Smoking status, n(%)	No	713 (97.9%)	34.7	280 (98.2%)	
Neuropathy	96	Smoking status, n(%)	Yes	15 (2.1%)	34.7	5 (1.8%)	
Neuropathy	96	Stroke, n(%)	No	733 (83.7%)	21.4	248 (87%)	
Neuropathy	96	Stroke, n(%)	Yes	143 (16.3%)	21.4	37 (13%)	
Neuropathy	96	Systolic blood pressure, mean(SD)		138.6 (18.5)	10.1	137.5 (18.1)	
Neuropathy	96	Time since last healthcare visit, mean(SD)		2 (2.3)	0	1.6 (2.1)	
Neuropathy	96	Total cholesterol, mean(SD)		5.1 (1.2)	48.5	5 (1.2)	
Neuropathy	97	Age at diagnosis of type 2 diabetes, mean(SD)		91.9 (3)	0	90.2 (2.8)	
Neuropathy	97	BMI, mean(SD)		25.8 (4.2)	39.6	25.6 (4)	
Neuropathy	97	HbA1c, mean(SD)		49.8 (10.5)	9.5	50.2 (10.9)	
Neuropathy	97	HDL-cholesterol, mean(SD)		1.4 (0.4)	59.3	1.4 (0.4)	

Neuropathy	97	Ischemic heart disease, n(%)	No	374 (62.8%)	18.8	111 (63.8%)	
Neuropathy	97	Ischemic heart disease, n(%)	Yes	222 (37.2%)	18.8	63 (36.2%)	
Neuropathy	97	Self-reported physical activity, n(%)	Never	159 (42.2%)	48.6	69 (39.7%)	
Neuropathy	97	Self-reported physical activity, n(%)	<1 times/week	51 (13.5%)	48.6	22 (12.6%)	
Neuropathy	97	Self-reported physical activity, n(%)	regularly - 1-2 times/week	65 (17.2%)	48.6	31 (17.8%)	
Neuropathy	97	Self-reported physical activity, n(%)	regularly - 3-5 times/week	45 (11.9%)	48.6	26 (14.9%)	
Neuropathy	97	Self-reported physical activity, n(%)	Daily	57 (15.1%)	48.6	26 (14.9%)	
Neuropathy	97	Sex, n(%)	Males	174 (23.7%)	0	47 (27%)	n=734 vs n=174
Neuropathy	97	Sex, n(%)	Females	560 (76.3%)	0	127 (73%)	
Neuropathy	97	Smoking status, n(%)	No	466 (97.9%)	35.1	170 (97.7%)	
Neuropathy	97	Smoking status, n(%)	Yes	10 (2.1%)	35.1	4 (2.3%)	
Neuropathy	97	Stroke, n(%)	No	481 (82.5%)	20.6	143 (82.2%)	
Neuropathy	97	Stroke, n(%)	Yes	102 (17.5%)	20.6	31 (17.8%)	
Neuropathy	97	Systolic blood pressure, mean(SD)		139.1 (18.6)	8.3	136.7 (19.2)	
Neuropathy	97	Time since last healthcare visit, mean(SD)		2 (2.2)	0	1.6 (1.9)	
Neuropathy	97	Total cholesterol, mean(SD)		5.1 (1.1)	51.8	5.1 (1.1)	
Neuropathy	98	Age at diagnosis of type 2 diabetes, mean(SD)		92.7 (3)	0	91.1 (3)	
Neuropathy	98	BMI, mean(SD)		25.4 (4)	40.5	25.2 (3.7)	
Neuropathy	98	HbA1c, mean(SD)		49.3 (10.9)	10.9	49.7 (10.6)	
Neuropathy	98	HDL-cholesterol, mean(SD)		1.4 (0.5)	58	1.4 (0.5)	
Neuropathy	98	Ischemic heart disease, n(%)	No	241 (62.3%)	17.5	75 (65.8%)	
Neuropathy	98	Ischemic heart disease, n(%)	Yes	146 (37.7%)	17.5	39 (34.2%)	
Neuropathy	98	Self-reported physical activity, n(%)	Never	107 (45.1%)	49.5	45 (39.5%)	

Neuropathy	98	Self-reported physical activity, n(%)	<1 times/week	31 (13.1%)	49.5	16 (14%)	
Neuropathy	98	Self-reported physical activity, n(%)	regularly - 1-2 times/week	41 (17.3%)	49.5	22 (19.3%)	
Neuropathy	98	Self-reported physical activity, n(%)	regularly - 3-5 times/week	27 (11.4%)	49.5	12 (10.5%)	
Neuropathy	98	Self-reported physical activity, n(%)	Daily	31 (13.1%)	49.5	19 (16.7%)	
Neuropathy	98	Sex, n(%)	Males	95 (20.3%)	0	30 (26.3%)	n=469 vs n=114
Neuropathy	98	Sex, n(%)	Females	374 (79.7%)	0	84 (73.7%)	
Neuropathy	98	Smoking status, n(%)	No	296 (98.3%)	35.8	113 (99.1%)	
Neuropathy	98	Smoking status, n(%)	Yes	5 (1.7%)	35.8	1 (0.9%)	
Neuropathy	98	Stroke, n(%)	No	311 (82.1%)	19.2	87 (76.3%)	
Neuropathy	98	Stroke, n(%)	Yes	68 (17.9%)	19.2	27 (23.7%)	
Neuropathy	98	Systolic blood pressure, mean(SD)		138.6 (18.8)	8.5	135.9 (18.5)	
Neuropathy	98	Time since last healthcare visit, mean(SD)		2.2 (2.3)	0	2 (2.2)	
Neuropathy	98	Total cholesterol, mean(SD)		5.2 (1.2)	49.9	5 (1.2)	
Neuropathy	99	Age at diagnosis of type 2 diabetes, mean(SD)		93.7 (3.1)	0	91.7 (3)	
Neuropathy	99	BMI, mean(SD)		25.7 (4.2)	42.3	25.1 (4)	
Neuropathy	99	HbA1c, mean(SD)		49.5 (11.8)	11.8	50.7 (12.1)	
Neuropathy	99	HDL-cholesterol, mean(SD)		1.4 (0.4)	60.6	1.4 (0.4)	
Neuropathy	99	Ischemic heart disease, n(%)	No	141 (61.6%)	17.9	35 (61.4%)	
Neuropathy	99	Ischemic heart disease, n(%)	Yes	88 (38.4%)	17.9	22 (38.6%)	
Neuropathy	99	Self-reported physical activity, n(%)	Never	66 (49.6%)	52.3	21 (36.8%)	
Neuropathy	99	Self-reported physical activity, n(%)	<1 times/week	16 (12%)	52.3	10 (17.5%)	
Neuropathy	99	Self-reported physical activity, n(%)	regularly - 1-2 times/week	20 (15%)	52.3	9 (15.8%)	
Neuropathy	99	Self-reported physical activity, n(%)	regularly - 3-5 times/week	12 (9%)	52.3	6 (10.5%)	

Neuropathy	99	Self-reported physical activity, n(%)	Daily	19 (14.3%)	52.3	11 (19.3%)	
Neuropathy	99	Sex, n(%)	Males	70 (25.1%)	0	17 (29.8%)	n=279 vs n=57
Neuropathy	99	Sex, n(%)	Females	209 (74.9%)	0	40 (70.2%)	
Neuropathy	99	Smoking status, n(%)	No	173 (97.7%)	36.6	57 (100%)	
Neuropathy	99	Smoking status, n(%)	Yes	4 (2.3%)	36.6	0 (0%)	
Neuropathy	99	Stroke, n(%)	No	192 (84.2%)	18.3	46 (80.7%)	
Neuropathy	99	Stroke, n(%)	Yes	36 (15.8%)	18.3	11 (19.3%)	
Neuropathy	99	Systolic blood pressure, mean(SD)		139.1 (18.6)	9.7	137.4 (17.1)	
Neuropathy	99	Time since last healthcare visit, mean(SD)		2.2 (2.4)	0	1.8 (2.2)	
Neuropathy	99	Total cholesterol, mean(SD)		5.4 (1.3)	54.1	5.3 (1.4)	
Neuropathy	100	Age at diagnosis of type 2 diabetes, mean(SD)		94.6 (2.9)	0	92.7 (2.6)	
Neuropathy	100	BMI, mean(SD)		25.4 (4.1)	50.7	24.8 (3.5)	
Neuropathy	100	HbA1c, mean(SD)		49.7 (11.7)	15.8	51 (13.2)	
Neuropathy	100	HDL-cholesterol, mean(SD)		1.4 (0.4)	69.7	1.4 (0.4)	
Neuropathy	100	Ischemic heart disease, n(%)	No	79 (63.2%)	17.8	17 (70.8%)	
Neuropathy	100	Ischemic heart disease, n(%)	Yes	46 (36.8%)	17.8	7 (29.2%)	
Neuropathy	100	Self-reported physical activity, n(%)	Never	37 (53.6%)	54.6	7 (29.2%)	
Neuropathy	100	Self-reported physical activity, n(%)	<1 times/week	10 (14.5%)	54.6	6 (25%)	
Neuropathy	100	Self-reported physical activity, n(%)	regularly - 1-2 times/week	10 (14.5%)	54.6	5 (20.8%)	
Neuropathy	100	Self-reported physical activity, n(%)	regularly - 3-5 times/week	6 (8.7%)	54.6	3 (12.5%)	
Neuropathy	100	Self-reported physical activity, n(%)	Daily	6 (8.7%)	54.6	3 (12.5%)	
Neuropathy	100	Sex, n(%)	Males	37 (24.3%)	0	7 (29.2%)	n=152 vs n=24
Neuropathy	100	Sex, n(%)	Females	115 (75.7%)	0	17 (70.8%)	

Neuropathy	100	Smoking status, n(%)	No	88 (96.7%)	40.1	24 (100%)	
Neuropathy	100	Smoking status, n(%)	Yes	3 (3.3%)	40.1	0 (0%)	
Neuropathy	100	Stroke, n(%)	No	107 (87.7%)	19.7	22 (91.7%)	
Neuropathy	100	Stroke, n(%)	Yes	15 (12.3%)	19.7	2 (8.3%)	
Neuropathy	100	Systolic blood pressure, mean(SD)		138.5 (18.3)	11.8	139 (17.7)	
Neuropathy	100	Time since last healthcare visit, mean(SD)		2.5 (2.5)	0	1.7 (2.5)	
Neuropathy	100	Total cholesterol, mean(SD)		5.3 (1.1)	63.8	5 (1.1)	
Ulcer	19	Age at diagnosis of type 2 diabetes, mean(SD)		18.5 (0.3)	0	18.5 (0.3)	
Ulcer	19	BMI, mean(SD)		34.2 (6.9)	17.7	35.1 (7.2)	
Ulcer	19	HbA1c, mean(SD)		55.1 (19.7)	19.4	57.1 (19)	
Ulcer	19	HDL-cholesterol, mean(SD)		0.9 (0.2)	46.8	0.9 (0.2)	
Ulcer	19	Ischemic heart disease, n(%)	No	44 (100%)	29	17 (100%)	
Ulcer	19	Neuropathy, n(%)	No	62 (100%)	0	17 (100%)	
Ulcer	19	Self-reported physical activity, n(%)	Never	10 (25%)	35.5	5 (29.4%)	
Ulcer	19	Self-reported physical activity, n(%)	<1 times/week	8 (20%)	35.5	2 (11.8%)	
Ulcer	19	Self-reported physical activity, n(%)	regularly - 1-2 times/week	8 (20%)	35.5	4 (23.5%)	
Ulcer	19	Self-reported physical activity, n(%)	regularly - 3-5 times/week	8 (20%)	35.5	3 (17.6%)	
Ulcer	19	Self-reported physical activity, n(%)	Daily	6 (15%)	35.5	3 (17.6%)	
Ulcer	19	Sex, n(%)	Males	22 (35.5%)	0	6 (35.3%)	n=62 vs n=17
Ulcer	19	Sex, n(%)	Females	40 (64.5%)	0	11 (64.7%)	
Ulcer	19	Smoking status, n(%)	No	28 (68.3%)	33.9	10 (58.8%)	
Ulcer	19	Smoking status, n(%)	Yes	13 (31.7%)	33.9	7 (41.2%)	
Ulcer	19	Stroke, n(%)	No	43 (100%)	30.6	17 (100%)	

Ulcer	19	Systolic blood pressure, mean(SD)		124.6 (10.3)	22.6	124.1 (8.5)	
Ulcer	19	Time since last healthcare visit, mean(SD)		0.3 (0.2)	0	0.1 (0.1)	
Ulcer	19	Total cholesterol, mean(SD)		4.8 (1.1)	45.2	5 (1.1)	
Ulcer	20	Age at diagnosis of type 2 diabetes, mean(SD)		19.2 (1.9)	0	19 (0.6)	
Ulcer	20	BMI, mean(SD)		35.6 (8)	22.2	36.8 (8.2)	
Ulcer	20	HbA1c, mean(SD)		55.6 (21.2)	15.3	58.1 (20.5)	
Ulcer	20	HDL-cholesterol, mean(SD)		1 (0.3)	42.4	1 (0.3)	
Ulcer	20	Ischemic heart disease, n(%)	No	118 (100%)	18.1	51 (100%)	
Ulcer	20	Neuropathy, n(%)	No	140 (97.2%)	0	49 (96.1%)	
Ulcer	20	Neuropathy, n(%)	Yes	4 (2.8%)	0	2 (3.9%)	
Ulcer	20	Self-reported physical activity, n(%)	Never	16 (16.7%)	33.3	12 (23.5%)	
Ulcer	20	Self-reported physical activity, n(%)	<1 times/week	13 (13.5%)	33.3	6 (11.8%)	
Ulcer	20	Self-reported physical activity, n(%)	regularly - 1-2 times/week	21 (21.9%)	33.3	12 (23.5%)	
Ulcer	20	Self-reported physical activity, n(%)	regularly - 3-5 times/week	28 (29.2%)	33.3	14 (27.5%)	
Ulcer	20	Self-reported physical activity, n(%)	Daily	18 (18.8%)	33.3	7 (13.7%)	
Ulcer	20	Sex, n(%)	Males	67 (46.9%)	0.7	25 (49%)	n=144 vs n=51
Ulcer	20	Sex, n(%)	Females	76 (53.1%)	0.7	26 (51%)	
Ulcer	20	Smoking status, n(%)	No	73 (73%)	30.6	35 (68.6%)	
Ulcer	20	Smoking status, n(%)	Yes	27 (27%)	30.6	16 (31.4%)	
Ulcer	20	Stroke, n(%)	No	117 (100%)	18.8	51 (100%)	
Ulcer	20	Systolic blood pressure, mean(SD)		123.7 (11.8)	21.5	125.2 (12.9)	
Ulcer	20	Time since last healthcare visit, mean(SD)		0.3 (1.8)	0	0.3 (0.3)	
Ulcer	20	Total cholesterol, mean(SD)		4.7 (1)	37.5	4.8 (1.1)	

Ulcer	21	Age at diagnosis of type 2 diabetes, mean(SD)		19.7 (1.6)	0	19.5 (0.8)	
Ulcer	21	BMI, mean(SD)		35.7 (7.6)	20.3	35.3 (7.4)	
Ulcer	21	HbA1c, mean(SD)		55.1 (20.2)	12.4	54.6 (20.8)	
Ulcer	21	HDL-cholesterol, mean(SD)		1 (0.3)	32	1 (0.3)	
Ulcer	21	Ischemic heart disease, n(%)	No	194 (99%)	18.7	110 (100%)	
Ulcer	21	Ischemic heart disease, n(%)	Yes	2 (1%)	18.7	0 (0%)	
Ulcer	21	Neuropathy, n(%)	No	236 (97.9%)	0	106 (96.4%)	
Ulcer	21	Neuropathy, n(%)	Yes	5 (2.1%)	0	4 (3.6%)	
Ulcer	21	Self-reported physical activity, n(%)	Never	31 (18.1%)	29	20 (18.2%)	
Ulcer	21	Self-reported physical activity, n(%)	<1 times/week	33 (19.3%)	29	21 (19.1%)	
Ulcer	21	Self-reported physical activity, n(%)	regularly - 1-2 times/week	33 (19.3%)	29	22 (20%)	
Ulcer	21	Self-reported physical activity, n(%)	regularly - 3-5 times/week	34 (19.9%)	29	23 (20.9%)	
Ulcer	21	Self-reported physical activity, n(%)	Daily	40 (23.4%)	29	24 (21.8%)	
Ulcer	21	Sex, n(%)	Males	118 (49.2%)	0.4	57 (51.8%)	n=241 vs n=110
Ulcer	21	Sex, n(%)	Females	122 (50.8%)	0.4	53 (48.2%)	
Ulcer	21	Smoking status, n(%)	No	133 (75.1%)	26.6	80 (72.7%)	
Ulcer	21	Smoking status, n(%)	Yes	44 (24.9%)	26.6	30 (27.3%)	
Ulcer	21	Stroke, n(%)	No	194 (100%)	19.5	110 (100%)	
Ulcer	21	Systolic blood pressure, mean(SD)		124 (13)	16.2	123.3 (12.9)	
Ulcer	21	Time since last healthcare visit, mean(SD)		0.5 (1.4)	0	0.5 (0.5)	
Ulcer	21	Total cholesterol, mean(SD)		4.8 (1)	28.2	4.8 (1)	
Ulcer	22	Age at diagnosis of type 2 diabetes, mean(SD)		20.3 (1.5)	0	20.1 (1)	
Ulcer	22	BMI, mean(SD)		35.6 (7.3)	18.9	35.5 (6.9)	

Ulcer	22	HbA1c, mean(SD)		55.9 (21.9)	10.7	55.7 (21.4)	
Ulcer	22	HDL-cholesterol, mean(SD)		1 (0.3)	27.9	1 (0.4)	
Ulcer	22	Ischemic heart disease, n(%)	No	298 (99%)	17.5	185 (99.5%)	
Ulcer	22	Ischemic heart disease, n(%)	Yes	3 (1%)	17.5	1 (0.5%)	
Ulcer	22	Neuropathy, n(%)	No	356 (97.5%)	0	181 (97.3%)	
Ulcer	22	Neuropathy, n(%)	Yes	9 (2.5%)	0	5 (2.7%)	
Ulcer	22	Self-reported physical activity, n(%)	Never	49 (18.8%)	28.5	42 (22.6%)	
Ulcer	22	Self-reported physical activity, n(%)	<1 times/week	46 (17.6%)	28.5	30 (16.1%)	
Ulcer	22	Self-reported physical activity, n(%)	regularly - 1-2 times/week	45 (17.2%)	28.5	29 (15.6%)	
Ulcer	22	Self-reported physical activity, n(%)	regularly - 3-5 times/week	53 (20.3%)	28.5	37 (19.9%)	
Ulcer	22	Self-reported physical activity, n(%)	Daily	68 (26.1%)	28.5	48 (25.8%)	
Ulcer	22	Sex, n(%)	Males	168 (46.2%)	0.3	86 (46.2%)	n=365 vs n=186
Ulcer	22	Sex, n(%)	Females	196 (53.8%)	0.3	100 (53.8%)	
Ulcer	22	Smoking status, n(%)	No	203 (75.2%)	26	143 (76.9%)	
Ulcer	22	Smoking status, n(%)	Yes	67 (24.8%)	26	43 (23.1%)	
Ulcer	22	Stroke, n(%)	No	296 (99.7%)	18.6	186 (100%)	
Ulcer	22	Stroke, n(%)	Yes	1 (0.3%)	18.6	0 (0%)	
Ulcer	22	Systolic blood pressure, mean(SD)		124.3 (13.7)	15.3	125.1 (13)	
Ulcer	22	Time since last healthcare visit, mean(SD)		0.6 (1.3)	0	0.5 (0.6)	
Ulcer	22	Total cholesterol, mean(SD)		4.9 (1.1)	24.9	4.9 (1.1)	
Ulcer	23	Age at diagnosis of type 2 diabetes, mean(SD)		21 (1.6)	0	20.7 (1.3)	
Ulcer	23	BMI, mean(SD)		35.8 (7.6)	18.9	35.9 (7)	
Ulcer	23	HbA1c, mean(SD)		55 (19.3)	9.1	55.5 (19.9)	

Ulcer	23	HDL-cholesterol, mean(SD)		1 (0.3)	28	1 (0.4)	
Ulcer	23	Ischemic heart disease, n(%)	No	417 (99.3%)	18.9	275 (100%)	
Ulcer	23	Ischemic heart disease, n(%)	Yes	3 (0.7%)	18.9	0 (0%)	
Ulcer	23	Neuropathy, n(%)	No	505 (97.5%)	0	266 (96.7%)	
Ulcer	23	Neuropathy, n(%)	Yes	13 (2.5%)	0	9 (3.3%)	
Ulcer	23	Self-reported physical activity, n(%)	Never	71 (18.6%)	26.4	54 (19.6%)	
Ulcer	23	Self-reported physical activity, n(%)	<1 times/week	63 (16.5%)	26.4	47 (17.1%)	
Ulcer	23	Self-reported physical activity, n(%)	regularly - 1-2 times/week	69 (18.1%)	26.4	51 (18.5%)	
Ulcer	23	Self-reported physical activity, n(%)	regularly - 3-5 times/week	86 (22.6%)	26.4	63 (22.9%)	
Ulcer	23	Self-reported physical activity, n(%)	Daily	92 (24.1%)	26.4	60 (21.8%)	
Ulcer	23	Sex, n(%)	Males	246 (47.6%)	0.2	135 (49.1%)	n=518 vs n=275
Ulcer	23	Sex, n(%)	Females	271 (52.4%)	0.2	140 (50.9%)	
Ulcer	23	Smoking status, n(%)	No	288 (72.5%)	23.4	205 (74.5%)	
Ulcer	23	Smoking status, n(%)	Yes	109 (27.5%)	23.4	70 (25.5%)	
Ulcer	23	Stroke, n(%)	No	415 (99.8%)	19.7	275 (100%)	
Ulcer	23	Stroke, n(%)	Yes	1 (0.2%)	19.7	0 (0%)	
Ulcer	23	Systolic blood pressure, mean(SD)		124.6 (13.6)	14.9	124.7 (13.6)	
Ulcer	23	Time since last healthcare visit, mean(SD)		0.7 (1.3)	0	0.6 (0.8)	
Ulcer	23	Total cholesterol, mean(SD)		4.8 (1.1)	24.1	4.8 (1.1)	
Ulcer	24	Age at diagnosis of type 2 diabetes, mean(SD)		21.8 (1.9)	0	21.4 (1.6)	
Ulcer	24	BMI, mean(SD)		35.7 (7.5)	17	36 (7)	
Ulcer	24	HbA1c, mean(SD)		55.1 (20.8)	8.8	54.2 (19.4)	
Ulcer	24	HDL-cholesterol, mean(SD)		1 (0.4)	28	1 (0.4)	

Ulcer	24	Ischemic heart disease, n(%)	No	599 (99.2%)	15.9	386 (100%)	
Ulcer	24	Ischemic heart disease, n(%)	Yes	5 (0.8%)	15.9	0 (0%)	
Ulcer	24	Neuropathy, n(%)	No	701 (97.6%)	0	372 (96.4%)	
Ulcer	24	Neuropathy, n(%)	Yes	17 (2.4%)	0	14 (3.6%)	
Ulcer	24	Self-reported physical activity, n(%)	Never	77 (14.3%)	24.9	52 (13.5%)	
Ulcer	24	Self-reported physical activity, n(%)	<1 times/week	90 (16.7%)	24.9	65 (16.8%)	
Ulcer	24	Self-reported physical activity, n(%)	regularly - 1-2 times/week	113 (21%)	24.9	84 (21.8%)	
Ulcer	24	Self-reported physical activity, n(%)	regularly - 3-5 times/week	130 (24.1%)	24.9	96 (24.9%)	
Ulcer	24	Self-reported physical activity, n(%)	Daily	129 (23.9%)	24.9	89 (23.1%)	
Ulcer	24	Sex, n(%)	Males	333 (46.5%)	0.3	181 (46.9%)	n=718 vs n=386
Ulcer	24	Sex, n(%)	Females	383 (53.5%)	0.3	205 (53.1%)	
Ulcer	24	Smoking status, n(%)	No	421 (72.8%)	19.5	286 (74.1%)	
Ulcer	24	Smoking status, n(%)	Yes	157 (27.2%)	19.5	100 (25.9%)	
Ulcer	24	Stroke, n(%)	No	596 (99.5%)	16.6	384 (99.5%)	
Ulcer	24	Stroke, n(%)	Yes	3 (0.5%)	16.6	2 (0.5%)	
Ulcer	24	Systolic blood pressure, mean(SD)		124.3 (13.6)	13	124.5 (13.5)	
Ulcer	24	Time since last healthcare visit, mean(SD)		0.8 (1.6)	0	0.7 (0.9)	
Ulcer	24	Total cholesterol, mean(SD)		4.9 (1.3)	25.2	4.8 (1.2)	
Ulcer	25	Age at diagnosis of type 2 diabetes, mean(SD)		22.4 (2)	0	22.1 (1.7)	
Ulcer	25	BMI, mean(SD)		35.7 (7.8)	15.6	35.4 (7.5)	
Ulcer	25	HbA1c, mean(SD)		53.6 (20.1)	7.7	53 (19.4)	
Ulcer	25	HDL-cholesterol, mean(SD)		1 (0.4)	26.5	1 (0.4)	
Ulcer	25	Ischemic heart disease, n(%)	No	757 (99.3%)	14.9	486 (100%)	

Ulcer	25	Ischemic heart disease, n(%)	Yes	5 (0.7%)	14.9	0 (0%)	
Ulcer	25	Neuropathy, n(%)	No	872 (97.4%)	0	467 (96.1%)	
Ulcer	25	Neuropathy, n(%)	Yes	23 (2.6%)	0	19 (3.9%)	
Ulcer	25	Self-reported physical activity, n(%)	Never	107 (15.6%)	23.4	72 (14.8%)	
Ulcer	25	Self-reported physical activity, n(%)	<1 times/week	108 (15.7%)	23.4	81 (16.7%)	
Ulcer	25	Self-reported physical activity, n(%)	regularly - 1-2 times/week	136 (19.8%)	23.4	93 (19.1%)	
Ulcer	25	Self-reported physical activity, n(%)	regularly - 3-5 times/week	165 (24.1%)	23.4	125 (25.7%)	
Ulcer	25	Self-reported physical activity, n(%)	Daily	170 (24.8%)	23.4	115 (23.7%)	
Ulcer	25	Sex, n(%)	Males	419 (46.9%)	0.2	235 (48.4%)	n=895 vs n=486
Ulcer	25	Sex, n(%)	Females	474 (53.1%)	0.2	251 (51.6%)	
Ulcer	25	Smoking status, n(%)	No	544 (74.7%)	18.7	370 (76.1%)	
Ulcer	25	Smoking status, n(%)	Yes	184 (25.3%)	18.7	116 (23.9%)	
Ulcer	25	Stroke, n(%)	No	750 (99.6%)	15.9	485 (99.8%)	
Ulcer	25	Stroke, n(%)	Yes	3 (0.4%)	15.9	1 (0.2%)	
Ulcer	25	Systolic blood pressure, mean(SD)		123.8 (13.4)	10.7	123.5 (12.9)	
Ulcer	25	Time since last healthcare visit, mean(SD)		0.9 (1.6)	0	0.7 (1)	
Ulcer	25	Total cholesterol, mean(SD)		4.8 (1.2)	23.6	4.8 (1.1)	
Ulcer	26	Age at diagnosis of type 2 diabetes, mean(SD)		23.2 (2.1)	0	22.8 (1.9)	
Ulcer	26	BMI, mean(SD)		35.4 (7.7)	14.3	35.2 (7.2)	
Ulcer	26	HbA1c, mean(SD)		53.5 (19.4)	7.9	52.8 (19.6)	
Ulcer	26	HDL-cholesterol, mean(SD)		1 (0.3)	25.9	1 (0.3)	
Ulcer	26	Ischemic heart disease, n(%)	No	917 (99.5%)	14.6	603 (99.8%)	
Ulcer	26	Ischemic heart disease, n(%)	Yes	5 (0.5%)	14.6	1 (0.2%)	

Ulcer	26	Neuropathy, n(%)	No	1048 (97.1%)	0	579 (95.9%)	
Ulcer	26	Neuropathy, n(%)	Yes	31 (2.9%)	0	25 (4.1%)	
Ulcer	26	Self-reported physical activity, n(%)	Never	120 (14.3%)	22.2	84 (13.9%)	
Ulcer	26	Self-reported physical activity, n(%)	<1 times/week	113 (13.5%)	22.2	81 (13.4%)	
Ulcer	26	Self-reported physical activity, n(%)	regularly - 1-2 times/week	164 (19.5%)	22.2	120 (19.9%)	
Ulcer	26	Self-reported physical activity, n(%)	regularly - 3-5 times/week	232 (27.6%)	22.2	172 (28.5%)	
Ulcer	26	Self-reported physical activity, n(%)	Daily	211 (25.1%)	22.2	147 (24.3%)	
Ulcer	26	Sex, n(%)	Males	544 (50.5%)	0.2	303 (50.2%)	n=1079 vs n=604
Ulcer	26	Sex, n(%)	Females	533 (49.5%)	0.2	301 (49.8%)	
Ulcer	26	Smoking status, n(%)	No	683 (76.3%)	17.1	461 (76.3%)	
Ulcer	26	Smoking status, n(%)	Yes	212 (23.7%)	17.1	143 (23.7%)	
Ulcer	26	Stroke, n(%)	No	906 (99.6%)	15.7	602 (99.7%)	
Ulcer	26	Stroke, n(%)	Yes	4 (0.4%)	15.7	2 (0.3%)	
Ulcer	26	Systolic blood pressure, mean(SD)		124.4 (13.4)	10.3	124.4 (13.3)	
Ulcer	26	Time since last healthcare visit, mean(SD)		0.9 (1.6)	0	0.7 (1)	
Ulcer	26	Total cholesterol, mean(SD)		4.8 (1.2)	22.9	4.8 (1.2)	
Ulcer	27	Age at diagnosis of type 2 diabetes, mean(SD)		24 (2.3)	0	23.6 (2.1)	
Ulcer	27	BMI, mean(SD)		35 (7.5)	15.3	35.2 (7.3)	
Ulcer	27	HbA1c, mean(SD)		53 (18.9)	7.5	53.6 (19.2)	
Ulcer	27	HDL-cholesterol, mean(SD)		1 (0.3)	26.2	1 (0.4)	
Ulcer	27	Ischemic heart disease, n(%)	No	1097 (99.3%)	13.9	719 (99.6%)	
Ulcer	27	Ischemic heart disease, n(%)	Yes	8 (0.7%)	13.9	3 (0.4%)	
Ulcer	27	Neuropathy, n(%)	No	1238 (96.4%)	0	686 (95%)	

Ulcer	27	Neuropathy, n(%)	Yes	46 (3.6%)	0	36 (5%)	
Ulcer	27	Self-reported physical activity, n(%)	Never	132 (13.4%)	23.4	96 (13.3%)	
Ulcer	27	Self-reported physical activity, n(%)	<1 times/week	137 (13.9%)	23.4	98 (13.6%)	
Ulcer	27	Self-reported physical activity, n(%)	regularly - 1-2 times/week	188 (19.1%)	23.4	137 (19%)	
Ulcer	27	Self-reported physical activity, n(%)	regularly - 3-5 times/week	284 (28.9%)	23.4	225 (31.2%)	
Ulcer	27	Self-reported physical activity, n(%)	Daily	243 (24.7%)	23.4	166 (23%)	
Ulcer	27	Sex, n(%)	Males	648 (50.6%)	0.2	379 (52.5%)	n=1284 vs n=722
Ulcer	27	Sex, n(%)	Females	633 (49.4%)	0.2	343 (47.5%)	
Ulcer	27	Smoking status, n(%)	No	798 (75.6%)	17.8	547 (75.8%)	
Ulcer	27	Smoking status, n(%)	Yes	257 (24.4%)	17.8	175 (24.2%)	
Ulcer	27	Stroke, n(%)	No	1090 (99.3%)	14.5	719 (99.6%)	
Ulcer	27	Stroke, n(%)	Yes	8 (0.7%)	14.5	3 (0.4%)	
Ulcer	27	Systolic blood pressure, mean(SD)		124.6 (13.8)	10.1	124.7 (14)	
Ulcer	27	Time since last healthcare visit, mean(SD)		1 (1.7)	0	0.7 (1.1)	
Ulcer	27	Total cholesterol, mean(SD)		4.8 (1.2)	21.7	4.8 (1.2)	
Ulcer	28	Age at diagnosis of type 2 diabetes, mean(SD)		24.8 (2.5)	0	24.4 (2.2)	
Ulcer	28	BMI, mean(SD)		35.1 (7.5)	15.2	35.1 (7.2)	
Ulcer	28	HbA1c, mean(SD)		52.9 (18.6)	7.6	53.7 (18.2)	
Ulcer	28	HDL-cholesterol, mean(SD)		1 (0.3)	26.1	1 (0.3)	
Ulcer	28	Ischemic heart disease, n(%)	No	1276 (98.8%)	14.2	837 (98.8%)	
Ulcer	28	Ischemic heart disease, n(%)	Yes	15 (1.2%)	14.2	10 (1.2%)	
Ulcer	28	Neuropathy, n(%)	No	1452 (96.5%)	0	805 (95%)	
Ulcer	28	Neuropathy, n(%)	Yes	52 (3.5%)	0	42 (5%)	

Ulcer	28	Self-reported physical activity, n(%)	Never	151 (13%)	22.9	110 (13%)	
Ulcer	28	Self-reported physical activity, n(%)	<1 times/week	126 (10.9%)	22.9	88 (10.4%)	
Ulcer	28	Self-reported physical activity, n(%)	regularly - 1-2 times/week	254 (21.9%)	22.9	181 (21.4%)	
Ulcer	28	Self-reported physical activity, n(%)	regularly - 3-5 times/week	320 (27.6%)	22.9	244 (28.8%)	
Ulcer	28	Self-reported physical activity, n(%)	Daily	308 (26.6%)	22.9	224 (26.4%)	
Ulcer	28	Sex, n(%)	Males	746 (49.7%)	0.2	438 (51.7%)	n=1504 vs n=847
Ulcer	28	Sex, n(%)	Females	755 (50.3%)	0.2	409 (48.3%)	
Ulcer	28	Smoking status, n(%)	No	942 (76%)	17.6	652 (77%)	
Ulcer	28	Smoking status, n(%)	Yes	297 (24%)	17.6	195 (23%)	
Ulcer	28	Stroke, n(%)	No	1277 (99.1%)	14.4	840 (99.2%)	
Ulcer	28	Stroke, n(%)	Yes	11 (0.9%)	14.4	7 (0.8%)	
Ulcer	28	Systolic blood pressure, mean(SD)		124.8 (13.4)	10.2	125.4 (13.5)	
Ulcer	28	Time since last healthcare visit, mean(SD)		1 (1.7)	0	0.8 (1.1)	
Ulcer	28	Total cholesterol, mean(SD)		4.8 (1.1)	21.7	4.8 (1.1)	
Ulcer	29	Age at diagnosis of type 2 diabetes, mean(SD)		25.7 (2.6)	0	25.2 (2.4)	
Ulcer	29	BMI, mean(SD)		34.8 (7.5)	14.7	34.7 (7.1)	
Ulcer	29	HbA1c, mean(SD)		53.8 (18.9)	7.3	54.1 (18.4)	
Ulcer	29	HDL-cholesterol, mean(SD)		1 (0.3)	25.9	1 (0.3)	
Ulcer	29	Ischemic heart disease, n(%)	No	1472 (99.1%)	13.7	979 (99.2%)	
Ulcer	29	Ischemic heart disease, n(%)	Yes	13 (0.9%)	13.7	8 (0.8%)	
Ulcer	29	Neuropathy, n(%)	No	1649 (95.9%)	0	927 (93.9%)	
Ulcer	29	Neuropathy, n(%)	Yes	71 (4.1%)	0	60 (6.1%)	
Ulcer	29	Self-reported physical activity, n(%)	Never	174 (13.1%)	22.6	127 (12.9%)	

Ulcer	29	Self-reported physical activity, n(%)	<1 times/week	164 (12.3%)	22.6	122 (12.4%)	
Ulcer	29	Self-reported physical activity, n(%)	regularly - 1-2 times/week	279 (21%)	22.6	200 (20.3%)	
Ulcer	29	Self-reported physical activity, n(%)	regularly - 3-5 times/week	353 (26.5%)	22.6	280 (28.4%)	
Ulcer	29	Self-reported physical activity, n(%)	Daily	361 (27.1%)	22.6	258 (26.1%)	
Ulcer	29	Sex, n(%)	Males	876 (51%)	0.2	511 (51.8%)	n=1720 vs n=987
Ulcer	29	Sex, n(%)	Females	840 (49%)	0.2	476 (48.2%)	
Ulcer	29	Smoking status, n(%)	No	1106 (77.4%)	16.9	756 (76.6%)	
Ulcer	29	Smoking status, n(%)	Yes	323 (22.6%)	16.9	231 (23.4%)	
Ulcer	29	Stroke, n(%)	No	1467 (99.3%)	14.1	981 (99.4%)	
Ulcer	29	Stroke, n(%)	Yes	11 (0.7%)	14.1	6 (0.6%)	
Ulcer	29	Systolic blood pressure, mean(SD)		124.5 (13.6)	9.7	124.8 (13.5)	
Ulcer	29	Time since last healthcare visit, mean(SD)		1 (1.7)	0	0.8 (1.1)	
Ulcer	29	Total cholesterol, mean(SD)		4.8 (1.1)	21.3	4.8 (1.1)	
Ulcer	30	Age at diagnosis of type 2 diabetes, mean(SD)		26.7 (2.7)	0	26.2 (2.6)	
Ulcer	30	BMI, mean(SD)		34.7 (7.6)	14.2	34.6 (7.4)	
Ulcer	30	HbA1c, mean(SD)		53.7 (19.2)	7	54 (18.7)	
Ulcer	30	HDL-cholesterol, mean(SD)		1 (0.3)	25.6	1 (0.3)	
Ulcer	30	Ischemic heart disease, n(%)	No	1677 (99.2%)	13.5	1124 (99.3%)	
Ulcer	30	Ischemic heart disease, n(%)	Yes	13 (0.8%)	13.5	8 (0.7%)	
Ulcer	30	Neuropathy, n(%)	No	1877 (96.1%)	0	1063 (93.9%)	
Ulcer	30	Neuropathy, n(%)	Yes	77 (3.9%)	0	69 (6.1%)	
Ulcer	30	Self-reported physical activity, n(%)	Never	189 (12.3%)	21.1	148 (13.1%)	
Ulcer	30	Self-reported physical activity, n(%)	<1 times/week	207 (13.4%)	21.1	141 (12.5%)	

Ulcer	30	Self-reported physical activity, n(%)	regularly - 1-2 times/week	338 (21.9%)	21.1	247 (21.8%)	
Ulcer	30	Self-reported physical activity, n(%)	regularly - 3-5 times/week	399 (25.9%)	21.1	292 (25.8%)	
Ulcer	30	Self-reported physical activity, n(%)	Daily	408 (26.5%)	21.1	304 (26.9%)	
Ulcer	30	Sex, n(%)	Males	1023 (52.5%)	0.2	618 (54.6%)	n=1954 vs n=1132
Ulcer	30	Sex, n(%)	Females	927 (47.5%)	0.2	514 (45.4%)	
Ulcer	30	Smoking status, n(%)	No	1273 (77.3%)	15.8	890 (78.6%)	
Ulcer	30	Smoking status, n(%)	Yes	373 (22.7%)	15.8	242 (21.4%)	
Ulcer	30	Stroke, n(%)	No	1673 (99.3%)	13.8	1125 (99.4%)	
Ulcer	30	Stroke, n(%)	Yes	11 (0.7%)	13.8	7 (0.6%)	
Ulcer	30	Systolic blood pressure, mean(SD)		124.7 (13.7)	8.8	125.2 (13.7)	
Ulcer	30	Time since last healthcare visit, mean(SD)		1 (1.7)	0	0.7 (1.1)	
Ulcer	30	Total cholesterol, mean(SD)		4.8 (1.2)	21.7	4.8 (1.1)	
Ulcer	31	Age at diagnosis of type 2 diabetes, mean(SD)		27.6 (2.7)	0	27 (2.7)	
Ulcer	31	BMI, mean(SD)		34.3 (7.4)	13.5	34.2 (7)	
Ulcer	31	HbA1c, mean(SD)		53.3 (18.6)	6.5	53.6 (18)	
Ulcer	31	HDL-cholesterol, mean(SD)		1 (0.3)	25	1 (0.3)	
Ulcer	31	Ischemic heart disease, n(%)	No	1931 (99.3%)	12.6	1282 (99.3%)	
Ulcer	31	Ischemic heart disease, n(%)	Yes	13 (0.7%)	12.6	9 (0.7%)	
Ulcer	31	Neuropathy, n(%)	No	2137 (96.1%)	0	1221 (94.6%)	
Ulcer	31	Neuropathy, n(%)	Yes	87 (3.9%)	0	70 (5.4%)	
Ulcer	31	Self-reported physical activity, n(%)	Never	216 (12.3%)	21.3	164 (12.7%)	
Ulcer	31	Self-reported physical activity, n(%)	<1 times/week	233 (13.3%)	21.3	163 (12.6%)	
Ulcer	31	Self-reported physical activity, n(%)	regularly - 1-2 times/week	373 (21.3%)	21.3	274 (21.2%)	

Ulcer	31	Self-reported physical activity, n(%)	regularly - 3-5 times/week	459 (26.2%)	21.3	344 (26.6%)	
Ulcer	31	Self-reported physical activity, n(%)	Daily	469 (26.8%)	21.3	346 (26.8%)	
Ulcer	31	Sex, n(%)	Males	1200 (54.1%)	0.2	730 (56.5%)	n=2224 vs n=1291
Ulcer	31	Sex, n(%)	Females	1020 (45.9%)	0.2	561 (43.5%)	
Ulcer	31	Smoking status, n(%)	No	1461 (77.1%)	14.7	1002 (77.6%)	
Ulcer	31	Smoking status, n(%)	Yes	435 (22.9%)	14.7	289 (22.4%)	
Ulcer	31	Stroke, n(%)	No	1925 (99.3%)	12.8	1279 (99.1%)	
Ulcer	31	Stroke, n(%)	Yes	14 (0.7%)	12.8	12 (0.9%)	
Ulcer	31	Systolic blood pressure, mean(SD)		124.7 (13.7)	8.9	125.1 (13.6)	
Ulcer	31	Time since last healthcare visit, mean(SD)		1 (1.6)	0	0.7 (1)	
Ulcer	31	Total cholesterol, mean(SD)		4.8 (1.2)	20.5	4.8 (1.2)	
Ulcer	32	Age at diagnosis of type 2 diabetes, mean(SD)		28.6 (2.7)	0	28.1 (2.7)	
Ulcer	32	BMI, mean(SD)		34.1 (7.5)	13.2	33.9 (7.4)	
Ulcer	32	HbA1c, mean(SD)		53 (18.7)	6.1	53.5 (18.2)	
Ulcer	32	HDL-cholesterol, mean(SD)		1 (0.3)	24	1 (0.3)	
Ulcer	32	Ischemic heart disease, n(%)	No	2236 (99.3%)	12.4	1507 (99.4%)	
Ulcer	32	Ischemic heart disease, n(%)	Yes	15 (0.7%)	12.4	9 (0.6%)	
Ulcer	32	Neuropathy, n(%)	No	2462 (95.8%)	0	1433 (94.5%)	
Ulcer	32	Neuropathy, n(%)	Yes	107 (4.2%)	0	83 (5.5%)	
Ulcer	32	Self-reported physical activity, n(%)	Never	275 (13.4%)	20.3	206 (13.6%)	
Ulcer	32	Self-reported physical activity, n(%)	<1 times/week	263 (12.8%)	20.3	187 (12.3%)	
Ulcer	32	Self-reported physical activity, n(%)	regularly - 1-2 times/week	431 (21%)	20.3	307 (20.3%)	
Ulcer	32	Self-reported physical activity, n(%)	regularly - 3-5 times/week	540 (26.4%)	20.3	427 (28.2%)	

Ulcer	32	Self-reported physical activity, n(%)	Daily	539 (26.3%)	20.3	389 (25.7%)	
Ulcer	32	Sex, n(%)	Males	1399 (54.6%)	0.2	869 (57.3%)	n=2569 vs n=1516
Ulcer	32	Sex, n(%)	Females	1165 (45.4%)	0.2	647 (42.7%)	
Ulcer	32	Smoking status, n(%)	No	1711 (77.5%)	14	1191 (78.6%)	
Ulcer	32	Smoking status, n(%)	Yes	498 (22.5%)	14	325 (21.4%)	
Ulcer	32	Stroke, n(%)	No	2235 (99.4%)	12.5	1505 (99.3%)	
Ulcer	32	Stroke, n(%)	Yes	13 (0.6%)	12.5	11 (0.7%)	
Ulcer	32	Systolic blood pressure, mean(SD)		124.9 (14.2)	7.8	125.3 (14)	
Ulcer	32	Time since last healthcare visit, mean(SD)		0.9 (1.5)	0	0.7 (1.1)	
Ulcer	32	Total cholesterol, mean(SD)		4.8 (1.2)	19.4	4.8 (1.2)	
Ulcer	33	Age at diagnosis of type 2 diabetes, mean(SD)		29.7 (2.6)	0	29.1 (2.7)	
Ulcer	33	BMI, mean(SD)		34 (7.5)	13.2	34 (7.5)	
Ulcer	33	HbA1c, mean(SD)		52.9 (17.9)	5.4	53 (17.1)	
Ulcer	33	HDL-cholesterol, mean(SD)		1 (0.3)	23	1.1 (0.3)	
Ulcer	33	Ischemic heart disease, n(%)	No	2576 (99.2%)	12.6	1731 (99.4%)	
Ulcer	33	Ischemic heart disease, n(%)	Yes	21 (0.8%)	12.6	10 (0.6%)	
Ulcer	33	Neuropathy, n(%)	No	2855 (96.1%)	0	1654 (95%)	
Ulcer	33	Neuropathy, n(%)	Yes	117 (3.9%)	0	87 (5%)	
Ulcer	33	Self-reported physical activity, n(%)	Never	325 (13.7%)	20.2	251 (14.4%)	
Ulcer	33	Self-reported physical activity, n(%)	<1 times/week	315 (13.3%)	20.2	223 (12.8%)	
Ulcer	33	Self-reported physical activity, n(%)	regularly - 1-2 times/week	497 (20.9%)	20.2	350 (20.1%)	
Ulcer	33	Self-reported physical activity, n(%)	regularly - 3-5 times/week	588 (24.8%)	20.2	434 (24.9%)	
Ulcer	33	Self-reported physical activity, n(%)	Daily	648 (27.3%)	20.2	483 (27.7%)	

Ulcer	33	Sex, n(%)	Males	1651 (55.6%)	0.2	987 (56.7%)	n=2972 vs n=1741
Ulcer	33	Sex, n(%)	Females	1316 (44.4%)	0.2	754 (43.3%)	
Ulcer	33	Smoking status, n(%)	No	1965 (76.4%)	13.5	1346 (77.3%)	
Ulcer	33	Smoking status, n(%)	Yes	607 (23.6%)	13.5	395 (22.7%)	
Ulcer	33	Stroke, n(%)	No	2578 (99.5%)	12.8	1731 (99.4%)	
Ulcer	33	Stroke, n(%)	Yes	13 (0.5%)	12.8	10 (0.6%)	
Ulcer	33	Systolic blood pressure, mean(SD)		125 (14.2)	7.4	125.1 (13.9)	
Ulcer	33	Time since last healthcare visit, mean(SD)		0.9 (1.5)	0	0.7 (1)	
Ulcer	33	Total cholesterol, mean(SD)		4.9 (1.2)	18.5	4.8 (1.2)	
Ulcer	34	Age at diagnosis of type 2 diabetes, mean(SD)		30.6 (2.7)	0	30.1 (2.6)	
Ulcer	34	BMI, mean(SD)		33.9 (7.5)	12	33.8 (7.4)	
Ulcer	34	HbA1c, mean(SD)		52.6 (17.4)	4.6	52.6 (16.7)	
Ulcer	34	HDL-cholesterol, mean(SD)		1.1 (0.3)	22.5	1.1 (0.3)	
Ulcer	34	Ischemic heart disease, n(%)	No	2963 (98.9%)	12	2004 (99.1%)	
Ulcer	34	Ischemic heart disease, n(%)	Yes	32 (1.1%)	12	19 (0.9%)	
Ulcer	34	Neuropathy, n(%)	No	3245 (95.4%)	0	1908 (94.3%)	
Ulcer	34	Neuropathy, n(%)	Yes	157 (4.6%)	0	115 (5.7%)	
Ulcer	34	Self-reported physical activity, n(%)	Never	370 (13.5%)	19.6	268 (13.2%)	
Ulcer	34	Self-reported physical activity, n(%)	<1 times/week	344 (12.6%)	19.6	254 (12.6%)	
Ulcer	34	Self-reported physical activity, n(%)	regularly - 1-2 times/week	592 (21.6%)	19.6	431 (21.3%)	
Ulcer	34	Self-reported physical activity, n(%)	regularly - 3-5 times/week	694 (25.4%)	19.6	516 (25.5%)	
Ulcer	34	Self-reported physical activity, n(%)	Daily	735 (26.9%)	19.6	554 (27.4%)	
Ulcer	34	Sex, n(%)	Males	1894 (55.8%)	0.2	1168 (57.7%)	n=3402 vs n=2023

Ulcer	34	Sex, n(%)	Females	1502 (44.2%)	0.2	855 (42.3%)	
Ulcer	34	Smoking status, n(%)	No	2301 (78.1%)	13.4	1601 (79.1%)	
Ulcer	34	Smoking status, n(%)	Yes	644 (21.9%)	13.4	422 (20.9%)	
Ulcer	34	Stroke, n(%)	No	2974 (99.5%)	12.1	2012 (99.5%)	
Ulcer	34	Stroke, n(%)	Yes	15 (0.5%)	12.1	11 (0.5%)	
Ulcer	34	Systolic blood pressure, mean(SD)		125.4 (14.4)	7.6	125.4 (14.1)	
Ulcer	34	Time since last healthcare visit, mean(SD)		0.9 (1.4)	0	0.7 (1)	
Ulcer	34	Total cholesterol, mean(SD)		4.9 (1.1)	17.8	4.8 (1.1)	
Ulcer	35	Age at diagnosis of type 2 diabetes, mean(SD)		31.6 (2.6)	0	31.1 (2.6)	
Ulcer	35	BMI, mean(SD)		33.9 (7.4)	12	33.8 (7.4)	
Ulcer	35	HbA1c, mean(SD)		52.6 (17.5)	4.5	52.6 (16.8)	
Ulcer	35	HDL-cholesterol, mean(SD)		1.1 (0.3)	21.2	1.1 (0.3)	
Ulcer	35	Ischemic heart disease, n(%)	No	3353 (99%)	12.3	2299 (98.9%)	
Ulcer	35	Ischemic heart disease, n(%)	Yes	33 (1%)	12.3	25 (1.1%)	
Ulcer	35	Neuropathy, n(%)	No	3676 (95.2%)	0	2182 (93.9%)	
Ulcer	35	Neuropathy, n(%)	Yes	185 (4.8%)	0	142 (6.1%)	
Ulcer	35	Self-reported physical activity, n(%)	Never	442 (14.2%)	19.5	323 (13.9%)	
Ulcer	35	Self-reported physical activity, n(%)	<1 times/week	386 (12.4%)	19.5	279 (12%)	
Ulcer	35	Self-reported physical activity, n(%)	regularly - 1-2 times/week	668 (21.5%)	19.5	509 (21.9%)	
Ulcer	35	Self-reported physical activity, n(%)	regularly - 3-5 times/week	779 (25%)	19.5	581 (25%)	
Ulcer	35	Self-reported physical activity, n(%)	Daily	835 (26.8%)	19.5	632 (27.2%)	
Ulcer	35	Sex, n(%)	Males	2167 (56.2%)	0.1	1353 (58.2%)	n=3861 vs n=2324
Ulcer	35	Sex, n(%)	Females	1689 (43.8%)	0.1	971 (41.8%)	

Ulcer	35	Smoking status, n(%)	No	2653 (79.3%)	13.3	1864 (80.2%)	
Ulcer	35	Smoking status, n(%)	Yes	694 (20.7%)	13.3	460 (19.8%)	
Ulcer	35	Stroke, n(%)	No	3370 (99.6%)	12.4	2312 (99.5%)	
Ulcer	35	Stroke, n(%)	Yes	14 (0.4%)	12.4	12 (0.5%)	
Ulcer	35	Systolic blood pressure, mean(SD)		125.6 (14.5)	7.2	125.3 (14.2)	
Ulcer	35	Time since last healthcare visit, mean(SD)		0.9 (1.4)	0	0.7 (1)	
Ulcer	35	Total cholesterol, mean(SD)		4.9 (1.1)	16.9	4.8 (1.1)	
Ulcer	36	Age at diagnosis of type 2 diabetes, mean(SD)		32.6 (2.6)	0	32.2 (2.6)	
Ulcer	36	BMI, mean(SD)		33.7 (7.2)	11.3	33.6 (7.2)	
Ulcer	36	HbA1c, mean(SD)		52.6 (17.6)	4.1	52.9 (17)	
Ulcer	36	HDL-cholesterol, mean(SD)		1.1 (0.3)	20.9	1.1 (0.3)	
Ulcer	36	Ischemic heart disease, n(%)	No	3765 (98.8%)	12.6	2654 (98.9%)	
Ulcer	36	Ischemic heart disease, n(%)	Yes	45 (1.2%)	12.6	30 (1.1%)	
Ulcer	36	Neuropathy, n(%)	No	4142 (95%)	0	2511 (93.6%)	
Ulcer	36	Neuropathy, n(%)	Yes	219 (5%)	0	173 (6.4%)	
Ulcer	36	Self-reported physical activity, n(%)	Never	535 (15%)	18.4	394 (14.7%)	
Ulcer	36	Self-reported physical activity, n(%)	<1 times/week	450 (12.6%)	18.4	327 (12.2%)	
Ulcer	36	Self-reported physical activity, n(%)	regularly - 1-2 times/week	741 (20.8%)	18.4	579 (21.6%)	
Ulcer	36	Self-reported physical activity, n(%)	regularly - 3-5 times/week	877 (24.6%)	18.4	654 (24.4%)	
Ulcer	36	Self-reported physical activity, n(%)	Daily	956 (26.9%)	18.4	730 (27.2%)	
Ulcer	36	Sex, n(%)	Males	2469 (56.7%)	0.2	1604 (59.8%)	n=4361 vs n=2684
Ulcer	36	Sex, n(%)	Females	1884 (43.3%)	0.2	1080 (40.2%)	
Ulcer	36	Smoking status, n(%)	No	3029 (78.9%)	12	2156 (80.3%)	

Ulcer	36	Smoking status, n(%)	Yes	808 (21.1%)	12	528 (19.7%)	
Ulcer	36	Stroke, n(%)	No	3785 (99.5%)	12.8	2666 (99.3%)	
Ulcer	36	Stroke, n(%)	Yes	19 (0.5%)	12.8	18 (0.7%)	
Ulcer	36	Systolic blood pressure, mean(SD)		125.9 (14.4)	7.3	125.9 (14.1)	
Ulcer	36	Time since last healthcare visit, mean(SD)		0.9 (1.4)	0	0.7 (1)	
Ulcer	36	Total cholesterol, mean(SD)		4.9 (1.2)	16.4	4.9 (1.2)	
Ulcer	37	Age at diagnosis of type 2 diabetes, mean(SD)		33.6 (2.6)	0	33.1 (2.6)	
Ulcer	37	BMI, mean(SD)		33.7 (7.2)	10.5	33.5 (7.1)	
Ulcer	37	HbA1c, mean(SD)		52.6 (17.5)	4	52.7 (16.9)	
Ulcer	37	HDL-cholesterol, mean(SD)		1.1 (0.3)	20.4	1.1 (0.3)	
Ulcer	37	Ischemic heart disease, n(%)	No	4342 (98.5%)	11.8	3052 (98.7%)	
Ulcer	37	Ischemic heart disease, n(%)	Yes	64 (1.5%)	11.8	39 (1.3%)	
Ulcer	37	Neuropathy, n(%)	No	4730 (94.6%)	0	2886 (93.4%)	
Ulcer	37	Neuropathy, n(%)	Yes	268 (5.4%)	0	205 (6.6%)	
Ulcer	37	Self-reported physical activity, n(%)	Never	602 (14.7%)	18.2	443 (14.3%)	
Ulcer	37	Self-reported physical activity, n(%)	<1 times/week	555 (13.6%)	18.2	414 (13.4%)	
Ulcer	37	Self-reported physical activity, n(%)	regularly - 1-2 times/week	836 (20.5%)	18.2	655 (21.2%)	
Ulcer	37	Self-reported physical activity, n(%)	regularly - 3-5 times/week	990 (24.2%)	18.2	736 (23.8%)	
Ulcer	37	Self-reported physical activity, n(%)	Daily	1104 (27%)	18.2	843 (27.3%)	
Ulcer	37	Sex, n(%)	Males	2844 (57%)	0.2	1855 (60%)	n=4998 vs n=3091
Ulcer	37	Sex, n(%)	Females	2144 (43%)	0.2	1236 (40%)	
Ulcer	37	Smoking status, n(%)	No	3515 (79.5%)	11.5	2497 (80.8%)	
Ulcer	37	Smoking status, n(%)	Yes	909 (20.5%)	11.5	594 (19.2%)	

Ulcer	37	Stroke, n(%)	No	4360 (99.2%)	12.1	3066 (99.2%)	
Ulcer	37	Stroke, n(%)	Yes	35 (0.8%)	12.1	25 (0.8%)	
Ulcer	37	Systolic blood pressure, mean(SD)		126.1 (14.2)	6	125.9 (13.7)	
Ulcer	37	Time since last healthcare visit, mean(SD)		0.9 (1.5)	0	0.7 (1)	
Ulcer	37	Total cholesterol, mean(SD)		4.9 (1.2)	16.1	4.8 (1.2)	
Ulcer	38	Age at diagnosis of type 2 diabetes, mean(SD)		34.5 (2.6)	0	34.1 (2.6)	
Ulcer	38	BMI, mean(SD)		33.5 (7.1)	11.2	33.4 (7)	
Ulcer	38	HbA1c, mean(SD)		52.4 (17.3)	4.1	52.7 (17)	
Ulcer	38	HDL-cholesterol, mean(SD)		1.1 (0.3)	19.6	1.1 (0.3)	
Ulcer	38	Ischemic heart disease, n(%)	No	4985 (98.3%)	11.6	3535 (98.3%)	
Ulcer	38	Ischemic heart disease, n(%)	Yes	84 (1.7%)	11.6	60 (1.7%)	
Ulcer	38	Neuropathy, n(%)	No	5424 (94.6%)	0	3354 (93.3%)	
Ulcer	38	Neuropathy, n(%)	Yes	308 (5.4%)	0	241 (6.7%)	
Ulcer	38	Self-reported physical activity, n(%)	Never	685 (14.7%)	18.5	521 (14.5%)	
Ulcer	38	Self-reported physical activity, n(%)	<1 times/week	612 (13.1%)	18.5	472 (13.1%)	
Ulcer	38	Self-reported physical activity, n(%)	regularly - 1-2 times/week	974 (20.8%)	18.5	776 (21.6%)	
Ulcer	38	Self-reported physical activity, n(%)	regularly - 3-5 times/week	1117 (23.9%)	18.5	831 (23.1%)	
Ulcer	38	Self-reported physical activity, n(%)	Daily	1286 (27.5%)	18.5	995 (27.7%)	
Ulcer	38	Sex, n(%)	Males	3287 (57.5%)	0.2	2157 (60%)	n=5732 vs n=3595
Ulcer	38	Sex, n(%)	Females	2434 (42.5%)	0.2	1438 (40%)	
Ulcer	38	Smoking status, n(%)	No	4024 (79.1%)	11.3	2891 (80.4%)	
Ulcer	38	Smoking status, n(%)	Yes	1063 (20.9%)	11.3	704 (19.6%)	
Ulcer	38	Stroke, n(%)	No	5014 (99.1%)	11.7	3567 (99.2%)	

Ulcer	38	Stroke, n(%)	Yes	45 (0.9%)	11.7	28 (0.8%)	
Ulcer	38	Systolic blood pressure, mean(SD)		126.4 (14.5)	6.1	126.2 (14.1)	
Ulcer	38	Time since last healthcare visit, mean(SD)		0.9 (1.4)	0	0.7 (1)	
Ulcer	38	Total cholesterol, mean(SD)		4.9 (1.1)	15.1	4.8 (1.1)	
Ulcer	39	Age at diagnosis of type 2 diabetes, mean(SD)		35.5 (2.7)	0	35.1 (2.7)	
Ulcer	39	BMI, mean(SD)		33.4 (7)	10.5	33.2 (6.8)	
Ulcer	39	HbA1c, mean(SD)		52.7 (17.4)	3.8	52.7 (17.1)	
Ulcer	39	HDL-cholesterol, mean(SD)		1.1 (0.3)	19.5	1.1 (0.3)	
Ulcer	39	Ischemic heart disease, n(%)	No	5816 (98.4%)	11.6	4173 (98.3%)	
Ulcer	39	Ischemic heart disease, n(%)	Yes	93 (1.6%)	11.6	72 (1.7%)	
Ulcer	39	Neuropathy, n(%)	No	6302 (94.3%)	0	3939 (92.8%)	
Ulcer	39	Neuropathy, n(%)	Yes	380 (5.7%)	0	306 (7.2%)	
Ulcer	39	Self-reported physical activity, n(%)	Never	757 (13.7%)	17.2	589 (13.9%)	
Ulcer	39	Self-reported physical activity, n(%)	<1 times/week	718 (13%)	17.2	541 (12.7%)	
Ulcer	39	Self-reported physical activity, n(%)	regularly - 1-2 times/week	1162 (21%)	17.2	885 (20.8%)	
Ulcer	39	Self-reported physical activity, n(%)	regularly - 3-5 times/week	1401 (25.3%)	17.2	1082 (25.5%)	
Ulcer	39	Self-reported physical activity, n(%)	Daily	1494 (27%)	17.2	1148 (27%)	
Ulcer	39	Sex, n(%)	Males	3863 (57.9%)	0.2	2550 (60.1%)	n=6682 vs n=4245
Ulcer	39	Sex, n(%)	Females	2807 (42.1%)	0.2	1695 (39.9%)	
Ulcer	39	Smoking status, n(%)	No	4707 (79.1%)	11	3417 (80.5%)	
Ulcer	39	Smoking status, n(%)	Yes	1243 (20.9%)	11	828 (19.5%)	
Ulcer	39	Stroke, n(%)	No	5842 (99.1%)	11.8	4203 (99%)	
Ulcer	39	Stroke, n(%)	Yes	54 (0.9%)	11.8	42 (1%)	

Ulcer	39	Systolic blood pressure, mean(SD)		126.8 (14.6)	5.7	126.7 (14.2)	
Ulcer	39	Time since last healthcare visit, mean(SD)		0.8 (1.4)	0	0.7 (1)	
Ulcer	39	Total cholesterol, mean(SD)		4.9 (1.2)	15.2	4.8 (1.2)	
Ulcer	40	Age at diagnosis of type 2 diabetes, mean(SD)		36.5 (2.6)	0	36 (2.7)	
Ulcer	40	BMI, mean(SD)		33.4 (6.8)	10.1	33.3 (6.8)	
Ulcer	40	HbA1c, mean(SD)		52.7 (17)	3.7	52.8 (16.8)	
Ulcer	40	HDL-cholesterol, mean(SD)		1.1 (0.3)	18.8	1.1 (0.3)	
Ulcer	40	Ischemic heart disease, n(%)	No	6639 (98.4%)	11.1	4772 (98.3%)	
Ulcer	40	Ischemic heart disease, n(%)	Yes	109 (1.6%)	11.1	82 (1.7%)	
Ulcer	40	Neuropathy, n(%)	No	7160 (94.3%)	0	4502 (92.7%)	
Ulcer	40	Neuropathy, n(%)	Yes	432 (5.7%)	0	352 (7.3%)	
Ulcer	40	Self-reported physical activity, n(%)	Never	955 (15%)	16.3	705 (14.5%)	
Ulcer	40	Self-reported physical activity, n(%)	<1 times/week	891 (14%)	16.3	676 (13.9%)	
Ulcer	40	Self-reported physical activity, n(%)	regularly - 1-2 times/week	1330 (20.9%)	16.3	1040 (21.4%)	
Ulcer	40	Self-reported physical activity, n(%)	regularly - 3-5 times/week	1521 (23.9%)	16.3	1191 (24.5%)	
Ulcer	40	Self-reported physical activity, n(%)	Daily	1659 (26.1%)	16.3	1242 (25.6%)	
Ulcer	40	Sex, n(%)	Males	4440 (58.6%)	0.2	2924 (60.2%)	n=7592 vs n=4854
Ulcer	40	Sex, n(%)	Females	3140 (41.4%)	0.2	1930 (39.8%)	
Ulcer	40	Smoking status, n(%)	No	5326 (78.5%)	10.6	3877 (79.9%)	
Ulcer	40	Smoking status, n(%)	Yes	1463 (21.5%)	10.6	977 (20.1%)	
Ulcer	40	Stroke, n(%)	No	6667 (99.1%)	11.4	4802 (98.9%)	
Ulcer	40	Stroke, n(%)	Yes	63 (0.9%)	11.4	52 (1.1%)	
Ulcer	40	Systolic blood pressure, mean(SD)		127.2 (14.6)	5.7	127 (14.2)	

Ulcer	40	Time since last healthcare visit, mean(SD)		0.8 (1.3)	0	0.7 (1)	
Ulcer	40	Total cholesterol, mean(SD)		4.9 (1.1)	14.7	4.8 (1.1)	
Ulcer	41	Age at diagnosis of type 2 diabetes, mean(SD)		37.5 (2.7)	0	37 (2.7)	
Ulcer	41	BMI, mean(SD)		33.3 (6.9)	10	33.2 (6.8)	
Ulcer	41	HbA1c, mean(SD)		52.7 (16.8)	3.4	52.8 (16.3)	
Ulcer	41	HDL-cholesterol, mean(SD)		1.1 (0.3)	18	1.1 (0.3)	
Ulcer	41	Ischemic heart disease, n(%)	No	7702 (98%)	11	5569 (98%)	
Ulcer	41	Ischemic heart disease, n(%)	Yes	160 (2%)	11	113 (2%)	
Ulcer	41	Neuropathy, n(%)	No	8297 (94%)	0	5240 (92.2%)	
Ulcer	41	Neuropathy, n(%)	Yes	532 (6%)	0	442 (7.8%)	
Ulcer	41	Self-reported physical activity, n(%)	Never	1133 (15.3%)	16	846 (14.9%)	
Ulcer	41	Self-reported physical activity, n(%)	<1 times/week	995 (13.4%)	16	744 (13.1%)	
Ulcer	41	Self-reported physical activity, n(%)	regularly - 1-2 times/week	1587 (21.4%)	16	1235 (21.7%)	
Ulcer	41	Self-reported physical activity, n(%)	regularly - 3-5 times/week	1789 (24.1%)	16	1410 (24.8%)	
Ulcer	41	Self-reported physical activity, n(%)	Daily	1916 (25.8%)	16	1447 (25.5%)	
Ulcer	41	Sex, n(%)	Males	5215 (59.2%)	0.2	3456 (60.8%)	n=8829 vs n=5682
Ulcer	41	Sex, n(%)	Females	3599 (40.8%)	0.2	2226 (39.2%)	
Ulcer	41	Smoking status, n(%)	No	6216 (78.4%)	10.1	4535 (79.8%)	
Ulcer	41	Smoking status, n(%)	Yes	1717 (21.6%)	10.1	1147 (20.2%)	
Ulcer	41	Stroke, n(%)	No	7759 (99%)	11.2	5619 (98.9%)	
Ulcer	41	Stroke, n(%)	Yes	79 (1%)	11.2	63 (1.1%)	
Ulcer	41	Systolic blood pressure, mean(SD)		127.7 (14.5)	5	127.6 (14.3)	
Ulcer	41	Time since last healthcare visit, mean(SD)		0.8 (1.3)	0	0.7 (1)	

Ulcer	41	Total cholesterol, mean(SD)		4.9 (1.1)	13.7	4.9 (1.1)	
Ulcer	42	Age at diagnosis of type 2 diabetes, mean(SD)		38.4 (2.7)	0	37.9 (2.7)	
Ulcer	42	BMI, mean(SD)		33.2 (6.7)	9.6	33.1 (6.6)	
Ulcer	42	HbA1c, mean(SD)		52.5 (16.6)	3.2	52.8 (16.2)	
Ulcer	42	HDL-cholesterol, mean(SD)		1.1 (0.3)	17.6	1.1 (0.3)	
Ulcer	42	Ischemic heart disease, n(%)	No	8716 (97.8%)	11.2	6384 (97.8%)	
Ulcer	42	Ischemic heart disease, n(%)	Yes	199 (2.2%)	11.2	145 (2.2%)	
Ulcer	42	Neuropathy, n(%)	No	9445 (94.1%)	0	6035 (92.4%)	
Ulcer	42	Neuropathy, n(%)	Yes	597 (5.9%)	0	494 (7.6%)	
Ulcer	42	Self-reported physical activity, n(%)	Never	1283 (15.2%)	15.9	955 (14.6%)	
Ulcer	42	Self-reported physical activity, n(%)	<1 times/week	1115 (13.2%)	15.9	850 (13%)	
Ulcer	42	Self-reported physical activity, n(%)	regularly - 1-2 times/week	1758 (20.8%)	15.9	1392 (21.3%)	
Ulcer	42	Self-reported physical activity, n(%)	regularly - 3-5 times/week	2056 (24.3%)	15.9	1595 (24.4%)	
Ulcer	42	Self-reported physical activity, n(%)	Daily	2238 (26.5%)	15.9	1737 (26.6%)	
Ulcer	42	Sex, n(%)	Males	5977 (59.6%)	0.1	3979 (60.9%)	n=10042 vs n=6529
Ulcer	42	Sex, n(%)	Females	4050 (40.4%)	0.1	2550 (39.1%)	
Ulcer	42	Smoking status, n(%)	No	7080 (78.4%)	10.1	5189 (79.5%)	
Ulcer	42	Smoking status, n(%)	Yes	1950 (21.6%)	10.1	1340 (20.5%)	
Ulcer	42	Stroke, n(%)	No	8791 (98.8%)	11.4	6447 (98.7%)	
Ulcer	42	Stroke, n(%)	Yes	111 (1.2%)	11.4	82 (1.3%)	
Ulcer	42	Systolic blood pressure, mean(SD)		128.1 (14.7)	5.2	127.9 (14.4)	
Ulcer	42	Time since last healthcare visit, mean(SD)		0.8 (1.3)	0	0.7 (1)	
Ulcer	42	Total cholesterol, mean(SD)		4.9 (1.2)	13.3	4.8 (1.2)	

Ulcer	43	Age at diagnosis of type 2 diabetes, mean(SD)		39.4 (2.7)	0	38.9 (2.7)	
Ulcer	43	BMI, mean(SD)		33.1 (6.6)	9.2	33 (6.6)	
Ulcer	43	HbA1c, mean(SD)		52.5 (16.3)	3.2	52.7 (15.9)	
Ulcer	43	HDL-cholesterol, mean(SD)		1.1 (0.3)	17.5	1.1 (0.3)	
Ulcer	43	Ischemic heart disease, n(%)	No	9848 (97.4%)	10.8	7277 (97.5%)	
Ulcer	43	Ischemic heart disease, n(%)	Yes	258 (2.6%)	10.8	187 (2.5%)	
Ulcer	43	Neuropathy, n(%)	No	10598 (93.5%)	0	6846 (91.7%)	
Ulcer	43	Neuropathy, n(%)	Yes	732 (6.5%)	0	618 (8.3%)	
Ulcer	43	Self-reported physical activity, n(%)	Never	1421 (14.9%)	15.7	1091 (14.6%)	
Ulcer	43	Self-reported physical activity, n(%)	<1 times/week	1296 (13.6%)	15.7	993 (13.3%)	
Ulcer	43	Self-reported physical activity, n(%)	regularly - 1-2 times/week	1976 (20.7%)	15.7	1560 (20.9%)	
Ulcer	43	Self-reported physical activity, n(%)	regularly - 3-5 times/week	2278 (23.9%)	15.7	1785 (23.9%)	
Ulcer	43	Self-reported physical activity, n(%)	Daily	2578 (27%)	15.7	2035 (27.3%)	
Ulcer	43	Sex, n(%)	Males	6848 (60.5%)	0.1	4626 (62%)	n=11330 vs n=7464
Ulcer	43	Sex, n(%)	Females	4466 (39.5%)	0.1	2838 (38%)	
Ulcer	43	Smoking status, n(%)	No	8039 (78.4%)	9.5	5917 (79.3%)	
Ulcer	43	Smoking status, n(%)	Yes	2219 (21.6%)	9.5	1547 (20.7%)	
Ulcer	43	Stroke, n(%)	No	9946 (98.7%)	11.1	7363 (98.6%)	
Ulcer	43	Stroke, n(%)	Yes	132 (1.3%)	11.1	101 (1.4%)	
Ulcer	43	Systolic blood pressure, mean(SD)		128.7 (14.8)	4.8	128.5 (14.5)	
Ulcer	43	Time since last healthcare visit, mean(SD)		0.8 (1.3)	0	0.7 (1)	
Ulcer	43	Total cholesterol, mean(SD)		4.9 (1.2)	13.2	4.8 (1.2)	
Ulcer	44	Age at diagnosis of type 2 diabetes, mean(SD)		40.4 (2.7)	0	39.9 (2.7)	

Ulcer	44	BMI, mean(SD)		33 (6.6)	9.1	32.9 (6.5)	
Ulcer	44	HbA1c, mean(SD)		52.7 (16.3)	3.1	52.7 (15.8)	
Ulcer	44	HDL-cholesterol, mean(SD)		1.1 (0.3)	17.5	1.1 (0.3)	
Ulcer	44	Ischemic heart disease, n(%)	No	11128 (96.9%)	11	8212 (96.9%)	
Ulcer	44	Ischemic heart disease, n(%)	Yes	361 (3.1%)	11	263 (3.1%)	
Ulcer	44	Neuropathy, n(%)	No	12015 (93.1%)	0	7730 (91.2%)	
Ulcer	44	Neuropathy, n(%)	Yes	897 (6.9%)	0	745 (8.8%)	
Ulcer	44	Self-reported physical activity, n(%)	Never	1598 (14.7%)	15.7	1216 (14.3%)	
Ulcer	44	Self-reported physical activity, n(%)	<1 times/week	1499 (13.8%)	15.7	1143 (13.5%)	
Ulcer	44	Self-reported physical activity, n(%)	regularly - 1-2 times/week	2236 (20.5%)	15.7	1747 (20.6%)	
Ulcer	44	Self-reported physical activity, n(%)	regularly - 3-5 times/week	2681 (24.6%)	15.7	2102 (24.8%)	
Ulcer	44	Self-reported physical activity, n(%)	Daily	2871 (26.4%)	15.7	2267 (26.7%)	
Ulcer	44	Sex, n(%)	Males	7849 (60.9%)	0.1	5281 (62.3%)	n=12912 vs n=8475
Ulcer	44	Sex, n(%)	Females	5046 (39.1%)	0.1	3194 (37.7%)	
Ulcer	44	Smoking status, n(%)	No	9153 (78.4%)	9.6	6715 (79.2%)	
Ulcer	44	Smoking status, n(%)	Yes	2523 (21.6%)	9.6	1760 (20.8%)	
Ulcer	44	Stroke, n(%)	No	11300 (98.6%)	11.3	8357 (98.6%)	
Ulcer	44	Stroke, n(%)	Yes	159 (1.4%)	11.3	118 (1.4%)	
Ulcer	44	Systolic blood pressure, mean(SD)		128.7 (14.6)	4.8	128.5 (14.4)	
Ulcer	44	Time since last healthcare visit, mean(SD)		0.8 (1.3)	0	0.7 (1)	
Ulcer	44	Total cholesterol, mean(SD)		4.9 (1.2)	13.3	4.8 (1.2)	
Ulcer	45	Age at diagnosis of type 2 diabetes, mean(SD)		41.4 (2.7)	0	40.9 (2.7)	
Ulcer	45	BMI, mean(SD)		33 (6.5)	9.4	32.9 (6.5)	

Ulcer	45	HbA1c, mean(SD)		52.5 (15.9)	3.1	52.5 (15.5)	
Ulcer	45	HDL-cholesterol, mean(SD)		1.1 (0.3)	16.8	1.1 (0.3)	
Ulcer	45	Ischemic heart disease, n(%)	No	12618 (96.3%)	10.8	9331 (96.4%)	
Ulcer	45	Ischemic heart disease, n(%)	Yes	490 (3.7%)	10.8	349 (3.6%)	
Ulcer	45	Neuropathy, n(%)	No	13612 (92.7%)	0	8786 (90.8%)	
Ulcer	45	Neuropathy, n(%)	Yes	1075 (7.3%)	0	894 (9.2%)	
Ulcer	45	Self-reported physical activity, n(%)	Never	1814 (14.6%)	15.5	1393 (14.4%)	
Ulcer	45	Self-reported physical activity, n(%)	<1 times/week	1665 (13.4%)	15.5	1262 (13%)	
Ulcer	45	Self-reported physical activity, n(%)	regularly - 1-2 times/week	2603 (21%)	15.5	2039 (21.1%)	
Ulcer	45	Self-reported physical activity, n(%)	regularly - 3-5 times/week	3066 (24.7%)	15.5	2423 (25%)	
Ulcer	45	Self-reported physical activity, n(%)	Daily	3260 (26.3%)	15.5	2563 (26.5%)	
Ulcer	45	Sex, n(%)	Males	9082 (61.9%)	0.1	6048 (62.5%)	n=14687 vs n=9680
Ulcer	45	Sex, n(%)	Females	5588 (38.1%)	0.1	3632 (37.5%)	
Ulcer	45	Smoking status, n(%)	No	10414 (78.3%)	9.5	7682 (79.4%)	
Ulcer	45	Smoking status, n(%)	Yes	2880 (21.7%)	9.5	1998 (20.6%)	
Ulcer	45	Stroke, n(%)	No	12869 (98.5%)	11.1	9542 (98.6%)	
Ulcer	45	Stroke, n(%)	Yes	192 (1.5%)	11.1	138 (1.4%)	
Ulcer	45	Systolic blood pressure, mean(SD)		129.4 (14.8)	4.6	129.1 (14.5)	
Ulcer	45	Time since last healthcare visit, mean(SD)		0.8 (1.3)	0	0.7 (1)	
Ulcer	45	Total cholesterol, mean(SD)		4.9 (1.2)	12.7	4.8 (1.1)	
Ulcer	46	Age at diagnosis of type 2 diabetes, mean(SD)		42.4 (2.7)	0	41.9 (2.7)	
Ulcer	46	BMI, mean(SD)		32.9 (6.4)	9.2	32.8 (6.3)	
Ulcer	46	HbA1c, mean(SD)		52.6 (16)	2.9	52.6 (15.5)	

Ulcer	46	HDL-cholesterol, mean(SD)		1.1 (0.3)	16.4	1.1 (0.3)	
Ulcer	46	Ischemic heart disease, n(%)	No	14199 (95.7%)	10.5	10548 (95.8%)	
Ulcer	46	Ischemic heart disease, n(%)	Yes	633 (4.3%)	10.5	464 (4.2%)	
Ulcer	46	Neuropathy, n(%)	No	15298 (92.3%)	0	9952 (90.4%)	
Ulcer	46	Neuropathy, n(%)	Yes	1269 (7.7%)	0	1060 (9.6%)	
Ulcer	46	Self-reported physical activity, n(%)	Never	2068 (14.7%)	15.1	1599 (14.5%)	
Ulcer	46	Self-reported physical activity, n(%)	<1 times/week	1940 (13.8%)	15.1	1487 (13.5%)	
Ulcer	46	Self-reported physical activity, n(%)	regularly - 1-2 times/week	2980 (21.2%)	15.1	2351 (21.3%)	
Ulcer	46	Self-reported physical activity, n(%)	regularly - 3-5 times/week	3451 (24.5%)	15.1	2735 (24.8%)	
Ulcer	46	Self-reported physical activity, n(%)	Daily	3619 (25.7%)	15.1	2840 (25.8%)	
Ulcer	46	Sex, n(%)	Males	10345 (62.5%)	0.1	6954 (63.1%)	n=16567 vs n=11012
Ulcer	46	Sex, n(%)	Females	6206 (37.5%)	0.1	4058 (36.9%)	
Ulcer	46	Smoking status, n(%)	No	11770 (78.2%)	9.2	8735 (79.3%)	
Ulcer	46	Smoking status, n(%)	Yes	3280 (21.8%)	9.2	2277 (20.7%)	
Ulcer	46	Stroke, n(%)	No	14512 (98.2%)	10.8	10816 (98.2%)	
Ulcer	46	Stroke, n(%)	Yes	262 (1.8%)	10.8	196 (1.8%)	
Ulcer	46	Systolic blood pressure, mean(SD)		129.7 (14.8)	4.3	129.4 (14.4)	
Ulcer	46	Time since last healthcare visit, mean(SD)		0.8 (1.3)	0	0.6 (1)	
Ulcer	46	Total cholesterol, mean(SD)		4.9 (1.2)	11.9	4.8 (1.1)	
Ulcer	47	Age at diagnosis of type 2 diabetes, mean(SD)		43.3 (2.7)	0	42.8 (2.7)	
Ulcer	47	BMI, mean(SD)		32.8 (6.3)	9	32.7 (6.3)	
Ulcer	47	HbA1c, mean(SD)		52.5 (15.7)	2.5	52.6 (15.2)	
Ulcer	47	HDL-cholesterol, mean(SD)		1.1 (0.3)	15.7	1.1 (0.3)	

Ulcer	47	Ischemic heart disease, n(%)	No	15893 (95.2%)	10.1	11929 (95.5%)	
Ulcer	47	Ischemic heart disease, n(%)	Yes	794 (4.8%)	10.1	557 (4.5%)	
Ulcer	47	Neuropathy, n(%)	No	17097 (92.2%)	0	11262 (90.2%)	
Ulcer	47	Neuropathy, n(%)	Yes	1456 (7.8%)	0	1224 (9.8%)	
Ulcer	47	Self-reported physical activity, n(%)	Never	2292 (14.5%)	14.8	1801 (14.4%)	
Ulcer	47	Self-reported physical activity, n(%)	<1 times/week	2181 (13.8%)	14.8	1673 (13.4%)	
Ulcer	47	Self-reported physical activity, n(%)	regularly - 1-2 times/week	3334 (21.1%)	14.8	2634 (21.1%)	
Ulcer	47	Self-reported physical activity, n(%)	regularly - 3-5 times/week	3868 (24.5%)	14.8	3089 (24.7%)	
Ulcer	47	Self-reported physical activity, n(%)	Daily	4129 (26.1%)	14.8	3289 (26.3%)	
Ulcer	47	Sex, n(%)	Males	11662 (62.9%)	0.1	7964 (63.8%)	n=18553 vs n=12486
Ulcer	47	Sex, n(%)	Females	6875 (37.1%)	0.1	4522 (36.2%)	
Ulcer	47	Smoking status, n(%)	No	13206 (78.1%)	8.9	9877 (79.1%)	
Ulcer	47	Smoking status, n(%)	Yes	3705 (21.9%)	8.9	2609 (20.9%)	
Ulcer	47	Stroke, n(%)	No	16309 (98%)	10.3	12250 (98.1%)	
Ulcer	47	Stroke, n(%)	Yes	326 (2%)	10.3	236 (1.9%)	
Ulcer	47	Systolic blood pressure, mean(SD)		129.9 (14.7)	4	129.5 (14.4)	
Ulcer	47	Time since last healthcare visit, mean(SD)		0.8 (1.3)	0	0.7 (1)	
Ulcer	47	Total cholesterol, mean(SD)		4.9 (1.1)	11.3	4.8 (1.1)	
Ulcer	48	Age at diagnosis of type 2 diabetes, mean(SD)		44.3 (2.7)	0	43.7 (2.7)	
Ulcer	48	BMI, mean(SD)		32.7 (6.3)	8.7	32.6 (6.2)	
Ulcer	48	HbA1c, mean(SD)		52.5 (15.5)	2.6	52.6 (15.2)	
Ulcer	48	HDL-cholesterol, mean(SD)		1.1 (0.3)	15.6	1.1 (0.3)	
Ulcer	48	Ischemic heart disease, n(%)	No	17474 (95%)	10	13219 (95.3%)	

Ulcer	48	Ischemic heart disease, n(%)	Yes	924 (5%)	10	657 (4.7%)	
Ulcer	48	Neuropathy, n(%)	No	18727 (91.6%)	0	12462 (89.8%)	
Ulcer	48	Neuropathy, n(%)	Yes	1711 (8.4%)	0	1414 (10.2%)	
Ulcer	48	Self-reported physical activity, n(%)	Never	2541 (14.5%)	14.3	2026 (14.6%)	
Ulcer	48	Self-reported physical activity, n(%)	<1 times/week	2394 (13.7%)	14.3	1814 (13.1%)	
Ulcer	48	Self-reported physical activity, n(%)	regularly - 1-2 times/week	3708 (21.2%)	14.3	2937 (21.2%)	
Ulcer	48	Self-reported physical activity, n(%)	regularly - 3-5 times/week	4298 (24.5%)	14.3	3450 (24.9%)	
Ulcer	48	Self-reported physical activity, n(%)	Daily	4569 (26.1%)	14.3	3649 (26.3%)	
Ulcer	48	Sex, n(%)	Males	12802 (62.7%)	0.1	8840 (63.7%)	n=20438 vs n=13876
Ulcer	48	Sex, n(%)	Females	7615 (37.3%)	0.1	5036 (36.3%)	
Ulcer	48	Smoking status, n(%)	No	14674 (78.4%)	8.4	11017 (79.4%)	
Ulcer	48	Smoking status, n(%)	Yes	4043 (21.6%)	8.4	2859 (20.6%)	
Ulcer	48	Stroke, n(%)	No	17974 (98%)	10.2	13604 (98%)	
Ulcer	48	Stroke, n(%)	Yes	374 (2%)	10.2	272 (2%)	
Ulcer	48	Systolic blood pressure, mean(SD)		130.4 (14.7)	3.9	130.1 (14.3)	
Ulcer	48	Time since last healthcare visit, mean(SD)		0.8 (1.2)	0	0.6 (1)	
Ulcer	48	Total cholesterol, mean(SD)		4.9 (1.1)	11.4	4.8 (1.1)	
Ulcer	49	Age at diagnosis of type 2 diabetes, mean(SD)		45.2 (2.7)	0	44.7 (2.7)	
Ulcer	49	BMI, mean(SD)		32.5 (6.1)	8.8	32.4 (6)	
Ulcer	49	HbA1c, mean(SD)		52.5 (15.4)	2.5	52.5 (15)	
Ulcer	49	HDL-cholesterol, mean(SD)		1.1 (0.3)	15.5	1.1 (0.3)	
Ulcer	49	Ischemic heart disease, n(%)	No	19406 (94.5%)	9.6	14724 (94.8%)	
Ulcer	49	Ischemic heart disease, n(%)	Yes	1139 (5.5%)	9.6	815 (5.2%)	

Ulcer	49	Neuropathy, n(%)	No	20738 (91.3%)	0	13894 (89.4%)	
Ulcer	49	Neuropathy, n(%)	Yes	1977 (8.7%)	0	1645 (10.6%)	
Ulcer	49	Self-reported physical activity, n(%)	Never	2869 (14.6%)	13.7	2298 (14.8%)	
Ulcer	49	Self-reported physical activity, n(%)	<1 times/week	2632 (13.4%)	13.7	2004 (12.9%)	
Ulcer	49	Self-reported physical activity, n(%)	regularly - 1-2 times/week	4116 (21%)	13.7	3261 (21%)	
Ulcer	49	Self-reported physical activity, n(%)	regularly - 3-5 times/week	4690 (23.9%)	13.7	3747 (24.1%)	
Ulcer	49	Self-reported physical activity, n(%)	Daily	5298 (27%)	13.7	4229 (27.2%)	
Ulcer	49	Sex, n(%)	Males	14253 (62.8%)	0.1	9927 (63.9%)	n=22715 vs n=15539
Ulcer	49	Sex, n(%)	Females	8442 (37.2%)	0.1	5612 (36.1%)	
Ulcer	49	Smoking status, n(%)	No	16230 (78%)	8.4	12288 (79.1%)	
Ulcer	49	Smoking status, n(%)	Yes	4586 (22%)	8.4	3251 (20.9%)	
Ulcer	49	Stroke, n(%)	No	20033 (97.9%)	9.9	15238 (98.1%)	
Ulcer	49	Stroke, n(%)	Yes	431 (2.1%)	9.9	301 (1.9%)	
Ulcer	49	Systolic blood pressure, mean(SD)		130.8 (14.8)	3.8	130.5 (14.5)	
Ulcer	49	Time since last healthcare visit, mean(SD)		0.8 (1.2)	0	0.6 (1)	
Ulcer	49	Total cholesterol, mean(SD)		4.9 (1.2)	11.3	4.8 (1.1)	
Ulcer	50	Age at diagnosis of type 2 diabetes, mean(SD)		46.2 (2.8)	0	45.7 (2.8)	
Ulcer	50	BMI, mean(SD)		32.4 (6.1)	8.8	32.3 (6)	
Ulcer	50	HbA1c, mean(SD)		52.4 (15.1)	2.4	52.5 (14.8)	
Ulcer	50	HDL-cholesterol, mean(SD)		1.1 (0.3)	15.2	1.1 (0.3)	
Ulcer	50	Ischemic heart disease, n(%)	No	21462 (93.9%)	9.6	16336 (94.2%)	
Ulcer	50	Ischemic heart disease, n(%)	Yes	1384 (6.1%)	9.6	1004 (5.8%)	
Ulcer	50	Neuropathy, n(%)	No	22986 (90.9%)	0	15421 (88.9%)	

Ulcer	50	Neuropathy, n(%)	Yes	2293 (9.1%)	0	1919 (11.1%)	
Ulcer	50	Self-reported physical activity, n(%)	Never	3195 (14.7%)	13.7	2565 (14.8%)	
Ulcer	50	Self-reported physical activity, n(%)	<1 times/week	2959 (13.6%)	13.7	2283 (13.2%)	
Ulcer	50	Self-reported physical activity, n(%)	regularly - 1-2 times/week	4475 (20.5%)	13.7	3562 (20.5%)	
Ulcer	50	Self-reported physical activity, n(%)	regularly - 3-5 times/week	5278 (24.2%)	13.7	4220 (24.3%)	
Ulcer	50	Self-reported physical activity, n(%)	Daily	5899 (27.1%)	13.7	4710 (27.2%)	
Ulcer	50	Sex, n(%)	Males	15898 (63%)	0.1	11096 (64%)	n=25279 vs n=17340
Ulcer	50	Sex, n(%)	Females	9356 (37%)	0.1	6244 (36%)	
Ulcer	50	Smoking status, n(%)	No	18067 (77.9%)	8.3	13682 (78.9%)	
Ulcer	50	Smoking status, n(%)	Yes	5114 (22.1%)	8.3	3658 (21.1%)	
Ulcer	50	Stroke, n(%)	No	22269 (97.8%)	9.9	16975 (97.9%)	
Ulcer	50	Stroke, n(%)	Yes	511 (2.2%)	9.9	365 (2.1%)	
Ulcer	50	Systolic blood pressure, mean(SD)		131.3 (14.9)	3.8	131 (14.6)	
Ulcer	50	Time since last healthcare visit, mean(SD)		0.7 (1.2)	0	0.6 (1)	
Ulcer	50	Total cholesterol, mean(SD)		4.9 (1.2)	11.1	4.8 (1.1)	
Ulcer	51	Age at diagnosis of type 2 diabetes, mean(SD)		47.2 (2.8)	0	46.6 (2.8)	
Ulcer	51	BMI, mean(SD)		32.3 (6)	8.4	32.2 (5.9)	
Ulcer	51	HbA1c, mean(SD)		52.4 (14.9)	2.2	52.4 (14.5)	
Ulcer	51	HDL-cholesterol, mean(SD)		1.1 (0.3)	14.3	1.1 (0.3)	
Ulcer	51	Ischemic heart disease, n(%)	No	23588 (93.4%)	9.2	18125 (93.7%)	
Ulcer	51	Ischemic heart disease, n(%)	Yes	1675 (6.6%)	9.2	1224 (6.3%)	
Ulcer	51	Neuropathy, n(%)	No	25102 (90.2%)	0	17046 (88.1%)	
Ulcer	51	Neuropathy, n(%)	Yes	2734 (9.8%)	0	2303 (11.9%)	

Ulcer	51	Self-reported physical activity, n(%)	Never	3490 (14.5%)	13.3	2805 (14.5%)	
Ulcer	51	Self-reported physical activity, n(%)	<1 times/week	3289 (13.6%)	13.3	2582 (13.3%)	
Ulcer	51	Self-reported physical activity, n(%)	regularly - 1-2 times/week	4932 (20.4%)	13.3	3949 (20.4%)	
Ulcer	51	Self-reported physical activity, n(%)	regularly - 3-5 times/week	5957 (24.7%)	13.3	4835 (25%)	
Ulcer	51	Self-reported physical activity, n(%)	Daily	6460 (26.8%)	13.3	5178 (26.8%)	
Ulcer	51	Sex, n(%)	Males	17489 (62.9%)	0.1	12343 (63.8%)	n=27836 vs n=19349
Ulcer	51	Sex, n(%)	Females	10315 (37.1%)	0.1	7006 (36.2%)	
Ulcer	51	Smoking status, n(%)	No	19913 (77.8%)	8	15227 (78.7%)	
Ulcer	51	Smoking status, n(%)	Yes	5695 (22.2%)	8	4122 (21.3%)	
Ulcer	51	Stroke, n(%)	No	24588 (97.6%)	9.5	18908 (97.7%)	
Ulcer	51	Stroke, n(%)	Yes	605 (2.4%)	9.5	441 (2.3%)	
Ulcer	51	Systolic blood pressure, mean(SD)		131.7 (15)	3.5	131.4 (14.7)	
Ulcer	51	Time since last healthcare visit, mean(SD)		0.7 (1.2)	0	0.6 (0.9)	
Ulcer	51	Total cholesterol, mean(SD)		4.9 (1.2)	10.4	4.8 (1.1)	
Ulcer	52	Age at diagnosis of type 2 diabetes, mean(SD)		48.1 (2.8)	0	47.6 (2.8)	
Ulcer	52	BMI, mean(SD)		32.2 (5.9)	8.3	32.1 (5.8)	
Ulcer	52	HbA1c, mean(SD)		52.5 (14.9)	2.2	52.5 (14.4)	
Ulcer	52	HDL-cholesterol, mean(SD)		1.1 (0.3)	14	1.2 (0.3)	
Ulcer	52	Ischemic heart disease, n(%)	No	25380 (93%)	9.1	19591 (93.3%)	
Ulcer	52	Ischemic heart disease, n(%)	Yes	1898 (7%)	9.1	1397 (6.7%)	
Ulcer	52	Neuropathy, n(%)	No	26917 (89.7%)	0	18348 (87.4%)	
Ulcer	52	Neuropathy, n(%)	Yes	3101 (10.3%)	0	2640 (12.6%)	
Ulcer	52	Self-reported physical activity, n(%)	Never	3774 (14.4%)	13	3022 (14.4%)	

Ulcer	52	Self-reported physical activity, n(%)	<1 times/week	3517 (13.5%)	13	2752 (13.1%)	
Ulcer	52	Self-reported physical activity, n(%)	regularly - 1-2 times/week	5339 (20.4%)	13	4237 (20.2%)	
Ulcer	52	Self-reported physical activity, n(%)	regularly - 3-5 times/week	6312 (24.2%)	13	5148 (24.5%)	
Ulcer	52	Self-reported physical activity, n(%)	Daily	7181 (27.5%)	13	5829 (27.8%)	
Ulcer	52	Sex, n(%)	Males	18814 (62.7%)	0.1	13299 (63.4%)	n=30018 vs n=20988
Ulcer	52	Sex, n(%)	Females	11170 (37.3%)	0.1	7689 (36.6%)	
Ulcer	52	Smoking status, n(%)	No	21441 (77.5%)	7.8	16457 (78.4%)	
Ulcer	52	Smoking status, n(%)	Yes	6223 (22.5%)	7.8	4531 (21.6%)	
Ulcer	52	Stroke, n(%)	No	26465 (97.4%)	9.5	20474 (97.6%)	
Ulcer	52	Stroke, n(%)	Yes	706 (2.6%)	9.5	514 (2.4%)	
Ulcer	52	Systolic blood pressure, mean(SD)		132 (15)	3.3	131.8 (14.7)	
Ulcer	52	Time since last healthcare visit, mean(SD)		0.7 (1.2)	0	0.6 (0.9)	
Ulcer	52	Total cholesterol, mean(SD)		4.9 (1.2)	10.1	4.8 (1.1)	
Ulcer	53	Age at diagnosis of type 2 diabetes, mean(SD)		49.1 (2.8)	0	48.5 (2.8)	
Ulcer	53	BMI, mean(SD)		32 (5.9)	8.4	31.9 (5.8)	
Ulcer	53	HbA1c, mean(SD)		52.3 (14.7)	2.4	52.4 (14.4)	
Ulcer	53	HDL-cholesterol, mean(SD)		1.2 (0.3)	14.3	1.2 (0.3)	
Ulcer	53	Ischemic heart disease, n(%)	No	26887 (92.5%)	9.2	20798 (92.8%)	
Ulcer	53	Ischemic heart disease, n(%)	Yes	2183 (7.5%)	9.2	1618 (7.2%)	
Ulcer	53	Neuropathy, n(%)	No	28499 (89%)	0	19433 (86.7%)	
Ulcer	53	Neuropathy, n(%)	Yes	3510 (11%)	0	2983 (13.3%)	
Ulcer	53	Self-reported physical activity, n(%)	Never	3970 (14.3%)	13	3169 (14.1%)	
Ulcer	53	Self-reported physical activity, n(%)	<1 times/week	3697 (13.3%)	13	2934 (13.1%)	

Ulcer	53	Self-reported physical activity, n(%)	regularly - 1-2 times/week	5715 (20.5%)	13	4577 (20.4%)	
Ulcer	53	Self-reported physical activity, n(%)	regularly - 3-5 times/week	6737 (24.2%)	13	5510 (24.6%)	
Ulcer	53	Self-reported physical activity, n(%)	Daily	7734 (27.8%)	13	6226 (27.8%)	
Ulcer	53	Sex, n(%)	Males	19974 (62.5%)	0.1	14158 (63.2%)	n=32009 vs n=22416
Ulcer	53	Sex, n(%)	Females	11997 (37.5%)	0.1	8258 (36.8%)	
Ulcer	53	Smoking status, n(%)	No	22777 (77.4%)	8.1	17552 (78.3%)	
Ulcer	53	Smoking status, n(%)	Yes	6653 (22.6%)	8.1	4864 (21.7%)	
Ulcer	53	Stroke, n(%)	No	28147 (97.1%)	9.5	21805 (97.3%)	
Ulcer	53	Stroke, n(%)	Yes	826 (2.9%)	9.5	611 (2.7%)	
Ulcer	53	Systolic blood pressure, mean(SD)		132.2 (14.8)	3.4	131.9 (14.6)	
Ulcer	53	Time since last healthcare visit, mean(SD)		0.7 (1.2)	0	0.6 (0.9)	
Ulcer	53	Total cholesterol, mean(SD)		4.9 (1.2)	10.4	4.8 (1.2)	
Ulcer	54	Age at diagnosis of type 2 diabetes, mean(SD)		50 (2.8)	0	49.5 (2.8)	
Ulcer	54	BMI, mean(SD)		31.9 (5.8)	8.2	31.8 (5.7)	
Ulcer	54	HbA1c, mean(SD)		52.3 (14.6)	2.4	52.4 (14.2)	
Ulcer	54	HDL-cholesterol, mean(SD)		1.2 (0.3)	14.2	1.2 (0.3)	
Ulcer	54	Ischemic heart disease, n(%)	No	28409 (91.8%)	8.9	22012 (92.1%)	
Ulcer	54	Ischemic heart disease, n(%)	Yes	2526 (8.2%)	8.9	1898 (7.9%)	
Ulcer	54	Neuropathy, n(%)	No	30062 (88.6%)	0	20632 (86.3%)	
Ulcer	54	Neuropathy, n(%)	Yes	3883 (11.4%)	0	3278 (13.7%)	
Ulcer	54	Self-reported physical activity, n(%)	Never	4091 (13.8%)	12.9	3263 (13.6%)	
Ulcer	54	Self-reported physical activity, n(%)	<1 times/week	3955 (13.4%)	12.9	3123 (13.1%)	
Ulcer	54	Self-reported physical activity, n(%)	regularly - 1-2 times/week	6019 (20.4%)	12.9	4855 (20.3%)	

Ulcer	54	Self-reported physical activity, n(%)	regularly - 3-5 times/week	7200 (24.3%)	12.9	5894 (24.7%)	
Ulcer	54	Self-reported physical activity, n(%)	Daily	8310 (28.1%)	12.9	6775 (28.3%)	
Ulcer	54	Sex, n(%)	Males	21112 (62.3%)	0.1	15019 (62.8%)	n=33945 vs n=23910
Ulcer	54	Sex, n(%)	Females	12799 (37.7%)	0.1	8891 (37.2%)	
Ulcer	54	Smoking status, n(%)	No	24211 (77.3%)	7.7	18685 (78.1%)	
Ulcer	54	Smoking status, n(%)	Yes	7108 (22.7%)	7.7	5225 (21.9%)	
Ulcer	54	Stroke, n(%)	No	29891 (97%)	9.2	23212 (97.1%)	
Ulcer	54	Stroke, n(%)	Yes	927 (3%)	9.2	698 (2.9%)	
Ulcer	54	Systolic blood pressure, mean(SD)		132.4 (14.9)	3.3	132.1 (14.6)	
Ulcer	54	Time since last healthcare visit, mean(SD)		0.7 (1.2)	0	0.6 (0.9)	
Ulcer	54	Total cholesterol, mean(SD)		4.9 (1.2)	10.2	4.8 (1.2)	
Ulcer	55	Age at diagnosis of type 2 diabetes, mean(SD)		51 (2.8)	0	50.5 (2.8)	
Ulcer	55	BMI, mean(SD)		31.7 (5.7)	8.3	31.6 (5.6)	
Ulcer	55	HbA1c, mean(SD)		52.1 (14.4)	2.4	52.2 (13.9)	
Ulcer	55	HDL-cholesterol, mean(SD)		1.2 (0.3)	14.2	1.2 (0.3)	
Ulcer	55	Ischemic heart disease, n(%)	No	29797 (91.1%)	9	23117 (91.5%)	
Ulcer	55	Ischemic heart disease, n(%)	Yes	2903 (8.9%)	9	2158 (8.5%)	
Ulcer	55	Neuropathy, n(%)	No	31653 (88.1%)	0	21644 (85.6%)	
Ulcer	55	Neuropathy, n(%)	Yes	4264 (11.9%)	0	3631 (14.4%)	
Ulcer	55	Self-reported physical activity, n(%)	Never	4283 (13.7%)	12.8	3427 (13.6%)	
Ulcer	55	Self-reported physical activity, n(%)	<1 times/week	4178 (13.3%)	12.8	3307 (13.1%)	
Ulcer	55	Self-reported physical activity, n(%)	regularly - 1-2 times/week	6463 (20.6%)	12.8	5166 (20.4%)	
Ulcer	55	Self-reported physical activity, n(%)	regularly - 3-5 times/week	7589 (24.2%)	12.8	6164 (24.4%)	

Ulcer	55	Self-reported physical activity, n(%)	Daily	8811 (28.1%)	12.8	7211 (28.5%)	
Ulcer	55	Sex, n(%)	Males	22303 (62.2%)	0.1	15792 (62.5%)	n=35917 vs n=25275
Ulcer	55	Sex, n(%)	Females	13575 (37.8%)	0.1	9483 (37.5%)	
Ulcer	55	Smoking status, n(%)	No	25650 (77.2%)	7.6	19732 (78.1%)	
Ulcer	55	Smoking status, n(%)	Yes	7554 (22.8%)	7.6	5543 (21.9%)	
Ulcer	55	Stroke, n(%)	No	31506 (96.7%)	9.3	24460 (96.8%)	
Ulcer	55	Stroke, n(%)	Yes	1063 (3.3%)	9.3	815 (3.2%)	
Ulcer	55	Systolic blood pressure, mean(SD)		132.8 (14.9)	3.4	132.4 (14.7)	
Ulcer	55	Time since last healthcare visit, mean(SD)		0.7 (1.2)	0	0.6 (0.9)	
Ulcer	55	Total cholesterol, mean(SD)		4.9 (1.2)	10.1	4.8 (1.2)	
Ulcer	56	Age at diagnosis of type 2 diabetes, mean(SD)		52 (2.8)	0	51.5 (2.8)	
Ulcer	56	BMI, mean(SD)		31.5 (5.7)	8.3	31.5 (5.6)	
Ulcer	56	HbA1c, mean(SD)		51.8 (13.9)	2.2	51.8 (13.4)	
Ulcer	56	HDL-cholesterol, mean(SD)		1.2 (0.4)	14	1.2 (0.3)	
Ulcer	56	Ischemic heart disease, n(%)	No	30963 (90.3%)	9	24130 (90.6%)	
Ulcer	56	Ischemic heart disease, n(%)	Yes	3339 (9.7%)	9	2512 (9.4%)	
Ulcer	56	Neuropathy, n(%)	No	33141 (87.9%)	0	22782 (85.5%)	
Ulcer	56	Neuropathy, n(%)	Yes	4565 (12.1%)	0	3860 (14.5%)	
Ulcer	56	Self-reported physical activity, n(%)	Never	4534 (13.8%)	12.6	3641 (13.7%)	
Ulcer	56	Self-reported physical activity, n(%)	<1 times/week	4324 (13.1%)	12.6	3389 (12.7%)	
Ulcer	56	Self-reported physical activity, n(%)	regularly - 1-2 times/week	6880 (20.9%)	12.6	5553 (20.8%)	
Ulcer	56	Self-reported physical activity, n(%)	regularly - 3-5 times/week	8026 (24.3%)	12.6	6573 (24.7%)	
Ulcer	56	Self-reported physical activity, n(%)	Daily	9198 (27.9%)	12.6	7486 (28.1%)	

Ulcer	56	Sex, n(%)	Males	23387 (62.1%)	0.1	16641 (62.5%)	n=37706 vs n=26642
Ulcer	56	Sex, n(%)	Females	14274 (37.9%)	0.1	10001 (37.5%)	
Ulcer	56	Smoking status, n(%)	No	26906 (77.2%)	7.6	20773 (78%)	
Ulcer	56	Smoking status, n(%)	Yes	7943 (22.8%)	7.6	5869 (22%)	
Ulcer	56	Stroke, n(%)	No	32940 (96.5%)	9.4	25720 (96.5%)	
Ulcer	56	Stroke, n(%)	Yes	1207 (3.5%)	9.4	922 (3.5%)	
Ulcer	56	Systolic blood pressure, mean(SD)		132.9 (14.8)	3.2	132.6 (14.6)	
Ulcer	56	Time since last healthcare visit, mean(SD)		0.7 (1.2)	0	0.6 (0.9)	
Ulcer	56	Total cholesterol, mean(SD)		4.9 (1.2)	10	4.8 (1.2)	
Ulcer	57	Age at diagnosis of type 2 diabetes, mean(SD)		53 (2.8)	0	52.4 (2.8)	
Ulcer	57	BMI, mean(SD)		31.4 (5.6)	8.2	31.4 (5.5)	
Ulcer	57	HbA1c, mean(SD)		51.7 (13.7)	2.2	51.7 (13.3)	
Ulcer	57	HDL-cholesterol, mean(SD)		1.2 (0.4)	13.7	1.2 (0.4)	
Ulcer	57	Ischemic heart disease, n(%)	No	32227 (89.4%)	8.9	25211 (89.8%)	
Ulcer	57	Ischemic heart disease, n(%)	Yes	3810 (10.6%)	8.9	2878 (10.2%)	
Ulcer	57	Neuropathy, n(%)	No	34589 (87.4%)	0	23889 (85%)	
Ulcer	57	Neuropathy, n(%)	Yes	4973 (12.6%)	0	4200 (15%)	
Ulcer	57	Self-reported physical activity, n(%)	Never	4639 (13.4%)	12.5	3734 (13.3%)	
Ulcer	57	Self-reported physical activity, n(%)	<1 times/week	4533 (13.1%)	12.5	3612 (12.9%)	
Ulcer	57	Self-reported physical activity, n(%)	regularly - 1-2 times/week	7192 (20.8%)	12.5	5885 (21%)	
Ulcer	57	Self-reported physical activity, n(%)	regularly - 3-5 times/week	8363 (24.2%)	12.5	6813 (24.3%)	
Ulcer	57	Self-reported physical activity, n(%)	Daily	9897 (28.6%)	12.5	8045 (28.6%)	
Ulcer	57	Sex, n(%)	Males	24540 (62.1%)	0.1	17483 (62.2%)	n=39562 vs n=28089

Ulcer	57	Sex, n(%)	Females	14972 (37.9%)	0.1	10606 (37.8%)	
Ulcer	57	Smoking status, n(%)	No	28557 (77.9%)	7.3	22068 (78.6%)	
Ulcer	57	Smoking status, n(%)	Yes	8106 (22.1%)	7.3	6021 (21.4%)	
Ulcer	57	Stroke, n(%)	No	34539 (96.3%)	9.4	27081 (96.4%)	
Ulcer	57	Stroke, n(%)	Yes	1320 (3.7%)	9.4	1008 (3.6%)	
Ulcer	57	Systolic blood pressure, mean(SD)		133.3 (15.1)	3.2	132.9 (14.8)	
Ulcer	57	Time since last healthcare visit, mean(SD)		0.7 (1.2)	0	0.6 (0.9)	
Ulcer	57	Total cholesterol, mean(SD)		4.9 (1.2)	9.8	4.8 (1.2)	
Ulcer	58	Age at diagnosis of type 2 diabetes, mean(SD)		53.9 (2.9)	0	53.4 (2.8)	
Ulcer	58	BMI, mean(SD)		31.3 (5.5)	8	31.2 (5.5)	
Ulcer	58	HbA1c, mean(SD)		51.5 (13.5)	2.2	51.5 (13)	
Ulcer	58	HDL-cholesterol, mean(SD)		1.2 (0.4)	13.5	1.2 (0.4)	
Ulcer	58	Ischemic heart disease, n(%)	No	33773 (88.9%)	8.6	26541 (89.3%)	
Ulcer	58	Ischemic heart disease, n(%)	Yes	4227 (11.1%)	8.6	3192 (10.7%)	
Ulcer	58	Neuropathy, n(%)	No	36263 (87.2%)	0	25191 (84.7%)	
Ulcer	58	Neuropathy, n(%)	Yes	5312 (12.8%)	0	4542 (15.3%)	
Ulcer	58	Self-reported physical activity, n(%)	Never	4798 (13.2%)	12.4	3881 (13.1%)	
Ulcer	58	Self-reported physical activity, n(%)	<1 times/week	4645 (12.8%)	12.4	3726 (12.5%)	
Ulcer	58	Self-reported physical activity, n(%)	regularly - 1-2 times/week	7650 (21%)	12.4	6238 (21%)	
Ulcer	58	Self-reported physical activity, n(%)	regularly - 3-5 times/week	8780 (24.1%)	12.4	7224 (24.3%)	
Ulcer	58	Self-reported physical activity, n(%)	Daily	10542 (28.9%)	12.4	8664 (29.1%)	
Ulcer	58	Sex, n(%)	Males	25695 (61.9%)	0.1	18508 (62.2%)	n=41575 vs n=29733
Ulcer	58	Sex, n(%)	Females	15823 (38.1%)	0.1	11225 (37.8%)	

Ulcer	58	Smoking status, n(%)	No	30305 (78.5%)	7.1	23548 (79.2%)	
Ulcer	58	Smoking status, n(%)	Yes	8305 (21.5%)	7.1	6185 (20.8%)	
Ulcer	58	Stroke, n(%)	No	36329 (96.1%)	9	28599 (96.2%)	
Ulcer	58	Stroke, n(%)	Yes	1493 (3.9%)	9	1134 (3.8%)	
Ulcer	58	Systolic blood pressure, mean(SD)		133.5 (15)	3.1	133.2 (14.7)	
Ulcer	58	Time since last healthcare visit, mean(SD)		0.7 (1.1)	0	0.6 (0.9)	
Ulcer	58	Total cholesterol, mean(SD)		4.9 (1.2)	9.6	4.8 (1.2)	
Ulcer	59	Age at diagnosis of type 2 diabetes, mean(SD)		54.9 (2.9)	0	54.4 (2.8)	
Ulcer	59	BMI, mean(SD)		31.1 (5.4)	8	31.1 (5.4)	
Ulcer	59	HbA1c, mean(SD)		51.3 (13.1)	2.1	51.4 (12.6)	
Ulcer	59	HDL-cholesterol, mean(SD)		1.2 (0.4)	13.3	1.2 (0.4)	
Ulcer	59	Ischemic heart disease, n(%)	No	35442 (88.1%)	8.5	27921 (88.5%)	
Ulcer	59	Ischemic heart disease, n(%)	Yes	4796 (11.9%)	8.5	3617 (11.5%)	
Ulcer	59	Neuropathy, n(%)	No	38139 (86.7%)	0	26542 (84.2%)	
Ulcer	59	Neuropathy, n(%)	Yes	5853 (13.3%)	0	4996 (15.8%)	
Ulcer	59	Self-reported physical activity, n(%)	Never	5030 (13%)	12.1	4077 (12.9%)	
Ulcer	59	Self-reported physical activity, n(%)	<1 times/week	4954 (12.8%)	12.1	3974 (12.6%)	
Ulcer	59	Self-reported physical activity, n(%)	regularly - 1-2 times/week	8102 (21%)	12.1	6571 (20.8%)	
Ulcer	59	Self-reported physical activity, n(%)	regularly - 3-5 times/week	9305 (24.1%)	12.1	7618 (24.2%)	
Ulcer	59	Self-reported physical activity, n(%)	Daily	11266 (29.1%)	12.1	9298 (29.5%)	
Ulcer	59	Sex, n(%)	Males	27197 (61.9%)	0.1	19619 (62.2%)	n=43992 vs n=31538
Ulcer	59	Sex, n(%)	Females	16738 (38.1%)	0.1	11919 (37.8%)	
Ulcer	59	Smoking status, n(%)	No	32252 (78.9%)	7.1	25152 (79.8%)	

Ulcer	59	Smoking status, n(%)	Yes	8633 (21.1%)	7.1	6386 (20.2%)	
Ulcer	59	Stroke, n(%)	No	38384 (95.8%)	9	30284 (96%)	
Ulcer	59	Stroke, n(%)	Yes	1663 (4.2%)	9	1254 (4%)	
Ulcer	59	Systolic blood pressure, mean(SD)		133.9 (15.1)	3	133.6 (14.9)	
Ulcer	59	Time since last healthcare visit, mean(SD)		0.7 (1.1)	0	0.6 (0.9)	
Ulcer	59	Total cholesterol, mean(SD)		4.9 (1.2)	9.3	4.8 (1.2)	
Ulcer	60	Age at diagnosis of type 2 diabetes, mean(SD)		55.9 (2.9)	0	55.4 (2.9)	
Ulcer	60	BMI, mean(SD)		31 (5.4)	8	31 (5.4)	
Ulcer	60	HbA1c, mean(SD)		51 (13)	2.2	51.1 (12.5)	
Ulcer	60	HDL-cholesterol, mean(SD)		1.2 (0.4)	13.3	1.2 (0.4)	
Ulcer	60	Ischemic heart disease, n(%)	No	37207 (87.4%)	8.7	29231 (87.8%)	
Ulcer	60	Ischemic heart disease, n(%)	Yes	5354 (12.6%)	8.7	4046 (12.2%)	
Ulcer	60	Neuropathy, n(%)	No	40131 (86.1%)	0	27792 (83.5%)	
Ulcer	60	Neuropathy, n(%)	Yes	6475 (13.9%)	0	5485 (16.5%)	
Ulcer	60	Self-reported physical activity, n(%)	Never	5345 (13.1%)	12.4	4303 (12.9%)	
Ulcer	60	Self-reported physical activity, n(%)	<1 times/week	5138 (12.6%)	12.4	4094 (12.3%)	
Ulcer	60	Self-reported physical activity, n(%)	regularly - 1-2 times/week	8515 (20.9%)	12.4	6941 (20.9%)	
Ulcer	60	Self-reported physical activity, n(%)	regularly - 3-5 times/week	9980 (24.4%)	12.4	8138 (24.5%)	
Ulcer	60	Self-reported physical activity, n(%)	Daily	11844 (29%)	12.4	9801 (29.5%)	
Ulcer	60	Sex, n(%)	Males	28651 (61.6%)	0.1	20613 (61.9%)	n=46606 vs n=33277
Ulcer	60	Sex, n(%)	Females	17889 (38.4%)	0.1	12664 (38.1%)	
Ulcer	60	Smoking status, n(%)	No	34331 (79.3%)	7.1	26664 (80.1%)	
Ulcer	60	Smoking status, n(%)	Yes	8971 (20.7%)	7.1	6613 (19.9%)	

Ulcer	60	Stroke, n(%)	No	40482 (95.6%)	9.1	31875 (95.8%)	
Ulcer	60	Stroke, n(%)	Yes	1885 (4.4%)	9.1	1402 (4.2%)	
Ulcer	60	Systolic blood pressure, mean(SD)		134.3 (15)	3	133.9 (14.7)	
Ulcer	60	Time since last healthcare visit, mean(SD)		0.7 (1.1)	0	0.6 (0.9)	
Ulcer	60	Total cholesterol, mean(SD)		4.8 (1.2)	9.3	4.8 (1.1)	
Ulcer	61	Age at diagnosis of type 2 diabetes, mean(SD)		57 (2.9)	0	56.4 (2.9)	
Ulcer	61	BMI, mean(SD)		30.9 (5.4)	8	30.8 (5.3)	
Ulcer	61	HbA1c, mean(SD)		50.8 (12.6)	2.2	51 (12.3)	
Ulcer	61	HDL-cholesterol, mean(SD)		1.2 (0.4)	13.1	1.2 (0.4)	
Ulcer	61	Ischemic heart disease, n(%)	No	39487 (86.8%)	8.7	31161 (87.3%)	
Ulcer	61	Ischemic heart disease, n(%)	Yes	6013 (13.2%)	8.7	4531 (12.7%)	
Ulcer	61	Neuropathy, n(%)	No	42811 (85.9%)	0	29702 (83.2%)	
Ulcer	61	Neuropathy, n(%)	Yes	7034 (14.1%)	0	5990 (16.8%)	
Ulcer	61	Self-reported physical activity, n(%)	Never	5650 (12.9%)	12.2	4498 (12.6%)	
Ulcer	61	Self-reported physical activity, n(%)	<1 times/week	5528 (12.6%)	12.2	4375 (12.3%)	
Ulcer	61	Self-reported physical activity, n(%)	regularly - 1-2 times/week	9125 (20.9%)	12.2	7476 (20.9%)	
Ulcer	61	Self-reported physical activity, n(%)	regularly - 3-5 times/week	10734 (24.5%)	12.2	8767 (24.6%)	
Ulcer	61	Self-reported physical activity, n(%)	Daily	12718 (29.1%)	12.2	10576 (29.6%)	
Ulcer	61	Sex, n(%)	Males	30546 (61.4%)	0.1	22071 (61.8%)	n=49845 vs n=35692
Ulcer	61	Sex, n(%)	Females	19235 (38.6%)	0.1	13621 (38.2%)	
Ulcer	61	Smoking status, n(%)	No	36981 (79.9%)	7.1	28777 (80.6%)	
Ulcer	61	Smoking status, n(%)	Yes	9329 (20.1%)	7.1	6915 (19.4%)	
Ulcer	61	Stroke, n(%)	No	43208 (95.4%)	9.1	34154 (95.7%)	

Ulcer	61	Stroke, n(%)	Yes	2094 (4.6%)	9.1	1538 (4.3%)	
Ulcer	61	Systolic blood pressure, mean(SD)		134.4 (15)	3	134 (14.8)	
Ulcer	61	Time since last healthcare visit, mean(SD)		0.7 (1.1)	0	0.6 (0.9)	
Ulcer	61	Total cholesterol, mean(SD)		4.8 (1.2)	9.2	4.8 (1.1)	
Ulcer	62	Age at diagnosis of type 2 diabetes, mean(SD)		57.9 (2.9)	0	57.4 (2.9)	
Ulcer	62	BMI, mean(SD)		30.7 (5.3)	8.1	30.7 (5.3)	
Ulcer	62	HbA1c, mean(SD)		50.5 (12.4)	2.1	50.7 (12)	
Ulcer	62	HDL-cholesterol, mean(SD)		1.2 (0.4)	13.1	1.2 (0.4)	
Ulcer	62	Ischemic heart disease, n(%)	No	41003 (86%)	8.8	32431 (86.5%)	
Ulcer	62	Ischemic heart disease, n(%)	Yes	6687 (14%)	8.8	5075 (13.5%)	
Ulcer	62	Neuropathy, n(%)	No	44545 (85.2%)	0	30892 (82.4%)	
Ulcer	62	Neuropathy, n(%)	Yes	7723 (14.8%)	0	6614 (17.6%)	
Ulcer	62	Self-reported physical activity, n(%)	Never	5947 (12.9%)	12.1	4795 (12.8%)	
Ulcer	62	Self-reported physical activity, n(%)	<1 times/week	5902 (12.8%)	12.1	4679 (12.5%)	
Ulcer	62	Self-reported physical activity, n(%)	regularly - 1-2 times/week	9326 (20.3%)	12.1	7596 (20.3%)	
Ulcer	62	Self-reported physical activity, n(%)	regularly - 3-5 times/week	11256 (24.5%)	12.1	9251 (24.7%)	
Ulcer	62	Self-reported physical activity, n(%)	Daily	13507 (29.4%)	12.1	11185 (29.8%)	
Ulcer	62	Sex, n(%)	Males	31859 (61%)	0.1	23073 (61.5%)	n=52268 vs n=37506
Ulcer	62	Sex, n(%)	Females	20336 (39%)	0.1	14433 (38.5%)	
Ulcer	62	Smoking status, n(%)	No	38928 (80.2%)	7.1	30427 (81.1%)	
Ulcer	62	Smoking status, n(%)	Yes	9617 (19.8%)	7.1	7079 (18.9%)	
Ulcer	62	Stroke, n(%)	No	45156 (95.1%)	9.2	35780 (95.4%)	
Ulcer	62	Stroke, n(%)	Yes	2317 (4.9%)	9.2	1726 (4.6%)	

Ulcer	62	Systolic blood pressure, mean(SD)		134.8 (15.1)	2.9	134.4 (14.9)	
Ulcer	62	Time since last healthcare visit, mean(SD)		0.7 (1.1)	0	0.6 (0.8)	
Ulcer	62	Total cholesterol, mean(SD)		4.8 (1.2)	9.1	4.8 (1.1)	
Ulcer	63	Age at diagnosis of type 2 diabetes, mean(SD)		58.9 (2.9)	0	58.3 (2.9)	
Ulcer	63	BMI, mean(SD)		30.6 (5.3)	8.2	30.6 (5.2)	
Ulcer	63	HbA1c, mean(SD)		50.3 (12.2)	2.1	50.5 (11.8)	
Ulcer	63	HDL-cholesterol, mean(SD)		1.2 (0.4)	13.3	1.2 (0.4)	
Ulcer	63	Ischemic heart disease, n(%)	No	42506 (85.2%)	8.9	33518 (85.6%)	
Ulcer	63	Ischemic heart disease, n(%)	Yes	7367 (14.8%)	8.9	5622 (14.4%)	
Ulcer	63	Neuropathy, n(%)	No	46338 (84.7%)	0	31990 (81.7%)	
Ulcer	63	Neuropathy, n(%)	Yes	8381 (15.3%)	0	7150 (18.3%)	
Ulcer	63	Self-reported physical activity, n(%)	Never	6159 (12.8%)	12.2	4942 (12.6%)	
Ulcer	63	Self-reported physical activity, n(%)	<1 times/week	6039 (12.6%)	12.2	4807 (12.3%)	
Ulcer	63	Self-reported physical activity, n(%)	regularly - 1-2 times/week	9842 (20.5%)	12.2	7955 (20.3%)	
Ulcer	63	Self-reported physical activity, n(%)	regularly - 3-5 times/week	11678 (24.3%)	12.2	9557 (24.4%)	
Ulcer	63	Self-reported physical activity, n(%)	Daily	14349 (29.9%)	12.2	11879 (30.4%)	
Ulcer	63	Sex, n(%)	Males	33220 (60.8%)	0.1	24000 (61.3%)	n=54719 vs n=39140
Ulcer	63	Sex, n(%)	Females	21423 (39.2%)	0.1	15140 (38.7%)	
Ulcer	63	Smoking status, n(%)	No	41100 (80.8%)	7.1	31974 (81.7%)	
Ulcer	63	Smoking status, n(%)	Yes	9746 (19.2%)	7.1	7166 (18.3%)	
Ulcer	63	Stroke, n(%)	No	47056 (94.8%)	9.3	37224 (95.1%)	
Ulcer	63	Stroke, n(%)	Yes	2574 (5.2%)	9.3	1916 (4.9%)	
Ulcer	63	Systolic blood pressure, mean(SD)		135.1 (15.2)	3	134.7 (14.9)	

Ulcer	63	Time since last healthcare visit, mean(SD)		0.7 (1.1)	0	0.6 (0.9)	
Ulcer	63	Total cholesterol, mean(SD)		4.8 (1.1)	9.1	4.8 (1.1)	
Ulcer	64	Age at diagnosis of type 2 diabetes, mean(SD)		59.8 (2.9)	0	59.3 (2.9)	
Ulcer	64	BMI, mean(SD)		30.5 (5.3)	8.2	30.5 (5.2)	
Ulcer	64	HbA1c, mean(SD)		50.1 (11.9)	2.2	50.3 (11.6)	
Ulcer	64	HDL-cholesterol, mean(SD)		1.3 (0.4)	13.2	1.3 (0.4)	
Ulcer	64	Ischemic heart disease, n(%)	No	43923 (84.5%)	8.7	34706 (84.9%)	
Ulcer	64	Ischemic heart disease, n(%)	Yes	8076 (15.5%)	8.7	6168 (15.1%)	
Ulcer	64	Neuropathy, n(%)	No	48008 (84.3%)	0	33172 (81.2%)	
Ulcer	64	Neuropathy, n(%)	Yes	8957 (15.7%)	0	7702 (18.8%)	
Ulcer	64	Self-reported physical activity, n(%)	Never	6190 (12.4%)	12.2	4977 (12.2%)	
Ulcer	64	Self-reported physical activity, n(%)	<1 times/week	6024 (12%)	12.2	4784 (11.7%)	
Ulcer	64	Self-reported physical activity, n(%)	regularly - 1-2 times/week	10077 (20.2%)	12.2	8208 (20.1%)	
Ulcer	64	Self-reported physical activity, n(%)	regularly - 3-5 times/week	12284 (24.6%)	12.2	10126 (24.8%)	
Ulcer	64	Self-reported physical activity, n(%)	Daily	15430 (30.9%)	12.2	12779 (31.3%)	
Ulcer	64	Sex, n(%)	Males	34530 (60.7%)	0.1	24930 (61%)	n=56965 vs n=40874
Ulcer	64	Sex, n(%)	Females	22355 (39.3%)	0.1	15944 (39%)	
Ulcer	64	Smoking status, n(%)	No	43054 (81.4%)	7.2	33625 (82.3%)	
Ulcer	64	Smoking status, n(%)	Yes	9836 (18.6%)	7.2	7249 (17.7%)	
Ulcer	64	Stroke, n(%)	No	48864 (94.4%)	9.2	38708 (94.7%)	
Ulcer	64	Stroke, n(%)	Yes	2883 (5.6%)	9.2	2166 (5.3%)	
Ulcer	64	Systolic blood pressure, mean(SD)		135.2 (15.2)	2.9	134.8 (14.9)	
Ulcer	64	Time since last healthcare visit, mean(SD)		0.7 (1.1)	0	0.6 (0.8)	

Ulcer	64	Total cholesterol, mean(SD)		4.8 (1.1)	9.1	4.8 (1.1)	
Ulcer	65	Age at diagnosis of type 2 diabetes, mean(SD)		60.8 (2.9)	0	60.2 (2.9)	
Ulcer	65	BMI, mean(SD)		30.4 (5.3)	8.2	30.4 (5.3)	
Ulcer	65	HbA1c, mean(SD)		49.7 (11.7)	2.1	49.9 (11.3)	
Ulcer	65	HDL-cholesterol, mean(SD)		1.3 (0.4)	13	1.3 (0.4)	
Ulcer	65	Ischemic heart disease, n(%)	No	45444 (83.7%)	8.7	35970 (84.2%)	
Ulcer	65	Ischemic heart disease, n(%)	Yes	8821 (16.3%)	8.7	6744 (15.8%)	
Ulcer	65	Neuropathy, n(%)	No	49605 (83.5%)	0	34251 (80.2%)	
Ulcer	65	Neuropathy, n(%)	Yes	9822 (16.5%)	0	8463 (19.8%)	
Ulcer	65	Self-reported physical activity, n(%)	Never	6393 (12.2%)	12	5151 (12.1%)	
Ulcer	65	Self-reported physical activity, n(%)	<1 times/week	6113 (11.7%)	12	4864 (11.4%)	
Ulcer	65	Self-reported physical activity, n(%)	regularly - 1-2 times/week	10452 (20%)	12	8484 (19.9%)	
Ulcer	65	Self-reported physical activity, n(%)	regularly - 3-5 times/week	12624 (24.2%)	12	10421 (24.4%)	
Ulcer	65	Self-reported physical activity, n(%)	Daily	16691 (31.9%)	12	13794 (32.3%)	
Ulcer	65	Sex, n(%)	Males	35865 (60.4%)	0.1	25923 (60.7%)	n=59427 vs n=42714
Ulcer	65	Sex, n(%)	Females	23491 (39.6%)	0.1	16791 (39.3%)	
Ulcer	65	Smoking status, n(%)	No	45332 (82%)	7	35322 (82.7%)	
Ulcer	65	Smoking status, n(%)	Yes	9923 (18%)	7	7392 (17.3%)	
Ulcer	65	Stroke, n(%)	No	50786 (94.1%)	9.2	40316 (94.4%)	
Ulcer	65	Stroke, n(%)	Yes	3187 (5.9%)	9.2	2398 (5.6%)	
Ulcer	65	Systolic blood pressure, mean(SD)		135.3 (15.1)	2.8	134.9 (14.8)	
Ulcer	65	Time since last healthcare visit, mean(SD)		0.7 (1.1)	0	0.6 (0.9)	
Ulcer	65	Total cholesterol, mean(SD)		4.8 (1.1)	8.8	4.7 (1.1)	

Ulcer	66	Age at diagnosis of type 2 diabetes, mean(SD)		61.8 (2.9)	0	61.2 (2.9)	
Ulcer	66	BMI, mean(SD)		30.3 (5.3)	8.2	30.3 (5.2)	
Ulcer	66	HbA1c, mean(SD)		49.6 (11.7)	2	49.7 (11.3)	
Ulcer	66	HDL-cholesterol, mean(SD)		1.3 (0.4)	12.8	1.3 (0.4)	
Ulcer	66	Ischemic heart disease, n(%)	No	47125 (83.1%)	8.6	37379 (83.5%)	
Ulcer	66	Ischemic heart disease, n(%)	Yes	9569 (16.9%)	8.6	7370 (16.5%)	
Ulcer	66	Neuropathy, n(%)	No	51166 (82.5%)	0	35403 (79.1%)	
Ulcer	66	Neuropathy, n(%)	Yes	10835 (17.5%)	0	9346 (20.9%)	
Ulcer	66	Self-reported physical activity, n(%)	Never	6469 (11.8%)	11.8	5175 (11.6%)	
Ulcer	66	Self-reported physical activity, n(%)	<1 times/week	6137 (11.2%)	11.8	4890 (10.9%)	
Ulcer	66	Self-reported physical activity, n(%)	regularly - 1-2 times/week	10531 (19.3%)	11.8	8559 (19.1%)	
Ulcer	66	Self-reported physical activity, n(%)	regularly - 3-5 times/week	13238 (24.2%)	11.8	10964 (24.5%)	
Ulcer	66	Self-reported physical activity, n(%)	Daily	18300 (33.5%)	11.8	15161 (33.9%)	
Ulcer	66	Sex, n(%)	Males	37217 (60.1%)	0.1	27049 (60.4%)	n=62001 vs n=44749
Ulcer	66	Sex, n(%)	Females	24712 (39.9%)	0.1	17700 (39.6%)	
Ulcer	66	Smoking status, n(%)	No	47784 (82.8%)	6.9	37295 (83.3%)	
Ulcer	66	Smoking status, n(%)	Yes	9949 (17.2%)	6.9	7454 (16.7%)	
Ulcer	66	Stroke, n(%)	No	52918 (93.8%)	9	42119 (94.1%)	
Ulcer	66	Stroke, n(%)	Yes	3493 (6.2%)	9	2630 (5.9%)	
Ulcer	66	Systolic blood pressure, mean(SD)		135.6 (15.3)	2.7	135.2 (15)	
Ulcer	66	Time since last healthcare visit, mean(SD)		0.7 (1.1)	0	0.6 (0.9)	
Ulcer	66	Total cholesterol, mean(SD)		4.8 (1.1)	8.7	4.7 (1.1)	
Ulcer	67	Age at diagnosis of type 2 diabetes, mean(SD)		62.7 (2.9)	0	62.1 (2.9)	

Ulcer	67	BMI, mean(SD)		30.2 (5.2)	8.2	30.2 (5.2)	
Ulcer	67	HbA1c, mean(SD)		49.3 (11.3)	2	49.4 (10.9)	
Ulcer	67	HDL-cholesterol, mean(SD)		1.3 (0.4)	12.6	1.3 (0.4)	
Ulcer	67	Ischemic heart disease, n(%)	No	48225 (82.3%)	8.4	38355 (82.9%)	
Ulcer	67	Ischemic heart disease, n(%)	Yes	10340 (17.7%)	8.4	7914 (17.1%)	
Ulcer	67	Neuropathy, n(%)	No	52202 (81.6%)	0	36158 (78.1%)	
Ulcer	67	Neuropathy, n(%)	Yes	11763 (18.4%)	0	10111 (21.9%)	
Ulcer	67	Self-reported physical activity, n(%)	Never	6652 (11.8%)	11.7	5301 (11.5%)	
Ulcer	67	Self-reported physical activity, n(%)	<1 times/week	6197 (11%)	11.7	4913 (10.6%)	
Ulcer	67	Self-reported physical activity, n(%)	regularly - 1-2 times/week	10486 (18.6%)	11.7	8492 (18.4%)	
Ulcer	67	Self-reported physical activity, n(%)	regularly - 3-5 times/week	13700 (24.3%)	11.7	11387 (24.6%)	
Ulcer	67	Self-reported physical activity, n(%)	Daily	19438 (34.4%)	11.7	16176 (35%)	
Ulcer	67	Sex, n(%)	Males	38094 (59.6%)	0.1	27800 (60.1%)	n=63965 vs n=46269
Ulcer	67	Sex, n(%)	Females	25801 (40.4%)	0.1	18469 (39.9%)	
Ulcer	67	Smoking status, n(%)	No	50006 (83.8%)	6.7	39065 (84.4%)	
Ulcer	67	Smoking status, n(%)	Yes	9669 (16.2%)	6.7	7204 (15.6%)	
Ulcer	67	Stroke, n(%)	No	54429 (93.4%)	8.9	43398 (93.8%)	
Ulcer	67	Stroke, n(%)	Yes	3827 (6.6%)	8.9	2871 (6.2%)	
Ulcer	67	Systolic blood pressure, mean(SD)		135.7 (15.2)	2.7	135.4 (14.9)	
Ulcer	67	Time since last healthcare visit, mean(SD)		0.7 (1.1)	0	0.6 (0.9)	
Ulcer	67	Total cholesterol, mean(SD)		4.8 (1.1)	8.5	4.7 (1.1)	
Ulcer	68	Age at diagnosis of type 2 diabetes, mean(SD)		63.6 (2.9)	0	63.1 (2.9)	
Ulcer	68	BMI, mean(SD)		30.1 (5.3)	8	30.1 (5.2)	

Ulcer	68	HbA1c, mean(SD)		49.1 (11)	1.9	49.2 (10.7)	
Ulcer	68	HDL-cholesterol, mean(SD)		1.3 (0.4)	12.4	1.3 (0.4)	
Ulcer	68	Ischemic heart disease, n(%)	No	48862 (81.6%)	8.3	39090 (82.1%)	
Ulcer	68	Ischemic heart disease, n(%)	Yes	11021 (18.4%)	8.3	8502 (17.9%)	
Ulcer	68	Neuropathy, n(%)	No	52706 (80.7%)	0	36723 (77.2%)	
Ulcer	68	Neuropathy, n(%)	Yes	12607 (19.3%)	0	10869 (22.8%)	
Ulcer	68	Self-reported physical activity, n(%)	Never	6864 (11.9%)	11.4	5509 (11.6%)	
Ulcer	68	Self-reported physical activity, n(%)	<1 times/week	6359 (11%)	11.4	5092 (10.7%)	
Ulcer	68	Self-reported physical activity, n(%)	regularly - 1-2 times/week	10439 (18%)	11.4	8436 (17.7%)	
Ulcer	68	Self-reported physical activity, n(%)	regularly - 3-5 times/week	13962 (24.1%)	11.4	11553 (24.3%)	
Ulcer	68	Self-reported physical activity, n(%)	Daily	20223 (35%)	11.4	17002 (35.7%)	
Ulcer	68	Sex, n(%)	Males	38638 (59.2%)	0.1	28383 (59.6%)	n=65313 vs n=47592
Ulcer	68	Sex, n(%)	Females	26605 (40.8%)	0.1	19209 (40.4%)	
Ulcer	68	Smoking status, n(%)	No	51511 (84.5%)	6.7	40473 (85%)	
Ulcer	68	Smoking status, n(%)	Yes	9453 (15.5%)	6.7	7119 (15%)	
Ulcer	68	Stroke, n(%)	No	55433 (93.1%)	8.8	44449 (93.4%)	
Ulcer	68	Stroke, n(%)	Yes	4118 (6.9%)	8.8	3143 (6.6%)	
Ulcer	68	Systolic blood pressure, mean(SD)		135.7 (15.2)	2.6	135.3 (14.9)	
Ulcer	68	Time since last healthcare visit, mean(SD)		0.7 (1.1)	0	0.6 (0.9)	
Ulcer	68	Total cholesterol, mean(SD)		4.7 (1.1)	8.4	4.7 (1.1)	
Ulcer	69	Age at diagnosis of type 2 diabetes, mean(SD)		64.5 (2.9)	0	64 (2.9)	
Ulcer	69	BMI, mean(SD)		30 (5.2)	7.9	30 (5.2)	
Ulcer	69	HbA1c, mean(SD)		49 (10.9)	2	49.1 (10.5)	

Ulcer	69	HDL-cholesterol, mean(SD)		1.3 (0.4)	12.3	1.3 (0.4)	
Ulcer	69	Ischemic heart disease, n(%)	No	49277 (80.8%)	7.9	39565 (81.3%)	
Ulcer	69	Ischemic heart disease, n(%)	Yes	11713 (19.2%)	7.9	9077 (18.7%)	
Ulcer	69	Neuropathy, n(%)	No	52742 (79.7%)	0	37022 (76.1%)	
Ulcer	69	Neuropathy, n(%)	Yes	13474 (20.3%)	0	11620 (23.9%)	
Ulcer	69	Self-reported physical activity, n(%)	Never	6962 (11.8%)	11.3	5627 (11.6%)	
Ulcer	69	Self-reported physical activity, n(%)	<1 times/week	6467 (11%)	11.3	5165 (10.6%)	
Ulcer	69	Self-reported physical activity, n(%)	regularly - 1-2 times/week	10701 (18.2%)	11.3	8787 (18.1%)	
Ulcer	69	Self-reported physical activity, n(%)	regularly - 3-5 times/week	14013 (23.8%)	11.3	11690 (24%)	
Ulcer	69	Self-reported physical activity, n(%)	Daily	20620 (35.1%)	11.3	17373 (35.7%)	
Ulcer	69	Sex, n(%)	Males	38871 (58.8%)	0.1	28772 (59.2%)	n=66216 vs n=48642
Ulcer	69	Sex, n(%)	Females	27275 (41.2%)	0.1	19870 (40.8%)	
Ulcer	69	Smoking status, n(%)	No	52804 (85.3%)	6.5	41789 (85.9%)	
Ulcer	69	Smoking status, n(%)	Yes	9075 (14.7%)	6.5	6853 (14.1%)	
Ulcer	69	Stroke, n(%)	No	56331 (92.9%)	8.4	45330 (93.2%)	
Ulcer	69	Stroke, n(%)	Yes	4336 (7.1%)	8.4	3312 (6.8%)	
Ulcer	69	Systolic blood pressure, mean(SD)		135.8 (15.2)	2.5	135.5 (14.9)	
Ulcer	69	Time since last healthcare visit, mean(SD)		0.7 (1.1)	0	0.6 (0.9)	
Ulcer	69	Total cholesterol, mean(SD)		4.7 (1.1)	8.3	4.7 (1.1)	
Ulcer	70	Age at diagnosis of type 2 diabetes, mean(SD)		65.5 (3)	0	64.9 (2.9)	
Ulcer	70	BMI, mean(SD)		29.9 (5.2)	7.8	29.9 (5.1)	
Ulcer	70	HbA1c, mean(SD)		48.8 (10.9)	2	48.9 (10.5)	
Ulcer	70	HDL-cholesterol, mean(SD)		1.3 (0.4)	12.4	1.3 (0.4)	

Ulcer	70	Ischemic heart disease, n(%)	No	49010 (80%)	7.8	39308 (80.6%)	
Ulcer	70	Ischemic heart disease, n(%)	Yes	12237 (20%)	7.8	9481 (19.4%)	
Ulcer	70	Neuropathy, n(%)	No	52150 (78.5%)	0	36472 (74.8%)	
Ulcer	70	Neuropathy, n(%)	Yes	14248 (21.5%)	0	12317 (25.2%)	
Ulcer	70	Self-reported physical activity, n(%)	Never	7051 (12%)	11.2	5772 (11.8%)	
Ulcer	70	Self-reported physical activity, n(%)	<1 times/week	6338 (10.7%)	11.2	5063 (10.4%)	
Ulcer	70	Self-reported physical activity, n(%)	regularly - 1-2 times/week	10693 (18.1%)	11.2	8754 (17.9%)	
Ulcer	70	Self-reported physical activity, n(%)	regularly - 3-5 times/week	14144 (24%)	11.2	11807 (24.2%)	
Ulcer	70	Self-reported physical activity, n(%)	Daily	20753 (35.2%)	11.2	17393 (35.6%)	
Ulcer	70	Sex, n(%)	Males	38754 (58.4%)	0.1	28717 (58.9%)	n=66398 vs n=48789
Ulcer	70	Sex, n(%)	Females	27589 (41.6%)	0.1	20072 (41.1%)	
Ulcer	70	Smoking status, n(%)	No	53400 (86.1%)	6.6	42273 (86.6%)	
Ulcer	70	Smoking status, n(%)	Yes	8620 (13.9%)	6.6	6516 (13.4%)	
Ulcer	70	Stroke, n(%)	No	56380 (92.6%)	8.3	45296 (92.8%)	
Ulcer	70	Stroke, n(%)	Yes	4525 (7.4%)	8.3	3493 (7.2%)	
Ulcer	70	Systolic blood pressure, mean(SD)		135.9 (15.3)	2.5	135.5 (15)	
Ulcer	70	Time since last healthcare visit, mean(SD)		0.7 (1.2)	0	0.6 (0.9)	
Ulcer	70	Total cholesterol, mean(SD)		4.7 (1.1)	8.3	4.7 (1.1)	
Ulcer	71	Age at diagnosis of type 2 diabetes, mean(SD)		66.4 (3)	0	65.8 (3)	
Ulcer	71	BMI, mean(SD)		29.8 (5.1)	7.7	29.8 (5.1)	
Ulcer	71	HbA1c, mean(SD)		48.8 (10.7)	1.8	48.8 (10.2)	
Ulcer	71	HDL-cholesterol, mean(SD)		1.3 (0.4)	12.3	1.3 (0.4)	
Ulcer	71	Ischemic heart disease, n(%)	No	48104 (79.2%)	7.5	38621 (79.6%)	

Ulcer	71	Ischemic heart disease, n(%)	Yes	12662 (20.8%)	7.5	9905 (20.4%)	
Ulcer	71	Neuropathy, n(%)	No	50998 (77.6%)	0	35748 (73.7%)	
Ulcer	71	Neuropathy, n(%)	Yes	14711 (22.4%)	0	12778 (26.3%)	
Ulcer	71	Self-reported physical activity, n(%)	Never	7072 (12.1%)	10.9	5793 (11.9%)	
Ulcer	71	Self-reported physical activity, n(%)	<1 times/week	6547 (11.2%)	10.9	5240 (10.8%)	
Ulcer	71	Self-reported physical activity, n(%)	regularly - 1-2 times/week	10649 (18.2%)	10.9	8748 (18%)	
Ulcer	71	Self-reported physical activity, n(%)	regularly - 3-5 times/week	13743 (23.5%)	10.9	11499 (23.7%)	
Ulcer	71	Self-reported physical activity, n(%)	Daily	20508 (35%)	10.9	17246 (35.5%)	
Ulcer	71	Sex, n(%)	Males	38054 (58%)	0.1	28251 (58.2%)	n=65709 vs n=48526
Ulcer	71	Sex, n(%)	Females	27601 (42%)	0.1	20275 (41.8%)	
Ulcer	71	Smoking status, n(%)	No	53322 (86.9%)	6.6	42408 (87.4%)	
Ulcer	71	Smoking status, n(%)	Yes	8064 (13.1%)	6.6	6118 (12.6%)	
Ulcer	71	Stroke, n(%)	No	55700 (92.2%)	8.1	44912 (92.6%)	
Ulcer	71	Stroke, n(%)	Yes	4719 (7.8%)	8.1	3614 (7.4%)	
Ulcer	71	Systolic blood pressure, mean(SD)		136 (15.3)	2.4	135.7 (15)	
Ulcer	71	Time since last healthcare visit, mean(SD)		0.7 (1.2)	0	0.6 (0.9)	
Ulcer	71	Total cholesterol, mean(SD)		4.7 (1.1)	8.3	4.6 (1.1)	
Ulcer	72	Age at diagnosis of type 2 diabetes, mean(SD)		67.3 (3)	0	66.8 (3)	
Ulcer	72	BMI, mean(SD)		29.7 (5.1)	7.7	29.6 (5.1)	
Ulcer	72	HbA1c, mean(SD)		48.6 (10.4)	1.8	48.7 (10)	
Ulcer	72	HDL-cholesterol, mean(SD)		1.3 (0.4)	12.3	1.3 (0.4)	
Ulcer	72	Ischemic heart disease, n(%)	No	46297 (78.4%)	7.4	37282 (78.9%)	
Ulcer	72	Ischemic heart disease, n(%)	Yes	12782 (21.6%)	7.4	9963 (21.1%)	

Ulcer	72	Neuropathy, n(%)	No	48728 (76.4%)	0	34132 (72.2%)	
Ulcer	72	Neuropathy, n(%)	Yes	15065 (23.6%)	0	13113 (27.8%)	
Ulcer	72	Self-reported physical activity, n(%)	Never	7033 (12.3%)	10.7	5756 (12.2%)	
Ulcer	72	Self-reported physical activity, n(%)	<1 times/week	6247 (11%)	10.7	5014 (10.6%)	
Ulcer	72	Self-reported physical activity, n(%)	regularly - 1-2 times/week	10465 (18.4%)	10.7	8598 (18.2%)	
Ulcer	72	Self-reported physical activity, n(%)	regularly - 3-5 times/week	13308 (23.4%)	10.7	11111 (23.5%)	
Ulcer	72	Self-reported physical activity, n(%)	Daily	19905 (34.9%)	10.7	16766 (35.5%)	
Ulcer	72	Sex, n(%)	Males	36585 (57.4%)	0.1	27345 (57.9%)	n=63793 vs n=47245
Ulcer	72	Sex, n(%)	Females	27156 (42.6%)	0.1	19900 (42.1%)	
Ulcer	72	Smoking status, n(%)	No	52275 (87.5%)	6.3	41510 (87.9%)	
Ulcer	72	Smoking status, n(%)	Yes	7474 (12.5%)	6.3	5735 (12.1%)	
Ulcer	72	Stroke, n(%)	No	53791 (91.6%)	7.9	43447 (92%)	
Ulcer	72	Stroke, n(%)	Yes	4959 (8.4%)	7.9	3798 (8%)	
Ulcer	72	Systolic blood pressure, mean(SD)		136 (15.5)	2.4	135.7 (15.2)	
Ulcer	72	Time since last healthcare visit, mean(SD)		0.7 (1.2)	0	0.6 (0.9)	
Ulcer	72	Total cholesterol, mean(SD)		4.7 (1.1)	8.3	4.6 (1.1)	
Ulcer	73	Age at diagnosis of type 2 diabetes, mean(SD)		68.3 (3.1)	0	67.7 (3)	
Ulcer	73	BMI, mean(SD)		29.5 (5.1)	7.9	29.5 (5.1)	
Ulcer	73	HbA1c, mean(SD)		48.6 (10.4)	1.9	48.7 (10)	
Ulcer	73	HDL-cholesterol, mean(SD)		1.3 (0.4)	12.4	1.3 (0.4)	
Ulcer	73	Ischemic heart disease, n(%)	No	44146 (77.5%)	7.5	35451 (78%)	
Ulcer	73	Ischemic heart disease, n(%)	Yes	12822 (22.5%)	7.5	10002 (22%)	
Ulcer	73	Neuropathy, n(%)	No	46380 (75.3%)	0	32239 (70.9%)	

Ulcer	73	Neuropathy, n(%)	Yes	15219 (24.7%)	0	13214 (29.1%)	
Ulcer	73	Self-reported physical activity, n(%)	Never	7044 (12.8%)	10.9	5733 (12.6%)	
Ulcer	73	Self-reported physical activity, n(%)	<1 times/week	6146 (11.2%)	10.9	4912 (10.8%)	
Ulcer	73	Self-reported physical activity, n(%)	regularly - 1-2 times/week	10106 (18.4%)	10.9	8333 (18.3%)	
Ulcer	73	Self-reported physical activity, n(%)	regularly - 3-5 times/week	12741 (23.2%)	10.9	10650 (23.4%)	
Ulcer	73	Self-reported physical activity, n(%)	Daily	18872 (34.4%)	10.9	15825 (34.8%)	
Ulcer	73	Sex, n(%)	Males	34843 (56.6%)	0.1	25986 (57.2%)	n=61599 vs n=45453
Ulcer	73	Sex, n(%)	Females	26691 (43.4%)	0.1	19467 (42.8%)	
Ulcer	73	Smoking status, n(%)	No	50899 (88.5%)	6.6	40326 (88.7%)	
Ulcer	73	Smoking status, n(%)	Yes	6643 (11.5%)	6.6	5127 (11.3%)	
Ulcer	73	Stroke, n(%)	No	51478 (90.9%)	8.1	41433 (91.2%)	
Ulcer	73	Stroke, n(%)	Yes	5153 (9.1%)	8.1	4020 (8.8%)	
Ulcer	73	Systolic blood pressure, mean(SD)		136 (15.5)	2.4	135.7 (15.3)	
Ulcer	73	Time since last healthcare visit, mean(SD)		0.7 (1.2)	0	0.6 (0.9)	
Ulcer	73	Total cholesterol, mean(SD)		4.7 (1.1)	8.5	4.6 (1.1)	
Ulcer	74	Age at diagnosis of type 2 diabetes, mean(SD)		69.2 (3.1)	0	68.7 (3)	
Ulcer	74	BMI, mean(SD)		29.4 (5.1)	8	29.4 (5.1)	
Ulcer	74	HbA1c, mean(SD)		48.6 (10.2)	1.8	48.6 (9.8)	
Ulcer	74	HDL-cholesterol, mean(SD)		1.3 (0.4)	12.5	1.3 (0.4)	
Ulcer	74	Ischemic heart disease, n(%)	No	41354 (76.6%)	7.6	33230 (77.2%)	
Ulcer	74	Ischemic heart disease, n(%)	Yes	12630 (23.4%)	7.6	9796 (22.8%)	
Ulcer	74	Neuropathy, n(%)	No	43213 (74%)	0	29895 (69.5%)	
Ulcer	74	Neuropathy, n(%)	Yes	15220 (26%)	0	13131 (30.5%)	

Ulcer	74	Self-reported physical activity, n(%)	Never	6997 (13.4%)	10.9	5681 (13.2%)	
Ulcer	74	Self-reported physical activity, n(%)	<1 times/week	6019 (11.6%)	10.9	4787 (11.1%)	
Ulcer	74	Self-reported physical activity, n(%)	regularly - 1-2 times/week	9664 (18.6%)	10.9	7927 (18.4%)	
Ulcer	74	Self-reported physical activity, n(%)	regularly - 3-5 times/week	11700 (22.5%)	10.9	9793 (22.8%)	
Ulcer	74	Self-reported physical activity, n(%)	Daily	17689 (34%)	10.9	14838 (34.5%)	
Ulcer	74	Sex, n(%)	Males	32698 (56%)	0.1	24255 (56.4%)	n=58433 vs n=43026
Ulcer	74	Sex, n(%)	Females	25665 (44%)	0.1	18771 (43.6%)	
Ulcer	74	Smoking status, n(%)	No	48618 (89.2%)	6.7	38478 (89.4%)	
Ulcer	74	Smoking status, n(%)	Yes	5917 (10.8%)	6.7	4548 (10.6%)	
Ulcer	74	Stroke, n(%)	No	48520 (90.4%)	8.2	39031 (90.7%)	
Ulcer	74	Stroke, n(%)	Yes	5147 (9.6%)	8.2	3995 (9.3%)	
Ulcer	74	Systolic blood pressure, mean(SD)		136.1 (15.7)	2.4	135.8 (15.4)	
Ulcer	74	Time since last healthcare visit, mean(SD)		0.7 (1.2)	0	0.6 (0.9)	
Ulcer	74	Total cholesterol, mean(SD)		4.6 (1.1)	8.6	4.6 (1.1)	
Ulcer	75	Age at diagnosis of type 2 diabetes, mean(SD)		70.2 (3.1)	0	69.6 (3)	
Ulcer	75	BMI, mean(SD)		29.3 (5)	8.1	29.3 (5)	
Ulcer	75	HbA1c, mean(SD)		48.5 (10.1)	1.9	48.6 (9.8)	
Ulcer	75	HDL-cholesterol, mean(SD)		1.3 (0.4)	12.6	1.3 (0.4)	
Ulcer	75	Ischemic heart disease, n(%)	No	38222 (75.5%)	7.6	30570 (76.1%)	
Ulcer	75	Ischemic heart disease, n(%)	Yes	12371 (24.5%)	7.6	9615 (23.9%)	
Ulcer	75	Neuropathy, n(%)	No	40015 (73.1%)	0	27468 (68.4%)	
Ulcer	75	Neuropathy, n(%)	Yes	14729 (26.9%)	0	12717 (31.6%)	
Ulcer	75	Self-reported physical activity, n(%)	Never	6809 (14%)	11.1	5542 (13.8%)	

Ulcer	75	Self-reported physical activity, n(%)	<1 times/week	5617 (11.5%)	11.1	4459 (11.1%)	
Ulcer	75	Self-reported physical activity, n(%)	regularly - 1-2 times/week	9100 (18.7%)	11.1	7440 (18.5%)	
Ulcer	75	Self-reported physical activity, n(%)	regularly - 3-5 times/week	10857 (22.3%)	11.1	9118 (22.7%)	
Ulcer	75	Self-reported physical activity, n(%)	Daily	16309 (33.5%)	11.1	13626 (33.9%)	
Ulcer	75	Sex, n(%)	Males	30143 (55.1%)	0.1	22313 (55.5%)	n=54744 vs n=40185
Ulcer	75	Sex, n(%)	Females	24538 (44.9%)	0.1	17872 (44.5%)	
Ulcer	75	Smoking status, n(%)	No	45877 (90%)	6.9	36309 (90.4%)	
Ulcer	75	Smoking status, n(%)	Yes	5108 (10%)	6.9	3876 (9.6%)	
Ulcer	75	Stroke, n(%)	No	45185 (90%)	8.3	36256 (90.2%)	
Ulcer	75	Stroke, n(%)	Yes	5042 (10%)	8.3	3929 (9.8%)	
Ulcer	75	Systolic blood pressure, mean(SD)		136.3 (15.7)	2.4	135.9 (15.5)	
Ulcer	75	Time since last healthcare visit, mean(SD)		0.7 (1.2)	0	0.6 (0.9)	
Ulcer	75	Total cholesterol, mean(SD)		4.6 (1.1)	8.7	4.6 (1.1)	
Ulcer	76	Age at diagnosis of type 2 diabetes, mean(SD)		71.3 (3.1)	0	70.7 (3)	
Ulcer	76	BMI, mean(SD)		29.2 (5)	8.3	29.2 (5)	
Ulcer	76	HbA1c, mean(SD)		48.5 (10.1)	2	48.6 (9.8)	
Ulcer	76	HDL-cholesterol, mean(SD)		1.3 (0.4)	13.2	1.3 (0.4)	
Ulcer	76	Ischemic heart disease, n(%)	No	35011 (74.5%)	7.7	27829 (75%)	
Ulcer	76	Ischemic heart disease, n(%)	Yes	11967 (25.5%)	7.7	9254 (25%)	
Ulcer	76	Neuropathy, n(%)	No	36874 (72.4%)	0	24967 (67.3%)	
Ulcer	76	Neuropathy, n(%)	Yes	14039 (27.6%)	0	12116 (32.7%)	
Ulcer	76	Self-reported physical activity, n(%)	Never	6539 (14.5%)	11.4	5312 (14.3%)	
Ulcer	76	Self-reported physical activity, n(%)	<1 times/week	5252 (11.6%)	11.4	4158 (11.2%)	

Ulcer	76	Self-reported physical activity, n(%)	regularly - 1-2 times/week	8387 (18.6%)	11.4	6810 (18.4%)	
Ulcer	76	Self-reported physical activity, n(%)	regularly - 3-5 times/week	9930 (22%)	11.4	8264 (22.3%)	
Ulcer	76	Self-reported physical activity, n(%)	Daily	15012 (33.3%)	11.4	12539 (33.8%)	
Ulcer	76	Sex, n(%)	Males	27691 (54.5%)	0.1	20373 (54.9%)	n=50913 vs n=37083
Ulcer	76	Sex, n(%)	Females	23162 (45.5%)	0.1	16710 (45.1%)	
Ulcer	76	Smoking status, n(%)	No	42926 (90.7%)	7.1	33770 (91.1%)	
Ulcer	76	Smoking status, n(%)	Yes	4390 (9.3%)	7.1	3313 (8.9%)	
Ulcer	76	Stroke, n(%)	No	41656 (89.3%)	8.4	33248 (89.7%)	
Ulcer	76	Stroke, n(%)	Yes	4974 (10.7%)	8.4	3835 (10.3%)	
Ulcer	76	Systolic blood pressure, mean(SD)		136.1 (15.8)	2.5	135.8 (15.5)	
Ulcer	76	Time since last healthcare visit, mean(SD)		0.7 (1.2)	0	0.6 (0.9)	
Ulcer	76	Total cholesterol, mean(SD)		4.6 (1.1)	9.2	4.6 (1.1)	
Ulcer	77	Age at diagnosis of type 2 diabetes, mean(SD)		72.3 (3.1)	0	71.6 (3)	
Ulcer	77	BMI, mean(SD)		29 (4.9)	8.4	29 (4.9)	
Ulcer	77	HbA1c, mean(SD)		48.5 (10)	2	48.6 (9.6)	
Ulcer	77	HDL-cholesterol, mean(SD)		1.3 (0.4)	13.7	1.3 (0.4)	
Ulcer	77	Ischemic heart disease, n(%)	No	31970 (73.4%)	7.9	25265 (73.9%)	
Ulcer	77	Ischemic heart disease, n(%)	Yes	11572 (26.6%)	7.9	8928 (26.1%)	
Ulcer	77	Neuropathy, n(%)	No	33928 (71.8%)	0	22716 (66.4%)	
Ulcer	77	Neuropathy, n(%)	Yes	13327 (28.2%)	0	11477 (33.6%)	
Ulcer	77	Self-reported physical activity, n(%)	Never	6199 (14.9%)	11.8	4995 (14.6%)	
Ulcer	77	Self-reported physical activity, n(%)	<1 times/week	5011 (12%)	11.8	3957 (11.6%)	
Ulcer	77	Self-reported physical activity, n(%)	regularly - 1-2 times/week	7715 (18.5%)	11.8	6295 (18.4%)	

Ulcer	77	Self-reported physical activity, n(%)	regularly - 3-5 times/week	9082 (21.8%)	11.8	7546 (22.1%)	
Ulcer	77	Self-reported physical activity, n(%)	Daily	13693 (32.8%)	11.8	11400 (33.3%)	
Ulcer	77	Sex, n(%)	Males	25318 (53.6%)	0.1	18627 (54.5%)	n=47255 vs n=34193
Ulcer	77	Sex, n(%)	Females	21880 (46.4%)	0.1	15566 (45.5%)	
Ulcer	77	Smoking status, n(%)	No	40127 (91.5%)	7.2	31407 (91.9%)	
Ulcer	77	Smoking status, n(%)	Yes	3736 (8.5%)	7.2	2786 (8.1%)	
Ulcer	77	Stroke, n(%)	No	38446 (88.9%)	8.5	30522 (89.3%)	
Ulcer	77	Stroke, n(%)	Yes	4815 (11.1%)	8.5	3671 (10.7%)	
Ulcer	77	Systolic blood pressure, mean(SD)		136.2 (15.9)	2.5	135.9 (15.6)	
Ulcer	77	Time since last healthcare visit, mean(SD)		0.7 (1.2)	0	0.6 (0.9)	
Ulcer	77	Total cholesterol, mean(SD)		4.6 (1.1)	9.6	4.6 (1.1)	
Ulcer	78	Age at diagnosis of type 2 diabetes, mean(SD)		73.3 (3.1)	0	72.6 (3)	
Ulcer	78	BMI, mean(SD)		28.9 (4.9)	8.7	28.9 (4.9)	
Ulcer	78	HbA1c, mean(SD)		48.6 (10)	2.1	48.7 (9.7)	
Ulcer	78	HDL-cholesterol, mean(SD)		1.3 (0.4)	14.1	1.3 (0.4)	
Ulcer	78	Ischemic heart disease, n(%)	No	29306 (72.4%)	8	22982 (72.8%)	
Ulcer	78	Ischemic heart disease, n(%)	Yes	11169 (27.6%)	8	8587 (27.2%)	
Ulcer	78	Neuropathy, n(%)	No	31241 (71%)	0	20704 (65.6%)	
Ulcer	78	Neuropathy, n(%)	Yes	12755 (29%)	0	10865 (34.4%)	
Ulcer	78	Self-reported physical activity, n(%)	Never	6000 (15.5%)	12	4776 (15.1%)	
Ulcer	78	Self-reported physical activity, n(%)	<1 times/week	4784 (12.4%)	12	3750 (11.9%)	
Ulcer	78	Self-reported physical activity, n(%)	regularly - 1-2 times/week	7104 (18.4%)	12	5800 (18.4%)	
Ulcer	78	Self-reported physical activity, n(%)	regularly - 3-5 times/week	8337 (21.5%)	12	6881 (21.8%)	

Ulcer	78	Self-reported physical activity, n(%)	Daily	12479 (32.2%)	12	10362 (32.8%)	
Ulcer	78	Sex, n(%)	Males	23075 (52.5%)	0.1	16826 (53.3%)	n=43996 vs n=31569
Ulcer	78	Sex, n(%)	Females	20867 (47.5%)	0.1	14743 (46.7%)	
Ulcer	78	Smoking status, n(%)	No	37514 (92%)	7.3	29149 (92.3%)	
Ulcer	78	Smoking status, n(%)	Yes	3264 (8%)	7.3	2420 (7.7%)	
Ulcer	78	Stroke, n(%)	No	35524 (88.5%)	8.7	28057 (88.9%)	
Ulcer	78	Stroke, n(%)	Yes	4630 (11.5%)	8.7	3512 (11.1%)	
Ulcer	78	Systolic blood pressure, mean(SD)		136.4 (16)	2.6	136.1 (15.7)	
Ulcer	78	Time since last healthcare visit, mean(SD)		0.7 (1.2)	0	0.6 (0.9)	
Ulcer	78	Total cholesterol, mean(SD)		4.6 (1.1)	9.9	4.6 (1.1)	
Ulcer	79	Age at diagnosis of type 2 diabetes, mean(SD)		74.2 (3.1)	0	73.6 (3)	
Ulcer	79	BMI, mean(SD)		28.8 (4.9)	9.2	28.7 (4.8)	
Ulcer	79	HbA1c, mean(SD)		48.7 (10.1)	2.1	48.8 (9.8)	
Ulcer	79	HDL-cholesterol, mean(SD)		1.3 (0.4)	14.9	1.3 (0.4)	
Ulcer	79	Ischemic heart disease, n(%)	No	27077 (71.6%)	8.4	21016 (72%)	
Ulcer	79	Ischemic heart disease, n(%)	Yes	10736 (28.4%)	8.4	8169 (28%)	
Ulcer	79	Neuropathy, n(%)	No	28962 (70.2%)	0	18783 (64.4%)	
Ulcer	79	Neuropathy, n(%)	Yes	12307 (29.8%)	0	10402 (35.6%)	
Ulcer	79	Self-reported physical activity, n(%)	Never	5826 (16.1%)	12.4	4533 (15.5%)	
Ulcer	79	Self-reported physical activity, n(%)	<1 times/week	4588 (12.7%)	12.4	3524 (12.1%)	
Ulcer	79	Self-reported physical activity, n(%)	regularly - 1-2 times/week	6781 (18.8%)	12.4	5467 (18.7%)	
Ulcer	79	Self-reported physical activity, n(%)	regularly - 3-5 times/week	7575 (21%)	12.4	6265 (21.5%)	
Ulcer	79	Self-reported physical activity, n(%)	Daily	11363 (31.4%)	12.4	9396 (32.2%)	

Ulcer	79	Sex, n(%)	Males	21154 (51.3%)	0.1	15245 (52.2%)	n=41269 vs n=29185
Ulcer	79	Sex, n(%)	Females	20056 (48.7%)	0.1	13940 (47.8%)	
Ulcer	79	Smoking status, n(%)	No	35376 (92.7%)	7.5	27140 (93%)	
Ulcer	79	Smoking status, n(%)	Yes	2782 (7.3%)	7.5	2045 (7%)	
Ulcer	79	Stroke, n(%)	No	32920 (87.8%)	9.1	25770 (88.3%)	
Ulcer	79	Stroke, n(%)	Yes	4584 (12.2%)	9.1	3415 (11.7%)	
Ulcer	79	Systolic blood pressure, mean(SD)		136.4 (16.1)	2.6	136.1 (15.9)	
Ulcer	79	Time since last healthcare visit, mean(SD)		0.8 (1.2)	0	0.6 (1)	
Ulcer	79	Total cholesterol, mean(SD)		4.6 (1.1)	10.5	4.6 (1.1)	
Ulcer	80	Age at diagnosis of type 2 diabetes, mean(SD)		75.2 (3.1)	0	74.5 (3)	
Ulcer	80	BMI, mean(SD)		28.6 (4.8)	9.7	28.6 (4.8)	
Ulcer	80	HbA1c, mean(SD)		48.9 (10.3)	2.2	49 (9.9)	
Ulcer	80	HDL-cholesterol, mean(SD)		1.3 (0.4)	15.6	1.3 (0.4)	
Ulcer	80	Ischemic heart disease, n(%)	No	24842 (70.6%)	8.7	19161 (71.1%)	
Ulcer	80	Ischemic heart disease, n(%)	Yes	10338 (29.4%)	8.7	7771 (28.9%)	
Ulcer	80	Neuropathy, n(%)	No	26672 (69.2%)	0	16990 (63.1%)	
Ulcer	80	Neuropathy, n(%)	Yes	11844 (30.8%)	0	9942 (36.9%)	
Ulcer	80	Self-reported physical activity, n(%)	Never	5718 (17.1%)	13	4414 (16.4%)	
Ulcer	80	Self-reported physical activity, n(%)	<1 times/week	4436 (13.2%)	13	3426 (12.7%)	
Ulcer	80	Self-reported physical activity, n(%)	regularly - 1-2 times/week	6389 (19.1%)	13	5114 (19%)	
Ulcer	80	Self-reported physical activity, n(%)	regularly - 3-5 times/week	6864 (20.5%)	13	5636 (20.9%)	
Ulcer	80	Self-reported physical activity, n(%)	Daily	10110 (30.2%)	13	8342 (31%)	
Ulcer	80	Sex, n(%)	Males	19306 (50.2%)	0.2	13731 (51%)	n=38516 vs n=26932

Ulcer	80	Sex, n(%)	Females	19151 (49.8%)	0.2	13201 (49%)	
Ulcer	80	Smoking status, n(%)	No	33066 (93.3%)	8	25202 (93.6%)	
Ulcer	80	Smoking status, n(%)	Yes	2368 (6.7%)	8	1730 (6.4%)	
Ulcer	80	Stroke, n(%)	No	30471 (87.3%)	9.4	23671 (87.9%)	
Ulcer	80	Stroke, n(%)	Yes	4433 (12.7%)	9.4	3261 (12.1%)	
Ulcer	80	Systolic blood pressure, mean(SD)		136.5 (16.2)	2.8	136.2 (15.9)	
Ulcer	80	Time since last healthcare visit, mean(SD)		0.8 (1.3)	0	0.6 (1)	
Ulcer	80	Total cholesterol, mean(SD)		4.6 (1.1)	11	4.6 (1.1)	
Ulcer	81	Age at diagnosis of type 2 diabetes, mean(SD)		76.2 (3.1)	0	75.5 (3)	
Ulcer	81	BMI, mean(SD)		28.5 (4.8)	10.3	28.5 (4.8)	
Ulcer	81	HbA1c, mean(SD)		49.1 (10.4)	2.4	49.2 (10.1)	
Ulcer	81	HDL-cholesterol, mean(SD)		1.3 (0.4)	16.9	1.3 (0.4)	
Ulcer	81	Ischemic heart disease, n(%)	No	22620 (69.7%)	8.9	17165 (70.4%)	
Ulcer	81	Ischemic heart disease, n(%)	Yes	9820 (30.3%)	8.9	7224 (29.6%)	
Ulcer	81	Neuropathy, n(%)	No	24354 (68.4%)	0	15014 (61.6%)	
Ulcer	81	Neuropathy, n(%)	Yes	11247 (31.6%)	0	9375 (38.4%)	
Ulcer	81	Self-reported physical activity, n(%)	Never	5439 (17.7%)	13.7	4130 (16.9%)	
Ulcer	81	Self-reported physical activity, n(%)	<1 times/week	4234 (13.8%)	13.7	3217 (13.2%)	
Ulcer	81	Self-reported physical activity, n(%)	regularly - 1-2 times/week	5922 (19.3%)	13.7	4704 (19.3%)	
Ulcer	81	Self-reported physical activity, n(%)	regularly - 3-5 times/week	6048 (19.7%)	13.7	4934 (20.2%)	
Ulcer	81	Self-reported physical activity, n(%)	Daily	9076 (29.5%)	13.7	7404 (30.4%)	
Ulcer	81	Sex, n(%)	Males	17477 (49.2%)	0.2	12240 (50.2%)	n=35601 vs n=24389
Ulcer	81	Sex, n(%)	Females	18066 (50.8%)	0.2	12149 (49.8%)	

Ulcer	81	Smoking status, n(%)	No	30569 (93.8%)	8.5	22947 (94.1%)	
Ulcer	81	Smoking status, n(%)	Yes	2017 (6.2%)	8.5	1442 (5.9%)	
Ulcer	81	Stroke, n(%)	No	27862 (86.7%)	9.7	21258 (87.2%)	
Ulcer	81	Stroke, n(%)	Yes	4274 (13.3%)	9.7	3131 (12.8%)	
Ulcer	81	Systolic blood pressure, mean(SD)		136.6 (16.4)	2.9	136.3 (16.1)	
Ulcer	81	Time since last healthcare visit, mean(SD)		0.8 (1.3)	0	0.6 (1)	
Ulcer	81	Total cholesterol, mean(SD)		4.7 (1.1)	11.9	4.6 (1.1)	
Ulcer	82	Age at diagnosis of type 2 diabetes, mean(SD)		77.2 (3.1)	0	76.4 (3)	
Ulcer	82	BMI, mean(SD)		28.4 (4.8)	10.6	28.4 (4.7)	
Ulcer	82	HbA1c, mean(SD)		49.3 (10.5)	2.3	49.5 (10.2)	
Ulcer	82	HDL-cholesterol, mean(SD)		1.3 (0.4)	17.9	1.3 (0.4)	
Ulcer	82	Ischemic heart disease, n(%)	No	20394 (68.9%)	9	15252 (69.4%)	
Ulcer	82	Ischemic heart disease, n(%)	Yes	9214 (31.1%)	9	6725 (30.6%)	
Ulcer	82	Neuropathy, n(%)	No	21972 (67.5%)	0	13290 (60.5%)	
Ulcer	82	Neuropathy, n(%)	Yes	10569 (32.5%)	0	8687 (39.5%)	
Ulcer	82	Self-reported physical activity, n(%)	Never	5270 (18.9%)	14.2	3939 (17.9%)	
Ulcer	82	Self-reported physical activity, n(%)	<1 times/week	3891 (13.9%)	14.2	2946 (13.4%)	
Ulcer	82	Self-reported physical activity, n(%)	regularly - 1-2 times/week	5399 (19.3%)	14.2	4244 (19.3%)	
Ulcer	82	Self-reported physical activity, n(%)	regularly - 3-5 times/week	5463 (19.6%)	14.2	4466 (20.3%)	
Ulcer	82	Self-reported physical activity, n(%)	Daily	7913 (28.3%)	14.2	6382 (29%)	
Ulcer	82	Sex, n(%)	Males	15661 (48.2%)	0.2	10797 (49.1%)	n=32541 vs n=21977
Ulcer	82	Sex, n(%)	Females	16824 (51.8%)	0.2	11180 (50.9%)	
Ulcer	82	Smoking status, n(%)	No	28026 (94.4%)	8.8	20840 (94.8%)	

Ulcer	82	Smoking status, n(%)	Yes	1654 (5.6%)	8.8	1137 (5.2%)	
Ulcer	82	Stroke, n(%)	No	25237 (86.1%)	9.9	19007 (86.5%)	
Ulcer	82	Stroke, n(%)	Yes	4071 (13.9%)	9.9	2970 (13.5%)	
Ulcer	82	Systolic blood pressure, mean(SD)		136.8 (16.6)	3	136.5 (16.3)	
Ulcer	82	Time since last healthcare visit, mean(SD)		0.8 (1.3)	0	0.7 (1)	
Ulcer	82	Total cholesterol, mean(SD)		4.7 (1.1)	12.6	4.6 (1.1)	
Ulcer	83	Age at diagnosis of type 2 diabetes, mean(SD)		78.1 (3.1)	0	77.3 (3)	
Ulcer	83	BMI, mean(SD)		28.2 (4.7)	11	28.2 (4.7)	
Ulcer	83	HbA1c, mean(SD)		49.4 (10.5)	2.3	49.6 (10.3)	
Ulcer	83	HDL-cholesterol, mean(SD)		1.3 (0.4)	19.1	1.3 (0.4)	
Ulcer	83	Ischemic heart disease, n(%)	No	18253 (67.9%)	9.2	13478 (68.5%)	
Ulcer	83	Ischemic heart disease, n(%)	Yes	8643 (32.1%)	9.2	6197 (31.5%)	
Ulcer	83	Neuropathy, n(%)	No	19670 (66.4%)	0	11596 (58.9%)	
Ulcer	83	Neuropathy, n(%)	Yes	9937 (33.6%)	0	8079 (41.1%)	
Ulcer	83	Self-reported physical activity, n(%)	Never	5036 (20%)	14.8	3737 (19%)	
Ulcer	83	Self-reported physical activity, n(%)	<1 times/week	3495 (13.9%)	14.8	2622 (13.3%)	
Ulcer	83	Self-reported physical activity, n(%)	regularly - 1-2 times/week	5018 (19.9%)	14.8	3938 (20%)	
Ulcer	83	Self-reported physical activity, n(%)	regularly - 3-5 times/week	4746 (18.8%)	14.8	3801 (19.3%)	
Ulcer	83	Self-reported physical activity, n(%)	Daily	6932 (27.5%)	14.8	5577 (28.3%)	
Ulcer	83	Sex, n(%)	Males	13930 (47.1%)	0.2	9418 (47.9%)	n=29607 vs n=19675
Ulcer	83	Sex, n(%)	Females	15627 (52.9%)	0.2	10257 (52.1%)	
Ulcer	83	Smoking status, n(%)	No	25482 (94.7%)	9.1	18699 (95%)	
Ulcer	83	Smoking status, n(%)	Yes	1430 (5.3%)	9.1	976 (5%)	

Ulcer	83	Stroke, n(%)	No	22770 (85.5%)	10	16878 (85.8%)	
Ulcer	83	Stroke, n(%)	Yes	3864 (14.5%)	10	2797 (14.2%)	
Ulcer	83	Systolic blood pressure, mean(SD)		136.8 (16.9)	2.9	136.3 (16.5)	
Ulcer	83	Time since last healthcare visit, mean(SD)		0.8 (1.3)	0	0.7 (1.1)	
Ulcer	83	Total cholesterol, mean(SD)		4.7 (1.1)	13.5	4.6 (1.1)	
Ulcer	84	Age at diagnosis of type 2 diabetes, mean(SD)		79.1 (3.1)	0	78.2 (3)	
Ulcer	84	BMI, mean(SD)		28 (4.6)	11.9	28.1 (4.6)	
Ulcer	84	HbA1c, mean(SD)		49.5 (10.7)	2.7	49.7 (10.4)	
Ulcer	84	HDL-cholesterol, mean(SD)		1.3 (0.4)	20.3	1.3 (0.4)	
Ulcer	84	Ischemic heart disease, n(%)	No	16120 (66.7%)	9.4	11616 (67.1%)	
Ulcer	84	Ischemic heart disease, n(%)	Yes	8036 (33.3%)	9.4	5694 (32.9%)	
Ulcer	84	Neuropathy, n(%)	No	17490 (65.6%)	0	9920 (57.3%)	
Ulcer	84	Neuropathy, n(%)	Yes	9160 (34.4%)	0	7390 (42.7%)	
Ulcer	84	Self-reported physical activity, n(%)	Never	4823 (21.5%)	15.7	3556 (20.5%)	
Ulcer	84	Self-reported physical activity, n(%)	<1 times/week	3252 (14.5%)	15.7	2407 (13.9%)	
Ulcer	84	Self-reported physical activity, n(%)	regularly - 1-2 times/week	4362 (19.4%)	15.7	3354 (19.4%)	
Ulcer	84	Self-reported physical activity, n(%)	regularly - 3-5 times/week	4145 (18.4%)	15.7	3299 (19.1%)	
Ulcer	84	Self-reported physical activity, n(%)	Daily	5891 (26.2%)	15.7	4694 (27.1%)	
Ulcer	84	Sex, n(%)	Males	12205 (45.9%)	0.2	8079 (46.7%)	n=26650 vs n=17310
Ulcer	84	Sex, n(%)	Females	14403 (54.1%)	0.2	9231 (53.3%)	
Ulcer	84	Smoking status, n(%)	No	22843 (95.1%)	9.9	16528 (95.5%)	
Ulcer	84	Smoking status, n(%)	Yes	1169 (4.9%)	9.9	782 (4.5%)	
Ulcer	84	Stroke, n(%)	No	20348 (85.1%)	10.3	14806 (85.5%)	

Ulcer	84	Stroke, n(%)	Yes	3564 (14.9%)	10.3	2504 (14.5%)	
Ulcer	84	Systolic blood pressure, mean(SD)		136.9 (17)	3.3	136.5 (16.7)	
Ulcer	84	Time since last healthcare visit, mean(SD)		0.9 (1.4)	0	0.7 (1.1)	
Ulcer	84	Total cholesterol, mean(SD)		4.7 (1.1)	14.6	4.7 (1.1)	
Ulcer	85	Age at diagnosis of type 2 diabetes, mean(SD)		80.1 (3.1)	0	79.2 (3)	
Ulcer	85	BMI, mean(SD)		27.9 (4.6)	12.6	27.9 (4.6)	
Ulcer	85	HbA1c, mean(SD)		49.6 (10.6)	2.8	49.7 (10.2)	
Ulcer	85	HDL-cholesterol, mean(SD)		1.3 (0.4)	21.4	1.3 (0.4)	
Ulcer	85	Ischemic heart disease, n(%)	No	14195 (65.8%)	9.7	10019 (66.1%)	
Ulcer	85	Ischemic heart disease, n(%)	Yes	7362 (34.2%)	9.7	5129 (33.9%)	
Ulcer	85	Neuropathy, n(%)	No	15451 (64.7%)	0	8478 (56%)	
Ulcer	85	Neuropathy, n(%)	Yes	8424 (35.3%)	0	6670 (44%)	
Ulcer	85	Self-reported physical activity, n(%)	Never	4459 (22.4%)	16.5	3252 (21.5%)	
Ulcer	85	Self-reported physical activity, n(%)	<1 times/week	2970 (14.9%)	16.5	2178 (14.4%)	
Ulcer	85	Self-reported physical activity, n(%)	regularly - 1-2 times/week	3908 (19.6%)	16.5	2987 (19.7%)	
Ulcer	85	Self-reported physical activity, n(%)	regularly - 3-5 times/week	3530 (17.7%)	16.5	2786 (18.4%)	
Ulcer	85	Self-reported physical activity, n(%)	Daily	5058 (25.4%)	16.5	3945 (26%)	
Ulcer	85	Sex, n(%)	Males	10645 (44.7%)	0.2	6916 (45.7%)	n=23875 vs n=15148
Ulcer	85	Sex, n(%)	Females	13190 (55.3%)	0.2	8232 (54.3%)	
Ulcer	85	Smoking status, n(%)	No	20427 (95.6%)	10.5	14534 (95.9%)	
Ulcer	85	Smoking status, n(%)	Yes	941 (4.4%)	10.5	614 (4.1%)	
Ulcer	85	Stroke, n(%)	No	18056 (84.6%)	10.6	12875 (85%)	
Ulcer	85	Stroke, n(%)	Yes	3291 (15.4%)	10.6	2273 (15%)	

Ulcer	85	Systolic blood pressure, mean(SD)		137.1 (17.1)	3.2	136.8 (16.8)	
Ulcer	85	Time since last healthcare visit, mean(SD)		0.9 (1.4)	0	0.7 (1.1)	
Ulcer	85	Total cholesterol, mean(SD)		4.7 (1.2)	15.8	4.7 (1.1)	
Ulcer	86	Age at diagnosis of type 2 diabetes, mean(SD)		81 (3.1)	0	80.1 (3)	
Ulcer	86	BMI, mean(SD)		27.7 (4.6)	13.4	27.7 (4.6)	
Ulcer	86	HbA1c, mean(SD)		49.8 (10.9)	3	49.9 (10.6)	
Ulcer	86	HDL-cholesterol, mean(SD)		1.3 (0.4)	23	1.3 (0.4)	
Ulcer	86	Ischemic heart disease, n(%)	No	12404 (65.3%)	9.9	8554 (65.5%)	
Ulcer	86	Ischemic heart disease, n(%)	Yes	6587 (34.7%)	9.9	4504 (34.5%)	
Ulcer	86	Neuropathy, n(%)	No	13498 (64%)	0	7109 (54.4%)	
Ulcer	86	Neuropathy, n(%)	Yes	7584 (36%)	0	5949 (45.6%)	
Ulcer	86	Self-reported physical activity, n(%)	Never	4047 (23.2%)	17.4	2885 (22.1%)	
Ulcer	86	Self-reported physical activity, n(%)	<1 times/week	2618 (15%)	17.4	1904 (14.6%)	
Ulcer	86	Self-reported physical activity, n(%)	regularly - 1-2 times/week	3407 (19.6%)	17.4	2564 (19.6%)	
Ulcer	86	Self-reported physical activity, n(%)	regularly - 3-5 times/week	3038 (17.4%)	17.4	2364 (18.1%)	
Ulcer	86	Self-reported physical activity, n(%)	Daily	4311 (24.7%)	17.4	3341 (25.6%)	
Ulcer	86	Sex, n(%)	Males	9071 (43.1%)	0.1	5792 (44.4%)	n=21082 vs n=13058
Ulcer	86	Sex, n(%)	Females	11981 (56.9%)	0.1	7266 (55.6%)	
Ulcer	86	Smoking status, n(%)	No	18003 (95.9%)	10.9	12562 (96.2%)	
Ulcer	86	Smoking status, n(%)	Yes	776 (4.1%)	10.9	496 (3.8%)	
Ulcer	86	Stroke, n(%)	No	15853 (84.3%)	10.8	11032 (84.5%)	
Ulcer	86	Stroke, n(%)	Yes	2948 (15.7%)	10.8	2026 (15.5%)	
Ulcer	86	Systolic blood pressure, mean(SD)		137.2 (17.3)	3.4	136.9 (17.1)	

Ulcer	86	Time since last healthcare visit, mean(SD)		0.9 (1.5)	0	0.8 (1.2)	
Ulcer	86	Total cholesterol, mean(SD)		4.7 (1.1)	17.2	4.7 (1.1)	
Ulcer	87	Age at diagnosis of type 2 diabetes, mean(SD)		82 (3.1)	0	81 (3)	
Ulcer	87	BMI, mean(SD)		27.5 (4.5)	14.4	27.5 (4.4)	
Ulcer	87	HbA1c, mean(SD)		49.9 (10.9)	3.1	50 (10.6)	
Ulcer	87	HDL-cholesterol, mean(SD)		1.3 (0.4)	25.1	1.4 (0.4)	
Ulcer	87	Ischemic heart disease, n(%)	No	10537 (64.4%)	10.5	7128 (65%)	
Ulcer	87	Ischemic heart disease, n(%)	Yes	5813 (35.6%)	10.5	3840 (35%)	
Ulcer	87	Neuropathy, n(%)	No	11594 (63.5%)	0	5810 (53%)	
Ulcer	87	Neuropathy, n(%)	Yes	6674 (36.5%)	0	5158 (47%)	
Ulcer	87	Self-reported physical activity, n(%)	Never	3630 (24.5%)	18.8	2548 (23.2%)	
Ulcer	87	Self-reported physical activity, n(%)	<1 times/week	2259 (15.2%)	18.8	1600 (14.6%)	
Ulcer	87	Self-reported physical activity, n(%)	regularly - 1-2 times/week	2982 (20.1%)	18.8	2199 (20%)	
Ulcer	87	Self-reported physical activity, n(%)	regularly - 3-5 times/week	2560 (17.3%)	18.8	1987 (18.1%)	
Ulcer	87	Self-reported physical activity, n(%)	Daily	3406 (23%)	18.8	2634 (24%)	
Ulcer	87	Sex, n(%)	Males	7625 (41.8%)	0.2	4710 (42.9%)	n=18268 vs n=10968
Ulcer	87	Sex, n(%)	Females	10613 (58.2%)	0.2	6258 (57.1%)	
Ulcer	87	Smoking status, n(%)	No	15526 (96.3%)	11.8	10609 (96.7%)	
Ulcer	87	Smoking status, n(%)	Yes	594 (3.7%)	11.8	359 (3.3%)	
Ulcer	87	Stroke, n(%)	No	13559 (83.8%)	11.5	9261 (84.4%)	
Ulcer	87	Stroke, n(%)	Yes	2612 (16.2%)	11.5	1707 (15.6%)	
Ulcer	87	Systolic blood pressure, mean(SD)		137.3 (17.5)	3.8	137 (17.2)	
Ulcer	87	Time since last healthcare visit, mean(SD)		1 (1.5)	0	0.8 (1.2)	

Ulcer	87	Total cholesterol, mean(SD)		4.8 (1.2)	19	4.7 (1.2)	
Ulcer	88	Age at diagnosis of type 2 diabetes, mean(SD)		82.9 (3.1)	0	81.9 (3)	
Ulcer	88	BMI, mean(SD)		27.3 (4.5)	15.5	27.3 (4.4)	
Ulcer	88	HbA1c, mean(SD)		50 (11)	3.1	50.2 (10.7)	
Ulcer	88	HDL-cholesterol, mean(SD)		1.3 (0.4)	26.9	1.4 (0.4)	
Ulcer	88	Ischemic heart disease, n(%)	No	9004 (64%)	10.6	5859 (64.2%)	
Ulcer	88	Ischemic heart disease, n(%)	Yes	5062 (36%)	10.6	3267 (35.8%)	
Ulcer	88	Neuropathy, n(%)	No	9953 (63.3%)	0	4732 (51.9%)	
Ulcer	88	Neuropathy, n(%)	Yes	5779 (36.7%)	0	4394 (48.1%)	
Ulcer	88	Self-reported physical activity, n(%)	Never	3300 (26.2%)	19.9	2239 (24.5%)	
Ulcer	88	Self-reported physical activity, n(%)	<1 times/week	2036 (16.2%)	19.9	1440 (15.8%)	
Ulcer	88	Self-reported physical activity, n(%)	regularly - 1-2 times/week	2419 (19.2%)	19.9	1794 (19.7%)	
Ulcer	88	Self-reported physical activity, n(%)	regularly - 3-5 times/week	2002 (15.9%)	19.9	1516 (16.6%)	
Ulcer	88	Self-reported physical activity, n(%)	Daily	2848 (22.6%)	19.9	2137 (23.4%)	
Ulcer	88	Sex, n(%)	Males	6402 (40.8%)	0.2	3865 (42.4%)	n=15732 vs n=9126
Ulcer	88	Sex, n(%)	Females	9306 (59.2%)	0.2	5261 (57.6%)	
Ulcer	88	Smoking status, n(%)	No	13301 (96.6%)	12.5	8848 (97%)	
Ulcer	88	Smoking status, n(%)	Yes	464 (3.4%)	12.5	278 (3%)	
Ulcer	88	Stroke, n(%)	No	11607 (83.5%)	11.6	7679 (84.1%)	
Ulcer	88	Stroke, n(%)	Yes	2293 (16.5%)	11.6	1447 (15.9%)	
Ulcer	88	Systolic blood pressure, mean(SD)		137.4 (17.6)	4	136.8 (17.1)	
Ulcer	88	Time since last healthcare visit, mean(SD)		1.1 (1.6)	0	0.8 (1.3)	
Ulcer	88	Total cholesterol, mean(SD)		4.8 (1.2)	20.7	4.7 (1.2)	

Ulcer	89	Age at diagnosis of type 2 diabetes, mean(SD)		83.8 (3.1)	0	82.8 (2.9)	
Ulcer	89	BMI, mean(SD)		27.1 (4.5)	16.9	27 (4.3)	
Ulcer	89	HbA1c, mean(SD)		50.2 (11.1)	3.4	50.2 (10.5)	
Ulcer	89	HDL-cholesterol, mean(SD)		1.3 (0.4)	28.9	1.4 (0.4)	
Ulcer	89	Ischemic heart disease, n(%)	No	7327 (63.3%)	11	4600 (63.2%)	
Ulcer	89	Ischemic heart disease, n(%)	Yes	4256 (36.7%)	11	2679 (36.8%)	
Ulcer	89	Neuropathy, n(%)	No	8204 (63.1%)	0	3674 (50.5%)	
Ulcer	89	Neuropathy, n(%)	Yes	4806 (36.9%)	0	3605 (49.5%)	
Ulcer	89	Self-reported physical activity, n(%)	Never	2785 (27.1%)	21.1	1829 (25.1%)	
Ulcer	89	Self-reported physical activity, n(%)	<1 times/week	1712 (16.7%)	21.1	1196 (16.4%)	
Ulcer	89	Self-reported physical activity, n(%)	regularly - 1-2 times/week	1926 (18.8%)	21.1	1376 (18.9%)	
Ulcer	89	Self-reported physical activity, n(%)	regularly - 3-5 times/week	1596 (15.5%)	21.1	1200 (16.5%)	
Ulcer	89	Self-reported physical activity, n(%)	Daily	2250 (21.9%)	21.1	1678 (23.1%)	
Ulcer	89	Sex, n(%)	Males	5118 (39.4%)	0.2	2977 (40.9%)	n=13010 vs n=7279
Ulcer	89	Sex, n(%)	Females	7871 (60.6%)	0.2	4302 (59.1%)	
Ulcer	89	Smoking status, n(%)	No	10906 (96.9%)	13.5	7071 (97.1%)	
Ulcer	89	Smoking status, n(%)	Yes	348 (3.1%)	13.5	208 (2.9%)	
Ulcer	89	Stroke, n(%)	No	9640 (84.3%)	12.1	6170 (84.8%)	
Ulcer	89	Stroke, n(%)	Yes	1797 (15.7%)	12.1	1109 (15.2%)	
Ulcer	89	Systolic blood pressure, mean(SD)		137.4 (17.8)	4.3	137.1 (17.5)	
Ulcer	89	Time since last healthcare visit, mean(SD)		1.1 (1.6)	0	0.9 (1.3)	
Ulcer	89	Total cholesterol, mean(SD)		4.8 (1.2)	22.5	4.8 (1.2)	
Ulcer	90	Age at diagnosis of type 2 diabetes, mean(SD)		84.9 (3.1)	0	83.7 (2.9)	

Ulcer	90	BMI, mean(SD)		26.8 (4.3)	18.1	26.8 (4.3)	
Ulcer	90	HbA1c, mean(SD)		50.3 (11.2)	3.7	50.3 (10.8)	
Ulcer	90	HDL-cholesterol, mean(SD)		1.4 (0.4)	30.7	1.4 (0.4)	
Ulcer	90	Ischemic heart disease, n(%)	No	5863 (62.9%)	10.9	3541 (62.7%)	
Ulcer	90	Ischemic heart disease, n(%)	Yes	3460 (37.1%)	10.9	2103 (37.3%)	
Ulcer	90	Neuropathy, n(%)	No	6679 (63.8%)	0	2834 (50.2%)	
Ulcer	90	Neuropathy, n(%)	Yes	3786 (36.2%)	0	2810 (49.8%)	
Ulcer	90	Self-reported physical activity, n(%)	Never	2278 (28%)	22.4	1479 (26.2%)	
Ulcer	90	Self-reported physical activity, n(%)	<1 times/week	1367 (16.8%)	22.4	932 (16.5%)	
Ulcer	90	Self-reported physical activity, n(%)	regularly - 1-2 times/week	1528 (18.8%)	22.4	1050 (18.6%)	
Ulcer	90	Self-reported physical activity, n(%)	regularly - 3-5 times/week	1199 (14.8%)	22.4	871 (15.4%)	
Ulcer	90	Self-reported physical activity, n(%)	Daily	1752 (21.6%)	22.4	1312 (23.2%)	
Ulcer	90	Sex, n(%)	Males	3970 (38%)	0.2	2281 (40.4%)	n=10465 vs n=5644
Ulcer	90	Sex, n(%)	Females	6476 (62%)	0.2	3363 (59.6%)	
Ulcer	90	Smoking status, n(%)	No	8668 (97.2%)	14.8	5509 (97.6%)	
Ulcer	90	Smoking status, n(%)	Yes	247 (2.8%)	14.8	135 (2.4%)	
Ulcer	90	Stroke, n(%)	No	7770 (84.6%)	12.3	4817 (85.3%)	
Ulcer	90	Stroke, n(%)	Yes	1410 (15.4%)	12.3	827 (14.7%)	
Ulcer	90	Systolic blood pressure, mean(SD)		137.9 (17.9)	4.4	137.8 (17.6)	
Ulcer	90	Time since last healthcare visit, mean(SD)		1.2 (1.6)	0	0.9 (1.4)	
Ulcer	90	Total cholesterol, mean(SD)		4.8 (1.2)	24.6	4.8 (1.2)	
Ulcer	91	Age at diagnosis of type 2 diabetes, mean(SD)		85.8 (3.1)	0	84.6 (2.9)	
Ulcer	91	BMI, mean(SD)		26.6 (4.3)	19.2	26.6 (4.2)	

Ulcer	91	HbA1c, mean(SD)		50.6 (11.5)	4.1	50.7 (11)	
Ulcer	91	HDL-cholesterol, mean(SD)		1.4 (0.4)	32.6	1.3 (0.4)	
Ulcer	91	Ischemic heart disease, n(%)	No	4639 (62.6%)	10.7	2697 (62.4%)	
Ulcer	91	Ischemic heart disease, n(%)	Yes	2771 (37.4%)	10.7	1624 (37.6%)	
Ulcer	91	Neuropathy, n(%)	No	5336 (64.3%)	0	2158 (49.9%)	
Ulcer	91	Neuropathy, n(%)	Yes	2963 (35.7%)	0	2163 (50.1%)	
Ulcer	91	Self-reported physical activity, n(%)	Never	1865 (29.6%)	24.1	1185 (27.4%)	
Ulcer	91	Self-reported physical activity, n(%)	<1 times/week	1031 (16.4%)	24.1	700 (16.2%)	
Ulcer	91	Self-reported physical activity, n(%)	regularly - 1-2 times/week	1188 (18.9%)	24.1	820 (19%)	
Ulcer	91	Self-reported physical activity, n(%)	regularly - 3-5 times/week	894 (14.2%)	24.1	649 (15%)	
Ulcer	91	Self-reported physical activity, n(%)	Daily	1321 (21%)	24.1	967 (22.4%)	
Ulcer	91	Sex, n(%)	Males	3021 (36.5%)	0.2	1729 (40%)	n=8299 vs n=4321
Ulcer	91	Sex, n(%)	Females	5265 (63.5%)	0.2	2592 (60%)	
Ulcer	91	Smoking status, n(%)	No	6786 (97.5%)	16.1	4222 (97.7%)	
Ulcer	91	Smoking status, n(%)	Yes	175 (2.5%)	16.1	99 (2.3%)	
Ulcer	91	Stroke, n(%)	No	6139 (84.3%)	12.2	3667 (84.9%)	
Ulcer	91	Stroke, n(%)	Yes	1147 (15.7%)	12.2	654 (15.1%)	
Ulcer	91	Systolic blood pressure, mean(SD)		137.8 (18)	4.5	137.7 (17.6)	
Ulcer	91	Time since last healthcare visit, mean(SD)		1.2 (1.7)	0	1 (1.4)	
Ulcer	91	Total cholesterol, mean(SD)		4.9 (1.2)	26.4	4.8 (1.2)	
Ulcer	92	Age at diagnosis of type 2 diabetes, mean(SD)		86.7 (3.1)	0	85.4 (2.9)	
Ulcer	92	BMI, mean(SD)		26.4 (4.3)	21.1	26.4 (4.3)	
Ulcer	92	HbA1c, mean(SD)		50.6 (11.5)	4.2	50.7 (11.2)	

Ulcer	92	HDL-cholesterol, mean(SD)		1.4 (0.4)	35.2	1.4 (0.4)	
Ulcer	92	Ischemic heart disease, n(%)	No	3503 (62.4%)	11.7	1968 (63.1%)	
Ulcer	92	Ischemic heart disease, n(%)	Yes	2115 (37.6%)	11.7	1150 (36.9%)	
Ulcer	92	Neuropathy, n(%)	No	4149 (65.2%)	0	1534 (49.2%)	
Ulcer	92	Neuropathy, n(%)	Yes	2212 (34.8%)	0	1584 (50.8%)	
Ulcer	92	Self-reported physical activity, n(%)	Never	1439 (30.7%)	26.4	884 (28.4%)	
Ulcer	92	Self-reported physical activity, n(%)	<1 times/week	768 (16.4%)	26.4	502 (16.1%)	
Ulcer	92	Self-reported physical activity, n(%)	regularly - 1-2 times/week	886 (18.9%)	26.4	594 (19.1%)	
Ulcer	92	Self-reported physical activity, n(%)	regularly - 3-5 times/week	632 (13.5%)	26.4	449 (14.4%)	
Ulcer	92	Self-reported physical activity, n(%)	Daily	955 (20.4%)	26.4	689 (22.1%)	
Ulcer	92	Sex, n(%)	Males	2261 (35.6%)	0.1	1229 (39.4%)	n=6361 vs n=3118
Ulcer	92	Sex, n(%)	Females	4092 (64.4%)	0.1	1889 (60.6%)	
Ulcer	92	Smoking status, n(%)	No	5116 (97.8%)	17.8	3061 (98.2%)	
Ulcer	92	Smoking status, n(%)	Yes	114 (2.2%)	17.8	57 (1.8%)	
Ulcer	92	Stroke, n(%)	No	4650 (84.3%)	13.2	2638 (84.6%)	
Ulcer	92	Stroke, n(%)	Yes	869 (15.7%)	13.2	480 (15.4%)	
Ulcer	92	Systolic blood pressure, mean(SD)		137.9 (18)	5.1	137.4 (17.5)	
Ulcer	92	Time since last healthcare visit, mean(SD)		1.3 (1.7)	0	1 (1.5)	
Ulcer	92	Total cholesterol, mean(SD)		4.9 (1.2)	28.9	4.9 (1.2)	
Ulcer	93	Age at diagnosis of type 2 diabetes, mean(SD)		87.6 (3.1)	0	86.3 (2.8)	
Ulcer	93	BMI, mean(SD)		26.3 (4.3)	22.7	26.2 (4.2)	
Ulcer	93	HbA1c, mean(SD)		50.6 (11.5)	5.1	50.8 (11)	
Ulcer	93	HDL-cholesterol, mean(SD)		1.4 (0.4)	37.3	1.4 (0.4)	

Ulcer	93	Ischemic heart disease, n(%)	No	2620 (63%)	12.2	1417 (64.2%)	
Ulcer	93	Ischemic heart disease, n(%)	Yes	1537 (37%)	12.2	789 (35.8%)	
Ulcer	93	Neuropathy, n(%)	No	3077 (65%)	0	1052 (47.7%)	
Ulcer	93	Neuropathy, n(%)	Yes	1655 (35%)	0	1154 (52.3%)	
Ulcer	93	Self-reported physical activity, n(%)	Never	1086 (32.1%)	28.6	630 (28.6%)	
Ulcer	93	Self-reported physical activity, n(%)	<1 times/week	527 (15.6%)	28.6	360 (16.3%)	
Ulcer	93	Self-reported physical activity, n(%)	regularly - 1-2 times/week	620 (18.3%)	28.6	400 (18.1%)	
Ulcer	93	Self-reported physical activity, n(%)	regularly - 3-5 times/week	462 (13.7%)	28.6	320 (14.5%)	
Ulcer	93	Self-reported physical activity, n(%)	Daily	685 (20.3%)	28.6	496 (22.5%)	
Ulcer	93	Sex, n(%)	Males	1649 (34.9%)	0.1	848 (38.4%)	n=4732 vs n=2206
Ulcer	93	Sex, n(%)	Females	3077 (65.1%)	0.1	1358 (61.6%)	
Ulcer	93	Smoking status, n(%)	No	3770 (98.1%)	18.8	2171 (98.4%)	
Ulcer	93	Smoking status, n(%)	Yes	74 (1.9%)	18.8	35 (1.6%)	
Ulcer	93	Stroke, n(%)	No	3458 (84.9%)	14	1892 (85.8%)	
Ulcer	93	Stroke, n(%)	Yes	613 (15.1%)	14	314 (14.2%)	
Ulcer	93	Systolic blood pressure, mean(SD)		138 (18.5)	5.9	137.2 (18.2)	
Ulcer	93	Time since last healthcare visit, mean(SD)		1.4 (1.8)	0	1.1 (1.5)	
Ulcer	93	Total cholesterol, mean(SD)		4.9 (1.2)	31.1	4.9 (1.2)	
Ulcer	94	Age at diagnosis of type 2 diabetes, mean(SD)		88.6 (3.1)	0	87.2 (2.8)	
Ulcer	94	BMI, mean(SD)		26 (4.2)	24.7	26 (4.1)	
Ulcer	94	HbA1c, mean(SD)		50.6 (11.4)	5.5	50.9 (11.4)	
Ulcer	94	HDL-cholesterol, mean(SD)		1.4 (0.4)	40.1	1.4 (0.4)	
Ulcer	94	Ischemic heart disease, n(%)	No	1882 (62.8%)	13	956 (63.4%)	

Ulcer	94	Ischemic heart disease, n(%)	Yes	1113 (37.2%)	13	551 (36.6%)	
Ulcer	94	Neuropathy, n(%)	No	2256 (65.6%)	0	719 (47.7%)	
Ulcer	94	Neuropathy, n(%)	Yes	1185 (34.4%)	0	788 (52.3%)	
Ulcer	94	Self-reported physical activity, n(%)	Never	796 (33.4%)	30.7	439 (29.1%)	
Ulcer	94	Self-reported physical activity, n(%)	<1 times/week	393 (16.5%)	30.7	255 (16.9%)	
Ulcer	94	Self-reported physical activity, n(%)	regularly - 1-2 times/week	425 (17.8%)	30.7	280 (18.6%)	
Ulcer	94	Self-reported physical activity, n(%)	regularly - 3-5 times/week	312 (13.1%)	30.7	217 (14.4%)	
Ulcer	94	Self-reported physical activity, n(%)	Daily	459 (19.2%)	30.7	316 (21%)	
Ulcer	94	Sex, n(%)	Males	1114 (32.4%)	0.2	545 (36.2%)	n=3441 vs n=1507
Ulcer	94	Sex, n(%)	Females	2320 (67.6%)	0.2	962 (63.8%)	
Ulcer	94	Smoking status, n(%)	No	2686 (97.8%)	20.2	1478 (98.1%)	
Ulcer	94	Smoking status, n(%)	Yes	61 (2.2%)	20.2	29 (1.9%)	
Ulcer	94	Stroke, n(%)	No	2476 (84.4%)	14.8	1294 (85.9%)	
Ulcer	94	Stroke, n(%)	Yes	456 (15.6%)	14.8	213 (14.1%)	
Ulcer	94	Systolic blood pressure, mean(SD)		138.3 (18.2)	6.2	137.6 (17.8)	
Ulcer	94	Time since last healthcare visit, mean(SD)		1.5 (1.9)	0	1.2 (1.6)	
Ulcer	94	Total cholesterol, mean(SD)		4.9 (1.2)	33.5	4.9 (1.2)	
Ulcer	95	Age at diagnosis of type 2 diabetes, mean(SD)		89.5 (3.1)	0	87.9 (2.8)	
Ulcer	95	BMI, mean(SD)		25.9 (4.2)	26.3	25.8 (4.1)	
Ulcer	95	HbA1c, mean(SD)		50.7 (11.4)	6.6	50.7 (11.2)	
Ulcer	95	HDL-cholesterol, mean(SD)		1.4 (0.4)	43.6	1.4 (0.4)	
Ulcer	95	Ischemic heart disease, n(%)	No	1345 (63.7%)	13.7	634 (64%)	
Ulcer	95	Ischemic heart disease, n(%)	Yes	765 (36.3%)	13.7	357 (36%)	

Ulcer	95	Neuropathy, n(%)	No	1637 (67%)	0	472 (47.6%)	
Ulcer	95	Neuropathy, n(%)	Yes	808 (33%)	0	519 (52.4%)	
Ulcer	95	Self-reported physical activity, n(%)	Never	579 (35.4%)	33.2	297 (30%)	
Ulcer	95	Self-reported physical activity, n(%)	<1 times/week	238 (14.6%)	33.2	144 (14.5%)	
Ulcer	95	Self-reported physical activity, n(%)	regularly - 1-2 times/week	278 (17%)	33.2	172 (17.4%)	
Ulcer	95	Self-reported physical activity, n(%)	regularly - 3-5 times/week	223 (13.6%)	33.2	152 (15.3%)	
Ulcer	95	Self-reported physical activity, n(%)	Daily	316 (19.3%)	33.2	226 (22.8%)	
Ulcer	95	Sex, n(%)	Males	727 (29.8%)	0.2	331 (33.4%)	n=2445 vs n=991
Ulcer	95	Sex, n(%)	Females	1712 (70.2%)	0.2	660 (66.6%)	
Ulcer	95	Smoking status, n(%)	No	1866 (98%)	22.1	975 (98.4%)	
Ulcer	95	Smoking status, n(%)	Yes	38 (2%)	22.1	16 (1.6%)	
Ulcer	95	Stroke, n(%)	No	1749 (84.6%)	15.4	856 (86.4%)	
Ulcer	95	Stroke, n(%)	Yes	319 (15.4%)	15.4	135 (13.6%)	
Ulcer	95	Systolic blood pressure, mean(SD)		138 (18.4)	6.6	137.4 (18.2)	
Ulcer	95	Time since last healthcare visit, mean(SD)		1.6 (2)	0	1.3 (1.7)	
Ulcer	95	Total cholesterol, mean(SD)		5 (1.2)	36.6	4.9 (1.2)	
Ulcer	96	Age at diagnosis of type 2 diabetes, mean(SD)		90.4 (3.1)	0	88.9 (2.7)	
Ulcer	96	BMI, mean(SD)		25.7 (4.1)	28.9	25.4 (3.8)	
Ulcer	96	HbA1c, mean(SD)		50.8 (11.4)	7.2	50.6 (11)	
Ulcer	96	HDL-cholesterol, mean(SD)		1.4 (0.4)	46.1	1.4 (0.4)	
Ulcer	96	Ischemic heart disease, n(%)	No	884 (64.2%)	14.9	389 (63.9%)	
Ulcer	96	Ischemic heart disease, n(%)	Yes	493 (35.8%)	14.9	220 (36.1%)	
Ulcer	96	Neuropathy, n(%)	No	1116 (69%)	0	285 (46.8%)	

Ulcer	96	Neuropathy, n(%)	Yes	502 (31%)	0	324 (53.2%)	
Ulcer	96	Self-reported physical activity, n(%)	Never	398 (38.8%)	36.7	210 (34.5%)	
Ulcer	96	Self-reported physical activity, n(%)	<1 times/week	148 (14.4%)	36.7	89 (14.6%)	
Ulcer	96	Self-reported physical activity, n(%)	regularly - 1-2 times/week	170 (16.6%)	36.7	103 (16.9%)	
Ulcer	96	Self-reported physical activity, n(%)	regularly - 3-5 times/week	138 (13.5%)	36.7	92 (15.1%)	
Ulcer	96	Self-reported physical activity, n(%)	Daily	171 (16.7%)	36.7	115 (18.9%)	
Ulcer	96	Sex, n(%)	Males	462 (28.6%)	0.2	188 (30.9%)	n=1618 vs n=609
Ulcer	96	Sex, n(%)	Females	1153 (71.4%)	0.2	421 (69.1%)	
Ulcer	96	Smoking status, n(%)	No	1183 (98.1%)	25.5	597 (98%)	
Ulcer	96	Smoking status, n(%)	Yes	23 (1.9%)	25.5	12 (2%)	
Ulcer	96	Stroke, n(%)	No	1151 (84.8%)	16.1	532 (87.4%)	
Ulcer	96	Stroke, n(%)	Yes	206 (15.2%)	16.1	77 (12.6%)	
Ulcer	96	Systolic blood pressure, mean(SD)		138.7 (18.6)	7.3	138 (18.4)	
Ulcer	96	Time since last healthcare visit, mean(SD)		1.7 (2.1)	0	1.3 (1.8)	
Ulcer	96	Total cholesterol, mean(SD)		5 (1.2)	39.2	5 (1.2)	
Ulcer	97	Age at diagnosis of type 2 diabetes, mean(SD)		91.4 (3.1)	0	89.8 (2.7)	
Ulcer	97	BMI, mean(SD)		25.8 (4)	31.3	25.4 (3.8)	
Ulcer	97	HbA1c, mean(SD)		50.5 (10.9)	7.2	50.8 (10.8)	
Ulcer	97	HDL-cholesterol, mean(SD)		1.4 (0.4)	49.4	1.4 (0.4)	
Ulcer	97	Ischemic heart disease, n(%)	No	555 (62.2%)	15	231 (61.6%)	
Ulcer	97	Ischemic heart disease, n(%)	Yes	338 (37.8%)	15	144 (38.4%)	
Ulcer	97	Neuropathy, n(%)	No	735 (69.9%)	0	174 (46.4%)	
Ulcer	97	Neuropathy, n(%)	Yes	316 (30.1%)	0	201 (53.6%)	

Ulcer	97	Self-reported physical activity, n(%)	Never	270 (40.8%)	37.1	138 (36.8%)	
Ulcer	97	Self-reported physical activity, n(%)	<1 times/week	98 (14.8%)	37.1	51 (13.6%)	
Ulcer	97	Self-reported physical activity, n(%)	regularly - 1-2 times/week	109 (16.5%)	37.1	68 (18.1%)	
Ulcer	97	Self-reported physical activity, n(%)	regularly - 3-5 times/week	84 (12.7%)	37.1	55 (14.7%)	
Ulcer	97	Self-reported physical activity, n(%)	Daily	100 (15.1%)	37.1	63 (16.8%)	
Ulcer	97	Sex, n(%)	Males	273 (26%)	0.2	112 (29.9%)	n=1051 vs n=375
Ulcer	97	Sex, n(%)	Females	776 (74%)	0.2	263 (70.1%)	
Ulcer	97	Smoking status, n(%)	No	759 (97.9%)	26.3	366 (97.6%)	
Ulcer	97	Smoking status, n(%)	Yes	16 (2.1%)	26.3	9 (2.4%)	
Ulcer	97	Stroke, n(%)	No	745 (84.6%)	16.2	323 (86.1%)	
Ulcer	97	Stroke, n(%)	Yes	136 (15.4%)	16.2	52 (13.9%)	
Ulcer	97	Systolic blood pressure, mean(SD)		138.4 (18.8)	6.3	137 (19.2)	
Ulcer	97	Time since last healthcare visit, mean(SD)		1.8 (2.1)	0	1.4 (1.7)	
Ulcer	97	Total cholesterol, mean(SD)		5.1 (1.1)	41.9	5 (1.1)	
Ulcer	98	Age at diagnosis of type 2 diabetes, mean(SD)		92.2 (3)	0	90.6 (2.8)	
Ulcer	98	BMI, mean(SD)		25.5 (3.9)	33.2	25.2 (3.7)	
Ulcer	98	HbA1c, mean(SD)		50.4 (11.5)	8.4	51.1 (11.1)	
Ulcer	98	HDL-cholesterol, mean(SD)		1.4 (0.4)	49.5	1.4 (0.5)	
Ulcer	98	Ischemic heart disease, n(%)	No	349 (62.5%)	14.3	141 (62.4%)	
Ulcer	98	Ischemic heart disease, n(%)	Yes	209 (37.5%)	14.3	85 (37.6%)	
Ulcer	98	Neuropathy, n(%)	No	469 (72%)	0	114 (50.4%)	
Ulcer	98	Neuropathy, n(%)	Yes	182 (28%)	0	112 (49.6%)	
Ulcer	98	Self-reported physical activity, n(%)	Never	164 (41.5%)	39.3	78 (34.5%)	

Ulcer	98	Self-reported physical activity, n(%)	<1 times/week	60 (15.2%)	39.3	36 (15.9%)	
Ulcer	98	Self-reported physical activity, n(%)	regularly - 1-2 times/week	69 (17.5%)	39.3	46 (20.4%)	
Ulcer	98	Self-reported physical activity, n(%)	regularly - 3-5 times/week	48 (12.2%)	39.3	28 (12.4%)	
Ulcer	98	Self-reported physical activity, n(%)	Daily	54 (13.7%)	39.3	38 (16.8%)	
Ulcer	98	Sex, n(%)	Males	153 (23.5%)	0.2	68 (30.1%)	n=651 vs n=226
Ulcer	98	Sex, n(%)	Females	497 (76.5%)	0.2	158 (69.9%)	
Ulcer	98	Smoking status, n(%)	No	463 (98.3%)	27.6	222 (98.2%)	
Ulcer	98	Smoking status, n(%)	Yes	8 (1.7%)	27.6	4 (1.8%)	
Ulcer	98	Stroke, n(%)	No	463 (84.3%)	15.7	189 (83.6%)	
Ulcer	98	Stroke, n(%)	Yes	86 (15.7%)	15.7	37 (16.4%)	
Ulcer	98	Systolic blood pressure, mean(SD)		137.9 (18.5)	6.8	136.1 (17.9)	
Ulcer	98	Time since last healthcare visit, mean(SD)		1.9 (2.2)	0	1.5 (1.9)	
Ulcer	98	Total cholesterol, mean(SD)		5.1 (1.2)	41.8	5 (1.2)	
Ulcer	99	Age at diagnosis of type 2 diabetes, mean(SD)		93.3 (3.1)	0	91.3 (2.8)	
Ulcer	99	BMI, mean(SD)		25.6 (4.1)	35.5	25.1 (4)	
Ulcer	99	HbA1c, mean(SD)		51.1 (12.8)	9.3	52.3 (12.9)	
Ulcer	99	HDL-cholesterol, mean(SD)		1.4 (0.4)	52	1.3 (0.4)	
Ulcer	99	Ischemic heart disease, n(%)	No	201 (62.6%)	14.9	73 (61.3%)	
Ulcer	99	Ischemic heart disease, n(%)	Yes	120 (37.4%)	14.9	46 (38.7%)	
Ulcer	99	Neuropathy, n(%)	No	280 (74.3%)	0	57 (47.9%)	
Ulcer	99	Neuropathy, n(%)	Yes	97 (25.7%)	0	62 (52.1%)	
Ulcer	99	Self-reported physical activity, n(%)	Never	104 (47.1%)	41.4	39 (32.8%)	
Ulcer	99	Self-reported physical activity, n(%)	<1 times/week	30 (13.6%)	41.4	22 (18.5%)	

Ulcer	99	Self-reported physical activity, n(%)	regularly - 1-2 times/week	34 (15.4%)	41.4	20 (16.8%)	
Ulcer	99	Self-reported physical activity, n(%)	regularly - 3-5 times/week	19 (8.6%)	41.4	13 (10.9%)	
Ulcer	99	Self-reported physical activity, n(%)	Daily	34 (15.4%)	41.4	25 (21%)	
Ulcer	99	Sex, n(%)	Males	88 (23.3%)	0	29 (24.4%)	n=377 vs n=119
Ulcer	99	Sex, n(%)	Females	289 (76.7%)	0	90 (75.6%)	
Ulcer	99	Smoking status, n(%)	No	264 (98.1%)	28.6	118 (99.2%)	
Ulcer	99	Smoking status, n(%)	Yes	5 (1.9%)	28.6	1 (0.8%)	
Ulcer	99	Stroke, n(%)	No	273 (85.6%)	15.4	103 (86.6%)	
Ulcer	99	Stroke, n(%)	Yes	46 (14.4%)	15.4	16 (13.4%)	
Ulcer	99	Systolic blood pressure, mean(SD)		138.6 (18)	7.4	138.1 (16.2)	
Ulcer	99	Time since last healthcare visit, mean(SD)		2 (2.3)	0	1.6 (2)	
Ulcer	99	Total cholesterol, mean(SD)		5.3 (1.2)	46.2	5.1 (1.2)	
Ulcer	100	Age at diagnosis of type 2 diabetes, mean(SD)		94.2 (3)	0	92 (2.5)	
Ulcer	100	BMI, mean(SD)		25.5 (4.1)	41.1	25.2 (4.1)	
Ulcer	100	HbA1c, mean(SD)		51.6 (12.9)	12.6	52.8 (13.8)	
Ulcer	100	HDL-cholesterol, mean(SD)		1.3 (0.4)	60.4	1.3 (0.3)	
Ulcer	100	Ischemic heart disease, n(%)	No	111 (63.8%)	15.9	33 (62.3%)	
Ulcer	100	Ischemic heart disease, n(%)	Yes	63 (36.2%)	15.9	20 (37.7%)	
Ulcer	100	Neuropathy, n(%)	No	152 (73.4%)	0	24 (45.3%)	
Ulcer	100	Neuropathy, n(%)	Yes	55 (26.6%)	0	29 (54.7%)	
Ulcer	100	Self-reported physical activity, n(%)	Never	55 (47.8%)	44.4	14 (26.4%)	
Ulcer	100	Self-reported physical activity, n(%)	<1 times/week	19 (16.5%)	44.4	14 (26.4%)	
Ulcer	100	Self-reported physical activity, n(%)	regularly - 1-2 times/week	16 (13.9%)	44.4	10 (18.9%)	

Ulcer	100	Self-reported physical activity, n(%)	regularly - 3-5 times/week	11 (9.6%)	44.4	8 (15.1%)	
Ulcer	100	Self-reported physical activity, n(%)	Daily	14 (12.2%)	44.4	7 (13.2%)	
Ulcer	100	Sex, n(%)	Males	45 (21.7%)	0	11 (20.8%)	n=207 vs n=53
Ulcer	100	Sex, n(%)	Females	162 (78.3%)	0	42 (79.2%)	
Ulcer	100	Smoking status, n(%)	No	140 (97.9%)	30.9	53 (100%)	
Ulcer	100	Smoking status, n(%)	Yes	3 (2.1%)	30.9	0 (0%)	
Ulcer	100	Stroke, n(%)	No	152 (88.9%)	17.4	50 (94.3%)	
Ulcer	100	Stroke, n(%)	Yes	19 (11.1%)	17.4	3 (5.7%)	
Ulcer	100	Systolic blood pressure, mean(SD)		137.5 (17.7)	8.7	136.8 (16)	
Ulcer	100	Time since last healthcare visit, mean(SD)		2.2 (2.4)	0	1.4 (1.9)	
Ulcer	100	Total cholesterol, mean(SD)		5.2 (1.1)	54.6	5 (1.1)	
