# Supplementary material for: Effect and prognostic value of physical activity for lower-extremity complications of type 2 diabetes across the adult lifespan: a national cohort study using Swedish registers
Source: BMJ Open. 2026 Nov 11;16(11):e114759. doi: 10.1136/bmjopen-2025-114759 (PMC13756448; doi:10.1136/bmjopen-2025-114759)
Supplement: Supplementary file 4 [file bmjopen-2025-114759.reviewer_comments.pdf]

PEER REVIEW HISTORY

BMJ Open publishes all reviews undertaken for accepted manuscripts. Reviewers are asked to complete a checklist review form and are provided with free text boxes to elaborate on their assessment. These free text comments are reproduced below.

ARTICLE DETAILS

Title (Provisional)

Effect and prognostic value of physical activity for lower-extremity complications of type 2 diabetes across the adult lifespan: a national cohort study using Swedish registers

Authors

Vogt, Thomas; Kennedy, Beatrice; Heiland, Emerald G; Byberg, Liisa; Michaëlsson, Karl; Brooke, Hannah

---

VERSION 1 - REVIEW

**Reviewer**	**1**
**Name**	**Kelson, Zoe**
**Affiliation**	**University of Exeter, Mathematics**
**Date**	**04-Feb-2026**
**COI**	**None**

This cohort study aimed to determine the effect of physical activity on the risk of later developing lower-extremity complications and its prognostic value for these complications across the adult lifespan of individuals with type 2 diabetes.

Reviewer comments:

"employed a landmarking approach with a 5-year follow-up and Fine-Gray subdistribution hazards models to investigate the relationships between physical activity and peripheral neuropathy/peripheral vascular disease, foot ulceration, and lower-extremity amputations throughout the adult lifespan, adjusting for standard clinical covariates. We compared the performance of multivariable models with and without physical activity using cross-validation." [Abstract]

The authors have succinctly described the analytical approach taken in the Abstract.

"Low physical activity was independently associated with a higher 5-year incidence of lower-extremity complications, across a wide range of ages. Between ages 47 and 78, never walking for 30 minutes (or equivalent) during a week was associated with a 1.17 to 1.52 times greater subdistribution hazard of peripheral neuropathy/peripheral vascular disease –

compared to daily walking. Age-specific estimates ranged from 1.36 to 3.37 for foot ulcers (ages 54 to 87) and from 1.65 to 3.93 for amputation (ages 69 to 85). " [Abstract]

Can statistical outcomes (e.g. p-values) and confidence intervals please be reported in the Abstract, and throughout the Results section?

"The multivariable model performance did not improve when physical activity was added to other clinical variables." [Abstract]

Can the authors please clarify this sentence in the Abstract?

"included all adults with newly diagnosed type 2 diabetes in Sweden (2007-2019) with ≥1 record in the National Diabetes Register (n=372,317)" [Abstract]

and

"We constructed a cohort based on all adults (≥ 18 years old) newly diagnosed with type 2 diabetes in Sweden in 2007-2019, with at least one record in the National Diabetes Register (NDR). The NDR contains information collected in primary and outpatient specialist care on individuals with any type of diabetes and has high coverage"

and

"Peripheral neuropathy/peripheral vascular disease [washout period 120 days]: n=345,133; foot ulceration [washout period 90 days]: n=365,398; amputation: n=371,779; major amputation: n=371,997)"

Can the authors please further specify the coverage and completeness of cohort?

"We determined the length of the washout period by graphically examining the overall incidence of all four outcomes following the diagnosis of type 2 diabetes."

Can the authors please conduct a sensitivity analysis using different washout periods?

"Information on self-reported physical activity was retrieved from the NDR, where it was recorded at each appointment in primary care and outpatient specialist care as the frequency of 30 minutes walking or equivalent during a week: (i) never, (ii) <1 times/week, (iii) regularly 1-2 times/week, (iv) regularly 3-5 times/week, and (v) daily."

Can the authors please specifically acknowledge the potential for (and impact of) self-reporting bias?

"From the NDR, we retrieved or derived the following known risk factors to be considered in statistical analyses: sex, age at diagnosis of type 2 diabetes, BMI, HbA1c, smoking status, systolic blood pressure, ratio of total to high-density lipoprotein cholesterol, past cardiovascular event(s), time since last healthcare visit, and existing peripheral neuropathy/peripheral vascular disease (described further in Supplement: Variables section). "

Can the authors please consider also including other variables, such as alcohol consumption and nutritional intake?

"We planned subgroup analyses by sex (male/female) and educational attainment."

and

"we repeated the model fitting steps described above separately for males and females, as well as for individuals with low and high education levels. We calculated the difference in log-sHR and their associated 95% CI (26) between males and females, and between those with low and high education levels."

Insightful subgroup analyses have been undertaken by the authors.

"A given individual could therefore be included in several landmark dataset if those conditions were met for several ages. We used the most recent value of the variables described above at each landmark age. "

Can the authors please clarify how repeated entries at different landmark ages (i.e. clustering by individual) was handled when interpreting the analyses?

"The proportion of missing values was between 0 and 20% in 88% of variables across all outcome-landmark age combinations (Supplement: Table 1.3). We applied multiple imputation by chained equations (22) to handle missing data as described in Supplement"

The authors have suitably handled missing data in the analysis.

Can they please assess whether missing data can be considered to be missing at random?

Can a sensitivity analysis on the approach taken for missing data please be performed, to help demonstrate the robustness of the study outcomes?

"we fitted three survival models for each outcome-landmark age combination. Model 1 only included self-reported physical activity. Model 2 included all prognostic factors described above except physical activity: sex, age at diagnosis of type 2 diabetes, BMI, HbA1c, smoking status, systolic blood pressure, ratio of total to high-density lipoprotein cholesterol, past cardiovascular event(s), and time since last healthcare visit. If the outcome was foot ulceration or amputation, we also included peripheral neuropathy/peripheral vascular disease. Model 3 incorporated physical activity and the variables from model 2. We calculated subdistribution hazard ratios (sHR) and their 95% confidence intervals (CI) using Fine-Gray subdistribution hazards models (24), which account for the competing risk of death and are better suited for prognosis-related research questions (25). "

and

"for the prediction of complications, we calculated time-dependent Brier scores (27) and time-dependent area under the receiver operating characteristic curves (AUC) (28) for model 2 and model 3, at five years of follow-up"

Can the authors please clarify the survival modelling approach taken in this study (e.g. have they applied a Cox regression analysis)?

Can the authors please also clarify the description of model 2, which is not clear whether 'sex, age at diagnosis of type 2 diabetes, BMI, HbA1c, smoking status, systolic blood pressure, ratio of total to high-density lipoprotein cholesterol, past cardiovascular event(s), and time since last healthcare visit' are included covariates or not?

Rather than a landmarking approach which segments data into multiple snapshots (potentially losing information and increasing computational effort), can the authors please consider including repeated measures in the analysis and applying a time-dependent Cox model, or adjusting for clustering by individual in a Cox regression model for example?

"we applied bootstrap cross-validation (500 repetitions) and repeatedly fitted models on training data while assessing model performance on validation data. In each of the 500 repetitions, the training data was obtained by sampling with replacement from the landmark dataset. The validation data consisted of all individuals not included in the training data. "

The authors do well to apply cross-validation, which helps to reduce the risk of over-fitting.

" To limit the computational burden, we calculated the Brier scores and AUCs only on a randomly chosen imputed dataset for each outcome-landmark age combination."

Can the authors please compute model performance metrics on all datasets, generating and reporting confidence intervals to satisfactorily communicate uncertainty?

"Finally, we did not find evidence of improvements in model performance that resulted from considering physical activity in addition to the other prognostic factors, and we did not see differences by landmark age (Figure 3, Supplement: Figures S7-S10, Table 4). For example, at landmark age 70, the change in Brier Score comparing model 3 to model 2 was -1.54e-4 (95% CI: -4.13e-4 - 1.71e-4) for peripheral neuropathy/peripheral vascular disease. More details about predictive performance for model 2 and model 3 separately can be found in Supplement (Figures S7-S10 and Table 4). "

Can the authors please report the more rigorous multivariate analyses as the primary outcomes of the study throughout the article?

Can the authors please clearly label all axis in the Figures?

"Nonetheless, some limitations apply. We could not include individuals with diagnosed type 2 diabetes who were not part of the NDR. Still, the NDR's coverage is estimated to be high, with 87% of all individuals diagnosed with diabetes in Sweden in 2019 (15), which limits the impact of this potential limitation. In addition, our measure of self-reported physical activity does not consider its intensity, duration, or type. Whether self-reported or not, different physical activity measures might have different prognostic relevance. Furthermore, foot ulcers and peripheral neuropathy/peripheral vascular disease are likely subject to misclassification. Cases of foot ulceration captured in inpatient and outpatient hospital-

based specialist care are only a subset of all incident foot ulcers among individuals with type 2 diabetes. Similar issues apply to peripheral neuropathy/peripheral vascular disease, as a clinical assessment of the feet determines its presence/absence, and therefore depends on individuals attending a visit with foot examination. Still, most patients have a foot examination each year — between 79.8% and 80.5% in 2019 (36). This limits the number of cases of peripheral neuropathy/peripheral vascular disease that could be missed and, therefore, the misclassification of actual cases as non-cases. Nevertheless, none of these issues are of concern for amputations as these procedures are always conducted in hospitals and recorded in the Patient Register. Noting that the results lend themselves to a similar interpretation across outcomes led us to believe that any misclassification of peripheral neuropathy/peripheral vascular disease or foot ulcers would not affect the overall interpretation of the study results"

The authors have provided a Discussion on the study limitations.

Thanks for providing a copy of the STROBE checklist.

Can the authors please confirm they adhered to STROBE reporting guidelines in the article?

Reviewer **2**

Name **Nagafuchi, Seiho**

Affiliation **Saga University Faculty of Medicine Graduate School of Medical Sciences, Internal Medicine**

Date **04-Jul-2026**

COI **None**

The manuscript was presented in a large scale patients with diabetes in Sweden, focusing on the outcome of diabetic complications, and acceptalbe content.

I would like to ask you to present the total number of patients studied in the materials and methods section as well as introduction, since the manuscript presented same patients number in amputation as amputation: n=371,779; major amputation: n=371,997:same number. The numbers should be reassessed and should correct them as such.

Reviewer **3**

Name **Looker, Helen**

Affiliation **National Institute of Diabetes and Digestive and Kidney Diseases Phoenix Epidemiology and Clinical Research Branch, CKDS**

Date **11-Aug-2026**

COI **None**

This manuscript details a study using Swedish data examining the role of self-reported physical activity on peripheral neuropathy in people with type 2 diabetes. They found that while self-report physical activity is associated with neuropathy and related outcomes, it was not significantly associated after inclusion in models with recognized risk factors entered as covariates. This manuscript takes full advantage of the wonderful data sources available in Sweden and are able to carry out models across the full spectrum of participant ages.

Questions/Comments

1. Please include some information on how diabetes type was determined.

2. You use the cut point of 30 mins or more exercise for your analysis. Is this a standard cut point used clinically? It would be helpful to provide more information on what was recorded in the clinical notes. Did you consider any other cut-points?

3. It would be helpful to define 'major amputation' – clearly it can be worked out by looking up the codes in the supplementary table but I think a brief sentence to explain your definition would help the general reader.

4. You found age limits to your associations between outcomes and exercise. I'd be interested to know how much that was driven by low outcome numbers (at the younger ages) or low numbers managing the 30 minutes of walking (at the higher ages as detailed in the results). I think this should be commented on when discussing the findings relating to the age limits for associations reported.

5. You report that activity didn't improve models including traditional risk factors and include an interesting discussion of why that may be. I would suggest considering the role of risk factor measurement accuracy to that discussion. Most of the risk factors you list are things we can measure directly rather than dependent on self report. As a result I would expect the categorization of physical activity to be far less accurate than those other measures which may influence its usefulness in a model. It remains plausible that a more accurate assessment of exercise would be informative in the multivariate models were it available.

6. I am still unclear how you determined the washout times – can you clarify what you were looking at when examining the incidence rates over time. Please make explicit that there was no washout time for either levels of amputation.

VERSION 1 - AUTHOR RESPONSE

Reviewer: 1
Prof. Zoe Kelson, University of Exeter
Comments to the Author:
This cohort study aimed to determine the effect of physical activity on the risk of later developing lower-extremity complications and its prognostic value for these complications across the adult lifespan of individuals with type 2 diabetes.

Reviewer comments:

"employed a landmarking approach with a 5-year follow-up and Fine-Gray subdistribution hazards models to investigate the relationships between physical activity and peripheral neuropathy/peripheral vascular disease, foot ulceration, and lower-extremity amputations throughout the adult lifespan, adjusting for standard clinical covariates. We compared the performance of multivariable models with and without physical activity using cross-validation." [Abstract]
The authors have succinctly described the analytical approach taken in the Abstract.

"Low physical activity was independently associated with a higher 5-year incidence of lower-extremity complications, across a wide range of ages. Between ages 47 and 78, never walking for 30 minutes (or equivalent) during a week was associated with a 1.17 to 1.52 times greater subdistribution hazard of peripheral neuropathy/peripheral vascular disease – compared to daily walking. Age-specific estimates ranged from 1.36 to 3.37 for foot ulcers (ages 54 to 87) and from 1.65 to 3.93 for amputation (ages 69 to 85). " [Abstract]
Can statistical outcomes (e.g. p-values) and confidence intervals please be reported in the Abstract, and throughout the Results section?

> We included 95% confidence intervals in the results section in the Abstract, and in the Results section in the main text. We believe that confidence intervals are more informative than reporting p-values.

"The multivariable model performance did not improve when physical activity was added to other clinical variables." [Abstract]
Can the authors please clarify this sentence in the Abstract?

> We expanded this sentence so that it reads: "When comparing the performance of multivariable models with and without physical activity using cross-validation, the addition of physical activity to other clinical variables did not improve model performance".

"included all adults with newly diagnosed type 2 diabetes in Sweden (2007-2019) with ≥1 record in the National Diabetes Register (n=372,317)" [Abstract]
and
"We constructed a cohort based on all adults (≥ 18 years old) newly diagnosed with type 2 diabetes in Sweden in 2007-2019, with at least one record in the National Diabetes Register (NDR). The NDR contains information collected in primary and outpatient specialist care on individuals with any type of diabetes and has high coverage"
and
"Peripheral neuropathy/peripheral vascular disease [washout period 120 days]: n=345,133; foot ulceration [washout period 90 days]: n=365,398; amputation: n=371,779; major amputation: n=371,997)"
Can the authors please further specify the coverage and completeness of cohort?

> We modified the main text, and added additional references, such that the text now reads: "The NDR contains information collected in primary and outpatient specialist care on individuals with any type of diabetes and has attained a high coverage (15). Coverage increased steadily from 50% in 2007 to 90% in 2011-2012 (16-21), with similar coverage for different diabetes types at least since the early 2010's (19-22)."

"We determined the length of the washout period by graphically examining the overall incidence of all four outcomes following the diagnosis of type 2 diabetes."
Can the authors please conduct a sensitivity analysis using different washout periods?

In the supplementary material, we have now included plots that display the incidence of peripheral neuropathy/peripheral vascular disease, foot ulcers, and amputations over the study follow-up (indexed at 0 years of follow-up, at the time of diagnosis of type 2 diabetes). These plots are Figures S2b, S2c, and S2d.

In these plots, the number of incident cases decreases over time. This is expected for two reasons. First, as more individuals experience an outcome, the population at risk became smaller. Second, and more importantly, because individuals were included if they were newly diagnosed with type 2 diabetes between 2007 and 2019, but the date of administrative censoring was set to 31st of December 2019 for everyone. Therefore, individuals with a diagnosis of type 2 diabetes in later years had a shorter follow-up time.

To decide on the washout period, we discussed the problem of identifying incident vs prevalent cases with clinicians. They warned us that cases of peripheral neuropathy/peripheral vascular disease and foot ulcers (but especially cases of peripheral neuropathy/peripheral vascular disease) in the months after a diagnosis of type 2 diabetes were likely to be prevalent cases: they were already present before the diagnosis of type 2 diabetes, but not identified by a nurse or a physician, and therefore not recorded in any register. What Figures S2b, S2c, and S2d show is that there is a spike in cases at the start of follow-up for peripheral neuropathy/peripheral vascular disease and foot ulcers. This spike does not follow the same pattern of a gradual decline in incident cases that is visible in the plots (explained in the paragraph above this one). There is no such spike for amputations, and this outcome follows the general pattern of a gradual decline in incident cases along the entire study period. In addition, unlike for peripheral neuropathy/peripheral vascular disease and foot ulcers, we know the exact date for when an amputation is performed, as it is a surgical procedure recorded in the Patient register – we can know directly whether it is incident or not. It does not rely on a diagnosis test or some sort of examination of the feet, which may capture peripheral neuropathy/peripheral vascular disease and foot ulcers, but not necessarily at the time of their appearance – i.e., they might have been present for days, weeks, or even months (especially in the case of peripheral neuropathy/peripheral vascular disease).

Including a washout period for peripheral neuropathy/peripheral vascular disease and foot ulcers also has clinical relevance for the question related to the prognostic value of physical activity: if incident-looking cases shortly after a diagnosis of type 2 diabetes are in large part prevalent cases, trying to predict their future occurrence will not help preventing them, as they have already occurred. Excluding what are likely to be prevalent cases helps avoiding this problem.

Finally, we included several lower-extremity complications in this study to be able to balance different sources of bias across outcomes, one of them being that "foot ulcers and peripheral neuropathy/peripheral vascular disease are likely subject to misclassification" (see Discussion→ Strengths and limitations→second paragraph). We note that the fact that results are similar for different outcomes, including amputations for which we can clearly identify incident cases, is a reassurance against

misclassification leading to contradictory overall conclusions (including misclassification of incident vs prevalent cases at the start of follow-up).

Based on figure S2b, S2c, we believe we have chosen appropriate wash-out periods and we do not think that sensitivity analyses with different washout periods would change the overall conclusions of the study.

"Information on self-reported physical activity was retrieved from the NDR, where it was recorded at each appointment in primary care and outpatient specialist care as the frequency of 30 minutes walking or equivalent during a week: (i) never, (ii) <1 times/week, (iii) regularly 1-2 times/week, (iv) regularly 3-5 times/week, and (v) daily."
Can the authors please specifically acknowledge the potential for (and impact of) self-reporting bias?

In the "Strengths and limitations" subsection of the Discussion, we added the following: "Physical activity was self-reported, and can therefore suffer from self-reporting bias. As a socially desirable practice, it may be prone to overreporting".

"From the NDR, we retrieved or derived the following known risk factors to be considered in statistical analyses: sex, age at diagnosis of type 2 diabetes, BMI, HbA1c, smoking status, systolic blood pressure, ratio of total to high-density lipoprotein cholesterol, past cardiovascular event(s), time since last healthcare visit, and existing peripheral neuropathy/peripheral vascular disease (described further in Supplement: Variables section)."
Can the authors please consider also including other variables, such as alcohol consumption and nutritional intake?

Alcohol consumption and nutritional intake are not variables that are collected in the National Diabetes Register, or in other Swedish national registers. We added the following in the "Strengths and limitations" subsection of the Discussion: "When determining the effect of physical activity on the risk of later developing lower-extremity complications, we could not adjust for variables like alcohol consumption, nutritional factors, or genetic factors, since these data are not recorded in Swedish national registers, and some residual confounding is therefore possible".

"We planned subgroup analyses by sex (male/female) and educational attainment."
and
"we repeated the model fitting steps described above separately for males and females, as well as for individuals with low and high education levels. We calculated the difference in log-sHR and their associated 95% CI (26) between males and females, and between those with low and high education levels."
Insightful subgroup analyses have been undertaken by the authors.

"A given individual could therefore be included in several landmark dataset if those conditions were met for several ages. We used the most recent value of the variables described above at each landmark age. "
Can the authors please clarify how repeated entries at different landmark ages (i.e. clustering by individual) was handled when interpreting the analyses?

Any given individual could be present at most once in a landmark dataset. Each landmark dataset was analysed separately, so that there was no clustering by individual in any statistical analysis. We modified the main text in Methods→Statistical analysis→Landmarking to clarify this: “Individuals could be included at most once in any given landmark dataset, and each landmark dataset was analysed separately (there was no clustering by individual in any analysis)”.

"The proportion of missing values was between 0 and 20% in 88% of variables across all outcome-landmark age combinations (Supplement: Table 1.3). We applied multiple imputation by chained equations (22) to handle missing data as described in Supplement"
The authors have suitably handled missing data in the analysis.
Can they please assess whether missing data can be considered to be missing at random?
Can a sensitivity analysis on the approach taken for missing data please be performed, to help demonstrate the robustness of the study outcomes?

To our knowledge it is not possible to prove that data are missing at random, as this would require showing that data are not missing not at random. If data are missing not at random, then by definition the information explaining the missingness is missing (p37 of (1)). For example, if the probability for physical activity to be missing depends on the level of physical activity itself. However, we followed the advice from others, who suggest that the inclusion of more predictors in multiple imputation makes the missing at random assumption more plausible (p67 of (1)). We therefore included additional variables in multiple imputation, that were not used in the analysis later, but only for the purpose of imputing data and making the missing at random assumption more credible. These additional variables are described in the “Multiple Imputation” section of the supplementary material.

To help demonstrate the robustness of the study results to the choice of missing data methods, we additionally conducted complete case analyses. The results were similar and lent themselves to the same conclusions as results using multiply imputed data. We did not include these results as they did not change substantive conclusions, and burdened the manuscript and supplement by doubling the number of figures and tables.

"we fitted three survival models for each outcome-landmark age combination. Model 1 only included self-reported physical activity. Model 2 included all prognostic factors described above except physical activity: sex, age at diagnosis of type 2 diabetes, BMI, HbA1c, smoking status, systolic blood pressure, ratio of total to high-density lipoprotein cholesterol, past cardiovascular event(s), and time since last healthcare visit. If the outcome was foot ulceration or amputation, we also included peripheral neuropathy/peripheral vascular disease. Model 3 incorporated physical activity and the variables from model 2. We calculated subdistribution hazard ratios (sHR) and their 95% confidence intervals (CI) using Fine-Gray subdistribution hazards models (24), which account for the competing risk of death and are better suited for prognosis-related research questions (25). "
and
"for the prediction of complications, we calculated time-dependent Brier scores (27) and time-dependent area under the receiver operating characteristic curves (AUC) (28) for model 2 and model 3, at five years of follow-up"
Can the authors please clarify the survival modelling approach taken in this study (e.g. have they applied a Cox regression analysis)?

As described in the text quoted, we used Fine-Gray subdistribution hazards models. We understand that the mention of "survival models" at the top of the subsection "Statistical modelling: survival analysis and prediction" might lead to a lack of clarity, and we therefore added a mention in parenthesis such that it now reads: "First, we fitted three survival (Fine-Gray subdistribution hazards) models for each outcome-landmark age combination".

Can the authors please also clarify the description of model 2, which is not clear whether 'sex, age at diagnosis of type 2 diabetes, BMI, HbA1c, smoking status, systolic blood pressure, ratio of total to high-density lipoprotein cholesterol, past cardiovascular event(s), and time since last healthcare visit' are included covariates or not?

In model 2, these covariates were indeed included (only physical activity was not included). The text now reads: "Model 2 included all prognostic factors described above except physical activity. It therefore included sex, age at diagnosis of type 2 diabetes, BMI, HbA1c, smoking status, systolic blood pressure, ratio of total to high-density lipoprotein cholesterol, past cardiovascular event(s), and time since last healthcare visit".

Rather than a landmarking approach which segments data into multiple snapshots (potentially losing information and increasing computational effort), can the authors please consider including repeated measures in the analysis and applying a time-dependent Cox model, or adjusting for clustering by individual in a Cox regression model for example?

The use of a landmarking approach was motivated by several considerations. To begin with, as stated in the Introduction, it is unclear how physical activity impacts the risk of complications at different ages. Therefore, using a landmarking approach enabled us to assess whether self-reported physical activity was associated with lower-extremity complications at all ages where a sufficient number of outcomes existed.

Furthermore, for our aim to assess the prognostic value of physical activity, using a landmarking approach has several advantages. First, it clarifies at what time the model performance is evaluated (here, at 5 years of follow-up), and we can correspondingly use measures of performance appropriate for evaluating performance at a specific time horizon in time-to-event settings (e.g., by using the time-dependent Brier score (2)). Second, it also clarifies what information can be used at each landmark age for the purpose of comparing the prognostic value of a model with, and a model without, physical activity: we can only include information available at the landmark age, which is why we used the most recently available information on covariates at a given landmark age, and not information on covariates after that landmark age (no time-varying covariates after landmark age). This is an attempt to mimic a realistic healthcare setting, where at any given point in time, no prediction of the future risk of lower-extremity complications can be made with information that does not exist already (i.e., no future information on time-varying covariates such as HbA1c). The covariates included in the statistical models were therefore the latest information available to a nurse or a physician at the time where prediction could be made about the, e.g., 5-year risk of lower-extremity complications.

In this study, we used Fine-Gray subdistribution hazards models and not Cox models because Fine-Gray subdistribution hazards models account for the competing risk of

death in a way that are better suited for prognosis-related research questions (3). This is because if a covariate is associated with an increase in the subdistribution hazard of an event, it will also be associated with an increase in the absolute risk of the event (4). This is not necessarily the case for Cox models if competing events (death) are present. Because the absolute risk of an event of interest at later times is the most relevant quantity for assessing the prognostic value of a variable or of a model, we favoured Fine-Gray subdistribution hazards models over Cox models. So for the reasons mentioned, we do not think that time-dependent Cox models or clustered Cox models would help us address the aims of our study.

"we applied bootstrap cross-validation (500 repetitions) and repeatedly fitted models on training data while assessing model performance on validation data. In each of the 500 repetitions, the training data was obtained by sampling with replacement from the landmark dataset. The validation data consisted of all individuals not included in the training data. "
The authors do well to apply cross-validation, which helps to reduce the risk of over-fitting.

" To limit the computational burden, we calculated the Brier scores and AUCs only on a randomly chosen imputed dataset for each outcome-landmark age combination."
Can the authors please compute model performance metrics on all datasets, generating and reporting confidence intervals to satisfactorily communicate uncertainty?

We did not find that adding physical activity to clinical indicators routinely assessed in clinical practice would improve the predictive performance of statistical models. Computing model performance metrics on all imputed datasets, by communicating the greater uncertainty that comes with imputation, would not change the substantive conclusions of the article, as this would just mean greater confidence intervals for results that are already not suggesting that physical activity has added prognostic value. Reanalysing all the imputed datasets to calculate performance measures would require substantial computing time. Based on previous analyses conducted on a randomly chosen imputed dataset for each outcome-landmark age combination, and based on the fact that multiple imputation generated 20 imputed datasets for each outcome-landmark age combination, we estimate that these additional analyses would require roughly: (i) 2 months for peripheral neuropathy/peripheral vascular disease, running the analyses in parallel on 16 cores, (ii) 3 weeks for foot ulcers, running the analyses in parallel on 12 cores, (iii) and 1 month for amputations, running the analyses in parallel on 5 cores. We do not deem these additional analyses to be justifiable given the above reasoning that the conclusions will remain the same.

"Finally, we did not find evidence of improvements in model performance that resulted from considering physical activity in addition to the other prognostic factors, and we did not see differences by landmark age (Figure 3, Supplement: Figures S7-S10, Table 4). For example, at landmark age 70, the change in Brier Score comparing model 3 to model 2 was -1.54e-4 (95% CI: -4.13e-4 - 1.71e-4) for peripheral neuropathy/peripheral vascular disease. More details about predictive performance for model 2 and model 3 separately can be found in Supplement (Figures S7-S10 and Table 4). "
Can the authors please report the more rigorous multivariate analyses as the primary outcomes of the study throughout the article?

We are not sure we understand this comment, and what the “more rigorous multivariate analyses” are. The second paragraph of the Results section reports

findings on the association of physical activity with the incidence of lower-extremity complications of type 2 diabetes, from multivariable Fine-Gray models adjusted for what we call there "clinically relevant variables". In case this was not clear, we added ", i.e., model 3" after "in models adjusted for clinically relevant variables".

What is quoted above comes from the last paragraph from the Results section, where we reported whether the addition of physical activity to a Fine-Gray model already containing prognostic factors such as age, sex, HbA1c, and BMI, improved the performance of the Fine-Gray model – whether it improved the Brier score and AUC. To assess the prognostic value of physical activity – to assess whether it "can be used to make more reliable predictions about future risk of medical conditions" (5) – we had to compare the performance of model 2 (containing clinical variables but not physical activity) and model 3 (containing clinical variables AND physical activity). An improvement in predictive performance (Brier score and/or AUC) would indicate that physical activity has added prognostic value. No improvement would indicate that it doesn't have prognostic value. Therefore, the important results with respect to this specific research question is the comparison of predictive performance between model 2 and model 3, not the predictive performance of model 3 in isolation.

The methods used to assess the prognostic value of physical activity are detailed at the end of the Methods section (4th paragraph below "Statistical modelling: survival analysis and prediction", starting with "Third …"), the related findings are summarised at the end of the Results section, and we discuss these findings in the 4th and 5th paragraphs of the Discussion, just before the "Strengths and limitations" subsection.

Please let us know if this addresses your comments, or if we misunderstood them.

Can the authors please clearly label all axis in the Figures?

To allow the panels within the figures showing the data to be as large (and therefore readable) as possible, we chose to use a common x-axis label and common y-axis label across the panels. This helped to avoid too much text on the figures and maintain a 'clean' and consistent appearance. We acknowledge that there can be differences in stylist opinions. Nonetheless, the meaning of each Figure is expanded upon in the Figure legends, and if an abbreviation is used, it is included in the Abbreviations section. For example, "sHR" is used as a label for the y-axis of Figure 2. The Figure legend states: "Association between level of self-reported physical activity and the ***subdistribution hazard*** of […]" (emphasis added). It also indicates that "The y-axis is on a base-2 logarithmic scale". The Abbreviations section states: "sHR: subdistribution hazard ratio", and we have now added this in Figure legends as well.

"Nonetheless, some limitations apply. We could not include individuals with diagnosed type 2 diabetes who were not part of the NDR. Still, the NDR's coverage is estimated to be high, with 87% of all individuals diagnosed with diabetes in Sweden in 2019 (15), which limits the impact of this potential limitation. In addition, our measure of self-reported physical activity does not consider its intensity, duration, or type. Whether self-reported or not, different physical activity measures might have different prognostic relevance. Furthermore, foot ulcers and peripheral neuropathy/peripheral vascular disease are likely subject to misclassification. Cases of foot ulceration captured in inpatient and outpatient hospital-based specialist care are only a subset of all incident foot ulcers among individuals with type 2

diabetes. Similar issues apply to peripheral neuropathy/peripheral vascular disease, as a clinical assessment of the feet determines its presence/absence, and therefore depends on individuals attending a visit with foot examination. Still, most patients have a foot examination each year — between 79.8% and 80.5% in 2019 (36). This limits the number of cases of peripheral neuropathy/peripheral vascular disease that could be missed and, therefore, the misclassification of actual cases as non-cases. Nevertheless, none of these issues are of concern for amputations as these procedures are always conducted in hospitals and recorded in the Patient Register. Noting that the results lend themselves to a similar interpretation across outcomes led us to believe that any misclassification of peripheral neuropathy/peripheral vascular disease or foot ulcers would not affect the overall interpretation of the study results"
The authors have provided a Discussion on the study limitations.

Thanks for providing a copy of the STROBE checklist.
Can the authors please confirm they adhered to STROBE reporting guidelines in the article?

> We added "We adhered to the STROBE reporting guidelines" at the end of the Methods section, just above the "Patient and Public Involvement statement" subsection.

Reviewer: 2
Dr. Seiho Nagafuchi, Saga University Faculty of Medicine Graduate School of Medical Sciences, Fukuoka Heartnet Hospital
Comments to the Author:
The manuscript was presented in a large scale patients with diabetes in Sweden, focusing on the outcome of diabetic complications, and acceptalbe content.
I would like to ask you to present the total number of patients studied in the materials and methods section as well as introduction, since the manuscript presented same patients number in amputation as amputation: n=371,779; major amputation: n=371,997:same number. The numbers should be reassessed and should correct them as such.

> The total numbers are indicated at the end of the "Study population" subsection in the Methods, where the numbers mentioned above (amputation: n=371,779; major amputation: n=371,997) come from. Figure S1, in the supplementary material, contains more details on how the study populations were derived. The two numbers mentioned above (amputation: n=371,779; major amputation: n=371,997) are not the same number. The study population free of lower-extremity major amputation at baseline is larger than the study population free of any lower-extremity amputation at baseline, by 218 individuals (371997 - 371779 = 218).

Reviewer: 3
Dr. Helen Looker, National Institute of Diabetes and Digestive and Kidney Diseases Phoenix Epidemiology and Clinical Research Branch
Comments to the Author:
This manuscript details a study using Swedish data examining the role of self-reported physical activity on peripheral neuropathy in people with type 2 diabetes. They found that while self-report physical activity is associated with neuropathy and related outcomes, it was not significantly associated after inclusion in models with recognized risk factors entered as covariates. This manuscript takes full advantage of the wonderful data sources available in Sweden and are able to carry out models across the full spectrum of participant ages.

Questions/Comments

1. Please include some information on how diabetes type was determined.

> We have clarified this in the first paragraph of the Methods section, which now reads: "We identified those with diagnosed type 2 diabetes based on clinical assessment as it was recorded by clinicians in the variable for 'diabetes type' in the NDR" .

2. You use the cut point of 30 mins or more exercise for your analysis. Is this a standard cut point used clinically? It would be helpful to provide more information on what was recorded in the clinical notes. Did you consider any other cut-points?

> Physical activity is recorded in the NDR as a five-level categorical variable reflecting frequency of 30 minutes walking or equivalent during a week reported by clinicians as: (i) never, (ii) <1 times/week, (iii) regularly 1-2 times/week, (iv) regularly 3-5 times/week, and (v) daily. Since the definition is pre-determined based on the way the data recorded in the NDR, there is no additional cut-points that we could consider. We describe this in the Methods section under "Self-reported physical activity and other prognostic factors". The main text reads: "Information on self-reported physical activity was retrieved from the NDR, where it was recorded at each appointment in primary care and outpatient specialist care as the frequency of 30 minutes walking or equivalent during a week: (i) never, (ii) <1 times/week, (iii) regularly 1-2 times/week, (iv) regularly 3-5 times/week, and (v) daily.".

3. It would be helpful to define 'major amputation' – clearly it can be worked out by looking up the codes in the supplementary table but I think a brief sentence to explain your definition would help the general reader.

> In the definition we used, major amputations include amputations performed above the ankle. We added to the main text, in the "Outcomes" subsection of the Methods: "Major amputations were amputations performed above the ankle and could include, for example, transtibial amputations and transpelvic amputations".

4. You found age limits to your associations between outcomes and exercise. I'd be interested to know how much that was driven by low outcome numbers (at the younger ages) or low numbers managing the 30 minutes of walking (at the higher ages as detailed in the results). I think this should be commented on when discussing the findings relating to the age limits for associations reported.

> The low number of events at younger ages might indeed limit our interpretation of the results. We added the following in the third paragraph of the Discussion: "The small number of events at younger ages also did not allow the precise estimation of measures of association".
>
> At older ages however, the competing risk of death might explain the lack of (or inverse) association of physical activity with peripheral neuropathy/peripheral vascular disease (Figure 2, panel A, > 80 years of age). As the risk of death is higher in this subgroup, it competes more strongly with the risk of peripheral neuropathy/peripheral vascular disease. This is because physical activity is associated with a reduced risk of all-cause mortality (e.g., see (6)), not only with peripheral neuropathy/peripheral vascular disease. This is why we noted, also in the third

paragraph of the Discussion, that "in individuals older than 80, physical activity might still delay mortality (9) even if it might not affect the risk of lower-extremity complications".

5. You report that activity didn't improve models including traditional risk factors and include an interesting discussion of why that may be. I would suggest considering the role of risk factor measurement accuracy to that discussion. Most of the risk factors you list are things we can measure directly rather than dependent on self report. As a result I would expect the categorization of physical activity to be far less accurate than those other measures which may influence its usefulness in a model. It remains plausible that a more accurate assessment of exercise would be informative in the multivariate models were it available.

> We added this suggestion to the following sentence in the 2nd paragraph of the "Strengths and limitations" subsection in the Discussion: "Whether self-reported or not, different physical activity measures, for example obtained through accelerometery-based technology, might provide additional prognostic value. ".

6. I am still unclear how you determined the washout times – can you clarify what you were looking at when examining the incidence rates over time. Please make explicit that there was no washout time for either levels of amputation.

> In the supplementary material, we have now included plots that display the incidence of peripheral neuropathy/peripheral vascular disease, foot ulcers, and amputations over the study follow-up (indexed at 0 years of follow-up, at the time of diagnosis of type 2 diabetes). These plots are Figures S2b, S2c, and S2d.
>
> In these plots, the number of incident cases decreases over time. This is expected for two reasons. First, as more individuals experience an outcome, the population at risk became smaller. Second, and more importantly, because individuals were included if they were newly diagnosed with type 2 diabetes between 2007 and 2019, but the date of administrative censoring was set to 31st of December 2019 for everyone. Therefore, individuals with a diagnosis of type 2 diabetes in later years had a shorter follow-up time.
>
> To decide on the washout period, we discussed the problem of identifying incident vs prevalent cases with clinicians. They warned us that cases of peripheral neuropathy/peripheral vascular disease and foot ulcers (but especially cases of peripheral neuropathy/peripheral vascular disease) in the months after a diagnosis of type 2 diabetes were likely to be prevalent cases: they were already present before the diagnosis of type 2 diabetes, but not identified by a nurse or a physician, and therefore not recorded in any register. What Figures S2b, S2c, and S2d show is that there is a spike in cases at the start of follow-up for peripheral neuropathy/peripheral vascular disease and foot ulcers. This spike does not follow the same pattern of a gradual decline in incident cases that is visible in the plots (explained in the paragraph above this one). There is no such spike for amputations, and this outcome follows the general pattern of a gradual decline in incident cases along the entire study period. In addition, unlike for peripheral neuropathy/peripheral vascular disease and foot ulcers, we know the exact date for when an amputation is performed, as it is a surgical procedure recorded in the Patient register – we can know directly whether it is incident or not. It does not rely on a diagnosis test or some sort of examination of the feet, which may capture peripheral neuropathy/peripheral vascular disease and foot

ulcers, but not necessarily at the time of their appearance – i.e., they might have been present for days, weeks, or even months (especially in the case of peripheral neuropathy/peripheral vascular disease).

Including a washout period for peripheral neuropathy/peripheral vascular disease and foot ulcers also has clinical relevance for the question related to the prognostic value of physical activity: if incident-looking cases shortly after a diagnosis of type 2 diabetes are in large part prevalent cases, trying to predict their future occurrence will not help preventing them, as they have already occurred. Excluding what are likely to be prevalent cases helps avoiding this problem.

To make explicit that there was no washout time for amputations, we added "[no washout period]" for amputations and major amputations in the 2nd paragraph of the Methods→Study population.

References

1. van Buuren S. Flexible Imputation of Missing Data. 2nd ed: Chapman and Hall/CRC; 2018.
2. Gerds TA, Schumacher M. Consistent Estimation of the Expected Brier Score in General Survival Models with Right-Censored Event Times. Biometrical Journal. 2006;48(6):1029-40.
3. Koller MT, Raatz H, Steyerberg EW, Wolbers M. Competing risks and the clinical community: irrelevance or ignorance? Statistics in Medicine. 2012;31(11-12):1089-97.
4. Austin PC, Fine JP. Practical recommendations for reporting Fine-Gray model analyses for competing risk data. Statistics in Medicine. 2017;36(27):4391-400.
5. Huitfeldt A. Is caviar a risk factor for being a millionaire? BMJ. 2016;355:i6536.
6. Zethelius B, Gudbjörnsdottir S, Eliasson B, Eeg-Olofsson K, Cederholm J. Level of physical activity associated with risk of cardiovascular diseases and mortality in patients with type-2 diabetes: report from the Swedish National Diabetes Register. European Journal of Preventive Cardiology. 2014;21(2):244-51.

VERSION 2 - REVIEW

**Reviewer**	**1**
**Name**	**Kelson, Zoe**
**Affiliation**	**University of Exeter, Mathematics**
**Date**	**30-Sep-2026**
**COI**	

Thanks to the authors for their responses, providing clarification and communicating their rationale.

Author response:

"To help demonstrate the robustness of the study results to the choice of missing data methods, we additionally conducted complete case analyses. The results were similar and lent themselves to the same conclusions as results using multiply imputed data. We did not include these results as they did not change substantive conclusions, and burdened the manuscript and supplement by doubling the number of figures and tables. "

Reviewer request:

Can the authors please briefly report this valuable sensitivity analysis in the article?

Author response:

"We did not find that adding physical activity to clinical indicators routinely assessed in clinical practice would improve the predictive performance of statistical models. Computing model performance metrics on all imputed datasets, by communicating the greater uncertainty that comes with imputation, would not change the substantive conclusions of the article, as this would just mean greater confidence intervals for results that are already not suggesting that physical activity has added prognostic value. Reanalysing all the imputed datasets to calculate performance measures would require substantial computing time. Based on previous analyses conducted on a randomly chosen imputed dataset for each outcome-landmark age combination, and based on the fact that multiple imputation generated 20 imputed datasets for each outcome-landmark age combination, we estimate that these additional analyses would require roughly: (i) 2 months for peripheral neuropathy/peripheral vascular disease, running the analyses in parallel on 16 cores, (ii) 3 weeks for foot ulcers, running the analyses in parallel on 12 cores, (iii) and 1 month for amputations, running the analyses in parallel on 5 cores. We do not deem these additional analyses to be justifiable given the above reasoning that the conclusions will remain the same"

Reviewer request:

Can this response to reviewer please be acknowledged and discussed in the article by the authors?

VERSION 2 - AUTHOR RESPONSE

Reviewer: 1
Prof. Zoe Kelson, University of Exeter
Comments to the Author:
Thanks to the authors for their responses, providing clarification and communicating their rationale.

Author response:
"To help demonstrate the robustness of the study results to the choice of missing data methods, we additionally conducted complete case analyses. The results were similar and lent themselves to the same conclusions as results using multiply imputed data. We did not include these results as they did not change substantive conclusions, and burdened the manuscript and supplement by doubling the

number of figures and tables. "
Reviewer request:
Can the authors please briefly report this valuable sensitivity analysis in the article?

> We added the following text in Methods⟶Statistical analysis⟶Multiple imputation: "To help demonstrate the robustness of the study results to the choice of missing data methods, we additionally conducted complete case analyses".
>
> At the end of the Results section, we added the following: "The results for complete case analyses were similar and lent themselves to the same conclusions as results using multiply imputed data".

Author response:
"We did not find that adding physical activity to clinical indicators routinely assessed in clinical practice would improve the predictive performance of statistical models. Computing model performance metrics on all imputed datasets, by communicating the greater uncertainty that comes with imputation, would not change the substantive conclusions of the article, as this would just mean greater confidence intervals for results that are already not suggesting that physical activity has added prognostic value. Reanalysing all the imputed datasets to calculate performance measures would require substantial computing time. Based on previous analyses conducted on a randomly chosen imputed dataset for each outcome-landmark age combination, and based on the fact that multiple imputation generated 20 imputed datasets for each outcome-landmark age combination, we estimate that these additional analyses would require roughly: (i) 2 months for peripheral neuropathy/peripheral vascular disease, running the analyses in parallel on 16 cores, (ii) 3 weeks for foot ulcers, running the analyses in parallel on 12 cores, (iii) and 1 month for amputations, running the analyses in parallel on 5 cores. We do not deem these additional analyses to be justifiable given the above reasoning that the conclusions will remain the same"
Reviewer request:
Can this response to reviewer please be acknowledged and discussed in the article by the authors?

> We included this response in a new "Bootstrapping" section in the Supplemental Material, and we refer to it at the end of the Methods section in the manuscript: "(see Supplement: Bootstrapping)" (7-8 lines before the start of the Results section).
